# Supplementary figures and images for: The non-vesicular cholesterol transporter GRAMD1C is a pan-coronavirus antiviral target (part 1 of 4)
Source: PLoS Biol. 2026 Apr 6;24(4):e3003736. doi: 10.1371/journal.pbio.3003736 (PMC13068348; doi:10.1371/journal.pbio.3003736)

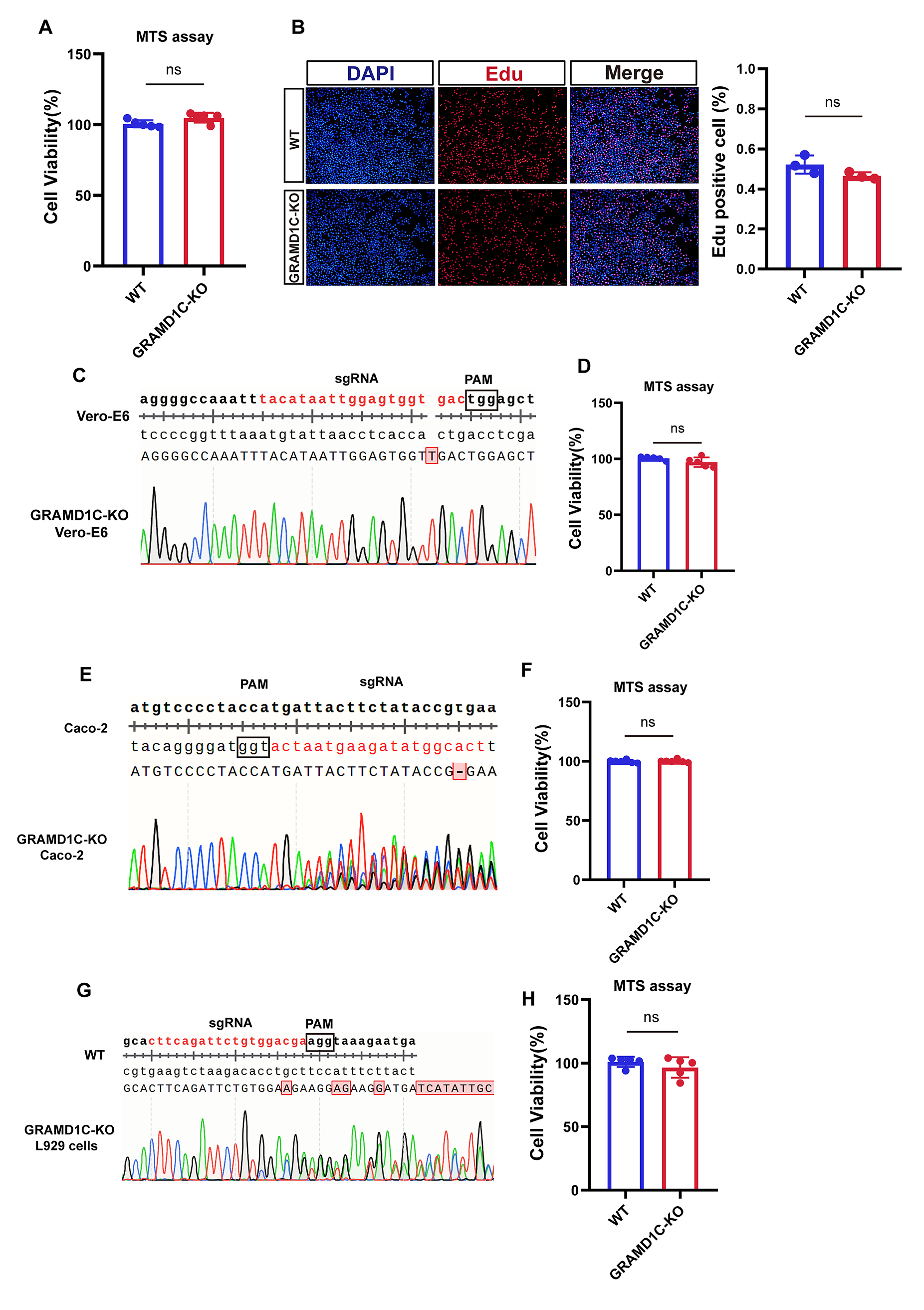

Supplement: S1 Fig — (A) GRAMD1C-KO PK-15 and WT cells were seeded into 96-well plates, and cell proliferation and viability were validated by MTS assay. (B) Proportion of EdU-positive cells between the GRAMD1C-KO PK-15 cells and WT cells. Left: representative pictures. Right: quantification of EdU-positive cells. (C) Genomic sequence analysis of GRAMD1C in GRAMD1C-KO Vero-E6 cells. (D) Cell viability of GRAMD1C-KO Vero-E6 cells was validated by MTS assay. (E) Genomic sequence analysis of GRAMD1C in GRAMD1C-KO Caco-2 cells. (F) Cell viability of GRAMD1C-KO Caco-2 cells was validated by MTS assay. (G) Genomic sequence analysis of GRAMD1C in GRAMD1C-KO L929 cells. (H) Cell viability of GRAMD1C-KO L929 cells was validated by MTS assay. The means and SDs of the results from three independent experiments are shown. ns, not significant. The data underlying this Figure can be found in S5 Data. (TIF) [file pbio.3003736.s001.tif]

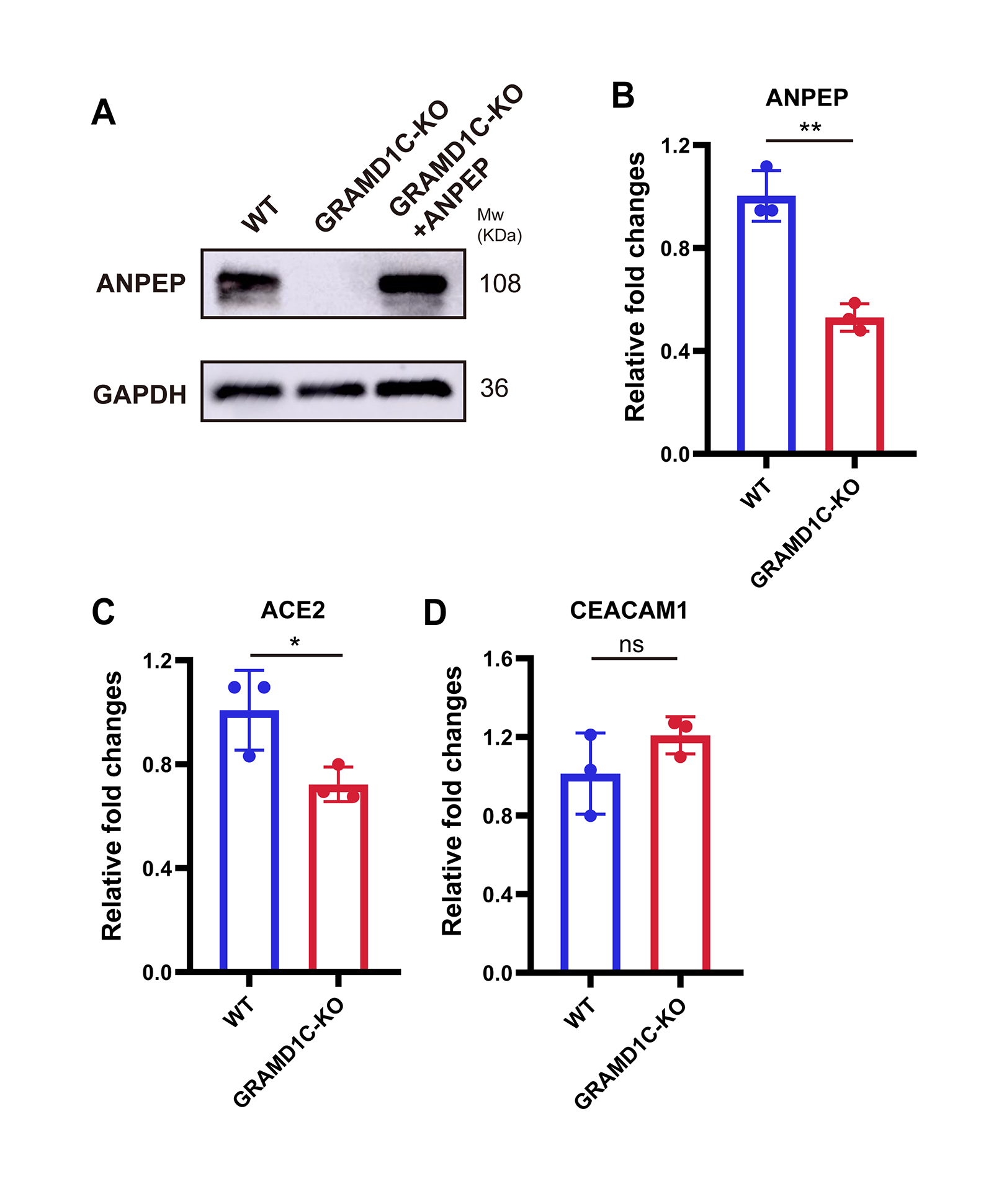

Supplement: S2 Fig — (A) western blot analysis validated the protein expression level of endogenous ANPEP in WT, KO, and GRAMD1C-rescued cells. (B) Relative ANPEP mRNA levels in WT and GRAMD1C-KO Caco-2 cells, normalized to β-actin. (C) Relative ACE2 mRNA levels in WT and GRAMD1C-KO Vero-E6 cells, normalized to β-actin. (D) Relative CEACAM1 mRNA levels in WT and GRAMD1C-KO L929 cells, normalized to β-actin. The data underlying this Figure can be found in S5 Data and S1 Raw Images. (TIF) [file pbio.3003736.s002.tif]

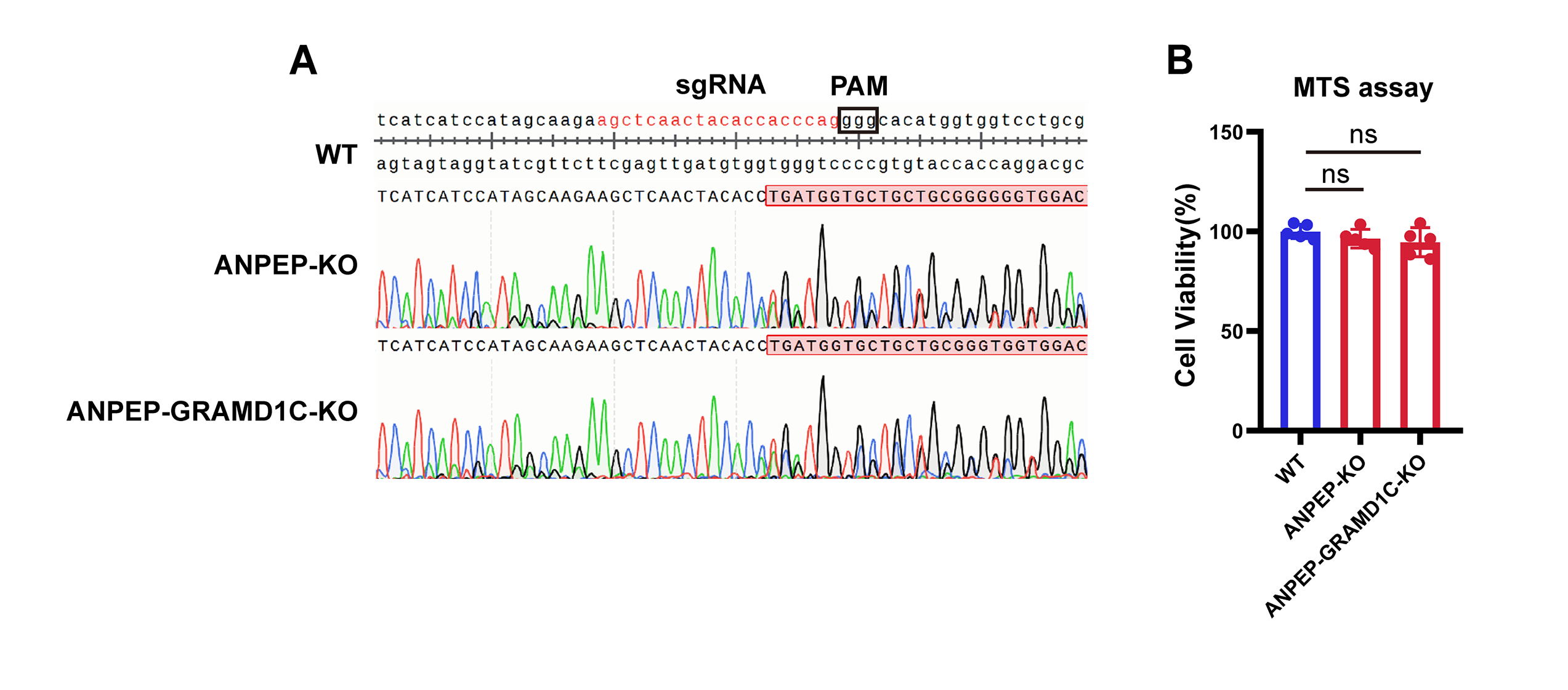

Supplement: S3 Fig — (A) Genomic sequence analysis of ANPEP in ANPEP-KO and ANPEP-GRAMD1C-KO cells. (B) Cell viability of ANPEP-KO and ANPEP-GRAMD1C-KO cells assessed by MTS assay. The data underlying this Figure can be found in S5 Data. (TIF) [file pbio.3003736.s003.tif]

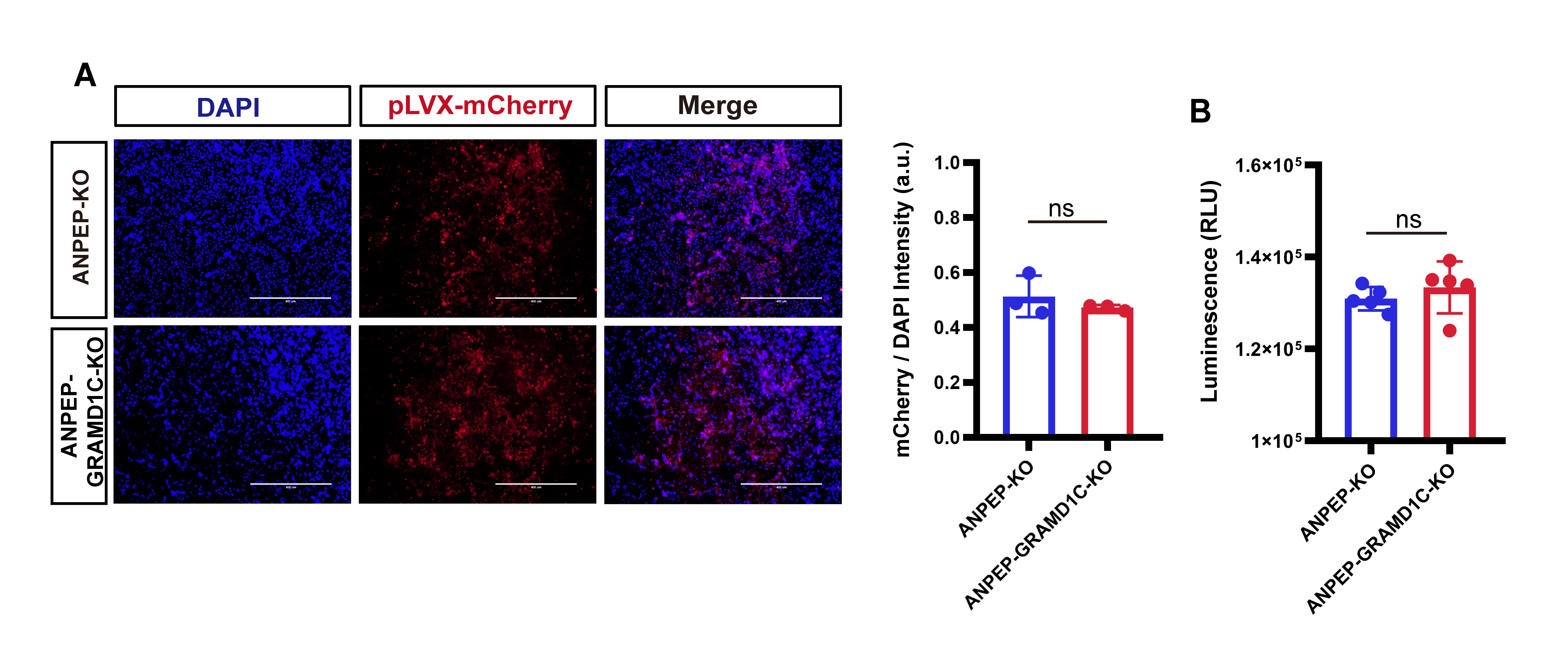

Supplement: S4 Fig — (A) The indicated KO cell lines were transfected with a pLVX-mCherry control plasmid. Left: representative images. Scale bar,400μm. Right: quantification of mCherry-positive) cells. (B) Cells were seeded at equal density and transfected with the pRL-TK plasmid. Luciferase activity, measured at 465 nm, was determined in ANPEP-KO and ANPEP-GRAMD1C-DKO cells to control for transfection efficiency. The data underlying this Figure can be found in S5 Data. (TIF) [file pbio.3003736.s004.tif]

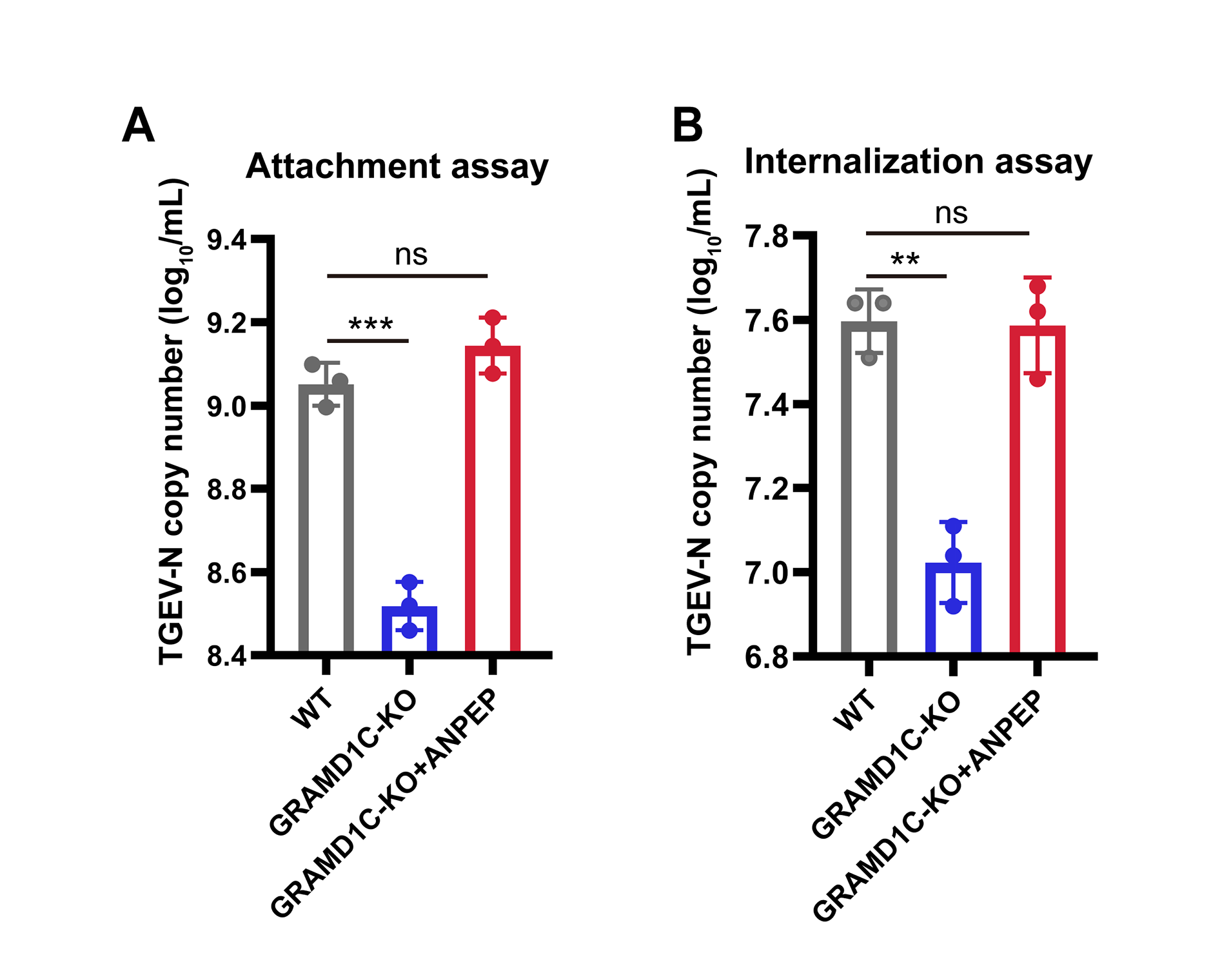

Supplement: S5 Fig — (A) WT and GRAMD1C-KO cells were infected with TGEV (MOI = 50) at 4 °C for 1 h and assessed for TGEV adsorption. The cells were harvested, and viral RNA was extracted to determine virion attachment at the cell surface. (B) WT and GRAMD1C-KO cells were infected with TGEV (MOI = 50) at 4 °C for 1 hour, followed by incubation at 37 °C for 30 min. The internalization was evaluated by absolute RT-qPCR. The data underlying this Figure can be found in S5 Data. (TIF) [file pbio.3003736.s005.tif]

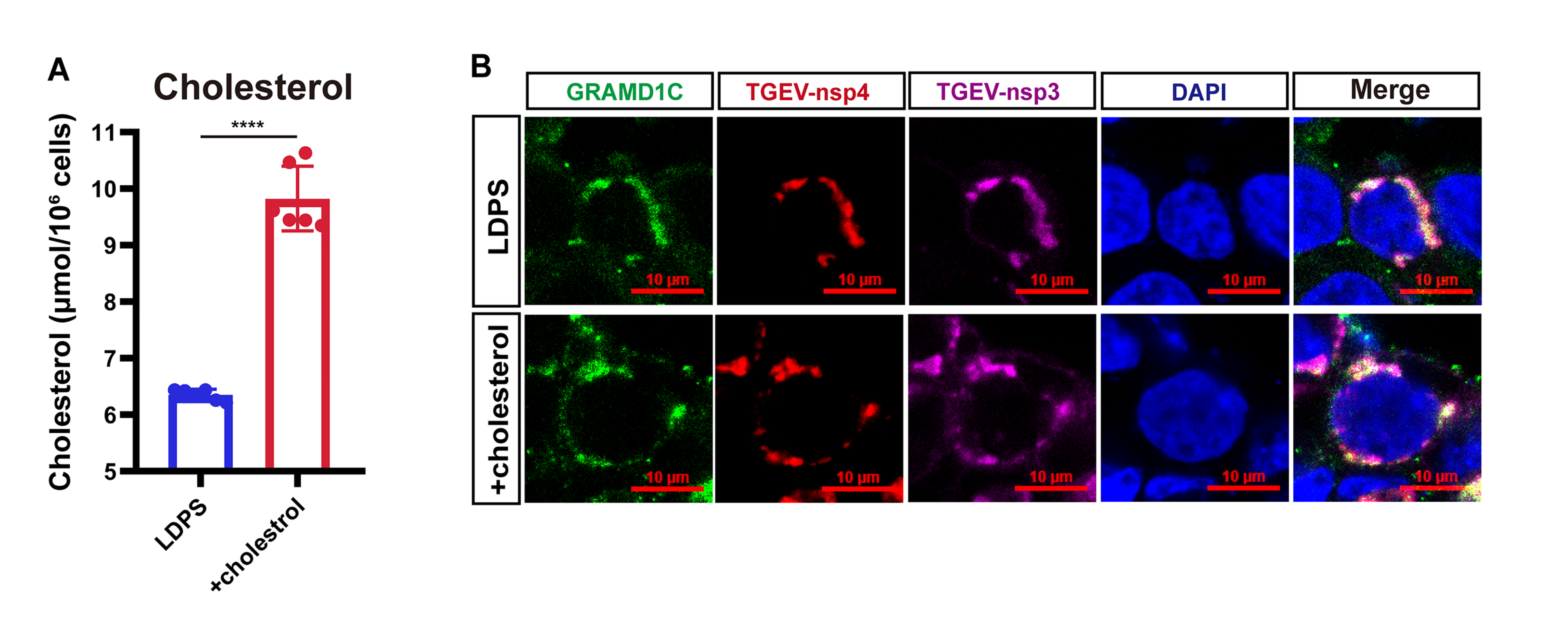

Supplement: S6 Fig — (A) Quantification of intracellular free cholesterol after cholesterol loaded. (B) Cells transfected with GRAMD1C and TGEV-nsp3/4 plasmids were switched to 5%LPDS medium ± 200 μM cholesterol for 1 hour prior to fixation and staining. The data underlying this Figure can be found in S5 Data. (TIF) [file pbio.3003736.s006.tif]

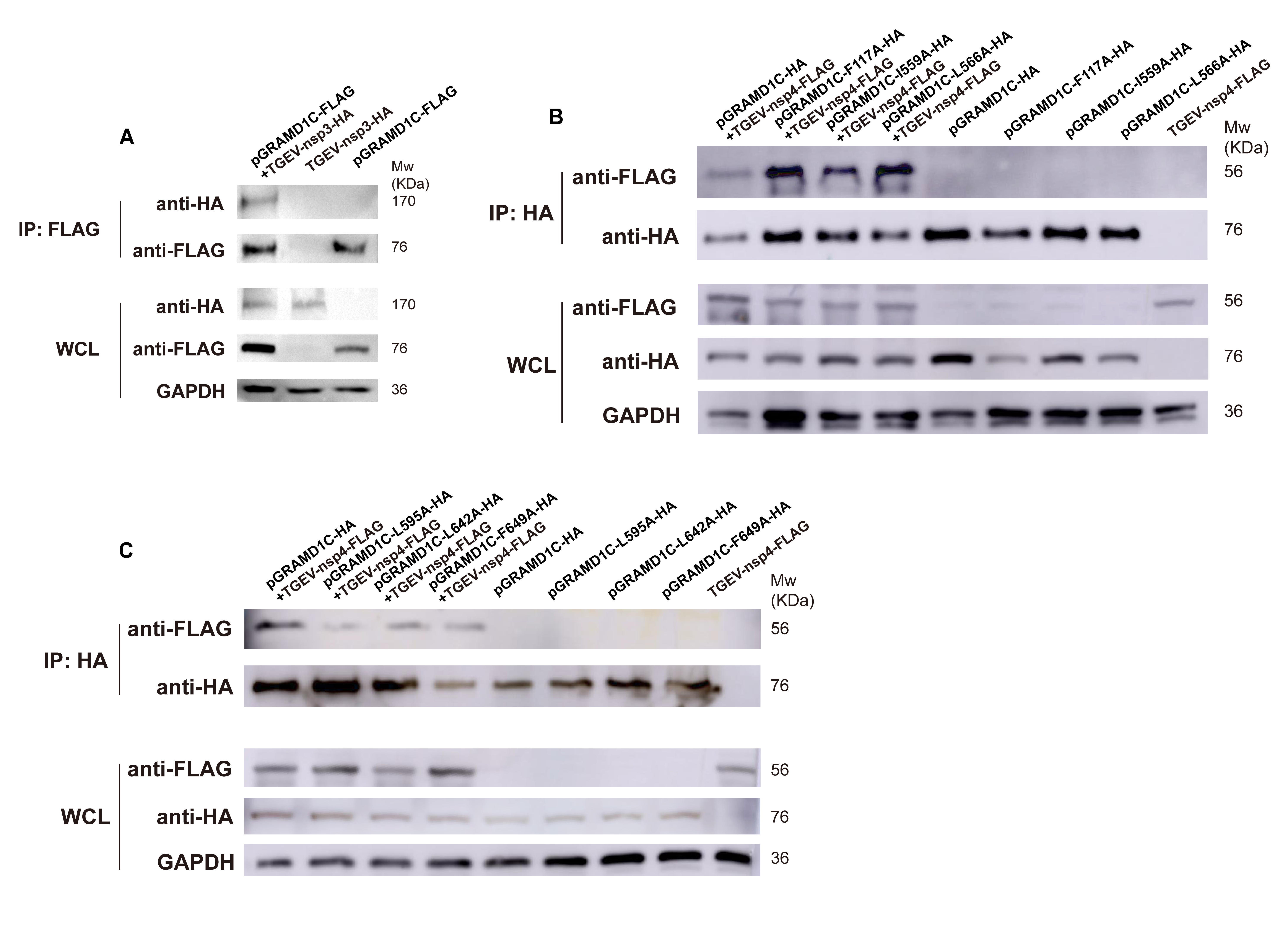

Supplement: S7 Fig — (A) Co-IP analysis of GRAMD1C point mutants (F117A, I559A, L566A) with TGEV-nsp4. (B) Co-IP analysis of GRAMD1C point mutants (L595A, L642A, F649A) with TGEV-nsp4. (C) Co-IP assay was performed in HEK293T cells transfected with pCAGGS-GRAMD1C-FLAG and pCAGGS-TGEV-nsp3-HA for 24 hours. The data underlying this Figure can be found in S1 Raw Images. (TIF) [file pbio.3003736.s007.tif]

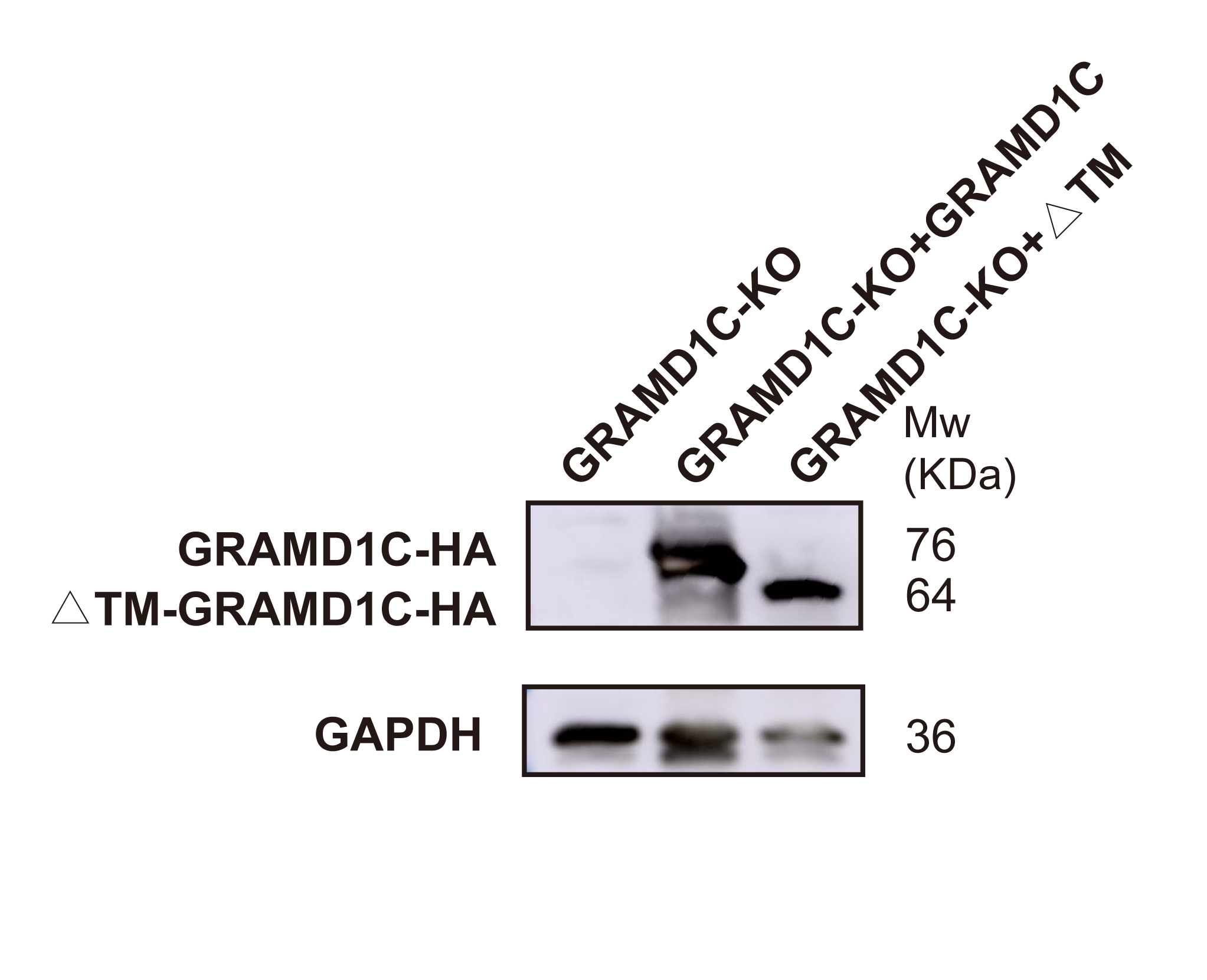

Supplement: S8 Fig — Western blot confirmed the successful rescue of full-length GRAMD1C and △TM-GRAMD1C in GRAMD1C-KO cells. GAPDH used as an internal control gene. The data underlying this Figure can be found in S1 Raw Images. (TIF) [file pbio.3003736.s008.tif]

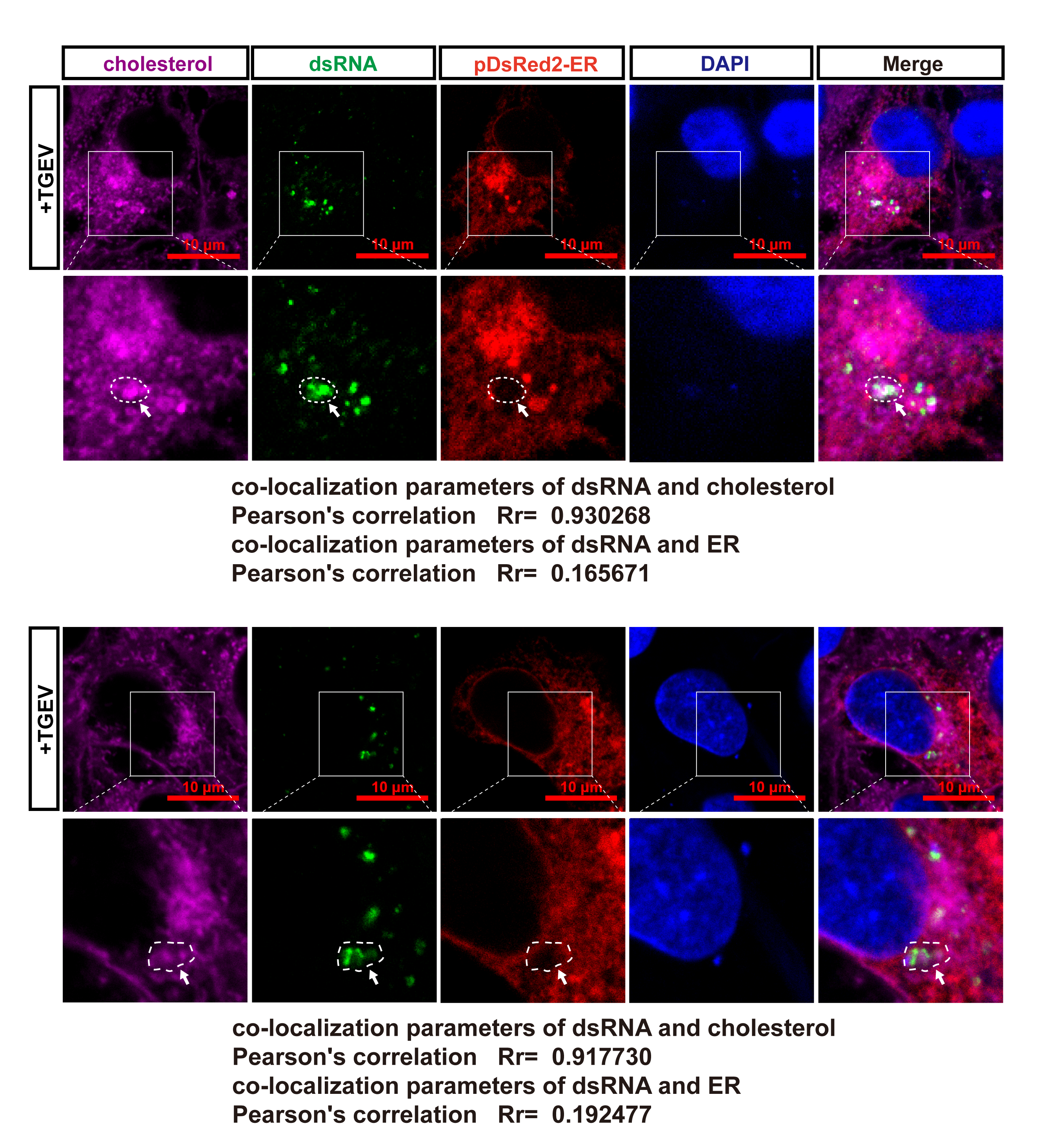

Supplement: S9 Fig — Two additional exemplary cells of Fig 4C. Confocal fluorescence microscopy analysis of the co-localization of free cholesterol and TGEV dsRNA in WT cells mock-infected or infected with TGEV (MOI = 0.1), at 12 hpi. Scale bars, 10 µm. (TIF) [file pbio.3003736.s009.tif]

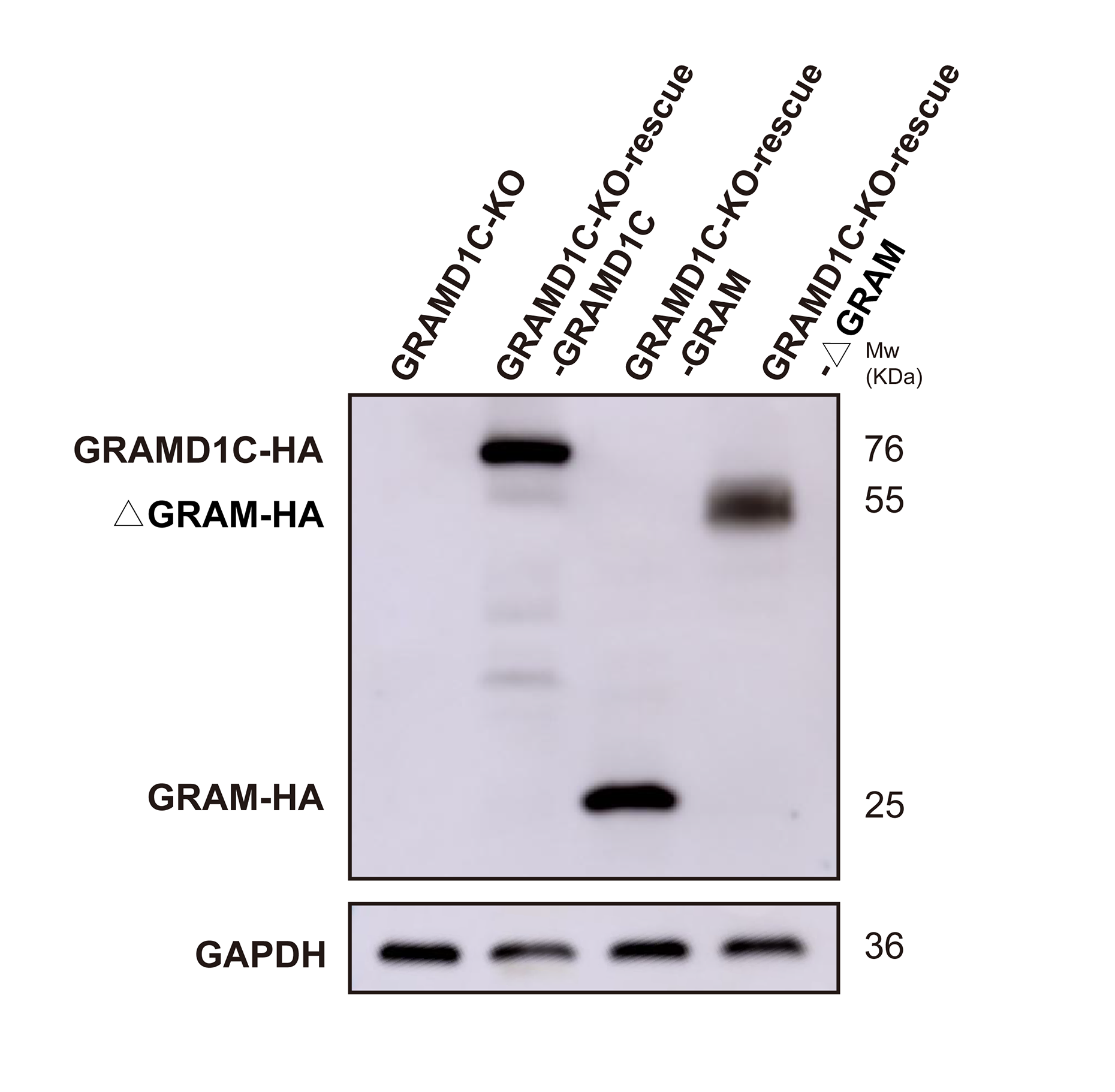

Supplement: S10 Fig — Western blot confirmed the successful rescue of full-length GRAMD1C and different domains in GRAMD1C-KO cells. GAPDH is used as an internal control gene. The data underlying this Figure can be found in S1 Raw Images. (TIF) [file pbio.3003736.s010.tif]

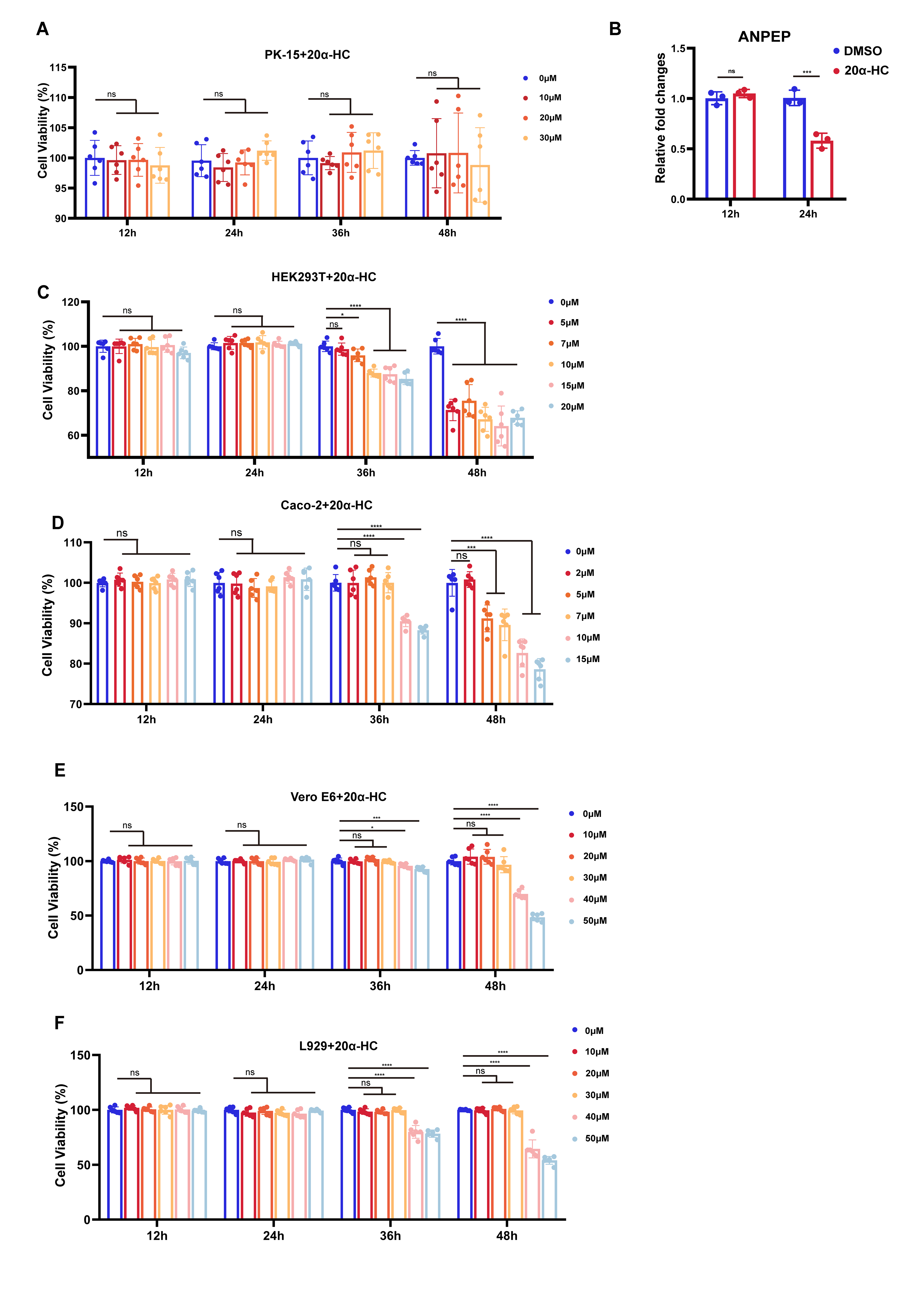

Supplement: S11 Fig — (A) PK-15 cells were treated with the indicated concentrations of 20α-HC for different time points prior to MTS analysis. (B) RT-qPCR assay for determination of relative mRNA levels of ANPEP in WT and 20α-HC-treated cells at indicated time points. (C) HEK-293T cells were treated with the indicated concentrations of 20α-HC for different time points prior to MTS analysis. (D) Caco-2 cells were treated with the indicated concentrations of 20α-HC for different time points prior to MTS analysis. (E) Vero-E6 cells were treated with the indicated concentrations of 20α-HC for different time points prior to MTS analysis. (F) L929 cells were treated with the indicated concentrations of 20α-HC for different time points prior to MTS analysis. The data underlying this Figure can be found in S5 Data. (TIF) [file pbio.3003736.s011.tif]

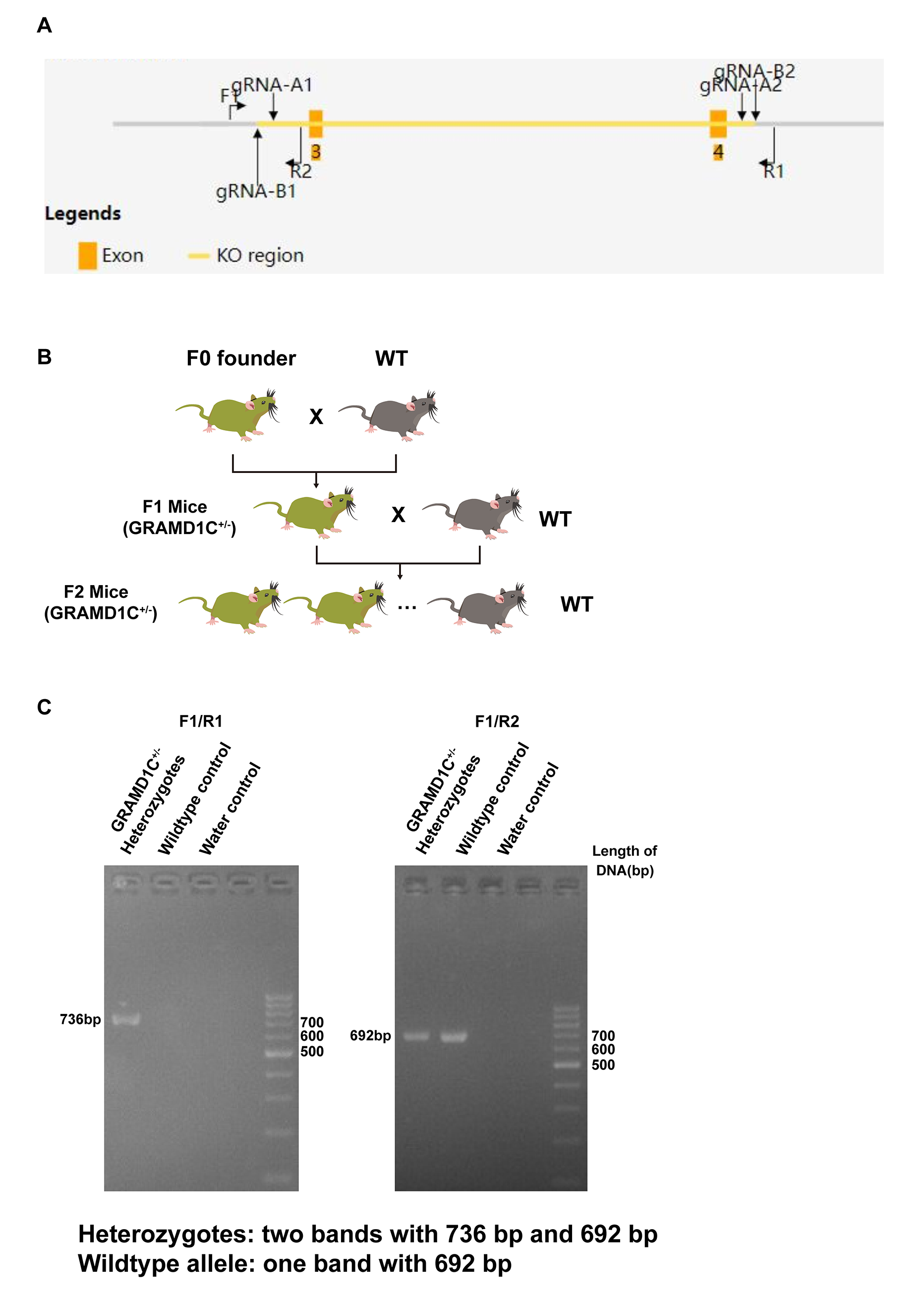

Supplement: S12 Fig — (A) Targeting Strategy of two sgRNAs of GRAMD1C-deficient mice. (B) A schematic diagram of generation of F2 GRAMD1C -deficient mice. (C) The results of tail piece (2–5 mm) PCR detection were consistent with the expected genetic profile of the mouse strain. Heterozygotes: two bands with 736 bp and 692 bp, Wild-type allele: one band with 692 bp. (TIF) [file pbio.3003736.s012.tif]

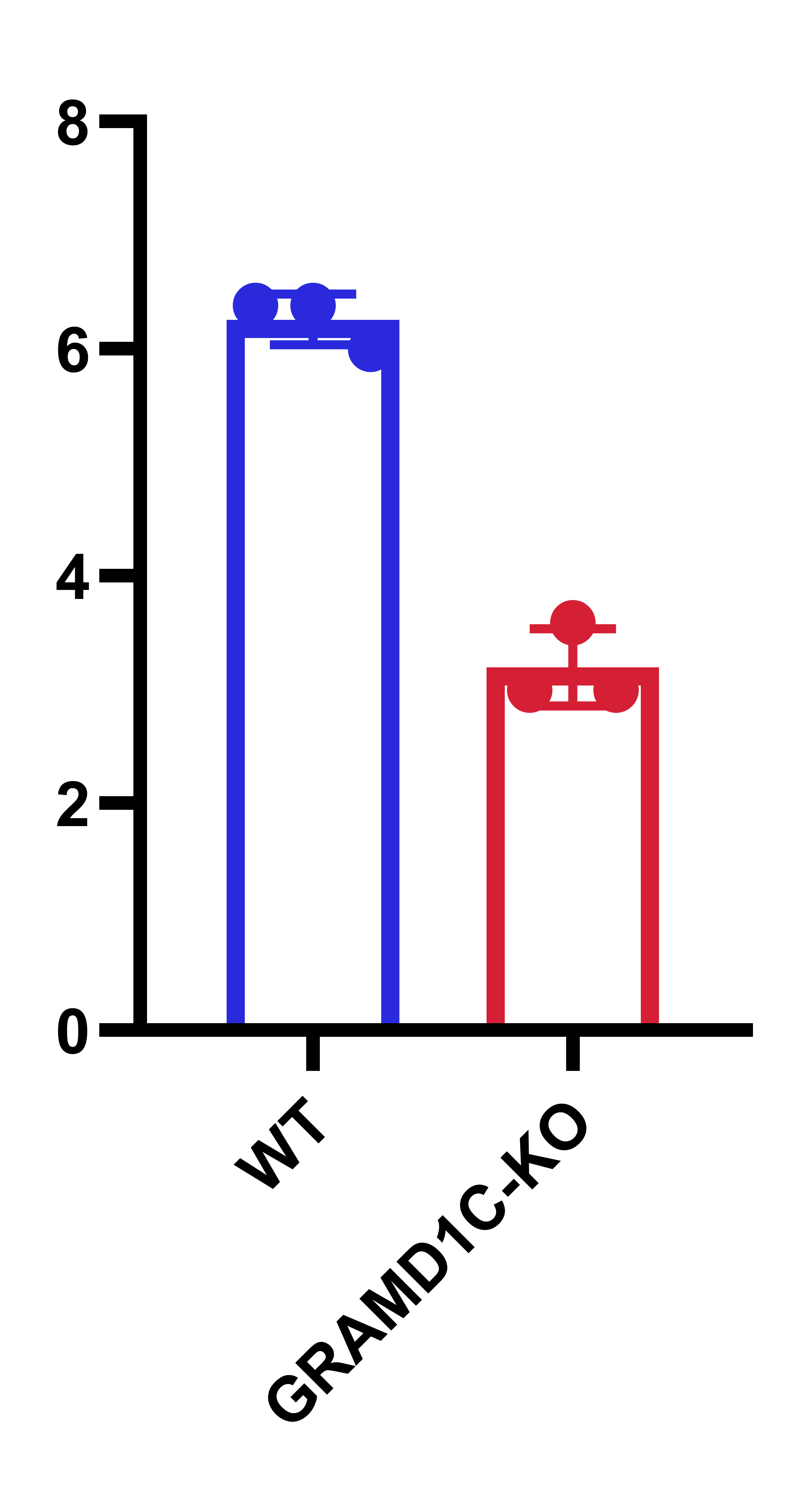

Supplement: S1 Data — This compressed folder contains the underlying numerical data and/or uncropped images used to generate the panels in Fig 1. (ZIP) [file pbio.3003736.s015.zip › S1 Data/Figure 1/H/PEDV-DR13-viral-titer/viral-titer-PEDV-DR13.tif]

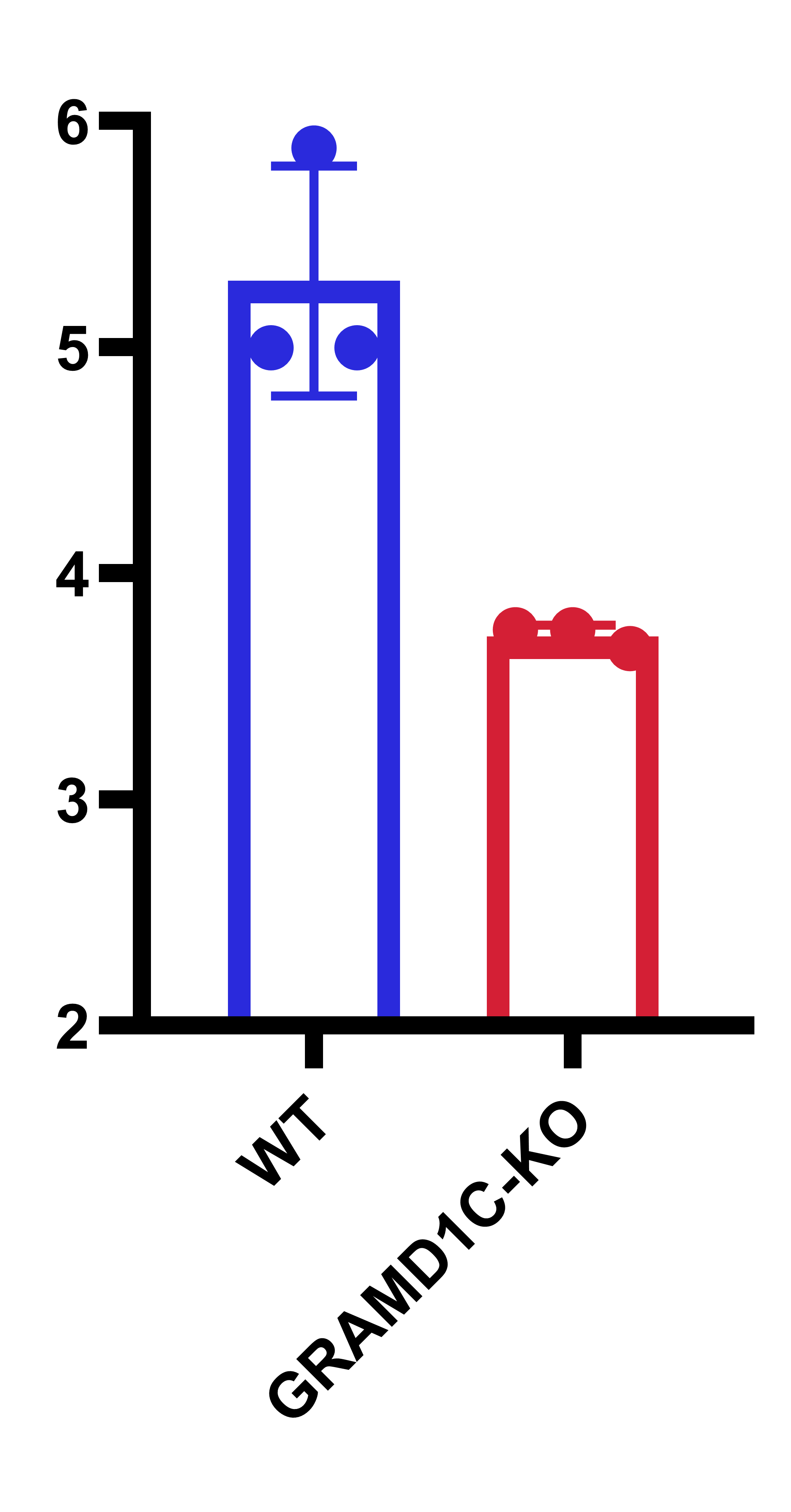

Supplement: S1 Data — This compressed folder contains the underlying numerical data and/or uncropped images used to generate the panels in Fig 1. (ZIP) [file pbio.3003736.s015.zip › S1 Data/Figure 1/I/229E-Viral-titer/titer.tif]

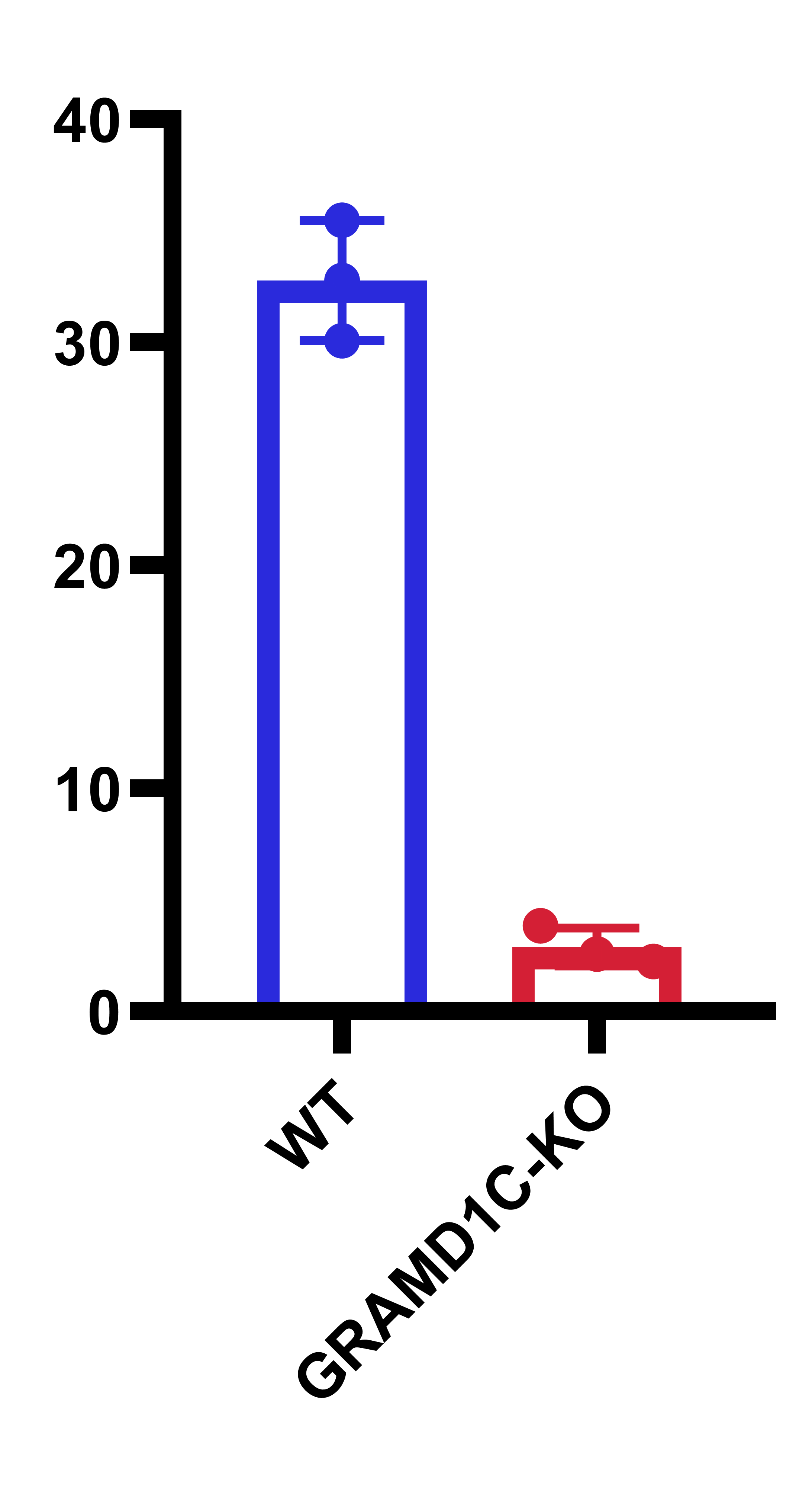

Supplement: S1 Data — This compressed folder contains the underlying numerical data and/or uncropped images used to generate the panels in Fig 1. (ZIP) [file pbio.3003736.s015.zip › S1 Data/Figure 1/I/IFA/229e.tif]

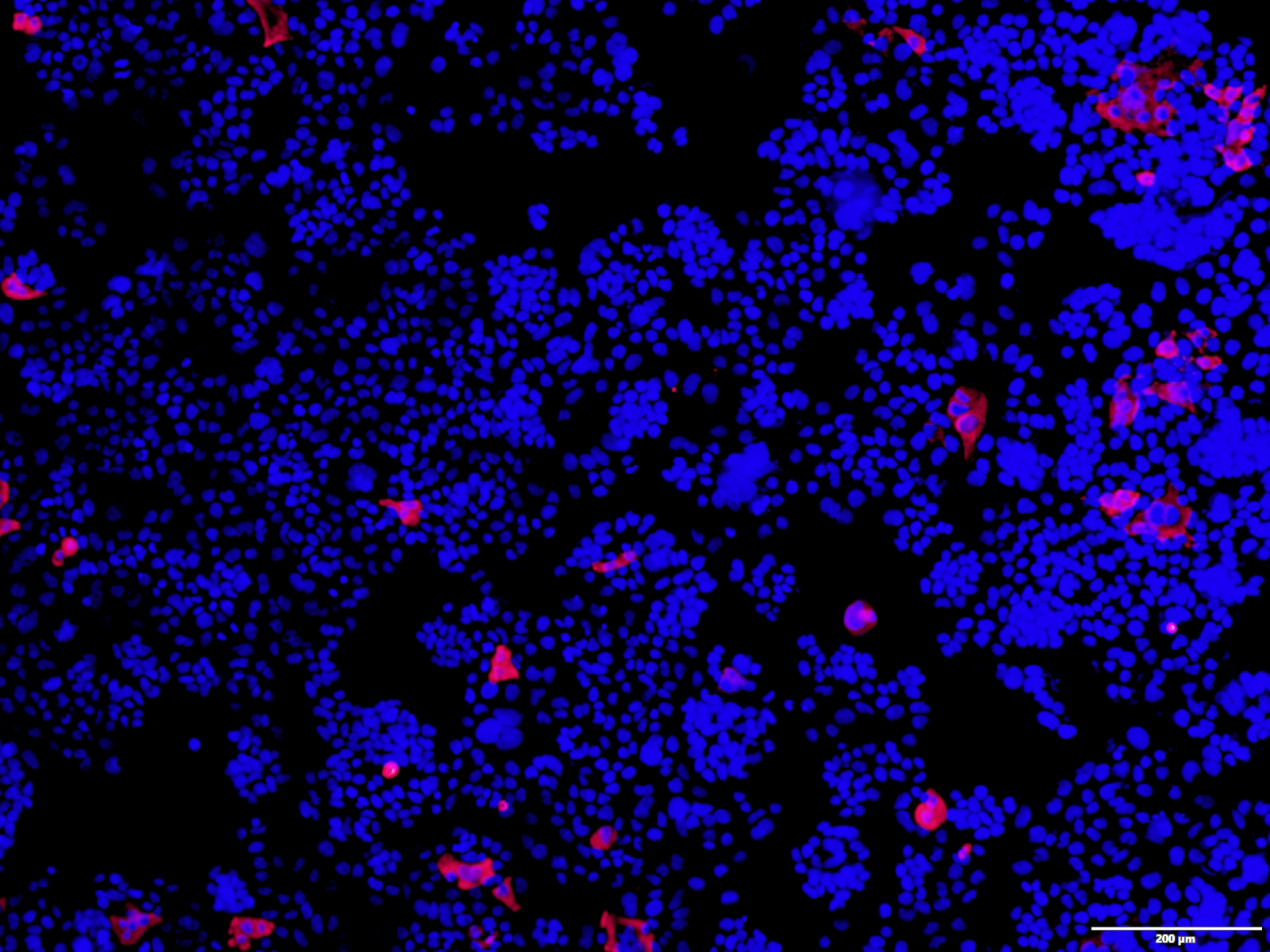

Supplement: S1 Data — This compressed folder contains the underlying numerical data and/or uncropped images used to generate the panels in Fig 1. (ZIP) [file pbio.3003736.s015.zip › S1 Data/Figure 1/I/IFA/CACO2-KO/KO/229E-1.png]

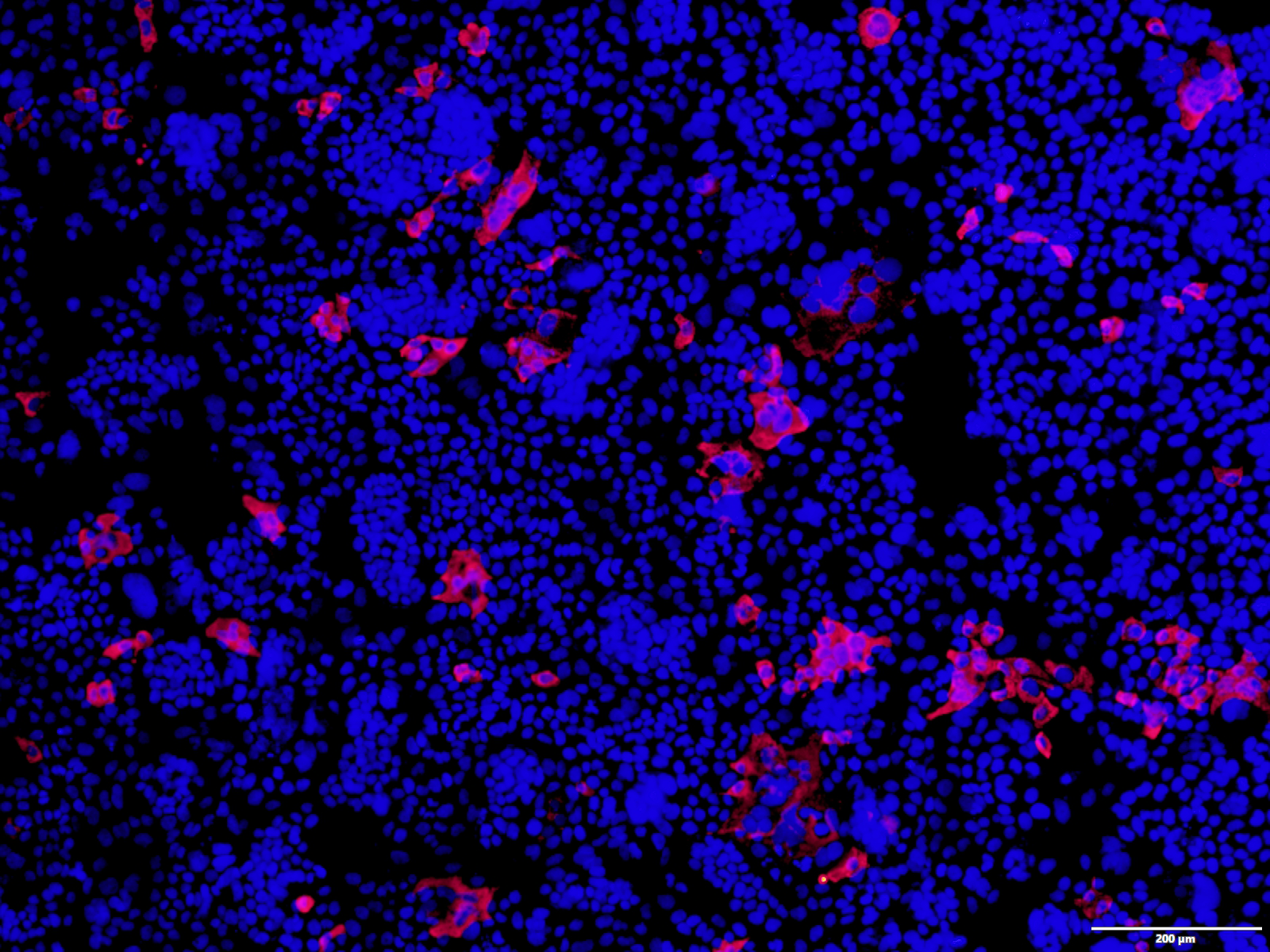

Supplement: S1 Data — This compressed folder contains the underlying numerical data and/or uncropped images used to generate the panels in Fig 1. (ZIP) [file pbio.3003736.s015.zip › S1 Data/Figure 1/I/IFA/CACO2-KO/KO/229E-2.png]

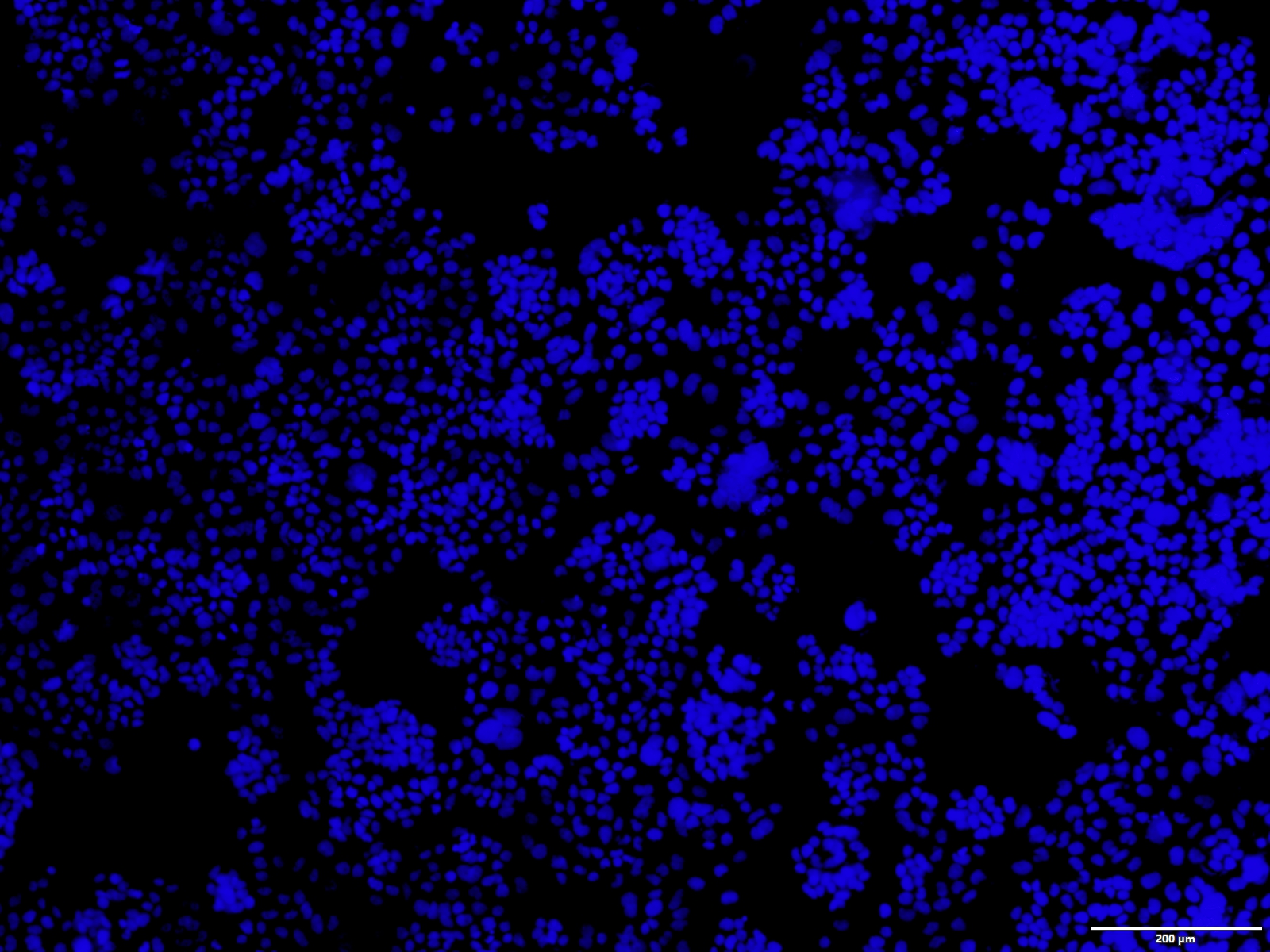

Supplement: S1 Data — This compressed folder contains the underlying numerical data and/or uncropped images used to generate the panels in Fig 1. (ZIP) [file pbio.3003736.s015.zip › S1 Data/Figure 1/I/IFA/CACO2-KO/KO/229E-DAPI-1.jpg]

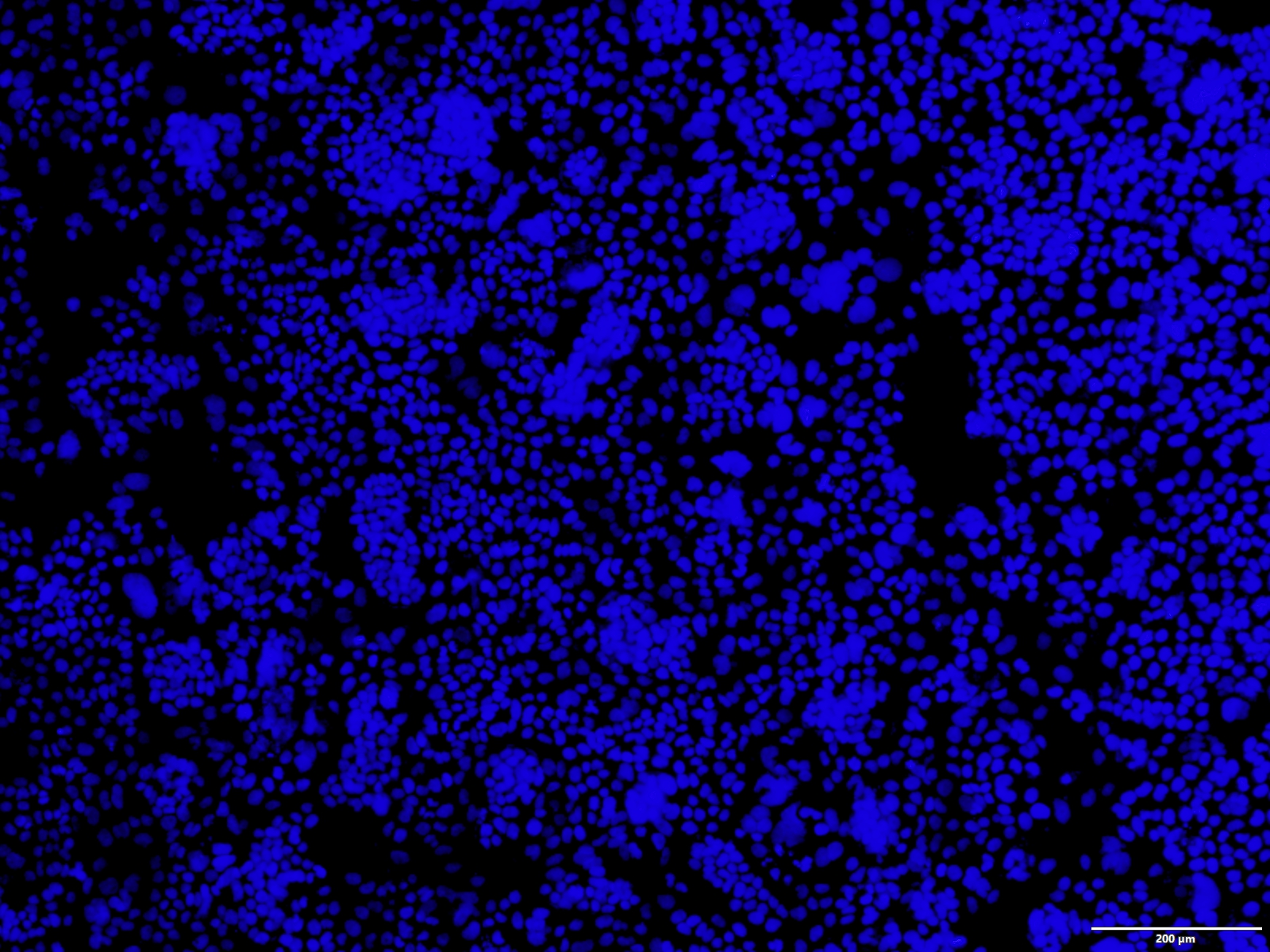

Supplement: S1 Data — This compressed folder contains the underlying numerical data and/or uncropped images used to generate the panels in Fig 1. (ZIP) [file pbio.3003736.s015.zip › S1 Data/Figure 1/I/IFA/CACO2-KO/KO/229E-DAPI-2.jpg]

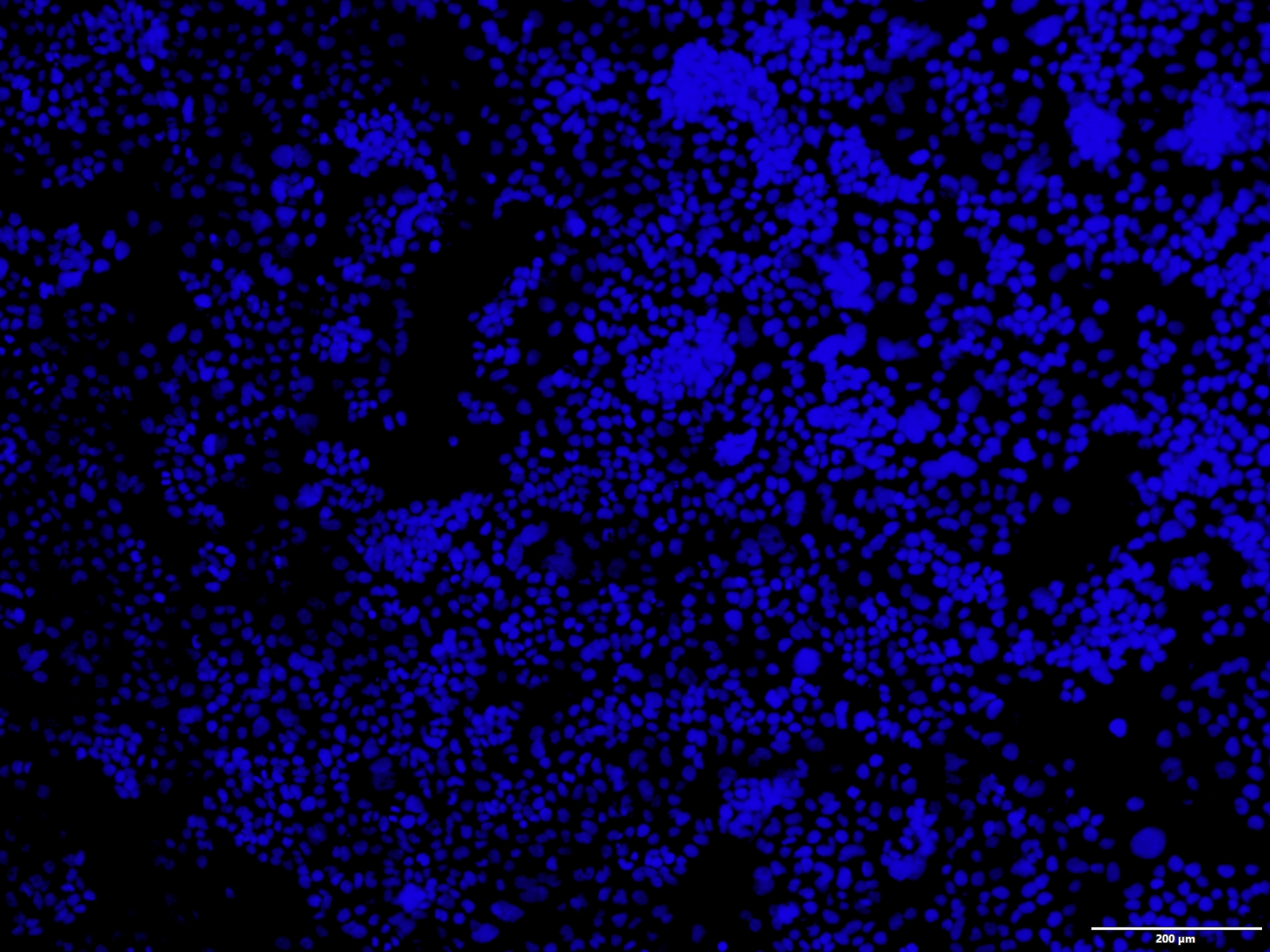

Supplement: S1 Data — This compressed folder contains the underlying numerical data and/or uncropped images used to generate the panels in Fig 1. (ZIP) [file pbio.3003736.s015.zip › S1 Data/Figure 1/I/IFA/CACO2-KO/KO/229E-DAPI-3.jpg]

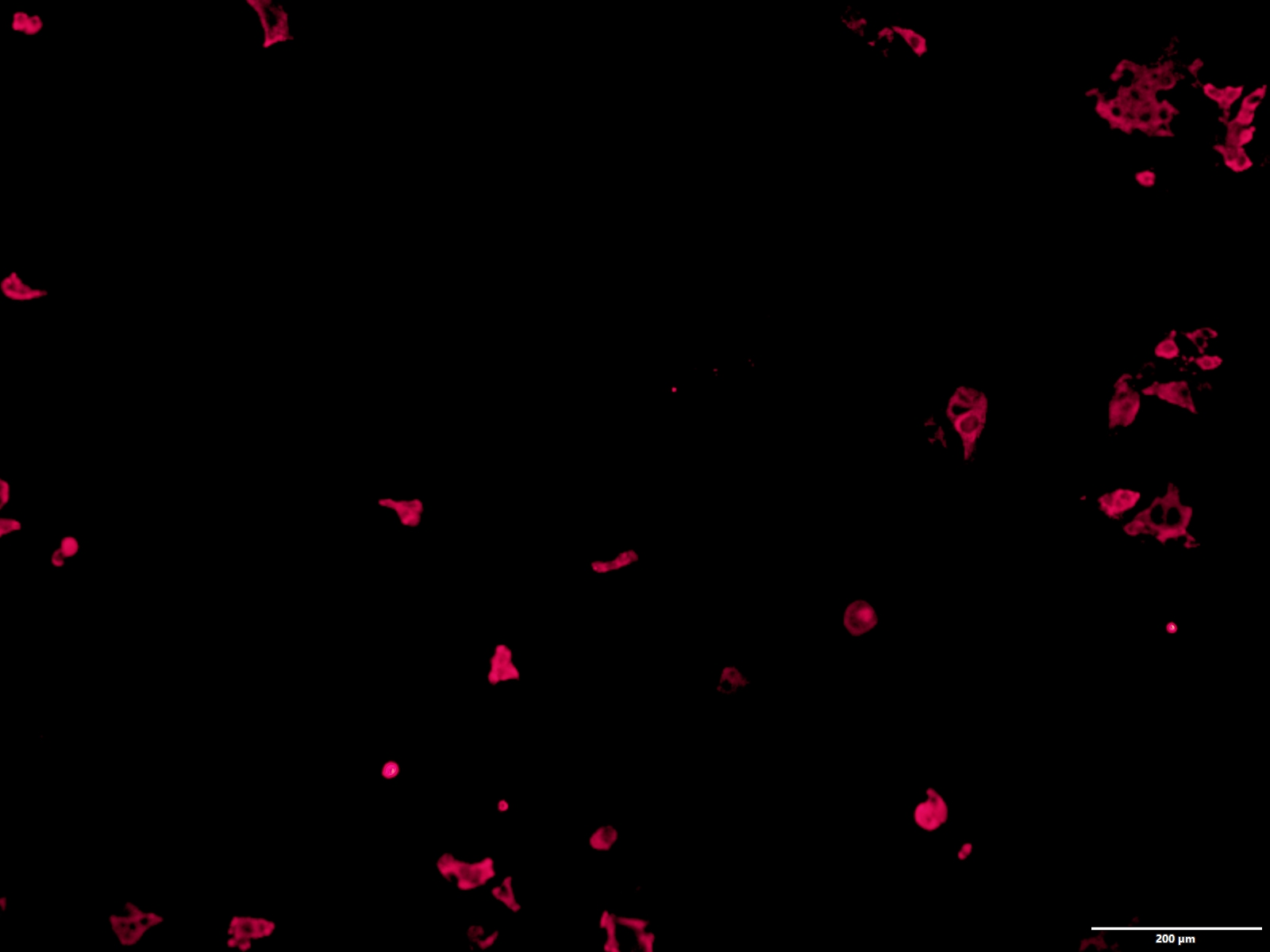

Supplement: S1 Data — This compressed folder contains the underlying numerical data and/or uncropped images used to generate the panels in Fig 1. (ZIP) [file pbio.3003736.s015.zip › S1 Data/Figure 1/I/IFA/CACO2-KO/KO/229E-N-1.jpg]

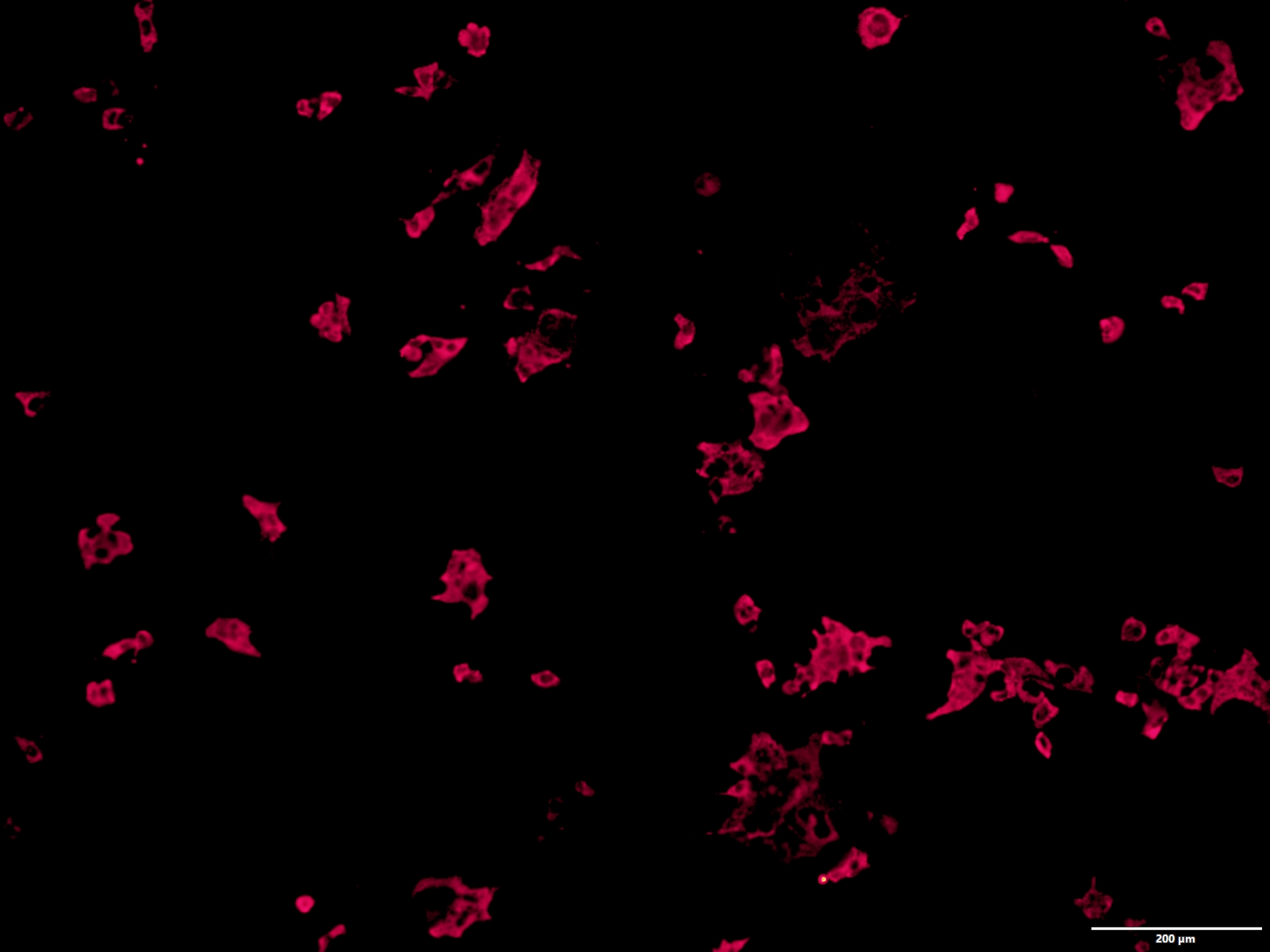

Supplement: S1 Data — This compressed folder contains the underlying numerical data and/or uncropped images used to generate the panels in Fig 1. (ZIP) [file pbio.3003736.s015.zip › S1 Data/Figure 1/I/IFA/CACO2-KO/KO/229E-N-2.jpg]

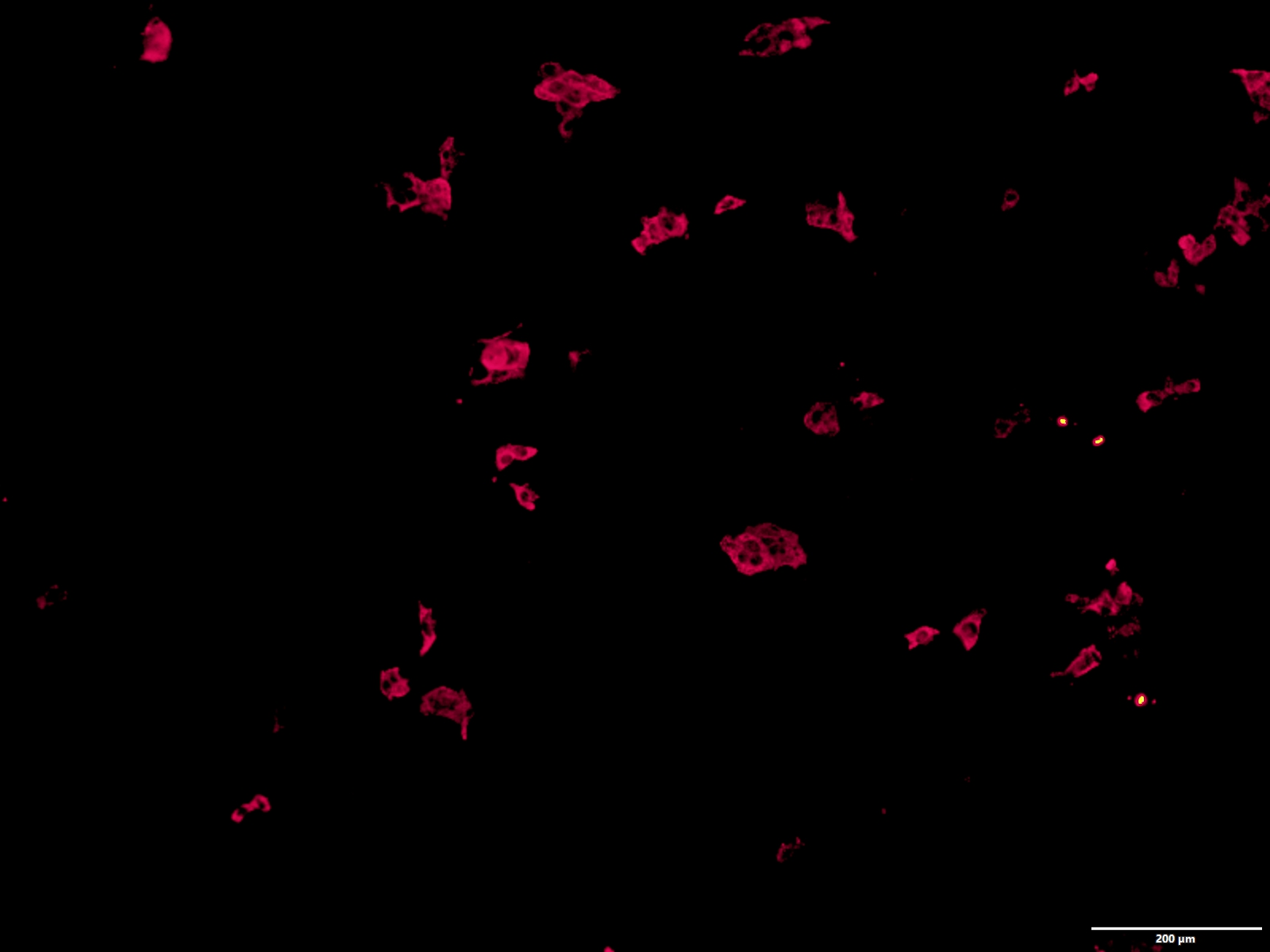

Supplement: S1 Data — This compressed folder contains the underlying numerical data and/or uncropped images used to generate the panels in Fig 1. (ZIP) [file pbio.3003736.s015.zip › S1 Data/Figure 1/I/IFA/CACO2-KO/KO/229E-N-3.jpg]

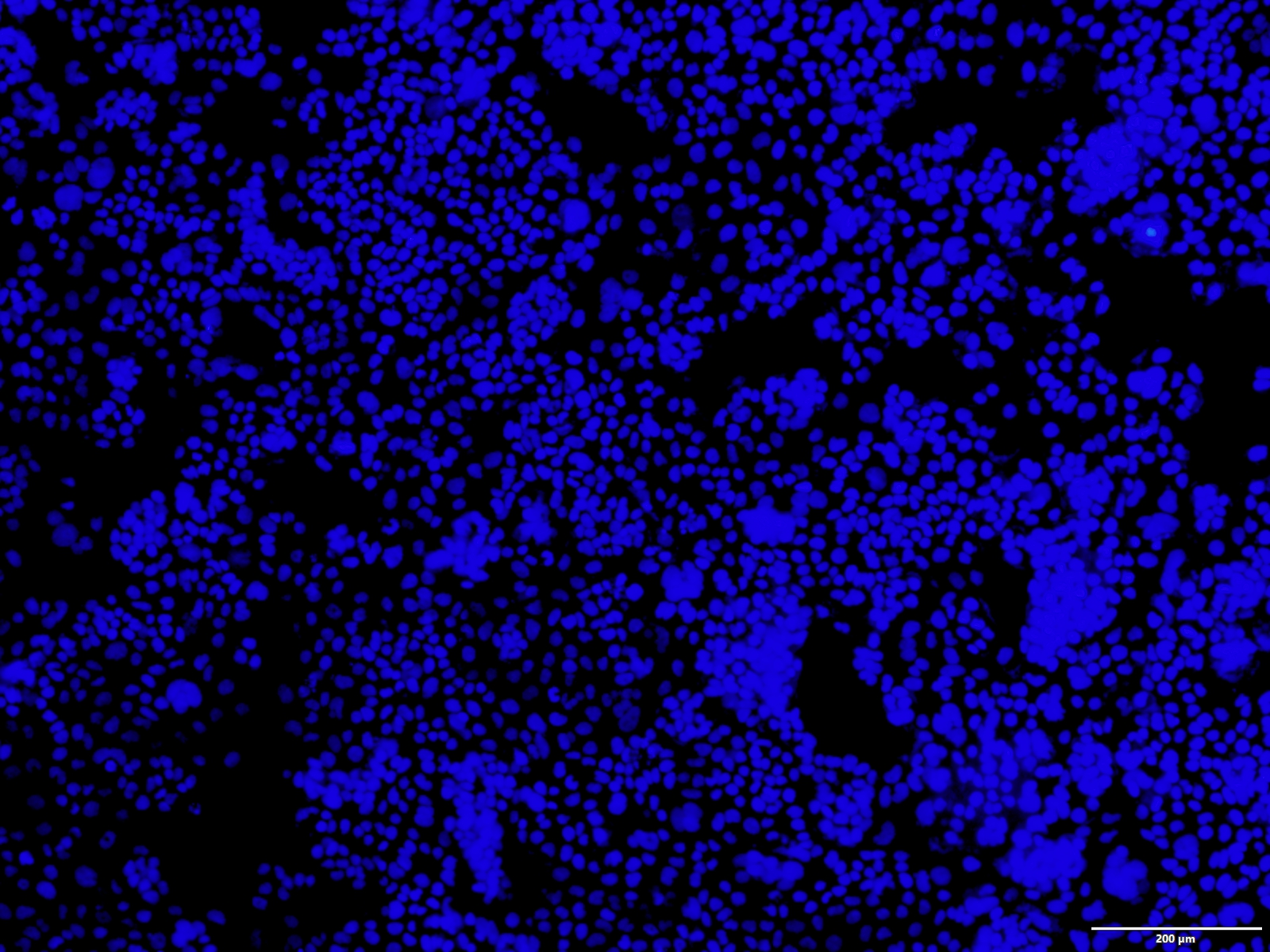

Supplement: S1 Data — This compressed folder contains the underlying numerical data and/or uncropped images used to generate the panels in Fig 1. (ZIP) [file pbio.3003736.s015.zip › S1 Data/Figure 1/I/IFA/caco2-WT/WT/229E-DAPI-5.jpg]

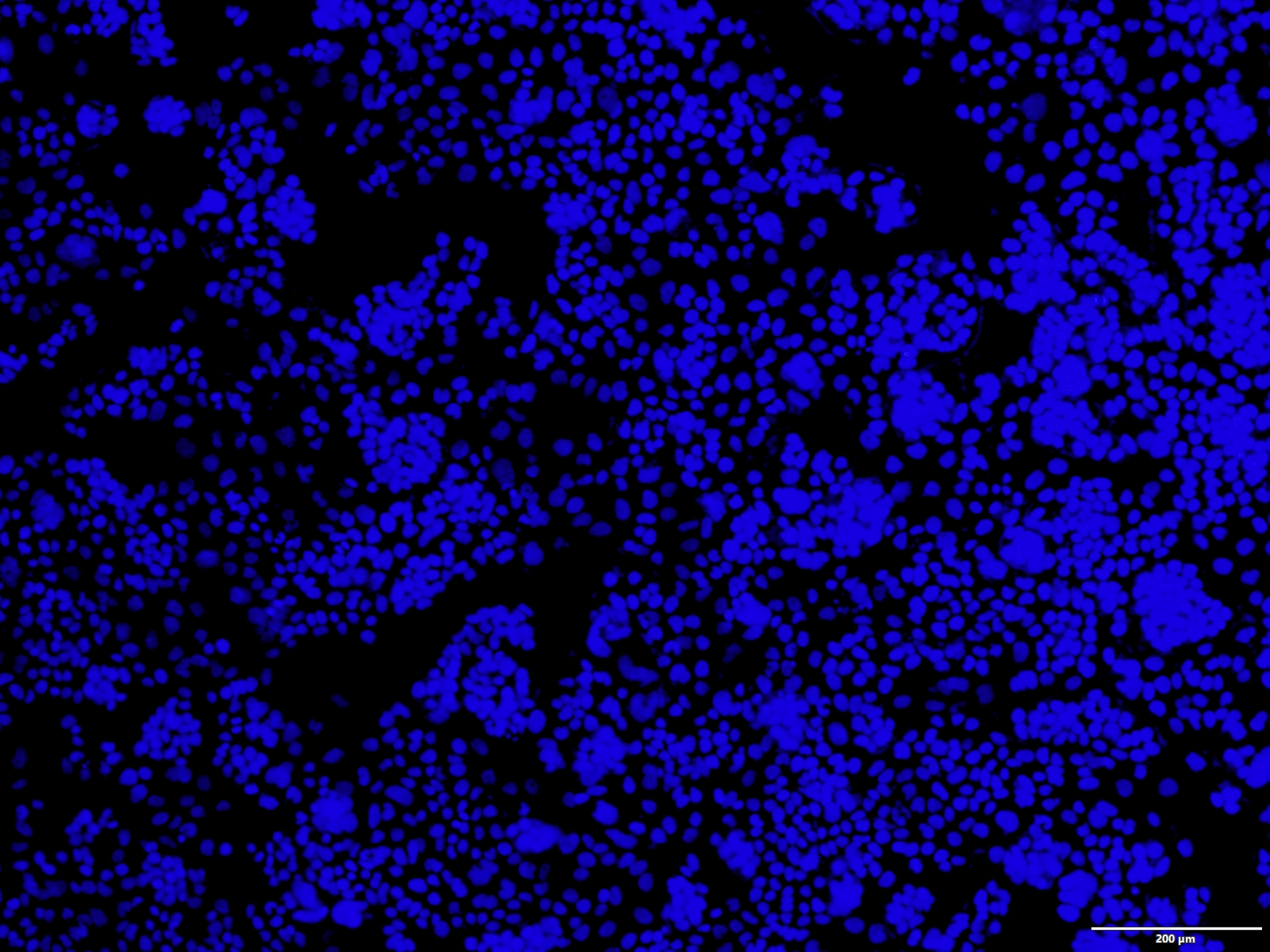

Supplement: S1 Data — This compressed folder contains the underlying numerical data and/or uncropped images used to generate the panels in Fig 1. (ZIP) [file pbio.3003736.s015.zip › S1 Data/Figure 1/I/IFA/caco2-WT/WT/229E-DAPI-6.jpg]

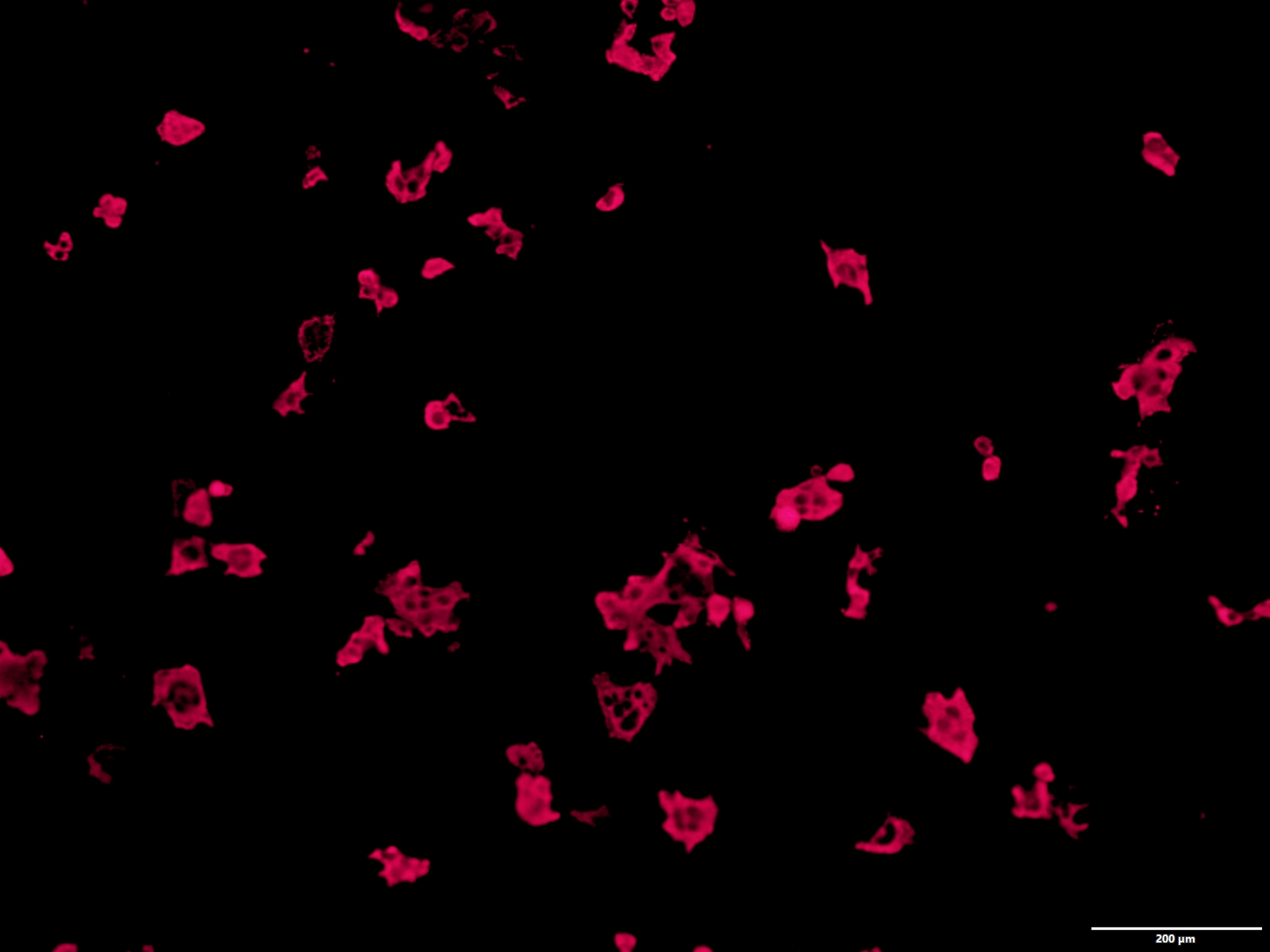

Supplement: S1 Data — This compressed folder contains the underlying numerical data and/or uncropped images used to generate the panels in Fig 1. (ZIP) [file pbio.3003736.s015.zip › S1 Data/Figure 1/I/IFA/caco2-WT/WT/229E-N-5.jpg]

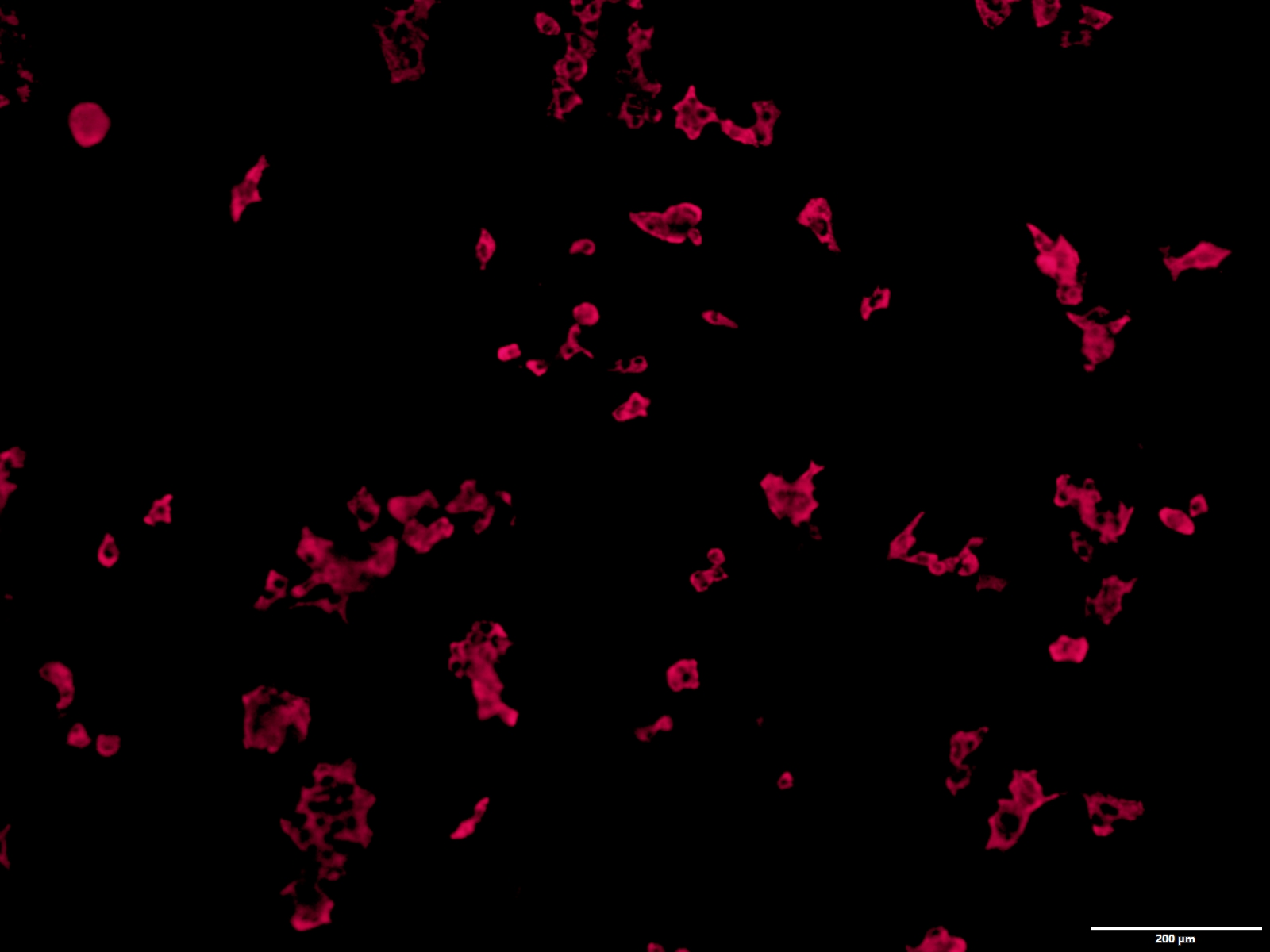

Supplement: S1 Data — This compressed folder contains the underlying numerical data and/or uncropped images used to generate the panels in Fig 1. (ZIP) [file pbio.3003736.s015.zip › S1 Data/Figure 1/I/IFA/caco2-WT/WT/229E-N-6.jpg]

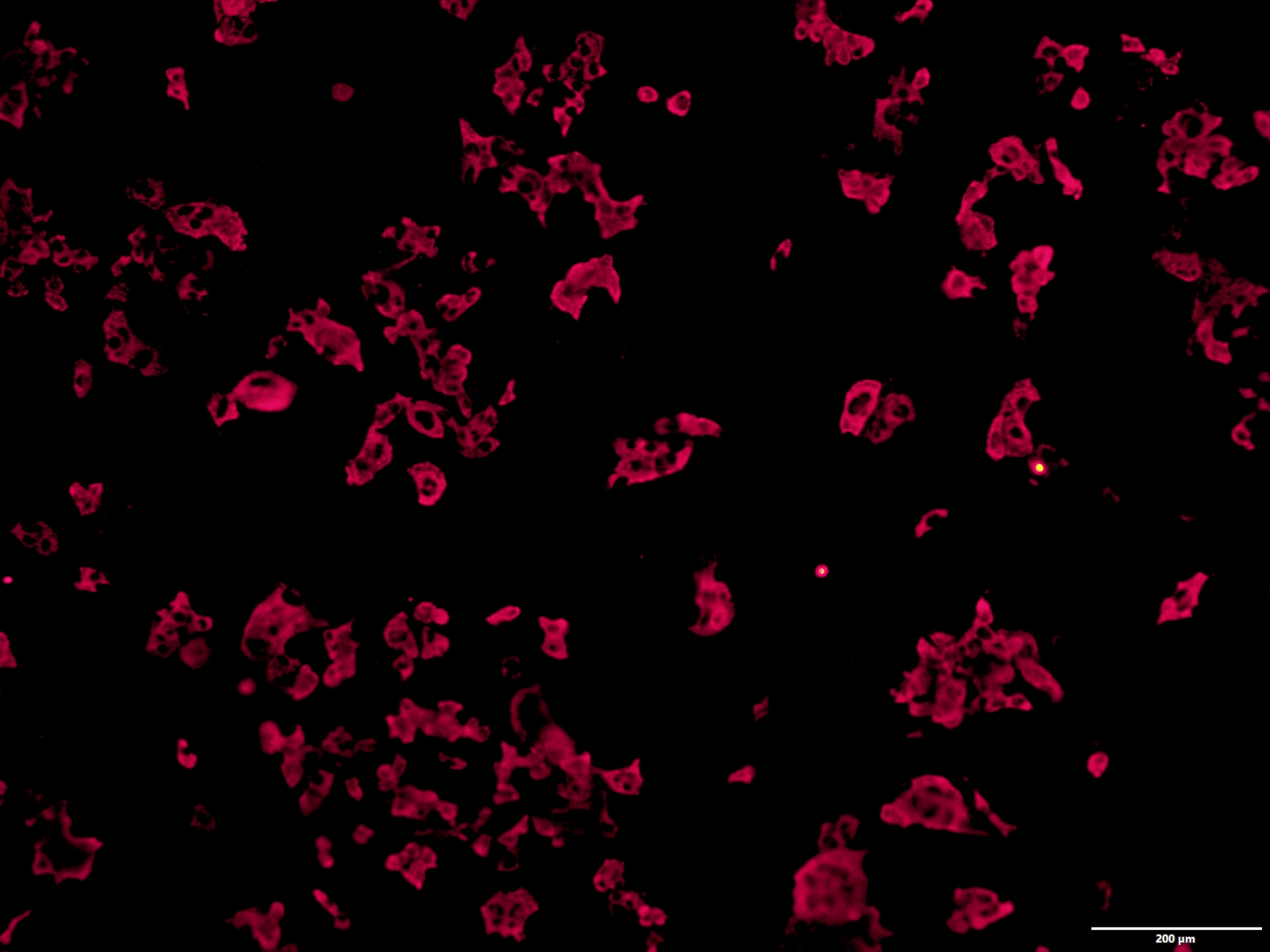

Supplement: S1 Data — This compressed folder contains the underlying numerical data and/or uncropped images used to generate the panels in Fig 1. (ZIP) [file pbio.3003736.s015.zip › S1 Data/Figure 1/I/IFA/caco2-WT/WT/caco2-229en-4.jpg]

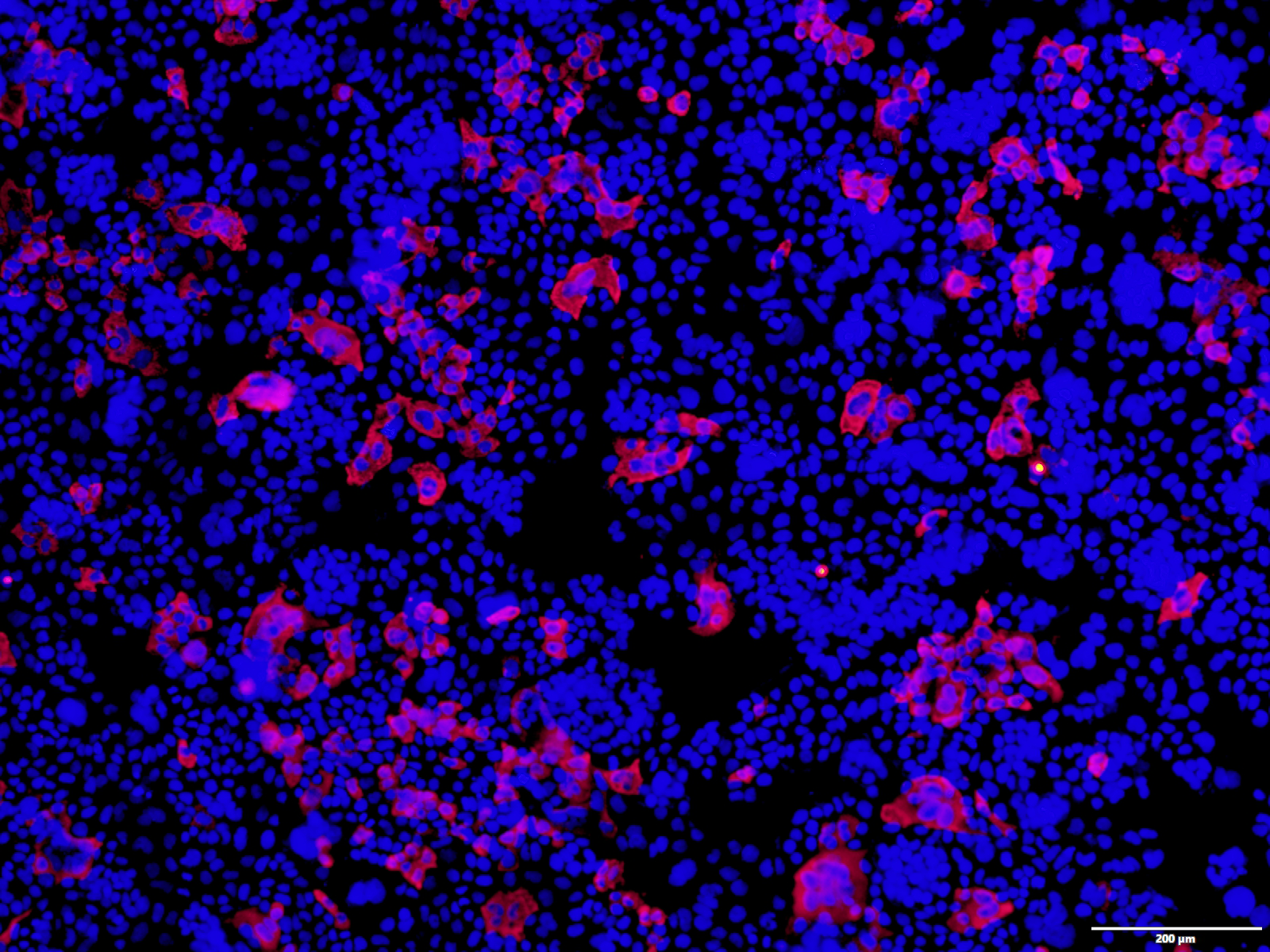

Supplement: S1 Data — This compressed folder contains the underlying numerical data and/or uncropped images used to generate the panels in Fig 1. (ZIP) [file pbio.3003736.s015.zip › S1 Data/Figure 1/I/IFA/caco2-WT/WT/caco2-dapi-4-merge.png]

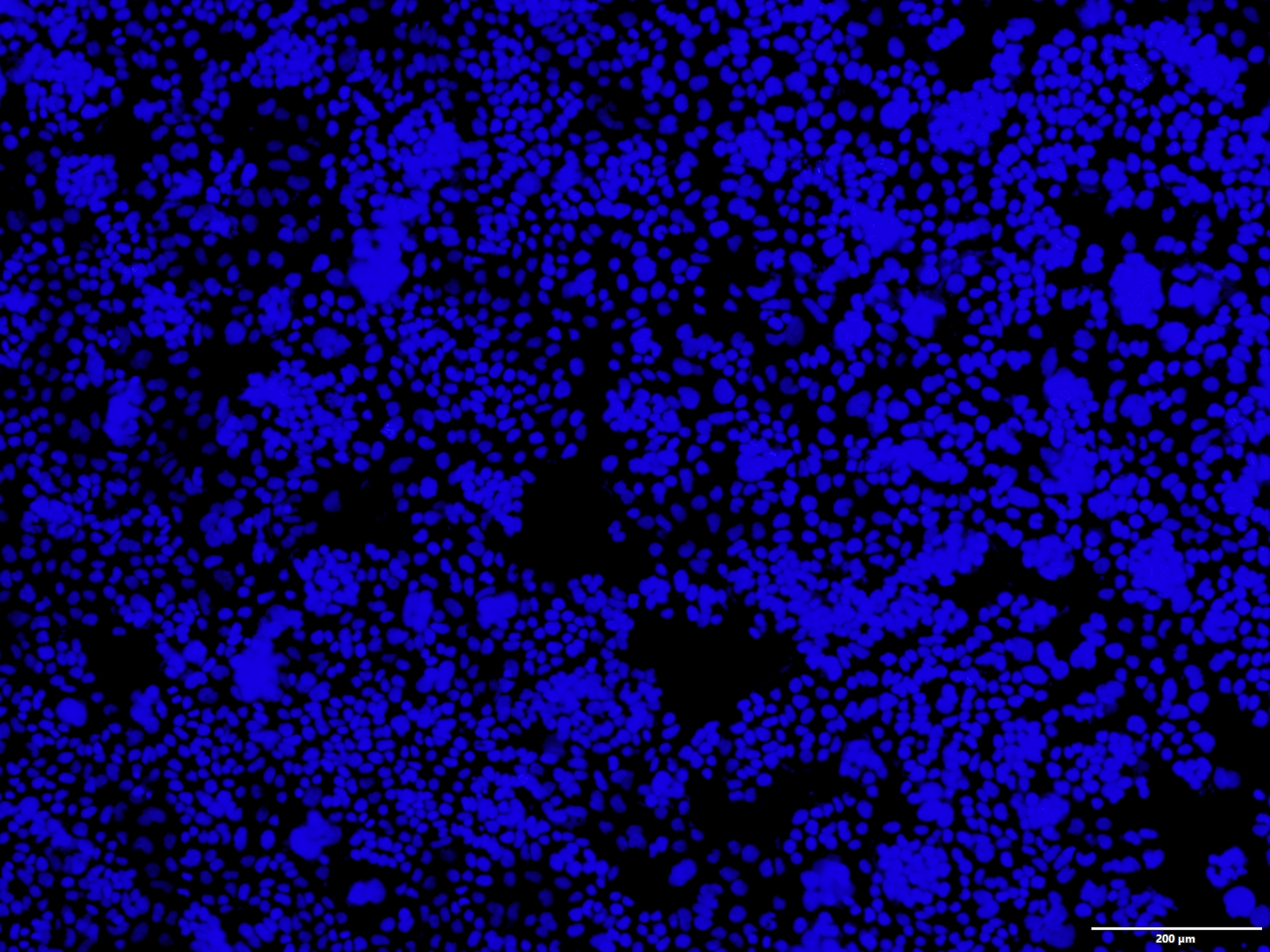

Supplement: S1 Data — This compressed folder contains the underlying numerical data and/or uncropped images used to generate the panels in Fig 1. (ZIP) [file pbio.3003736.s015.zip › S1 Data/Figure 1/I/IFA/caco2-WT/WT/caco2-dapi-4.jpg]

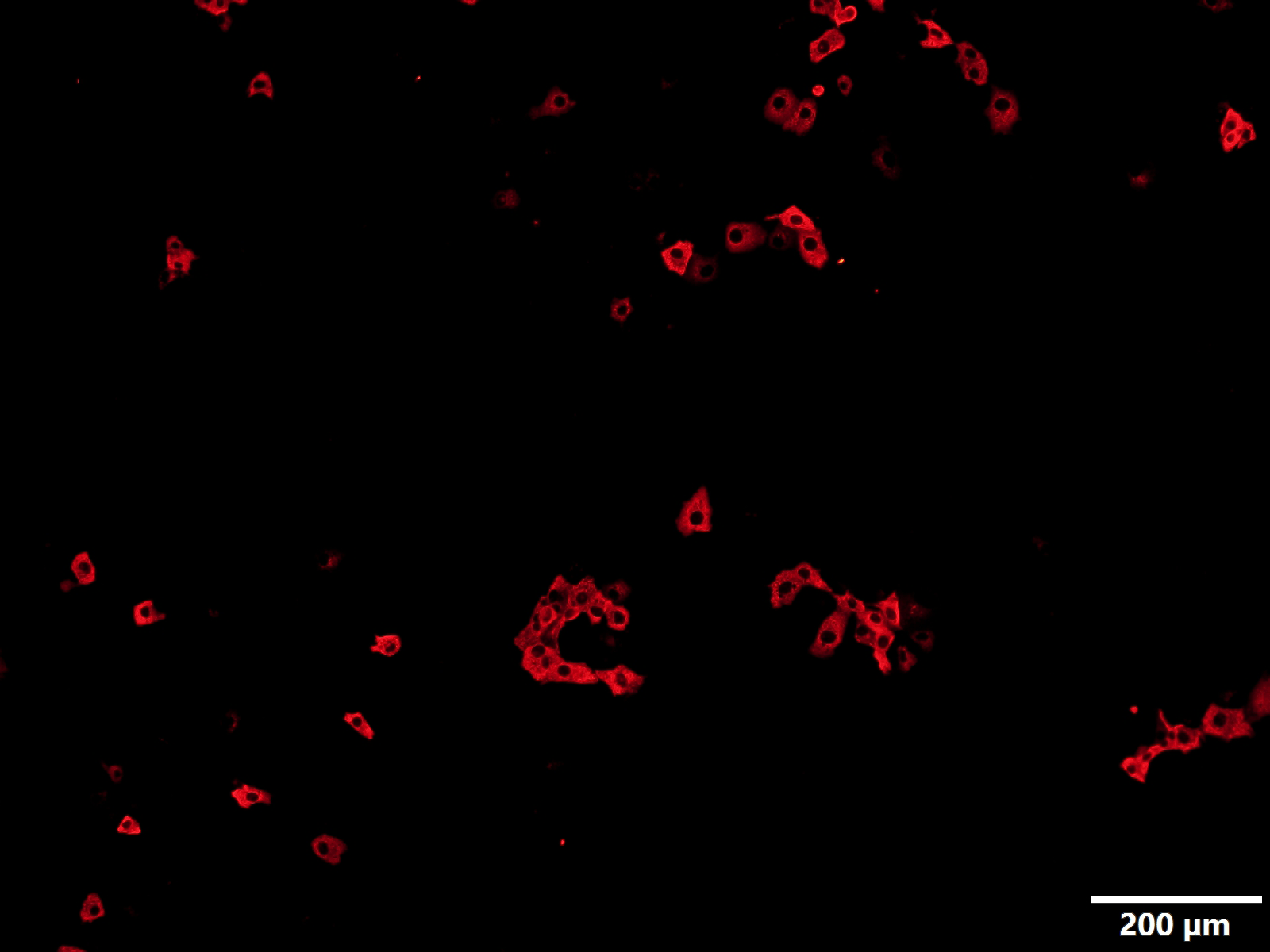

Supplement: S1 Data — This compressed folder contains the underlying numerical data and/or uncropped images used to generate the panels in Fig 1. (ZIP) [file pbio.3003736.s015.zip › S1 Data/Figure 1/J/IFA-0.01moi-24h/ko/1.png]

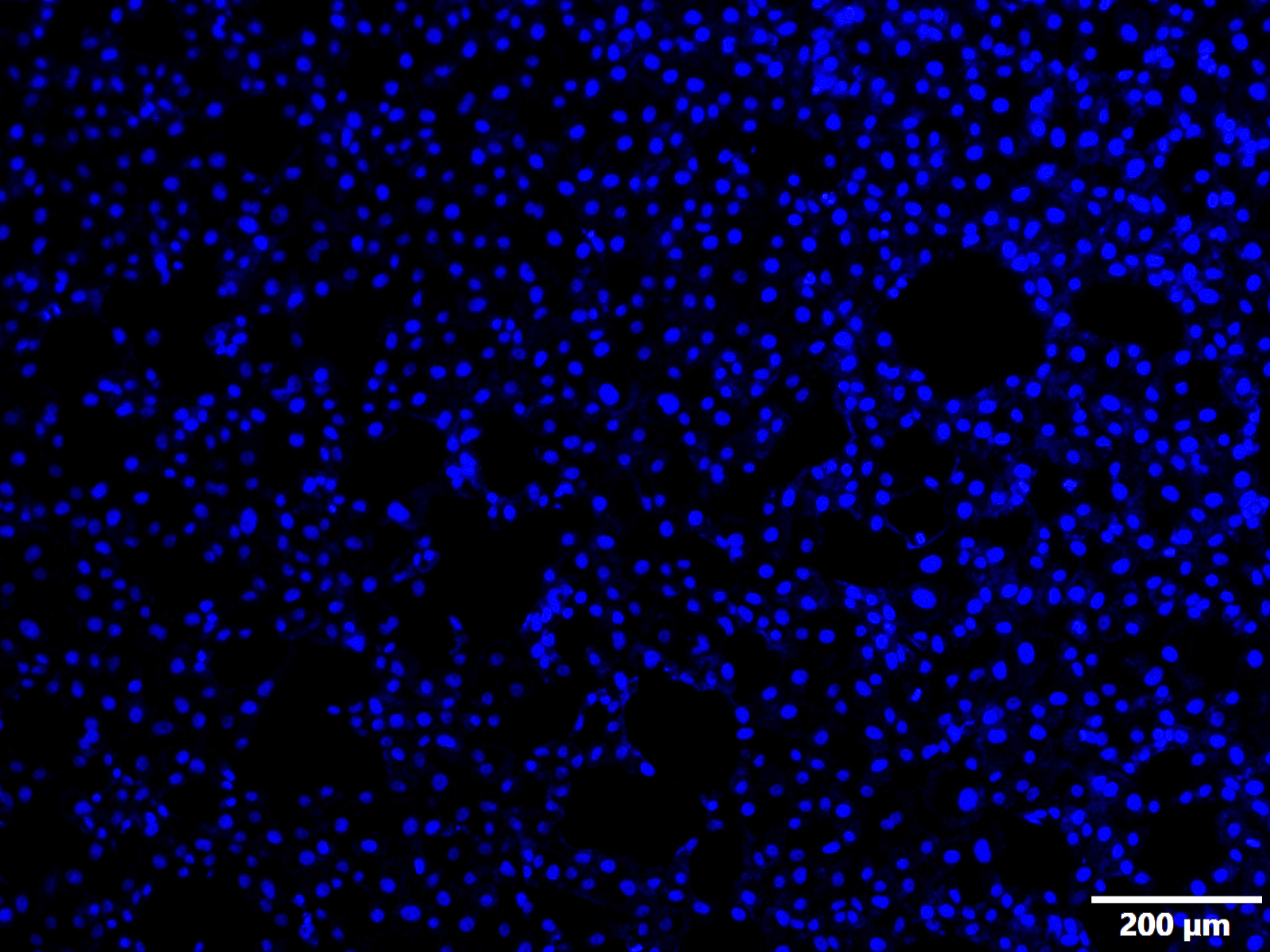

Supplement: S1 Data — This compressed folder contains the underlying numerical data and/or uncropped images used to generate the panels in Fig 1. (ZIP) [file pbio.3003736.s015.zip › S1 Data/Figure 1/J/IFA-0.01moi-24h/ko/2.png]

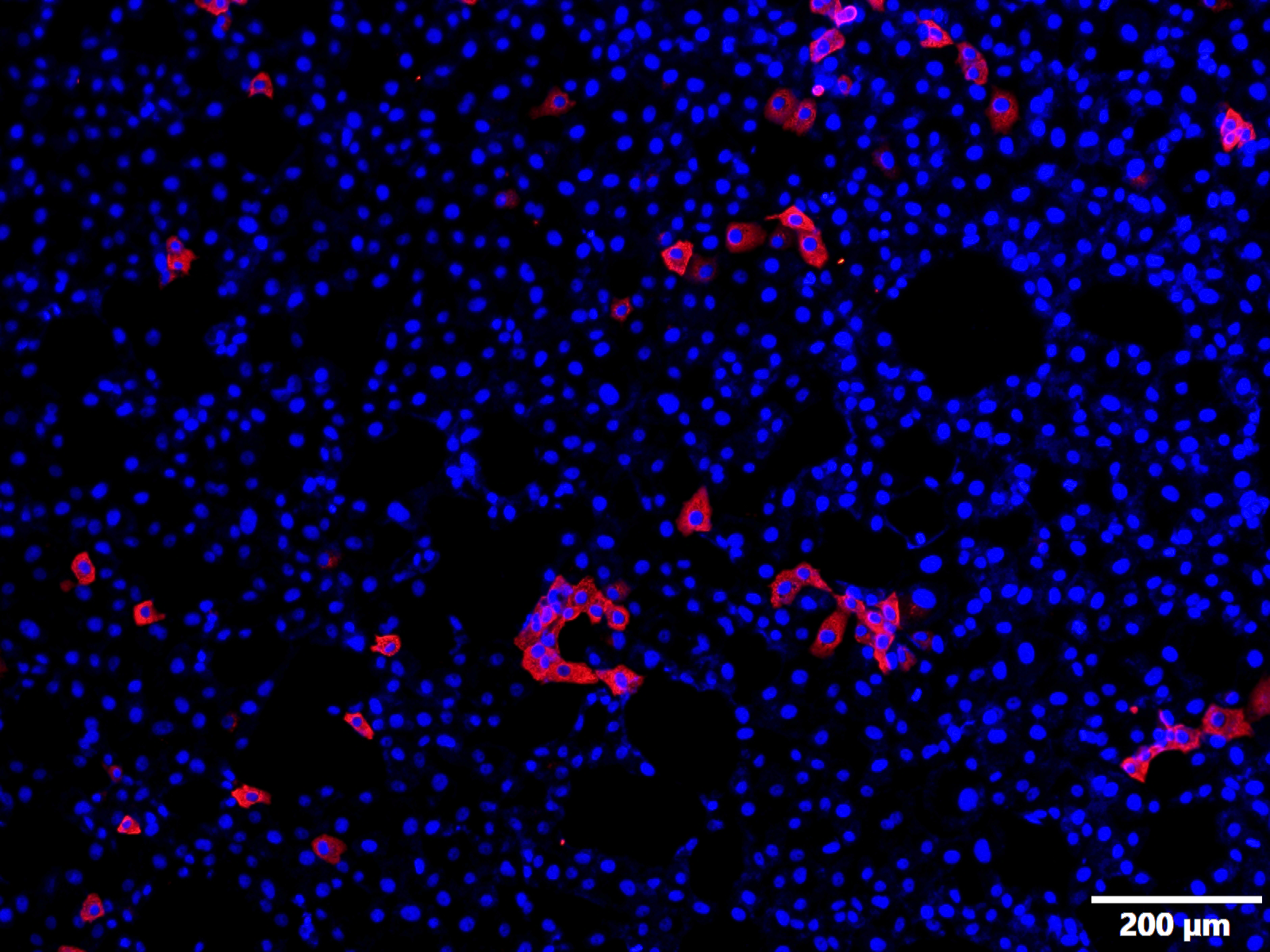

Supplement: S1 Data — This compressed folder contains the underlying numerical data and/or uncropped images used to generate the panels in Fig 1. (ZIP) [file pbio.3003736.s015.zip › S1 Data/Figure 1/J/IFA-0.01moi-24h/ko/3.png]

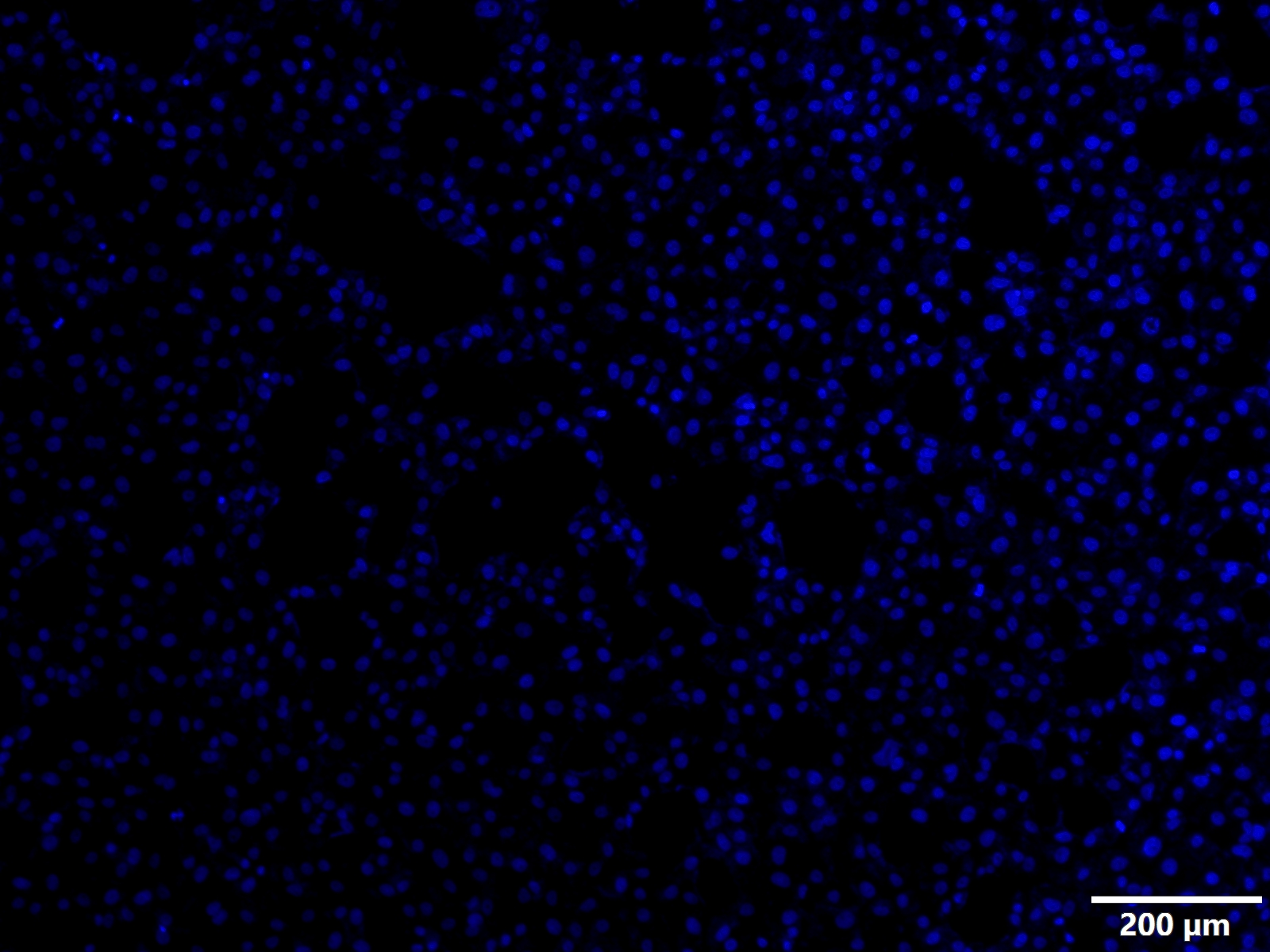

Supplement: S1 Data — This compressed folder contains the underlying numerical data and/or uncropped images used to generate the panels in Fig 1. (ZIP) [file pbio.3003736.s015.zip › S1 Data/Figure 1/J/IFA-0.01moi-24h/ko/4.jpg]

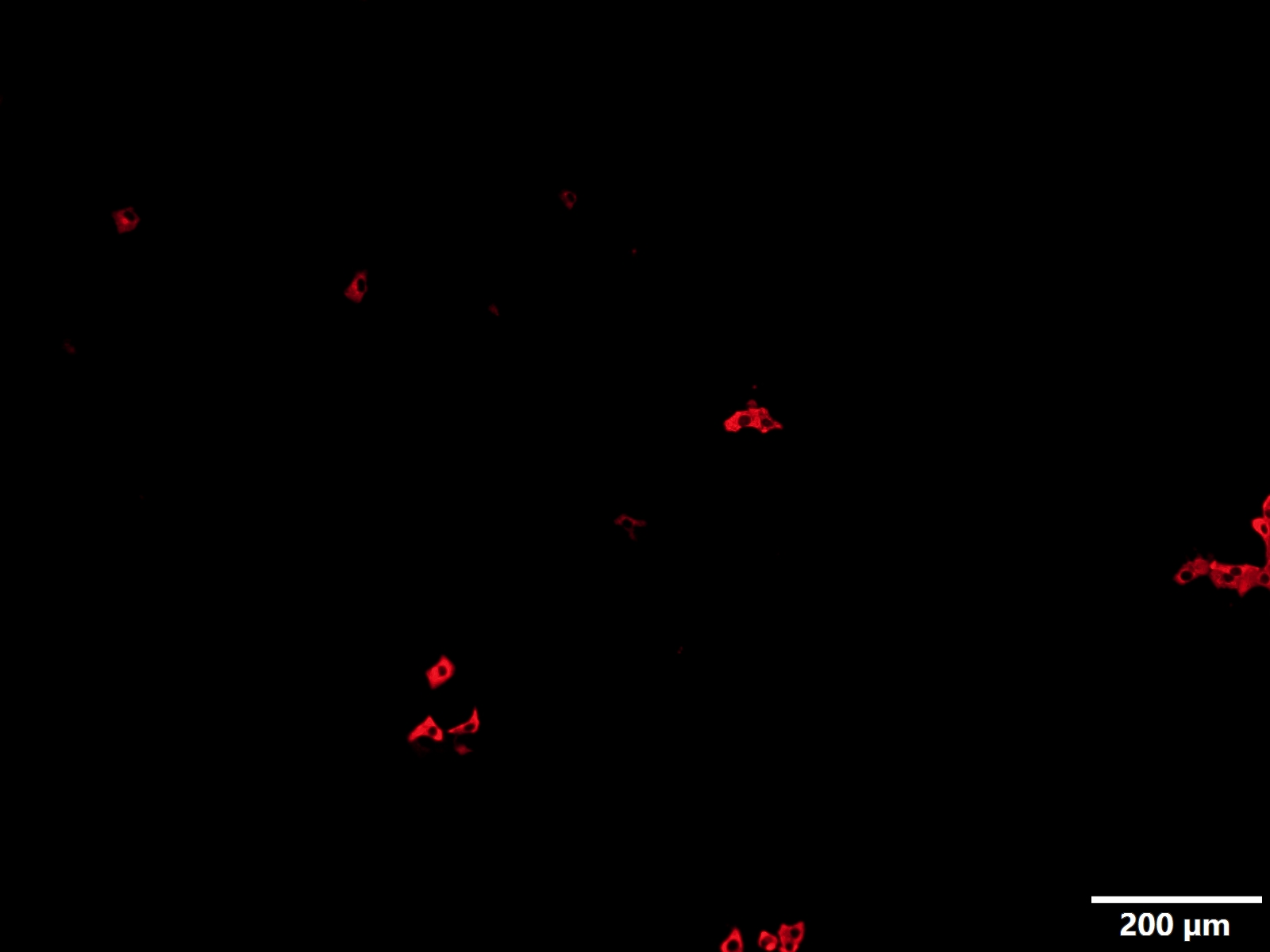

Supplement: S1 Data — This compressed folder contains the underlying numerical data and/or uncropped images used to generate the panels in Fig 1. (ZIP) [file pbio.3003736.s015.zip › S1 Data/Figure 1/J/IFA-0.01moi-24h/ko/5.jpg]

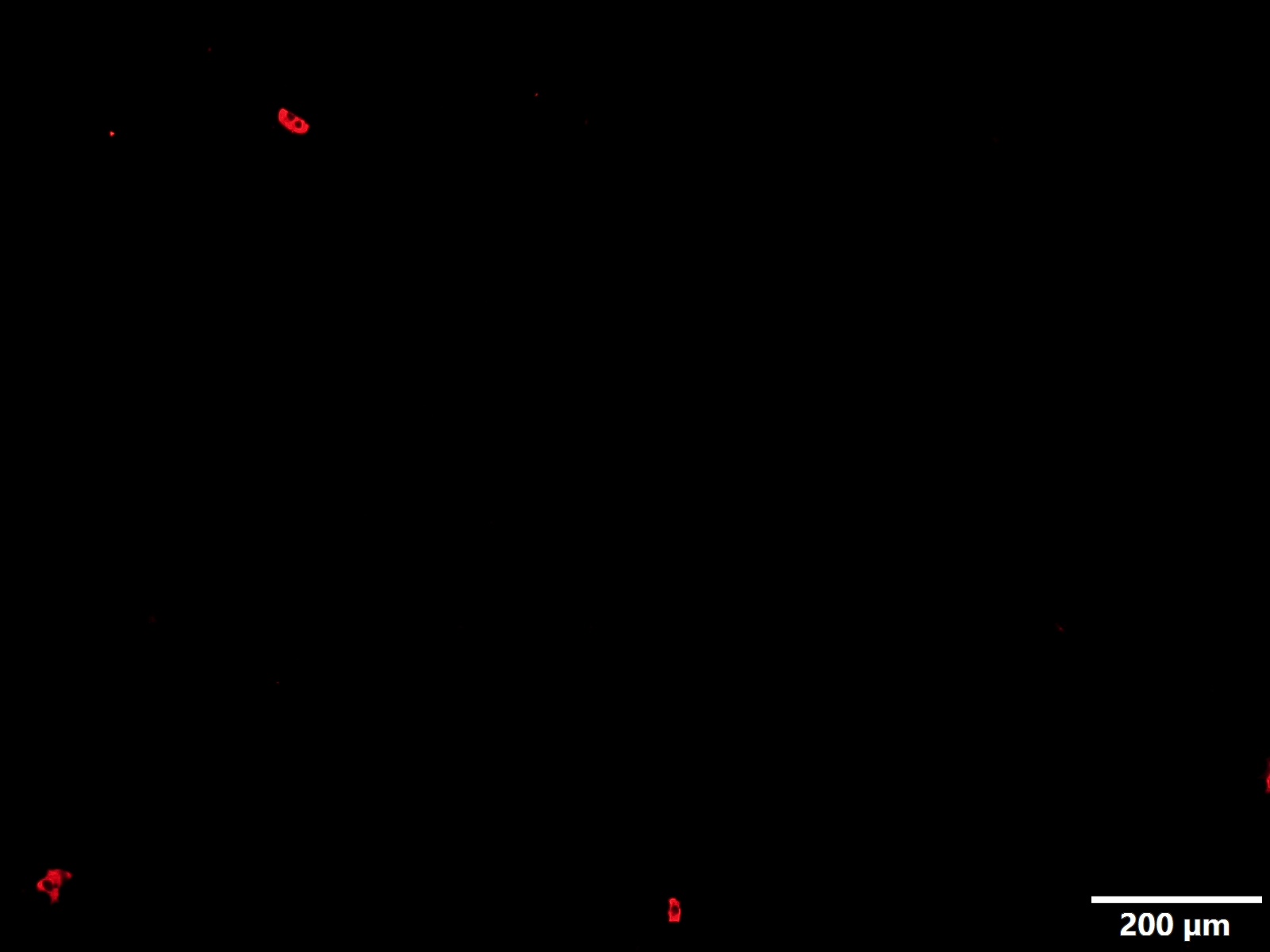

Supplement: S1 Data — This compressed folder contains the underlying numerical data and/or uncropped images used to generate the panels in Fig 1. (ZIP) [file pbio.3003736.s015.zip › S1 Data/Figure 1/J/IFA-0.01moi-24h/ko/6.jpg]

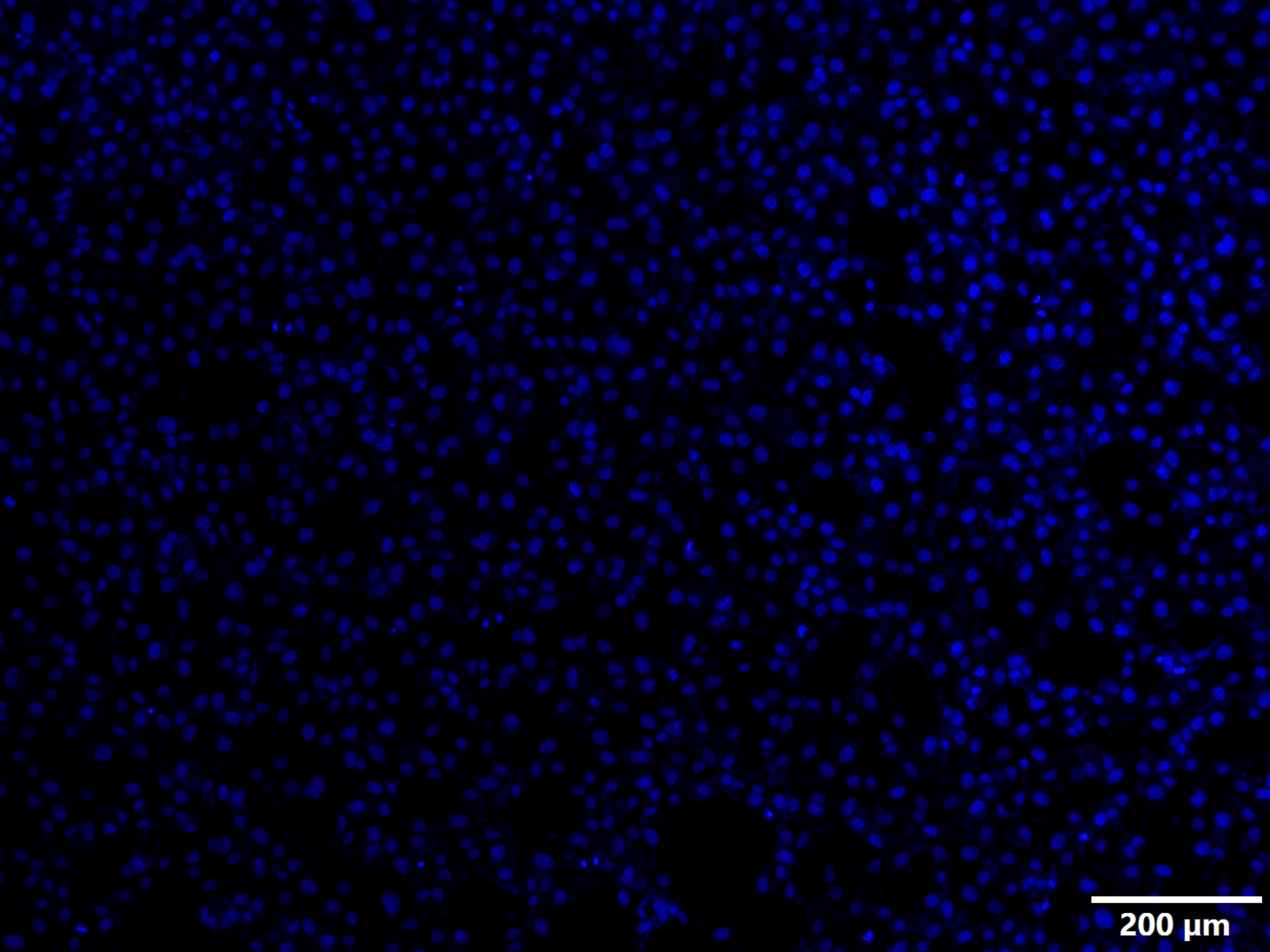

Supplement: S1 Data — This compressed folder contains the underlying numerical data and/or uncropped images used to generate the panels in Fig 1. (ZIP) [file pbio.3003736.s015.zip › S1 Data/Figure 1/J/IFA-0.01moi-24h/ko/7.jpg]

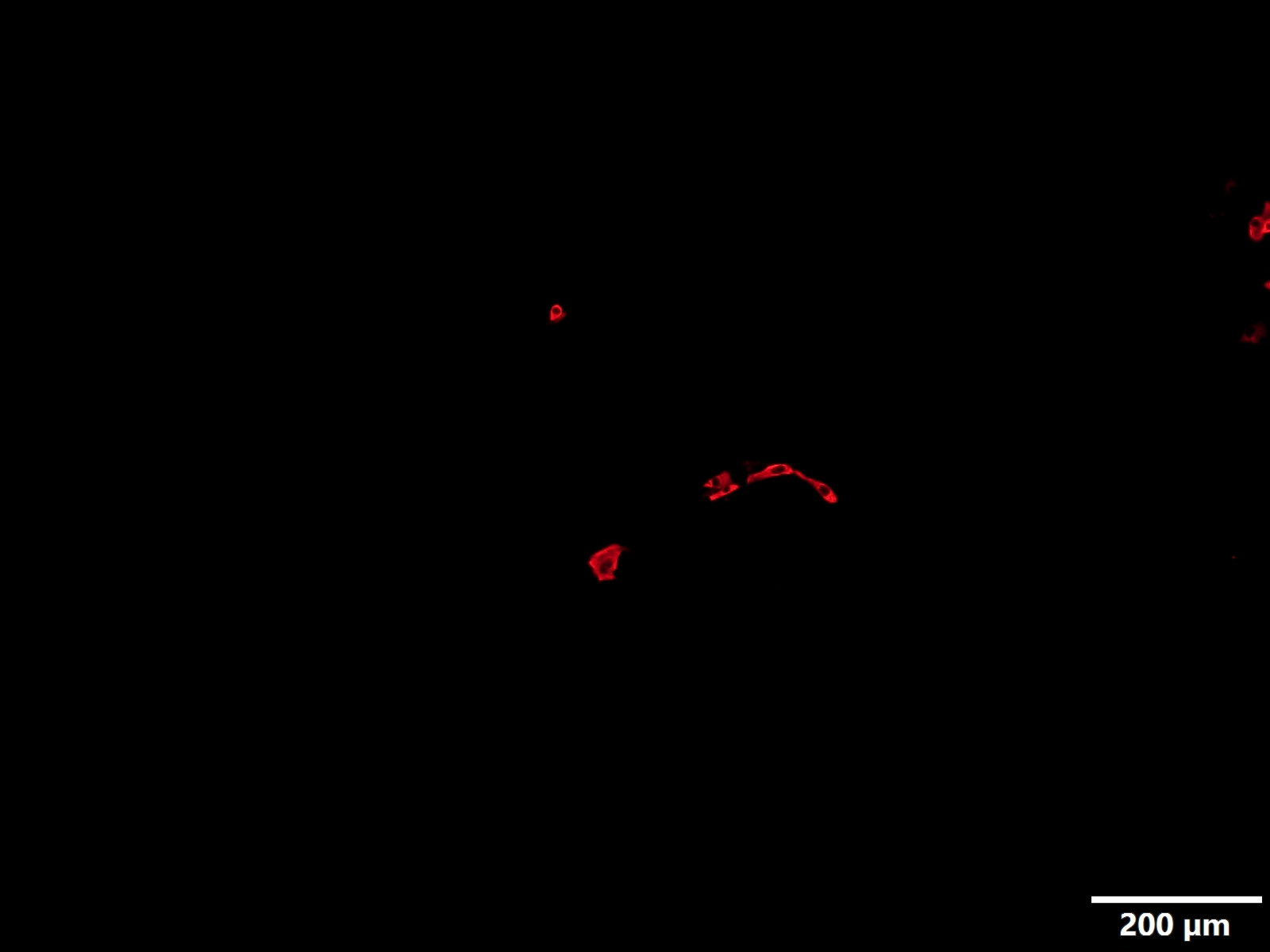

Supplement: S1 Data — This compressed folder contains the underlying numerical data and/or uncropped images used to generate the panels in Fig 1. (ZIP) [file pbio.3003736.s015.zip › S1 Data/Figure 1/J/IFA-0.01moi-24h/ko/8.jpg]

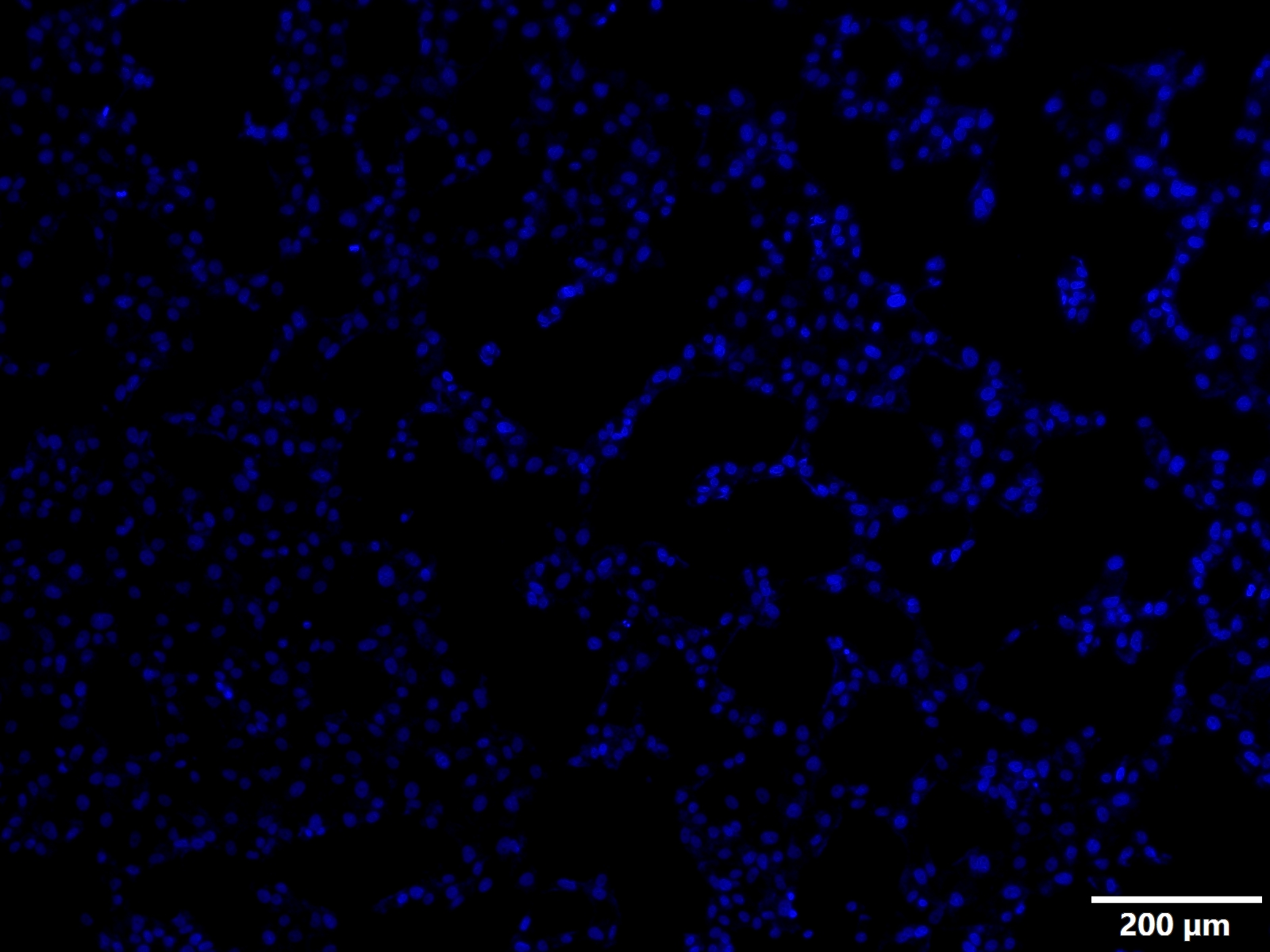

Supplement: S1 Data — This compressed folder contains the underlying numerical data and/or uncropped images used to generate the panels in Fig 1. (ZIP) [file pbio.3003736.s015.zip › S1 Data/Figure 1/J/IFA-0.01moi-24h/ko/9.jpg]

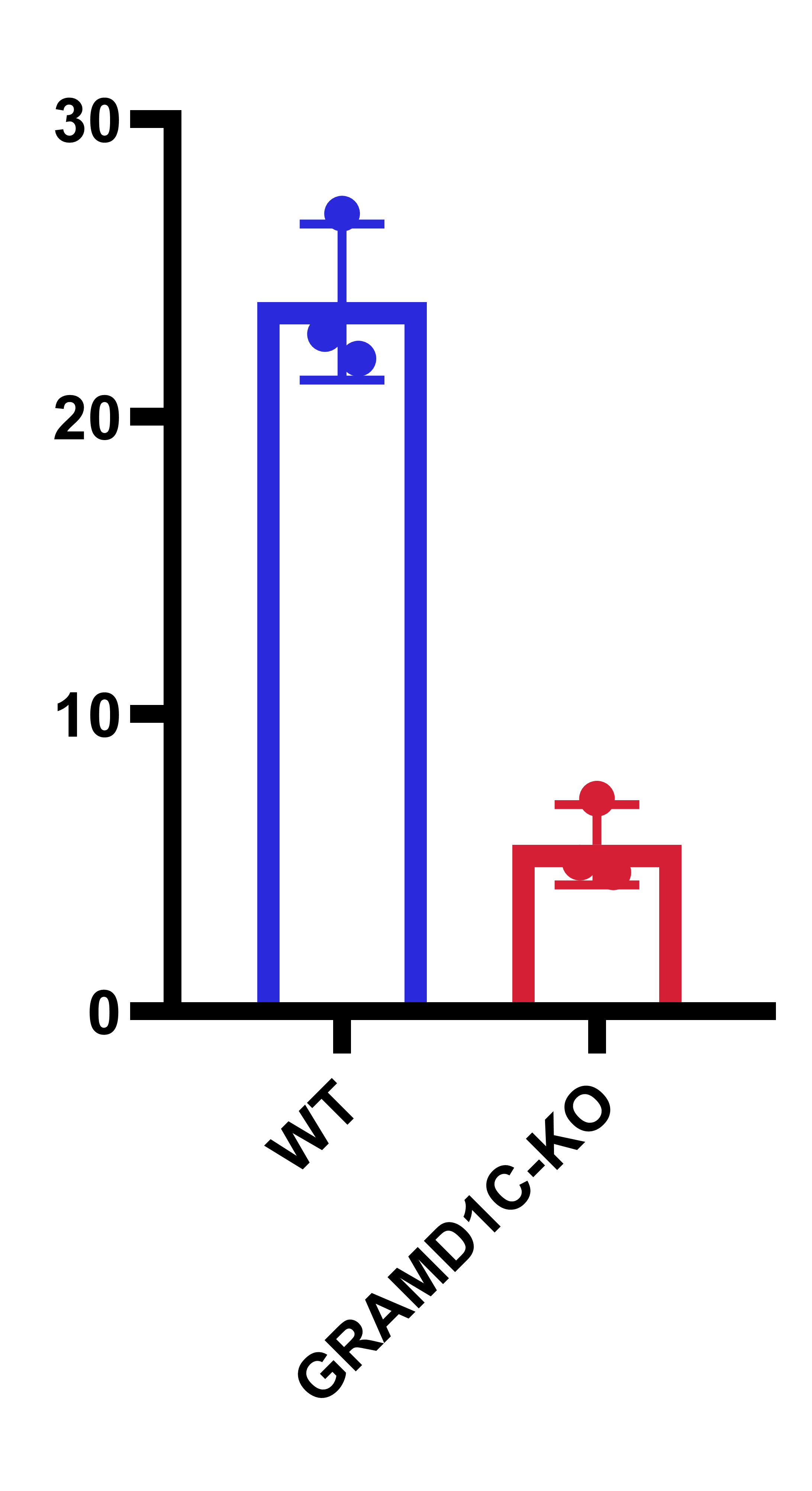

Supplement: S1 Data — This compressed folder contains the underlying numerical data and/or uncropped images used to generate the panels in Fig 1. (ZIP) [file pbio.3003736.s015.zip › S1 Data/Figure 1/J/IFA-0.01moi-24h/sars-2-positive-cells.tif]

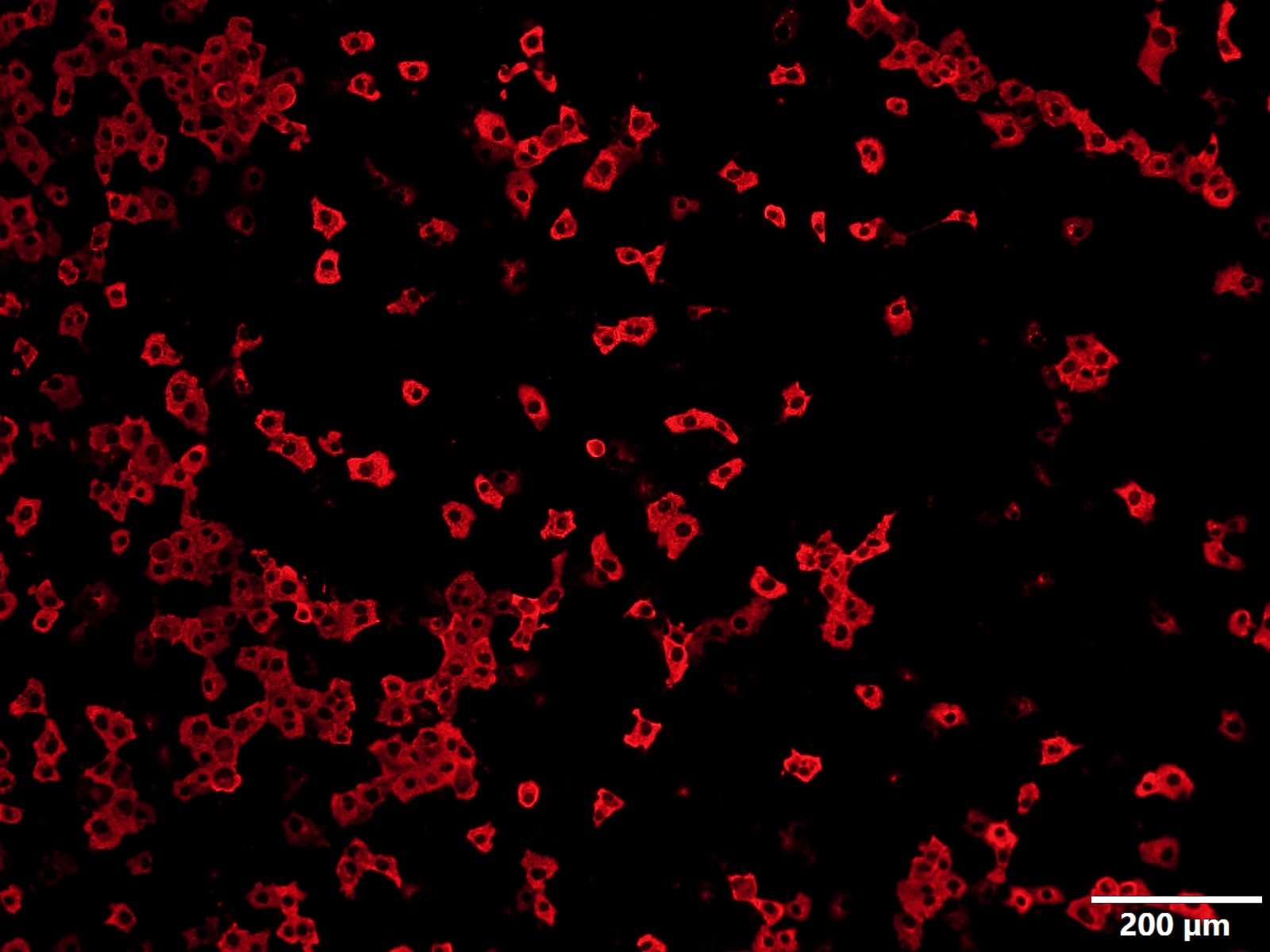

Supplement: S1 Data — This compressed folder contains the underlying numerical data and/or uncropped images used to generate the panels in Fig 1. (ZIP) [file pbio.3003736.s015.zip › S1 Data/Figure 1/J/IFA-0.01moi-24h/VERO/1.png]

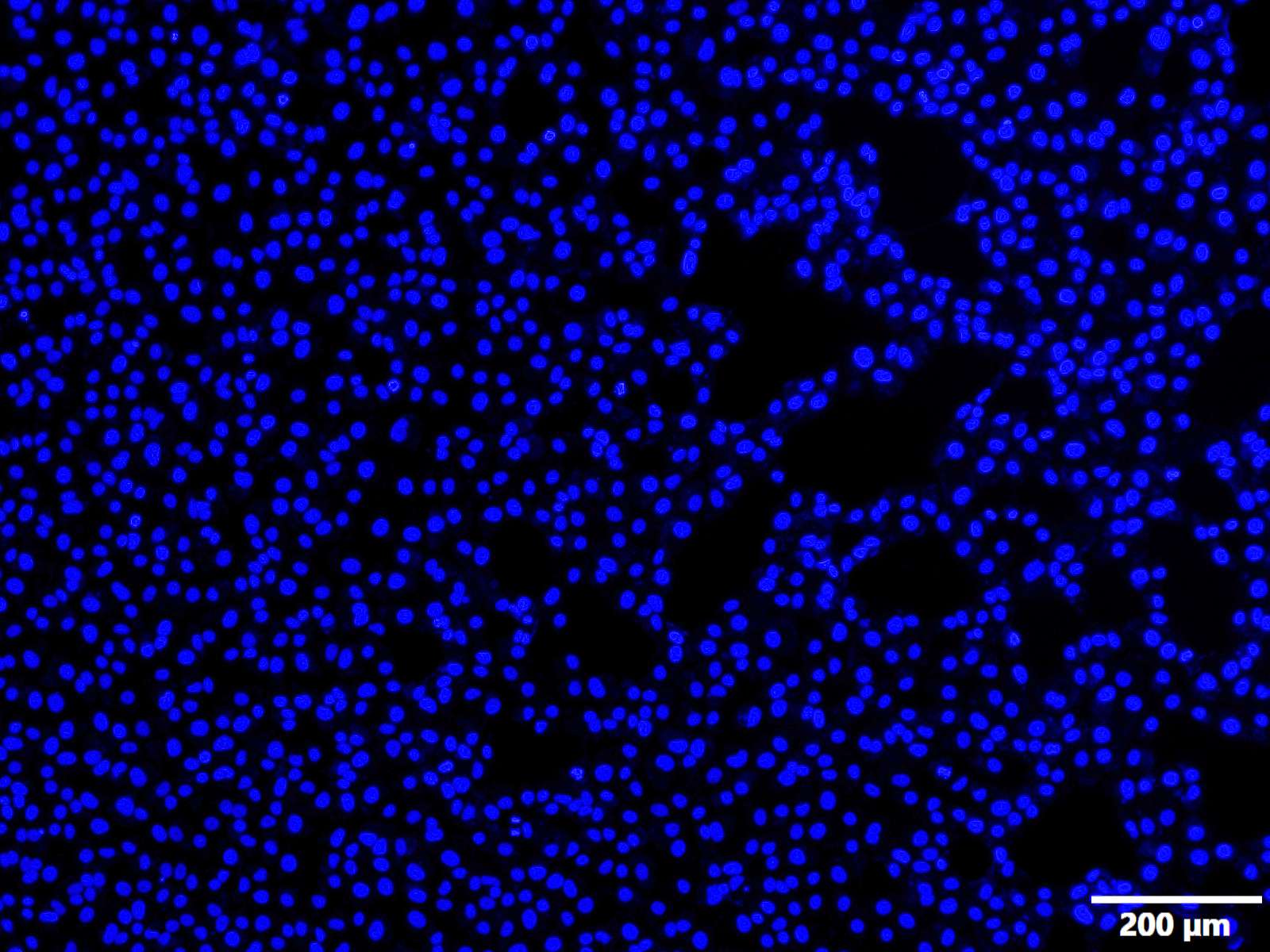

Supplement: S1 Data — This compressed folder contains the underlying numerical data and/or uncropped images used to generate the panels in Fig 1. (ZIP) [file pbio.3003736.s015.zip › S1 Data/Figure 1/J/IFA-0.01moi-24h/VERO/2.png]

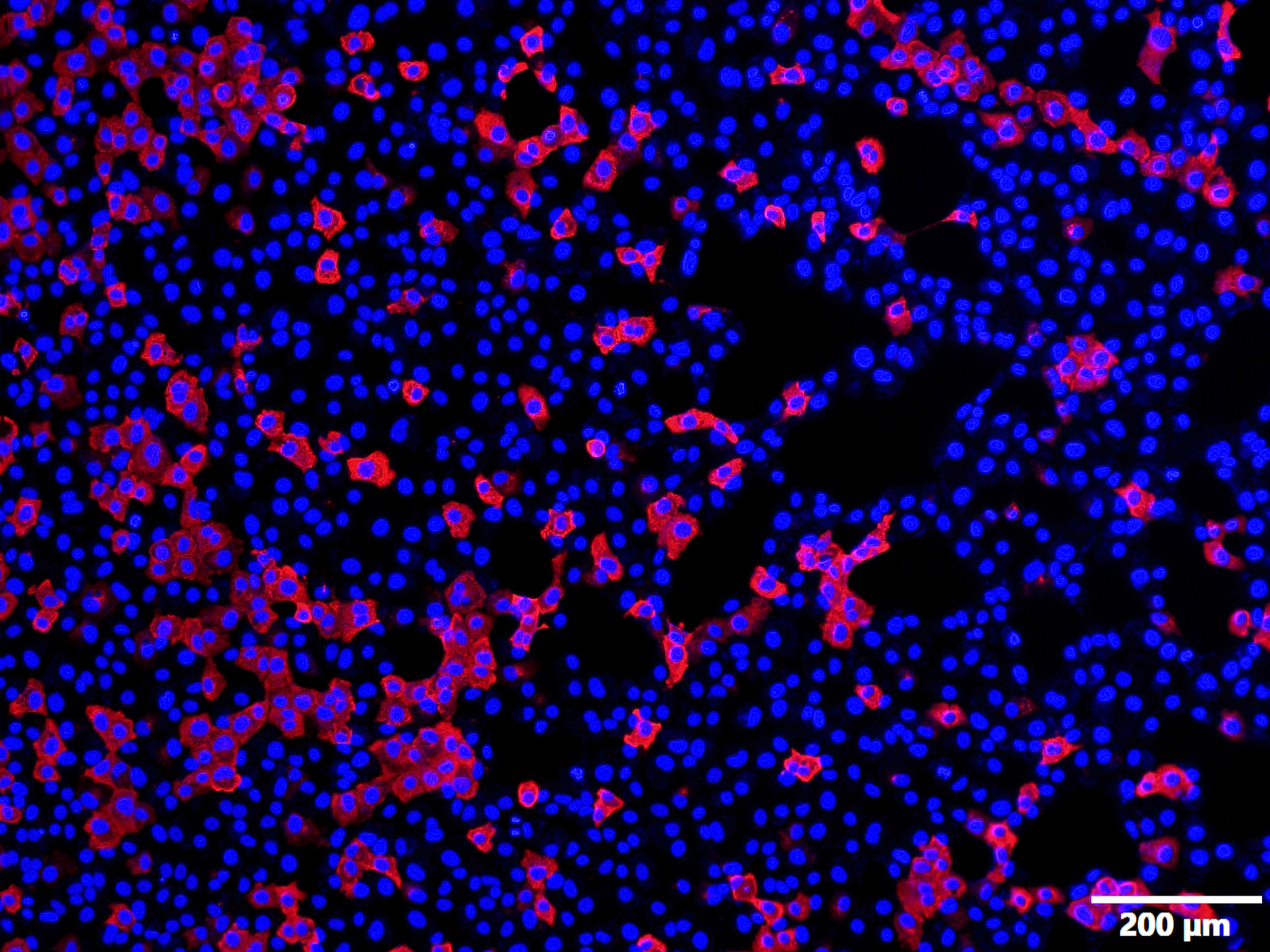

Supplement: S1 Data — This compressed folder contains the underlying numerical data and/or uncropped images used to generate the panels in Fig 1. (ZIP) [file pbio.3003736.s015.zip › S1 Data/Figure 1/J/IFA-0.01moi-24h/VERO/3.png]

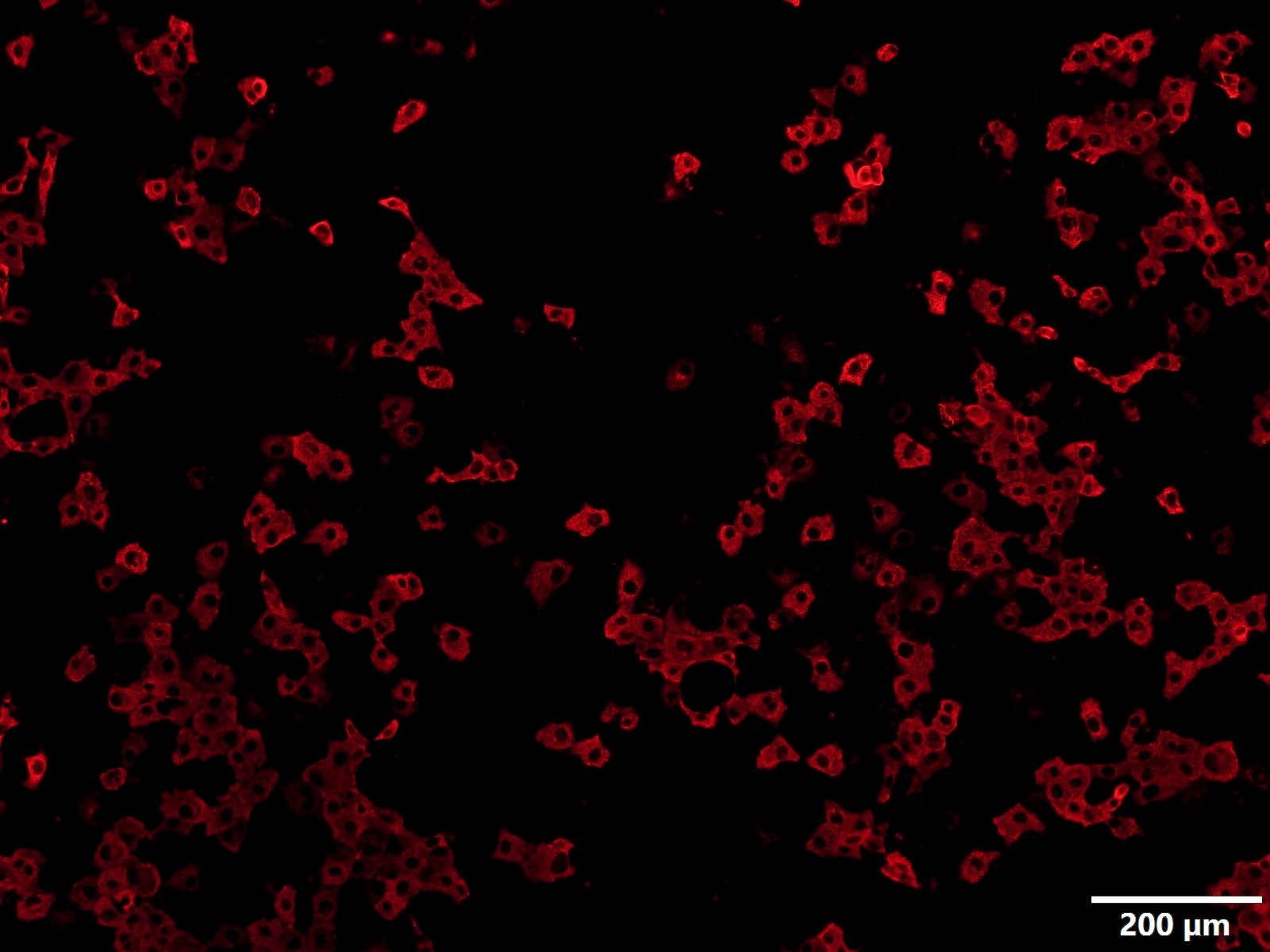

Supplement: S1 Data — This compressed folder contains the underlying numerical data and/or uncropped images used to generate the panels in Fig 1. (ZIP) [file pbio.3003736.s015.zip › S1 Data/Figure 1/J/IFA-0.01moi-24h/VERO/4.jpg]

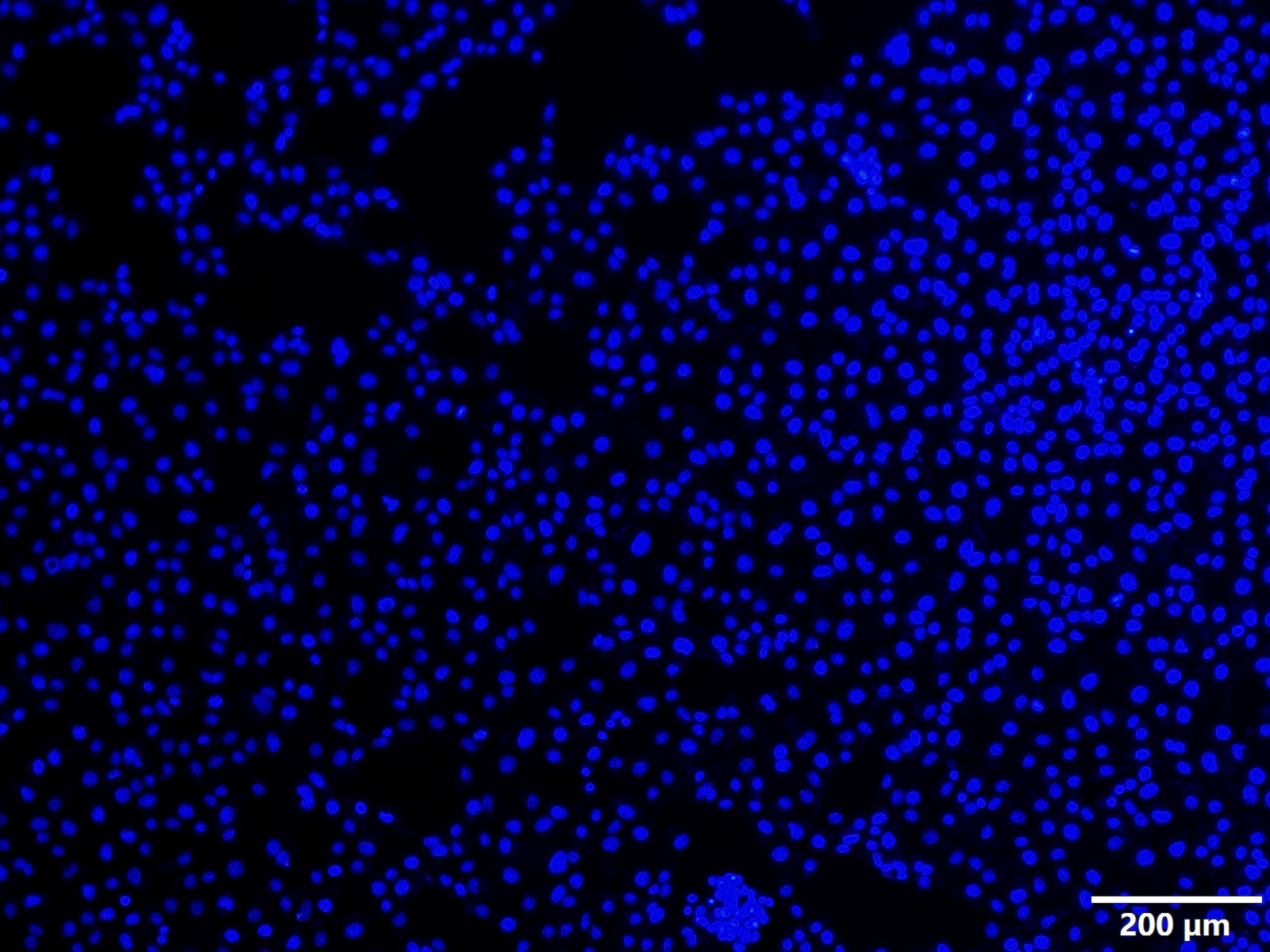

Supplement: S1 Data — This compressed folder contains the underlying numerical data and/or uncropped images used to generate the panels in Fig 1. (ZIP) [file pbio.3003736.s015.zip › S1 Data/Figure 1/J/IFA-0.01moi-24h/VERO/5.jpg]

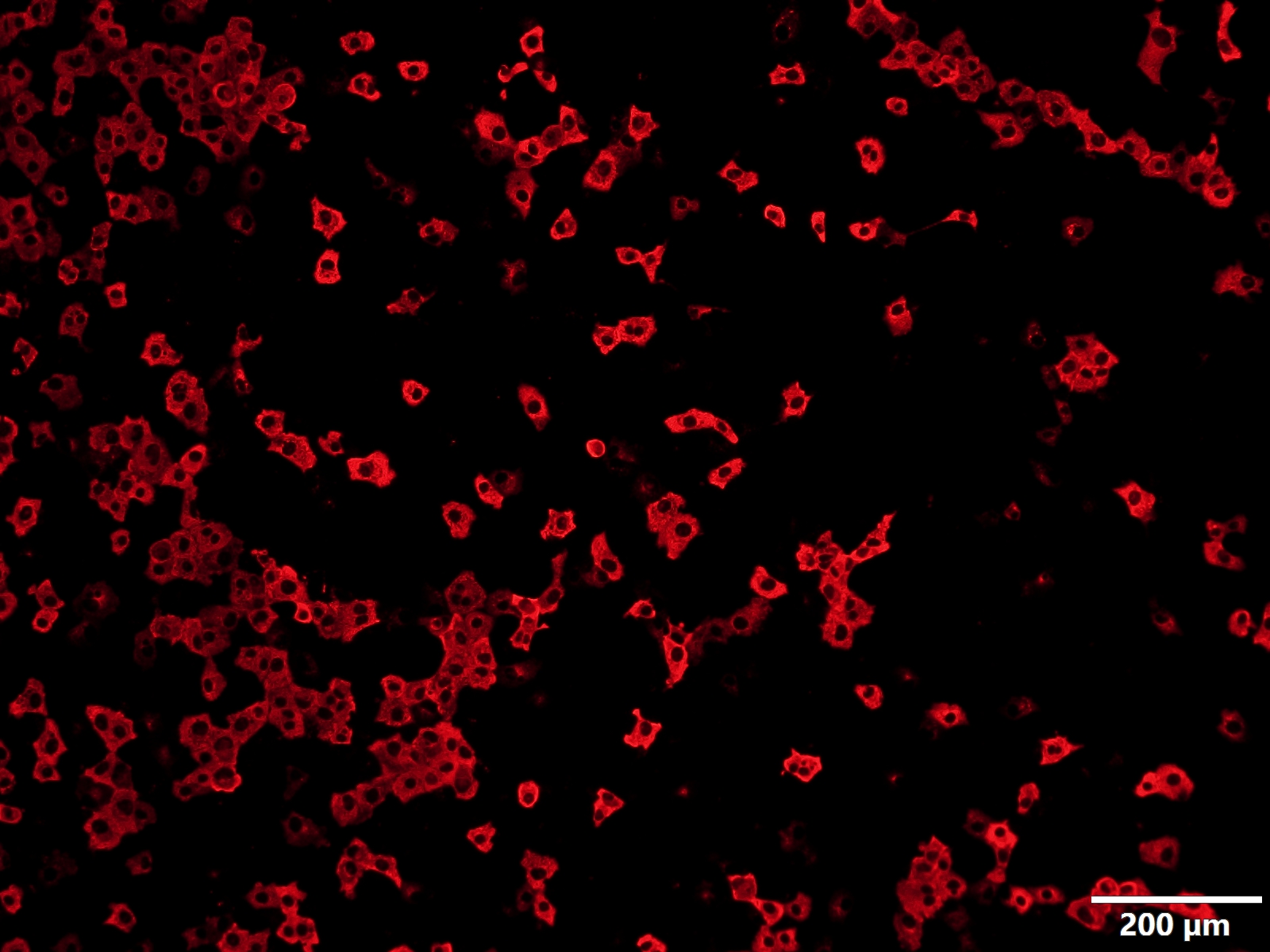

Supplement: S1 Data — This compressed folder contains the underlying numerical data and/or uncropped images used to generate the panels in Fig 1. (ZIP) [file pbio.3003736.s015.zip › S1 Data/Figure 1/J/IFA-0.01moi-24h/VERO/6.jpg]

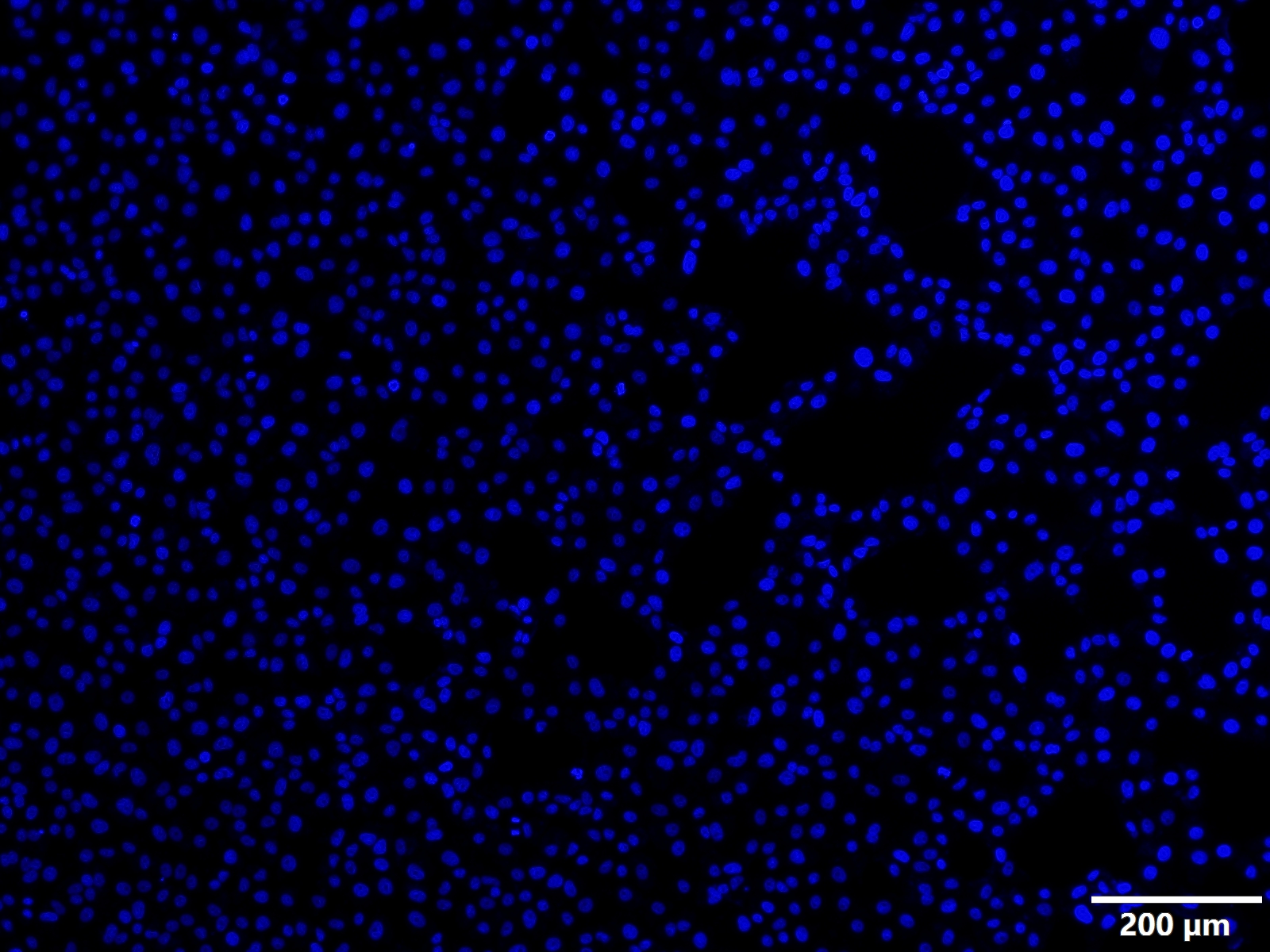

Supplement: S1 Data — This compressed folder contains the underlying numerical data and/or uncropped images used to generate the panels in Fig 1. (ZIP) [file pbio.3003736.s015.zip › S1 Data/Figure 1/J/IFA-0.01moi-24h/VERO/7.jpg]

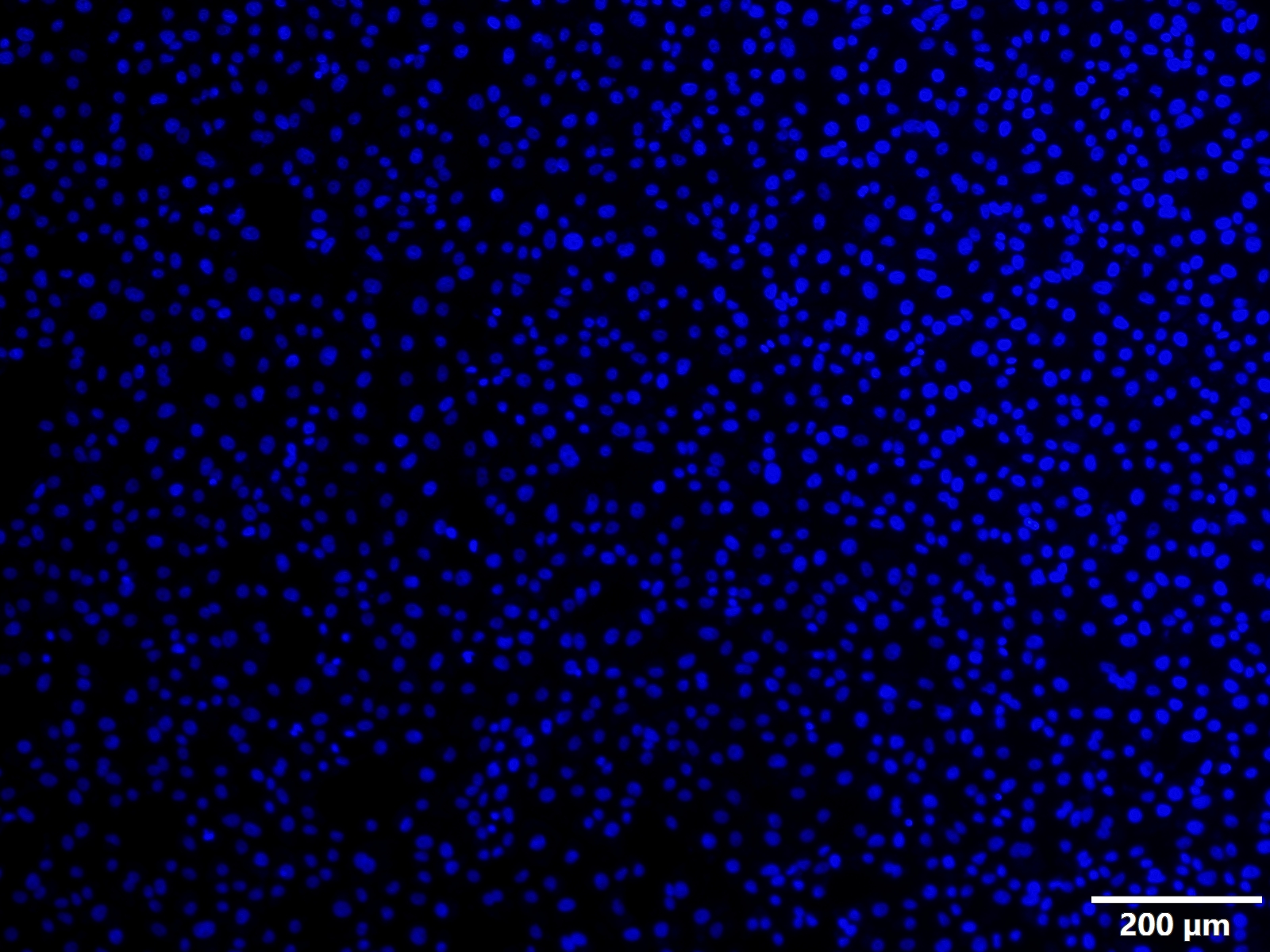

Supplement: S1 Data — This compressed folder contains the underlying numerical data and/or uncropped images used to generate the panels in Fig 1. (ZIP) [file pbio.3003736.s015.zip › S1 Data/Figure 1/J/IFA-0.01moi-24h/VERO/8.jpg]

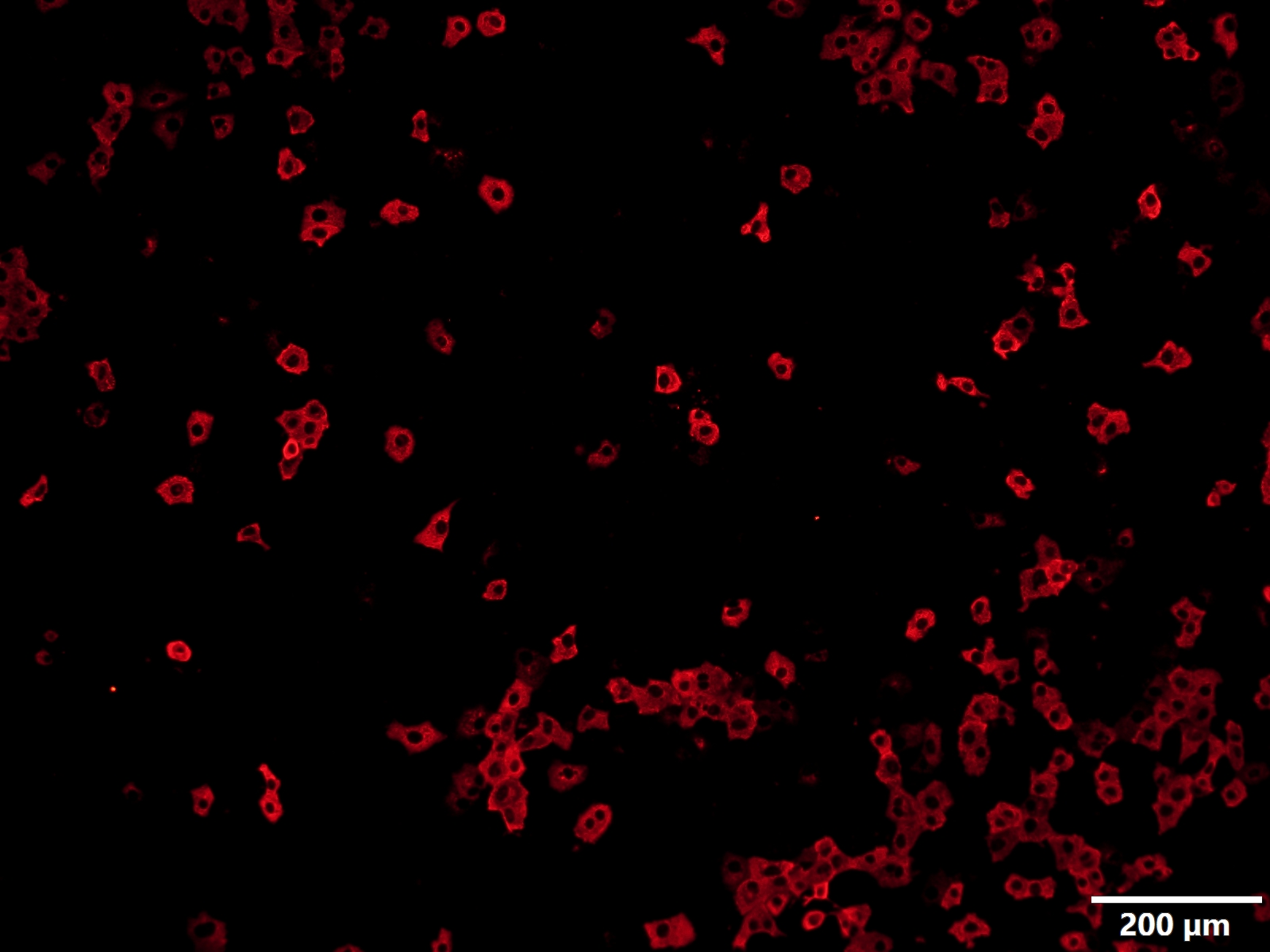

Supplement: S1 Data — This compressed folder contains the underlying numerical data and/or uncropped images used to generate the panels in Fig 1. (ZIP) [file pbio.3003736.s015.zip › S1 Data/Figure 1/J/IFA-0.01moi-24h/VERO/9.jpg]

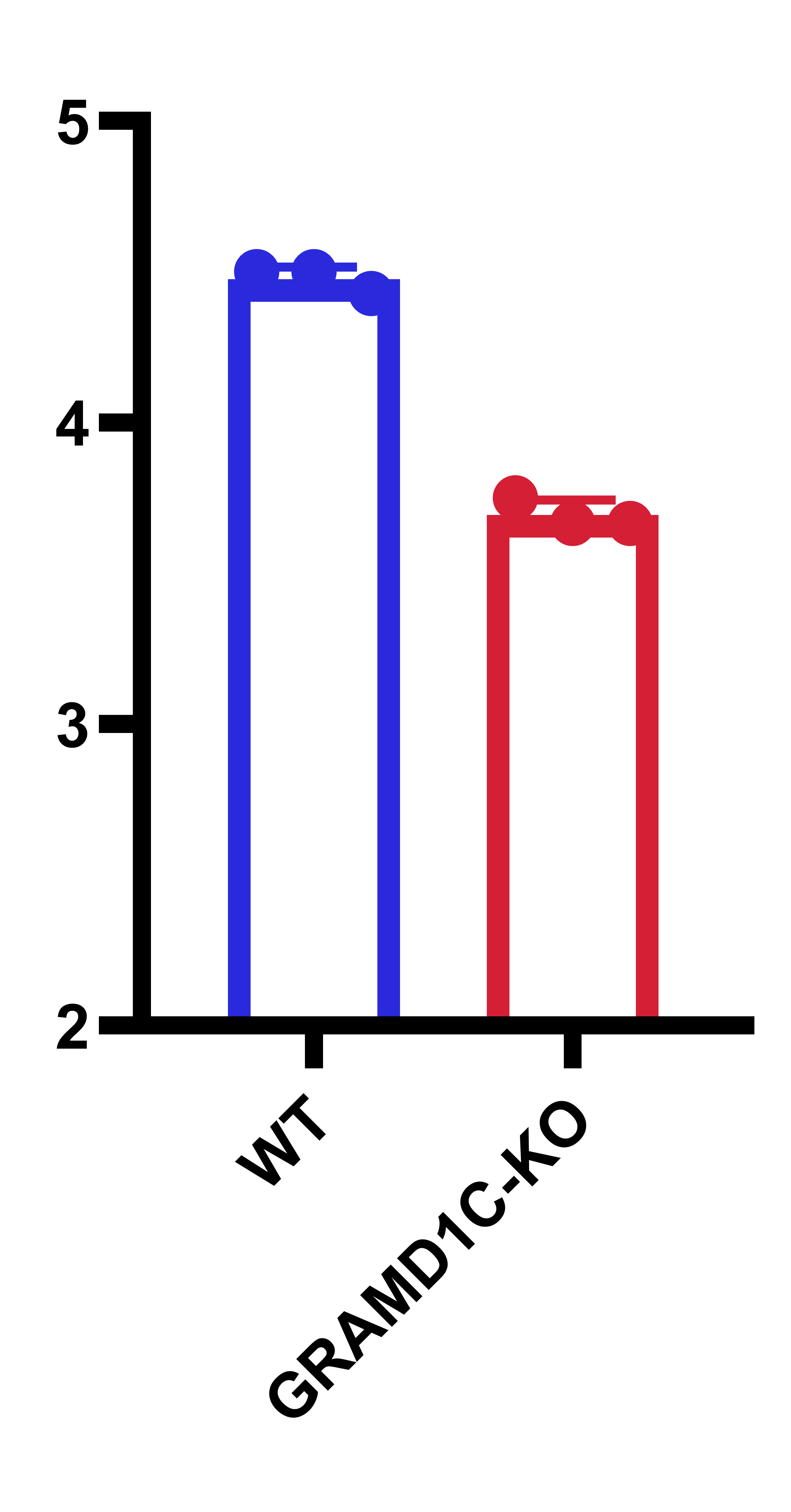

Supplement: S1 Data — This compressed folder contains the underlying numerical data and/or uncropped images used to generate the panels in Fig 1. (ZIP) [file pbio.3003736.s015.zip › S1 Data/Figure 1/J/viral-titer/titer.tif]

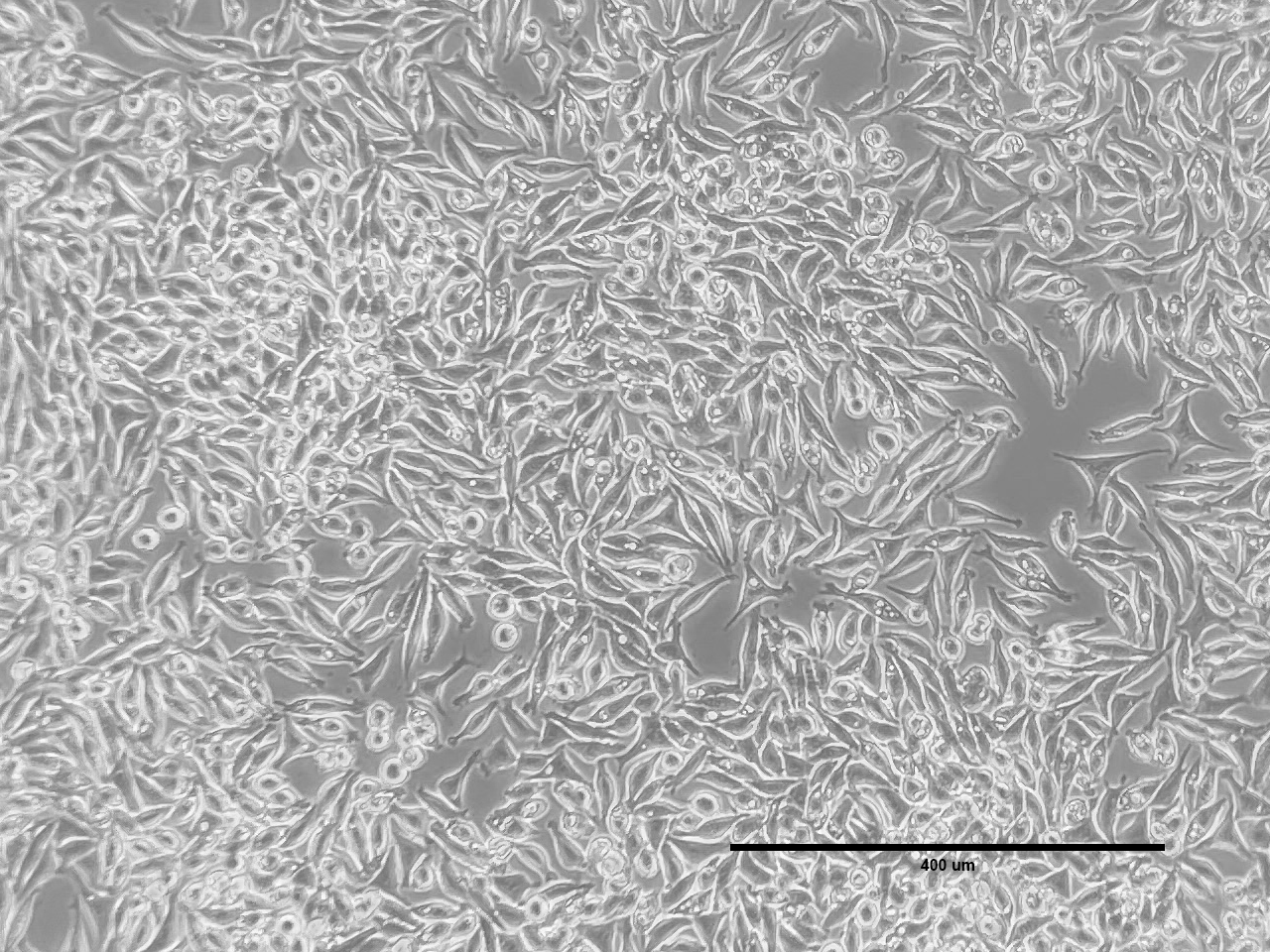

Supplement: S1 Data — This compressed folder contains the underlying numerical data and/or uncropped images used to generate the panels in Fig 1. (ZIP) [file pbio.3003736.s015.zip › S1 Data/Figure 1/K/l929-ko/ko-0.01moi.png]

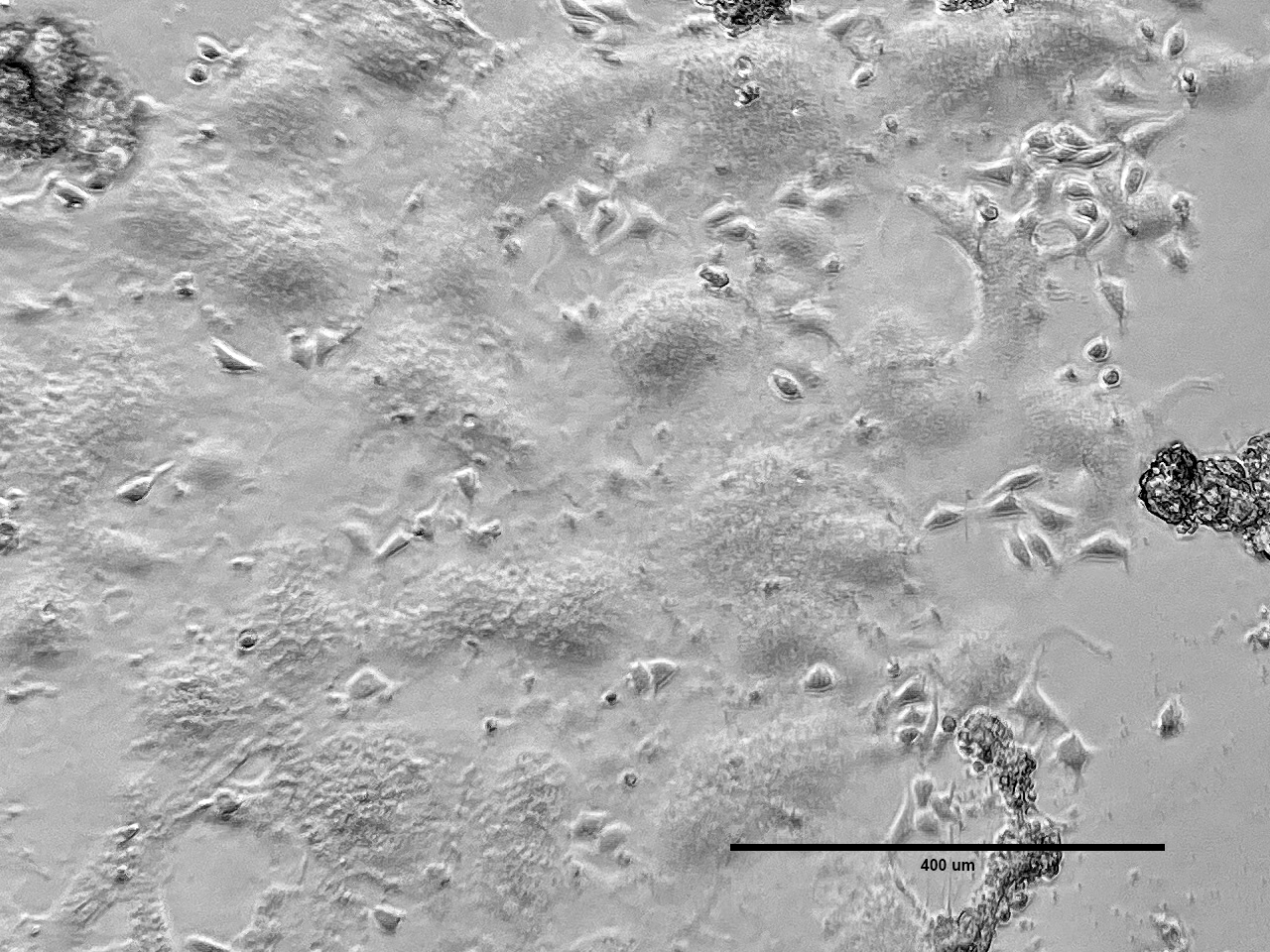

Supplement: S1 Data — This compressed folder contains the underlying numerical data and/or uncropped images used to generate the panels in Fig 1. (ZIP) [file pbio.3003736.s015.zip › S1 Data/Figure 1/K/l929-ko/l929-0.01moi.png]

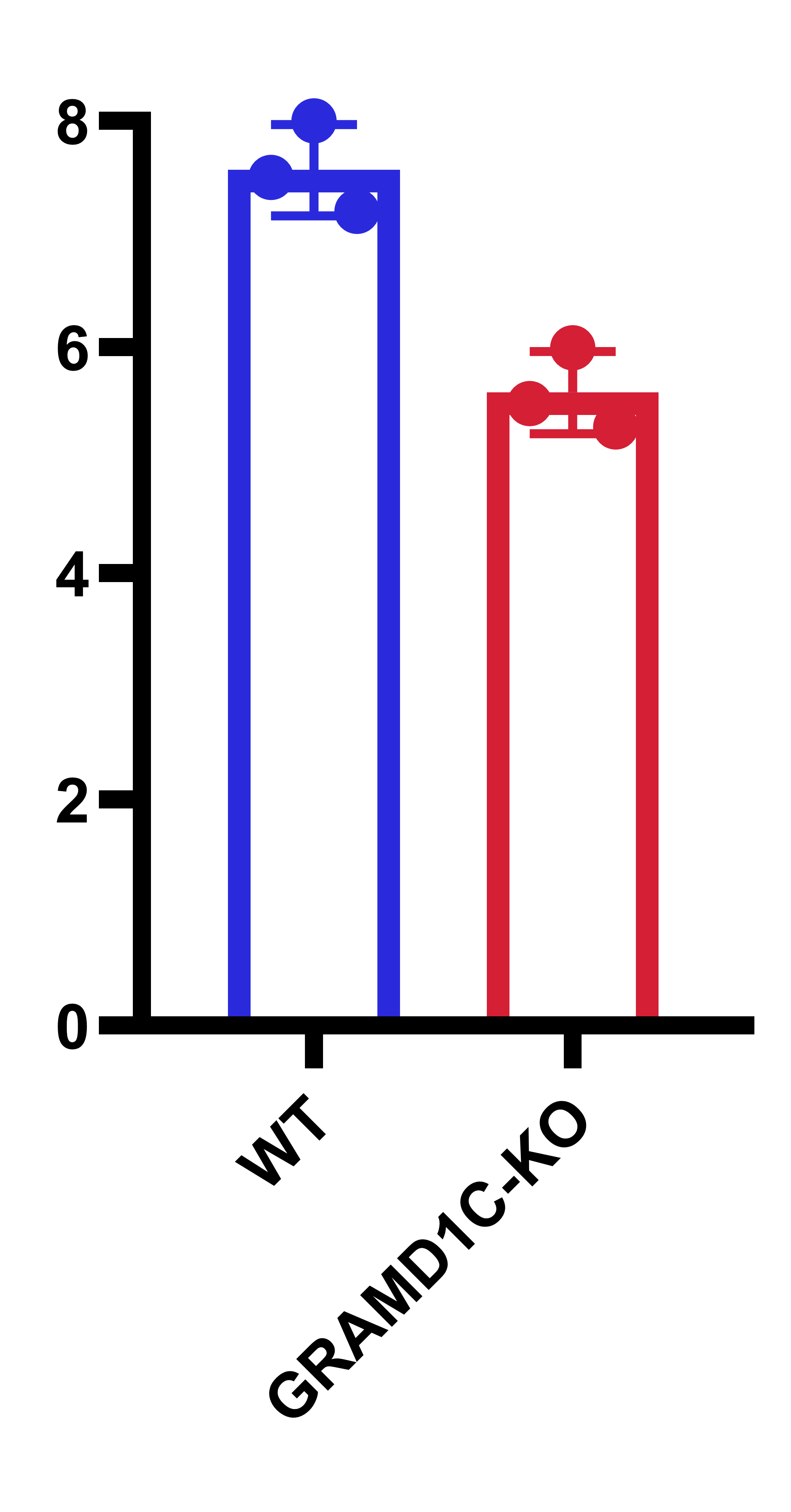

Supplement: S1 Data — This compressed folder contains the underlying numerical data and/or uncropped images used to generate the panels in Fig 1. (ZIP) [file pbio.3003736.s015.zip › S1 Data/Figure 1/K/MHV-viral-titer.tif]

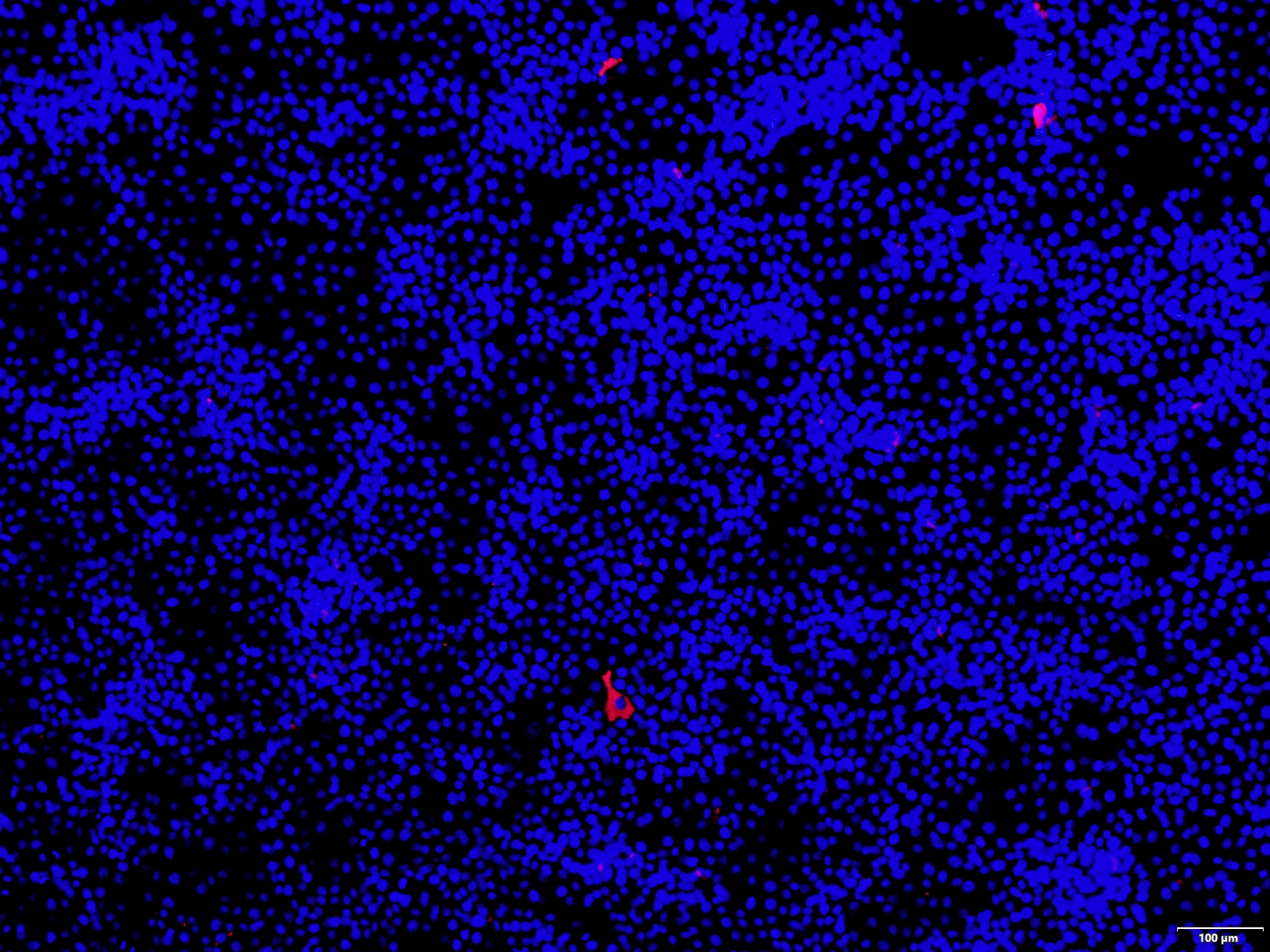

Supplement: S1 Data — This compressed folder contains the underlying numerical data and/or uncropped images used to generate the panels in Fig 1. (ZIP) [file pbio.3003736.s015.zip › S1 Data/Figure 1/L/IFA/ko/22-6-1.jpg]

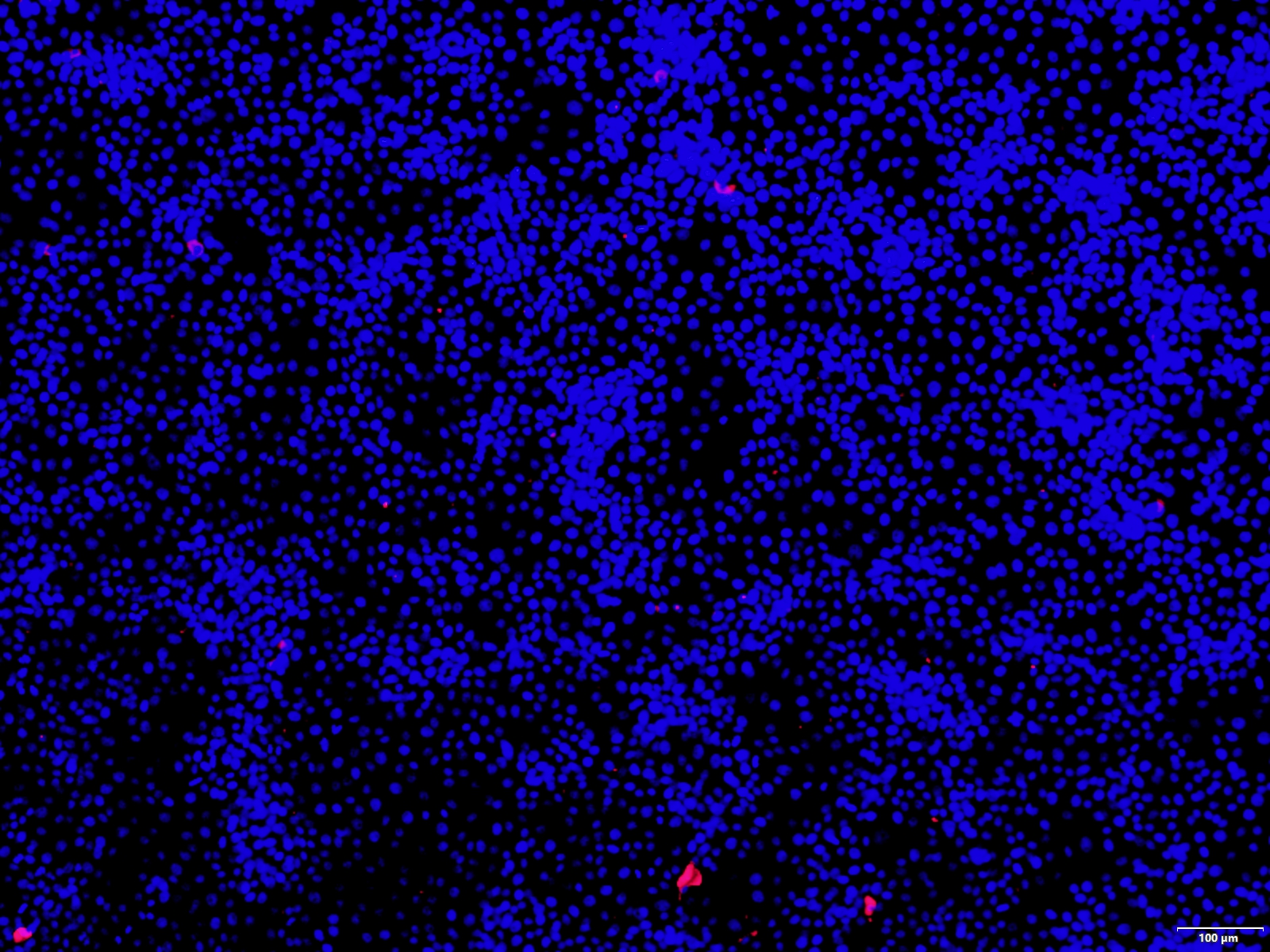

Supplement: S1 Data — This compressed folder contains the underlying numerical data and/or uncropped images used to generate the panels in Fig 1. (ZIP) [file pbio.3003736.s015.zip › S1 Data/Figure 1/L/IFA/ko/22-6-2.jpg]

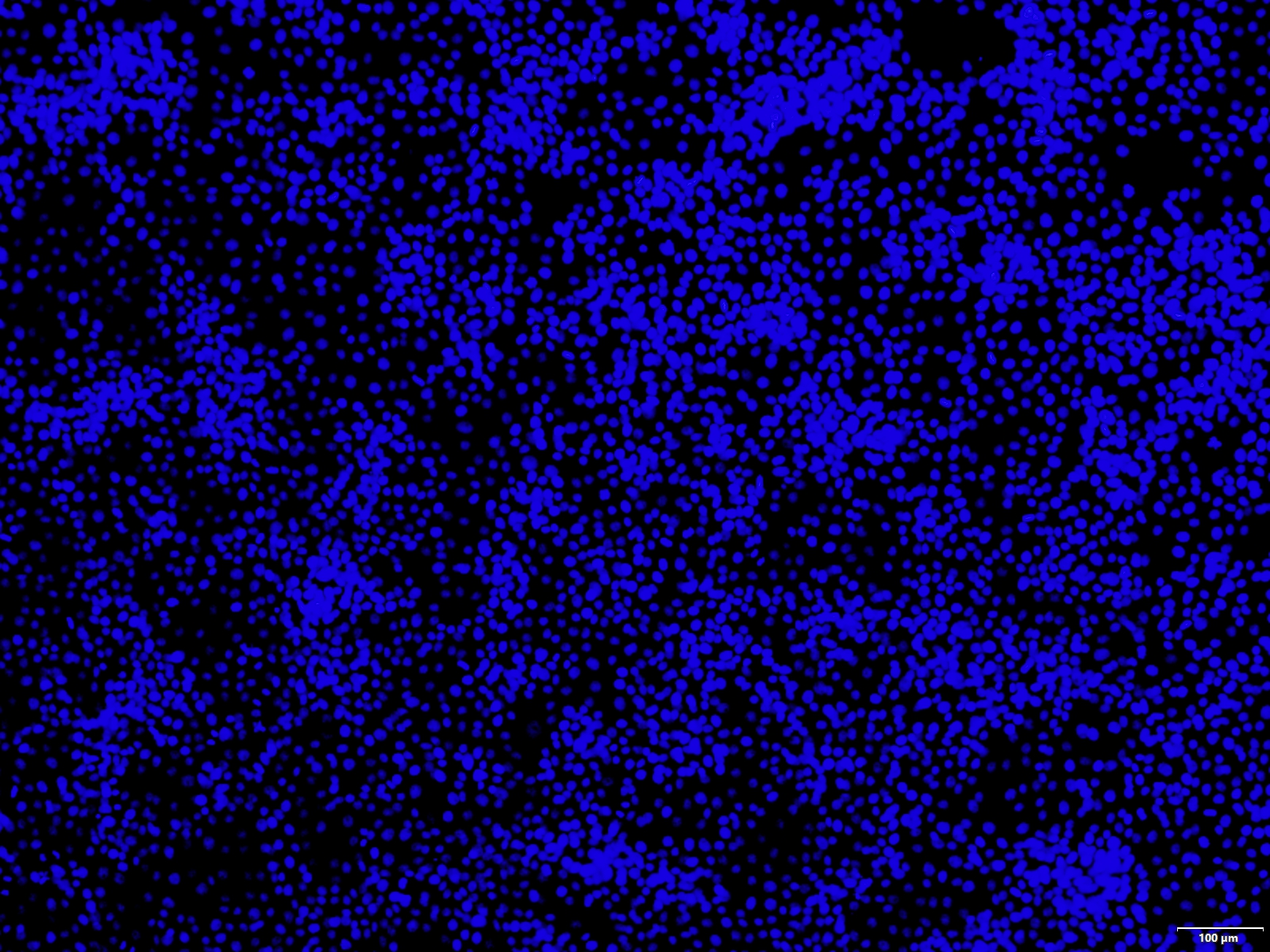

Supplement: S1 Data — This compressed folder contains the underlying numerical data and/or uncropped images used to generate the panels in Fig 1. (ZIP) [file pbio.3003736.s015.zip › S1 Data/Figure 1/L/IFA/ko/22-6-dapi-1.jpg]

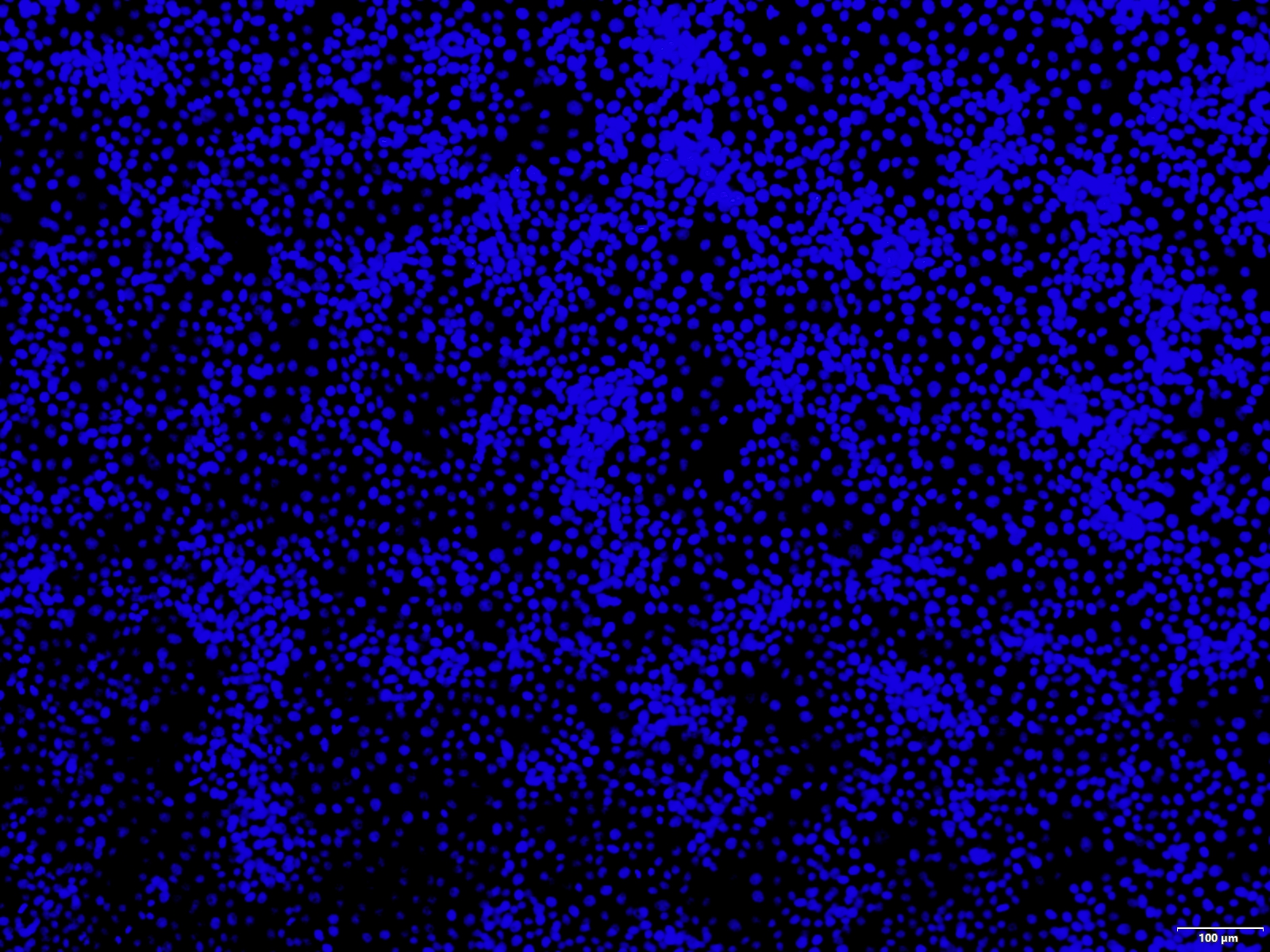

Supplement: S1 Data — This compressed folder contains the underlying numerical data and/or uncropped images used to generate the panels in Fig 1. (ZIP) [file pbio.3003736.s015.zip › S1 Data/Figure 1/L/IFA/ko/22-6-dapi-2.jpg]

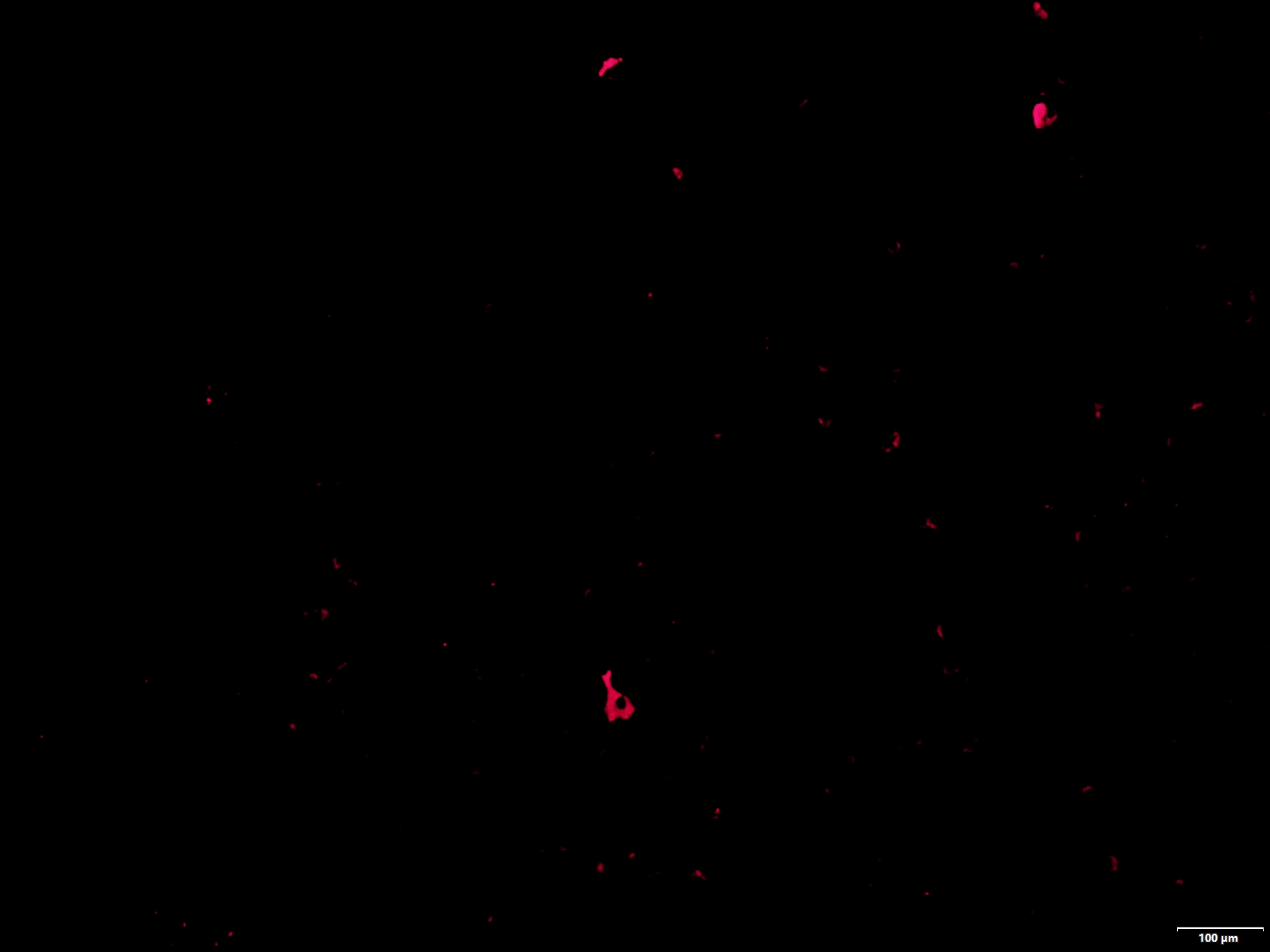

Supplement: S1 Data — This compressed folder contains the underlying numerical data and/or uncropped images used to generate the panels in Fig 1. (ZIP) [file pbio.3003736.s015.zip › S1 Data/Figure 1/L/IFA/ko/22-6-pdcov-n-1.jpg]

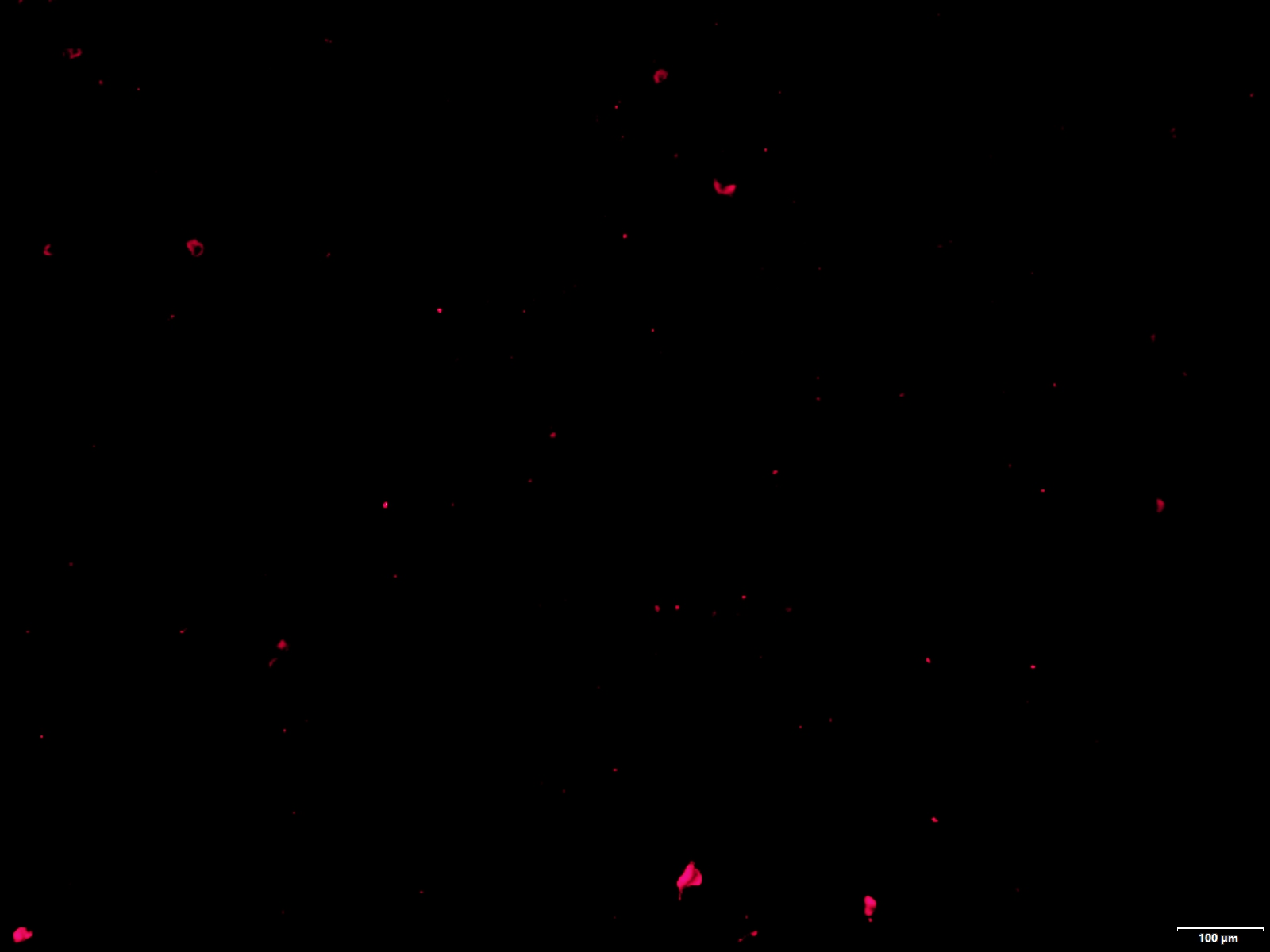

Supplement: S1 Data — This compressed folder contains the underlying numerical data and/or uncropped images used to generate the panels in Fig 1. (ZIP) [file pbio.3003736.s015.zip › S1 Data/Figure 1/L/IFA/ko/22-6-pdcov-n-2.jpg]

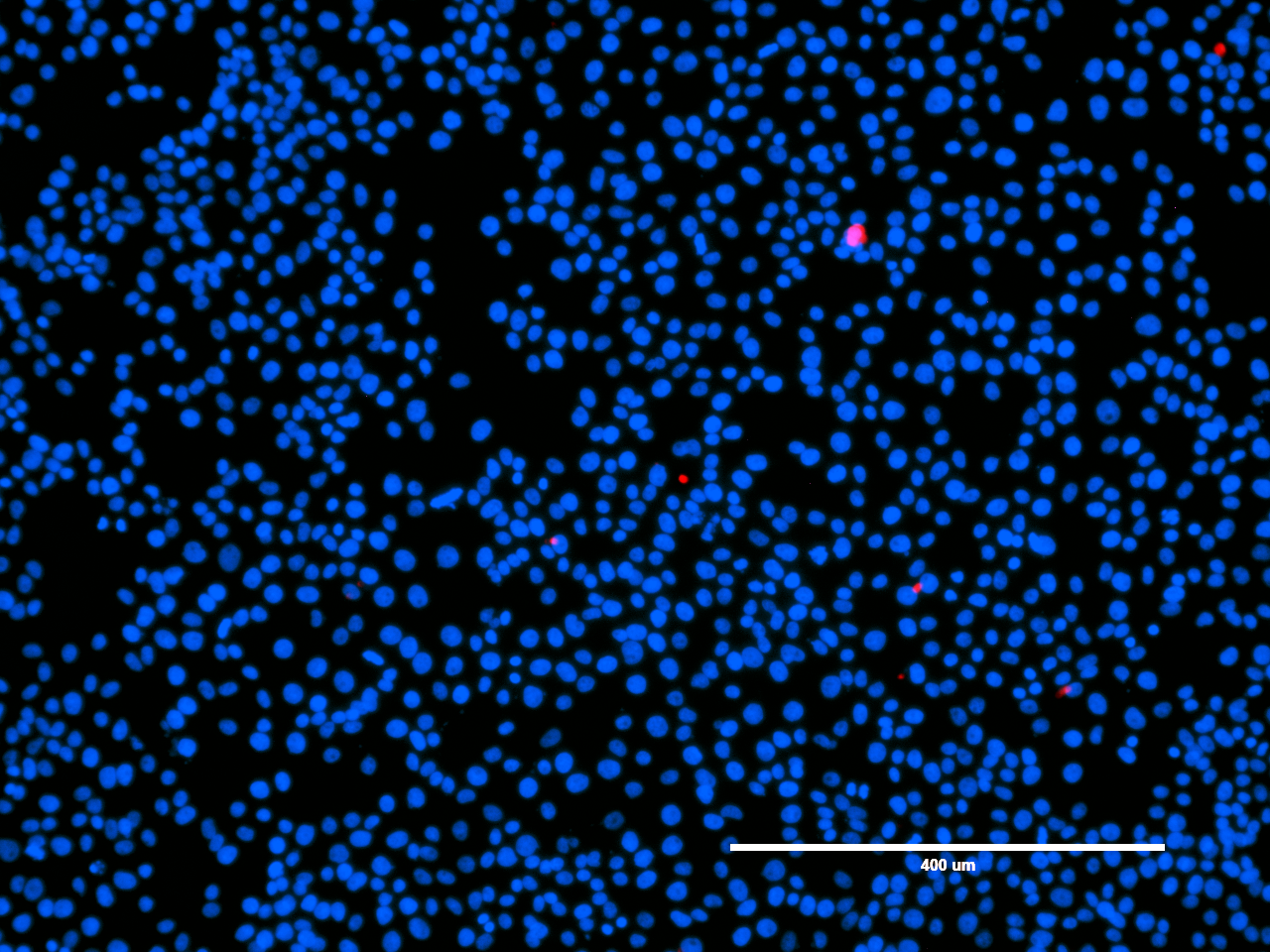

Supplement: S1 Data — This compressed folder contains the underlying numerical data and/or uncropped images used to generate the panels in Fig 1. (ZIP) [file pbio.3003736.s015.zip › S1 Data/Figure 1/L/IFA/ko/ko-5ug-5moi-24h-delta-1.png]

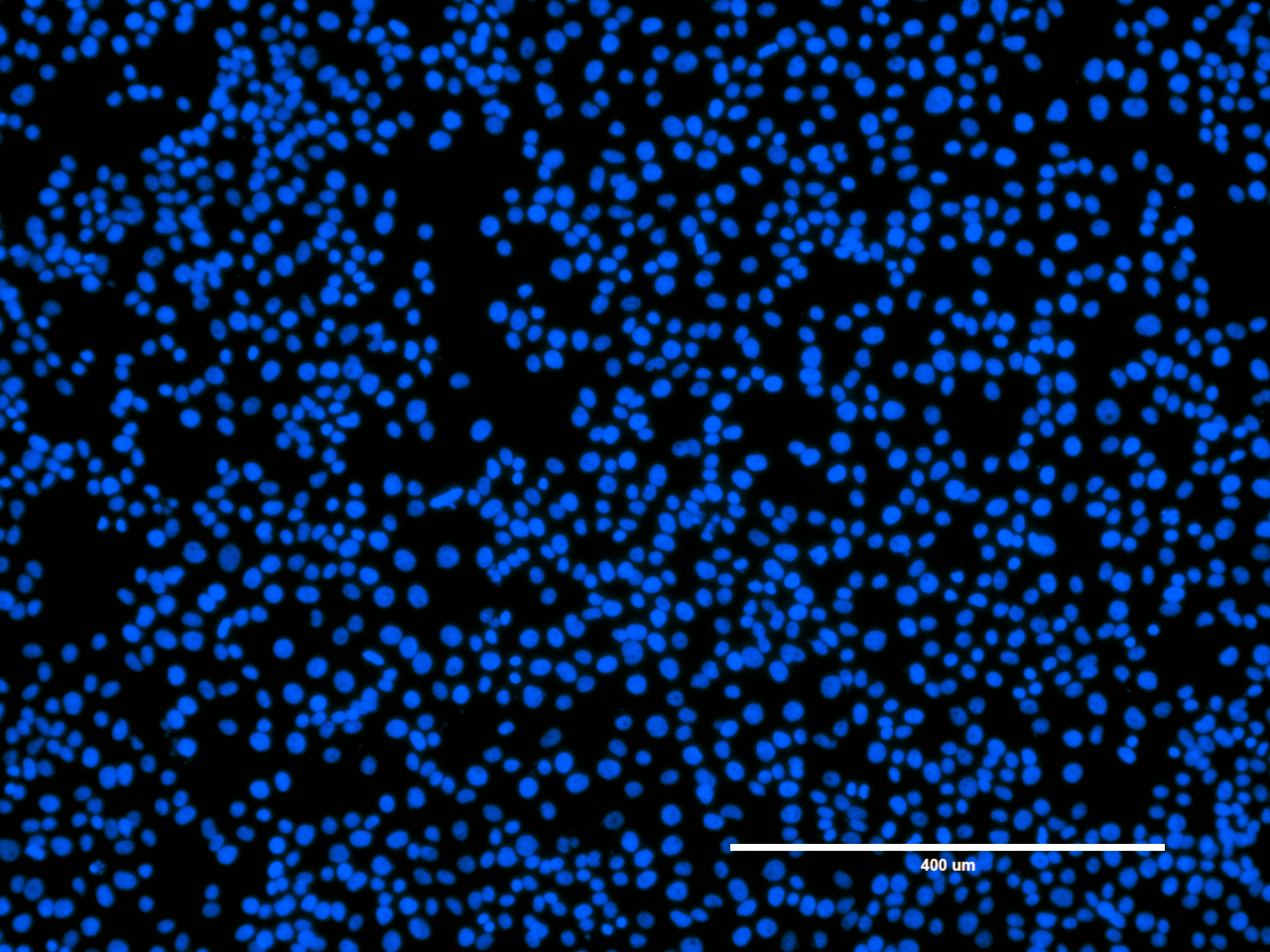

Supplement: S1 Data — This compressed folder contains the underlying numerical data and/or uncropped images used to generate the panels in Fig 1. (ZIP) [file pbio.3003736.s015.zip › S1 Data/Figure 1/L/IFA/ko/ko-5ug-5moi-24h-delta_DAPI-1.png]

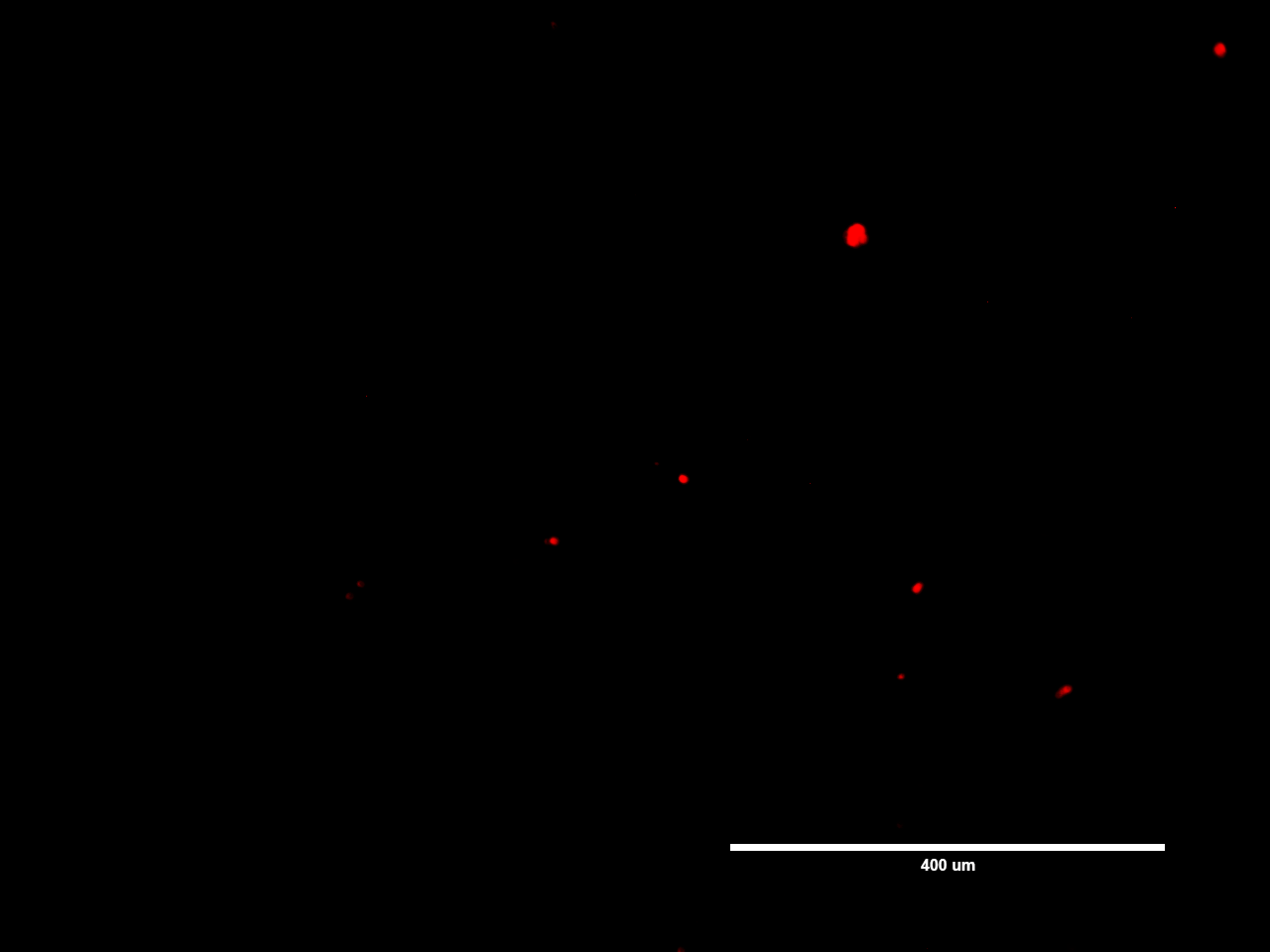

Supplement: S1 Data — This compressed folder contains the underlying numerical data and/or uncropped images used to generate the panels in Fig 1. (ZIP) [file pbio.3003736.s015.zip › S1 Data/Figure 1/L/IFA/ko/ko-5ug-5moi-24h-delta_N-1.png]

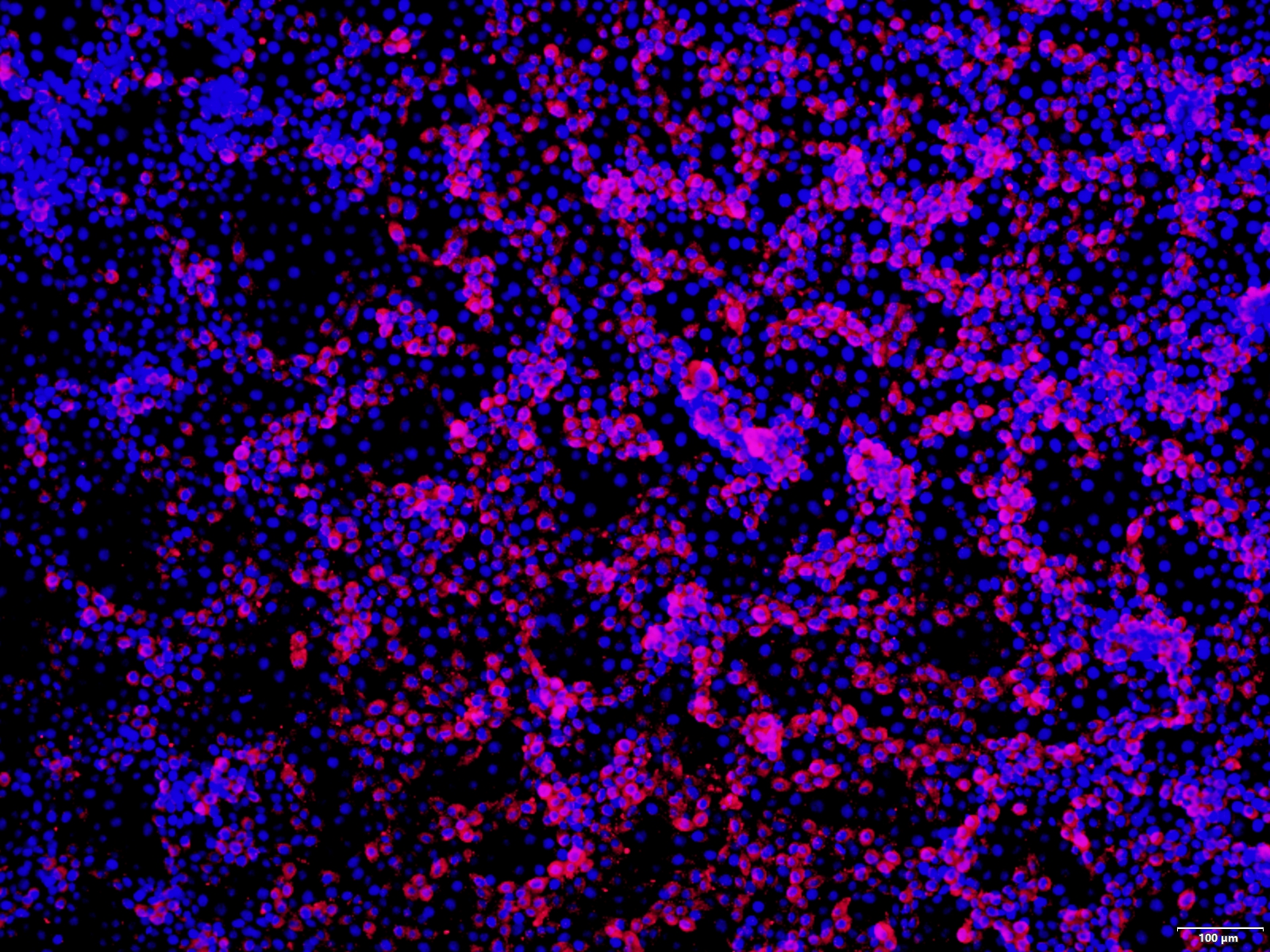

Supplement: S1 Data — This compressed folder contains the underlying numerical data and/or uncropped images used to generate the panels in Fig 1. (ZIP) [file pbio.3003736.s015.zip › S1 Data/Figure 1/L/IFA/PK/pk-2.jpg]

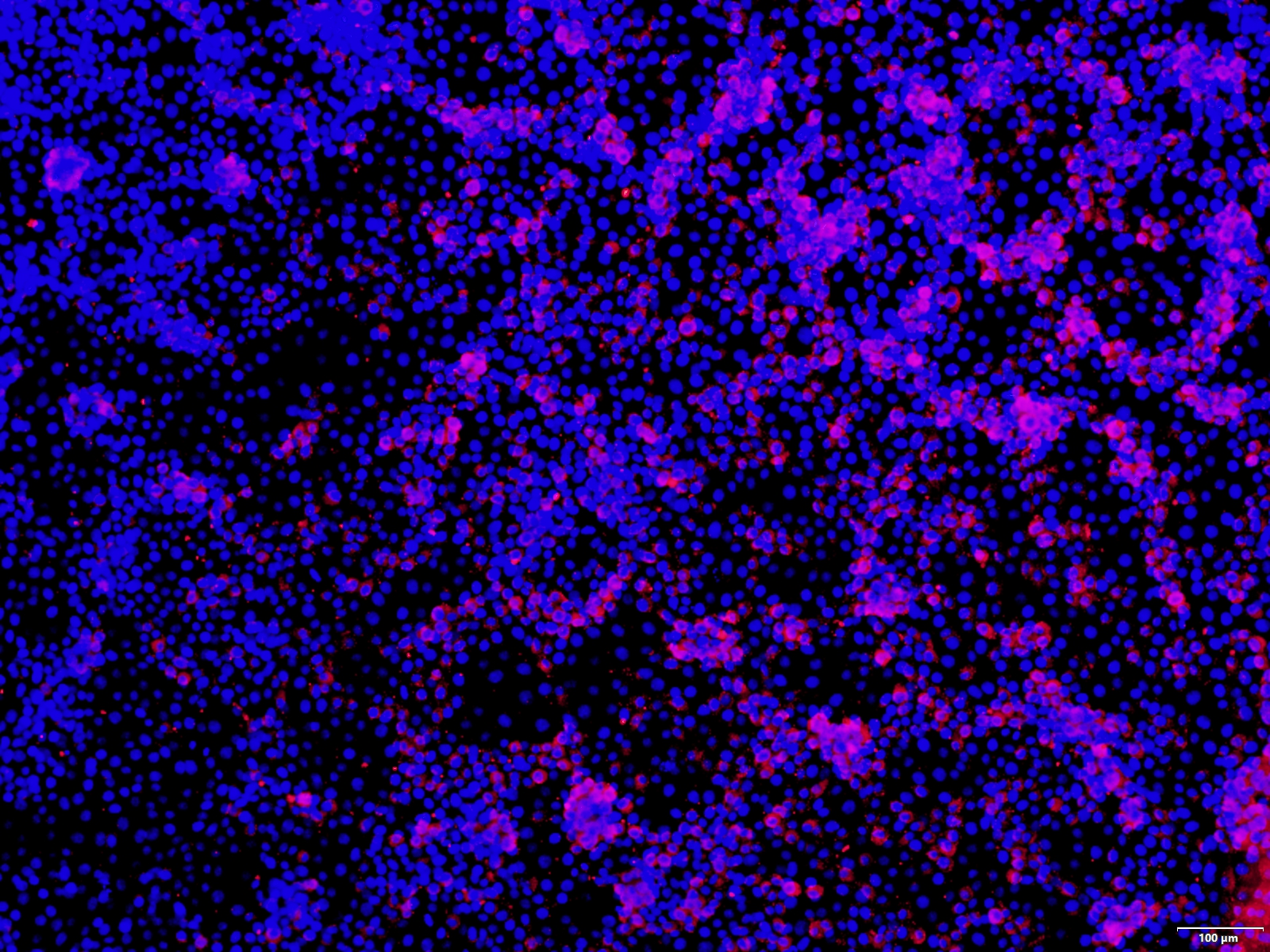

Supplement: S1 Data — This compressed folder contains the underlying numerical data and/or uncropped images used to generate the panels in Fig 1. (ZIP) [file pbio.3003736.s015.zip › S1 Data/Figure 1/L/IFA/PK/pk-3.jpg]

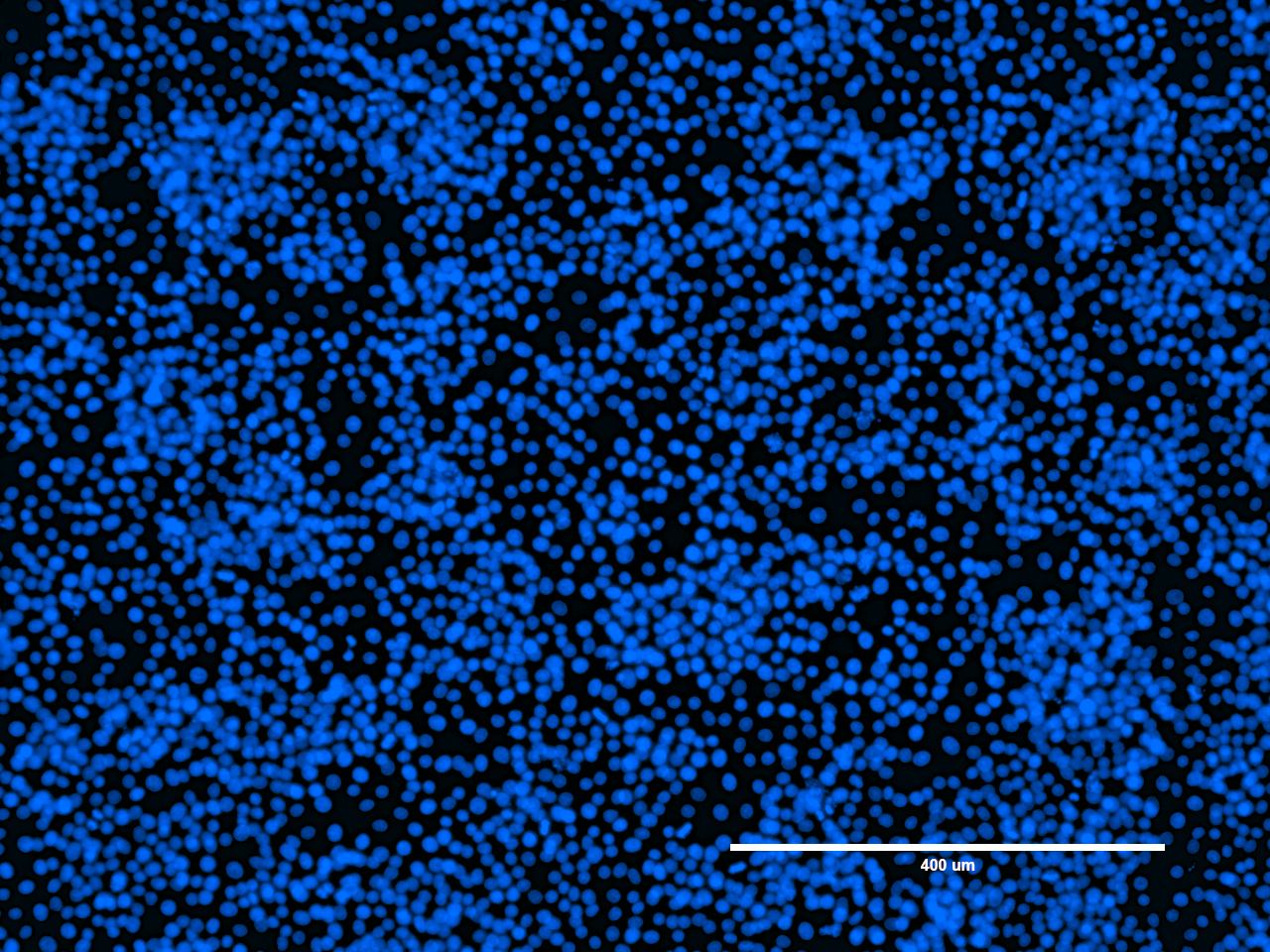

Supplement: S1 Data — This compressed folder contains the underlying numerical data and/or uncropped images used to generate the panels in Fig 1. (ZIP) [file pbio.3003736.s015.zip › S1 Data/Figure 1/L/IFA/PK/pk-5ug-5moi-24h-delta_DAPInew.png]

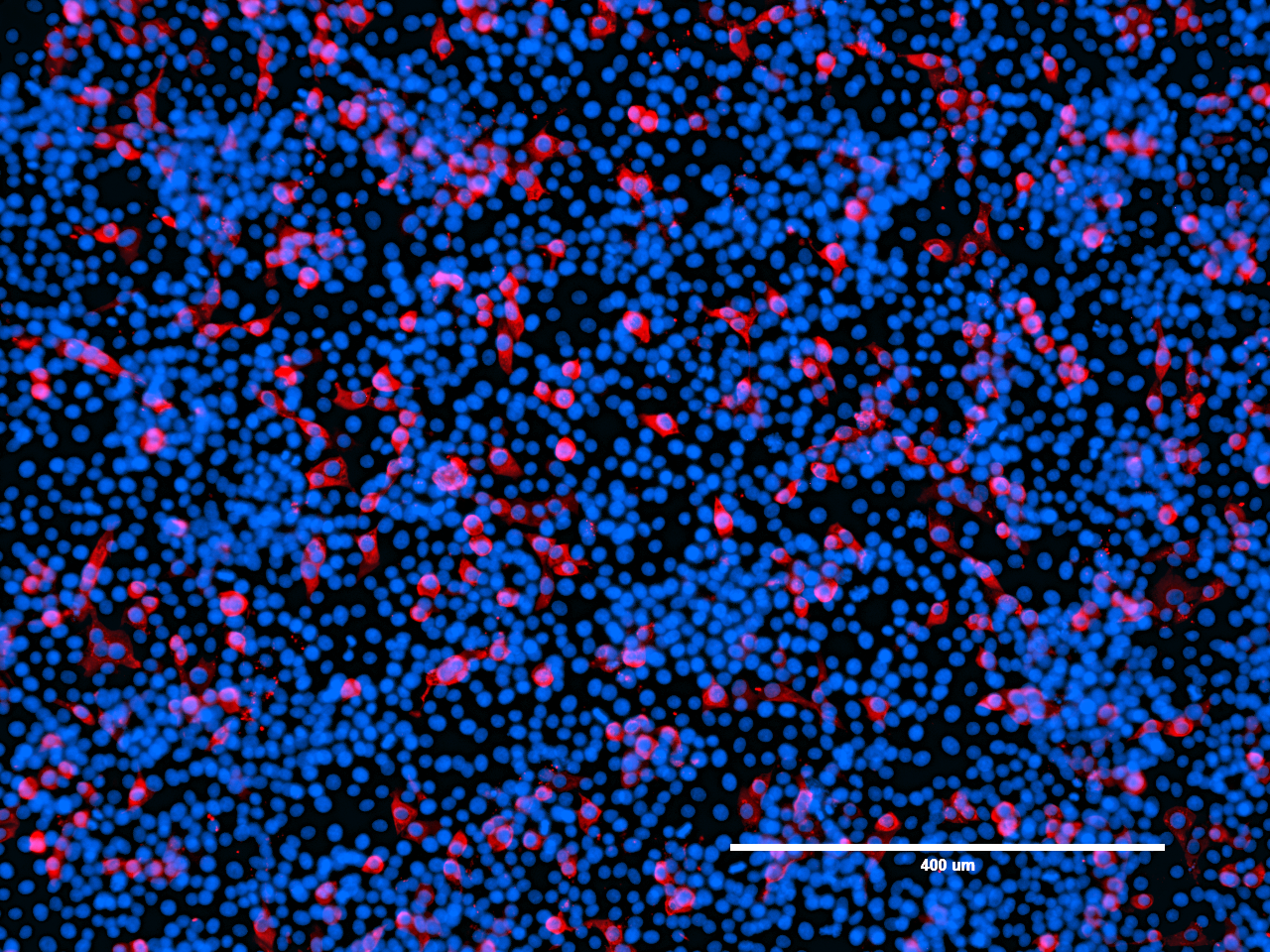

Supplement: S1 Data — This compressed folder contains the underlying numerical data and/or uncropped images used to generate the panels in Fig 1. (ZIP) [file pbio.3003736.s015.zip › S1 Data/Figure 1/L/IFA/PK/pk-5ug-5moi-24h-delta_new.png]

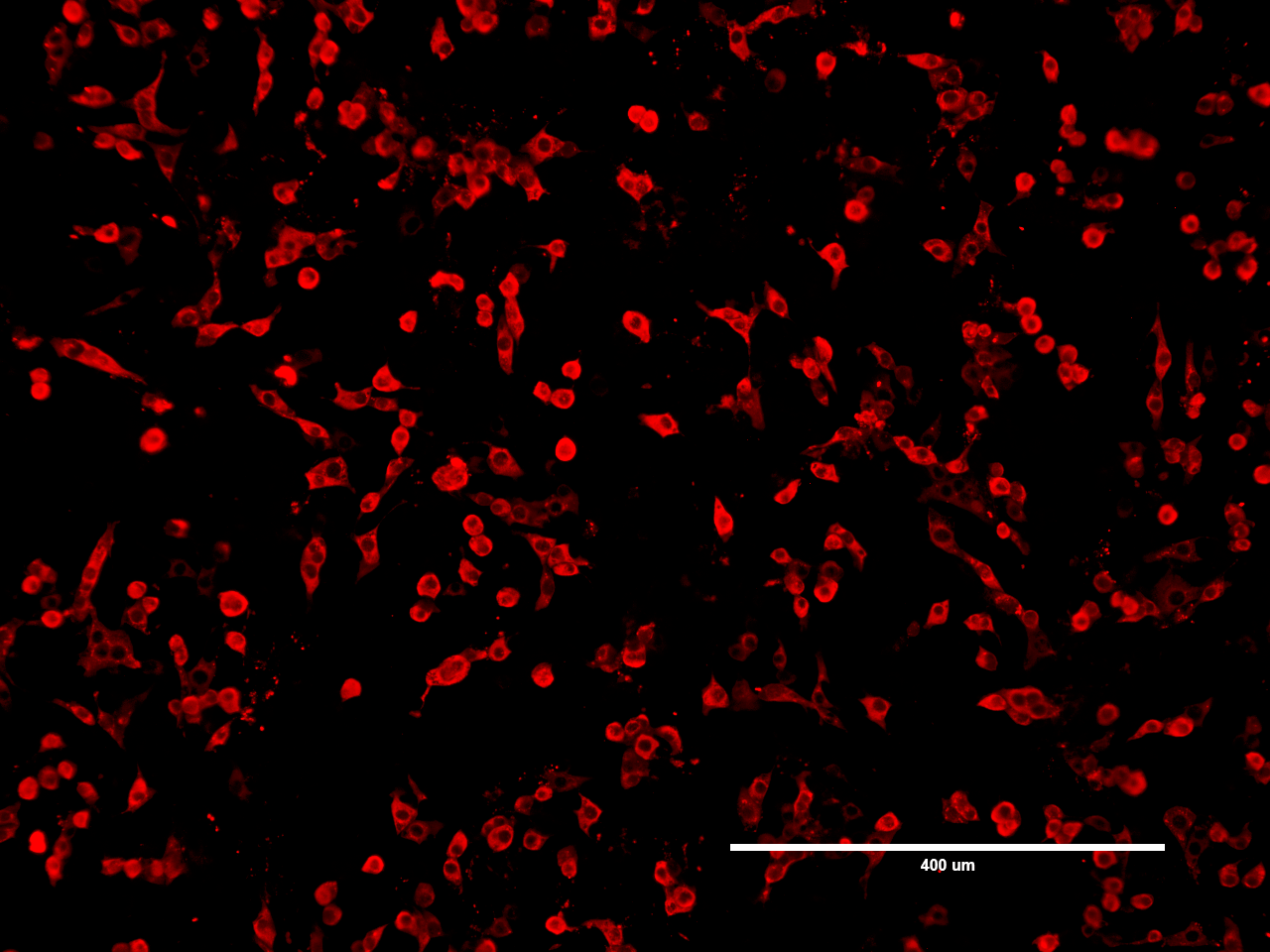

Supplement: S1 Data — This compressed folder contains the underlying numerical data and/or uncropped images used to generate the panels in Fig 1. (ZIP) [file pbio.3003736.s015.zip › S1 Data/Figure 1/L/IFA/PK/pk-5ug-5moi-24h-delta_TxRed.png]

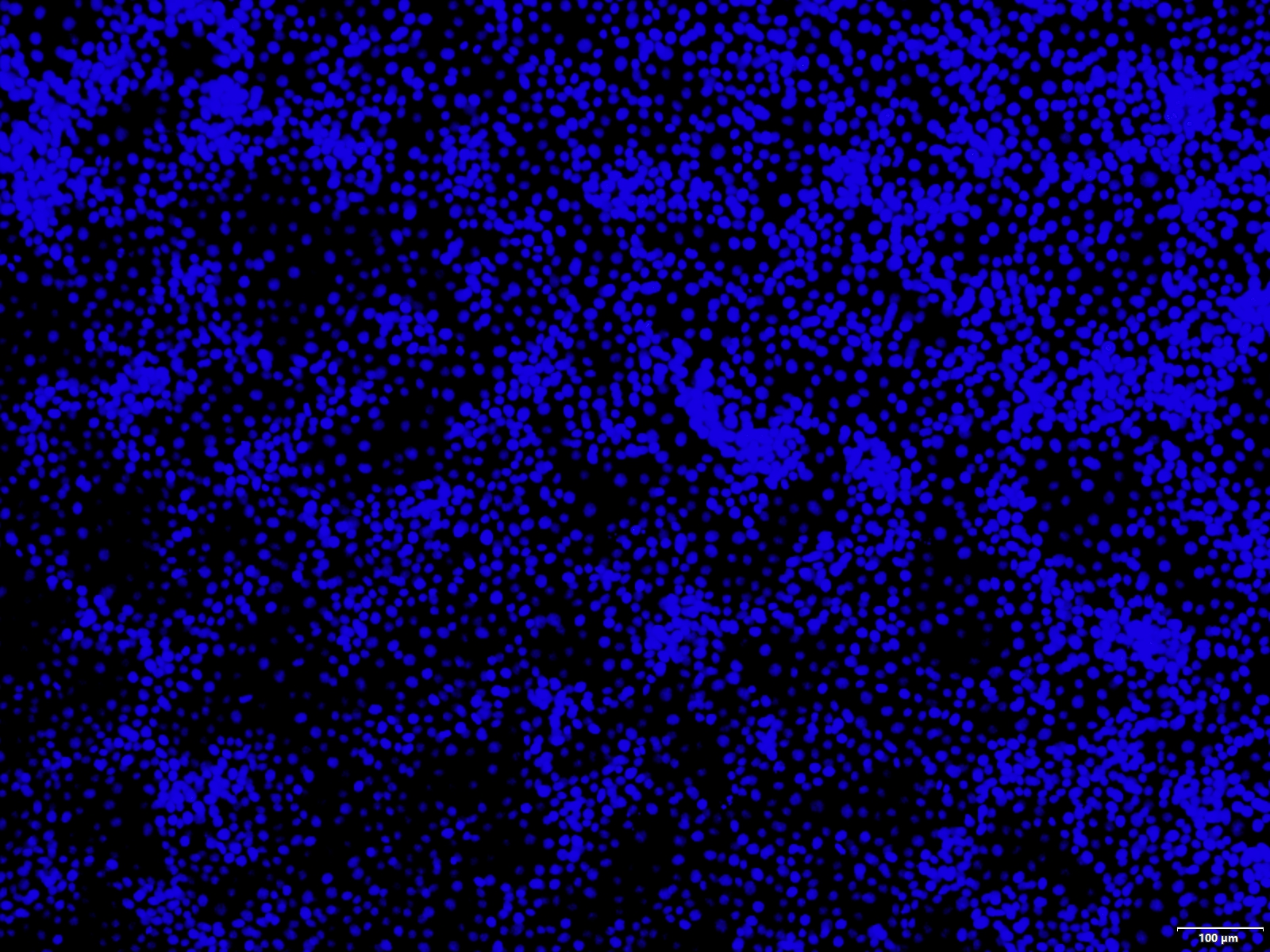

Supplement: S1 Data — This compressed folder contains the underlying numerical data and/or uncropped images used to generate the panels in Fig 1. (ZIP) [file pbio.3003736.s015.zip › S1 Data/Figure 1/L/IFA/PK/pk-dapi-2.jpg]

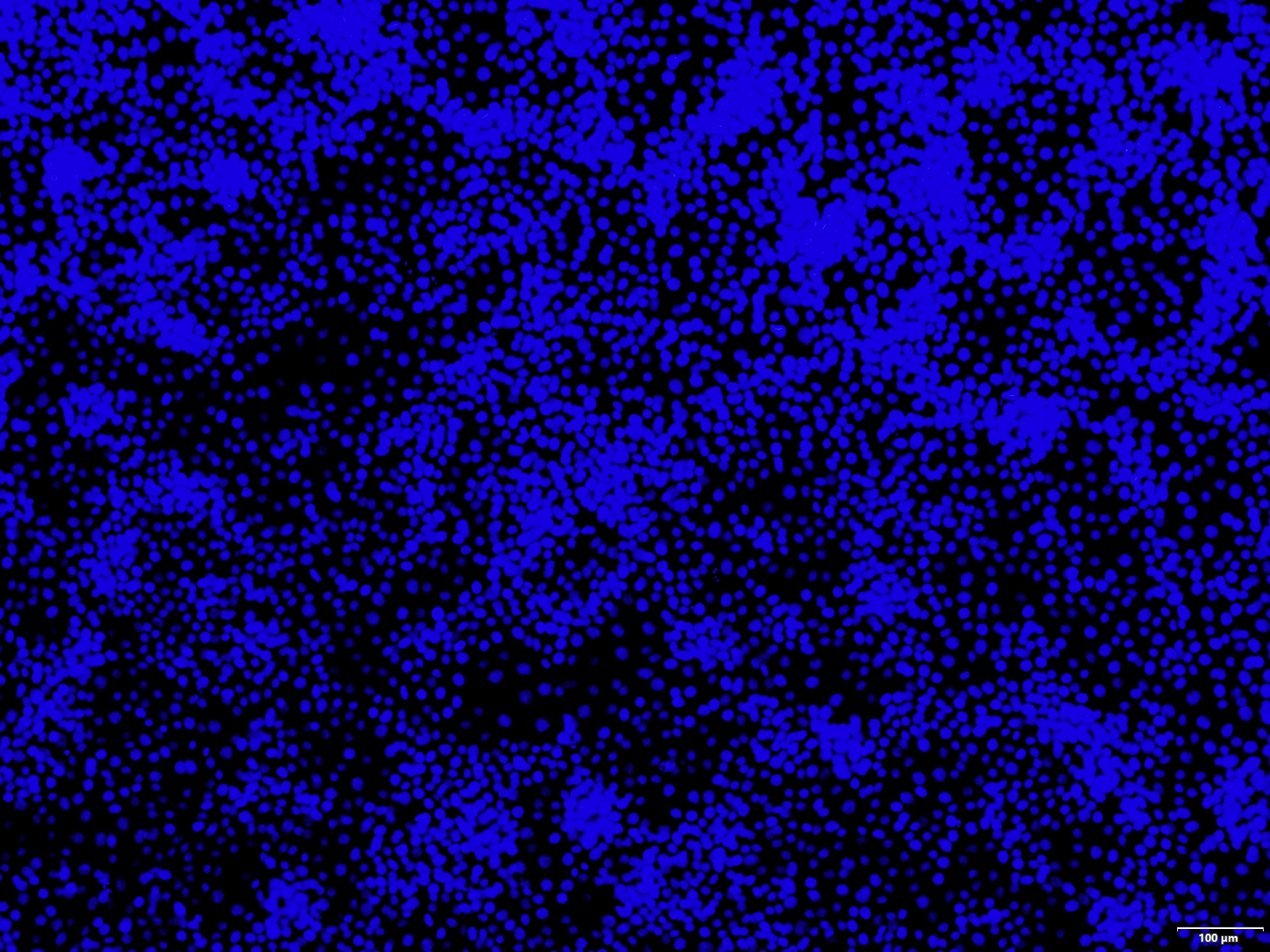

Supplement: S1 Data — This compressed folder contains the underlying numerical data and/or uncropped images used to generate the panels in Fig 1. (ZIP) [file pbio.3003736.s015.zip › S1 Data/Figure 1/L/IFA/PK/pk-dapi-3.jpg]

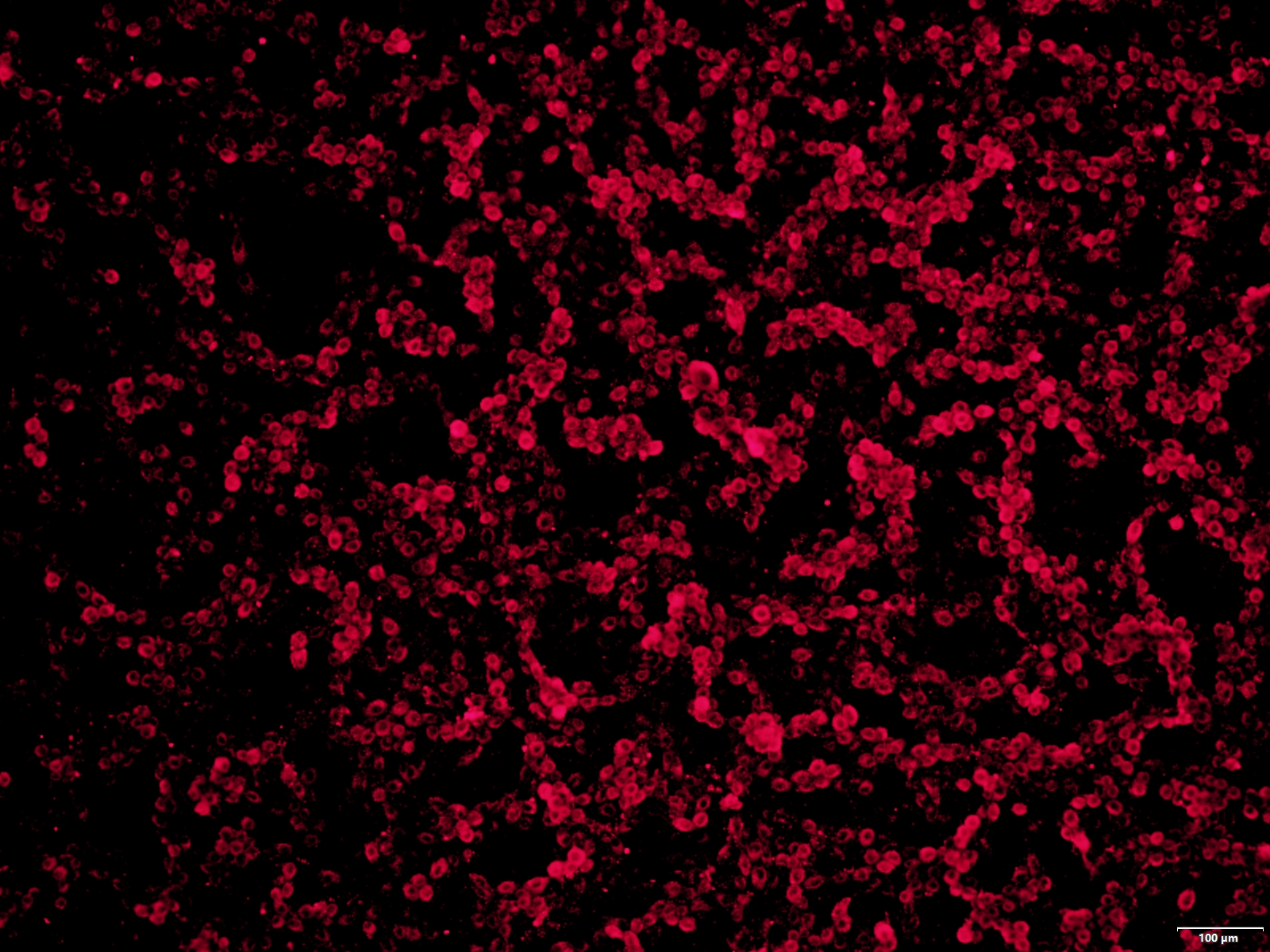

Supplement: S1 Data — This compressed folder contains the underlying numerical data and/or uncropped images used to generate the panels in Fig 1. (ZIP) [file pbio.3003736.s015.zip › S1 Data/Figure 1/L/IFA/PK/pk-pdcov-n-2.jpg]

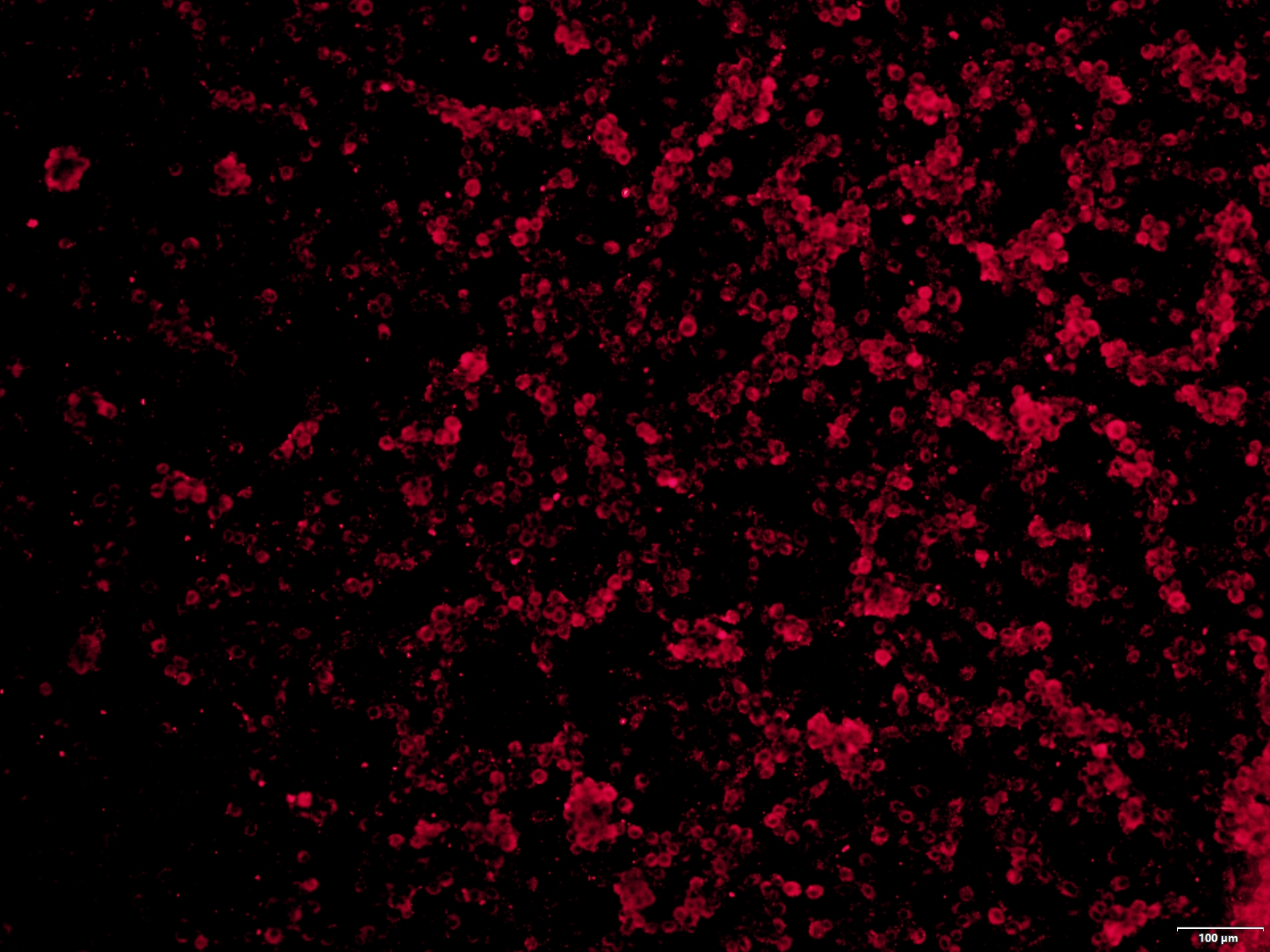

Supplement: S1 Data — This compressed folder contains the underlying numerical data and/or uncropped images used to generate the panels in Fig 1. (ZIP) [file pbio.3003736.s015.zip › S1 Data/Figure 1/L/IFA/PK/pk-pdcov-n-3.jpg]

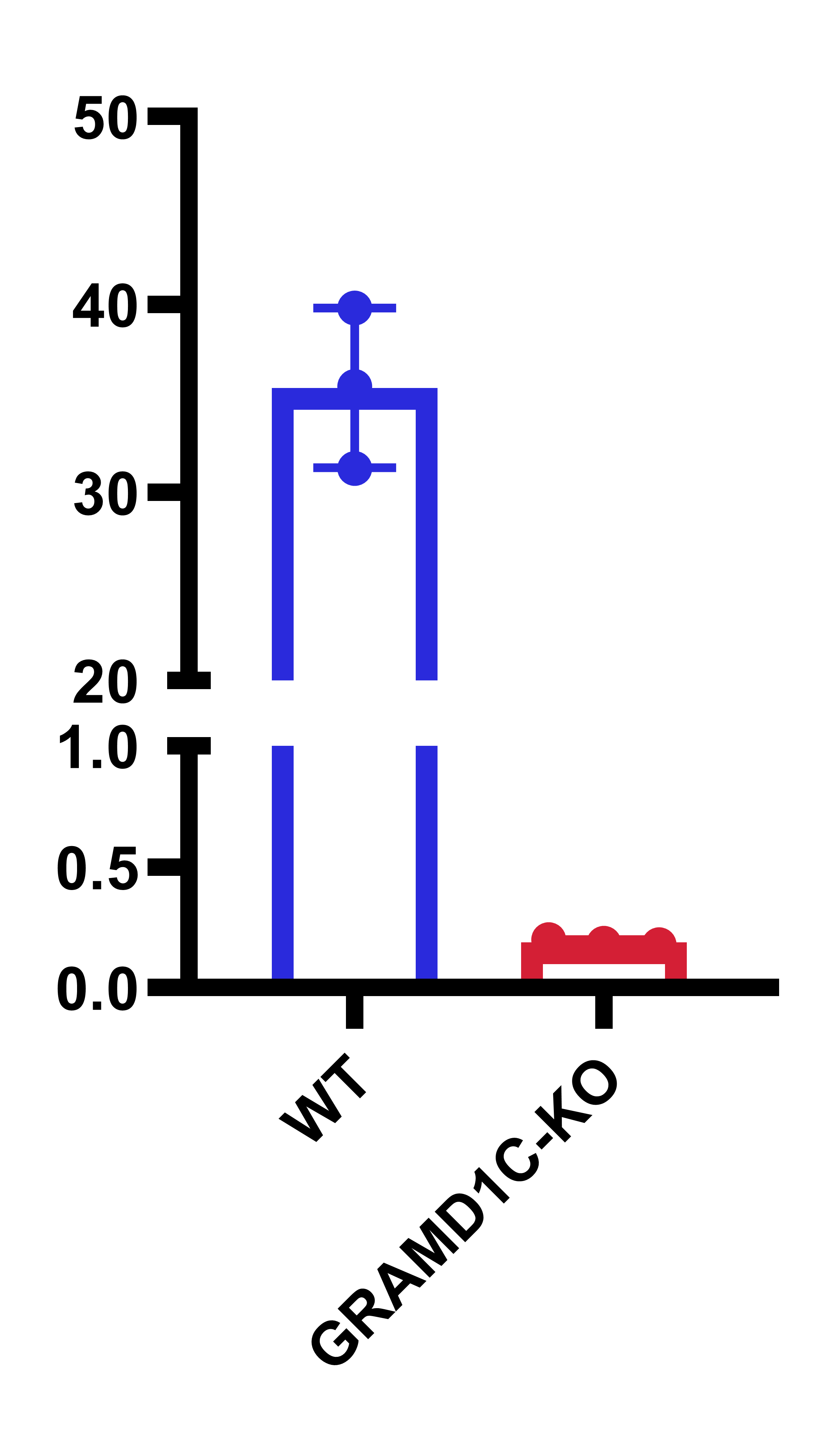

Supplement: S1 Data — This compressed folder contains the underlying numerical data and/or uncropped images used to generate the panels in Fig 1. (ZIP) [file pbio.3003736.s015.zip › S1 Data/Figure 1/L/IFA/positive-cells.tif]

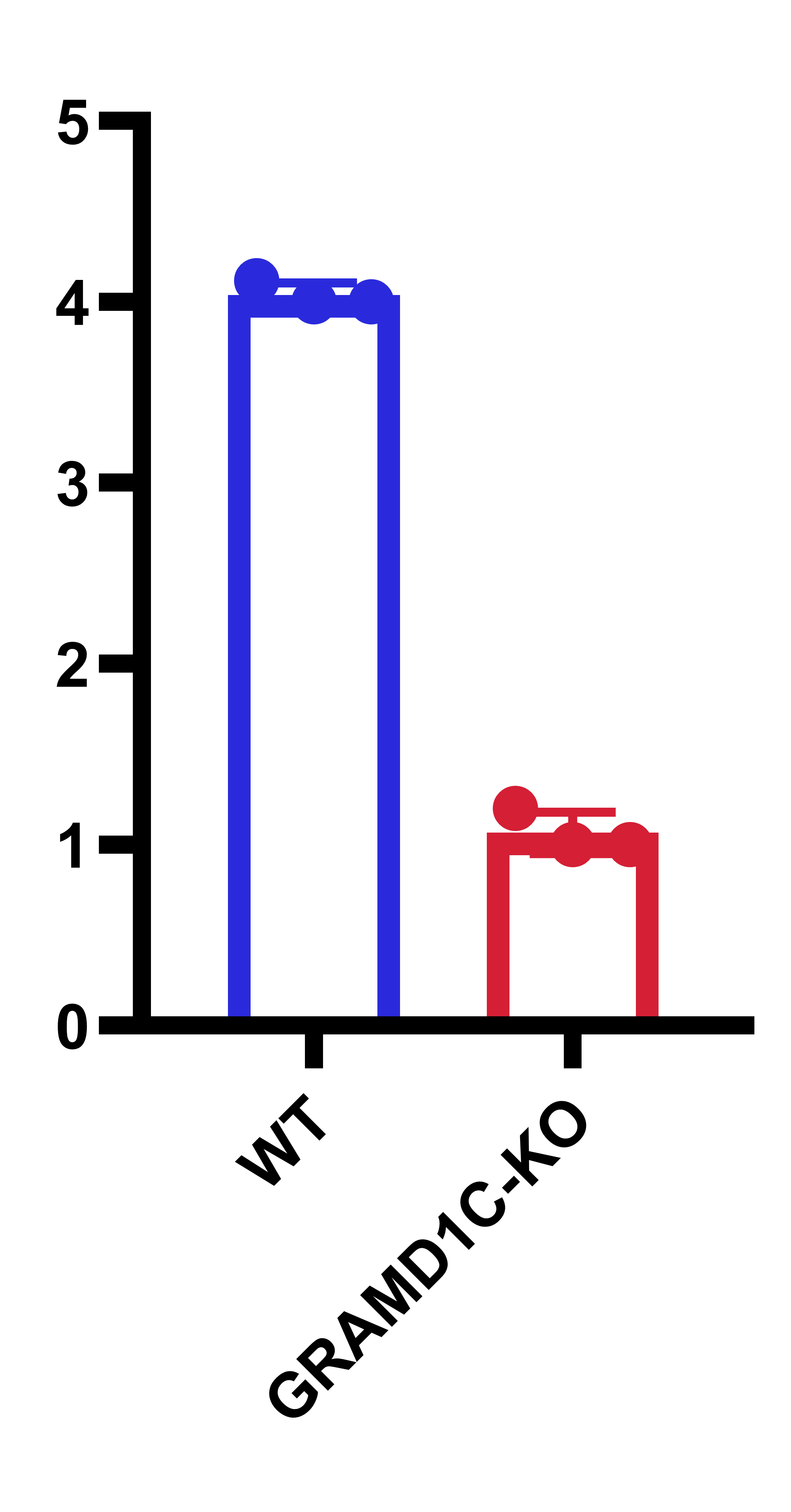

Supplement: S1 Data — This compressed folder contains the underlying numerical data and/or uncropped images used to generate the panels in Fig 1. (ZIP) [file pbio.3003736.s015.zip › S1 Data/Figure 1/L/PDCOV-titer/titer.tif]

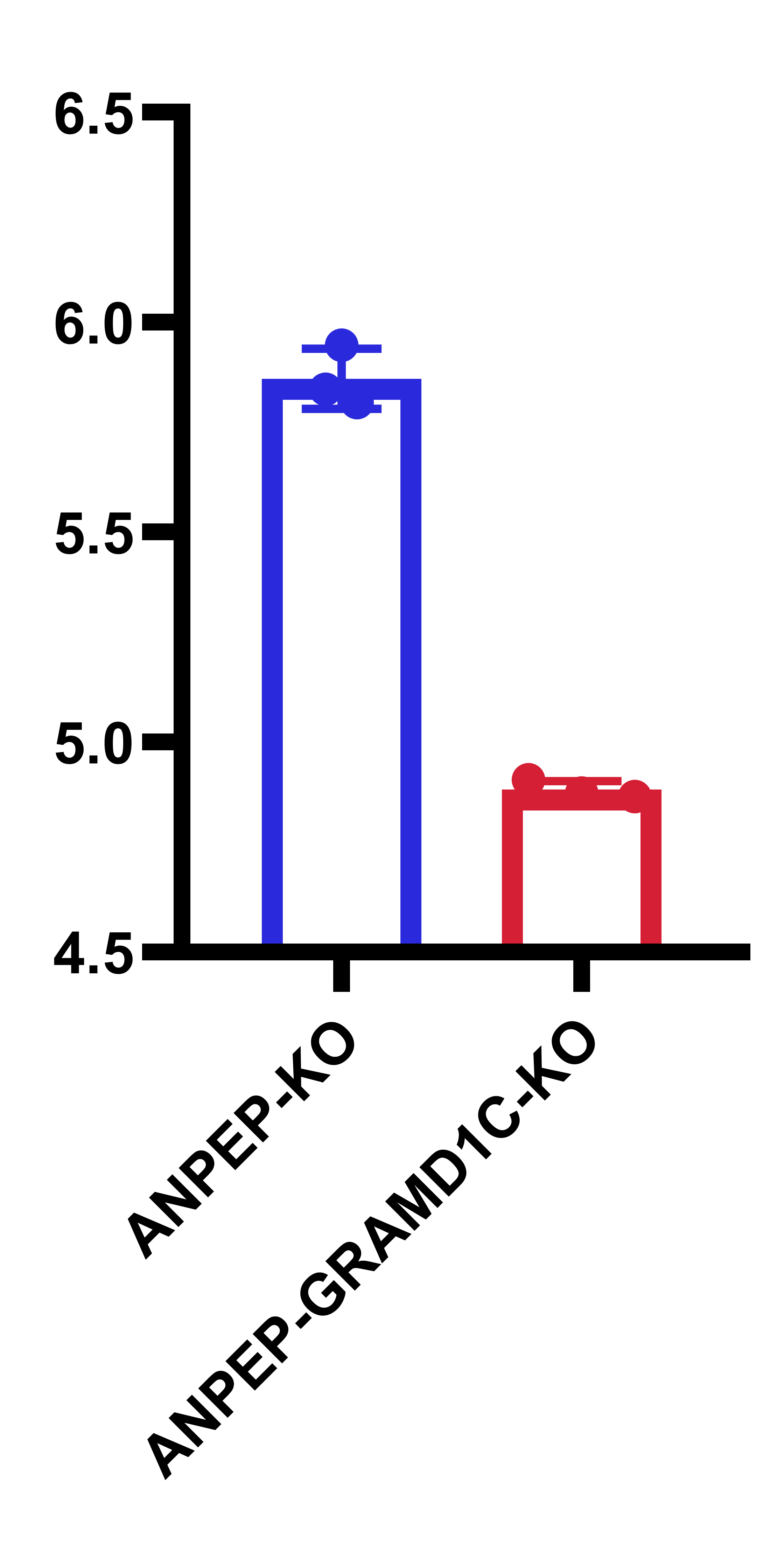

Supplement: S2 Data — This compressed folder contains the underlying numerical data and/or uncropped images used to generate the panels in Fig 2. (ZIP) [file pbio.3003736.s016.zip › S2 Data/Figure 2/B/ANPEP-1C-DKO-TGEV-BAC.tif]

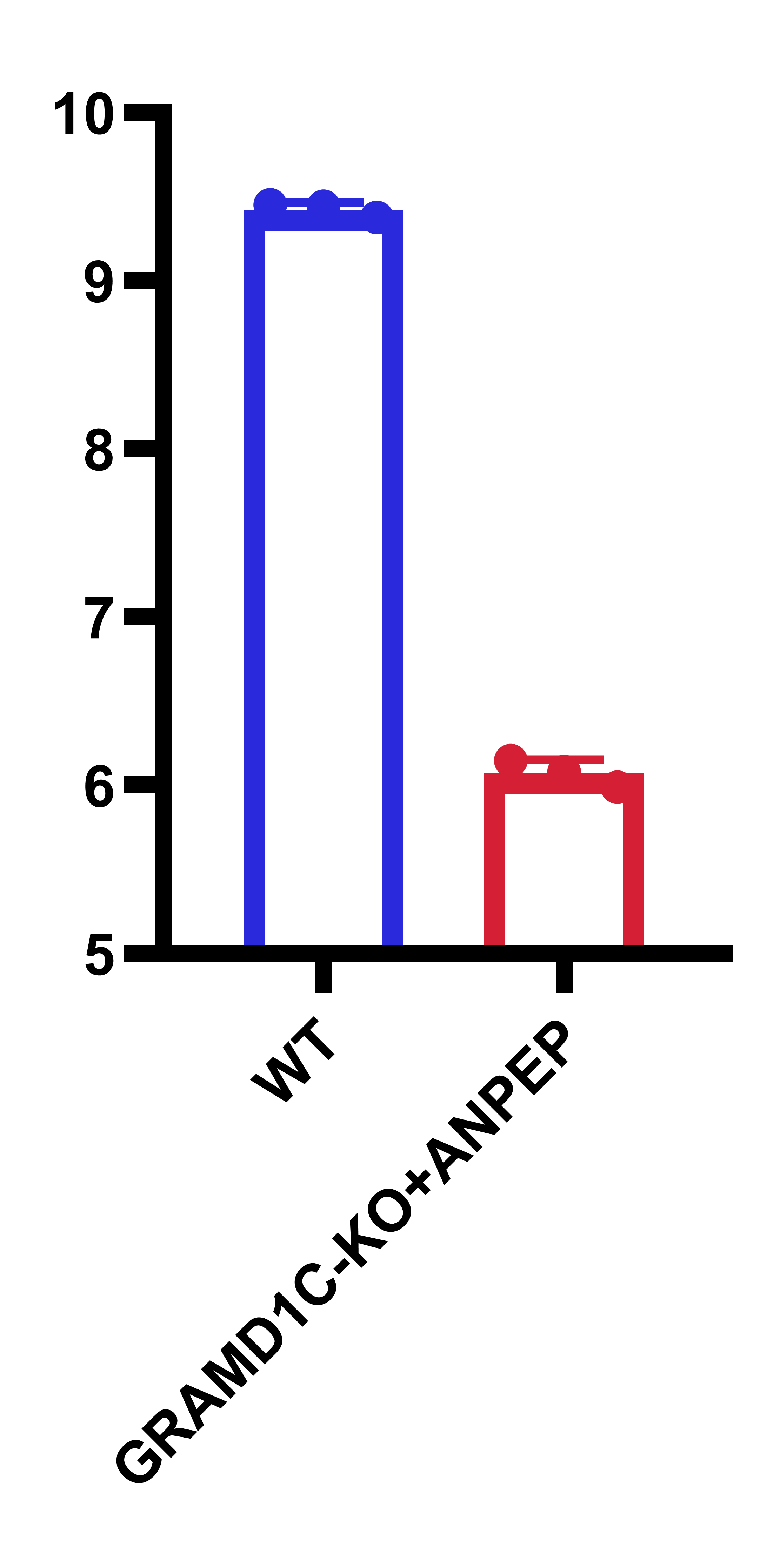

Supplement: S2 Data — This compressed folder contains the underlying numerical data and/or uncropped images used to generate the panels in Fig 2. (ZIP) [file pbio.3003736.s016.zip › S2 Data/Figure 2/C/KO+ANPEP-TGEVBAC-60H.tif]

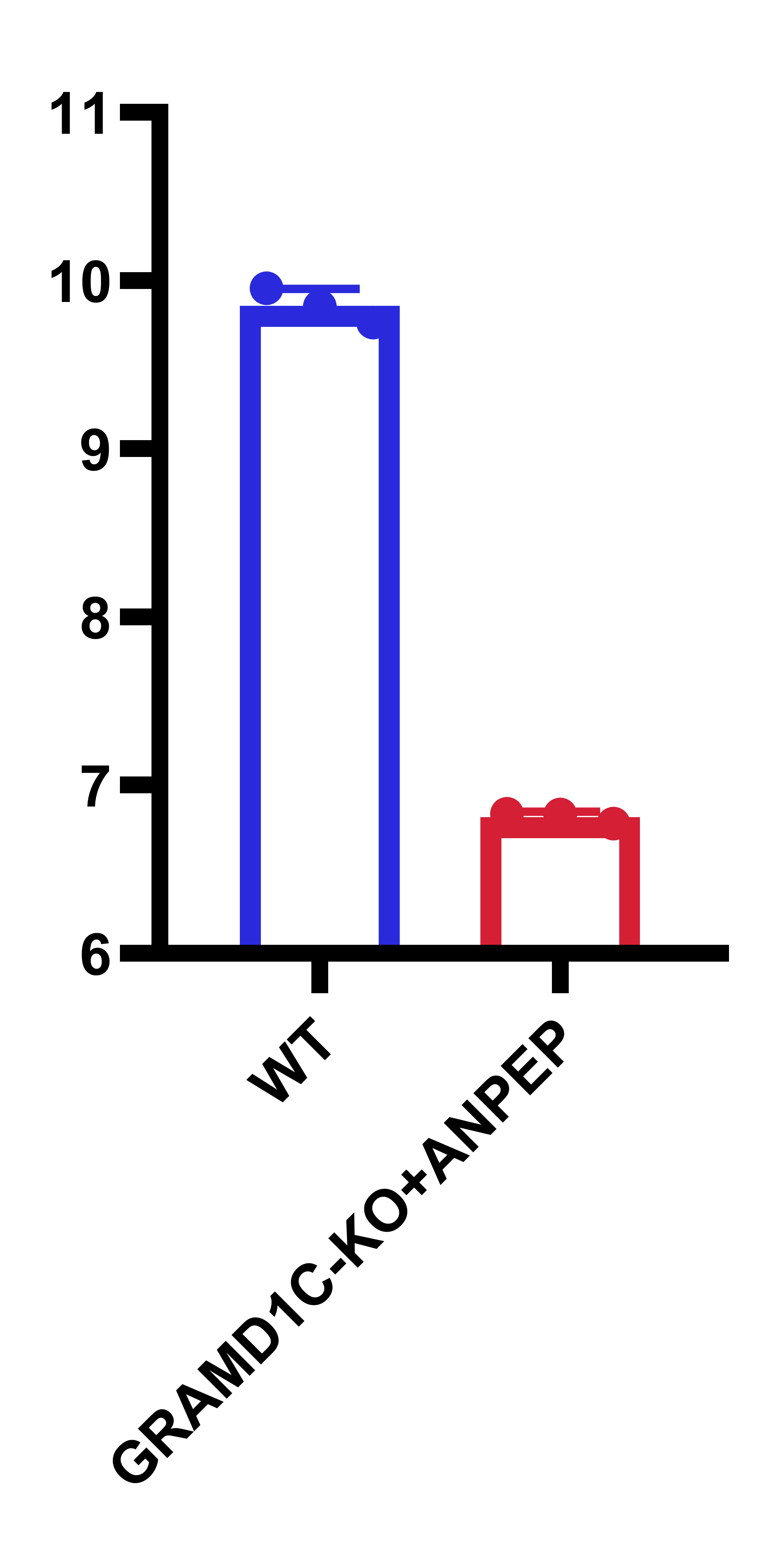

Supplement: S2 Data — This compressed folder contains the underlying numerical data and/or uncropped images used to generate the panels in Fig 2. (ZIP) [file pbio.3003736.s016.zip › S2 Data/Figure 2/D/KO+ANPEP-QPCR-TGEV-N.tif]

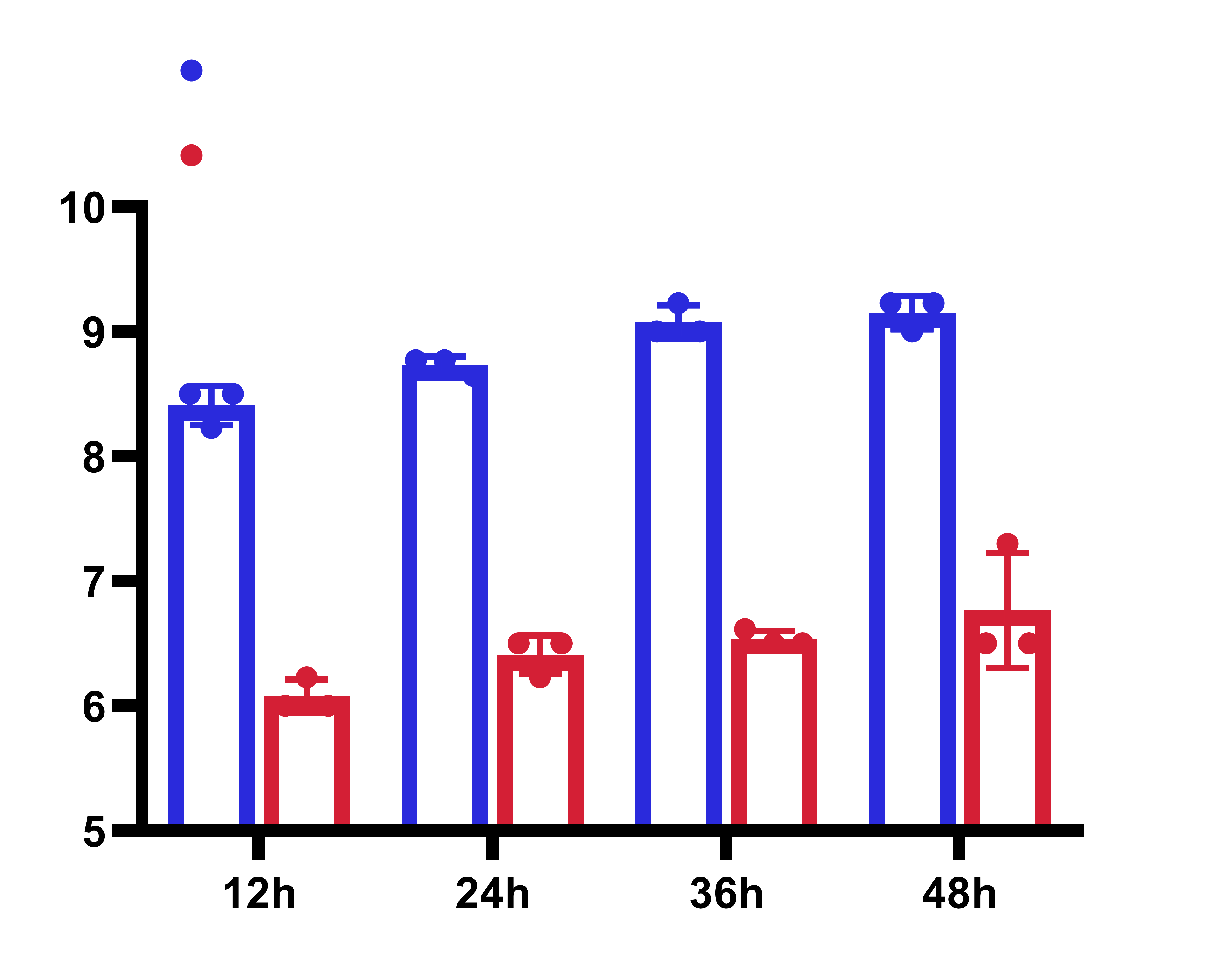

Supplement: S2 Data — This compressed folder contains the underlying numerical data and/or uncropped images used to generate the panels in Fig 2. (ZIP) [file pbio.3003736.s016.zip › S2 Data/Figure 2/E/1moi-ko_anpep-12h24h36h48h.tif]

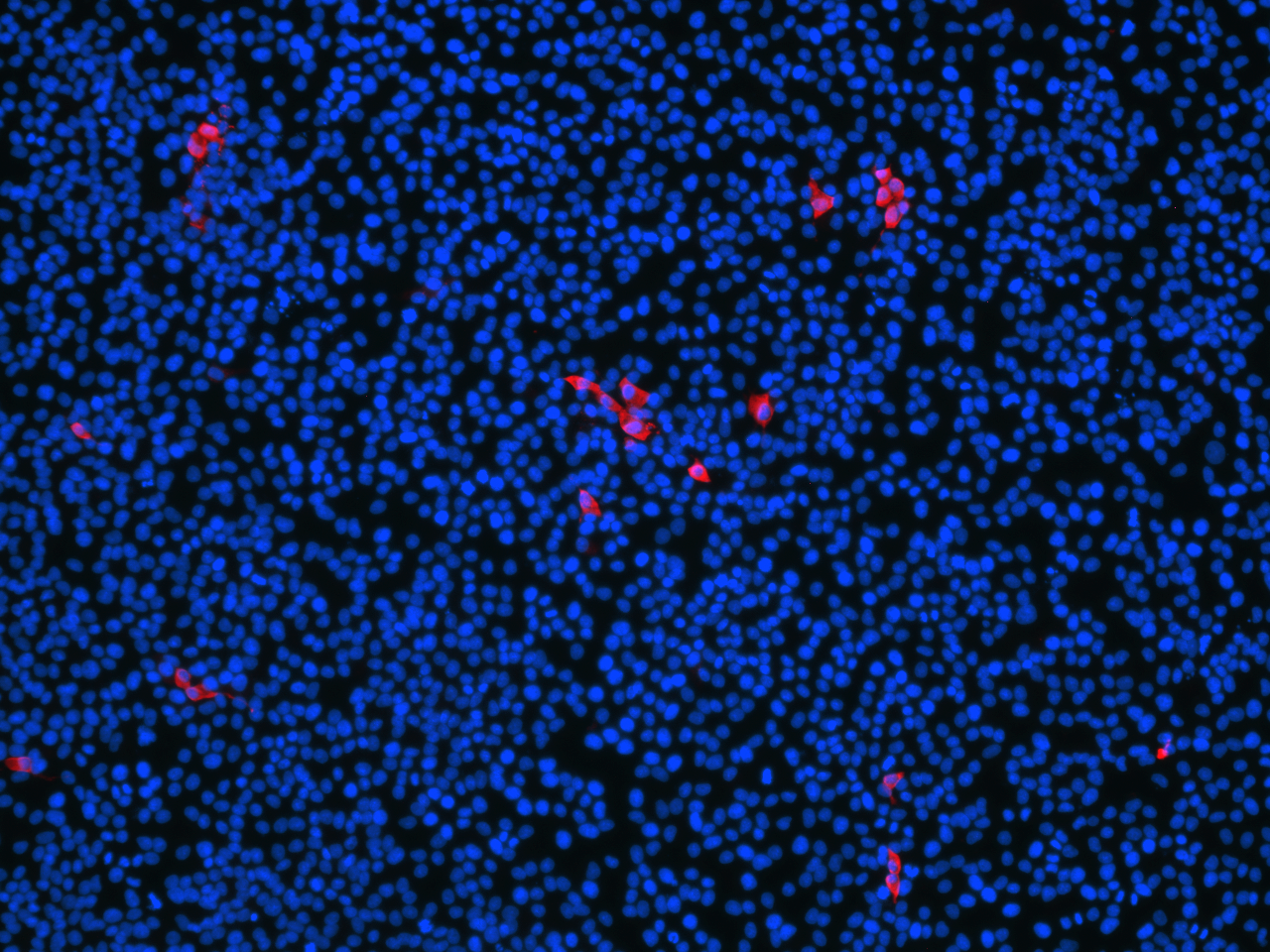

Supplement: S2 Data — This compressed folder contains the underlying numerical data and/or uncropped images used to generate the panels in Fig 2. (ZIP) [file pbio.3003736.s016.zip › S2 Data/Figure 2/F/ko+anpep/tgev-0.01moi-24h-1-14-1_DAPI-merge.png]

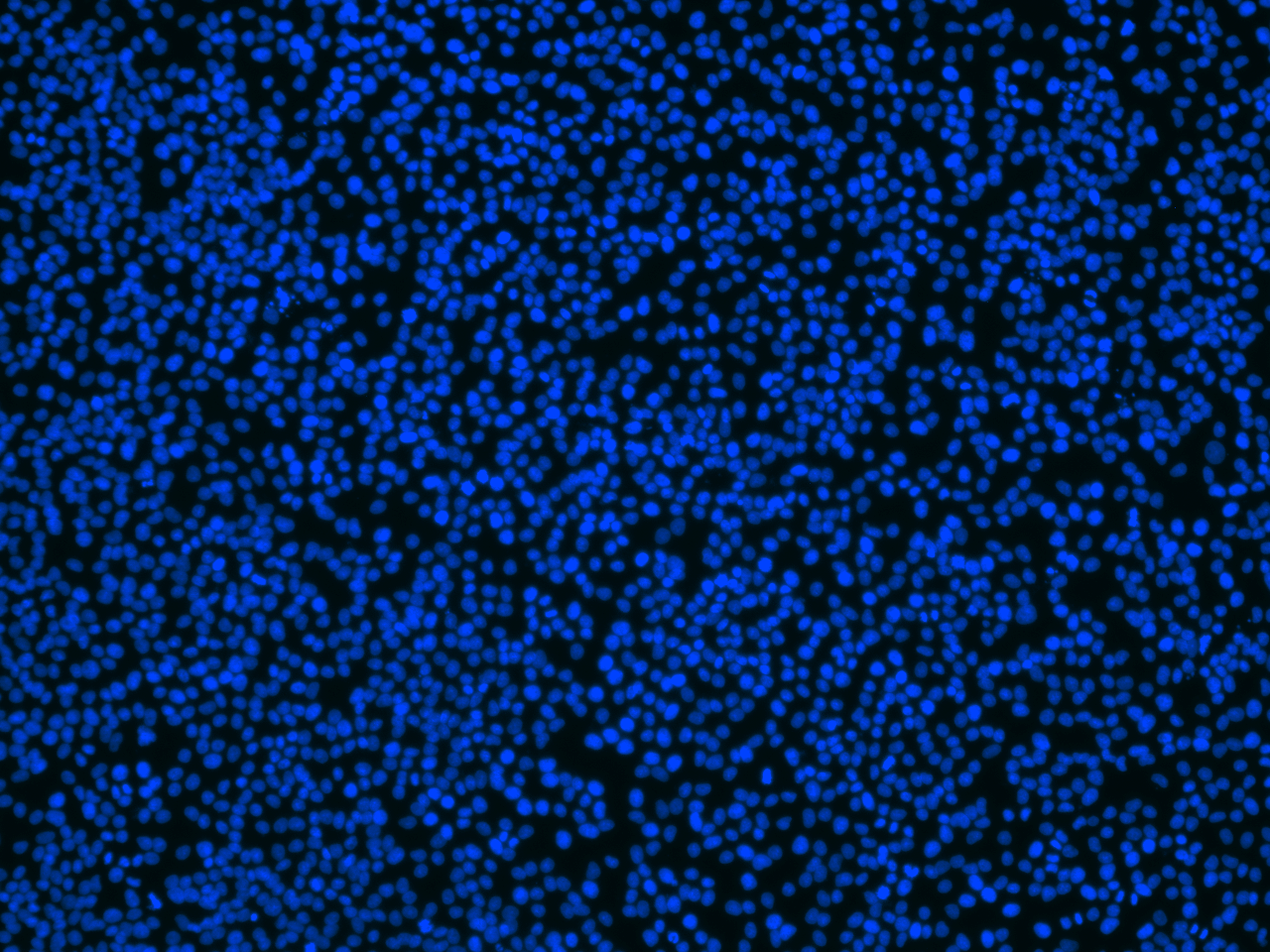

Supplement: S2 Data — This compressed folder contains the underlying numerical data and/or uncropped images used to generate the panels in Fig 2. (ZIP) [file pbio.3003736.s016.zip › S2 Data/Figure 2/F/ko+anpep/tgev-0.01moi-24h-1-14-1_DAPI.png]

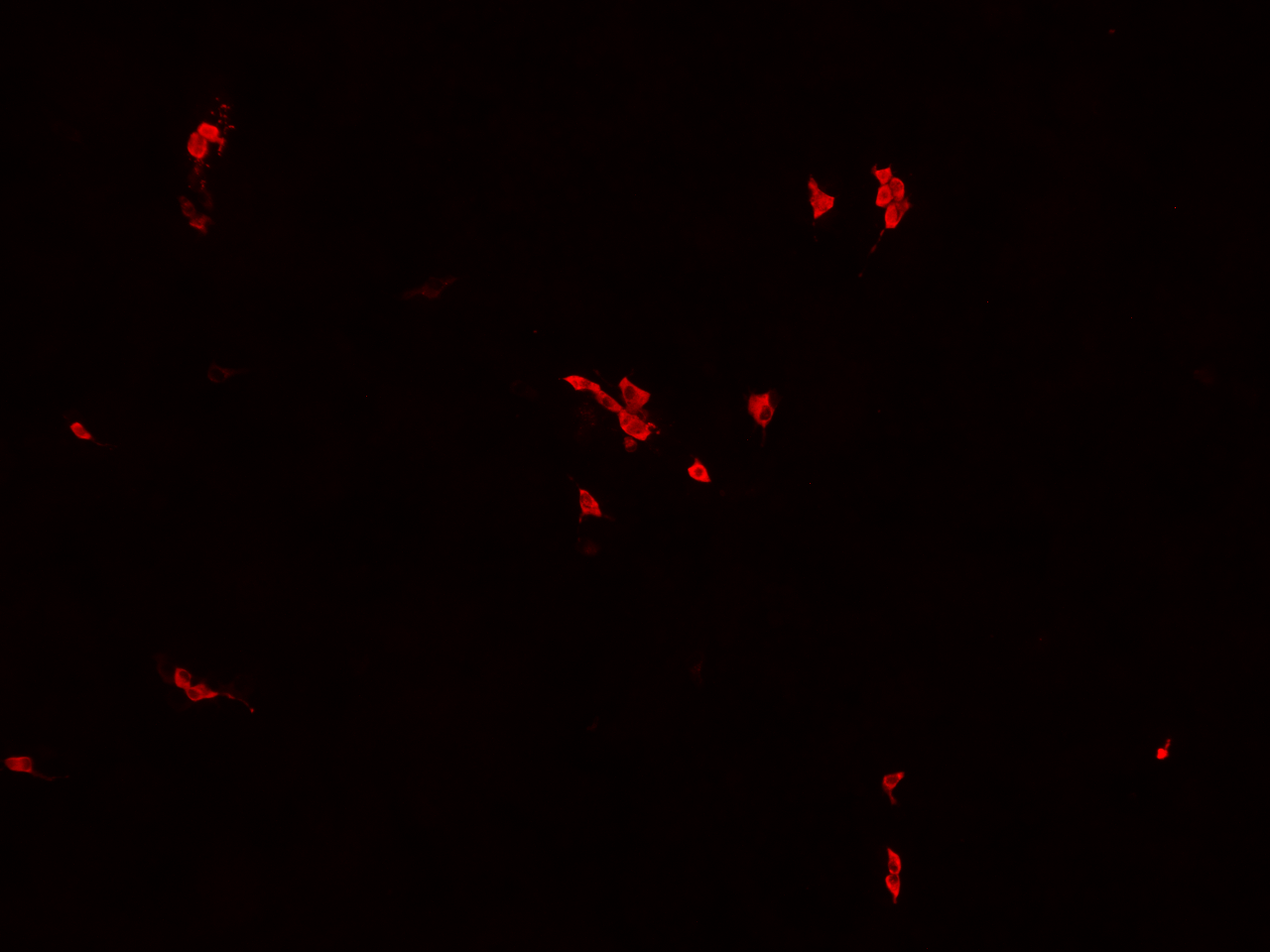

Supplement: S2 Data — This compressed folder contains the underlying numerical data and/or uncropped images used to generate the panels in Fig 2. (ZIP) [file pbio.3003736.s016.zip › S2 Data/Figure 2/F/ko+anpep/tgev-0.01moi-24h-1-14-1_TxRed.png]

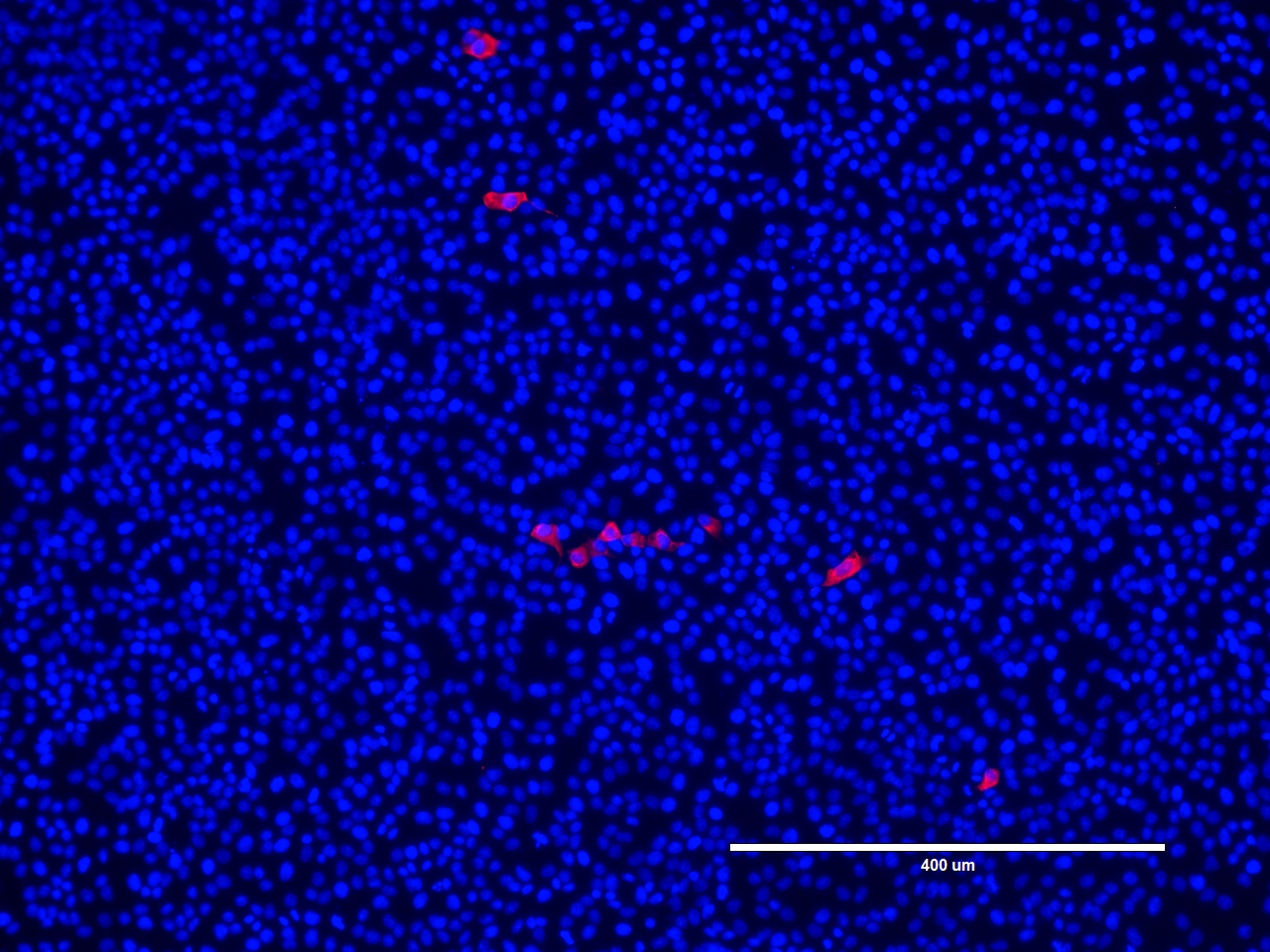

Supplement: S2 Data — This compressed folder contains the underlying numerical data and/or uncropped images used to generate the panels in Fig 2. (ZIP) [file pbio.3003736.s016.zip › S2 Data/Figure 2/F/ko+anpep/tgev-0.01moi-24h-1-14-2.tif]

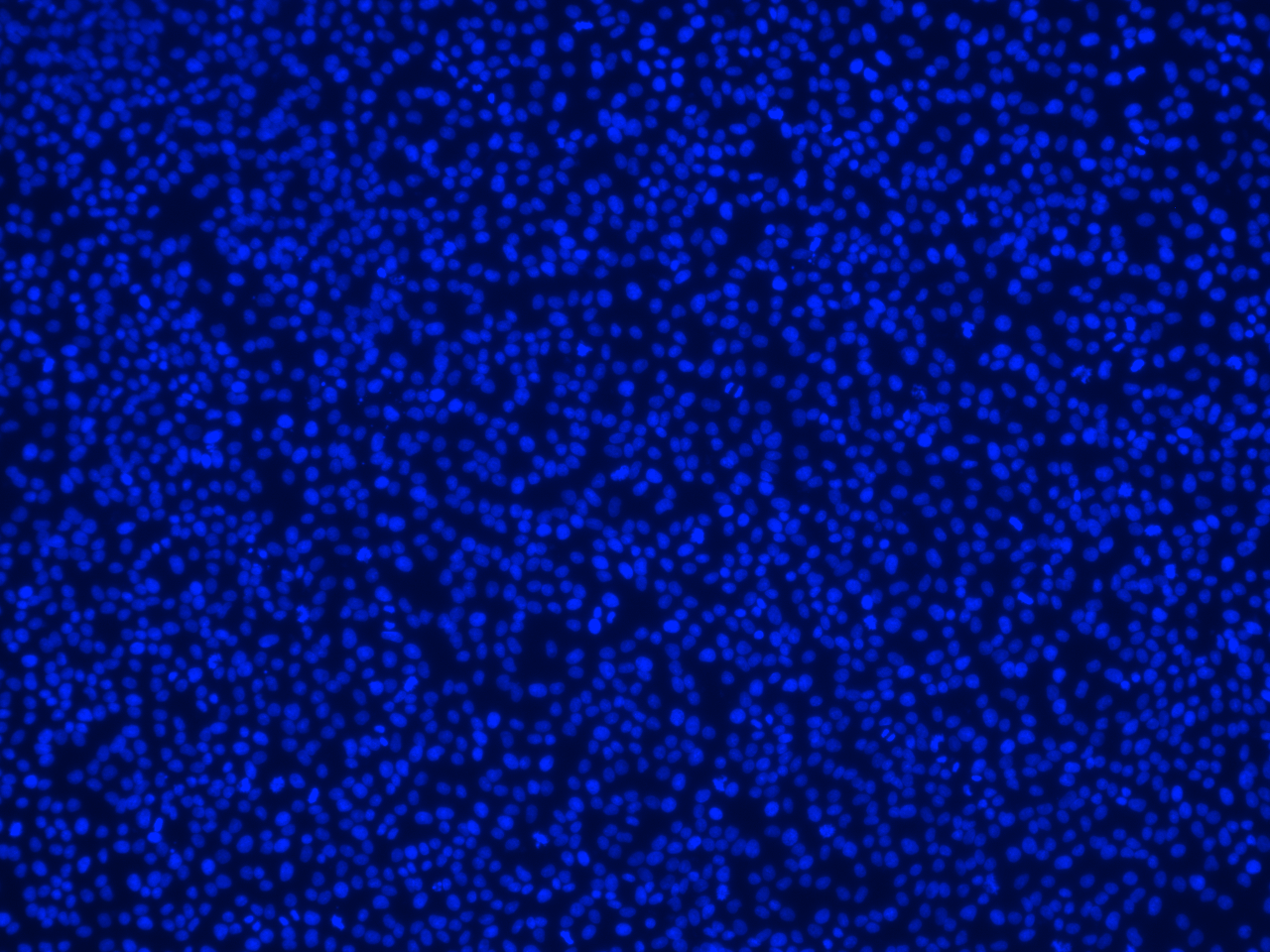

Supplement: S2 Data — This compressed folder contains the underlying numerical data and/or uncropped images used to generate the panels in Fig 2. (ZIP) [file pbio.3003736.s016.zip › S2 Data/Figure 2/F/ko+anpep/tgev-0.01moi-24h-1-14-2_DAPI.tif]

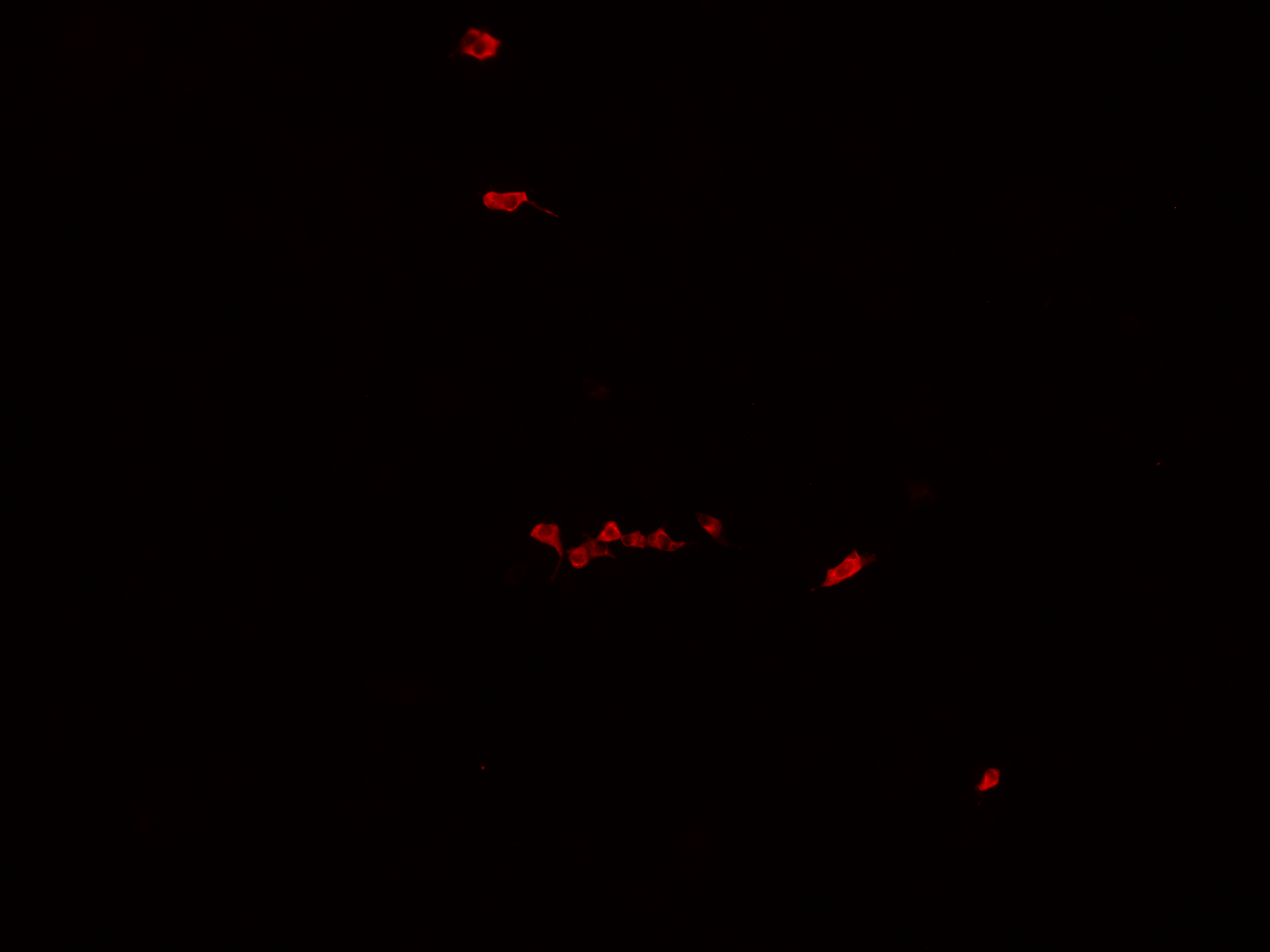

Supplement: S2 Data — This compressed folder contains the underlying numerical data and/or uncropped images used to generate the panels in Fig 2. (ZIP) [file pbio.3003736.s016.zip › S2 Data/Figure 2/F/ko+anpep/tgev-0.01moi-24h-1-14-2_TxRed.tif]

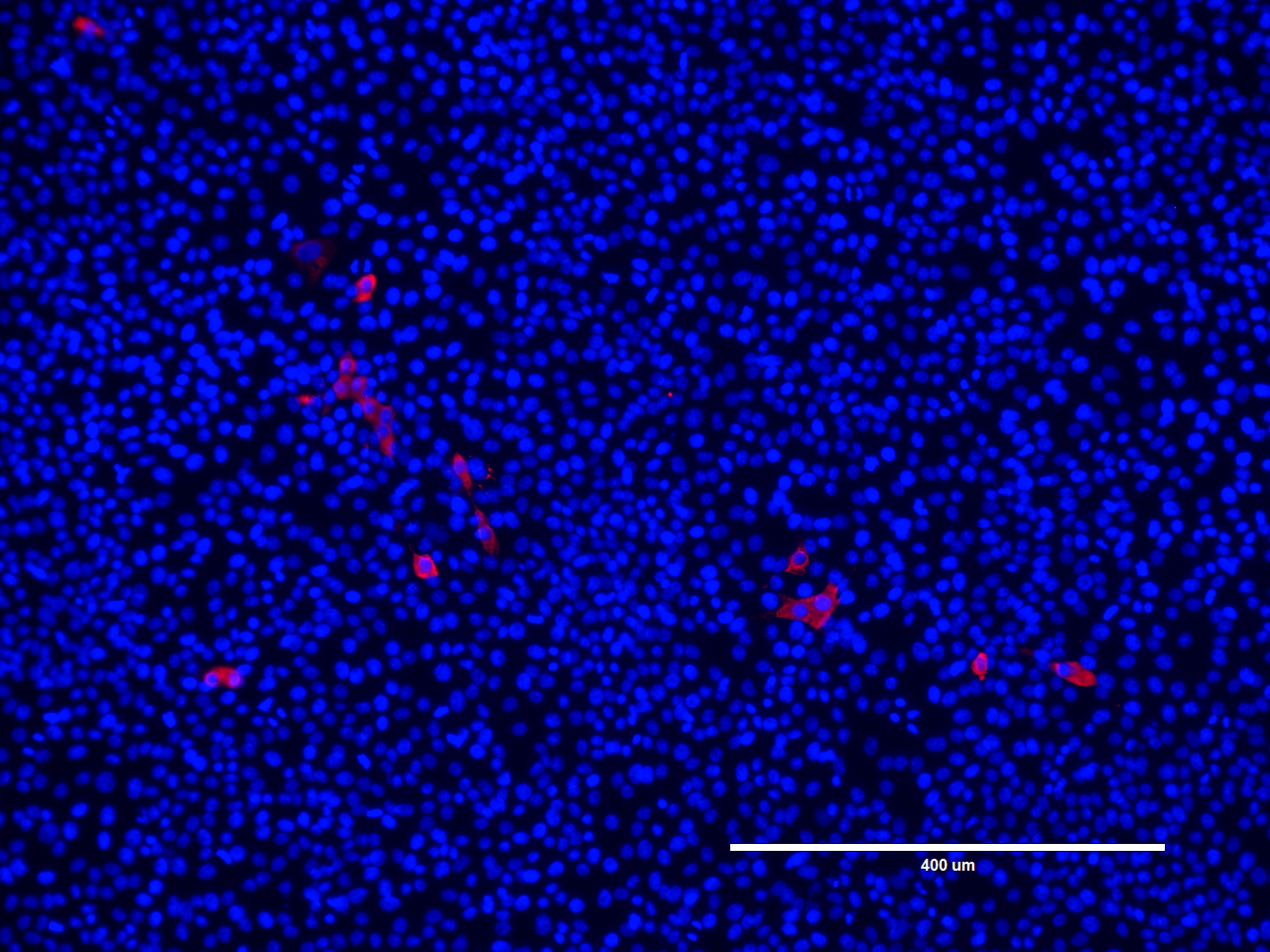

Supplement: S2 Data — This compressed folder contains the underlying numerical data and/or uncropped images used to generate the panels in Fig 2. (ZIP) [file pbio.3003736.s016.zip › S2 Data/Figure 2/F/ko+anpep/tgev-0.01moi-24h-2-16-2.tif]

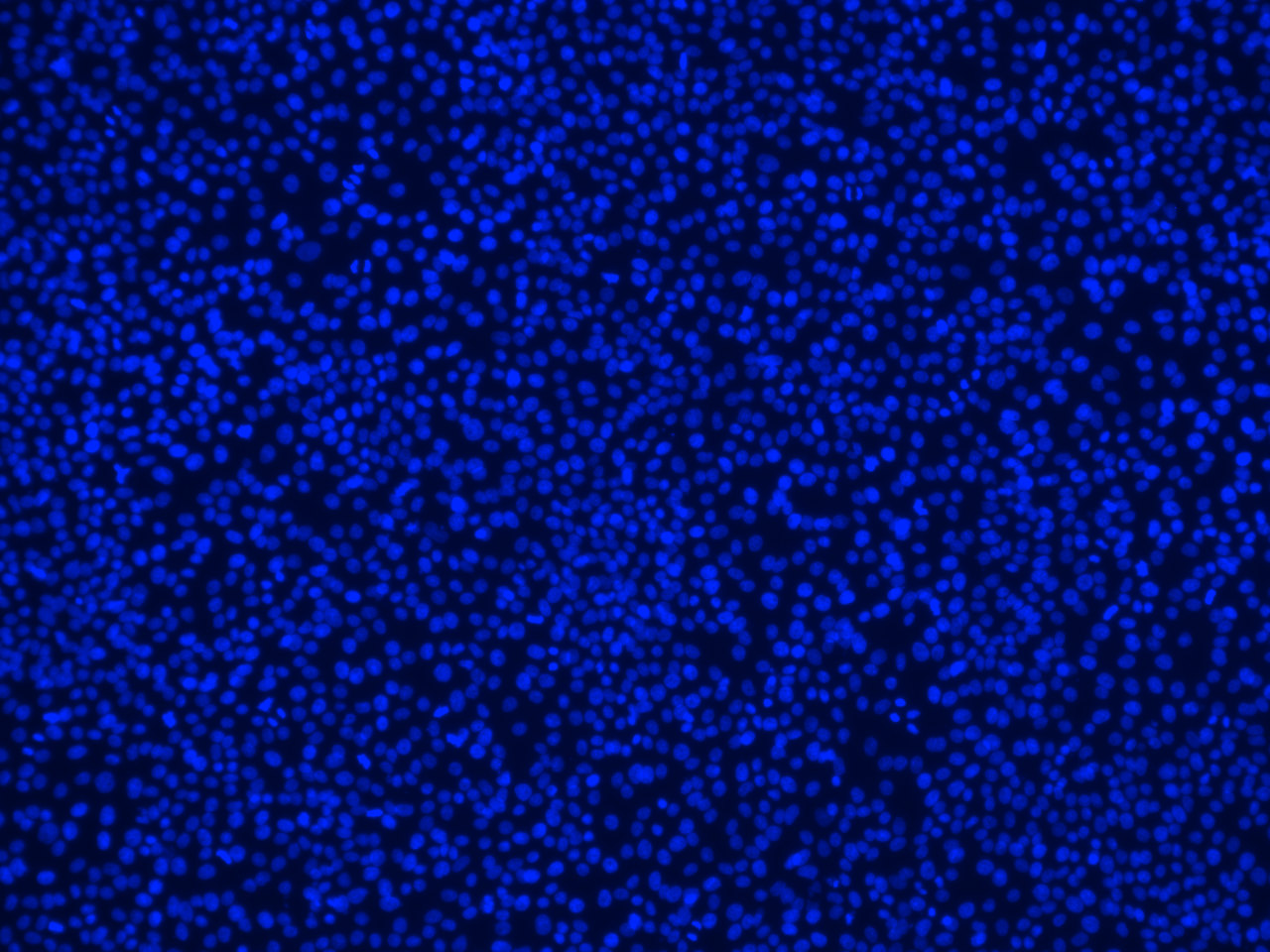

Supplement: S2 Data — This compressed folder contains the underlying numerical data and/or uncropped images used to generate the panels in Fig 2. (ZIP) [file pbio.3003736.s016.zip › S2 Data/Figure 2/F/ko+anpep/tgev-0.01moi-24h-2-16-2_DAPI.tif]

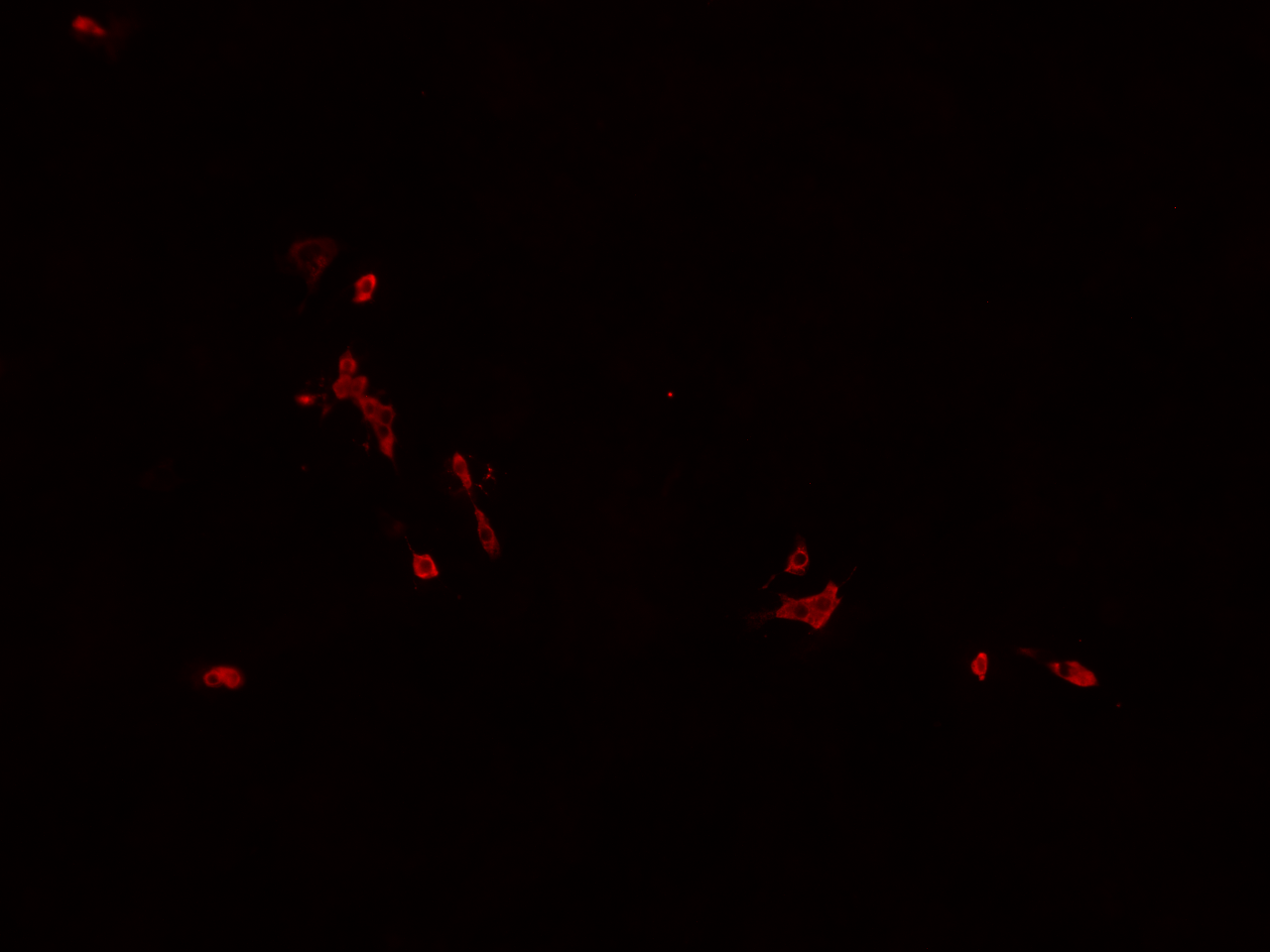

Supplement: S2 Data — This compressed folder contains the underlying numerical data and/or uncropped images used to generate the panels in Fig 2. (ZIP) [file pbio.3003736.s016.zip › S2 Data/Figure 2/F/ko+anpep/tgev-0.01moi-24h-2-16-2_TxRed.tif]

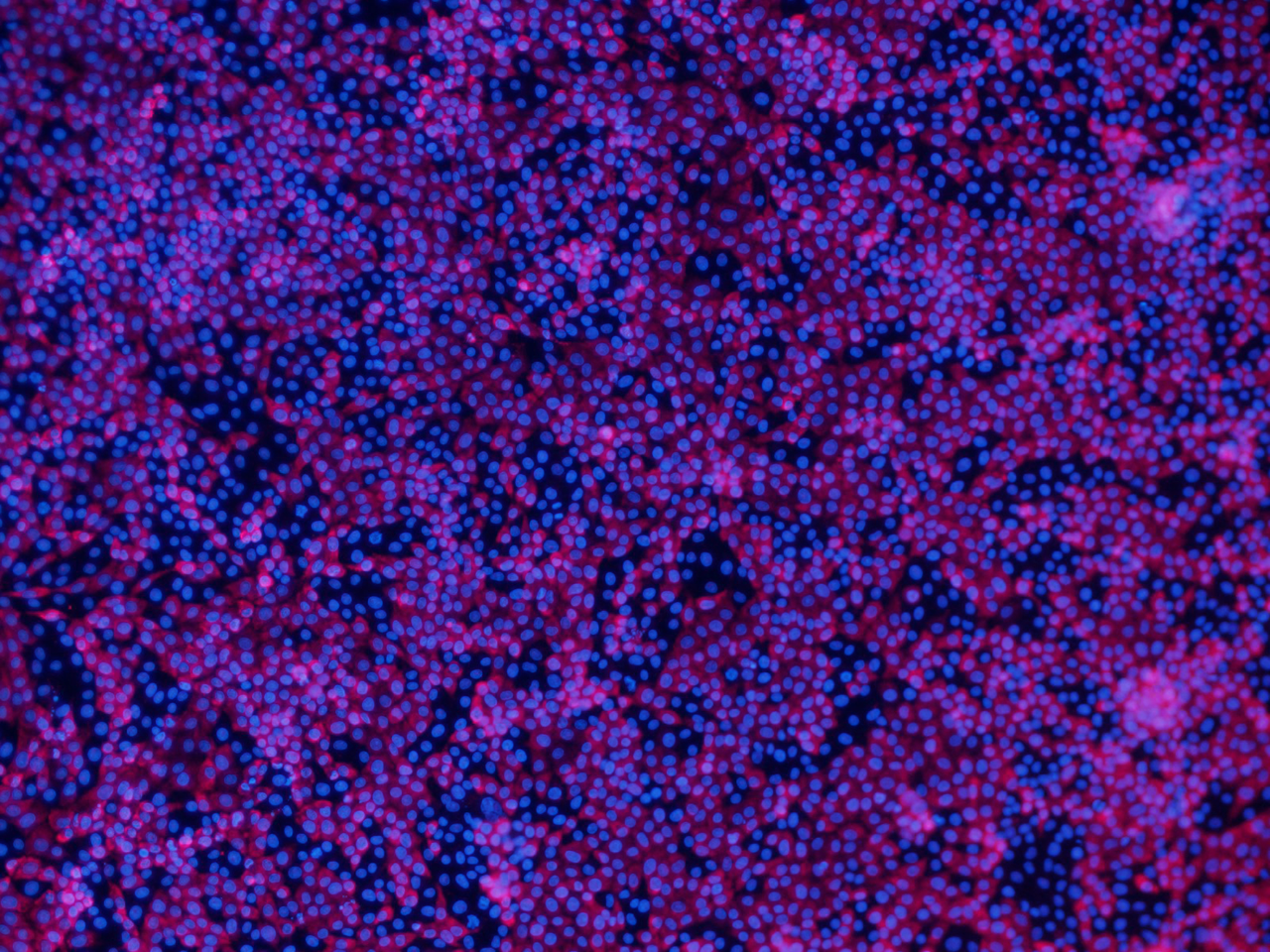

Supplement: S2 Data — This compressed folder contains the underlying numerical data and/or uncropped images used to generate the panels in Fig 2. (ZIP) [file pbio.3003736.s016.zip › S2 Data/Figure 2/F/PK/pk-1.png]

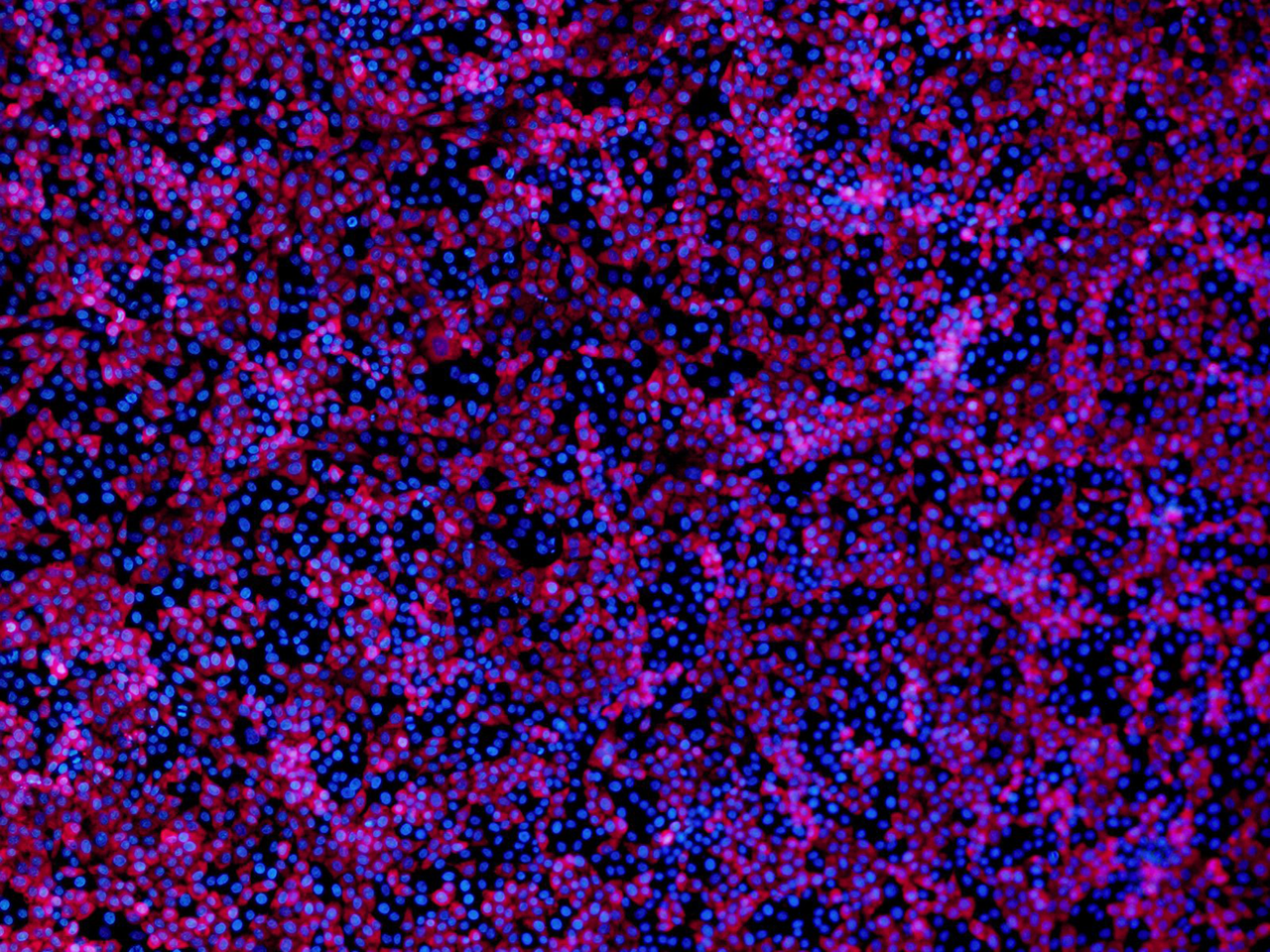

Supplement: S2 Data — This compressed folder contains the underlying numerical data and/or uncropped images used to generate the panels in Fig 2. (ZIP) [file pbio.3003736.s016.zip › S2 Data/Figure 2/F/PK/PK-2.png]

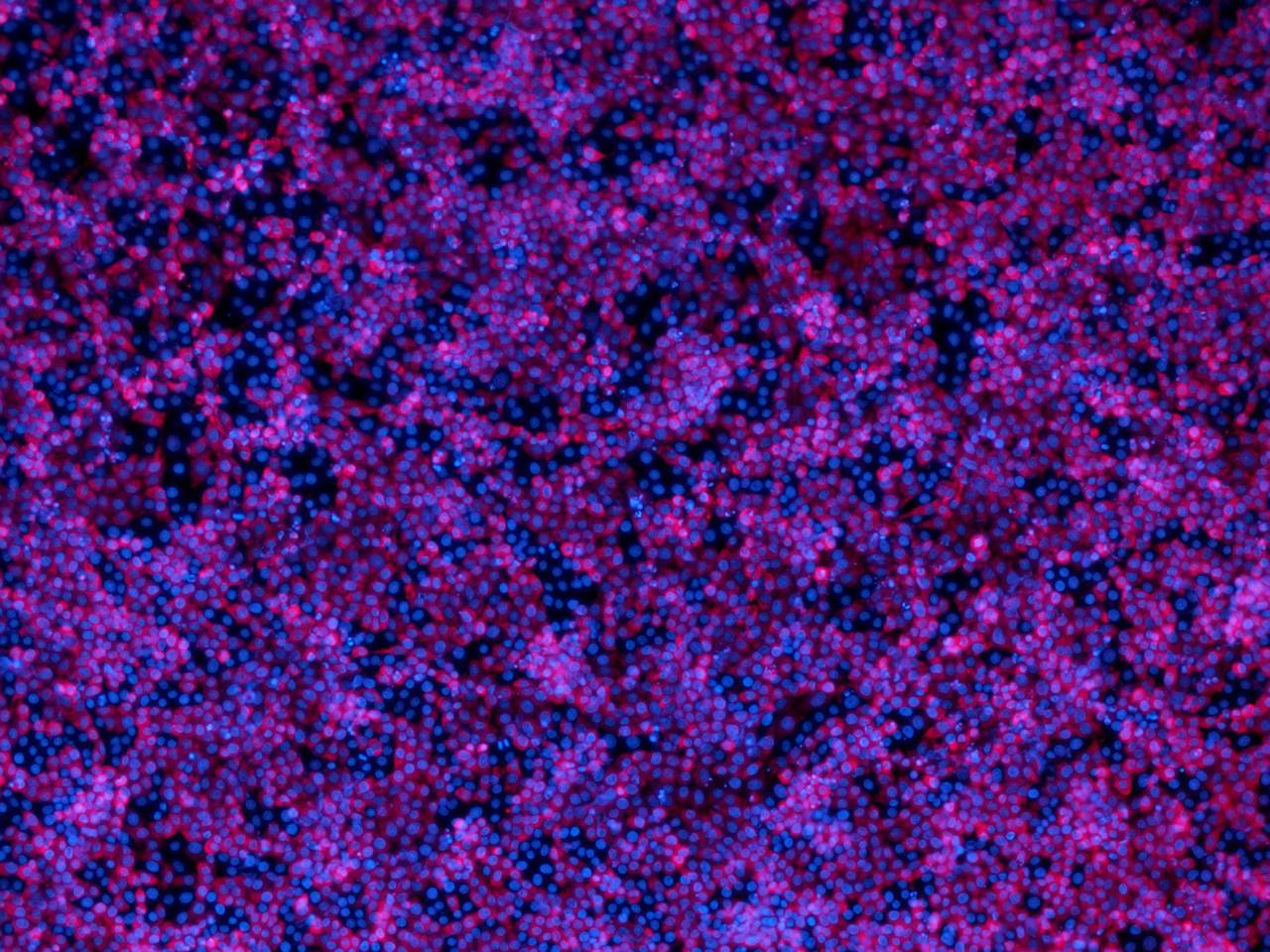

Supplement: S2 Data — This compressed folder contains the underlying numerical data and/or uncropped images used to generate the panels in Fig 2. (ZIP) [file pbio.3003736.s016.zip › S2 Data/Figure 2/F/PK/PK-3.png]

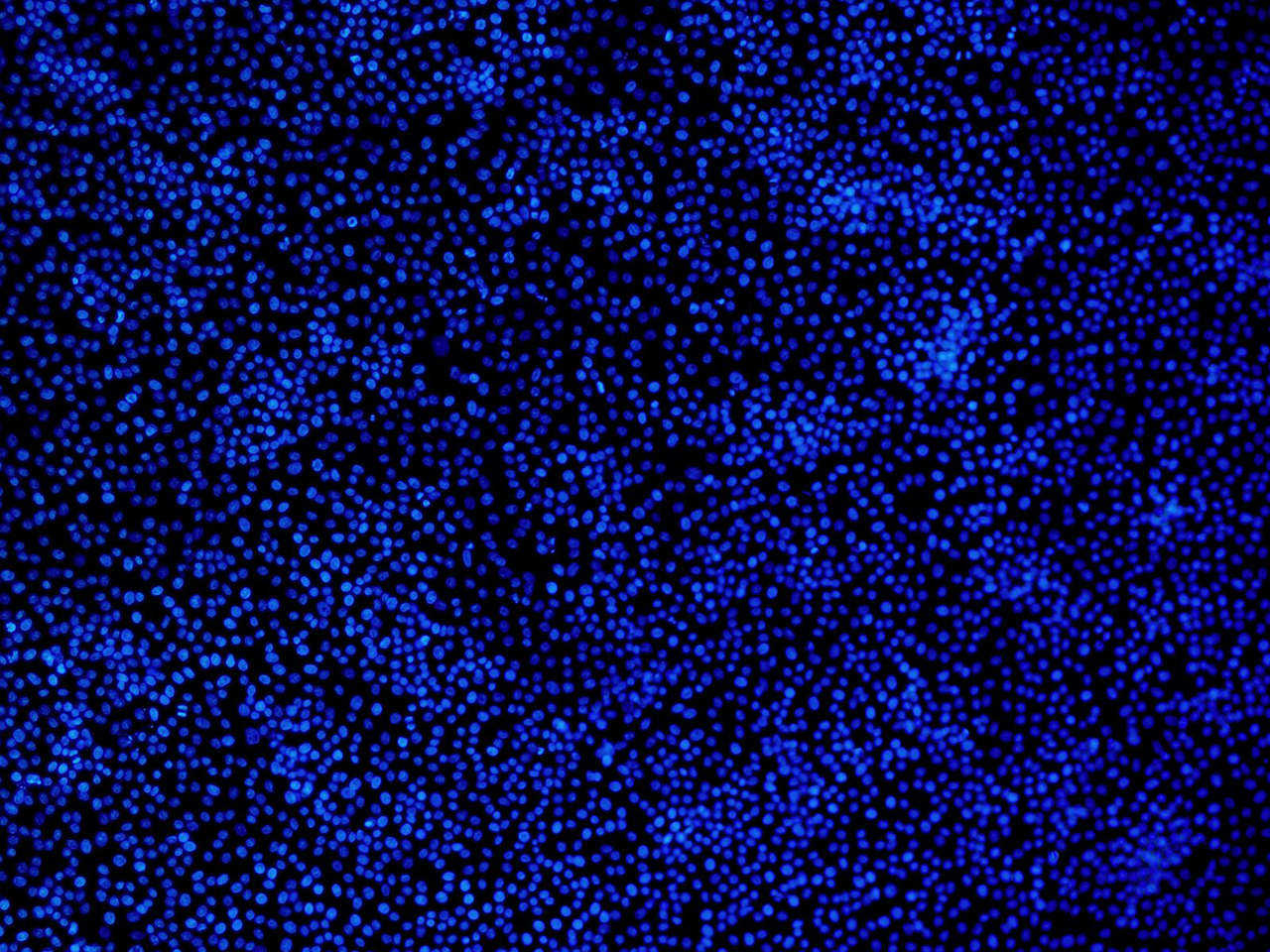

Supplement: S2 Data — This compressed folder contains the underlying numerical data and/or uncropped images used to generate the panels in Fig 2. (ZIP) [file pbio.3003736.s016.zip › S2 Data/Figure 2/F/PK/PK-DAPI-2.png]

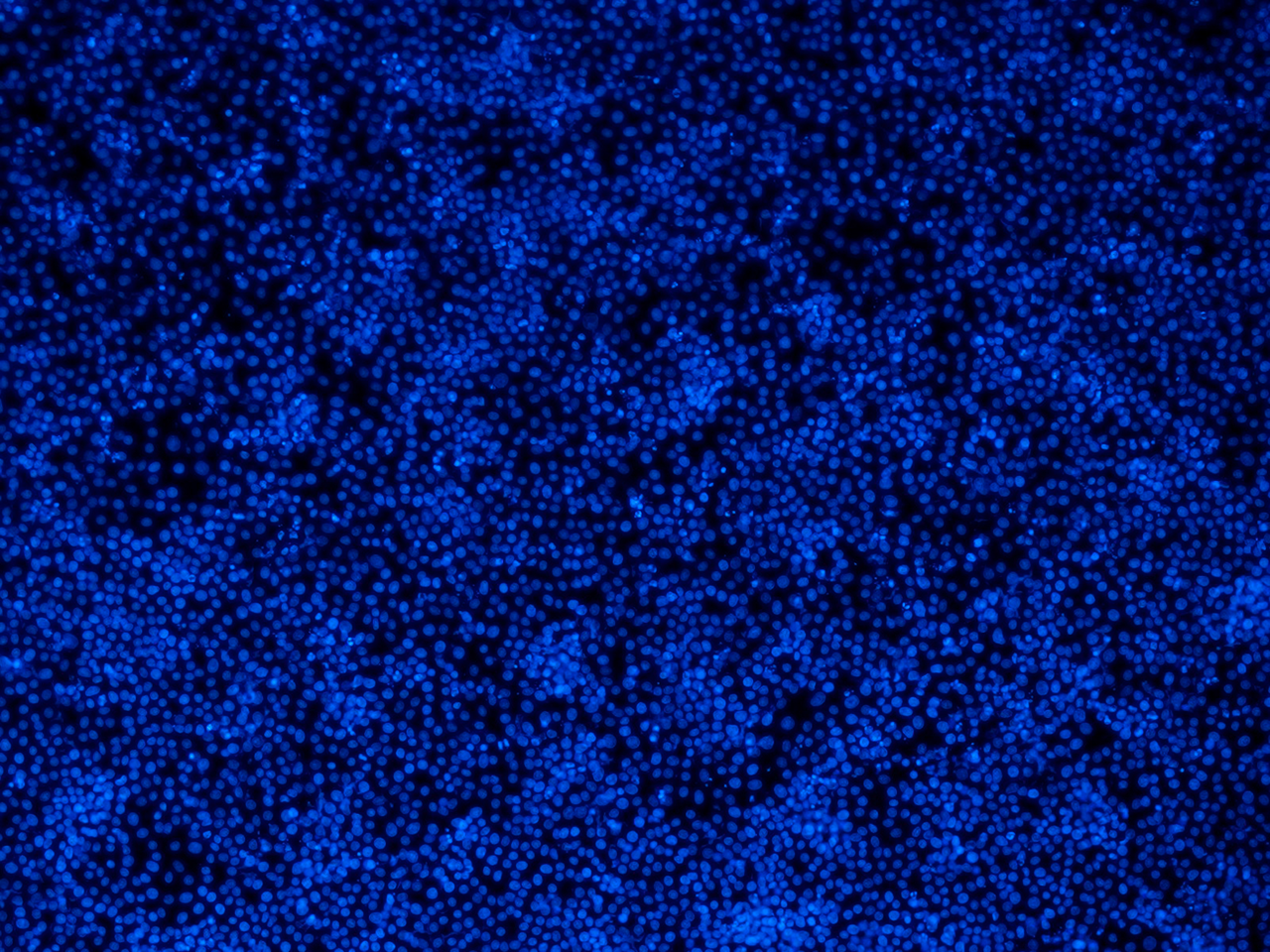

Supplement: S2 Data — This compressed folder contains the underlying numerical data and/or uncropped images used to generate the panels in Fig 2. (ZIP) [file pbio.3003736.s016.zip › S2 Data/Figure 2/F/PK/PK-DAPI-3.png]

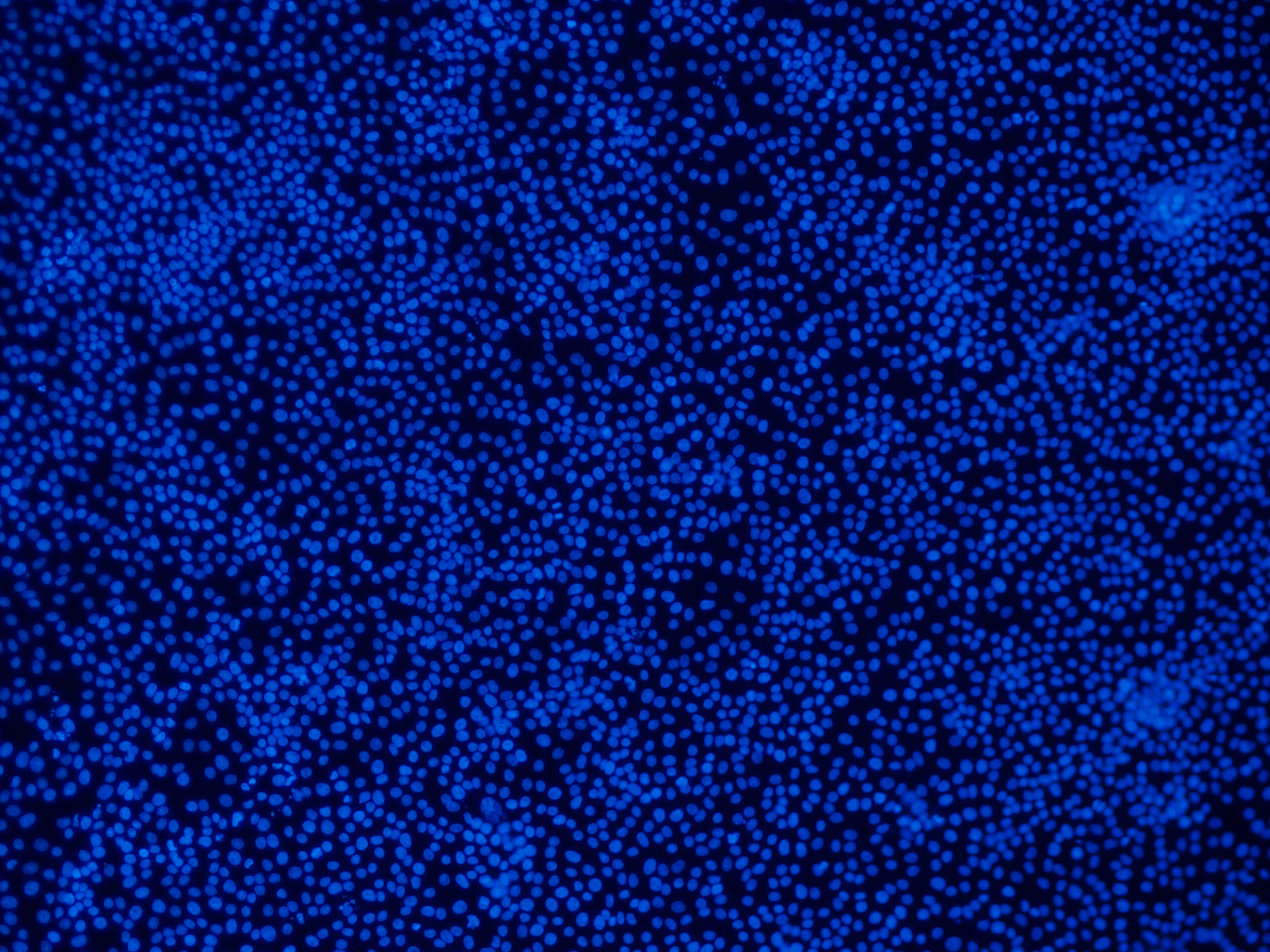

Supplement: S2 Data — This compressed folder contains the underlying numerical data and/or uncropped images used to generate the panels in Fig 2. (ZIP) [file pbio.3003736.s016.zip › S2 Data/Figure 2/F/PK/pk-dAPI.png]

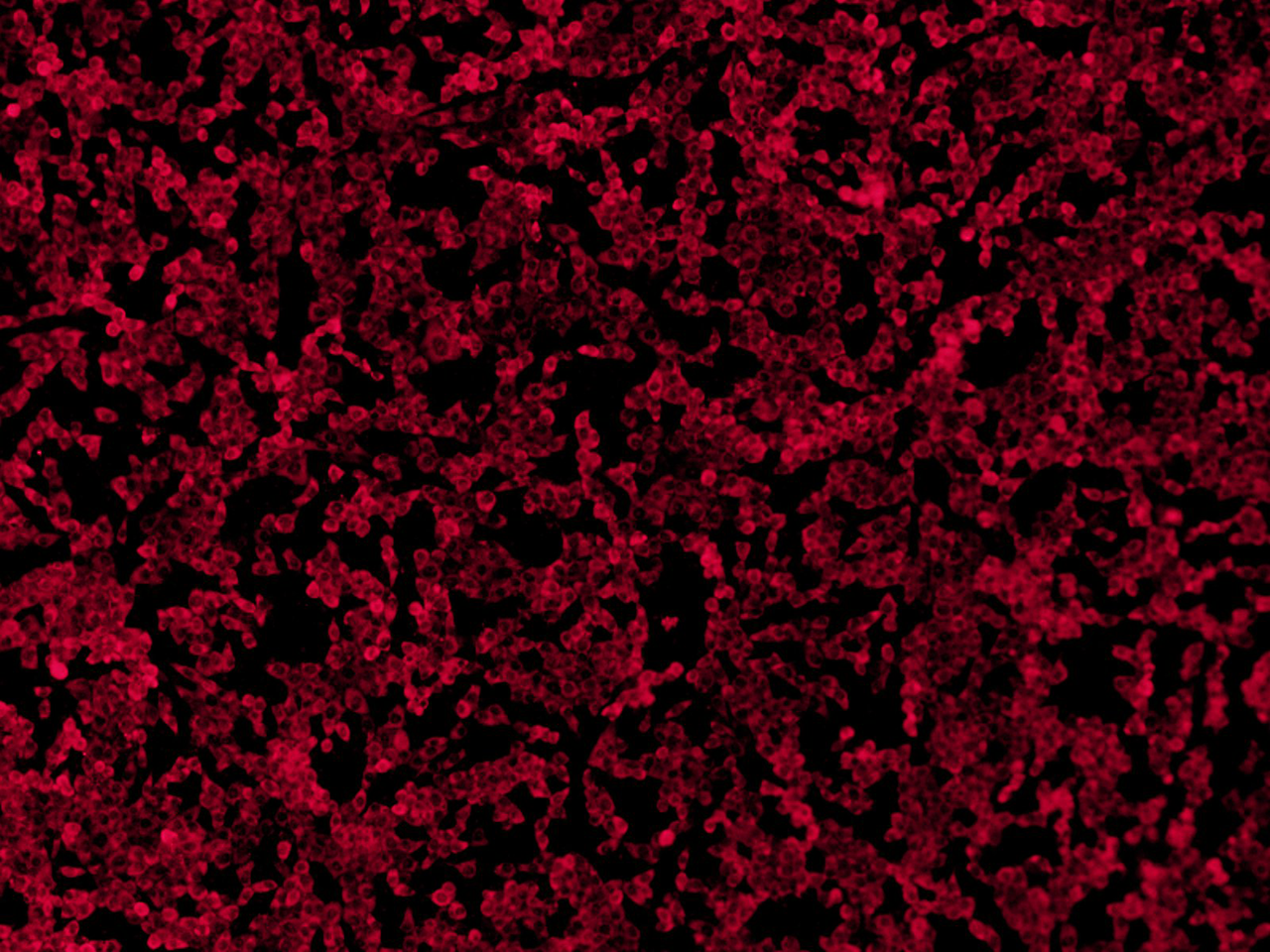

Supplement: S2 Data — This compressed folder contains the underlying numerical data and/or uncropped images used to generate the panels in Fig 2. (ZIP) [file pbio.3003736.s016.zip › S2 Data/Figure 2/F/PK/PK-TGEV-2.png]

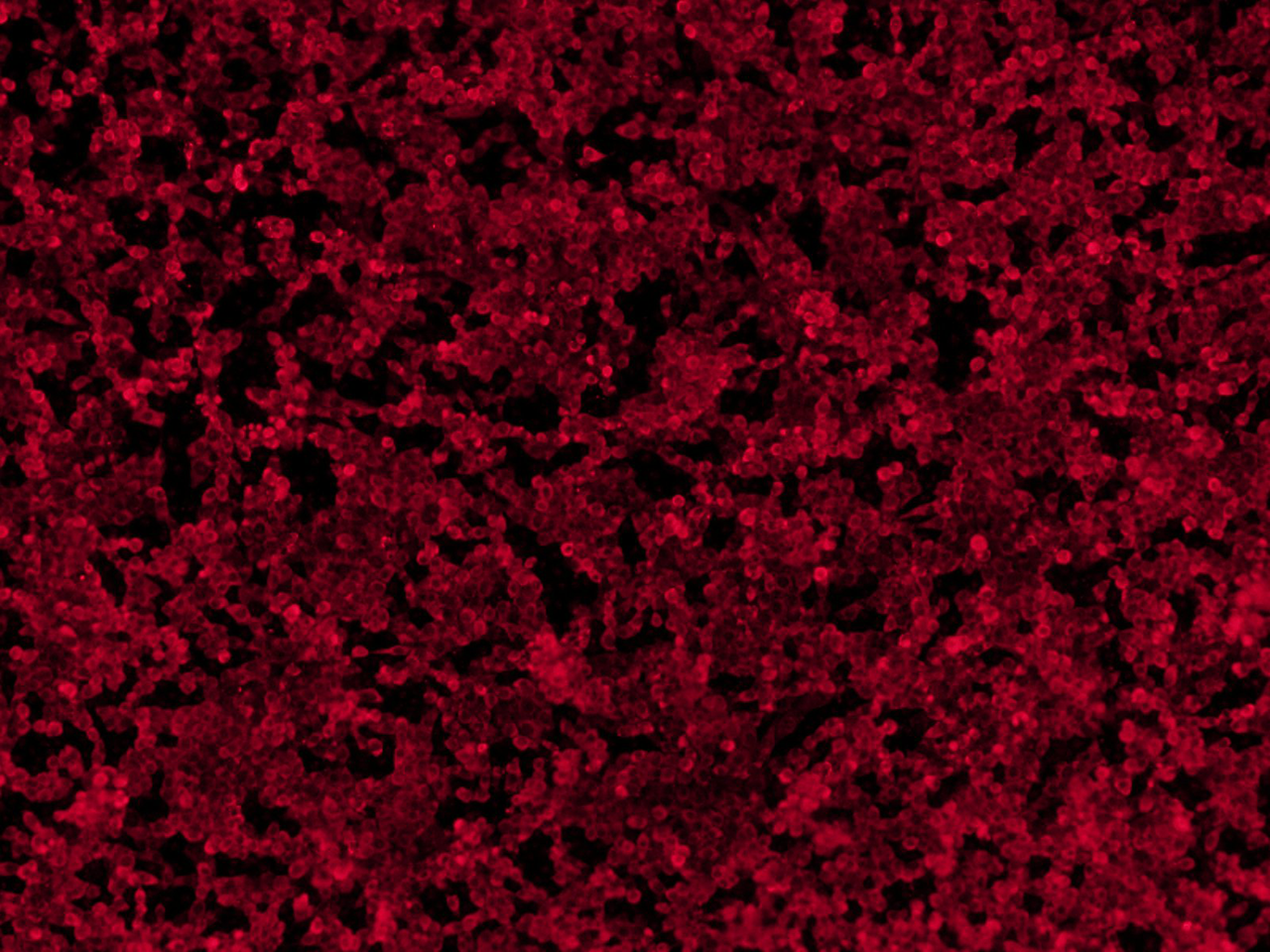

Supplement: S2 Data — This compressed folder contains the underlying numerical data and/or uncropped images used to generate the panels in Fig 2. (ZIP) [file pbio.3003736.s016.zip › S2 Data/Figure 2/F/PK/PK-TGEV-N-3.png]

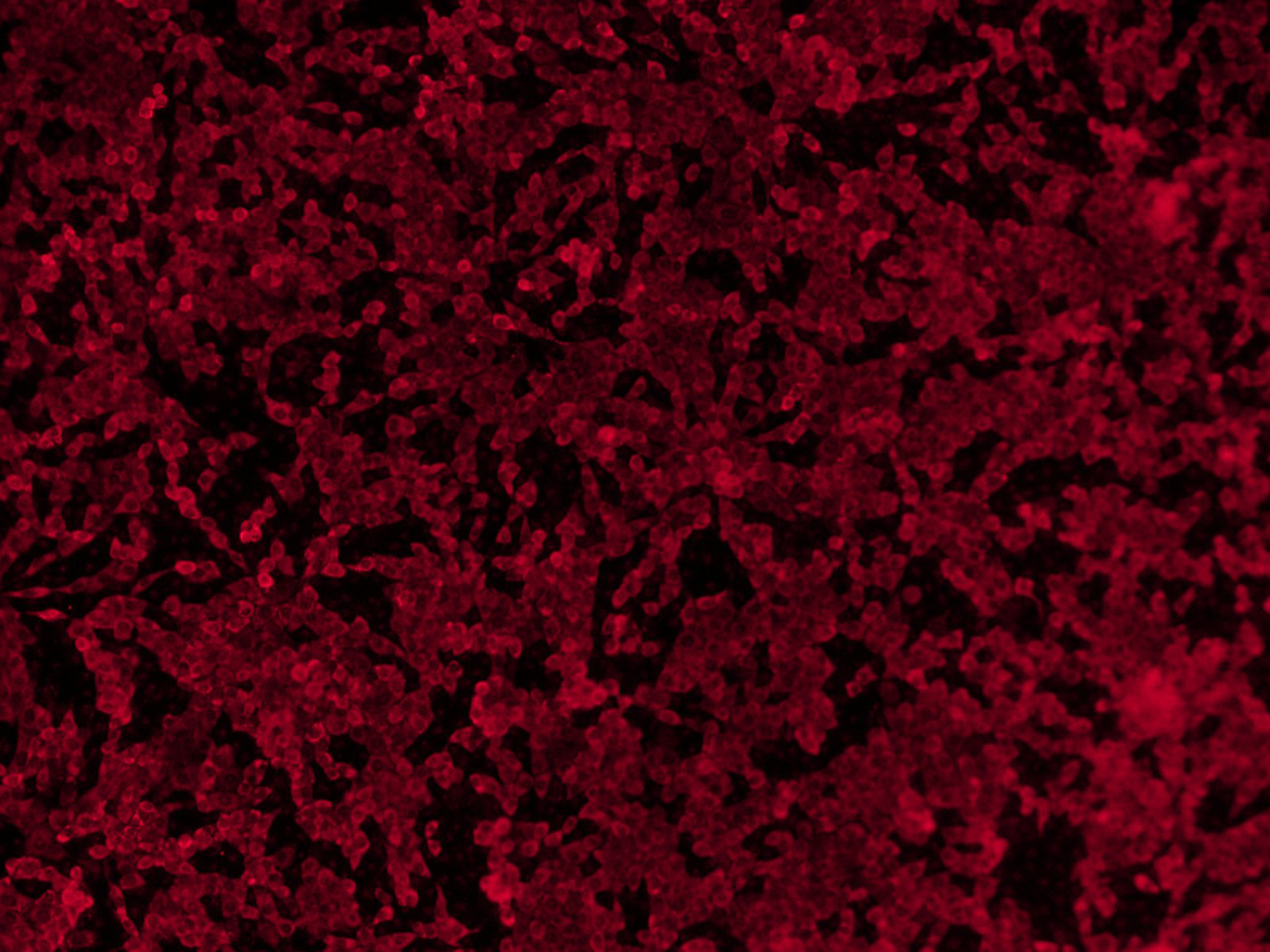

Supplement: S2 Data — This compressed folder contains the underlying numerical data and/or uncropped images used to generate the panels in Fig 2. (ZIP) [file pbio.3003736.s016.zip › S2 Data/Figure 2/F/PK/PK-TGEV.png]

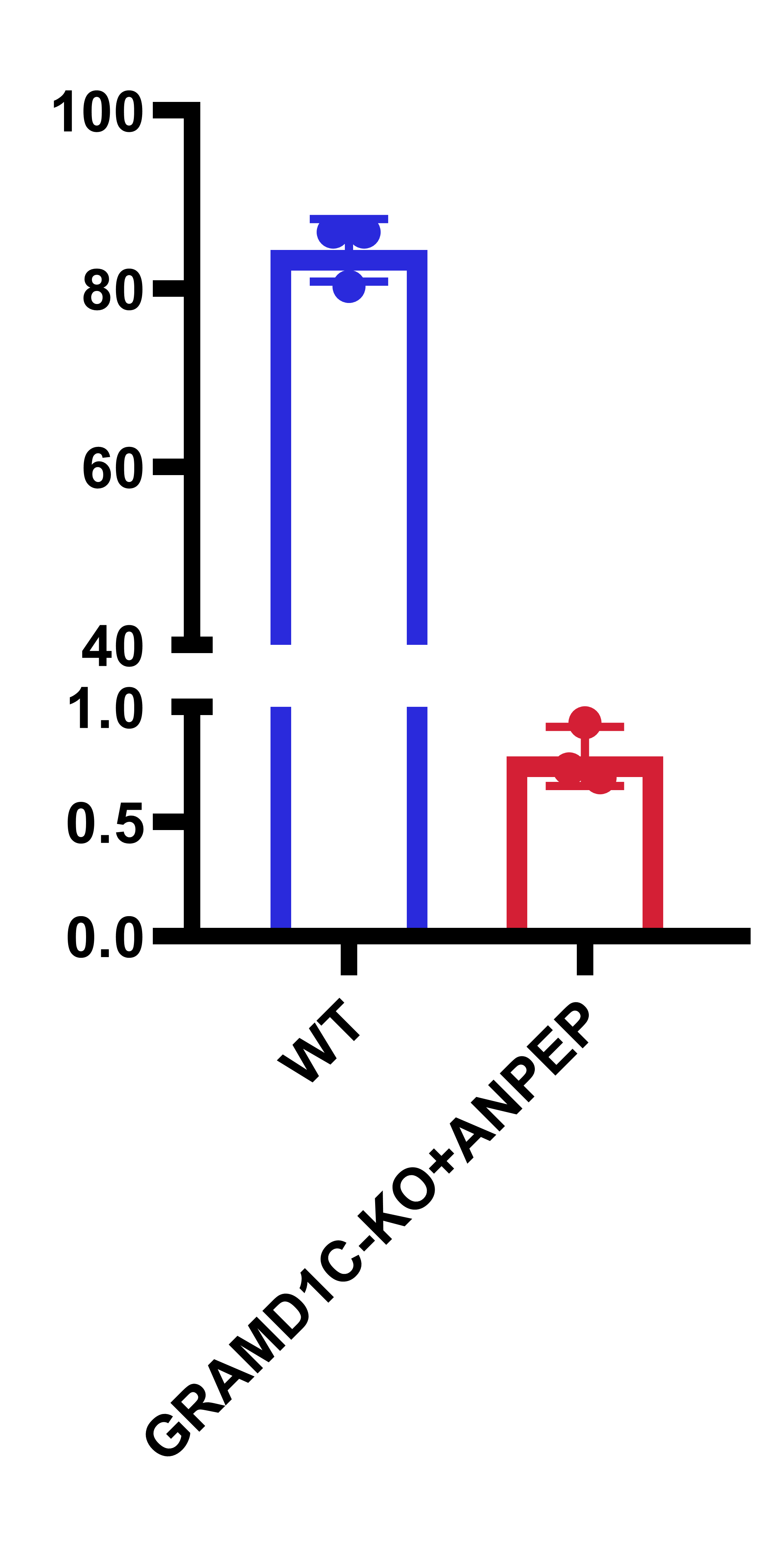

Supplement: S2 Data — This compressed folder contains the underlying numerical data and/or uncropped images used to generate the panels in Fig 2. (ZIP) [file pbio.3003736.s016.zip › S2 Data/Figure 2/F/positive-cells.tif]

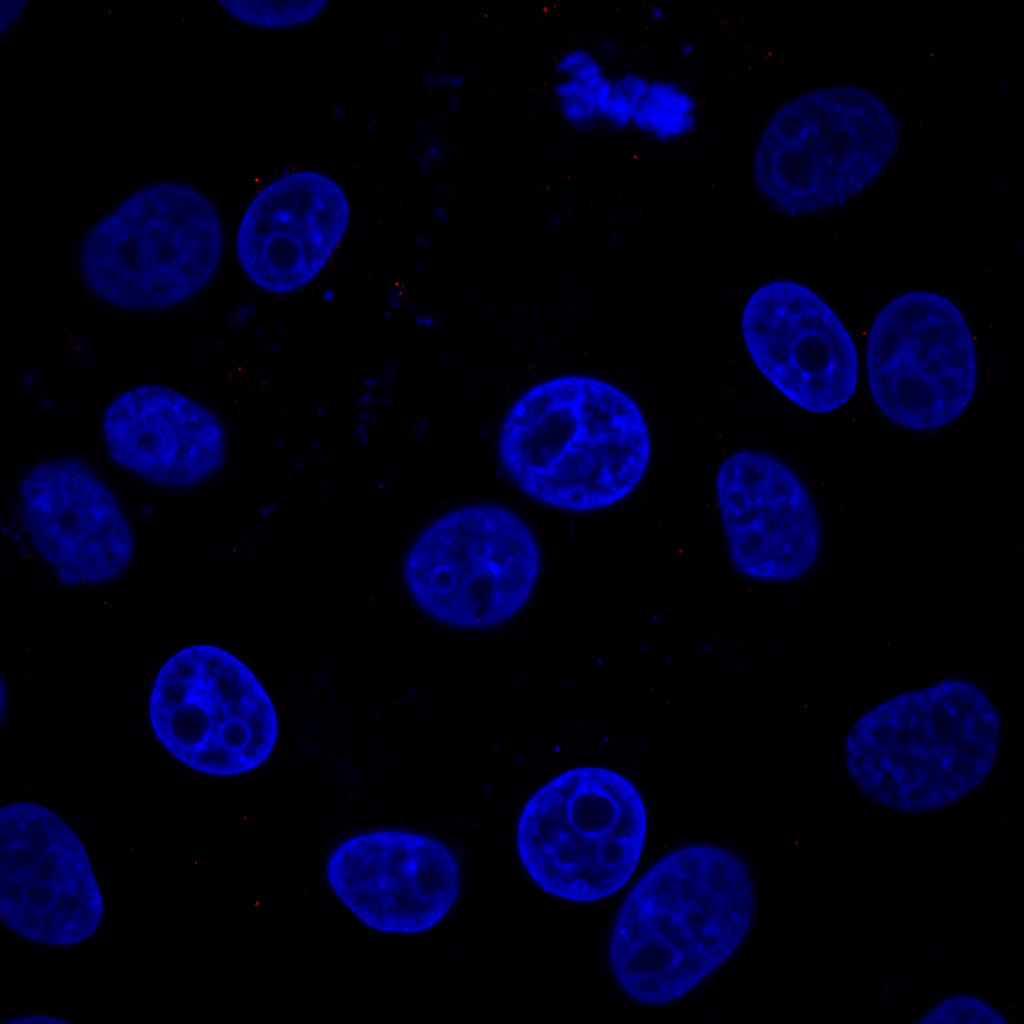

Supplement: S2 Data — This compressed folder contains the underlying numerical data and/or uncropped images used to generate the panels in Fig 2. (ZIP) [file pbio.3003736.s016.zip › S2 Data/Figure 2/G/KO+ANPEP/2-4-APN-1_RGB.tif]

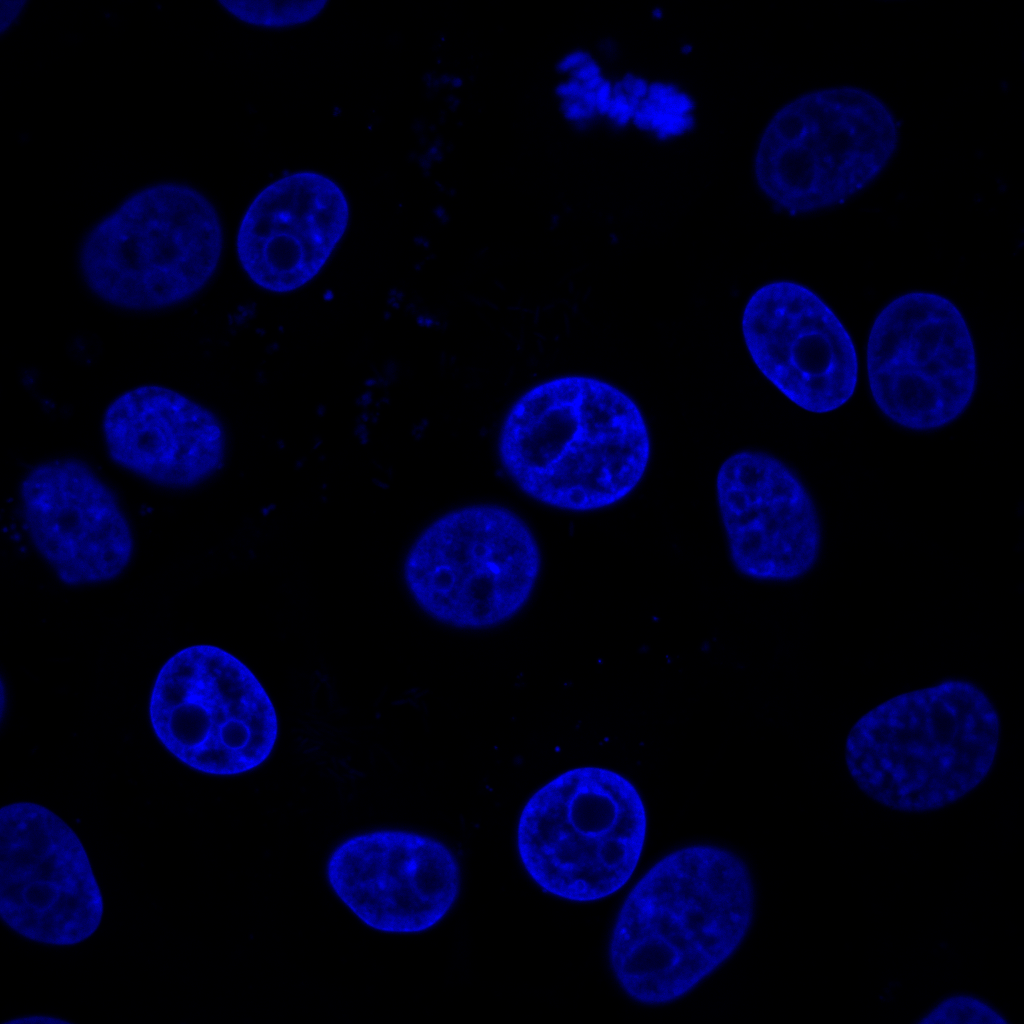

Supplement: S2 Data — This compressed folder contains the underlying numerical data and/or uncropped images used to generate the panels in Fig 2. (ZIP) [file pbio.3003736.s016.zip › S2 Data/Figure 2/G/KO+ANPEP/2-4-APN-1_RGB_DAPI.tif]

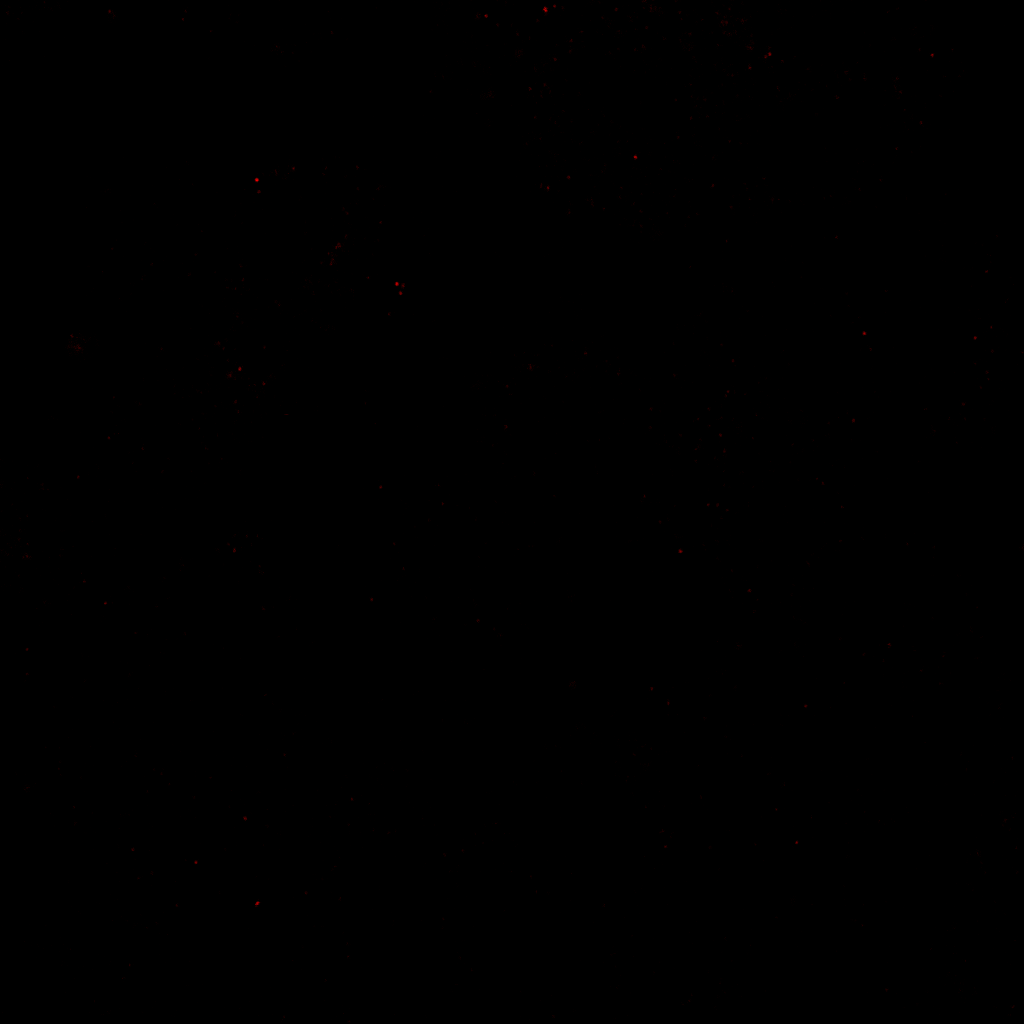

Supplement: S2 Data — This compressed folder contains the underlying numerical data and/or uncropped images used to generate the panels in Fig 2. (ZIP) [file pbio.3003736.s016.zip › S2 Data/Figure 2/G/KO+ANPEP/2-4-APN-1_RGB_TRITC.tif]

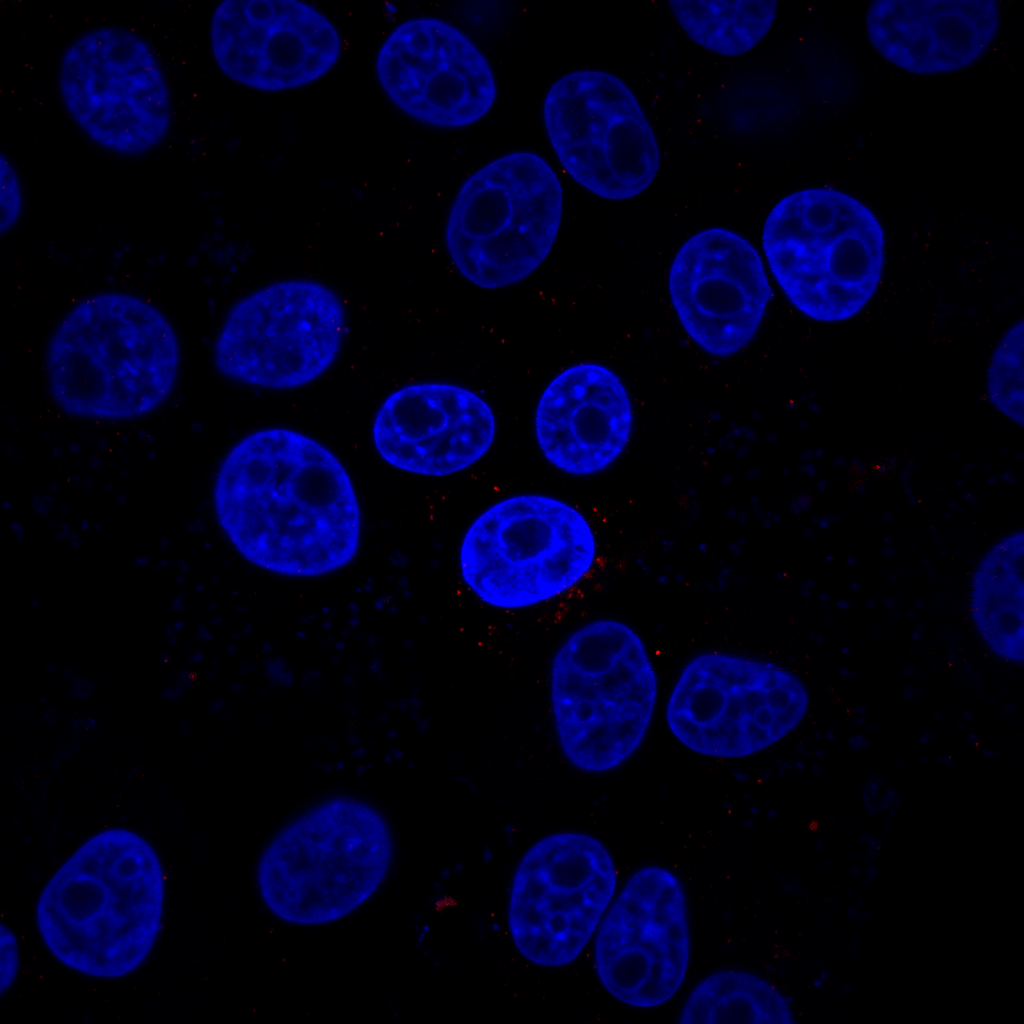

Supplement: S2 Data — This compressed folder contains the underlying numerical data and/or uncropped images used to generate the panels in Fig 2. (ZIP) [file pbio.3003736.s016.zip › S2 Data/Figure 2/G/KO+ANPEP/2-4-APN-3_RGB.tif]
