# Supplementary figures and images for: The non-vesicular cholesterol transporter GRAMD1C is a pan-coronavirus antiviral target (part 2 of 4)
Source: PLoS Biol. 2026 Apr 6;24(4):e3003736. doi: 10.1371/journal.pbio.3003736 (PMC13068348; doi:10.1371/journal.pbio.3003736)

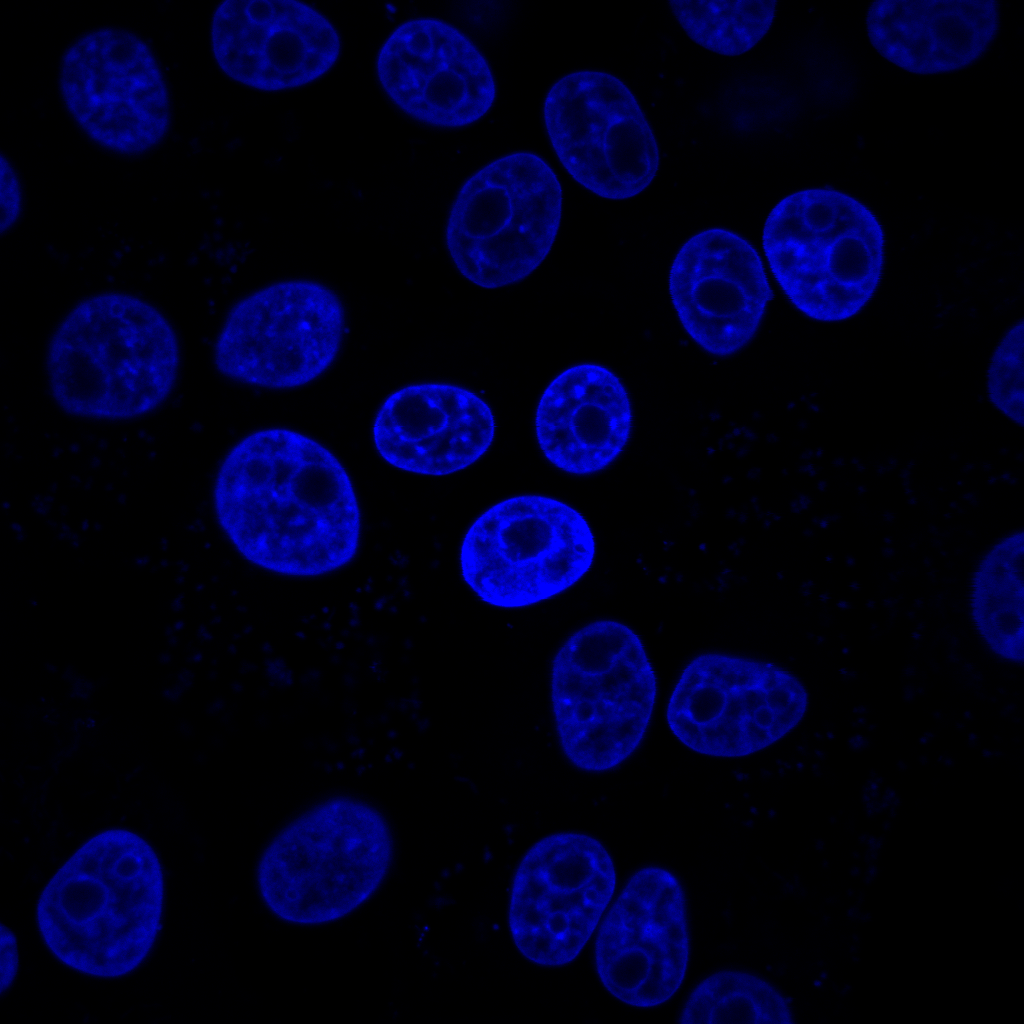

Supplement: S2 Data — This compressed folder contains the underlying numerical data and/or uncropped images used to generate the panels in Fig 2. (ZIP) [file pbio.3003736.s016.zip › S2 Data/Figure 2/G/KO+ANPEP/2-4-APN-3_RGB_DAPI.tif]

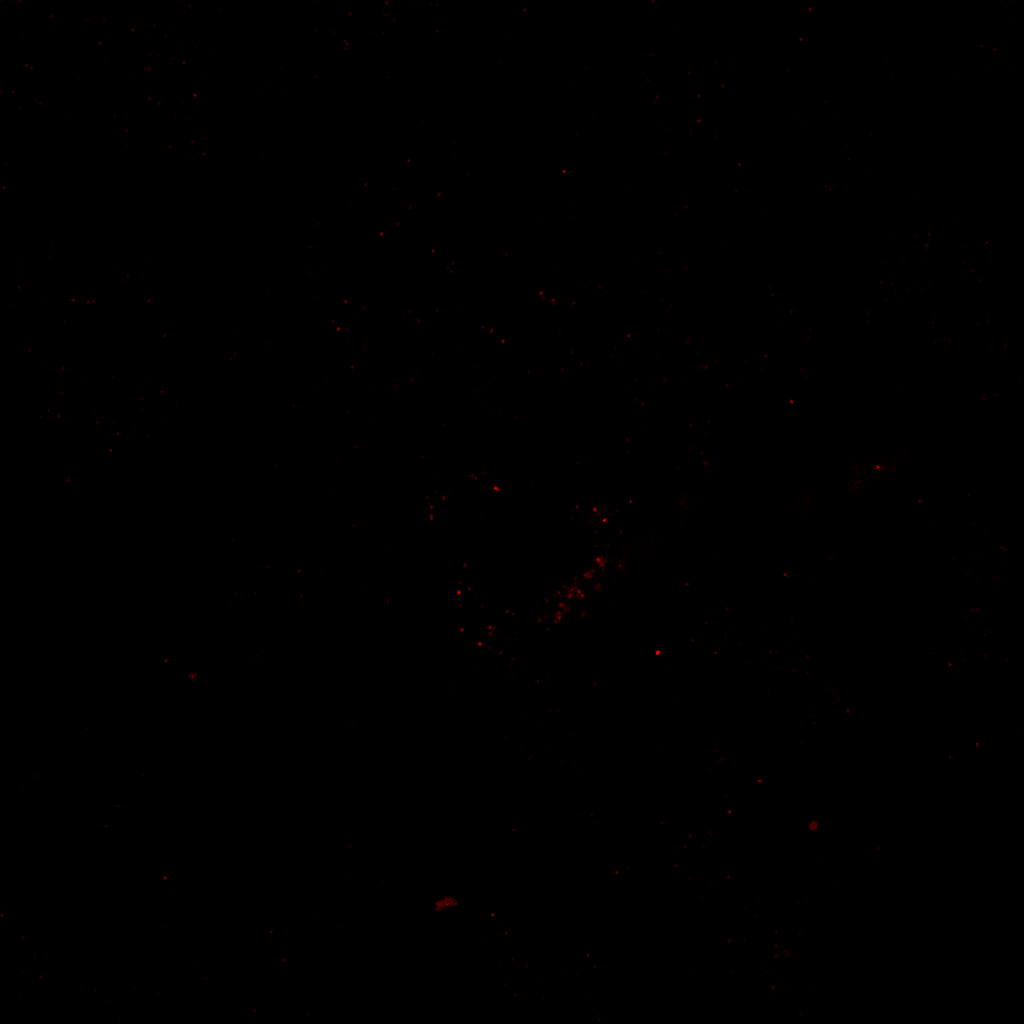

Supplement: S2 Data — This compressed folder contains the underlying numerical data and/or uncropped images used to generate the panels in Fig 2. (ZIP) [file pbio.3003736.s016.zip › S2 Data/Figure 2/G/KO+ANPEP/2-4-APN-3_RGB_TRITC.tif]

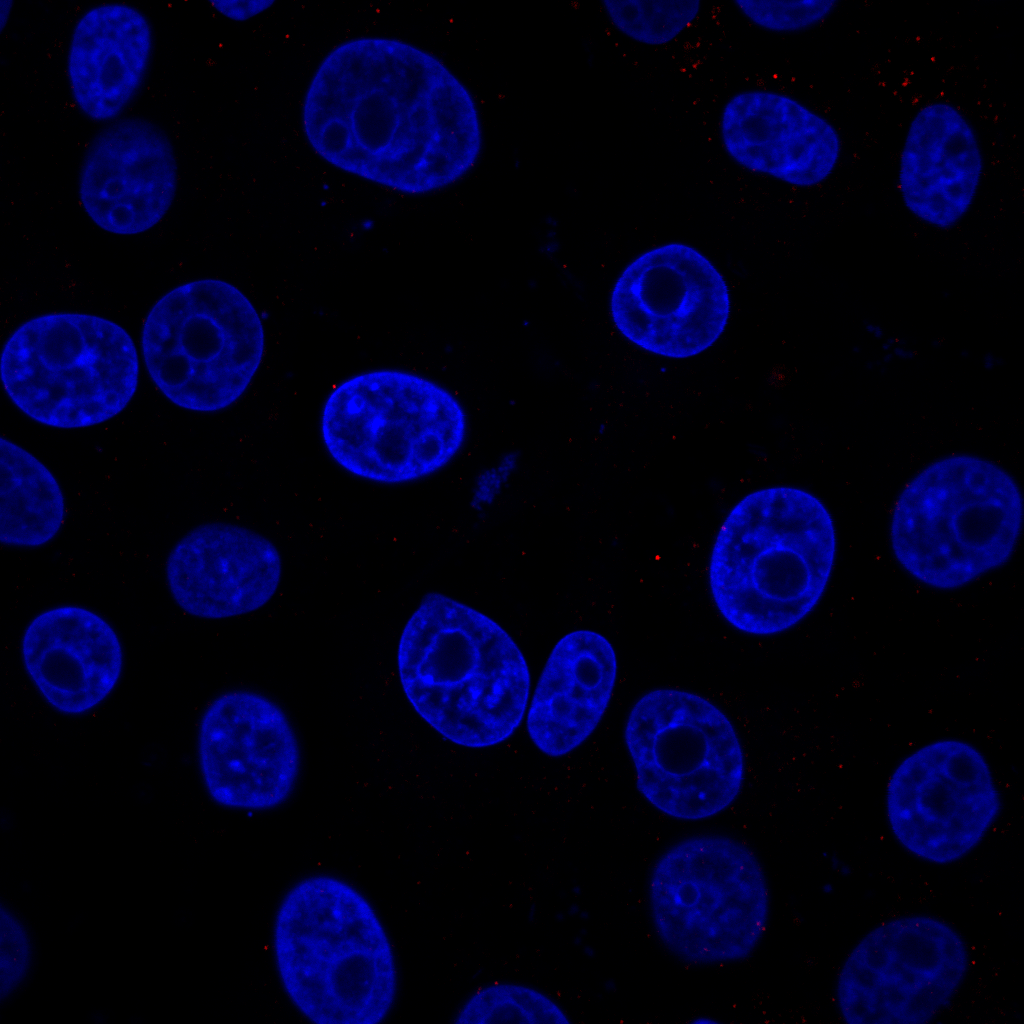

Supplement: S2 Data — This compressed folder contains the underlying numerical data and/or uncropped images used to generate the panels in Fig 2. (ZIP) [file pbio.3003736.s016.zip › S2 Data/Figure 2/G/KO+ANPEP/2-4-APN-4_RGB.tif]

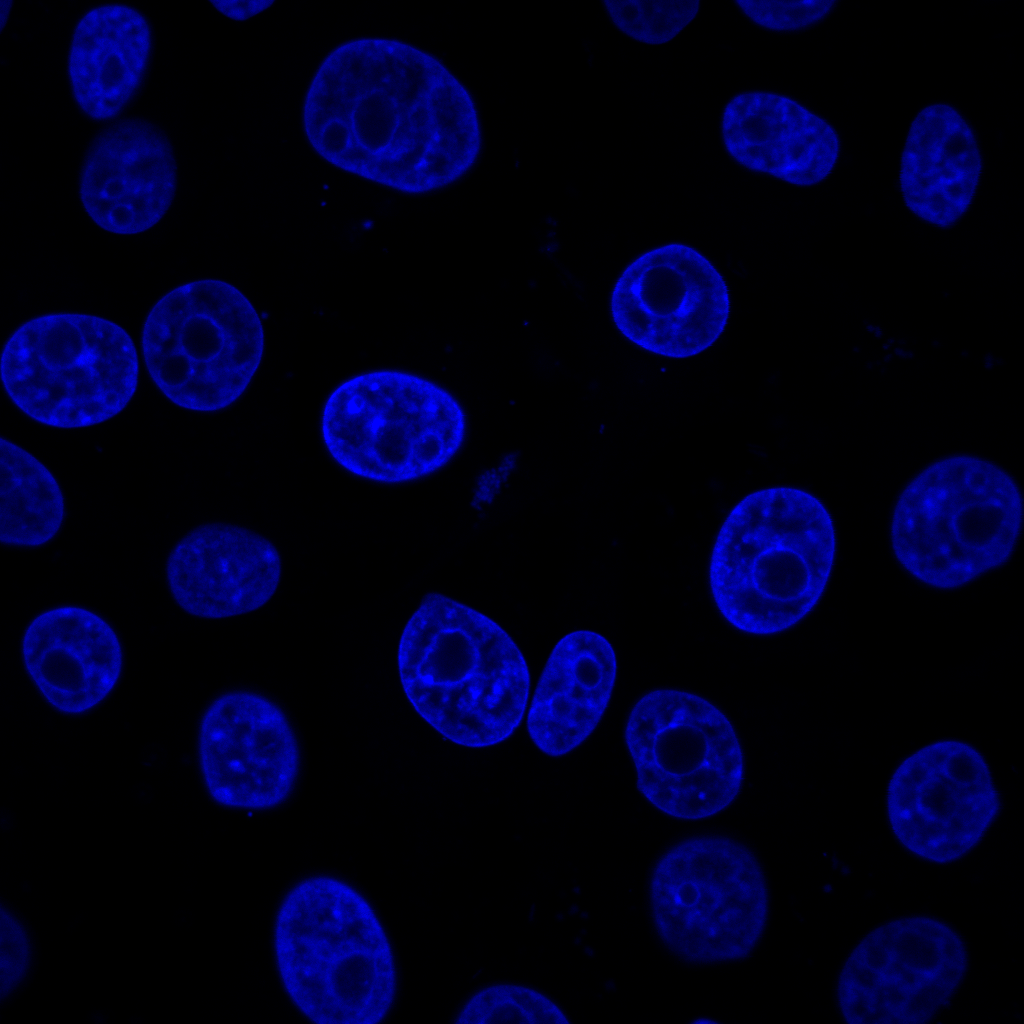

Supplement: S2 Data — This compressed folder contains the underlying numerical data and/or uncropped images used to generate the panels in Fig 2. (ZIP) [file pbio.3003736.s016.zip › S2 Data/Figure 2/G/KO+ANPEP/2-4-APN-4_RGB_DAPI.tif]

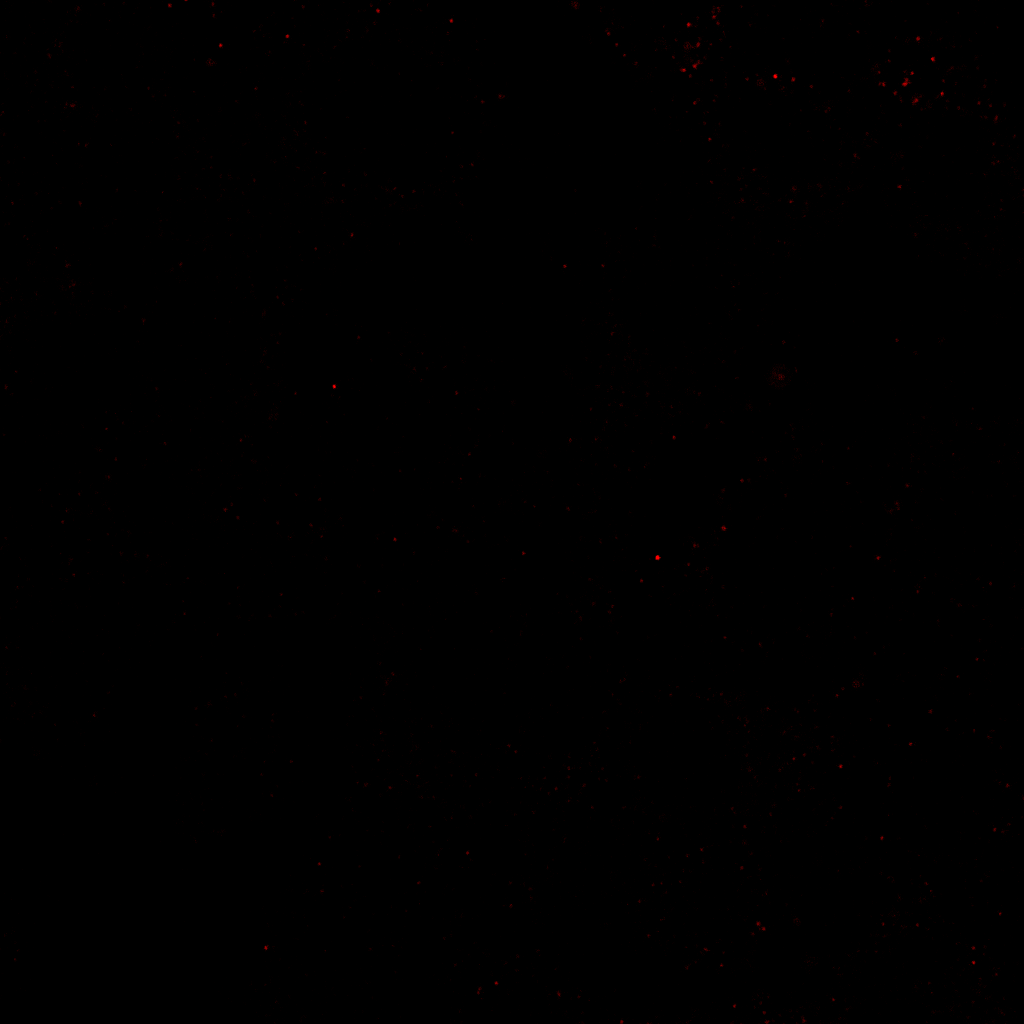

Supplement: S2 Data — This compressed folder contains the underlying numerical data and/or uncropped images used to generate the panels in Fig 2. (ZIP) [file pbio.3003736.s016.zip › S2 Data/Figure 2/G/KO+ANPEP/2-4-APN-4_RGB_TRITC.tif]

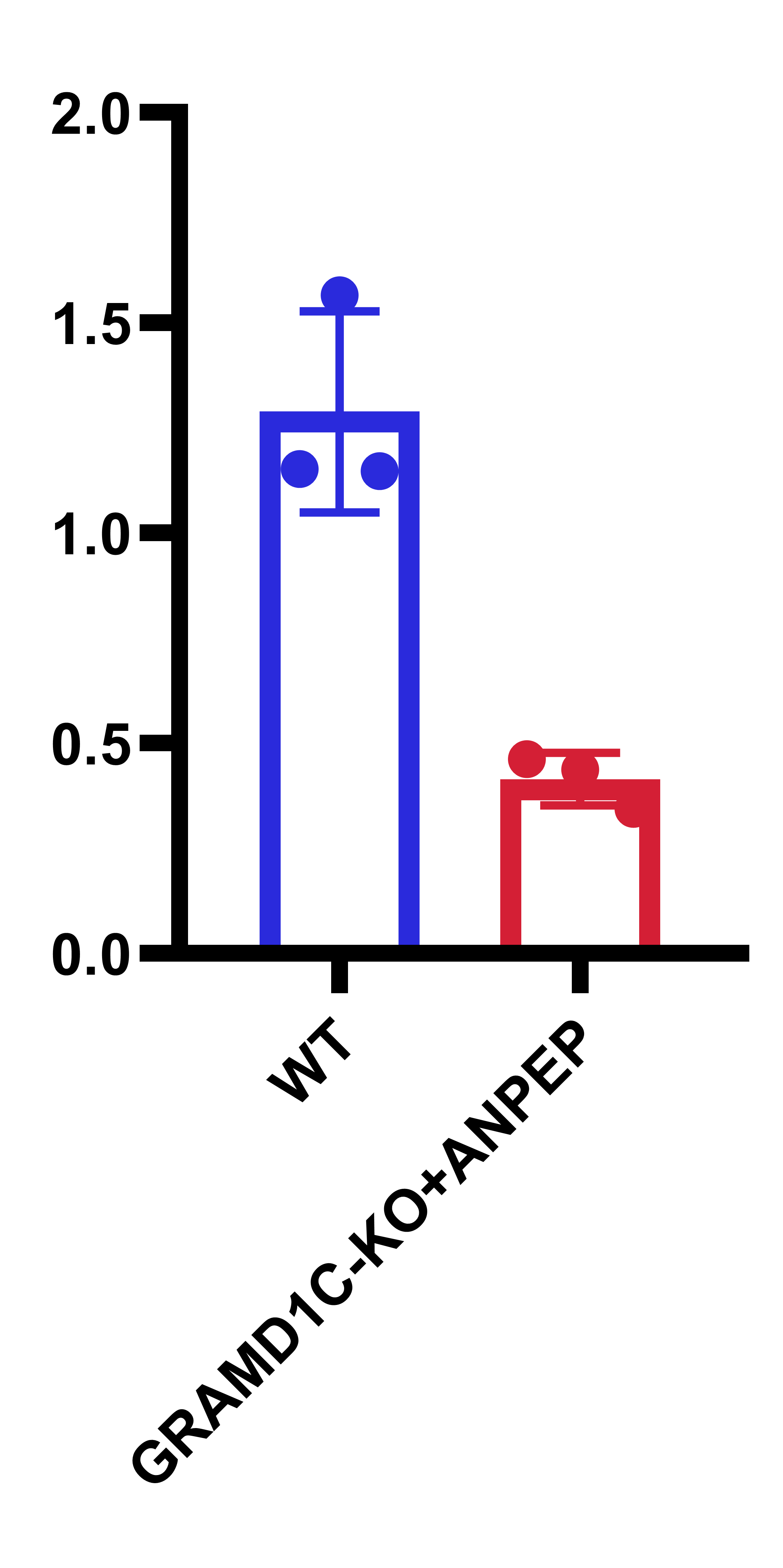

Supplement: S2 Data — This compressed folder contains the underlying numerical data and/or uncropped images used to generate the panels in Fig 2. (ZIP) [file pbio.3003736.s016.zip › S2 Data/Figure 2/G/ko+apn-dsrna-quantification.tif]

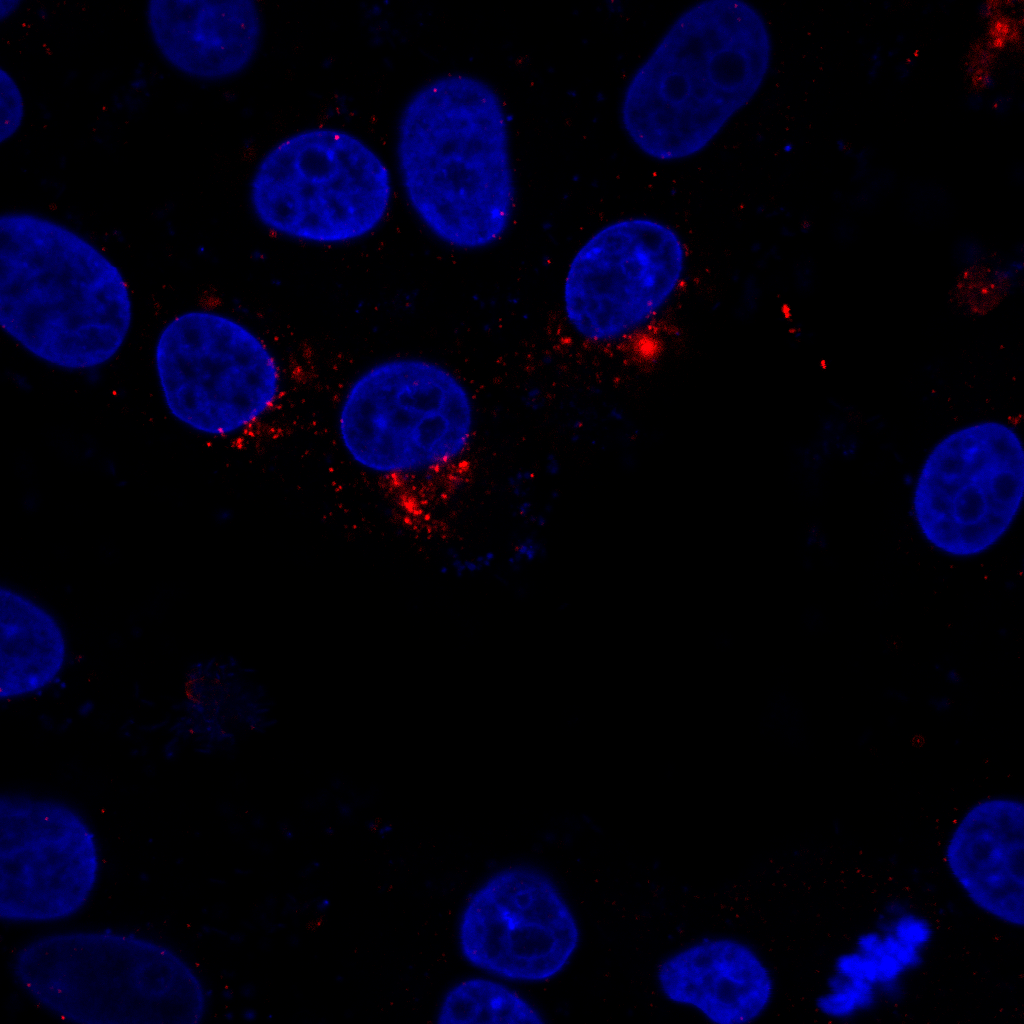

Supplement: S2 Data — This compressed folder contains the underlying numerical data and/or uncropped images used to generate the panels in Fig 2. (ZIP) [file pbio.3003736.s016.zip › S2 Data/Figure 2/G/PK/PK-1_RGB.tif]

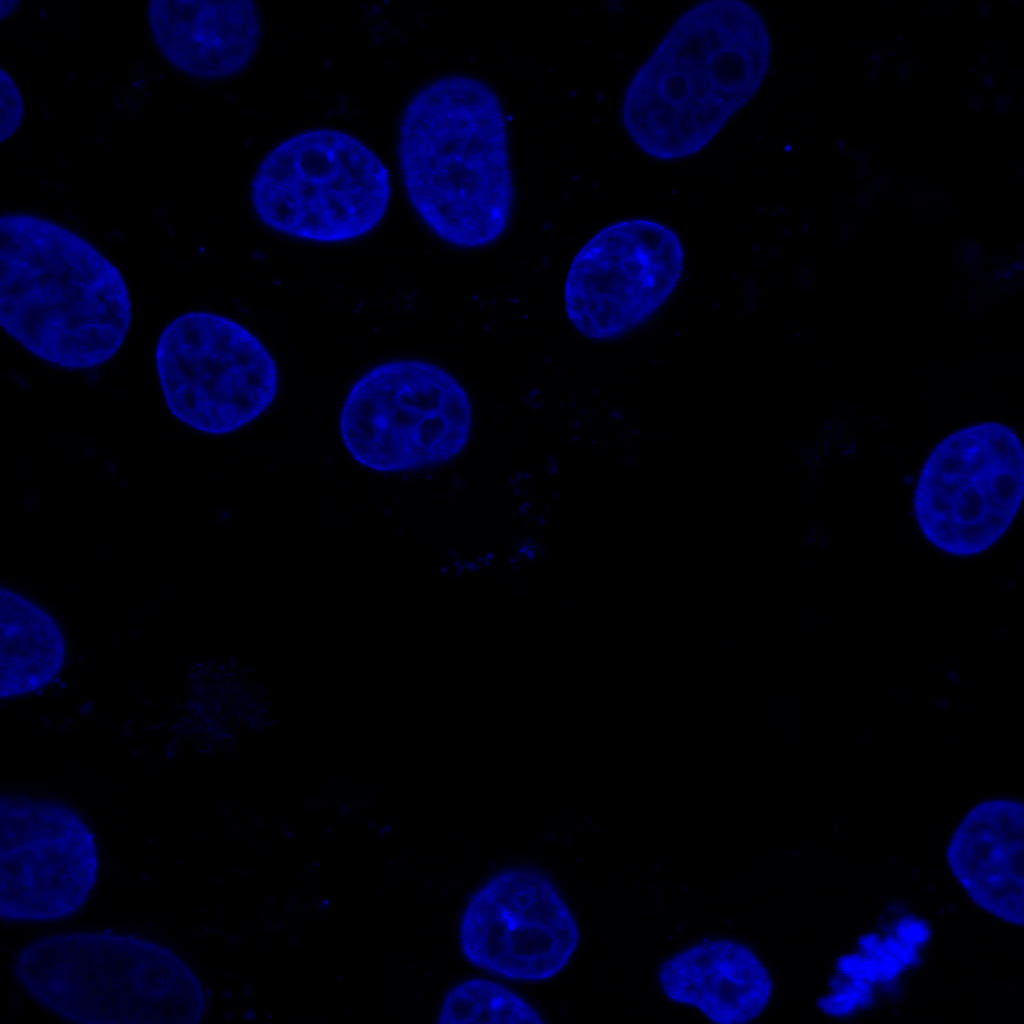

Supplement: S2 Data — This compressed folder contains the underlying numerical data and/or uncropped images used to generate the panels in Fig 2. (ZIP) [file pbio.3003736.s016.zip › S2 Data/Figure 2/G/PK/PK-1_RGB_DAPI.tif]

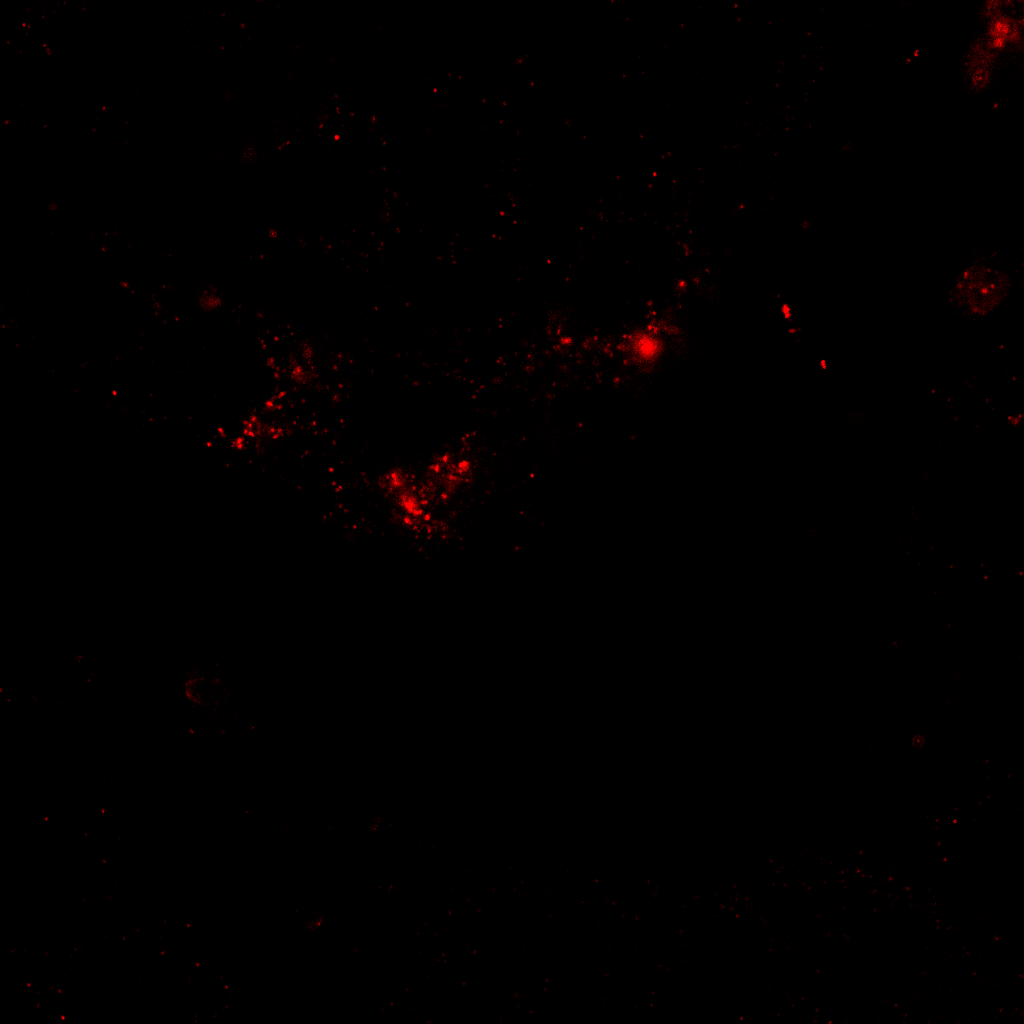

Supplement: S2 Data — This compressed folder contains the underlying numerical data and/or uncropped images used to generate the panels in Fig 2. (ZIP) [file pbio.3003736.s016.zip › S2 Data/Figure 2/G/PK/PK-1_RGB_TRITC.tif]

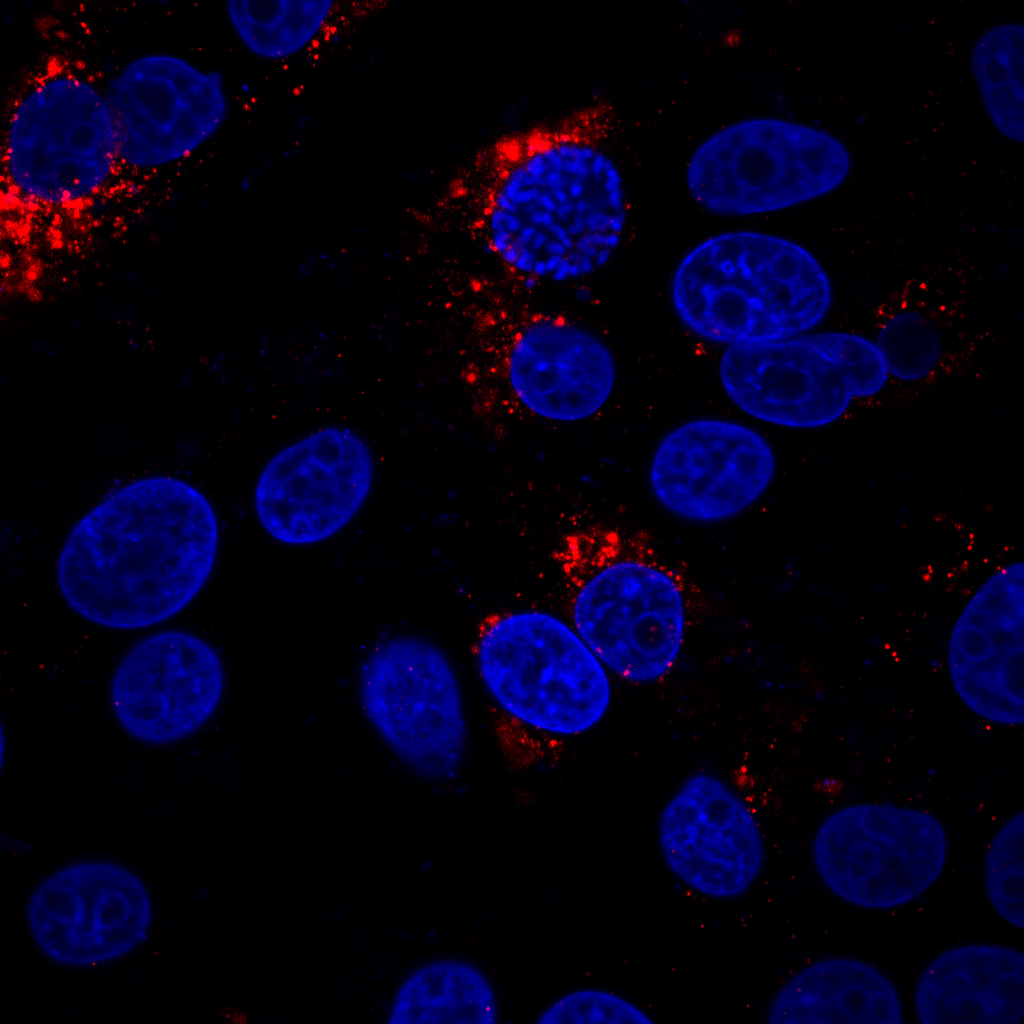

Supplement: S2 Data — This compressed folder contains the underlying numerical data and/or uncropped images used to generate the panels in Fig 2. (ZIP) [file pbio.3003736.s016.zip › S2 Data/Figure 2/G/PK/PK-2_RGB.tif]

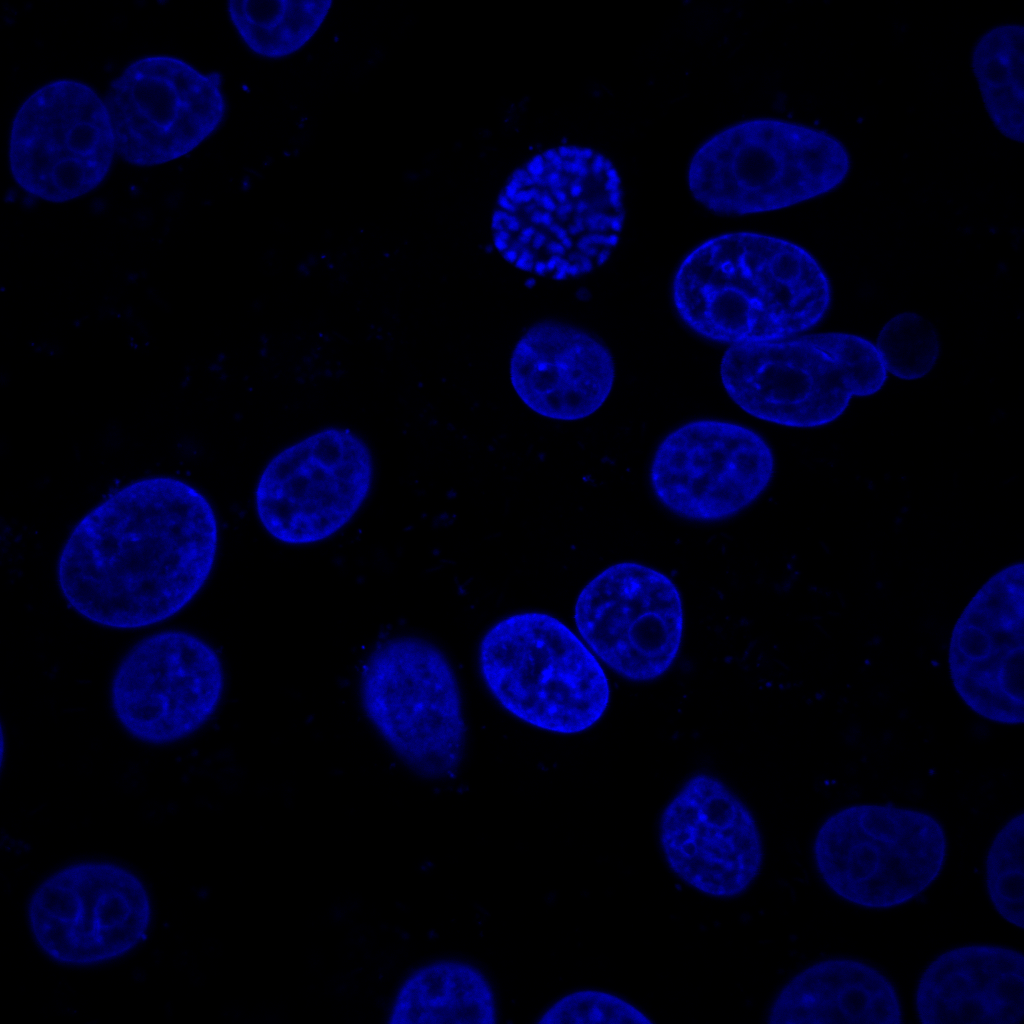

Supplement: S2 Data — This compressed folder contains the underlying numerical data and/or uncropped images used to generate the panels in Fig 2. (ZIP) [file pbio.3003736.s016.zip › S2 Data/Figure 2/G/PK/PK-2_RGB_DAPI.tif]

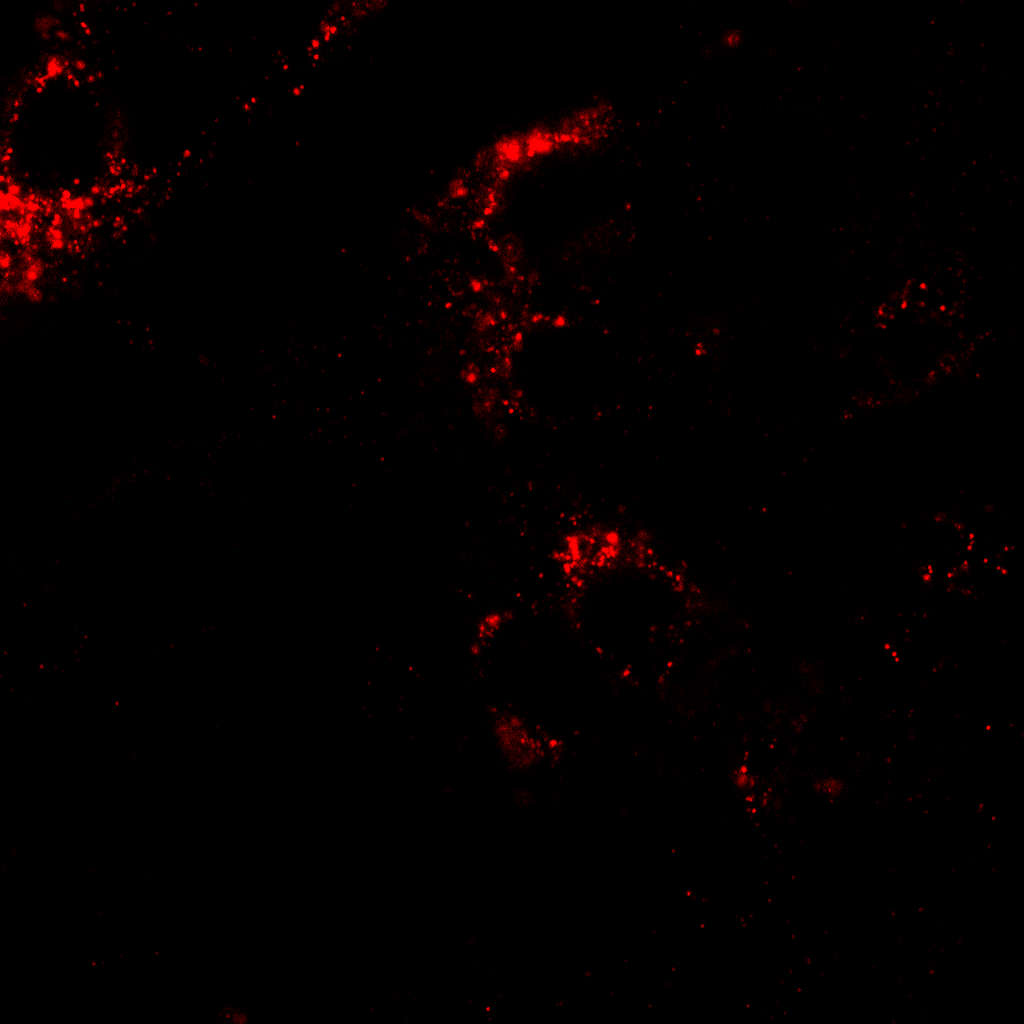

Supplement: S2 Data — This compressed folder contains the underlying numerical data and/or uncropped images used to generate the panels in Fig 2. (ZIP) [file pbio.3003736.s016.zip › S2 Data/Figure 2/G/PK/PK-2_RGB_TRITC.tif]

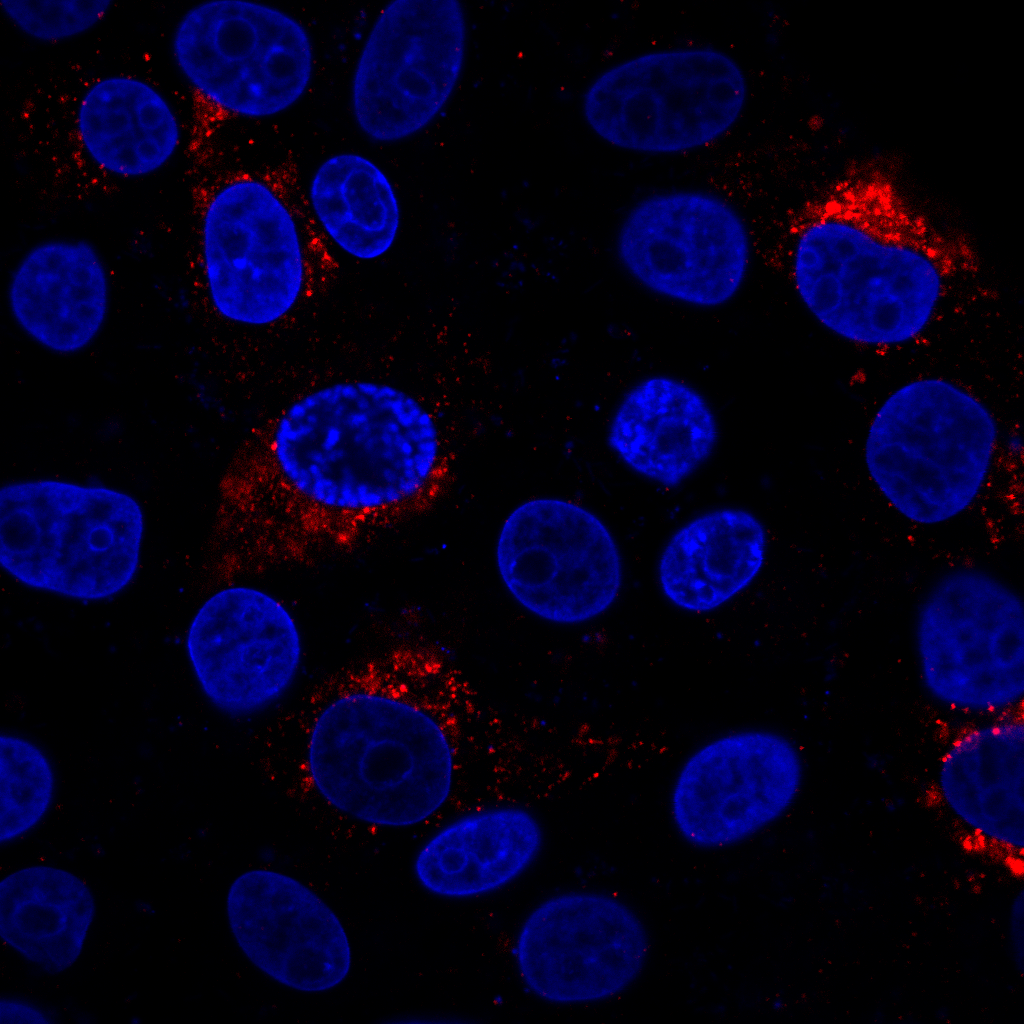

Supplement: S2 Data — This compressed folder contains the underlying numerical data and/or uncropped images used to generate the panels in Fig 2. (ZIP) [file pbio.3003736.s016.zip › S2 Data/Figure 2/G/PK/PK-3_RGB.tif]

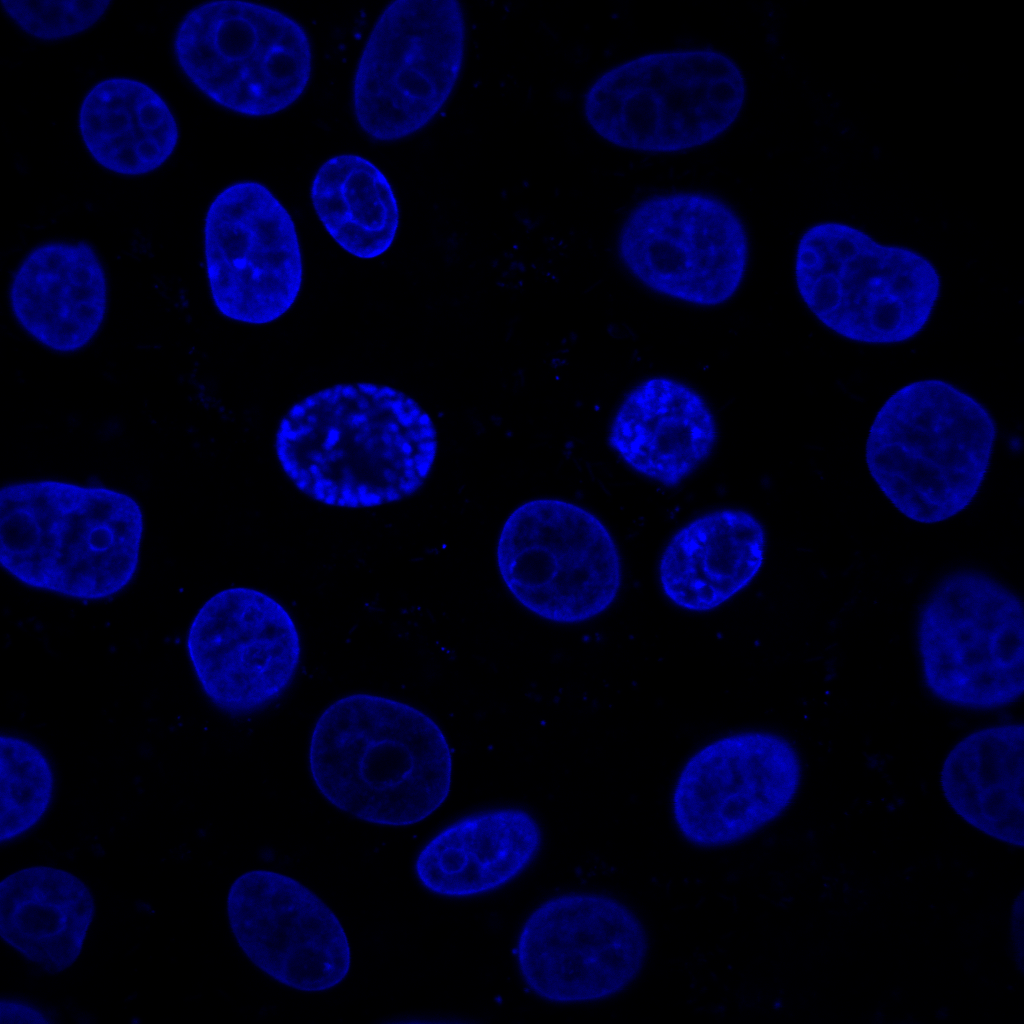

Supplement: S2 Data — This compressed folder contains the underlying numerical data and/or uncropped images used to generate the panels in Fig 2. (ZIP) [file pbio.3003736.s016.zip › S2 Data/Figure 2/G/PK/PK-3_RGB_DAPI.tif]

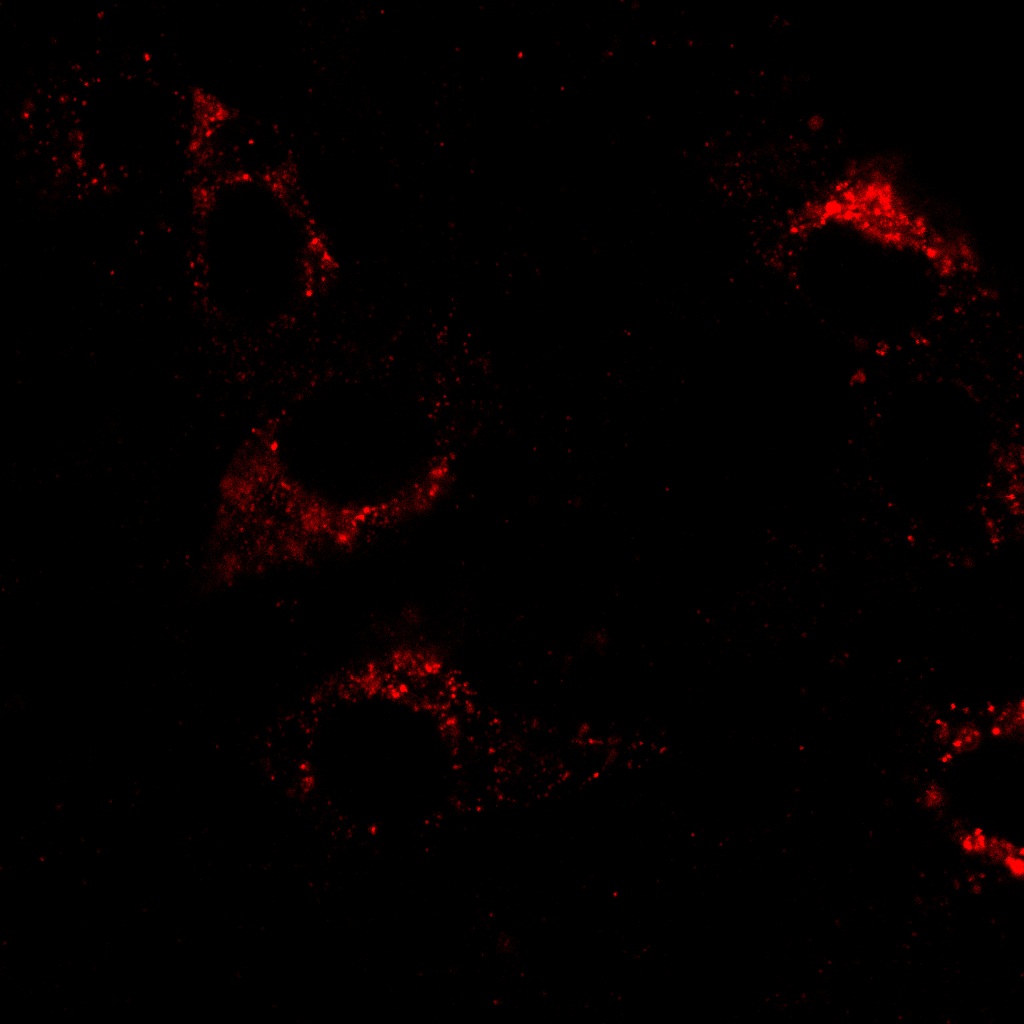

Supplement: S2 Data — This compressed folder contains the underlying numerical data and/or uncropped images used to generate the panels in Fig 2. (ZIP) [file pbio.3003736.s016.zip › S2 Data/Figure 2/G/PK/PK-3_RGB_TRITC.tif]

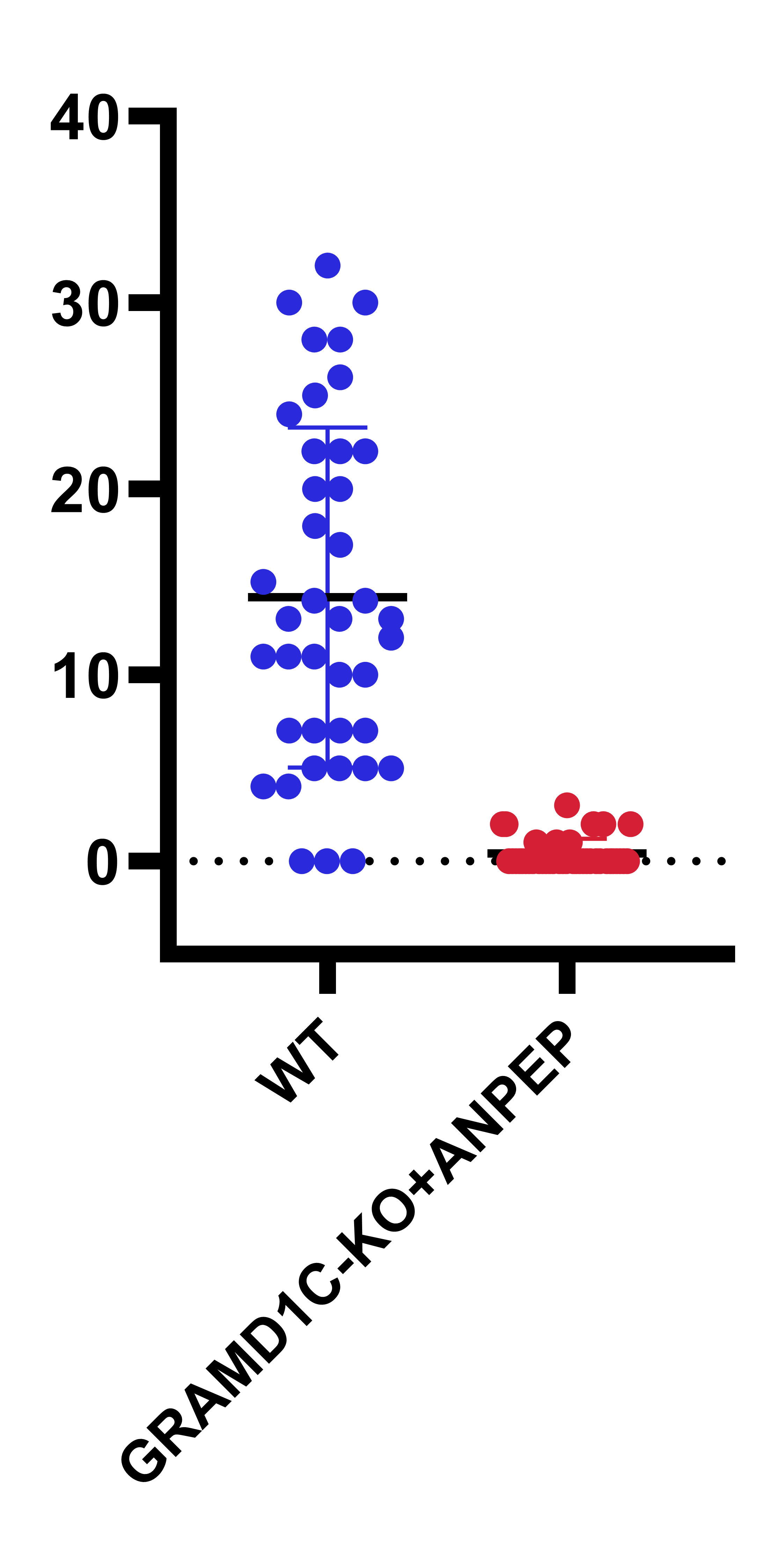

Supplement: S2 Data — This compressed folder contains the underlying numerical data and/or uncropped images used to generate the panels in Fig 2. (ZIP) [file pbio.3003736.s016.zip › S2 Data/Figure 2/H/DMV.tif]

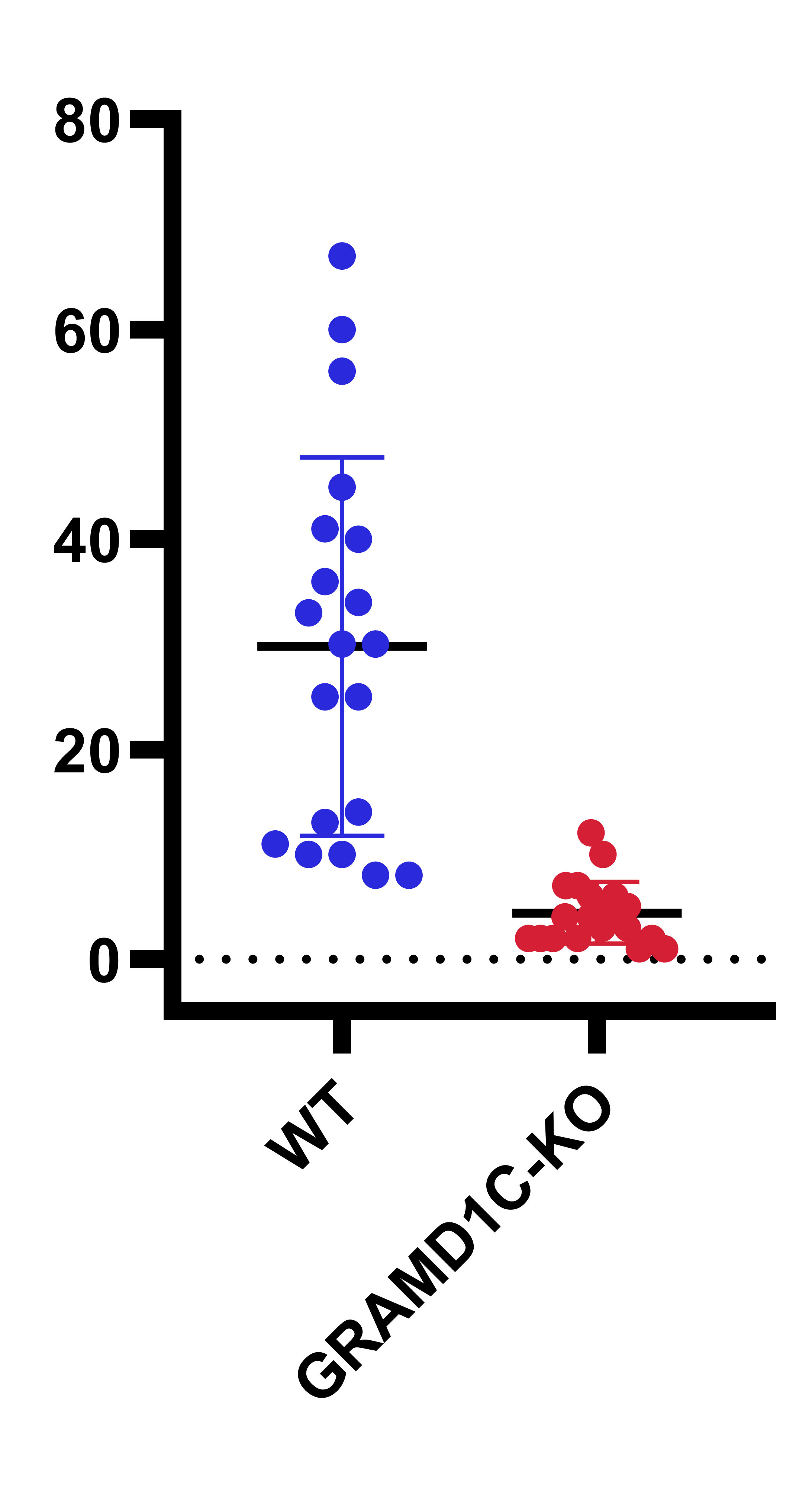

Supplement: S2 Data — This compressed folder contains the underlying numerical data and/or uncropped images used to generate the panels in Fig 2. (ZIP) [file pbio.3003736.s016.zip › S2 Data/Figure 2/I/pk-ko-dmv.tif]

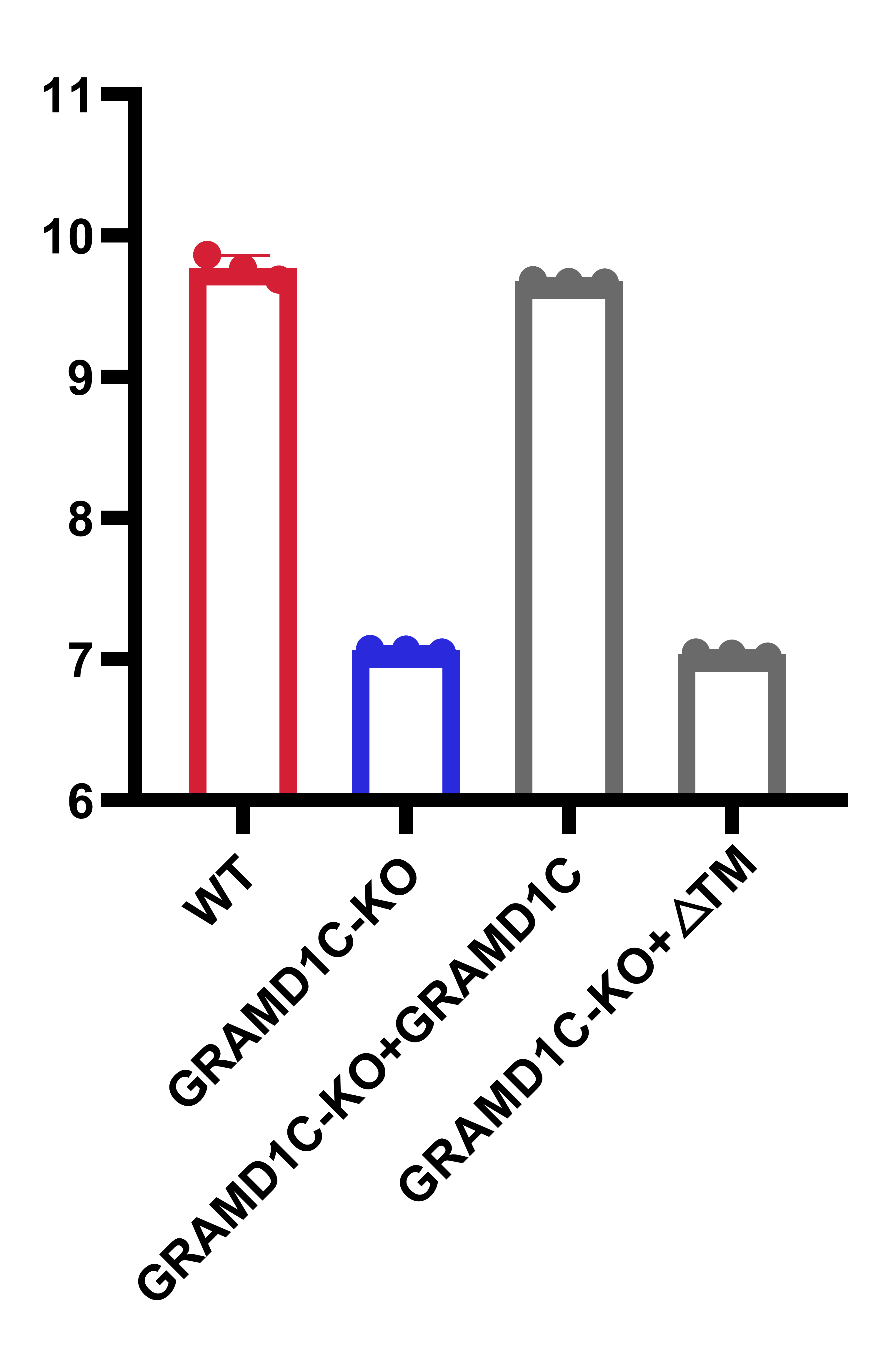

Supplement: S3 Data — This compressed folder contains the underlying numerical data and/or uncropped images used to generate the panels in Figs 3I and 4. (ZIP) [file pbio.3003736.s017.zip › S3 Data/Figure 3I/domain-rescue.tif]

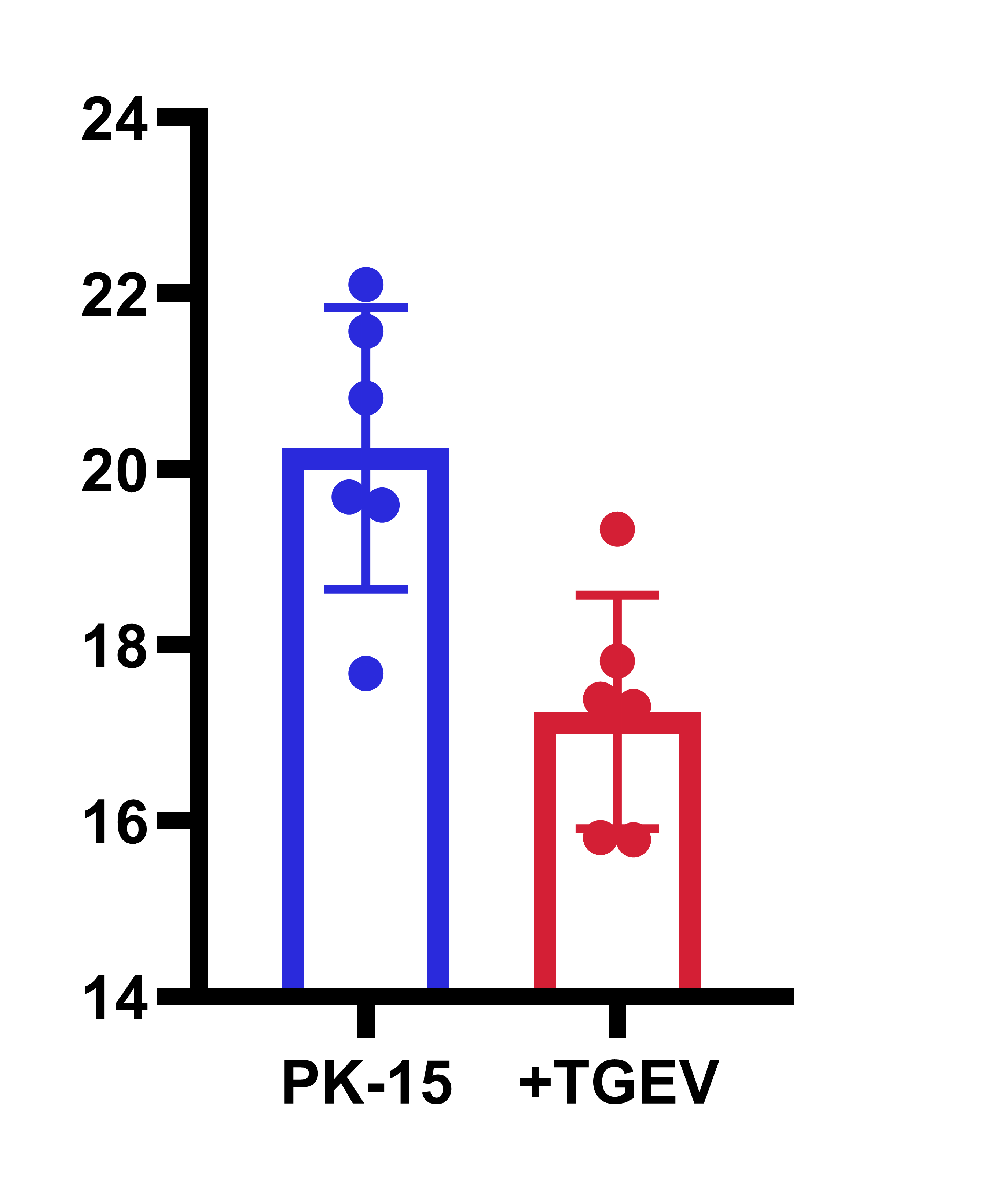

Supplement: S3 Data — This compressed folder contains the underlying numerical data and/or uncropped images used to generate the panels in Figs 3I and 4. (ZIP) [file pbio.3003736.s017.zip › S3 Data/Figure4/E/cho+TGEV.tif]

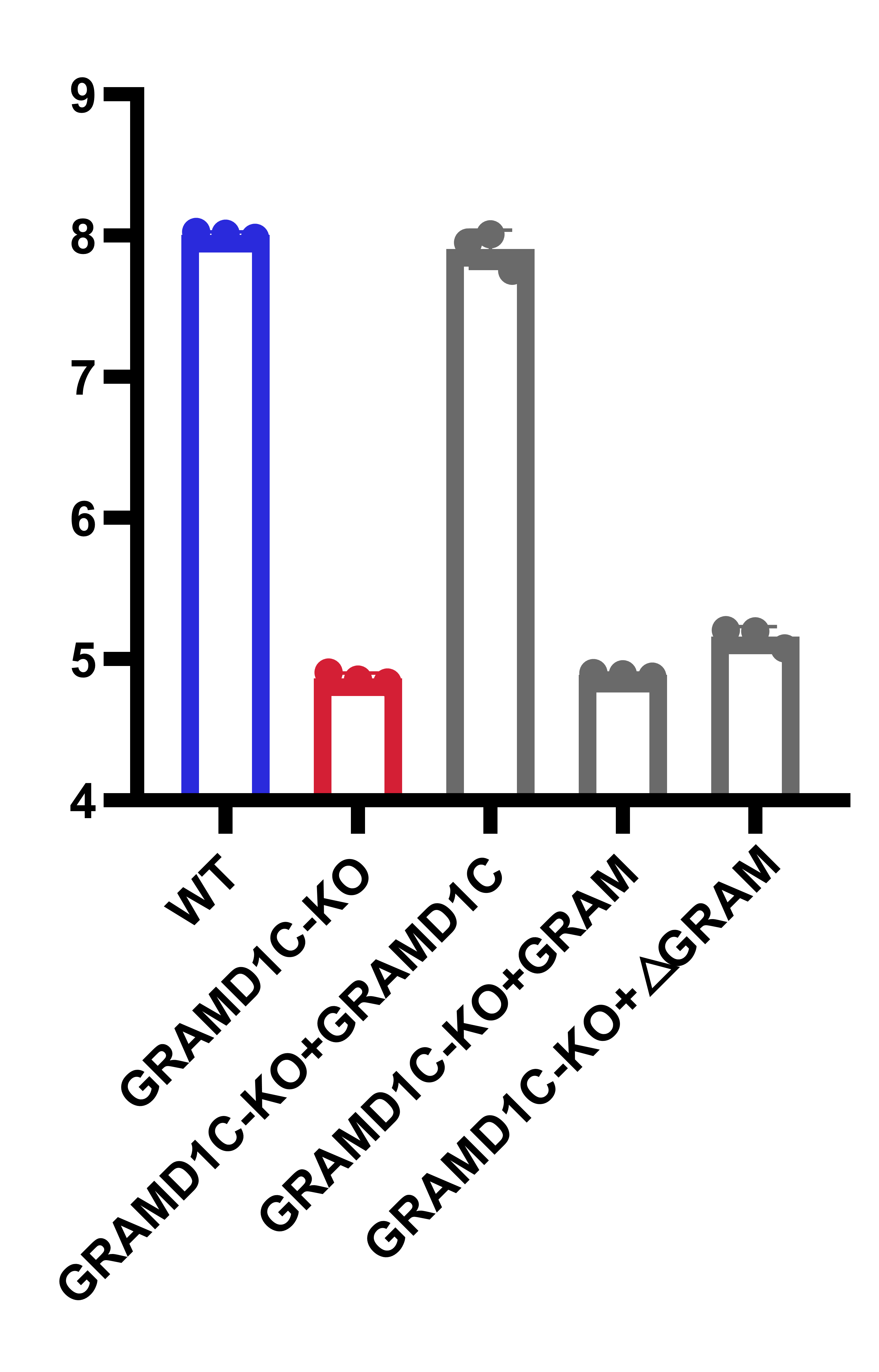

Supplement: S3 Data — This compressed folder contains the underlying numerical data and/or uncropped images used to generate the panels in Figs 3I and 4. (ZIP) [file pbio.3003736.s017.zip › S3 Data/Figure4/G/domain-rescue.tif]

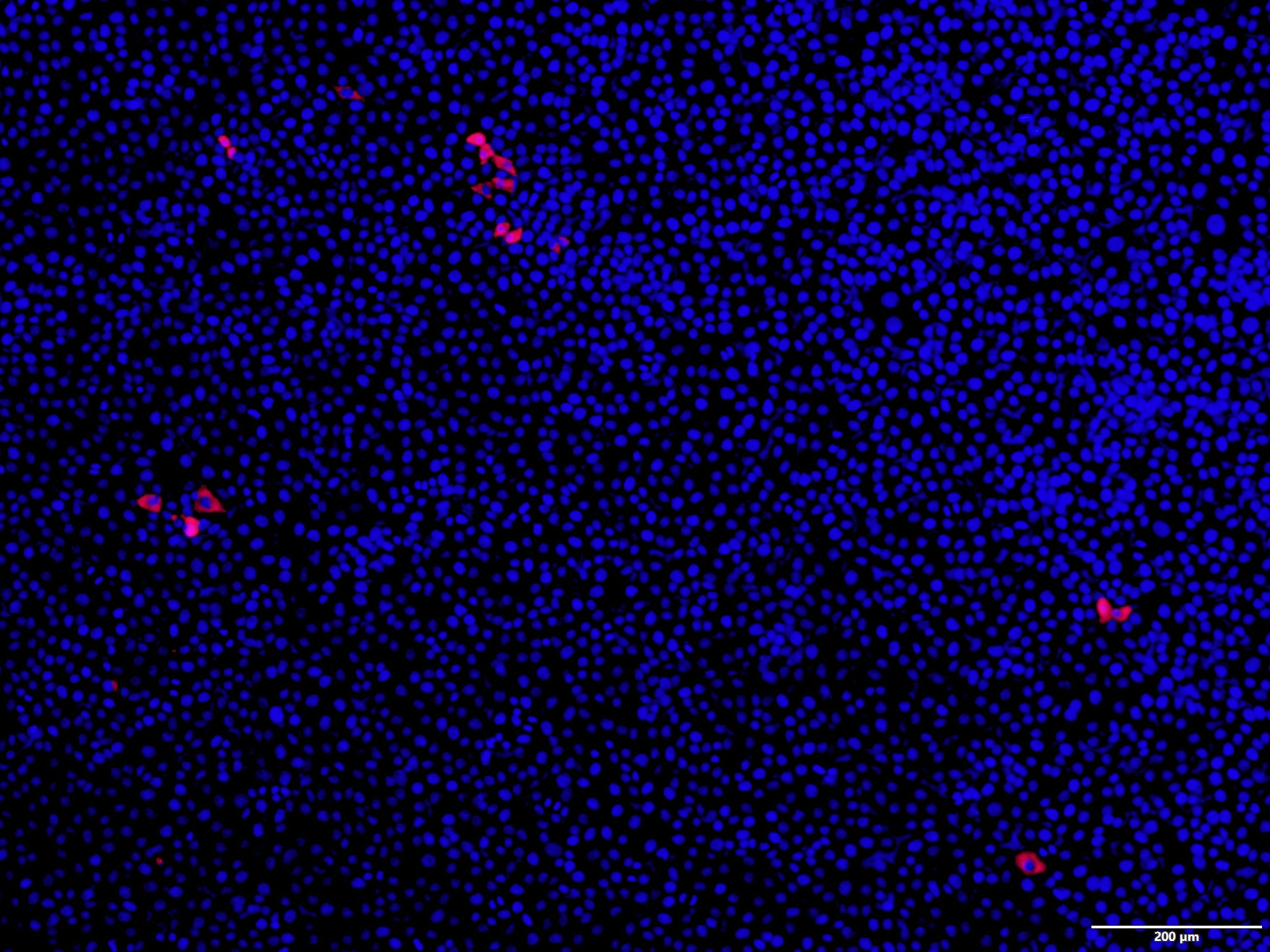

Supplement: S3 Data — This compressed folder contains the underlying numerical data and/or uncropped images used to generate the panels in Figs 3I and 4. (ZIP) [file pbio.3003736.s017.zip › S3 Data/Figure4/H/KO/22-5-1.jpg]

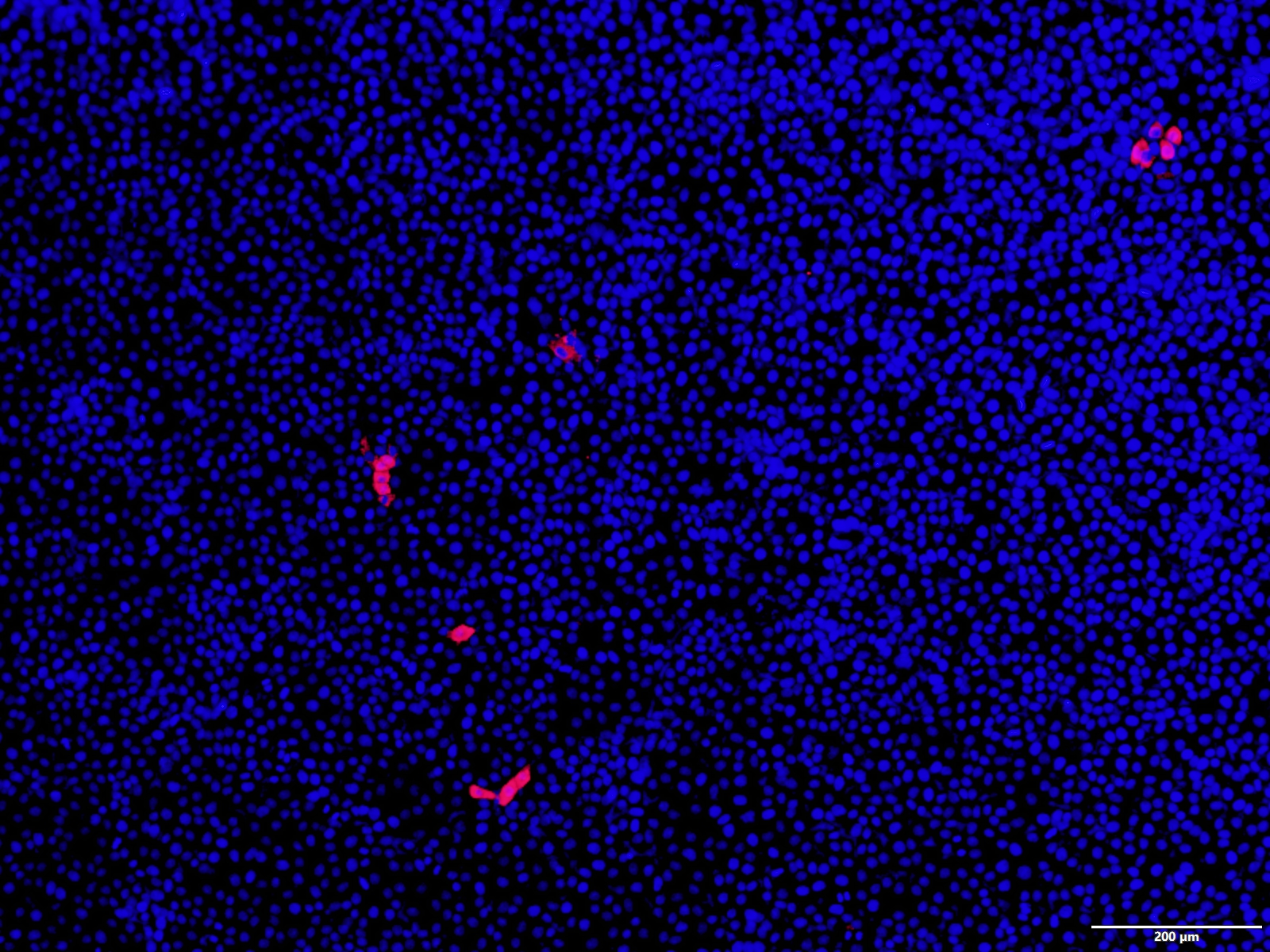

Supplement: S3 Data — This compressed folder contains the underlying numerical data and/or uncropped images used to generate the panels in Figs 3I and 4. (ZIP) [file pbio.3003736.s017.zip › S3 Data/Figure4/H/KO/22-5-2.jpg]

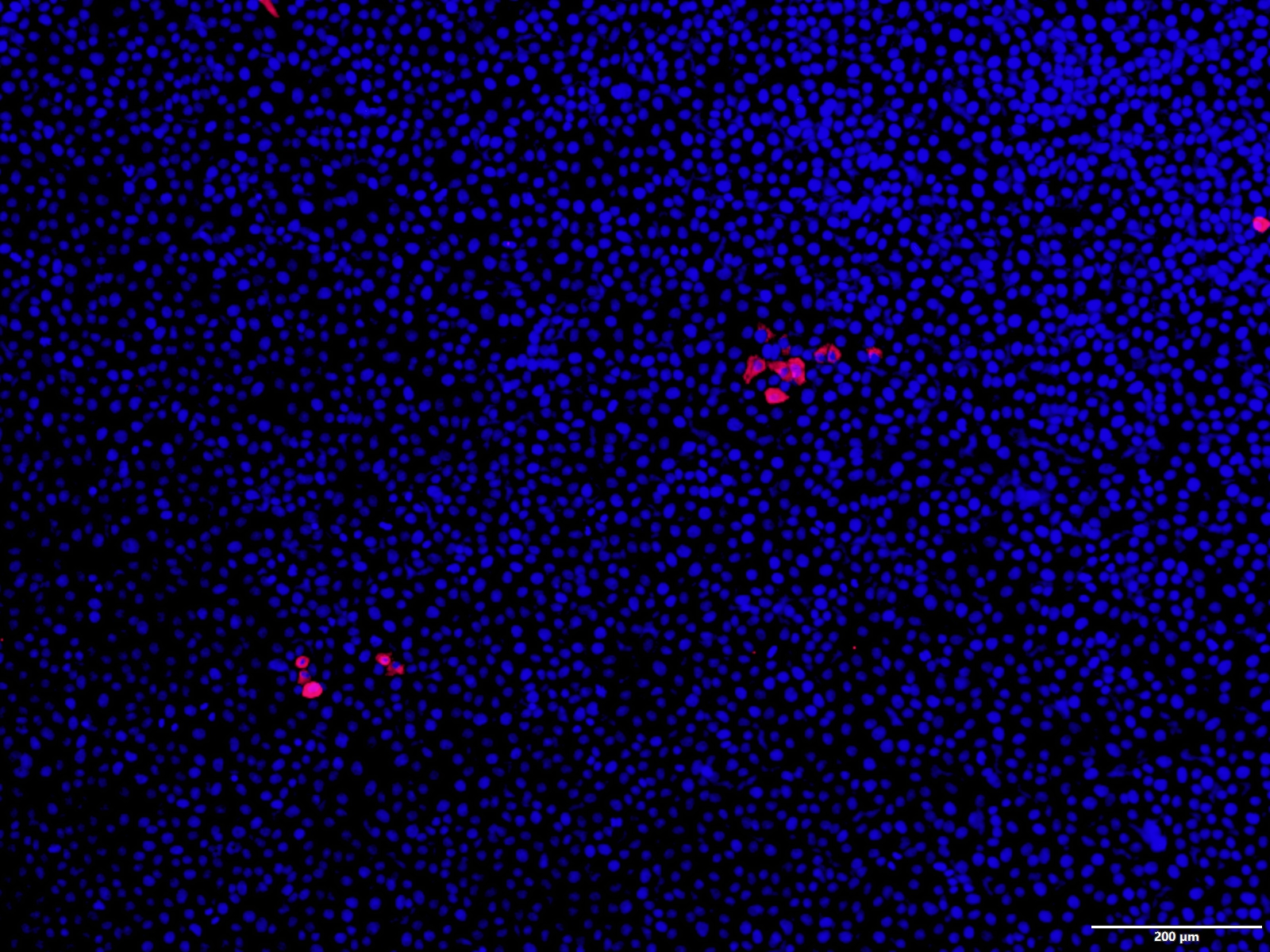

Supplement: S3 Data — This compressed folder contains the underlying numerical data and/or uncropped images used to generate the panels in Figs 3I and 4. (ZIP) [file pbio.3003736.s017.zip › S3 Data/Figure4/H/KO/22-5-3.jpg]

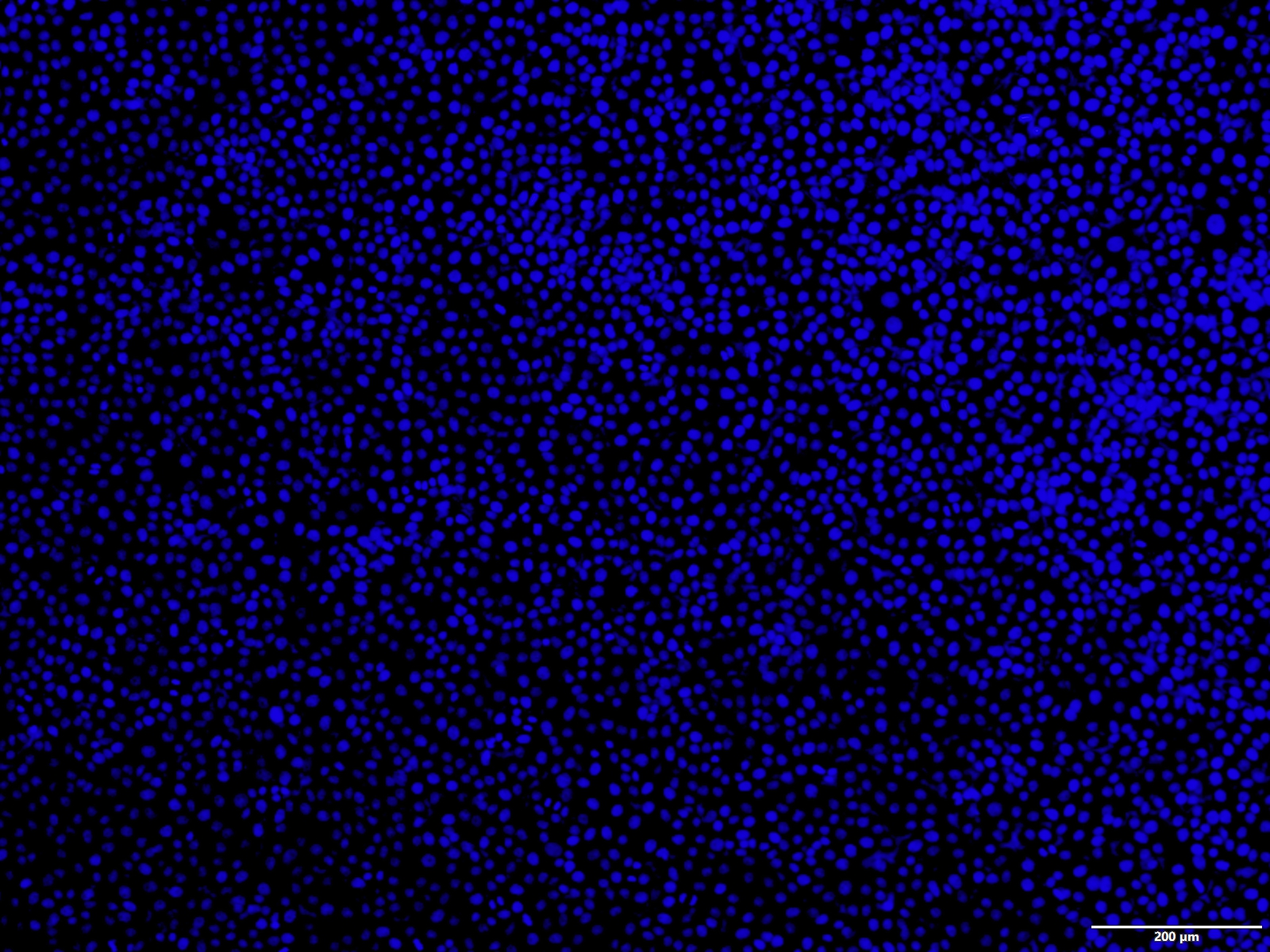

Supplement: S3 Data — This compressed folder contains the underlying numerical data and/or uncropped images used to generate the panels in Figs 3I and 4. (ZIP) [file pbio.3003736.s017.zip › S3 Data/Figure4/H/KO/22-5-dapi-1.jpg]

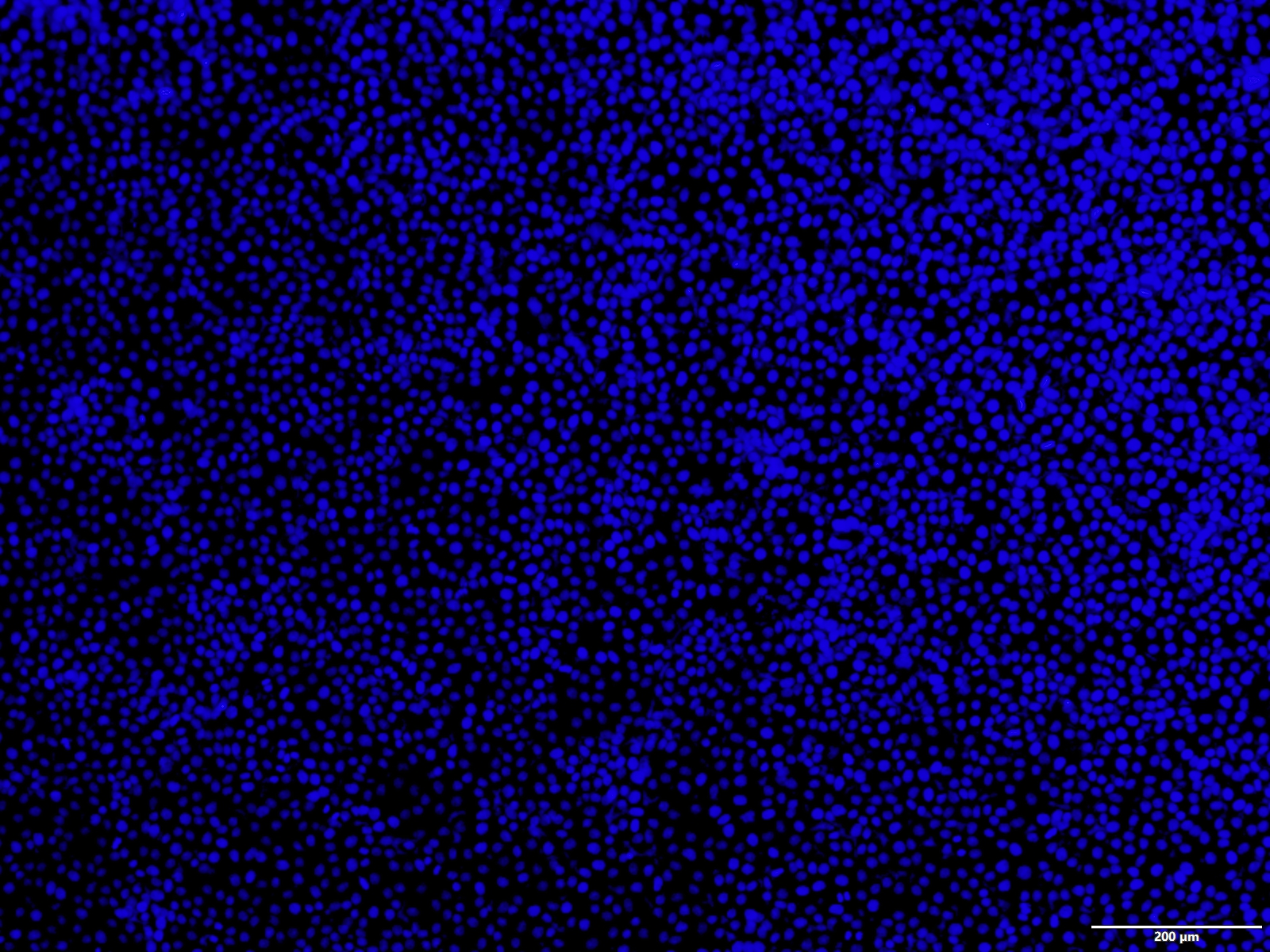

Supplement: S3 Data — This compressed folder contains the underlying numerical data and/or uncropped images used to generate the panels in Figs 3I and 4. (ZIP) [file pbio.3003736.s017.zip › S3 Data/Figure4/H/KO/22-5-dapi-2.jpg]

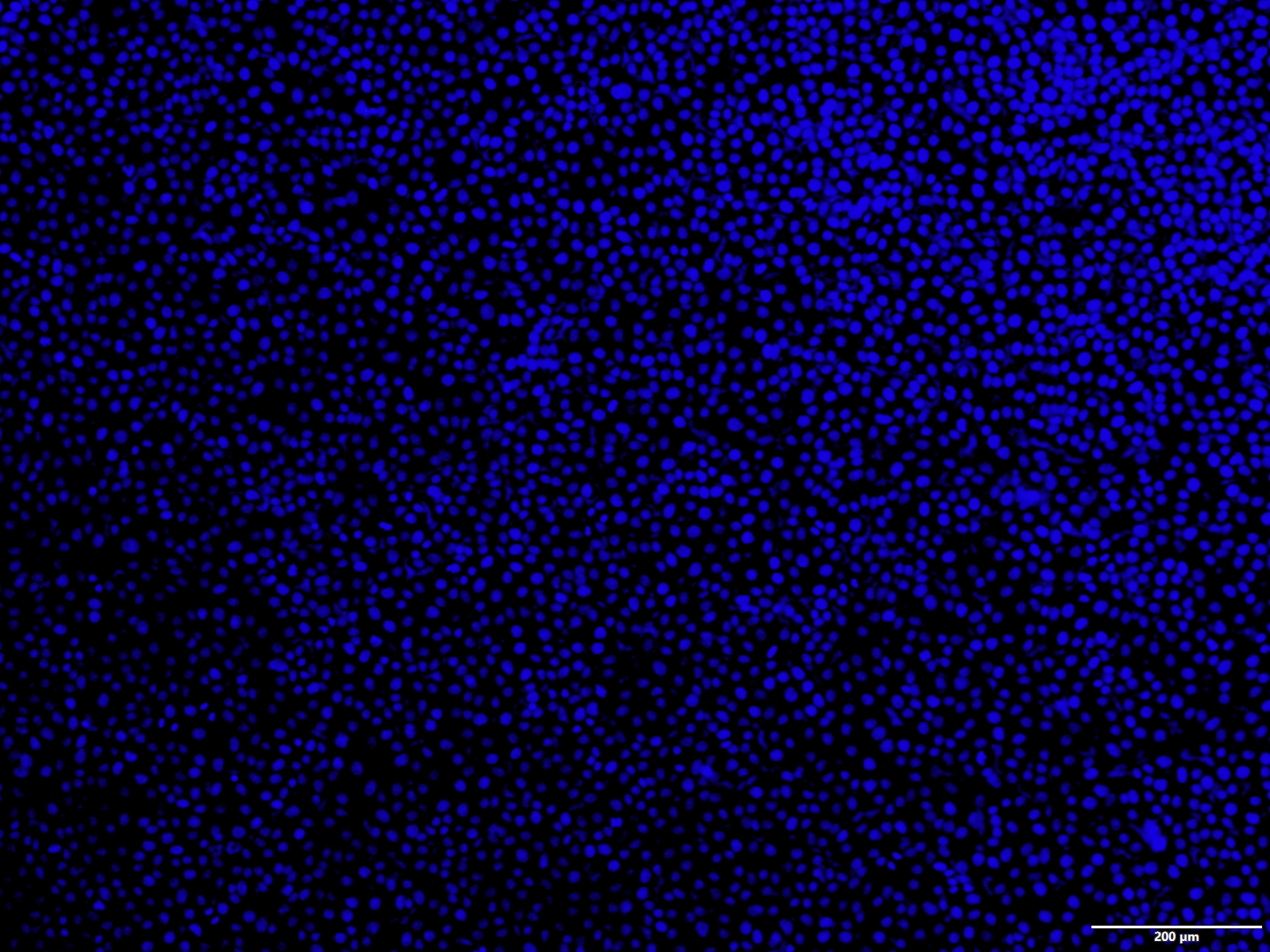

Supplement: S3 Data — This compressed folder contains the underlying numerical data and/or uncropped images used to generate the panels in Figs 3I and 4. (ZIP) [file pbio.3003736.s017.zip › S3 Data/Figure4/H/KO/22-5-dapi-3.jpg]

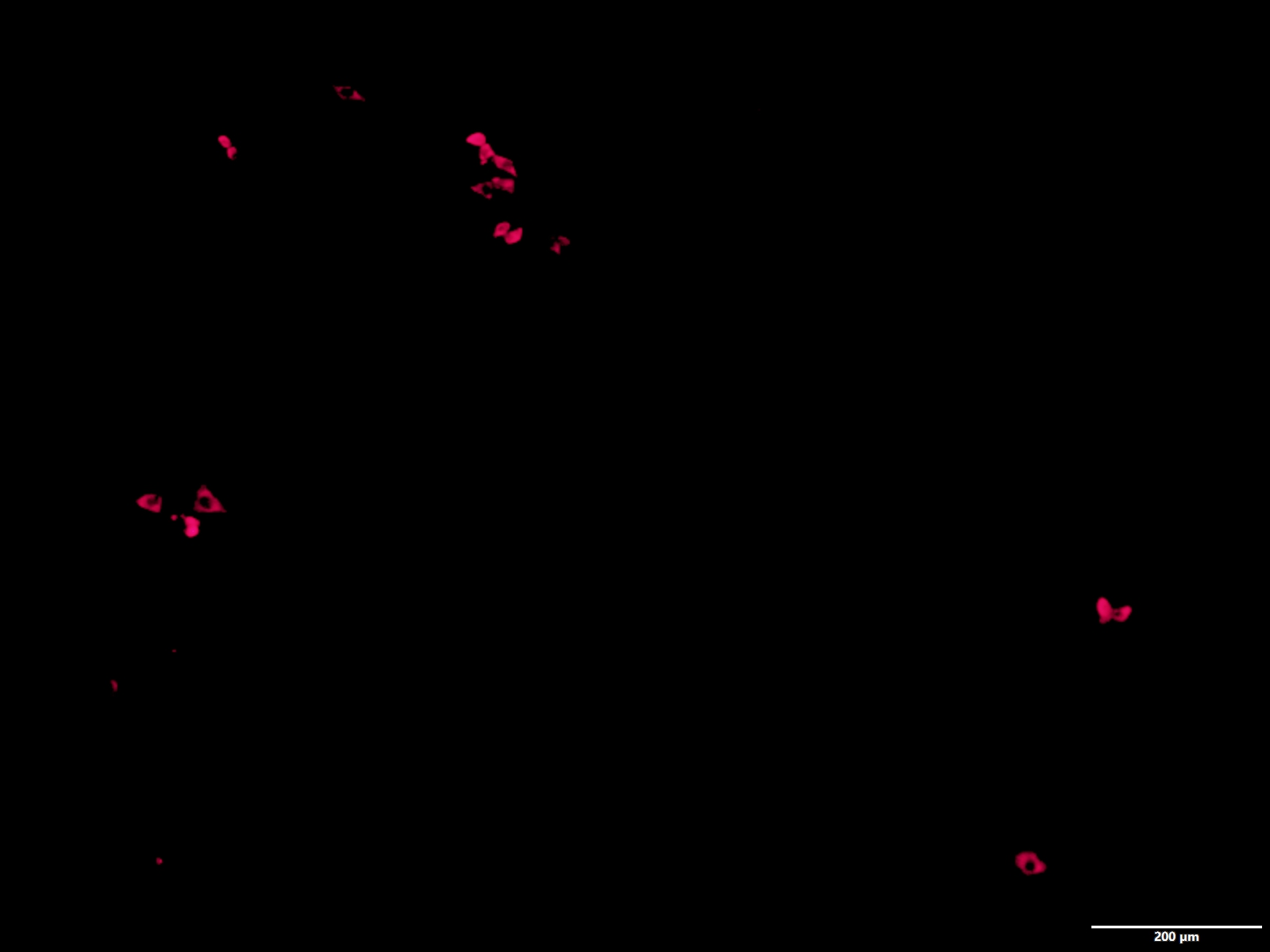

Supplement: S3 Data — This compressed folder contains the underlying numerical data and/or uncropped images used to generate the panels in Figs 3I and 4. (ZIP) [file pbio.3003736.s017.zip › S3 Data/Figure4/H/KO/22-5-tgev-n-1.jpg]

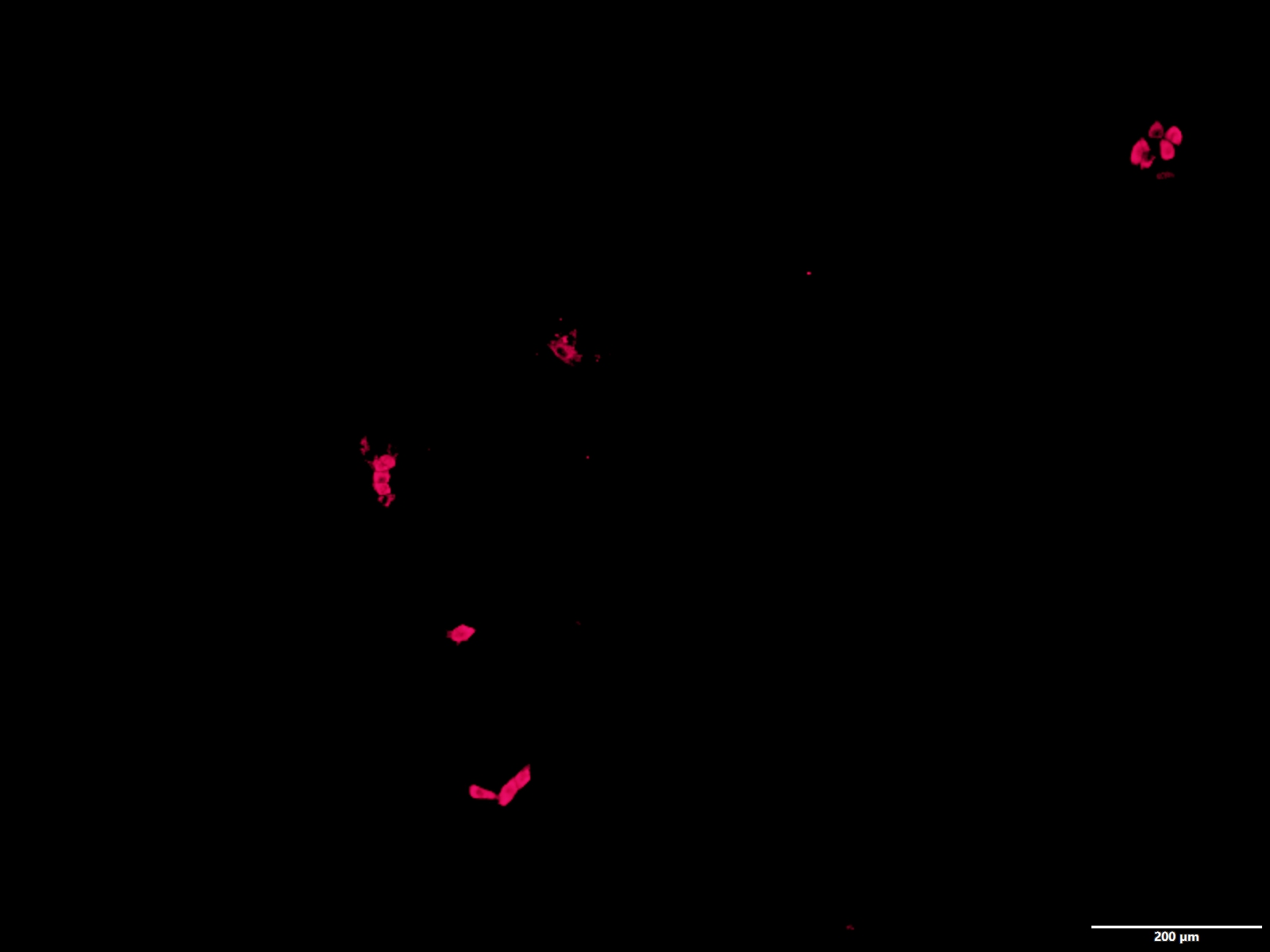

Supplement: S3 Data — This compressed folder contains the underlying numerical data and/or uncropped images used to generate the panels in Figs 3I and 4. (ZIP) [file pbio.3003736.s017.zip › S3 Data/Figure4/H/KO/22-5-tgev-n-2.jpg]

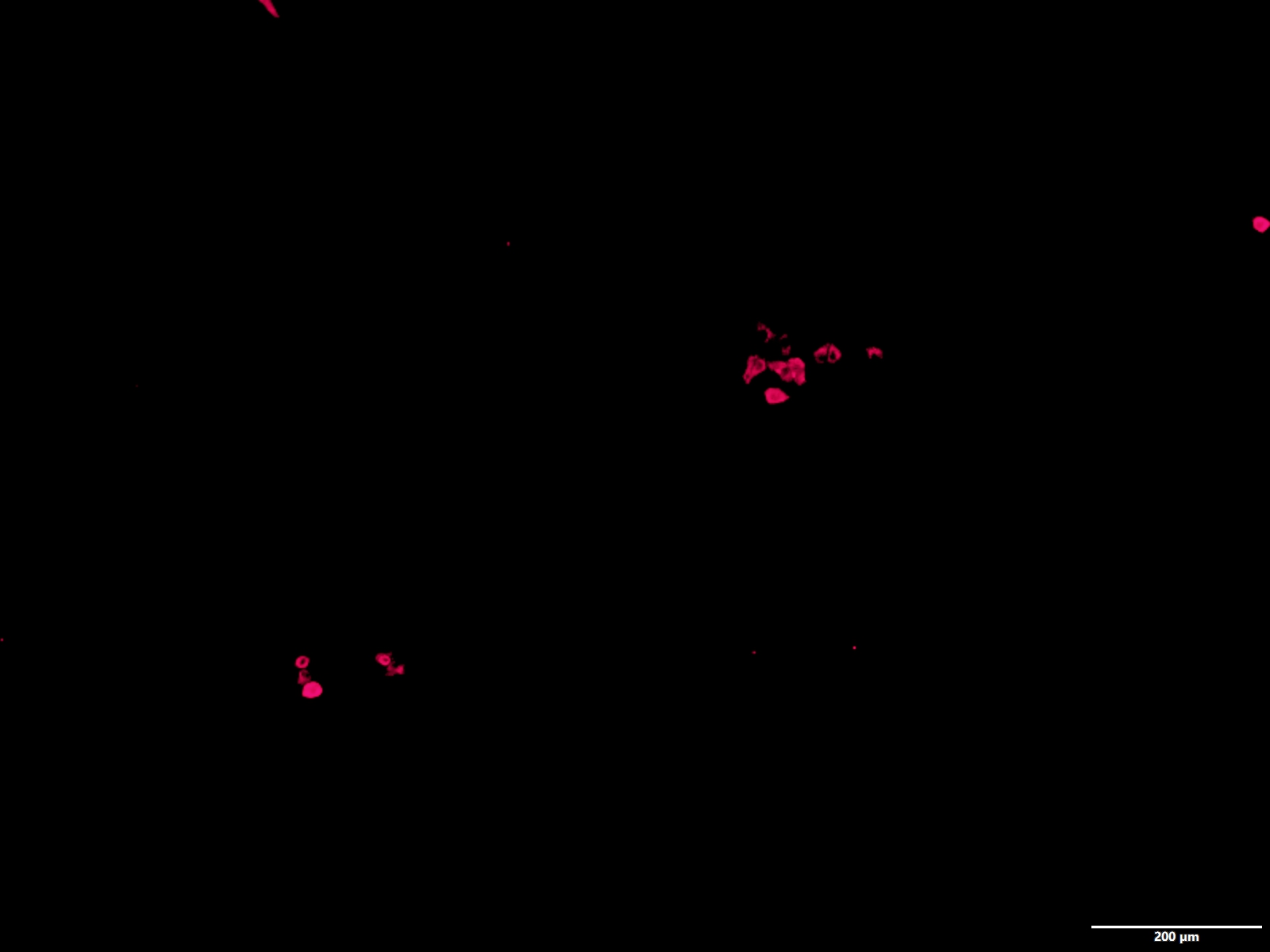

Supplement: S3 Data — This compressed folder contains the underlying numerical data and/or uncropped images used to generate the panels in Figs 3I and 4. (ZIP) [file pbio.3003736.s017.zip › S3 Data/Figure4/H/KO/22-5-tgev-n-3.jpg]

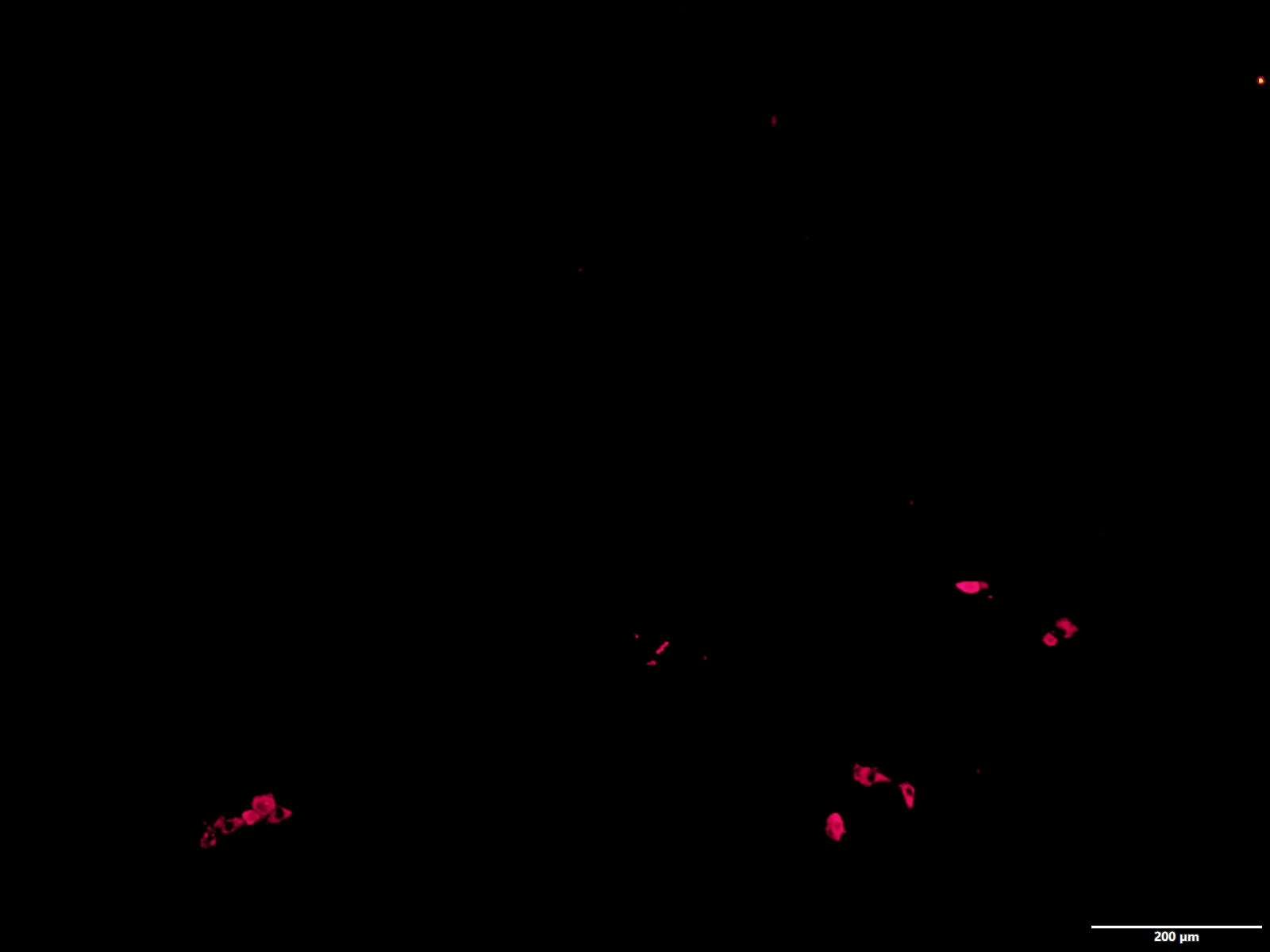

Supplement: S3 Data — This compressed folder contains the underlying numerical data and/or uncropped images used to generate the panels in Figs 3I and 4. (ZIP) [file pbio.3003736.s017.zip › S3 Data/Figure4/H/KO+CHO/22-5+dan--tgev-n-1.jpg]

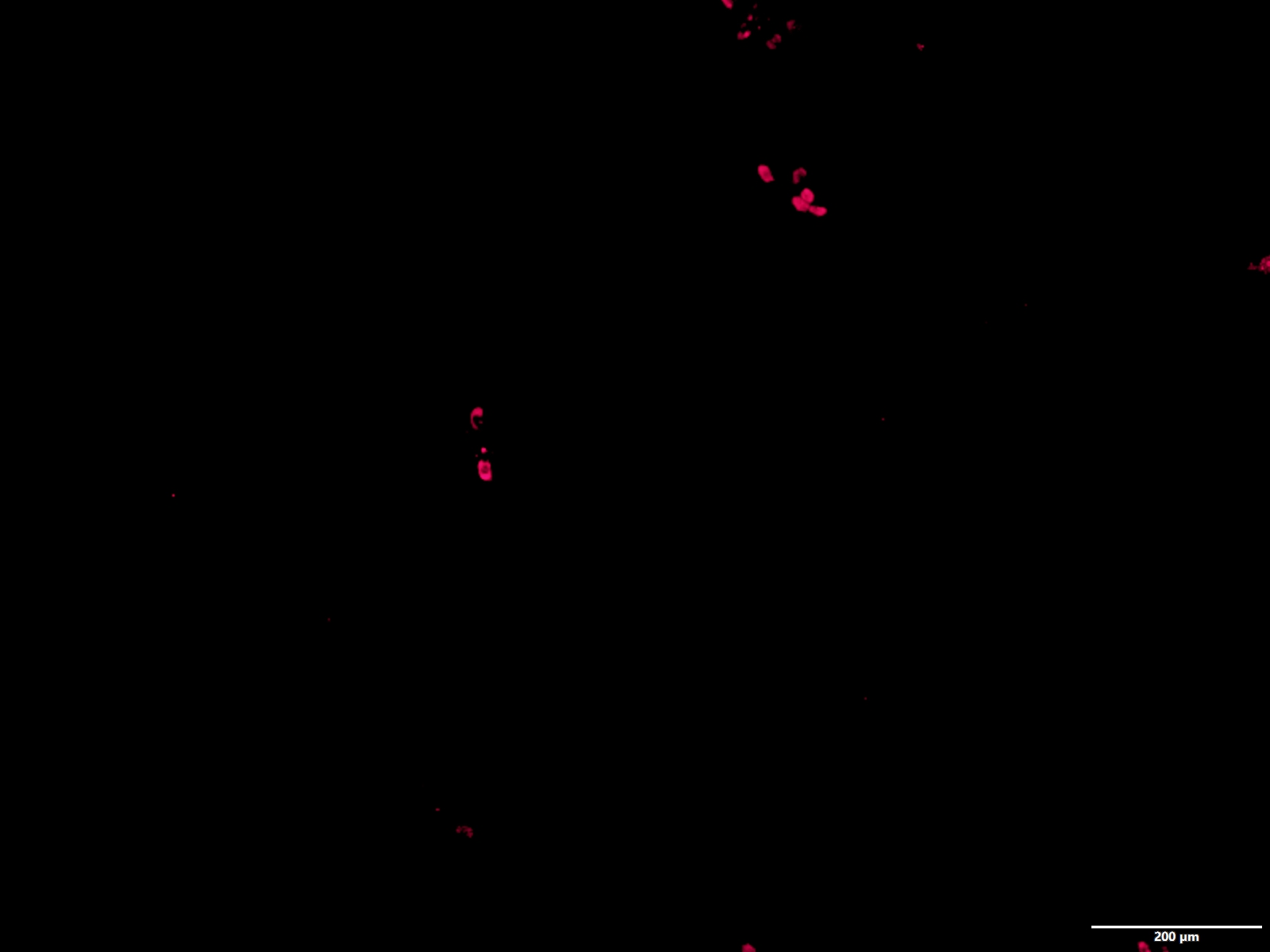

Supplement: S3 Data — This compressed folder contains the underlying numerical data and/or uncropped images used to generate the panels in Figs 3I and 4. (ZIP) [file pbio.3003736.s017.zip › S3 Data/Figure4/H/KO+CHO/22-5+dan--tgev-n-2.jpg]

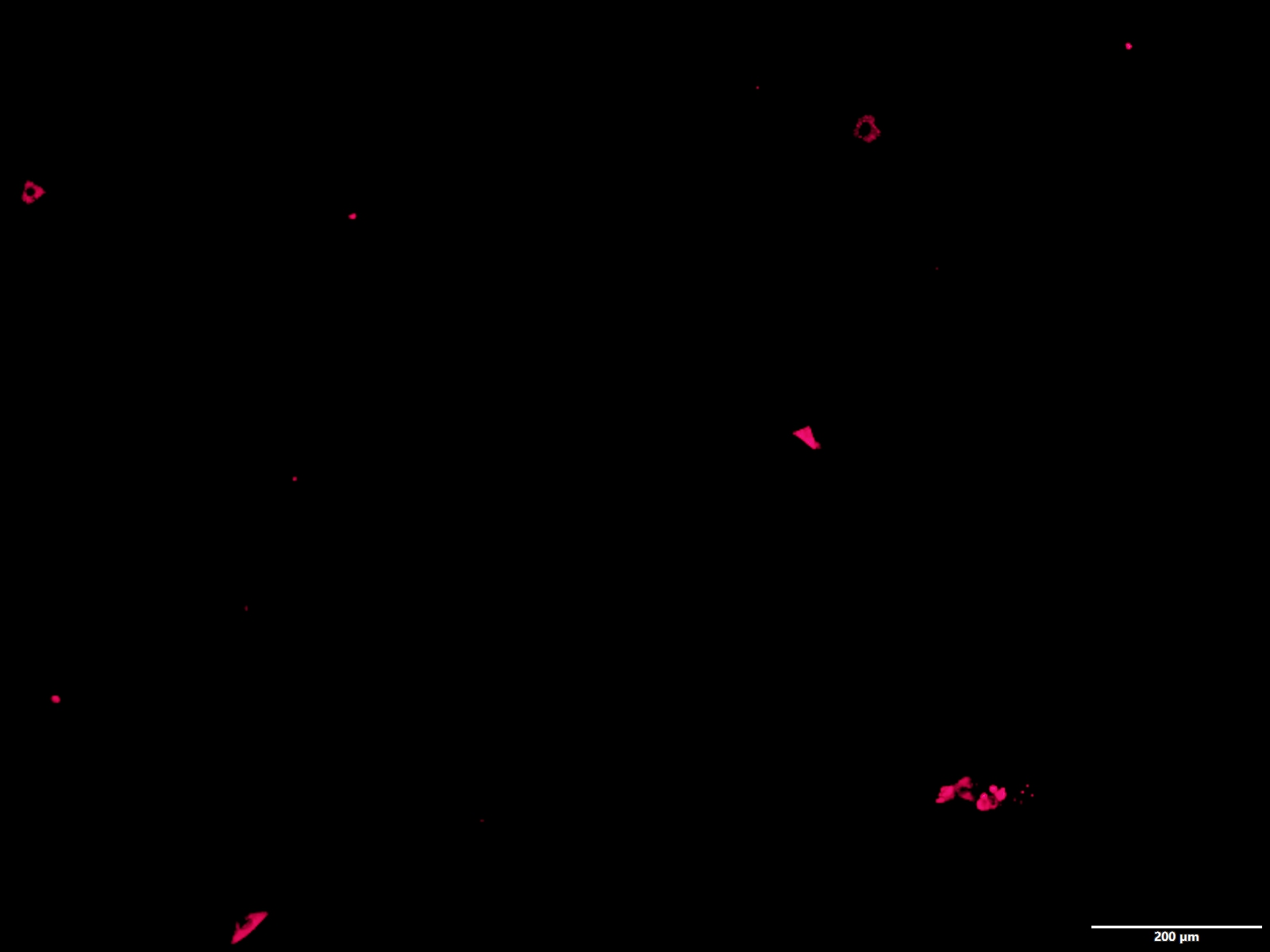

Supplement: S3 Data — This compressed folder contains the underlying numerical data and/or uncropped images used to generate the panels in Figs 3I and 4. (ZIP) [file pbio.3003736.s017.zip › S3 Data/Figure4/H/KO+CHO/22-5+dan--tgev-n-3.jpg]

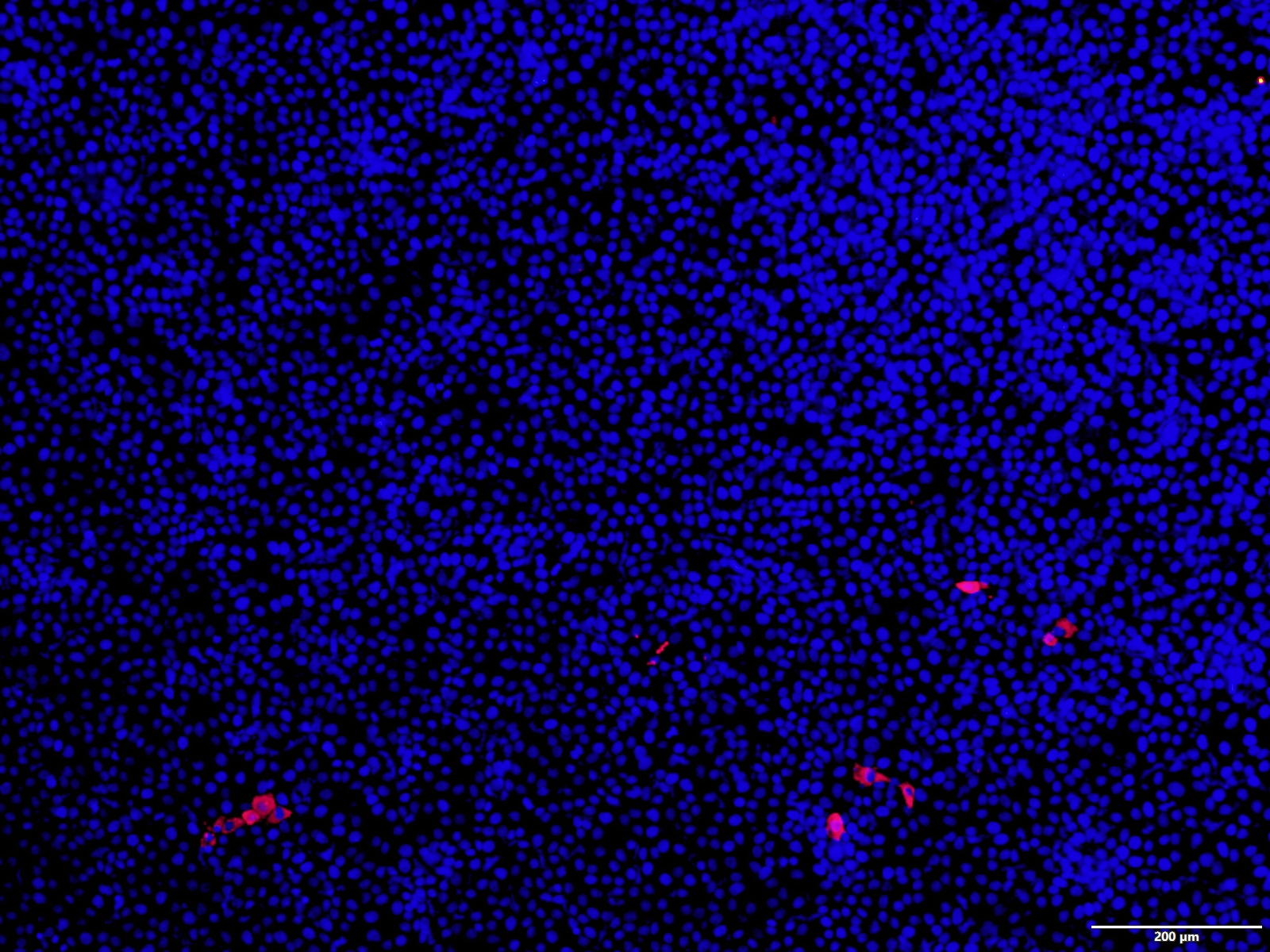

Supplement: S3 Data — This compressed folder contains the underlying numerical data and/or uncropped images used to generate the panels in Figs 3I and 4. (ZIP) [file pbio.3003736.s017.zip › S3 Data/Figure4/H/KO+CHO/22-5+dan-1.jpg]

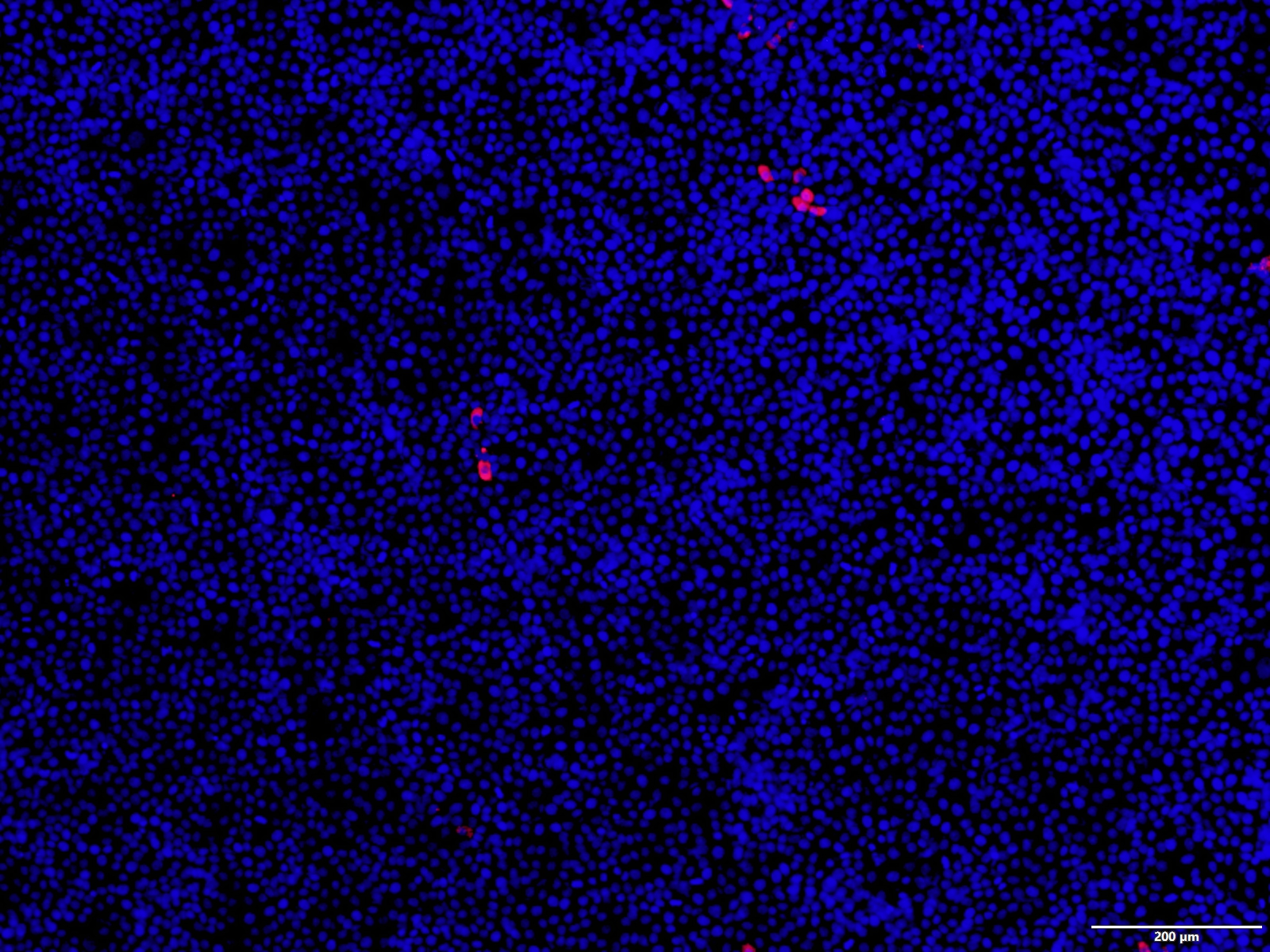

Supplement: S3 Data — This compressed folder contains the underlying numerical data and/or uncropped images used to generate the panels in Figs 3I and 4. (ZIP) [file pbio.3003736.s017.zip › S3 Data/Figure4/H/KO+CHO/22-5+dan-2.jpg]

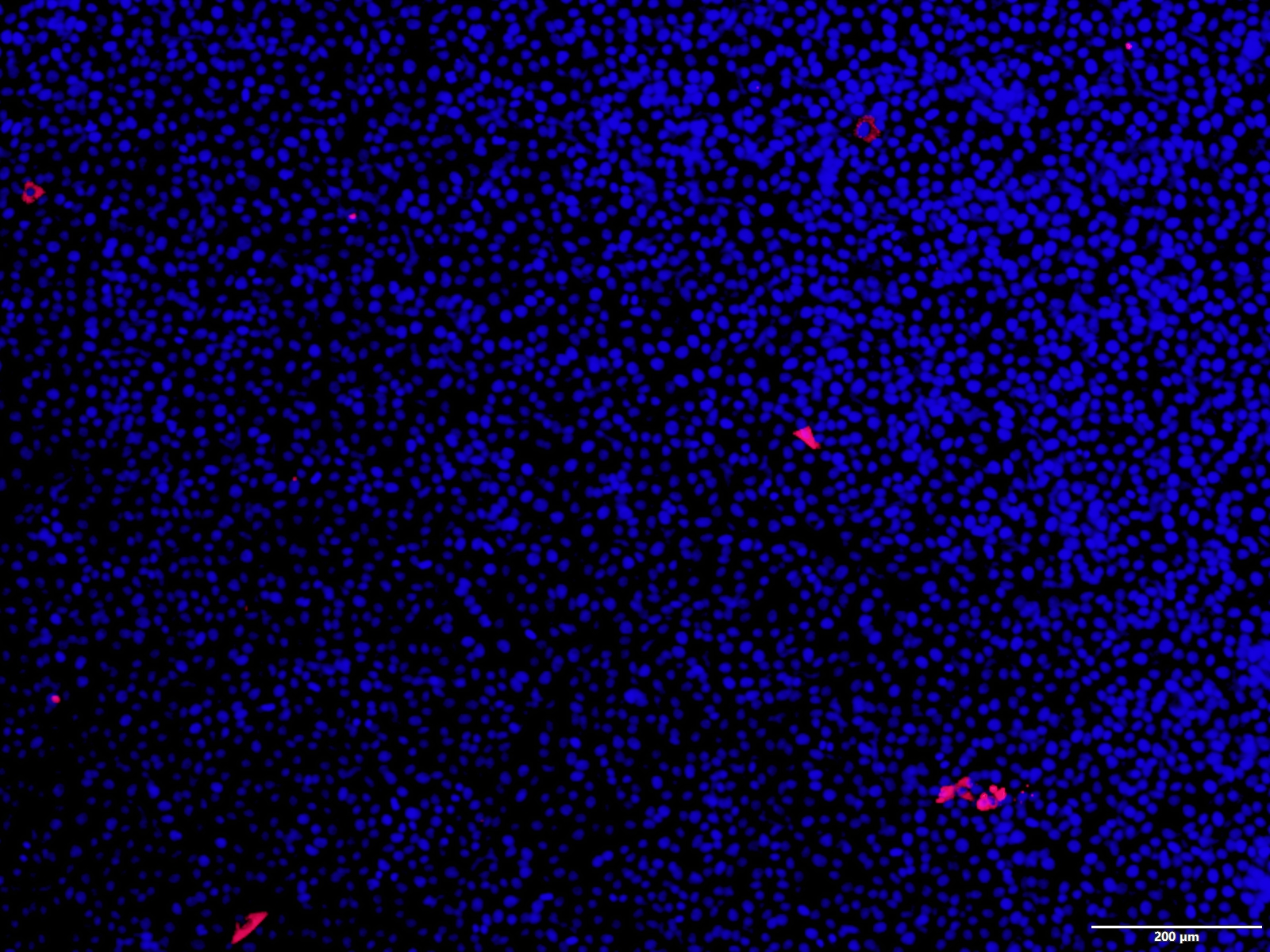

Supplement: S3 Data — This compressed folder contains the underlying numerical data and/or uncropped images used to generate the panels in Figs 3I and 4. (ZIP) [file pbio.3003736.s017.zip › S3 Data/Figure4/H/KO+CHO/22-5+dan-3.jpg]

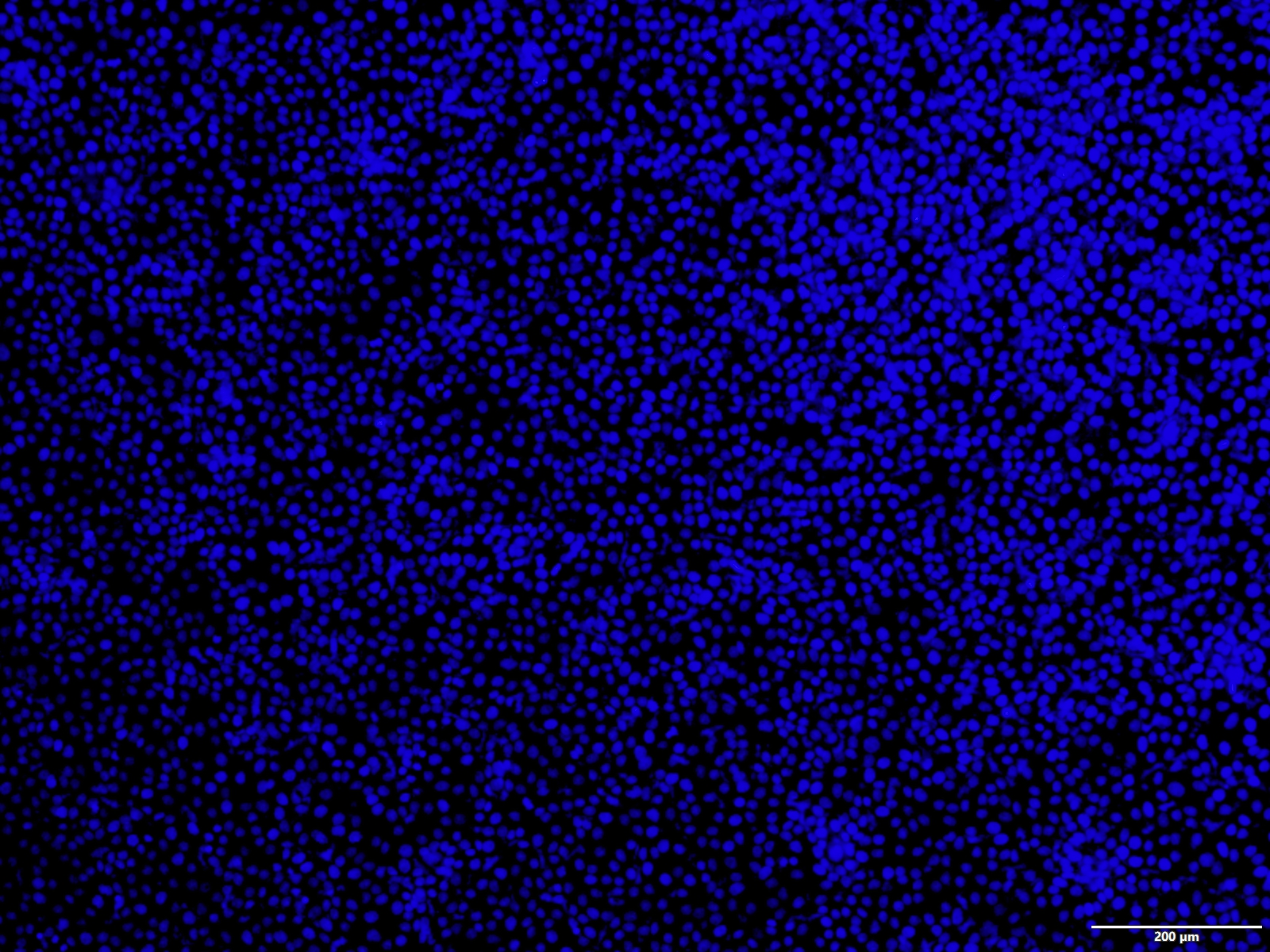

Supplement: S3 Data — This compressed folder contains the underlying numerical data and/or uncropped images used to generate the panels in Figs 3I and 4. (ZIP) [file pbio.3003736.s017.zip › S3 Data/Figure4/H/KO+CHO/22-5+dan-dapi-1.jpg]

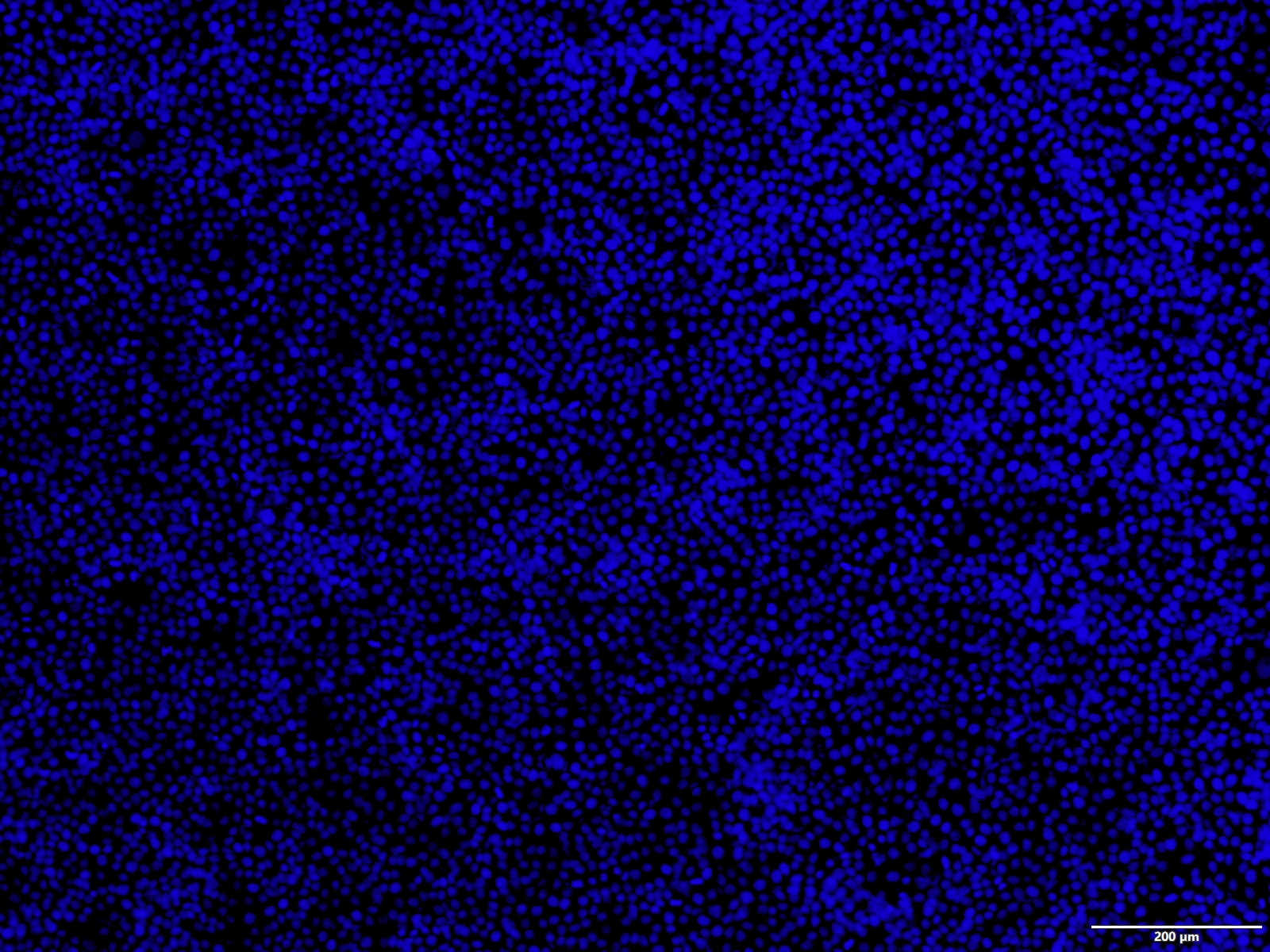

Supplement: S3 Data — This compressed folder contains the underlying numerical data and/or uncropped images used to generate the panels in Figs 3I and 4. (ZIP) [file pbio.3003736.s017.zip › S3 Data/Figure4/H/KO+CHO/22-5+dan-dapi-2.jpg]

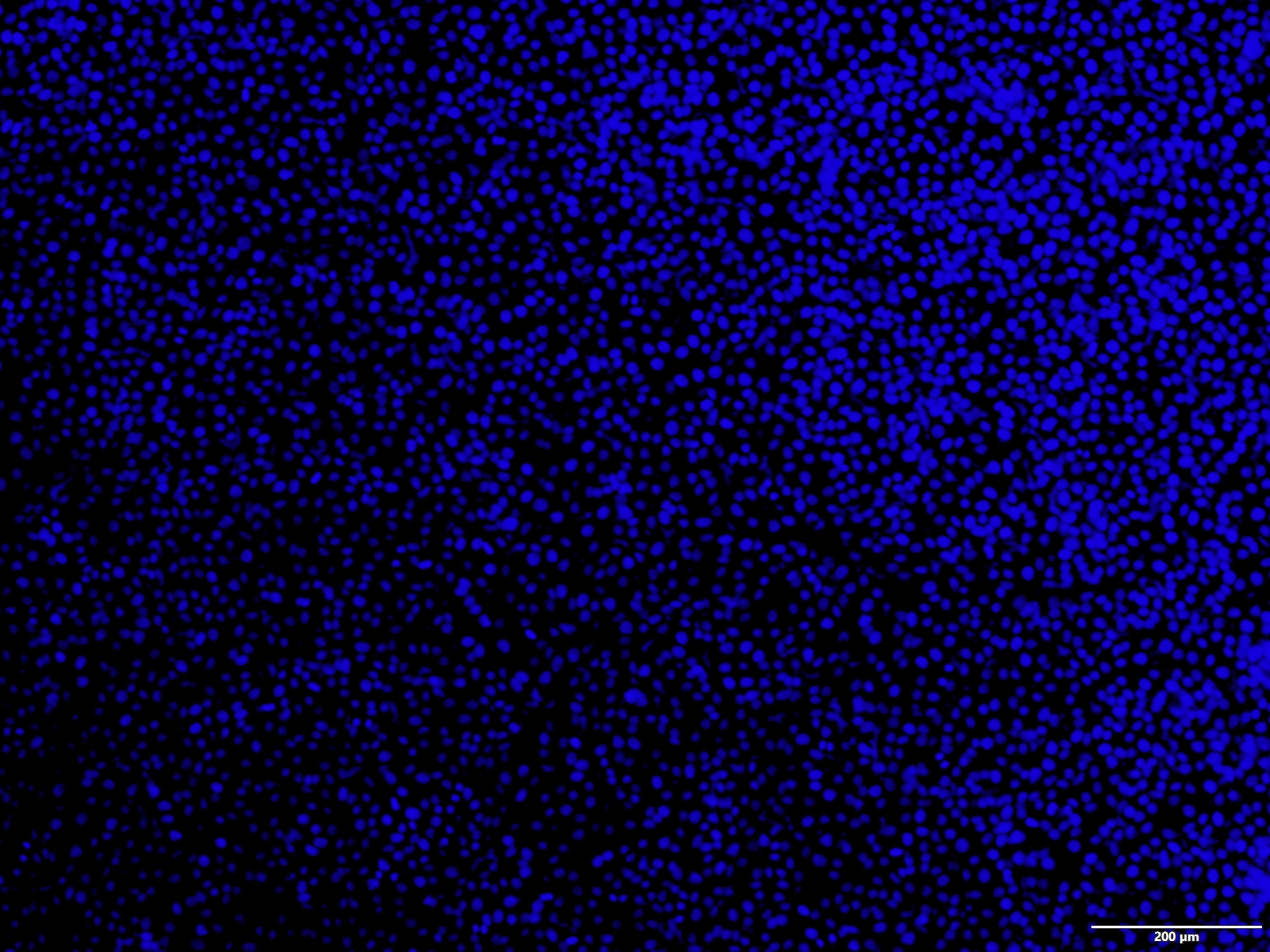

Supplement: S3 Data — This compressed folder contains the underlying numerical data and/or uncropped images used to generate the panels in Figs 3I and 4. (ZIP) [file pbio.3003736.s017.zip › S3 Data/Figure4/H/KO+CHO/22-5+dan-dapi-3.jpg]

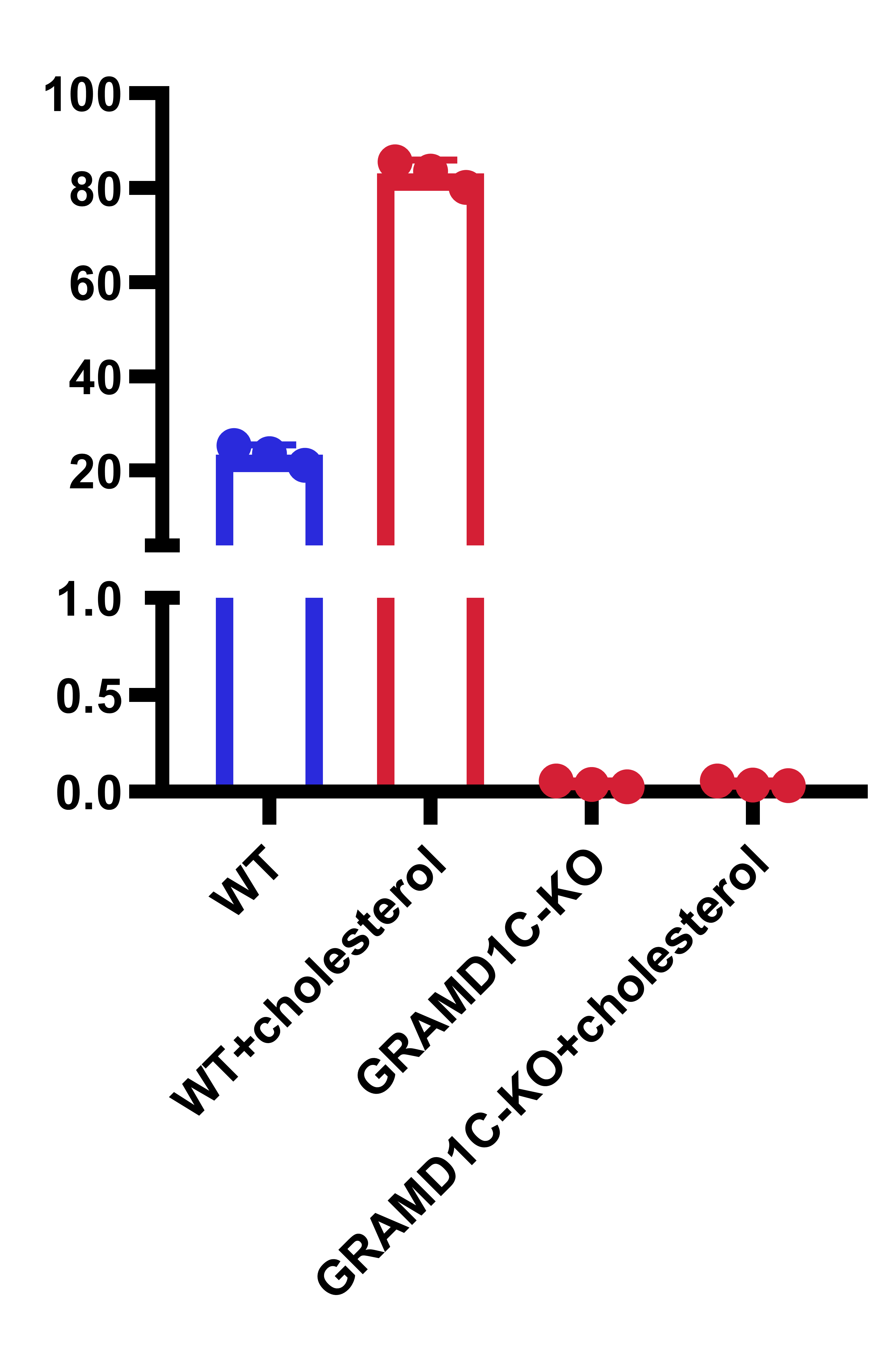

Supplement: S3 Data — This compressed folder contains the underlying numerical data and/or uncropped images used to generate the panels in Figs 3I and 4. (ZIP) [file pbio.3003736.s017.zip › S3 Data/Figure4/H/positive-cells.tif]

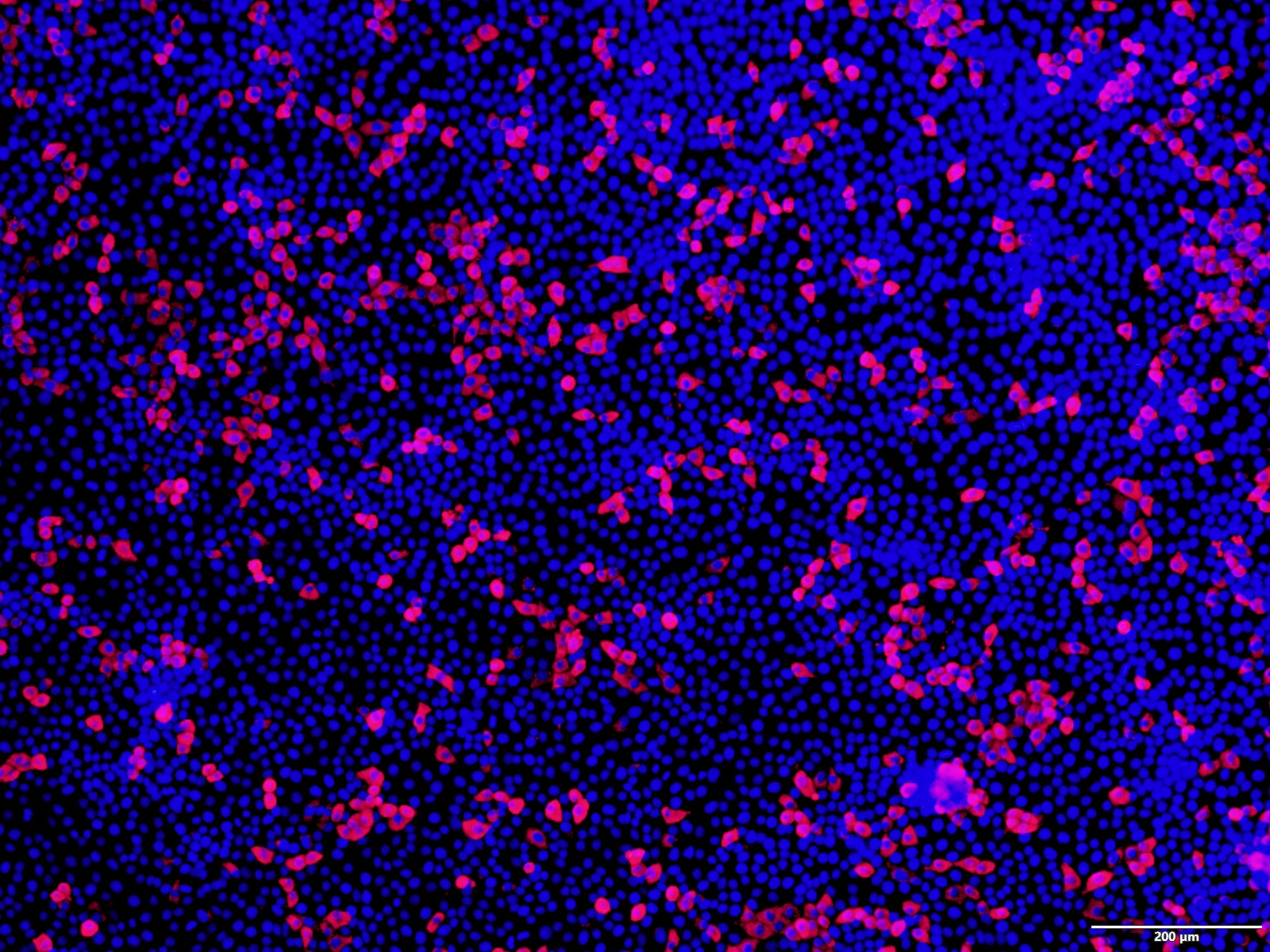

Supplement: S3 Data — This compressed folder contains the underlying numerical data and/or uncropped images used to generate the panels in Figs 3I and 4. (ZIP) [file pbio.3003736.s017.zip › S3 Data/Figure4/H/WT/pk-1.jpg]

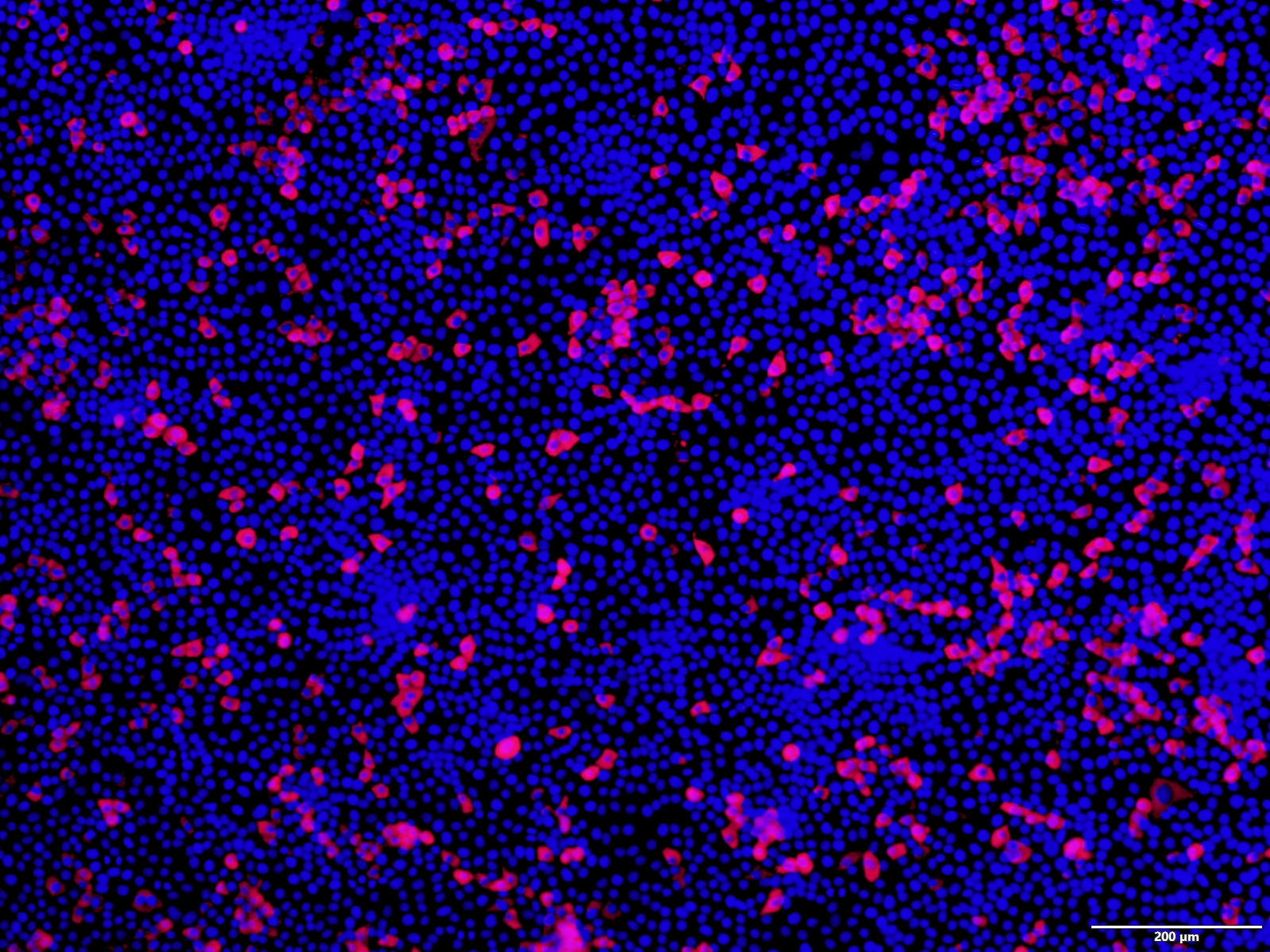

Supplement: S3 Data — This compressed folder contains the underlying numerical data and/or uncropped images used to generate the panels in Figs 3I and 4. (ZIP) [file pbio.3003736.s017.zip › S3 Data/Figure4/H/WT/pk-2.jpg]

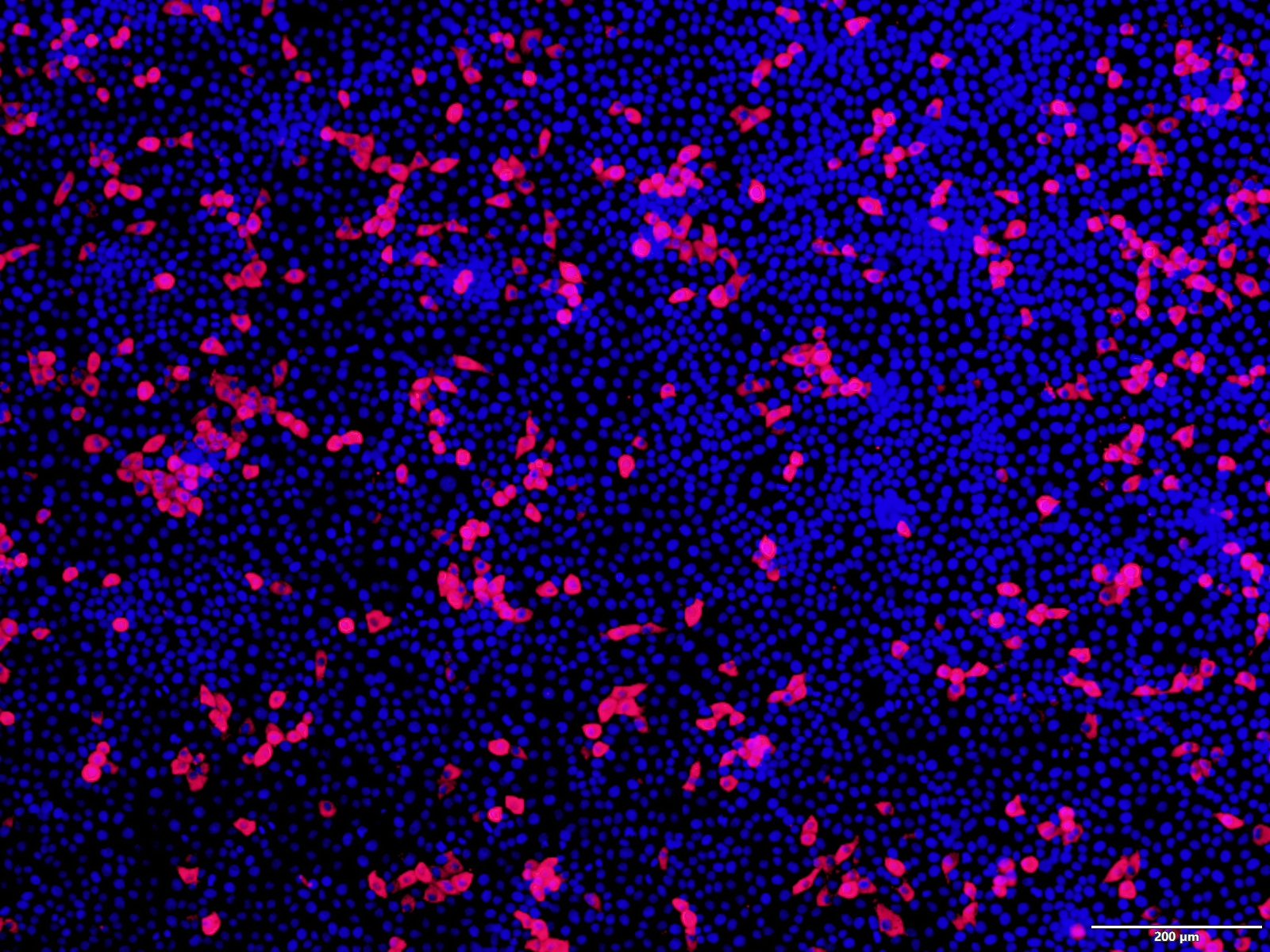

Supplement: S3 Data — This compressed folder contains the underlying numerical data and/or uncropped images used to generate the panels in Figs 3I and 4. (ZIP) [file pbio.3003736.s017.zip › S3 Data/Figure4/H/WT/pk-3.jpg]

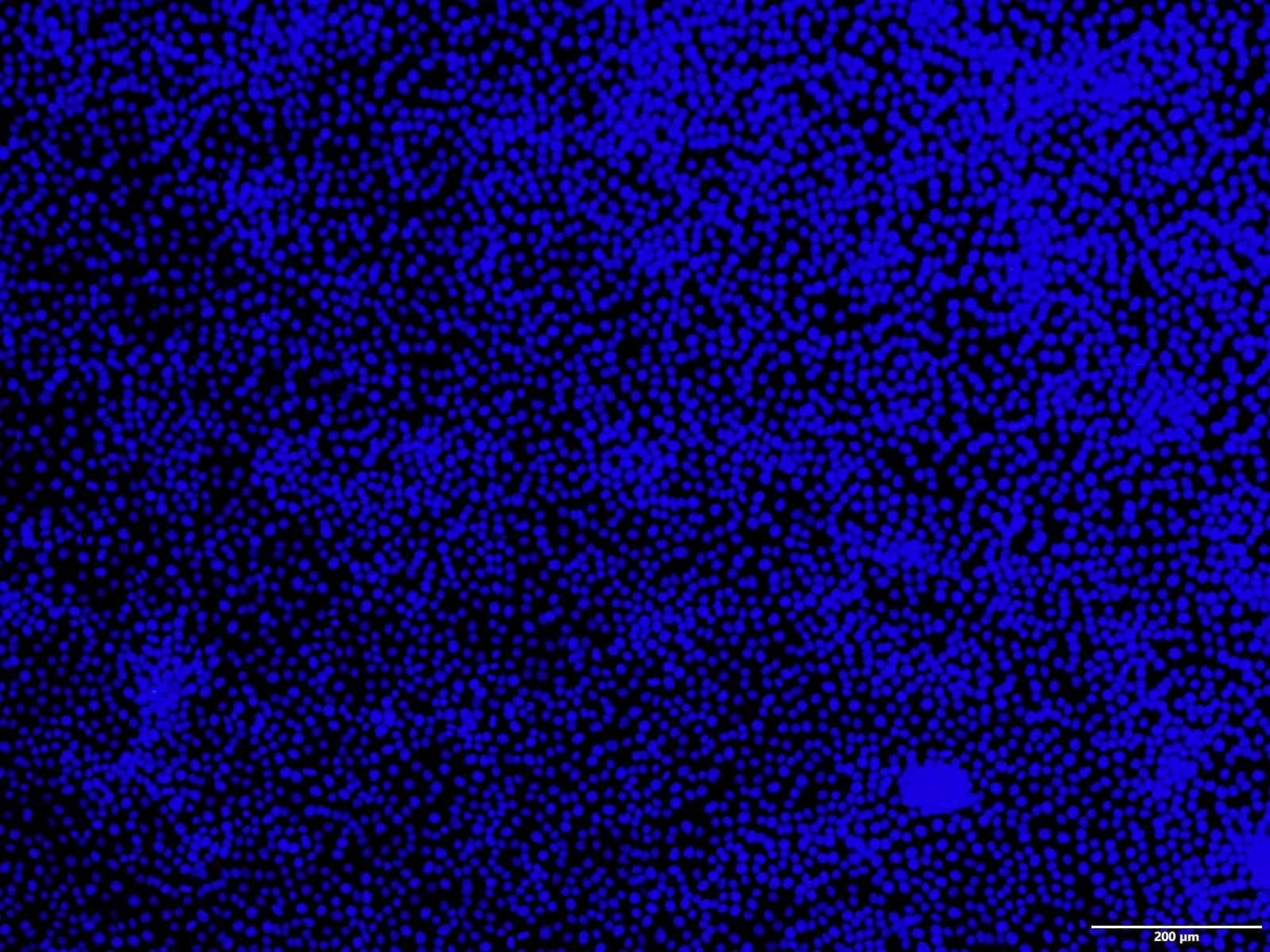

Supplement: S3 Data — This compressed folder contains the underlying numerical data and/or uncropped images used to generate the panels in Figs 3I and 4. (ZIP) [file pbio.3003736.s017.zip › S3 Data/Figure4/H/WT/pk-dapi-1.jpg]

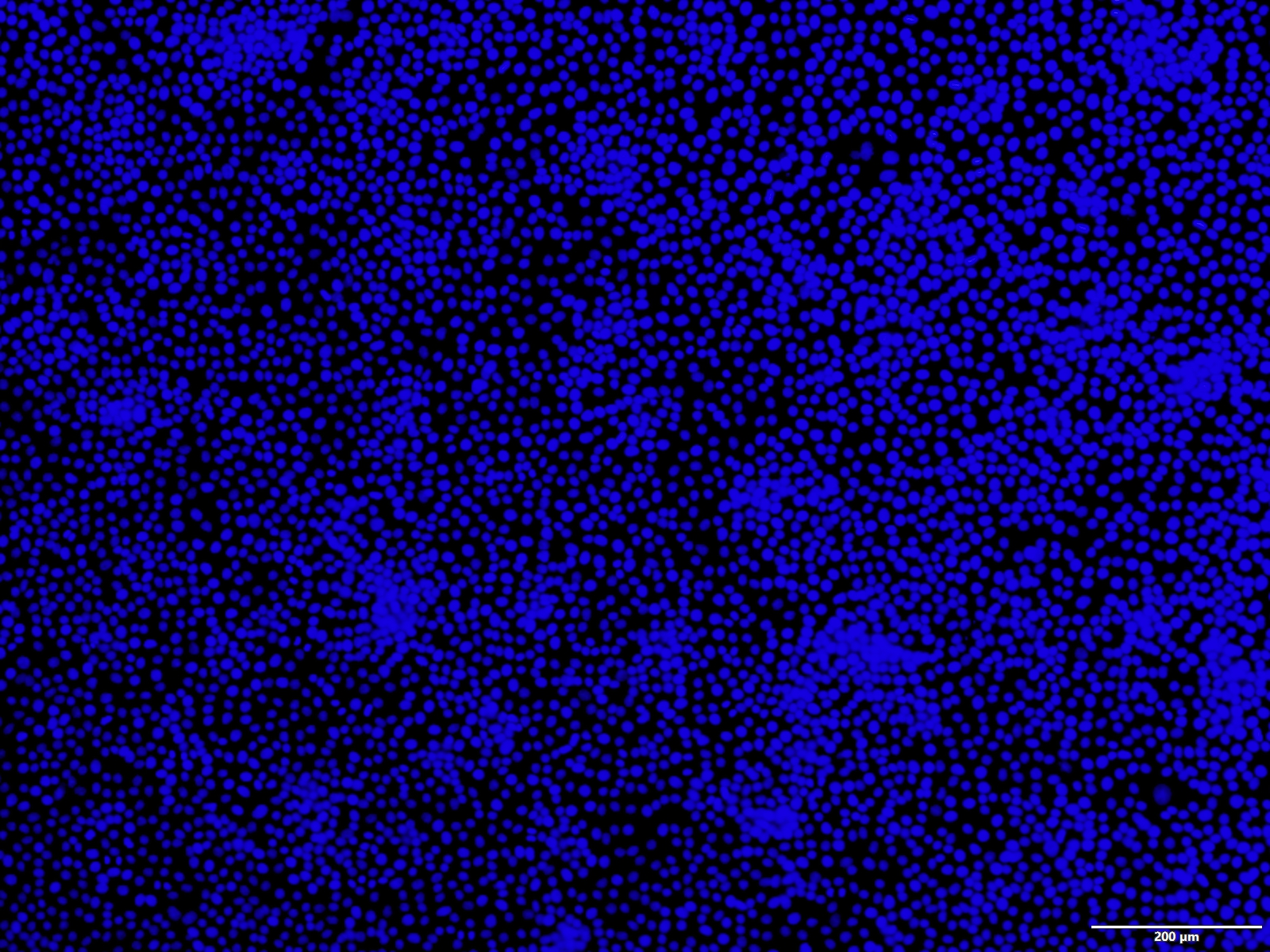

Supplement: S3 Data — This compressed folder contains the underlying numerical data and/or uncropped images used to generate the panels in Figs 3I and 4. (ZIP) [file pbio.3003736.s017.zip › S3 Data/Figure4/H/WT/pk-dapi-2.jpg]

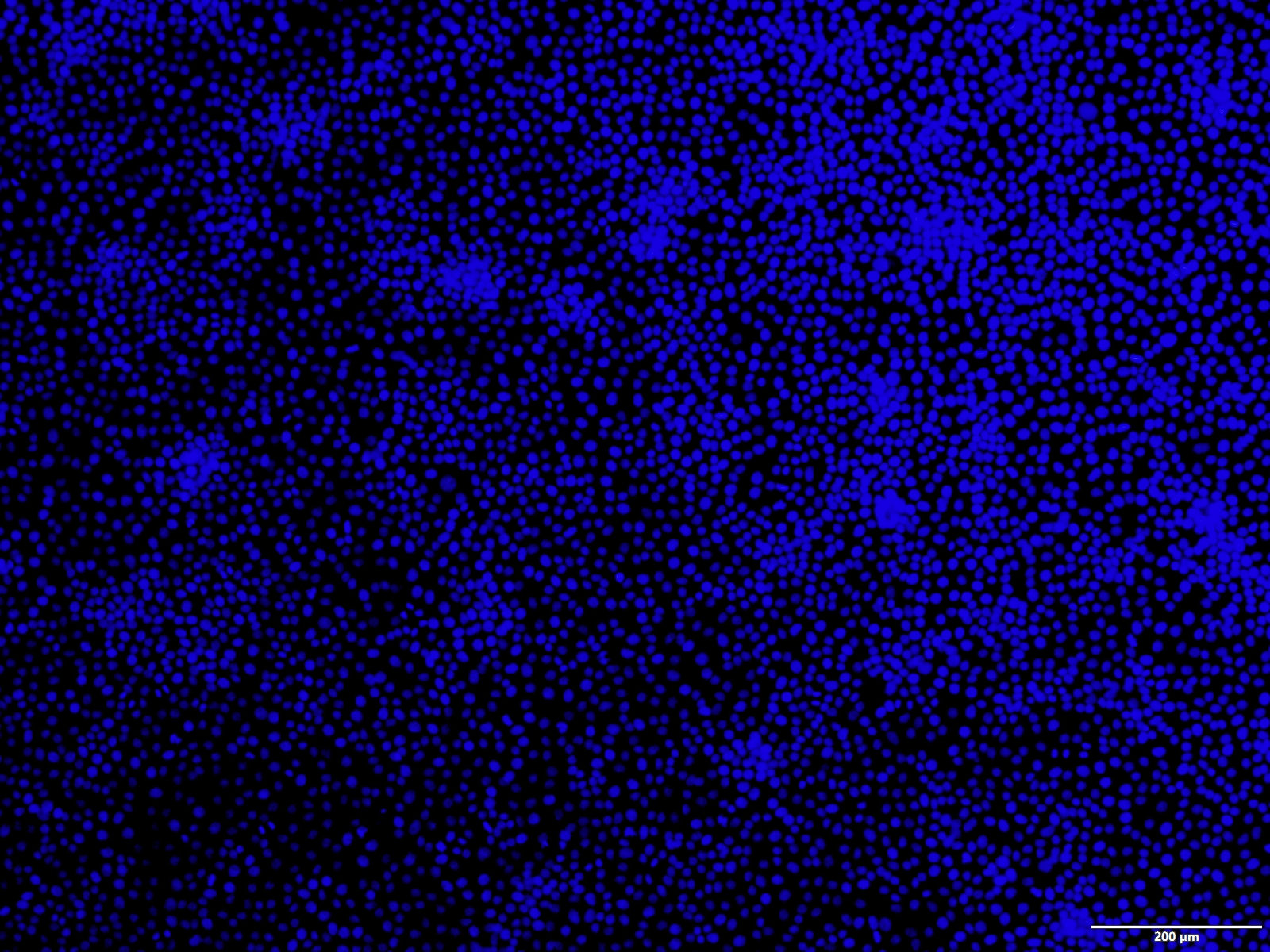

Supplement: S3 Data — This compressed folder contains the underlying numerical data and/or uncropped images used to generate the panels in Figs 3I and 4. (ZIP) [file pbio.3003736.s017.zip › S3 Data/Figure4/H/WT/pk-dapi-3.jpg]

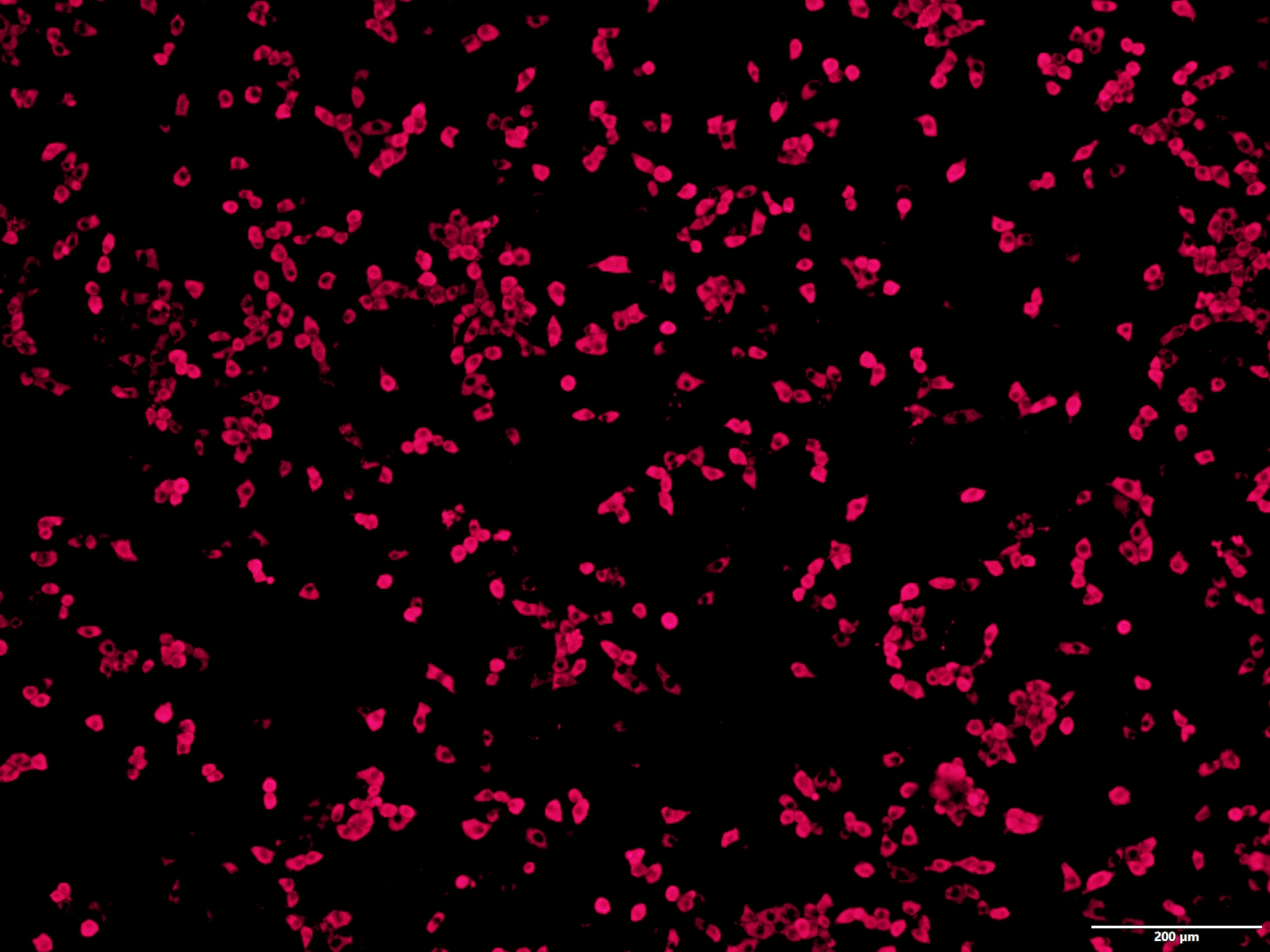

Supplement: S3 Data — This compressed folder contains the underlying numerical data and/or uncropped images used to generate the panels in Figs 3I and 4. (ZIP) [file pbio.3003736.s017.zip › S3 Data/Figure4/H/WT/pk-tgev-n-1.jpg]

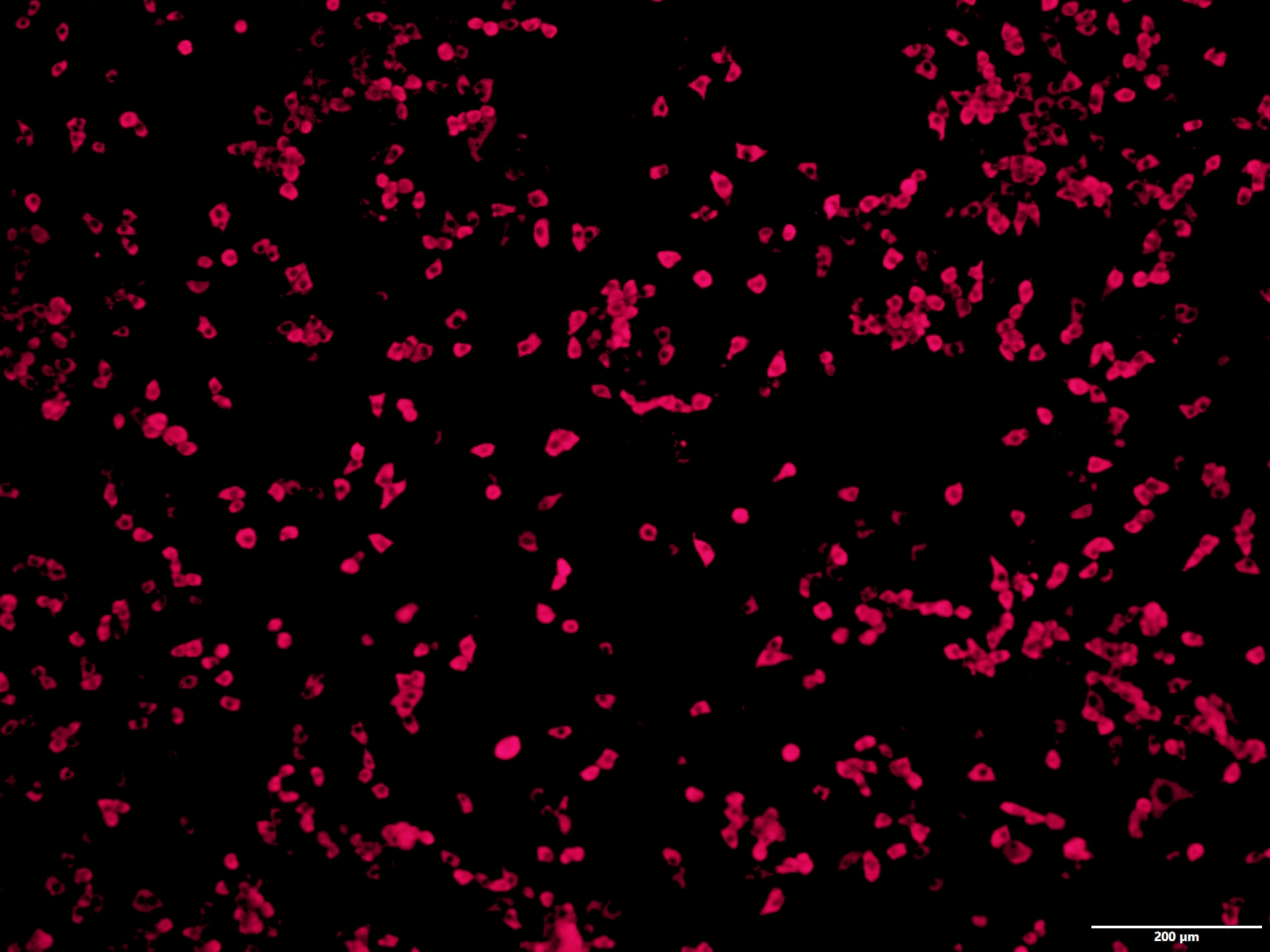

Supplement: S3 Data — This compressed folder contains the underlying numerical data and/or uncropped images used to generate the panels in Figs 3I and 4. (ZIP) [file pbio.3003736.s017.zip › S3 Data/Figure4/H/WT/pk-tgev-n-2.jpg]

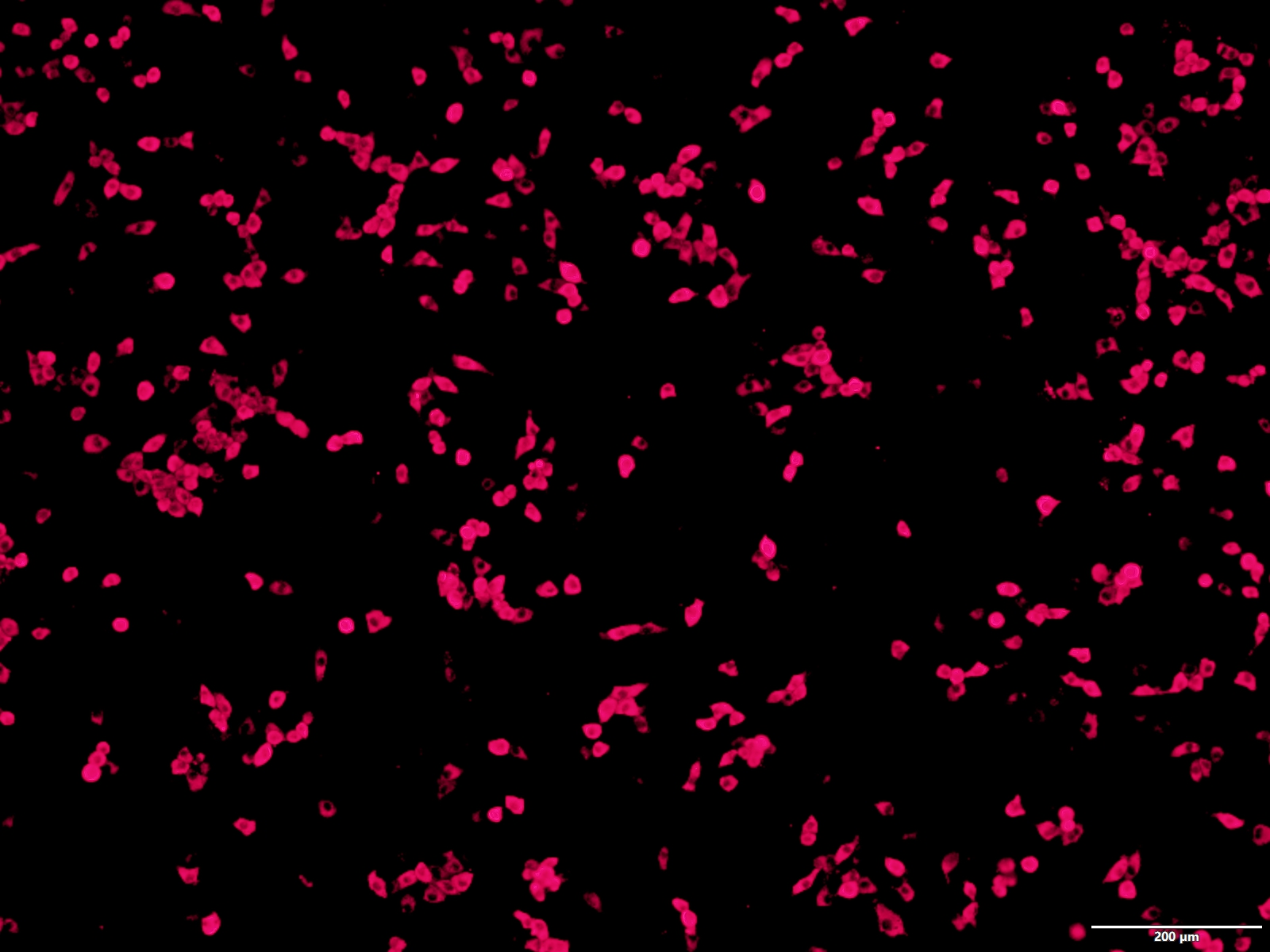

Supplement: S3 Data — This compressed folder contains the underlying numerical data and/or uncropped images used to generate the panels in Figs 3I and 4. (ZIP) [file pbio.3003736.s017.zip › S3 Data/Figure4/H/WT/pk-tgev-n-3.jpg]

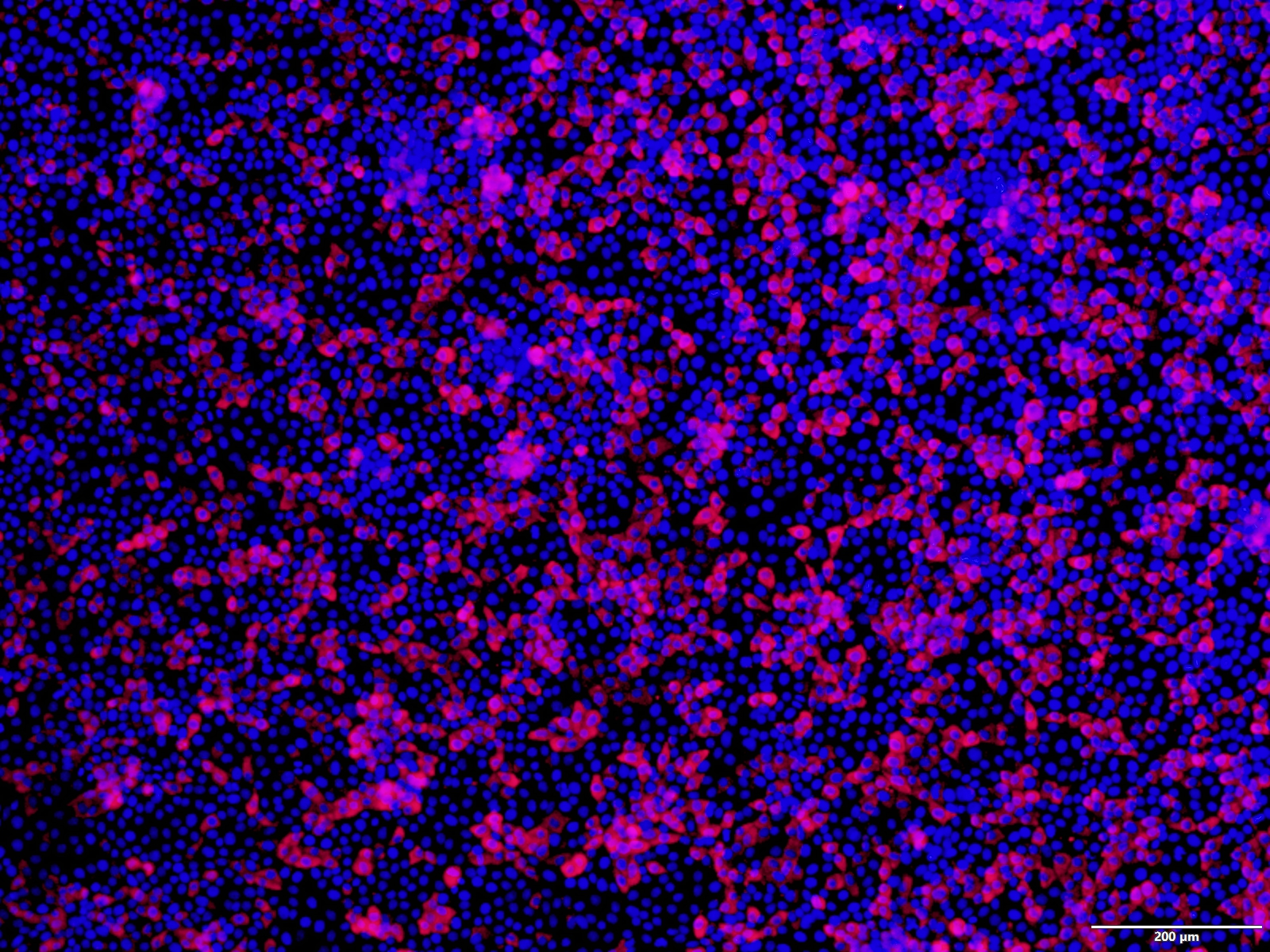

Supplement: S3 Data — This compressed folder contains the underlying numerical data and/or uncropped images used to generate the panels in Figs 3I and 4. (ZIP) [file pbio.3003736.s017.zip › S3 Data/Figure4/H/WT+CHO/pk+dan-1.jpg]

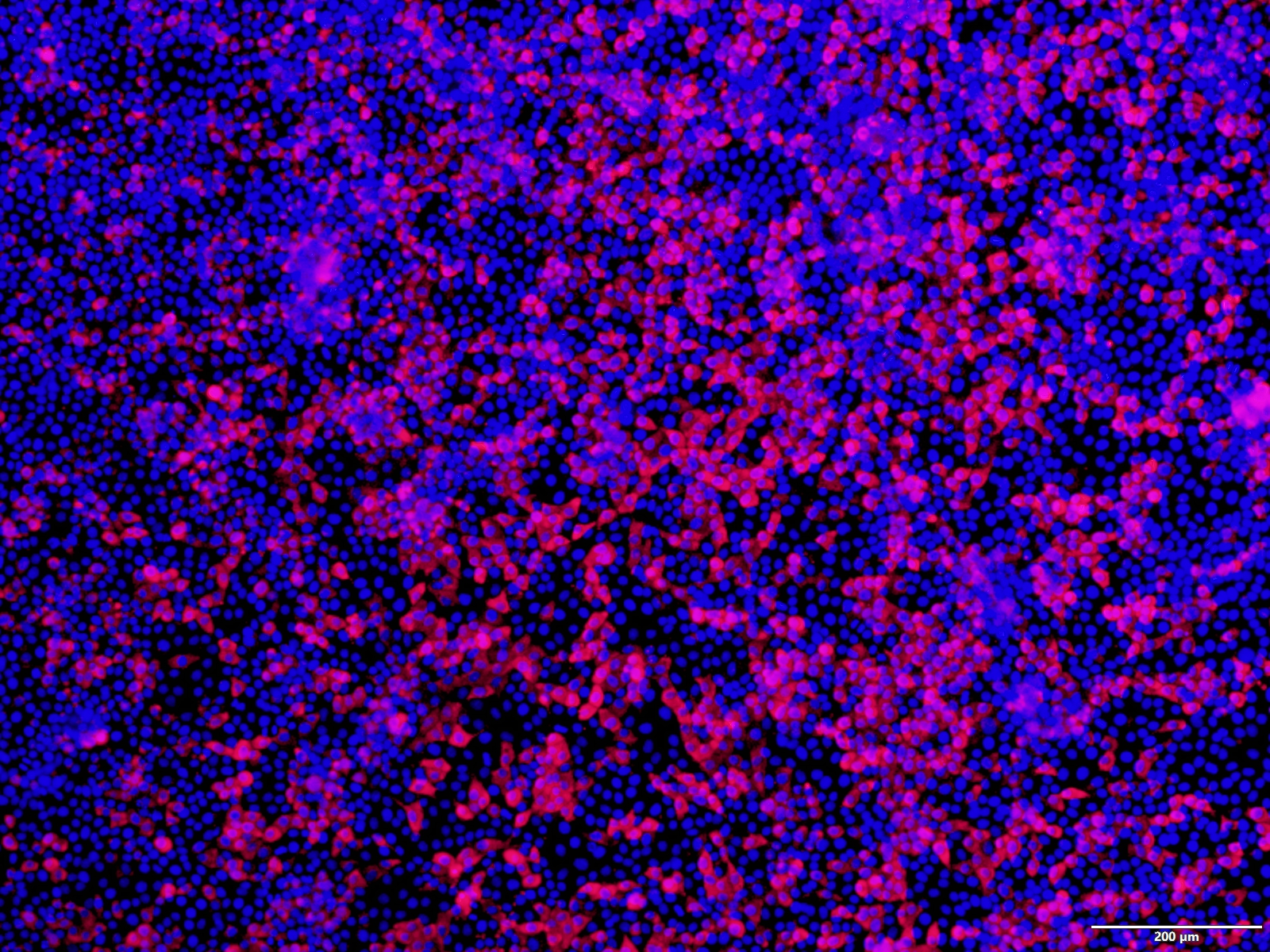

Supplement: S3 Data — This compressed folder contains the underlying numerical data and/or uncropped images used to generate the panels in Figs 3I and 4. (ZIP) [file pbio.3003736.s017.zip › S3 Data/Figure4/H/WT+CHO/pk+dan-2.jpg]

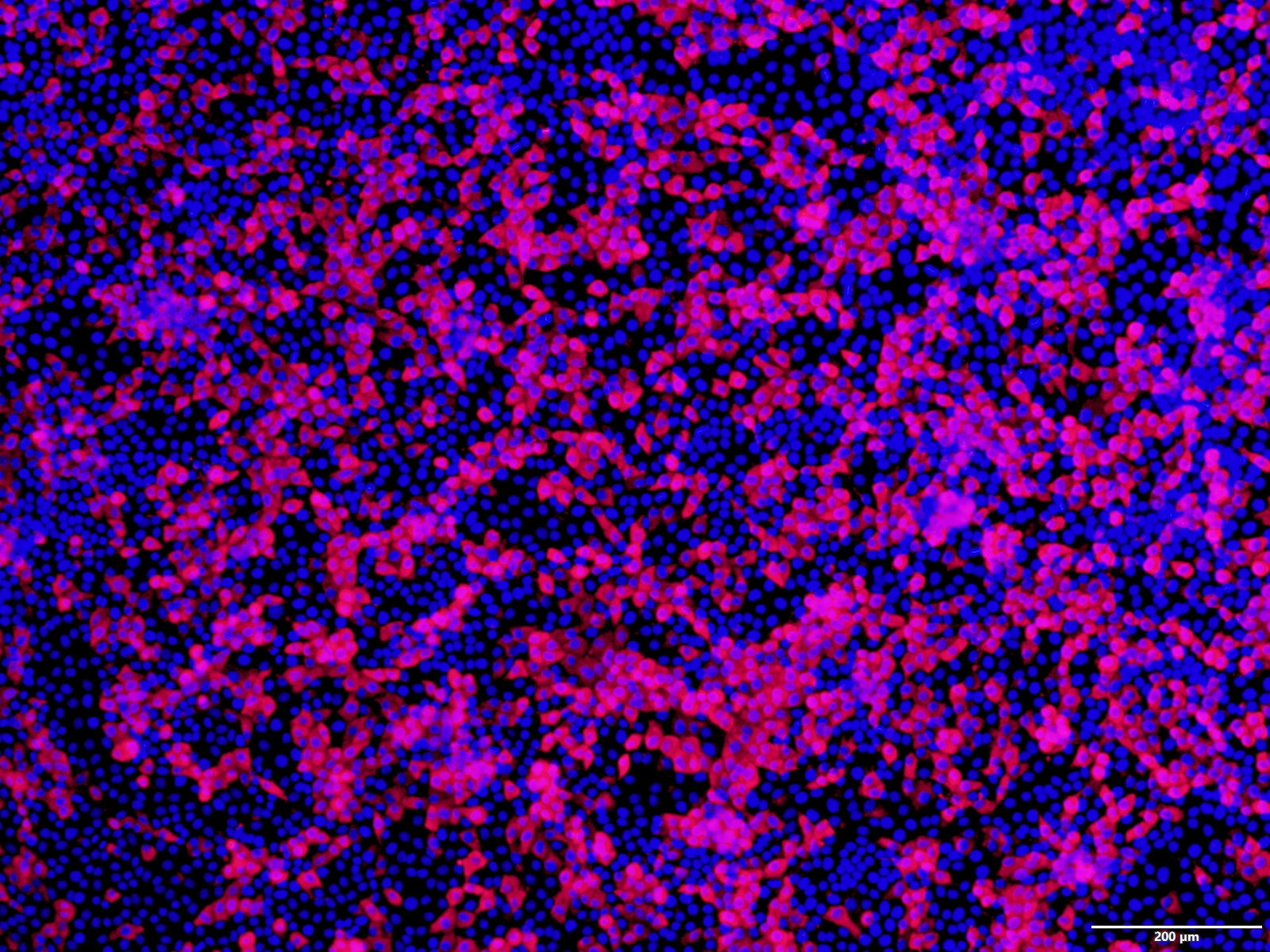

Supplement: S3 Data — This compressed folder contains the underlying numerical data and/or uncropped images used to generate the panels in Figs 3I and 4. (ZIP) [file pbio.3003736.s017.zip › S3 Data/Figure4/H/WT+CHO/pk+dan-3.jpg]

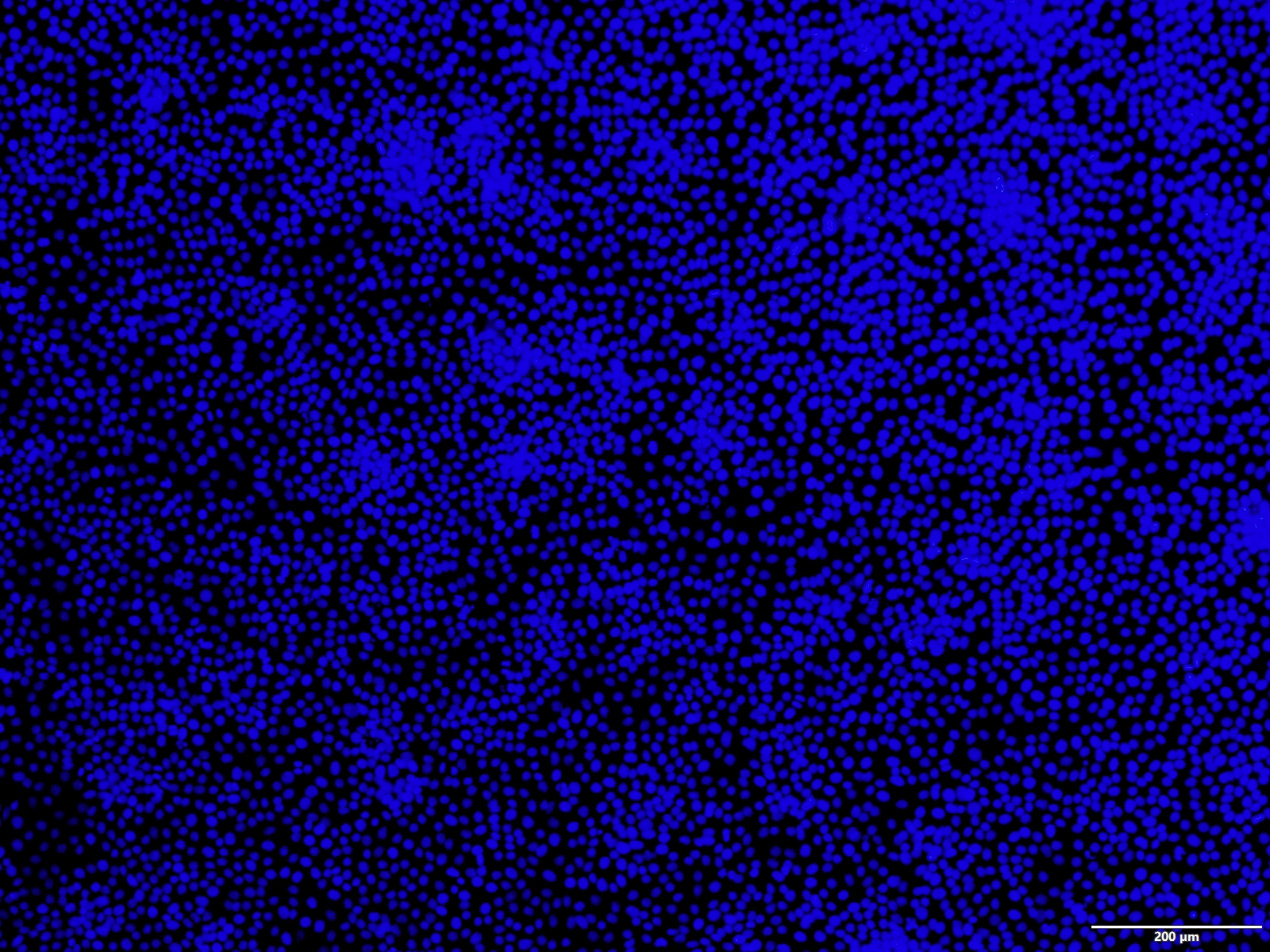

Supplement: S3 Data — This compressed folder contains the underlying numerical data and/or uncropped images used to generate the panels in Figs 3I and 4. (ZIP) [file pbio.3003736.s017.zip › S3 Data/Figure4/H/WT+CHO/pk-+dan-dapi-1.jpg]

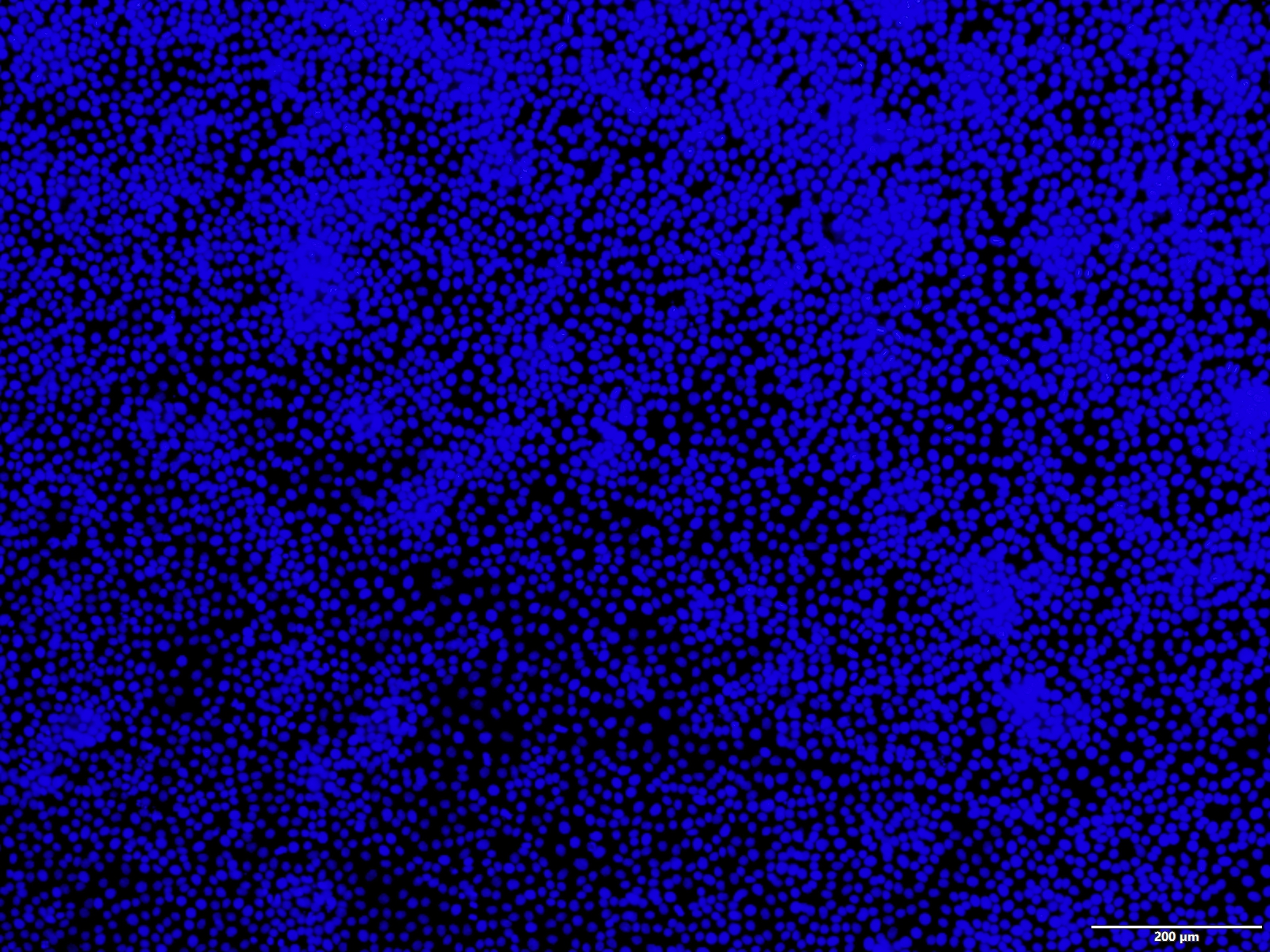

Supplement: S3 Data — This compressed folder contains the underlying numerical data and/or uncropped images used to generate the panels in Figs 3I and 4. (ZIP) [file pbio.3003736.s017.zip › S3 Data/Figure4/H/WT+CHO/pk-+dan-dapi-2.jpg]

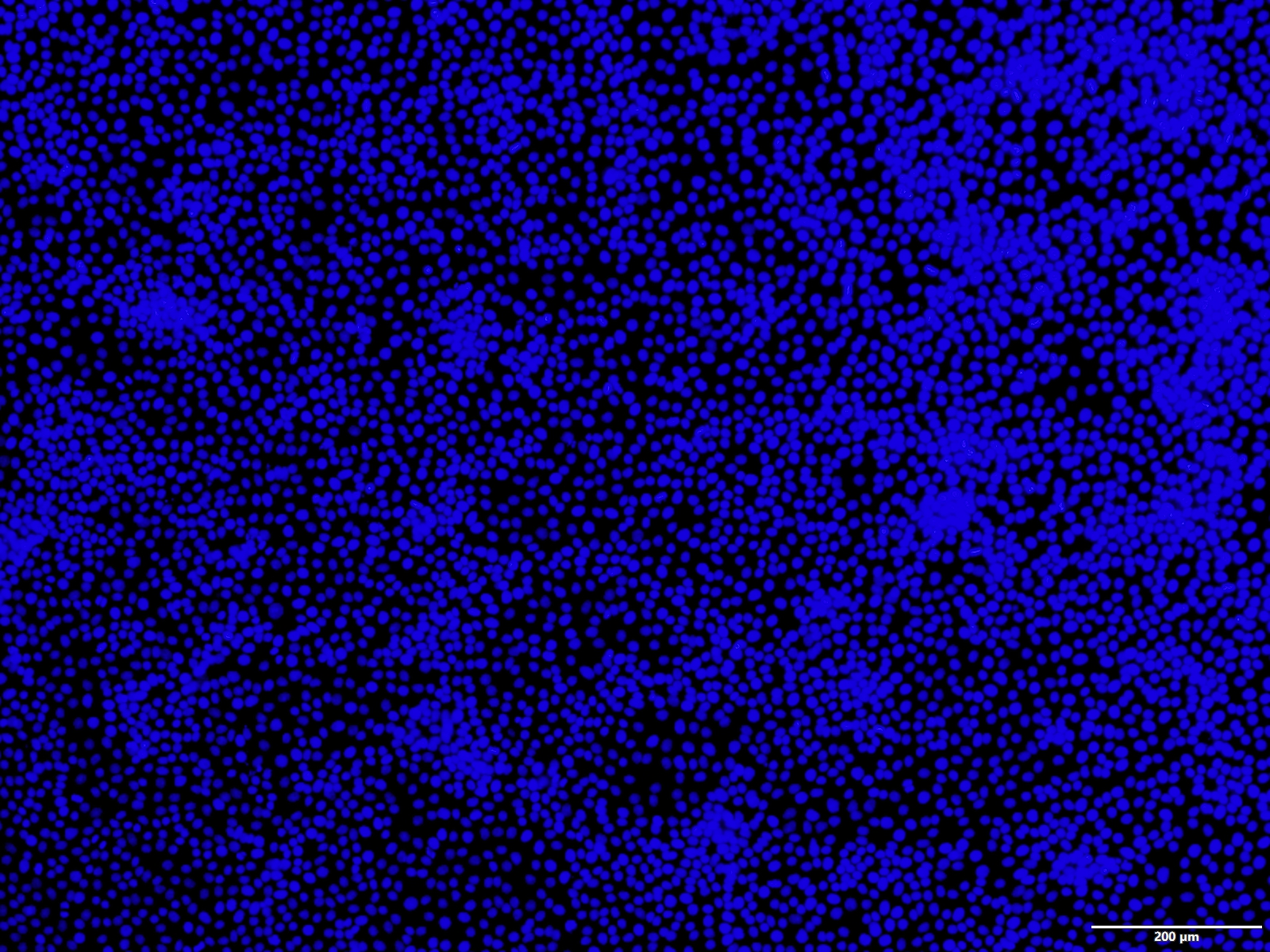

Supplement: S3 Data — This compressed folder contains the underlying numerical data and/or uncropped images used to generate the panels in Figs 3I and 4. (ZIP) [file pbio.3003736.s017.zip › S3 Data/Figure4/H/WT+CHO/pk-+dan-dapi-3.jpg]

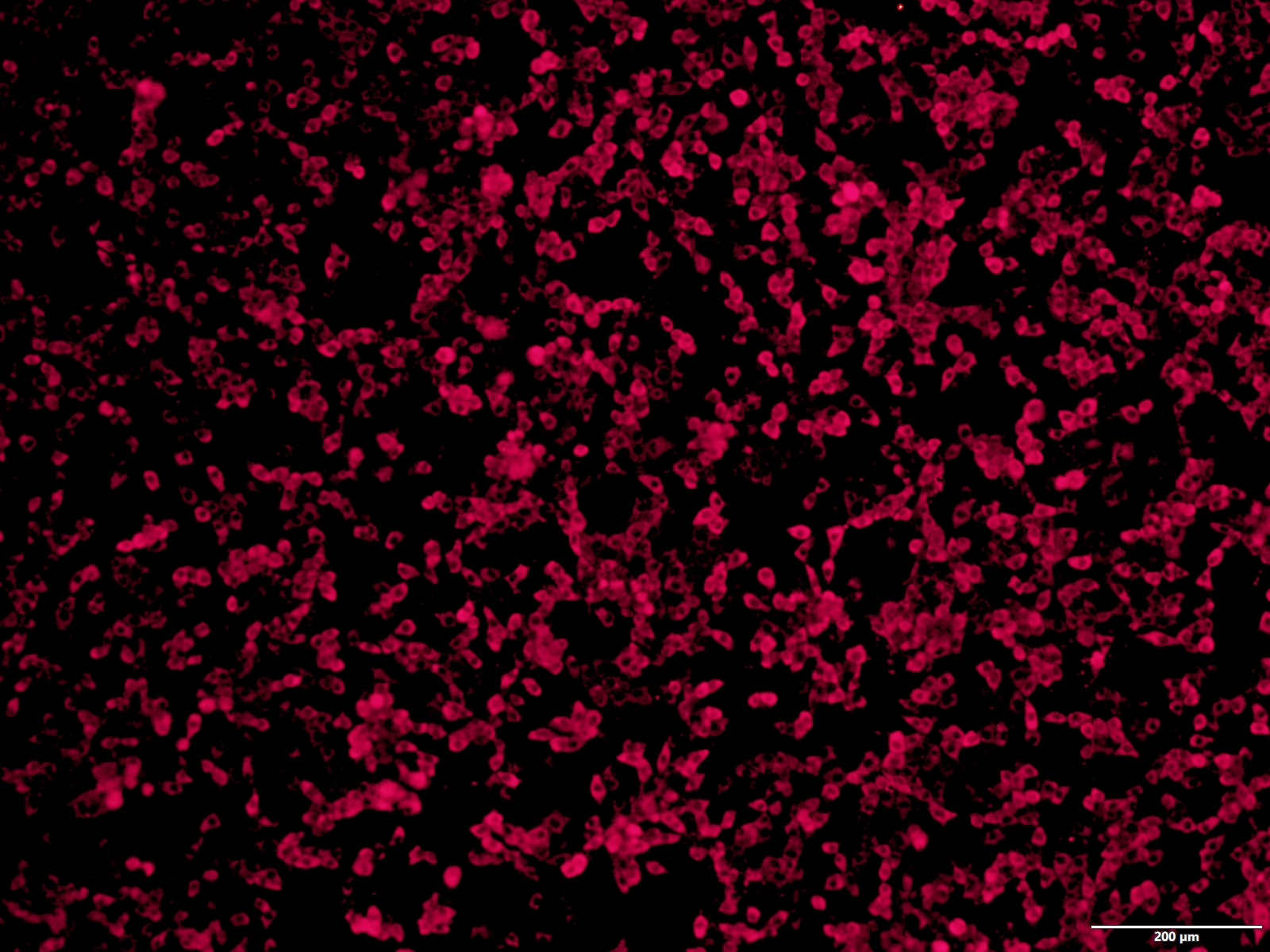

Supplement: S3 Data — This compressed folder contains the underlying numerical data and/or uncropped images used to generate the panels in Figs 3I and 4. (ZIP) [file pbio.3003736.s017.zip › S3 Data/Figure4/H/WT+CHO/pk-+dan-tgev-n-1.jpg]

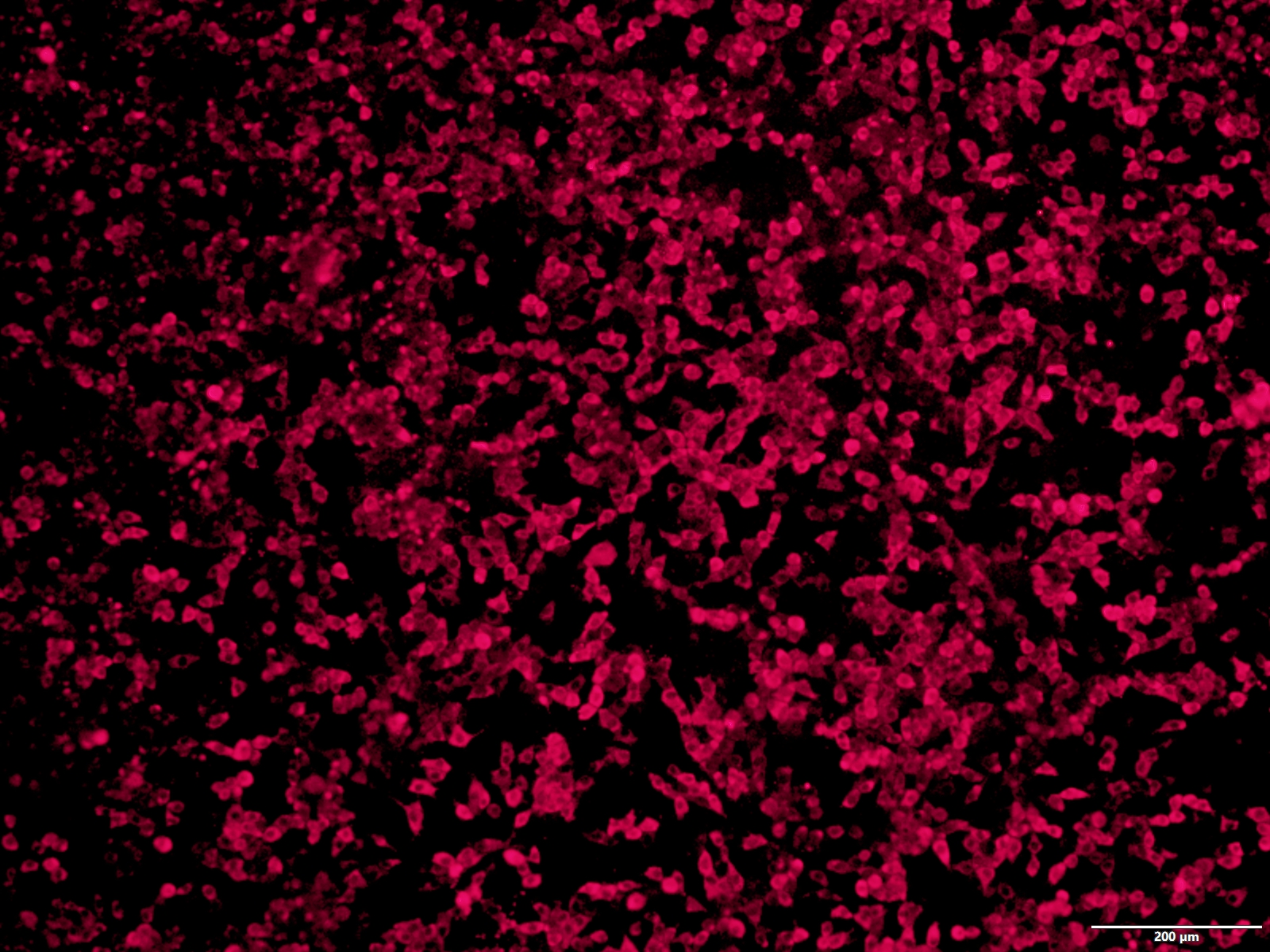

Supplement: S3 Data — This compressed folder contains the underlying numerical data and/or uncropped images used to generate the panels in Figs 3I and 4. (ZIP) [file pbio.3003736.s017.zip › S3 Data/Figure4/H/WT+CHO/pk-+dan-tgev-n-2.jpg]

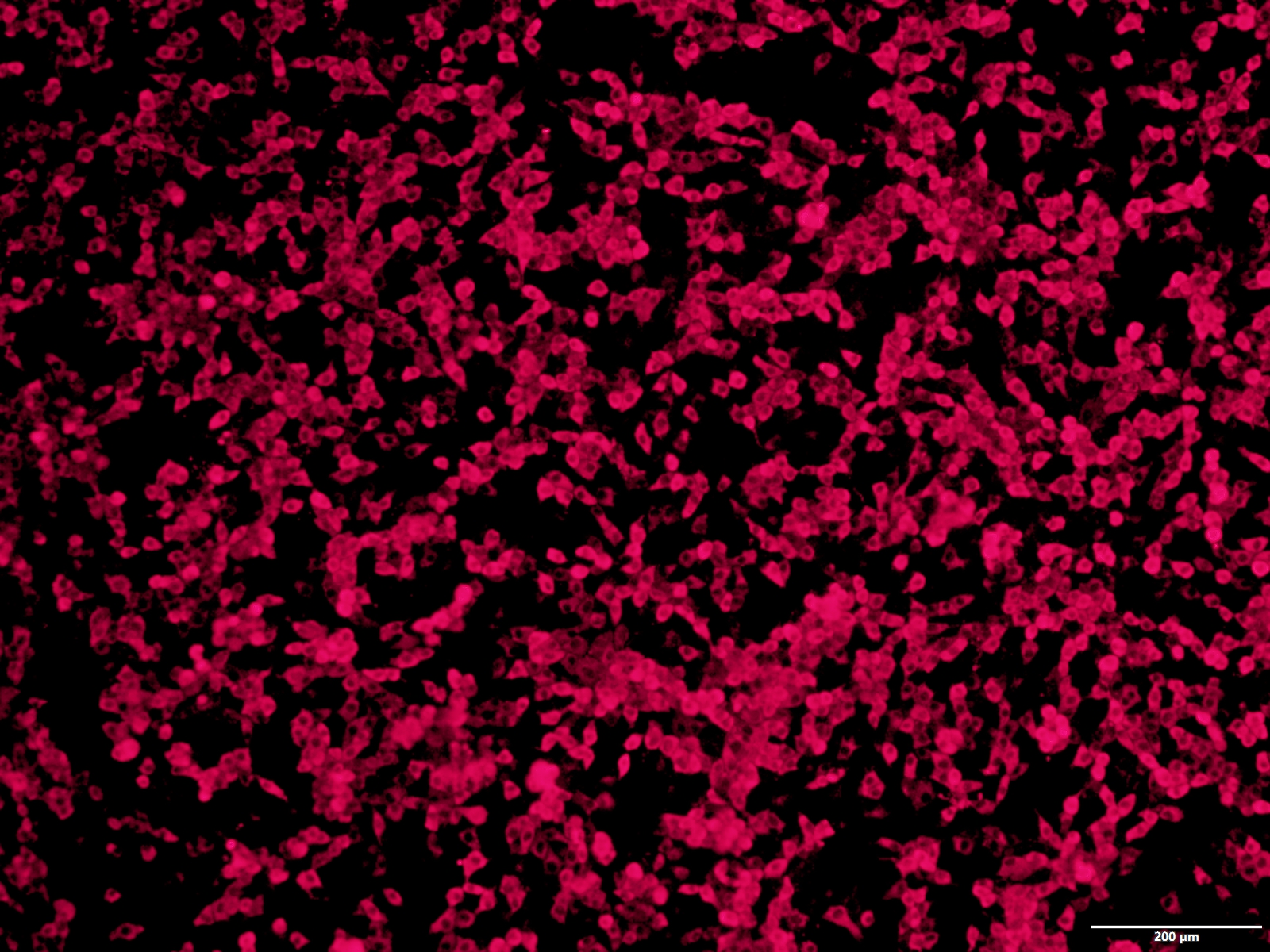

Supplement: S3 Data — This compressed folder contains the underlying numerical data and/or uncropped images used to generate the panels in Figs 3I and 4. (ZIP) [file pbio.3003736.s017.zip › S3 Data/Figure4/H/WT+CHO/pk-+dan-tgev-n-3.jpg]

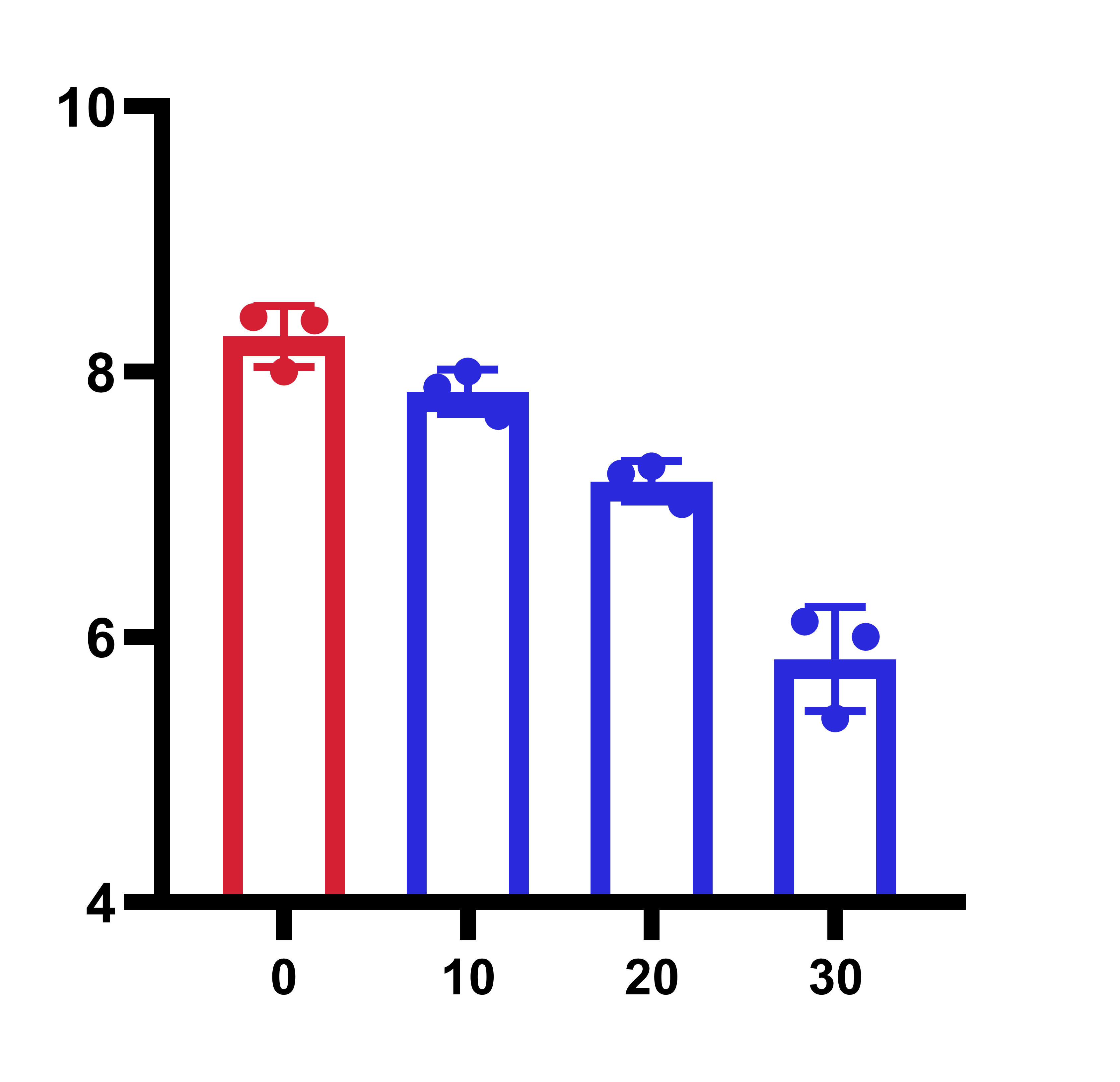

Supplement: S4 Data — This compressed folder contains the underlying numerical data and/or uncropped images used to generate the panels in Fig 5. (ZIP) [file pbio.3003736.s018.zip › S4 Data/Figure 5/B/20HC-TITER.tif]

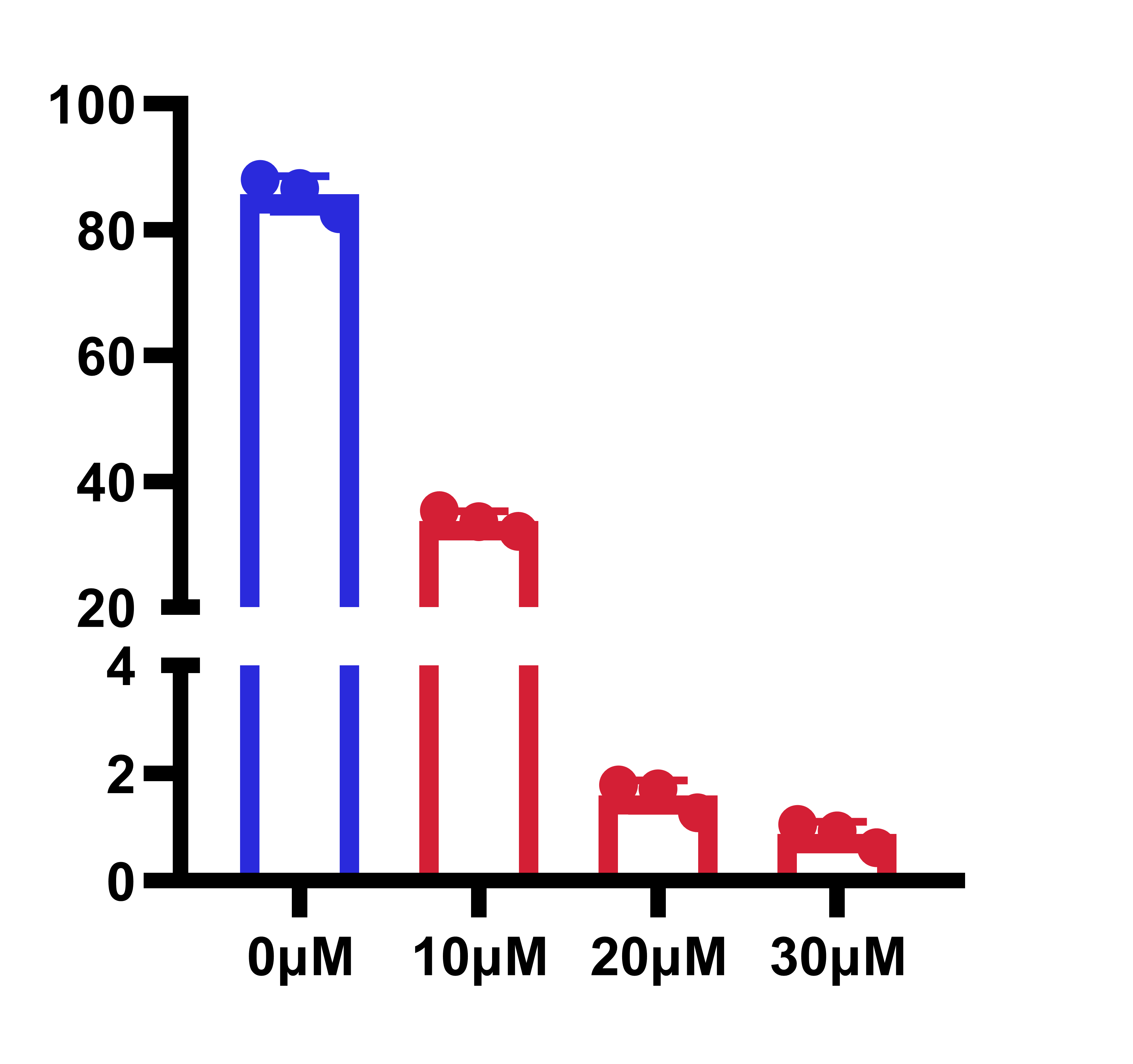

Supplement: S4 Data — This compressed folder contains the underlying numerical data and/or uncropped images used to generate the panels in Fig 5. (ZIP) [file pbio.3003736.s018.zip › S4 Data/Figure 5/C/POSITIVE-CELLS.tif]

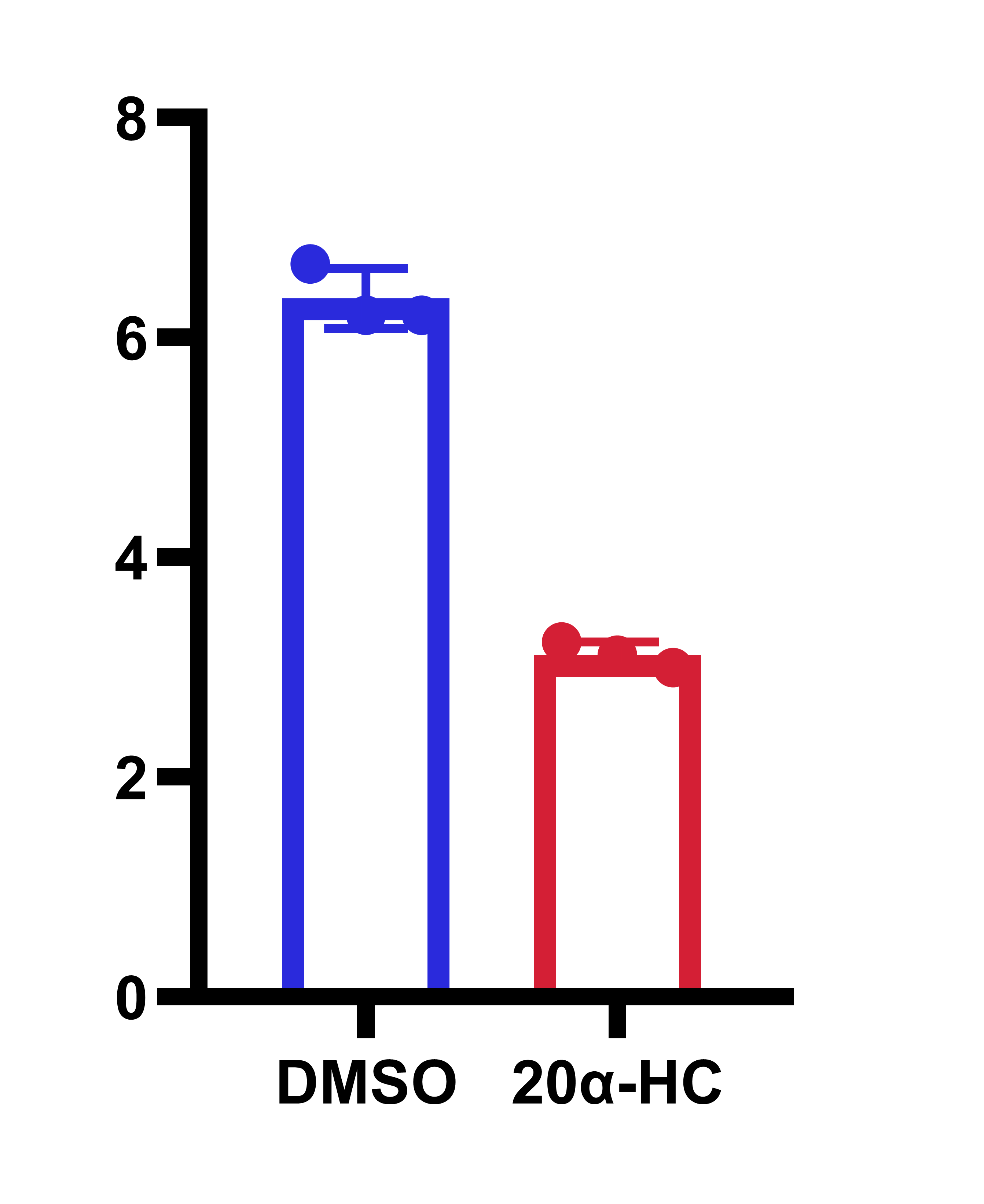

Supplement: S4 Data — This compressed folder contains the underlying numerical data and/or uncropped images used to generate the panels in Fig 5. (ZIP) [file pbio.3003736.s018.zip › S4 Data/Figure 5/D/20HC-TITER/TITER.tif]

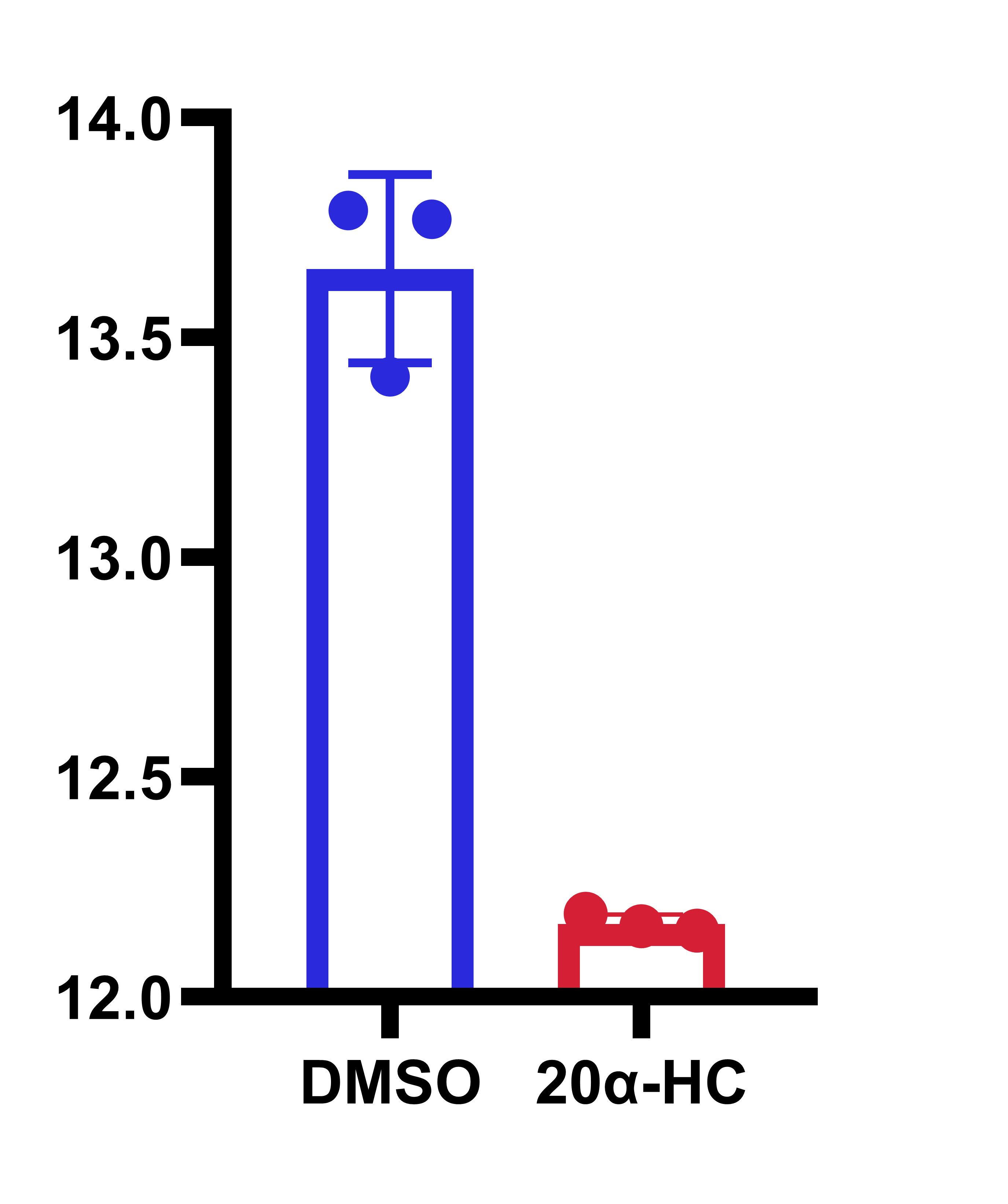

Supplement: S4 Data — This compressed folder contains the underlying numerical data and/or uncropped images used to generate the panels in Fig 5. (ZIP) [file pbio.3003736.s018.zip › S4 Data/Figure 5/D/QPCR/QP-1STAGE.tif]

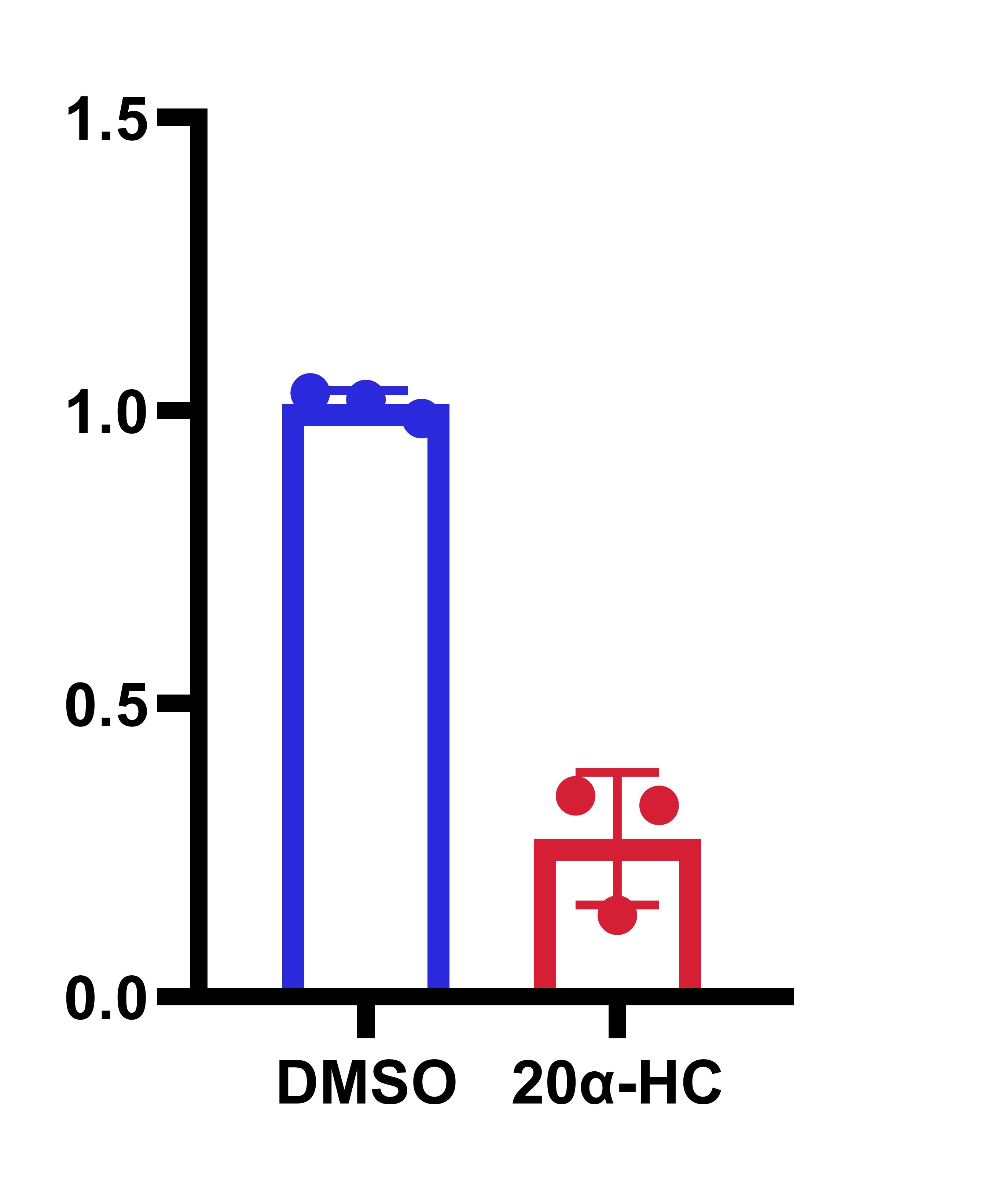

Supplement: S4 Data — This compressed folder contains the underlying numerical data and/or uncropped images used to generate the panels in Fig 5. (ZIP) [file pbio.3003736.s018.zip › S4 Data/Figure 5/E/20-HC-mean.tif]

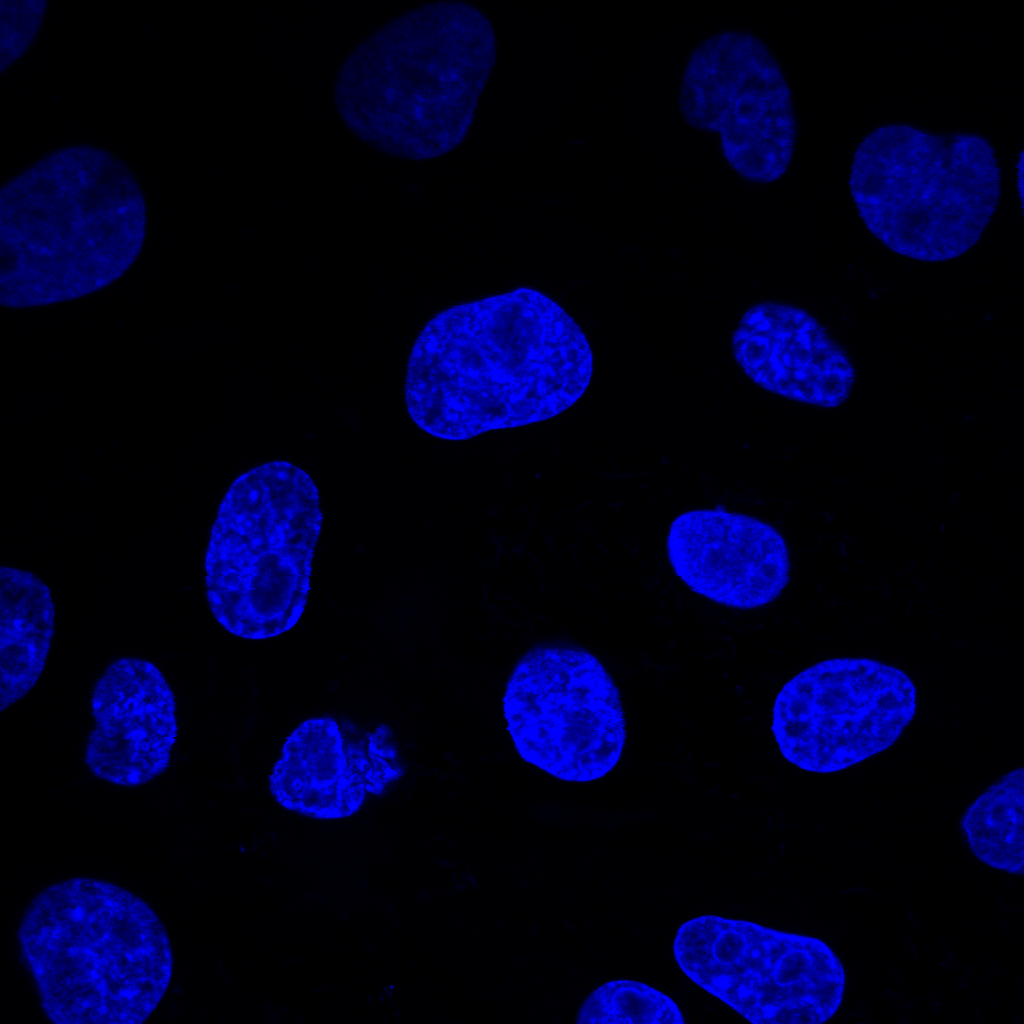

Supplement: S4 Data — This compressed folder contains the underlying numerical data and/or uncropped images used to generate the panels in Fig 5. (ZIP) [file pbio.3003736.s018.zip › S4 Data/Figure 5/E/pk-15-tgev-2_RGB_DAPI.tif]

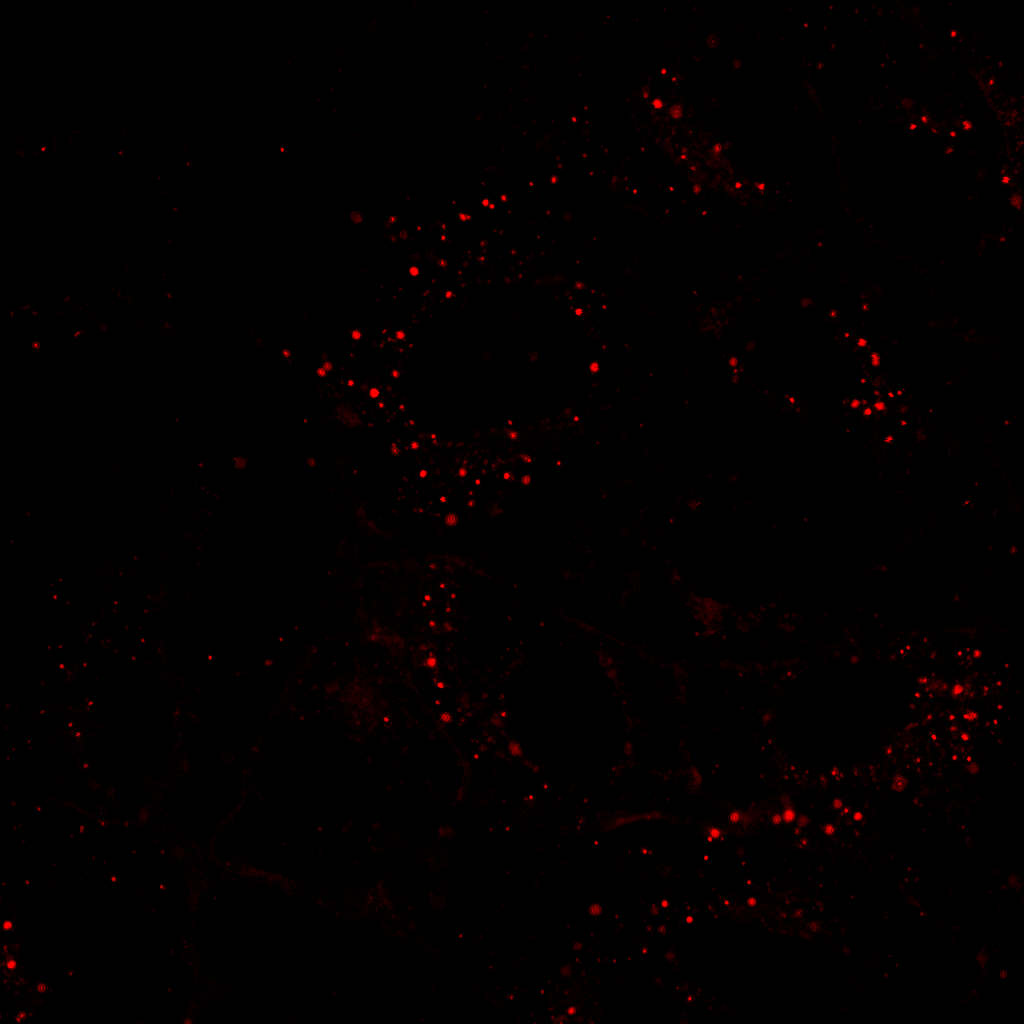

Supplement: S4 Data — This compressed folder contains the underlying numerical data and/or uncropped images used to generate the panels in Fig 5. (ZIP) [file pbio.3003736.s018.zip › S4 Data/Figure 5/E/pk-15-tgev-2_RGB_TRITC.tif]

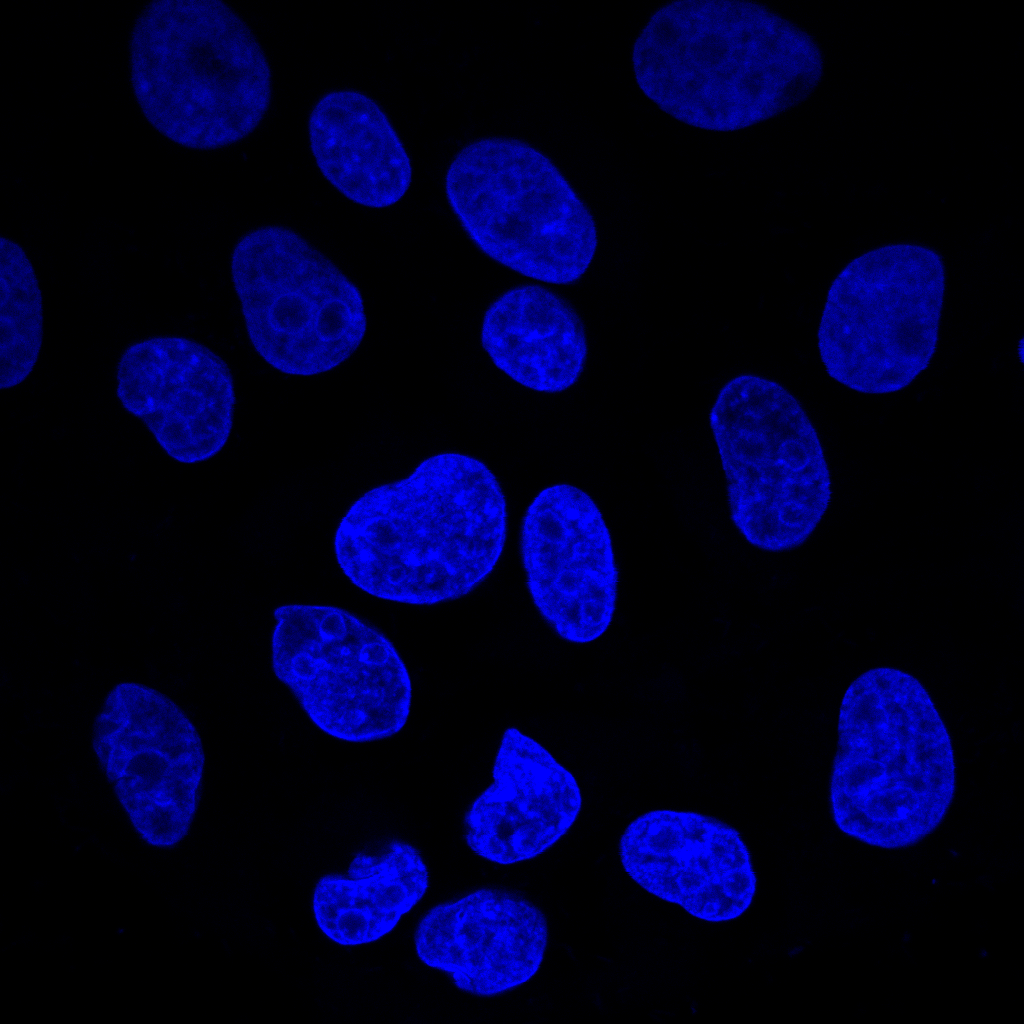

Supplement: S4 Data — This compressed folder contains the underlying numerical data and/or uncropped images used to generate the panels in Fig 5. (ZIP) [file pbio.3003736.s018.zip › S4 Data/Figure 5/E/pk-15-tgev-3_RGB_DAPI.tif]

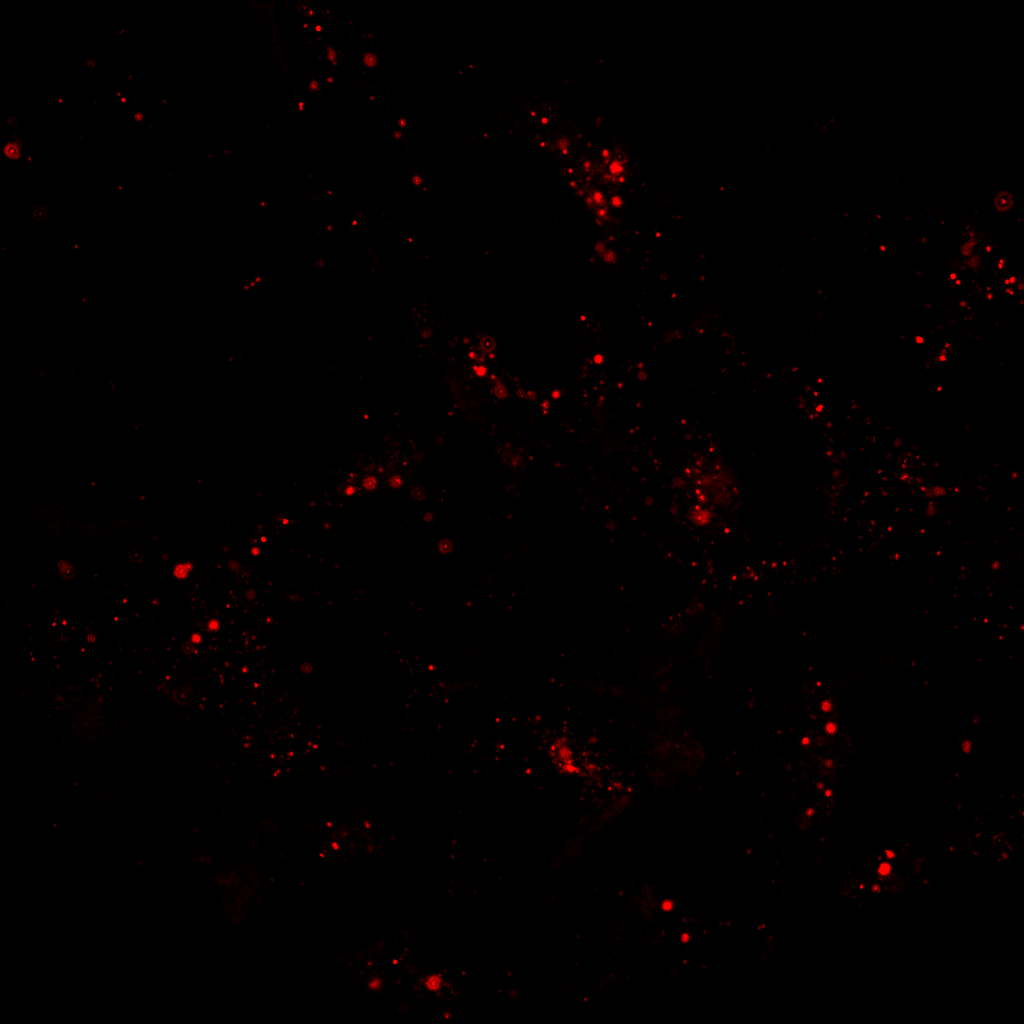

Supplement: S4 Data — This compressed folder contains the underlying numerical data and/or uncropped images used to generate the panels in Fig 5. (ZIP) [file pbio.3003736.s018.zip › S4 Data/Figure 5/E/pk-15-tgev-3_RGB_TRITC.tif]

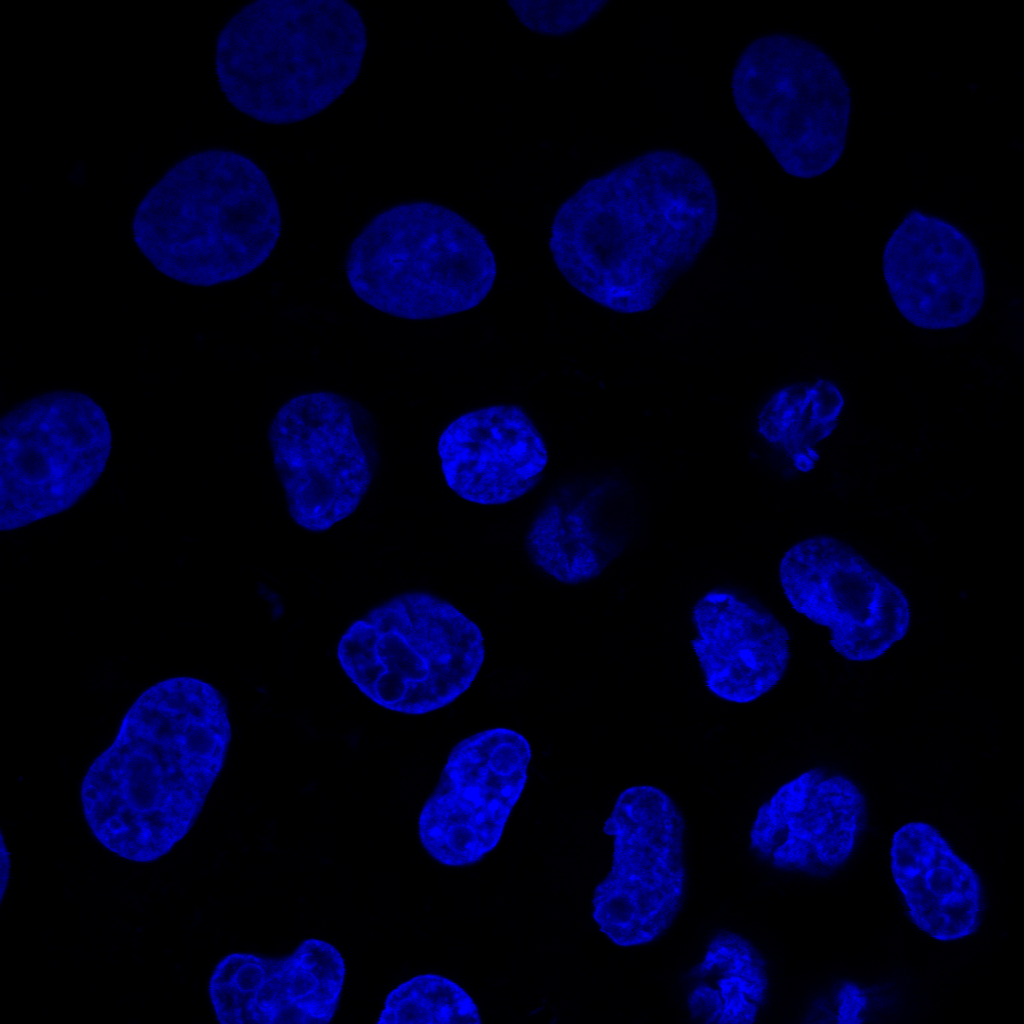

Supplement: S4 Data — This compressed folder contains the underlying numerical data and/or uncropped images used to generate the panels in Fig 5. (ZIP) [file pbio.3003736.s018.zip › S4 Data/Figure 5/E/pk-15-tgev-4_RGB_DAPI.tif]

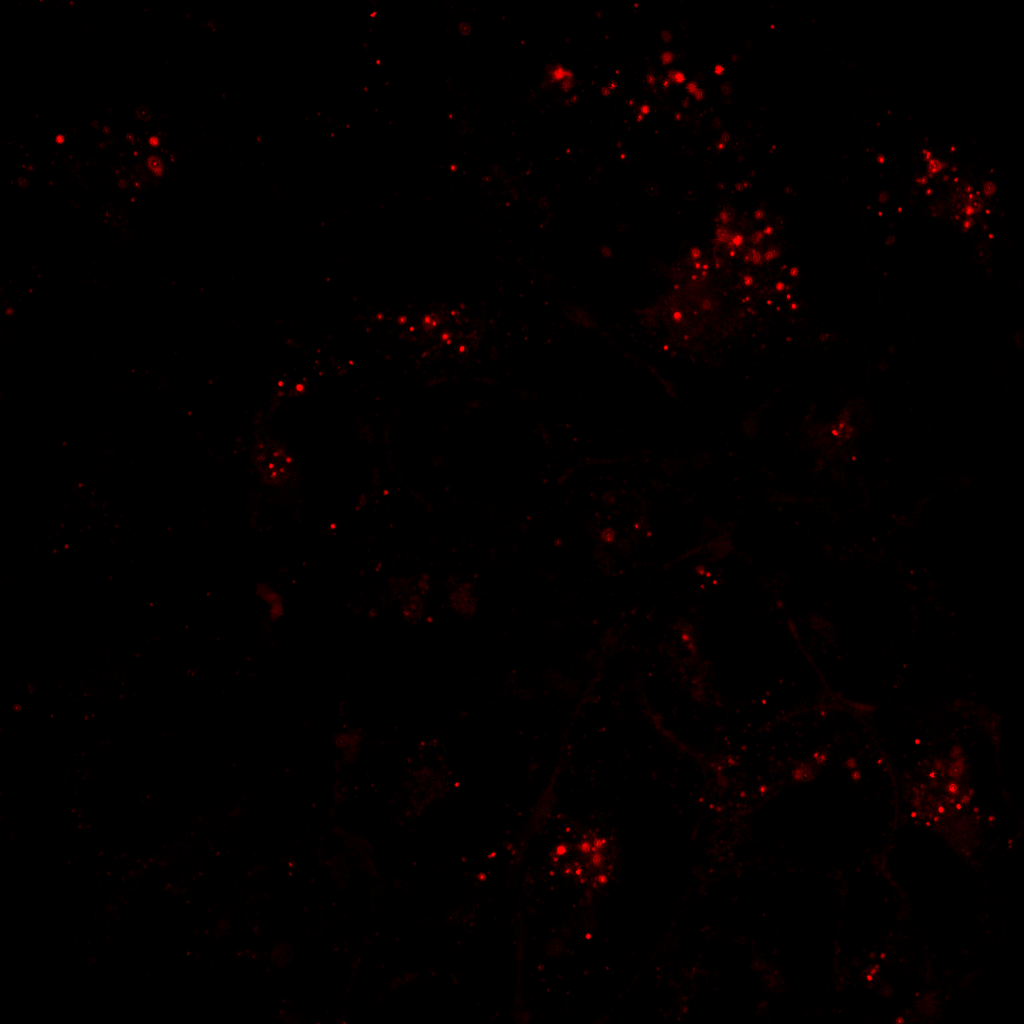

Supplement: S4 Data — This compressed folder contains the underlying numerical data and/or uncropped images used to generate the panels in Fig 5. (ZIP) [file pbio.3003736.s018.zip › S4 Data/Figure 5/E/pk-15-tgev-4_RGB_TRITC.tif]

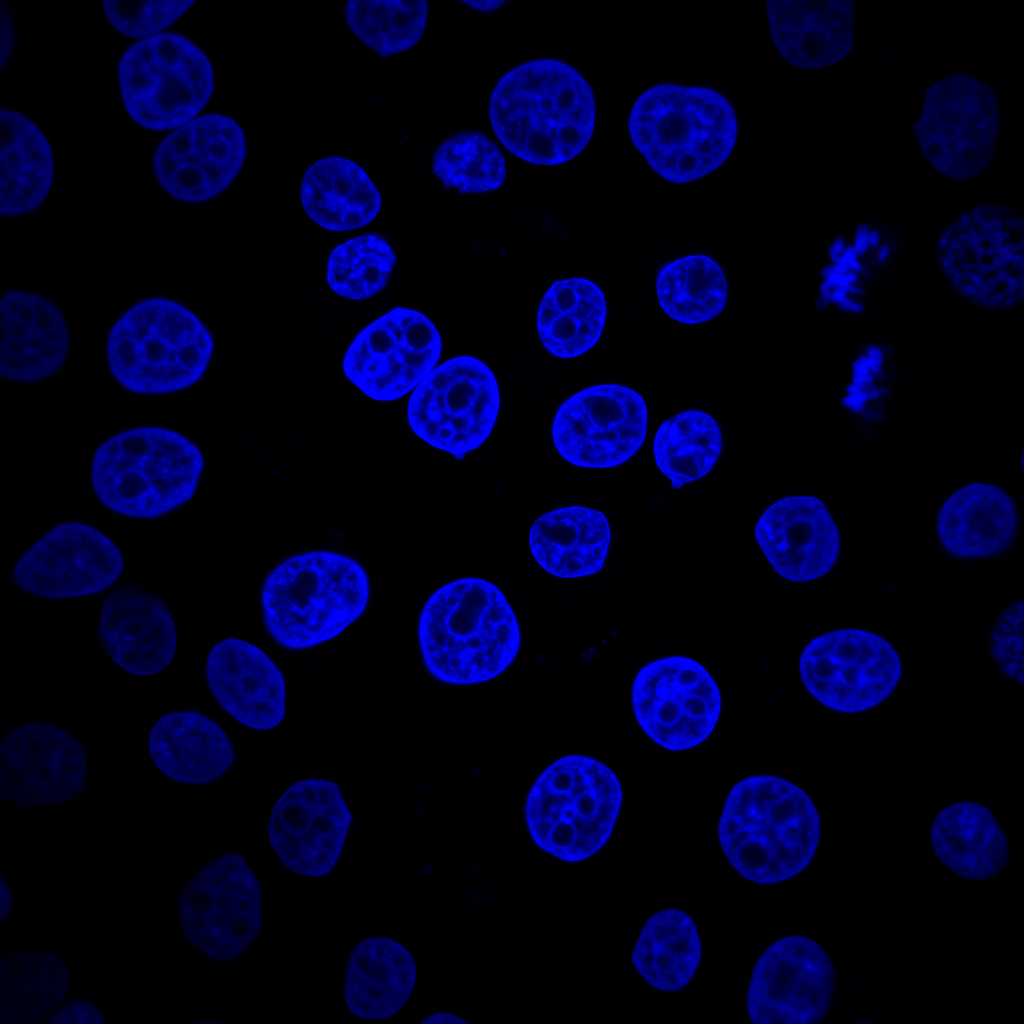

Supplement: S4 Data — This compressed folder contains the underlying numerical data and/or uncropped images used to generate the panels in Fig 5. (ZIP) [file pbio.3003736.s018.zip › S4 Data/Figure 5/E/pk-20hc-3_RGB_DAPI.tif]

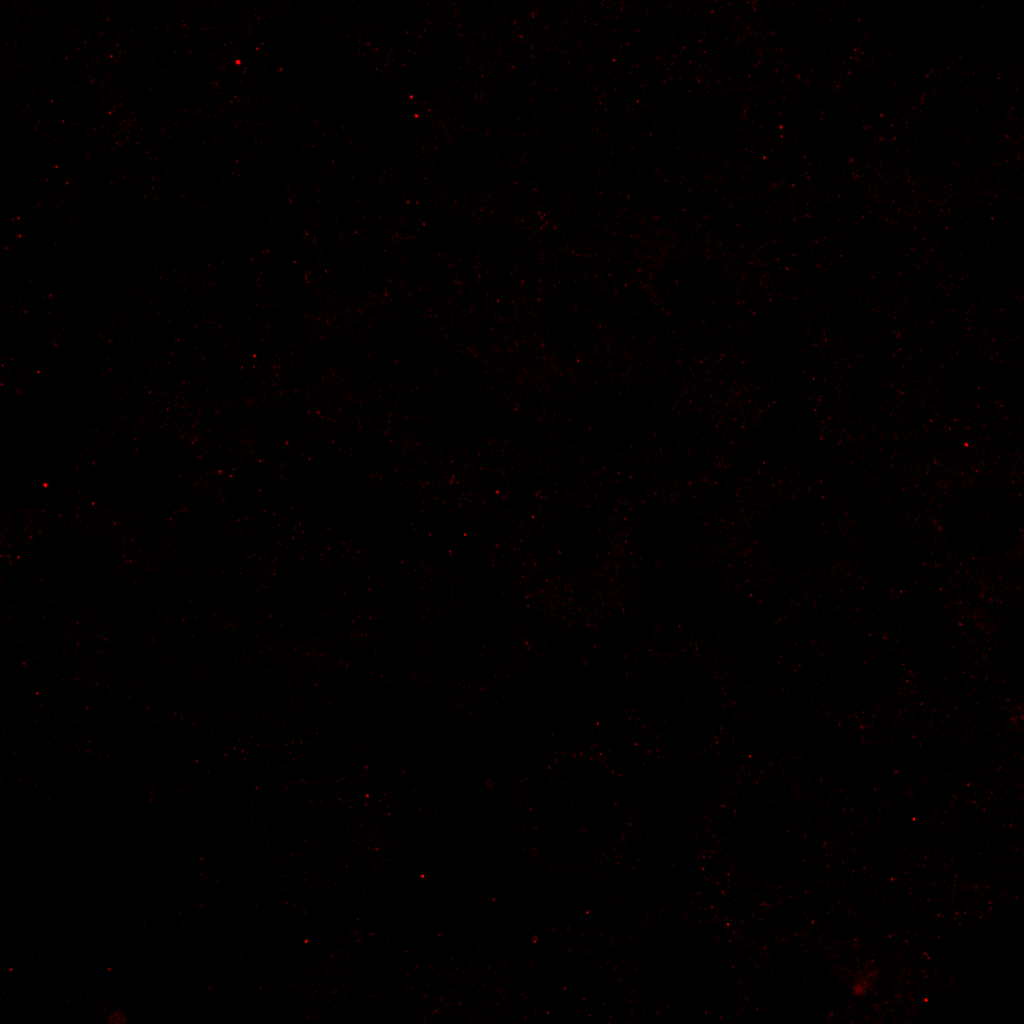

Supplement: S4 Data — This compressed folder contains the underlying numerical data and/or uncropped images used to generate the panels in Fig 5. (ZIP) [file pbio.3003736.s018.zip › S4 Data/Figure 5/E/pk-20hc-3_RGB_TRITC.tif]

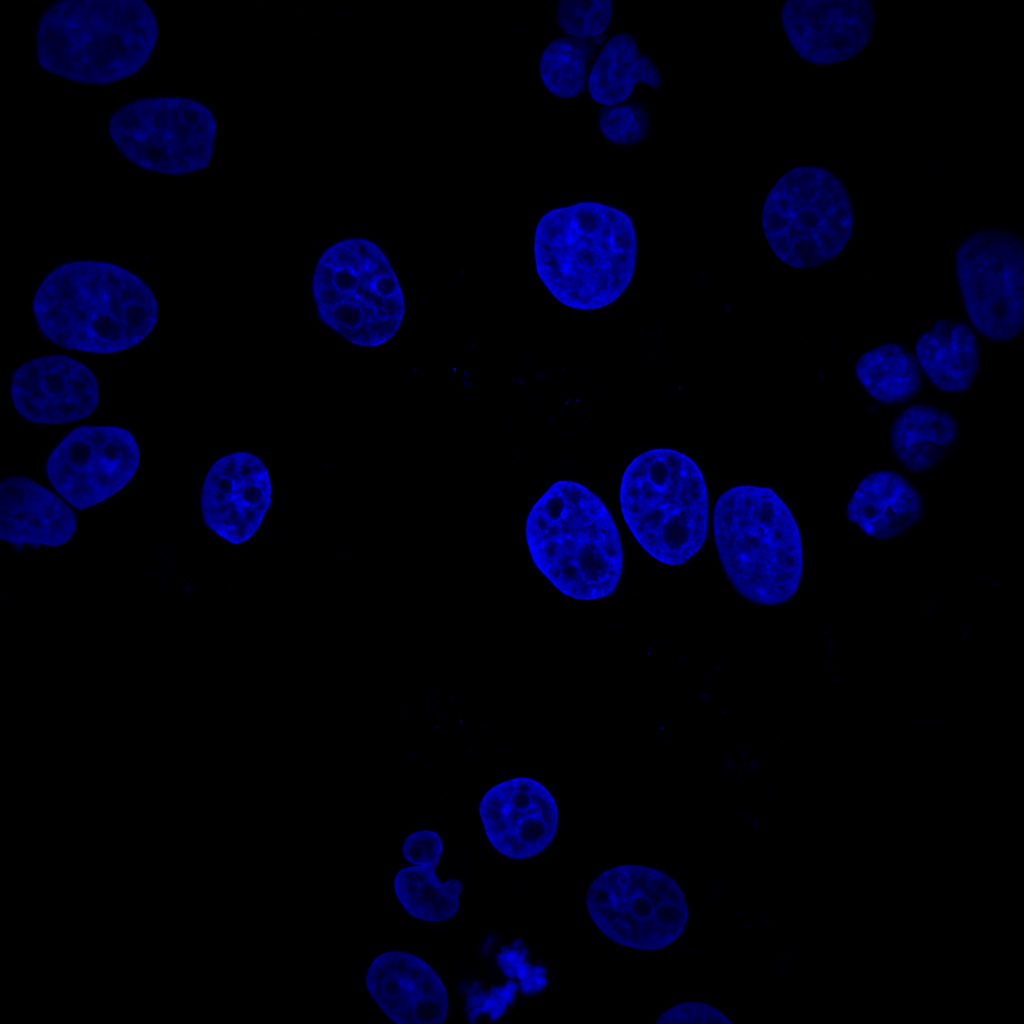

Supplement: S4 Data — This compressed folder contains the underlying numerical data and/or uncropped images used to generate the panels in Fig 5. (ZIP) [file pbio.3003736.s018.zip › S4 Data/Figure 5/E/pk-20hc-4_RGB_DAPI.tif]

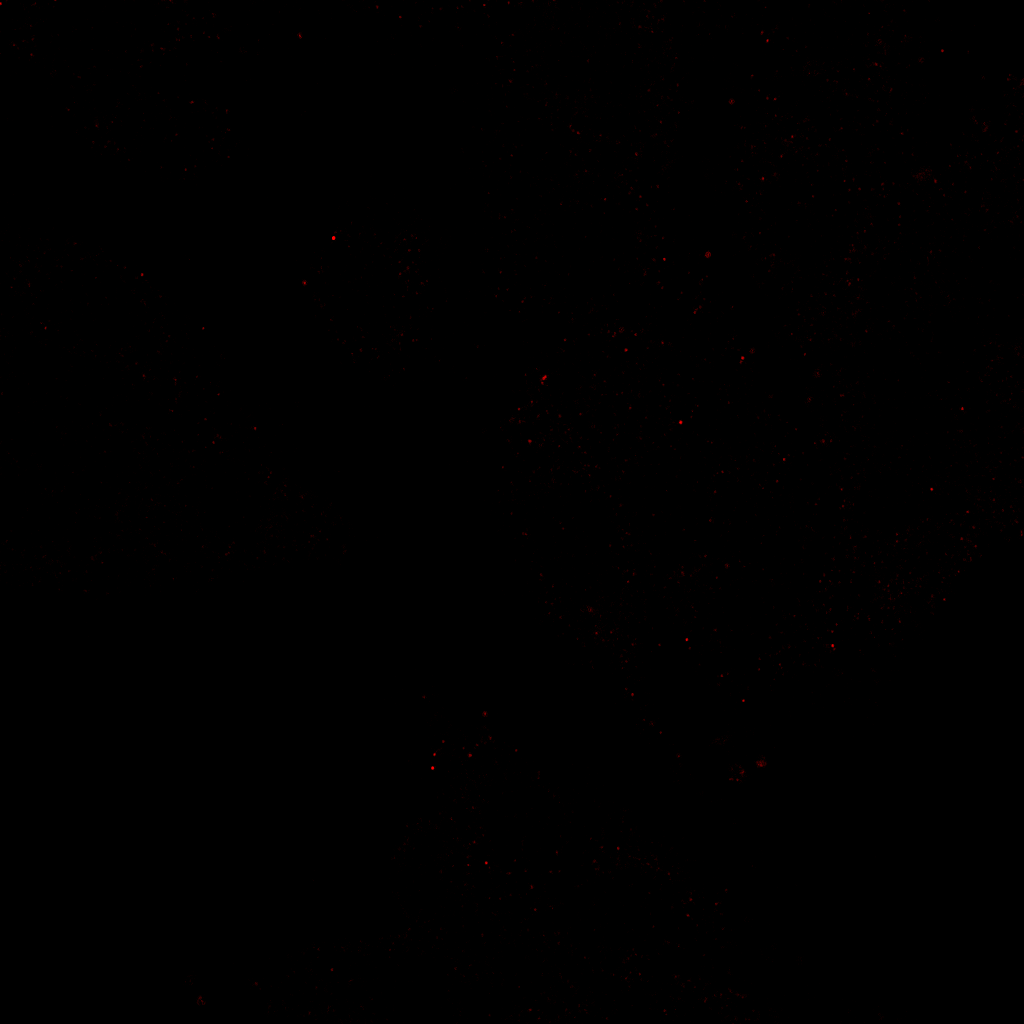

Supplement: S4 Data — This compressed folder contains the underlying numerical data and/or uncropped images used to generate the panels in Fig 5. (ZIP) [file pbio.3003736.s018.zip › S4 Data/Figure 5/E/pk-20hc-4_RGB_TRITC.tif]

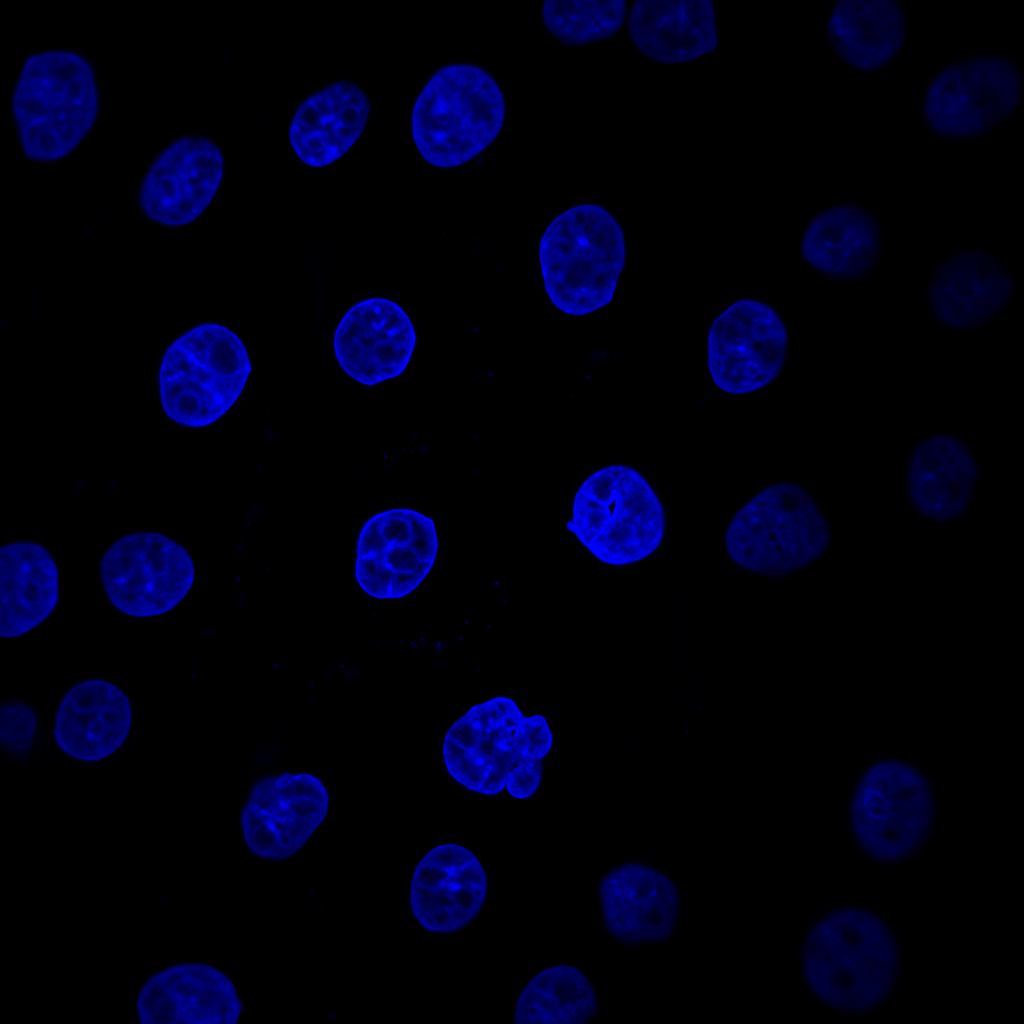

Supplement: S4 Data — This compressed folder contains the underlying numerical data and/or uncropped images used to generate the panels in Fig 5. (ZIP) [file pbio.3003736.s018.zip › S4 Data/Figure 5/E/pk20hc-1_RGB_DAPI.tif]

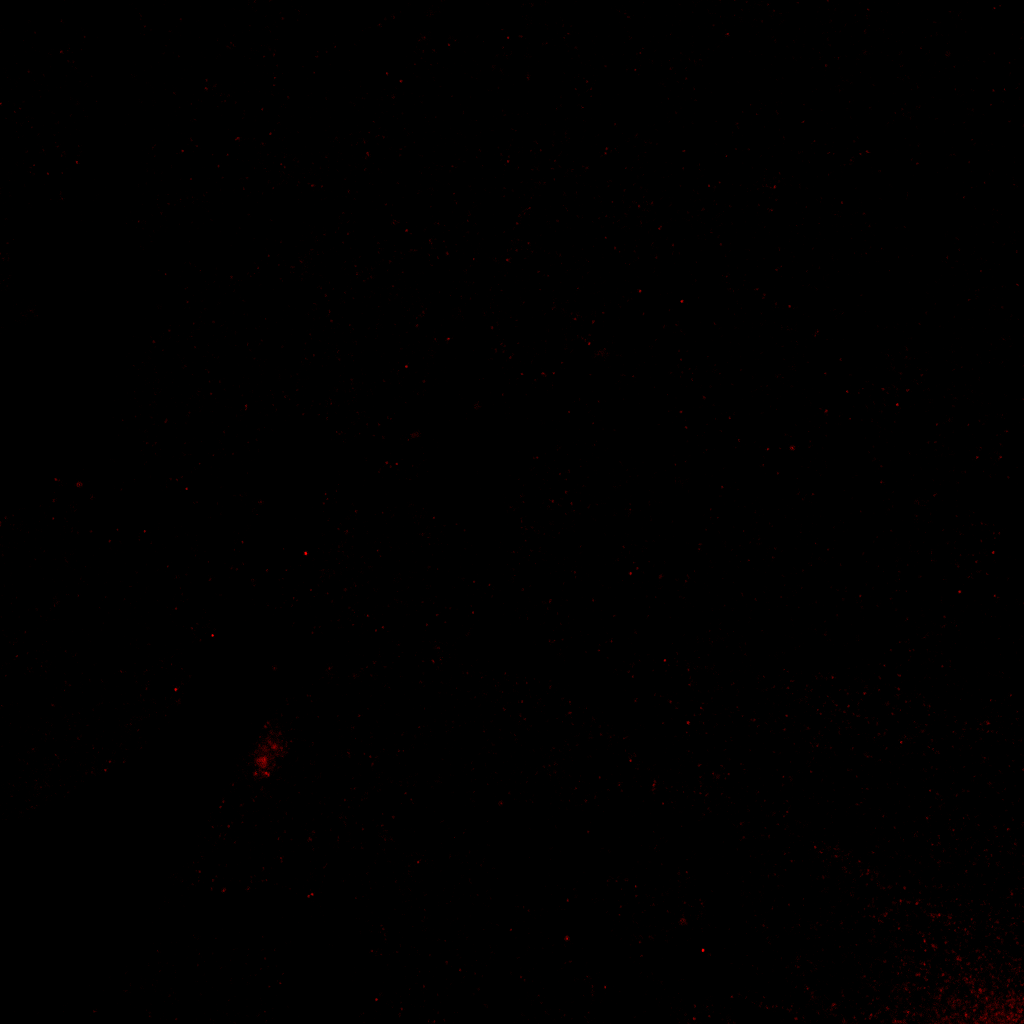

Supplement: S4 Data — This compressed folder contains the underlying numerical data and/or uncropped images used to generate the panels in Fig 5. (ZIP) [file pbio.3003736.s018.zip › S4 Data/Figure 5/E/pk20hc-1_RGB_TRITC.tif]

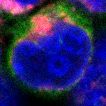

Supplement: S4 Data — This compressed folder contains the underlying numerical data and/or uncropped images used to generate the panels in Fig 5. (ZIP) [file pbio.3003736.s018.zip › S4 Data/Figure 5/F/Co-localization analysis/10uM/1/10-4_RGB.png]

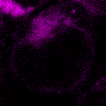

Supplement: S4 Data — This compressed folder contains the underlying numerical data and/or uncropped images used to generate the panels in Fig 5. (ZIP) [file pbio.3003736.s018.zip › S4 Data/Figure 5/F/Co-localization analysis/10uM/1/10-4_RGB_Cy5.png]

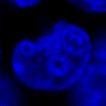

Supplement: S4 Data — This compressed folder contains the underlying numerical data and/or uncropped images used to generate the panels in Fig 5. (ZIP) [file pbio.3003736.s018.zip › S4 Data/Figure 5/F/Co-localization analysis/10uM/1/10-4_RGB_DAPI.png]

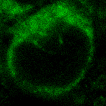

Supplement: S4 Data — This compressed folder contains the underlying numerical data and/or uncropped images used to generate the panels in Fig 5. (ZIP) [file pbio.3003736.s018.zip › S4 Data/Figure 5/F/Co-localization analysis/10uM/1/10-4_RGB_FITC.png]

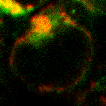

Supplement: S4 Data — This compressed folder contains the underlying numerical data and/or uncropped images used to generate the panels in Fig 5. (ZIP) [file pbio.3003736.s018.zip › S4 Data/Figure 5/F/Co-localization analysis/10uM/1/10-4_RGB_TRITC.jpg]

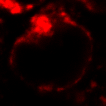

Supplement: S4 Data — This compressed folder contains the underlying numerical data and/or uncropped images used to generate the panels in Fig 5. (ZIP) [file pbio.3003736.s018.zip › S4 Data/Figure 5/F/Co-localization analysis/10uM/1/10-4_RGB_TRITC.png]

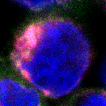

Supplement: S4 Data — This compressed folder contains the underlying numerical data and/or uncropped images used to generate the panels in Fig 5. (ZIP) [file pbio.3003736.s018.zip › S4 Data/Figure 5/F/Co-localization analysis/10uM/2/10-4_RGB-2.png]

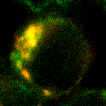

Supplement: S4 Data — This compressed folder contains the underlying numerical data and/or uncropped images used to generate the panels in Fig 5. (ZIP) [file pbio.3003736.s018.zip › S4 Data/Figure 5/F/Co-localization analysis/10uM/2/10-4_RGB_Cy5-2.jpg]

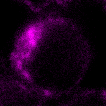

Supplement: S4 Data — This compressed folder contains the underlying numerical data and/or uncropped images used to generate the panels in Fig 5. (ZIP) [file pbio.3003736.s018.zip › S4 Data/Figure 5/F/Co-localization analysis/10uM/2/10-4_RGB_Cy5-2.png]

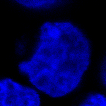

Supplement: S4 Data — This compressed folder contains the underlying numerical data and/or uncropped images used to generate the panels in Fig 5. (ZIP) [file pbio.3003736.s018.zip › S4 Data/Figure 5/F/Co-localization analysis/10uM/2/10-4_RGB_DAPI-2.png]

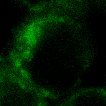

Supplement: S4 Data — This compressed folder contains the underlying numerical data and/or uncropped images used to generate the panels in Fig 5. (ZIP) [file pbio.3003736.s018.zip › S4 Data/Figure 5/F/Co-localization analysis/10uM/2/10-4_RGB_FITC-2.png]

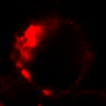

Supplement: S4 Data — This compressed folder contains the underlying numerical data and/or uncropped images used to generate the panels in Fig 5. (ZIP) [file pbio.3003736.s018.zip › S4 Data/Figure 5/F/Co-localization analysis/10uM/2/10-4_RGB_TRITC-2.png]

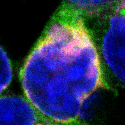

Supplement: S4 Data — This compressed folder contains the underlying numerical data and/or uncropped images used to generate the panels in Fig 5. (ZIP) [file pbio.3003736.s018.zip › S4 Data/Figure 5/F/Co-localization analysis/10uM/3/10-5_RGB.png]

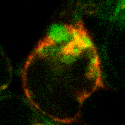

Supplement: S4 Data — This compressed folder contains the underlying numerical data and/or uncropped images used to generate the panels in Fig 5. (ZIP) [file pbio.3003736.s018.zip › S4 Data/Figure 5/F/Co-localization analysis/10uM/3/10-5_RGB_Cy5.jpg]

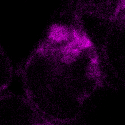

Supplement: S4 Data — This compressed folder contains the underlying numerical data and/or uncropped images used to generate the panels in Fig 5. (ZIP) [file pbio.3003736.s018.zip › S4 Data/Figure 5/F/Co-localization analysis/10uM/3/10-5_RGB_Cy5.png]

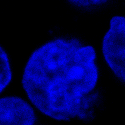

Supplement: S4 Data — This compressed folder contains the underlying numerical data and/or uncropped images used to generate the panels in Fig 5. (ZIP) [file pbio.3003736.s018.zip › S4 Data/Figure 5/F/Co-localization analysis/10uM/3/10-5_RGB_DAPI.png]

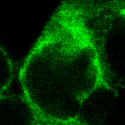

Supplement: S4 Data — This compressed folder contains the underlying numerical data and/or uncropped images used to generate the panels in Fig 5. (ZIP) [file pbio.3003736.s018.zip › S4 Data/Figure 5/F/Co-localization analysis/10uM/3/10-5_RGB_FITC.png]

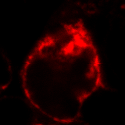

Supplement: S4 Data — This compressed folder contains the underlying numerical data and/or uncropped images used to generate the panels in Fig 5. (ZIP) [file pbio.3003736.s018.zip › S4 Data/Figure 5/F/Co-localization analysis/10uM/3/10-5_RGB_TRITC.png]

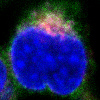

Supplement: S4 Data — This compressed folder contains the underlying numerical data and/or uncropped images used to generate the panels in Fig 5. (ZIP) [file pbio.3003736.s018.zip › S4 Data/Figure 5/F/Co-localization analysis/10uM/4/10-5_RGB.png]

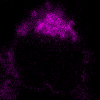

Supplement: S4 Data — This compressed folder contains the underlying numerical data and/or uncropped images used to generate the panels in Fig 5. (ZIP) [file pbio.3003736.s018.zip › S4 Data/Figure 5/F/Co-localization analysis/10uM/4/10-5_RGB_Cy5.png]

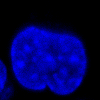

Supplement: S4 Data — This compressed folder contains the underlying numerical data and/or uncropped images used to generate the panels in Fig 5. (ZIP) [file pbio.3003736.s018.zip › S4 Data/Figure 5/F/Co-localization analysis/10uM/4/10-5_RGB_DAPI.png]

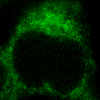

Supplement: S4 Data — This compressed folder contains the underlying numerical data and/or uncropped images used to generate the panels in Fig 5. (ZIP) [file pbio.3003736.s018.zip › S4 Data/Figure 5/F/Co-localization analysis/10uM/4/10-5_RGB_FITC.png]

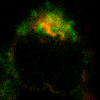

Supplement: S4 Data — This compressed folder contains the underlying numerical data and/or uncropped images used to generate the panels in Fig 5. (ZIP) [file pbio.3003736.s018.zip › S4 Data/Figure 5/F/Co-localization analysis/10uM/4/10-5_RGB_TRITC.jpg]

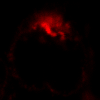

Supplement: S4 Data — This compressed folder contains the underlying numerical data and/or uncropped images used to generate the panels in Fig 5. (ZIP) [file pbio.3003736.s018.zip › S4 Data/Figure 5/F/Co-localization analysis/10uM/4/10-5_RGB_TRITC.png]

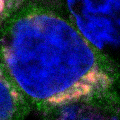

Supplement: S4 Data — This compressed folder contains the underlying numerical data and/or uncropped images used to generate the panels in Fig 5. (ZIP) [file pbio.3003736.s018.zip › S4 Data/Figure 5/F/Co-localization analysis/10uM/5/10-6_RGB.png]

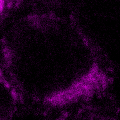

Supplement: S4 Data — This compressed folder contains the underlying numerical data and/or uncropped images used to generate the panels in Fig 5. (ZIP) [file pbio.3003736.s018.zip › S4 Data/Figure 5/F/Co-localization analysis/10uM/5/10-6_RGB_Cy5.png]
