# Supplementary material for: The non-vesicular cholesterol transporter GRAMD1C is a pan-coronavirus antiviral target
Source: PLoS Biol. 2026 Apr 6;24(4):e3003736. doi: 10.1371/journal.pbio.3003736 (PMC13068348; doi:10.1371/journal.pbio.3003736)
Supplement: S4 Data — This compressed folder contains the underlying numerical data and/or uncropped images used to generate the panels in Fig 5. (ZIP) [file pbio.3003736.s018.zip › S4 Data/Figure 5/F/Co-localization analysis.docx]

DMSO

Red - Green co-localization parameters of PK-5_RGB_MERGE.jpg (AOI)

Pearson's correlation Rr= 0.930524

Overlap coefficient R = 0.917843

Overlap coefficient k1= 0.846976

Overlap coefficient k2= 0.994639

Co-localization (ch2>0) m1= 0.998960

Co-localization (ch1>0) m2= 0.908523

Red - Green co-localization parameters of PK-5_RGB_MERGE.jpg:2 (AOI)

Pearson's correlation Rr= 0.915346

Overlap coefficient R = 0.916240

Overlap coefficient k1= 0.817781

Overlap coefficient k2= 1.026552

Co-localization (ch2>0) m1= 0.998926

Co-localization (ch1>0) m2= 0.927751

Red - Green co-localization parameters of PK-5_RGBMERGE.jpg (AOI)

Pearson's correlation Rr= 0.929904

Overlap coefficient R = 0.918238

Overlap coefficient k1= 1.038810

Overlap coefficient k2= 0.811660

Co-localization (ch2>0) m1= 0.997056

Co-localization (ch1>0) m2= 0.896103

Red - Green co-localization parameters of PK-6_RGB_MERGE.jpg (AOI)

Pearson's correlation Rr= 0.937453

Overlap coefficient R = 0.931682

Overlap coefficient k1= 0.894531

Overlap coefficient k2= 0.970376

Co-localization (ch2>0) m1= 0.999431

Co-localization (ch1>0) m2= 0.900860

Red - Green co-localization parameters of PK-6_RGBMERGE.jpg (AOI)

Pearson's correlation Rr= 0.876981

Overlap coefficient R = 0.867514

Overlap coefficient k1= 0.902970

Overlap coefficient k2= 0.833450

Co-localization (ch2>0) m1= 0.996894

Co-localization (ch1>0) m2= 0.885190

Red - Green co-localization parameters of PK-6_RGB_MERGE.jpg:2 (AOI)

Pearson's correlation Rr= 0.893614

Overlap coefficient R = 0.887495

Overlap coefficient k1= 0.976478

Overlap coefficient k2= 0.806622

Co-localization (ch2>0) m1= 0.997883

Co-localization (ch1>0) m2= 0.907606

10um

Red - Green co-localization parameters of 10-4_RGB_TRITC.jpg (AOI)

Pearson's correlation Rr= 0.787409

Overlap coefficient R = 0.835881

Overlap coefficient k1= 0.737336

Overlap coefficient k2= 0.947598

Co-localization (ch2>0) m1= 0.958073

Co-localization (ch1>0) m2= 0.960589

Red - Green co-localization parameters of 10-4_RGB_Cy5-2.jpg (AOI)

Pearson's correlation Rr= 0.880388

Overlap coefficient R = 0.898060

Overlap coefficient k1= 0.915301

Overlap coefficient k2= 0.881144

Co-localization (ch2>0) m1= 0.980622

Co-localization (ch1>0) m2= 0.958355

Red - Green co-localization parameters of 10-5_RGB_Cy5.jpg (AOI)

Pearson's correlation Rr= 0.745755

Overlap coefficient R = 0.808632

Overlap coefficient k1= 0.747002

Overlap coefficient k2= 0.875348

Co-localization (ch2>0) m1= 0.979866

Co-localization (ch1>0) m2= 0.953549

Red - Green co-localization parameters of 10-5_RGB_TRITC.jpg (AOI)

Pearson's correlation Rr= 0.814004

Overlap coefficient R = 0.839600

Overlap coefficient k1= 0.896240

Overlap coefficient k2= 0.786541

Co-localization (ch2>0) m1= 0.968588

Co-localization (ch1>0) m2= 0.927290

Red - Green co-localization parameters of 10-6_RGB_TRITC.jpg (AOI)

Pearson's correlation Rr= 0.738316

Overlap coefficient R = 0.761402

Overlap coefficient k1= 0.776263

Overlap coefficient k2= 0.746827

Co-localization (ch2>0) m1= 0.990679

Co-localization (ch1>0) m2= 0.877130

Red - Green co-localization parameters of 10-6_RGB_TRITC.jpg:2 (AOI)

Pearson's correlation Rr= 0.588064

Overlap coefficient R = 0.681793

Overlap coefficient k1= 0.600968

Overlap coefficient k2= 0.773488

Co-localization (ch2>0) m1= 0.958229

Co-localization (ch1>0) m2= 0.924208
