# Supplementary figures and images for: The non-vesicular cholesterol transporter GRAMD1C is a pan-coronavirus antiviral target (part 3 of 4)
Source: PLoS Biol. 2026 Apr 6;24(4):e3003736. doi: 10.1371/journal.pbio.3003736 (PMC13068348; doi:10.1371/journal.pbio.3003736)

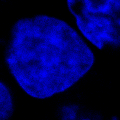

Supplement: S4 Data — This compressed folder contains the underlying numerical data and/or uncropped images used to generate the panels in Fig 5. (ZIP) [file pbio.3003736.s018.zip › S4 Data/Figure 5/F/Co-localization analysis/10uM/5/10-6_RGB_DAPI.png]

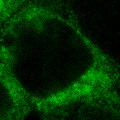

Supplement: S4 Data — This compressed folder contains the underlying numerical data and/or uncropped images used to generate the panels in Fig 5. (ZIP) [file pbio.3003736.s018.zip › S4 Data/Figure 5/F/Co-localization analysis/10uM/5/10-6_RGB_FITC.png]

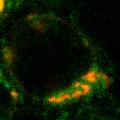

Supplement: S4 Data — This compressed folder contains the underlying numerical data and/or uncropped images used to generate the panels in Fig 5. (ZIP) [file pbio.3003736.s018.zip › S4 Data/Figure 5/F/Co-localization analysis/10uM/5/10-6_RGB_TRITC.jpg]

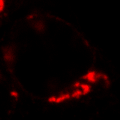

Supplement: S4 Data — This compressed folder contains the underlying numerical data and/or uncropped images used to generate the panels in Fig 5. (ZIP) [file pbio.3003736.s018.zip › S4 Data/Figure 5/F/Co-localization analysis/10uM/5/10-6_RGB_TRITC.png]

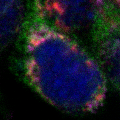

Supplement: S4 Data — This compressed folder contains the underlying numerical data and/or uncropped images used to generate the panels in Fig 5. (ZIP) [file pbio.3003736.s018.zip › S4 Data/Figure 5/F/Co-localization analysis/10uM/6/10-6_RGB.png]

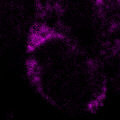

Supplement: S4 Data — This compressed folder contains the underlying numerical data and/or uncropped images used to generate the panels in Fig 5. (ZIP) [file pbio.3003736.s018.zip › S4 Data/Figure 5/F/Co-localization analysis/10uM/6/10-6_RGB_Cy5.png]

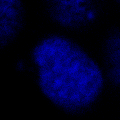

Supplement: S4 Data — This compressed folder contains the underlying numerical data and/or uncropped images used to generate the panels in Fig 5. (ZIP) [file pbio.3003736.s018.zip › S4 Data/Figure 5/F/Co-localization analysis/10uM/6/10-6_RGB_DAPI.png]

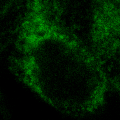

Supplement: S4 Data — This compressed folder contains the underlying numerical data and/or uncropped images used to generate the panels in Fig 5. (ZIP) [file pbio.3003736.s018.zip › S4 Data/Figure 5/F/Co-localization analysis/10uM/6/10-6_RGB_FITC.png]

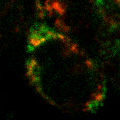

Supplement: S4 Data — This compressed folder contains the underlying numerical data and/or uncropped images used to generate the panels in Fig 5. (ZIP) [file pbio.3003736.s018.zip › S4 Data/Figure 5/F/Co-localization analysis/10uM/6/10-6_RGB_TRITC.jpg]

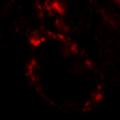

Supplement: S4 Data — This compressed folder contains the underlying numerical data and/or uncropped images used to generate the panels in Fig 5. (ZIP) [file pbio.3003736.s018.zip › S4 Data/Figure 5/F/Co-localization analysis/10uM/6/10-6_RGB_TRITC.png]

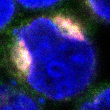

Supplement: S4 Data — This compressed folder contains the underlying numerical data and/or uncropped images used to generate the panels in Fig 5. (ZIP) [file pbio.3003736.s018.zip › S4 Data/Figure 5/F/Co-localization analysis/DMSO/1/PK-5_RGB.png]

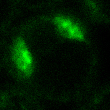

Supplement: S4 Data — This compressed folder contains the underlying numerical data and/or uncropped images used to generate the panels in Fig 5. (ZIP) [file pbio.3003736.s018.zip › S4 Data/Figure 5/F/Co-localization analysis/DMSO/1/PK-5_RGB_Cy5.jpg]

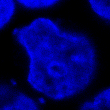

Supplement: S4 Data — This compressed folder contains the underlying numerical data and/or uncropped images used to generate the panels in Fig 5. (ZIP) [file pbio.3003736.s018.zip › S4 Data/Figure 5/F/Co-localization analysis/DMSO/1/PK-5_RGB_DAPI.png]

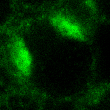

Supplement: S4 Data — This compressed folder contains the underlying numerical data and/or uncropped images used to generate the panels in Fig 5. (ZIP) [file pbio.3003736.s018.zip › S4 Data/Figure 5/F/Co-localization analysis/DMSO/1/PK-5_RGB_FITC.png]

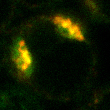

Supplement: S4 Data — This compressed folder contains the underlying numerical data and/or uncropped images used to generate the panels in Fig 5. (ZIP) [file pbio.3003736.s018.zip › S4 Data/Figure 5/F/Co-localization analysis/DMSO/1/PK-5_RGB_MERGE.jpg]

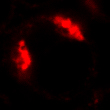

Supplement: S4 Data — This compressed folder contains the underlying numerical data and/or uncropped images used to generate the panels in Fig 5. (ZIP) [file pbio.3003736.s018.zip › S4 Data/Figure 5/F/Co-localization analysis/DMSO/1/PK-5_RGB_TRITC.jpg]

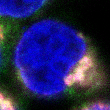

Supplement: S4 Data — This compressed folder contains the underlying numerical data and/or uncropped images used to generate the panels in Fig 5. (ZIP) [file pbio.3003736.s018.zip › S4 Data/Figure 5/F/Co-localization analysis/DMSO/2/PK-5_RGB.png]

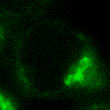

Supplement: S4 Data — This compressed folder contains the underlying numerical data and/or uncropped images used to generate the panels in Fig 5. (ZIP) [file pbio.3003736.s018.zip › S4 Data/Figure 5/F/Co-localization analysis/DMSO/2/PK-5_RGB_Cy5.jpg]

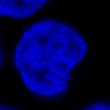

Supplement: S4 Data — This compressed folder contains the underlying numerical data and/or uncropped images used to generate the panels in Fig 5. (ZIP) [file pbio.3003736.s018.zip › S4 Data/Figure 5/F/Co-localization analysis/DMSO/2/PK-5_RGB_DAPI.png]

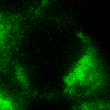

Supplement: S4 Data — This compressed folder contains the underlying numerical data and/or uncropped images used to generate the panels in Fig 5. (ZIP) [file pbio.3003736.s018.zip › S4 Data/Figure 5/F/Co-localization analysis/DMSO/2/PK-5_RGB_FITC.png]

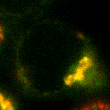

Supplement: S4 Data — This compressed folder contains the underlying numerical data and/or uncropped images used to generate the panels in Fig 5. (ZIP) [file pbio.3003736.s018.zip › S4 Data/Figure 5/F/Co-localization analysis/DMSO/2/PK-5_RGB_MERGE.jpg]

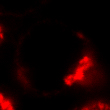

Supplement: S4 Data — This compressed folder contains the underlying numerical data and/or uncropped images used to generate the panels in Fig 5. (ZIP) [file pbio.3003736.s018.zip › S4 Data/Figure 5/F/Co-localization analysis/DMSO/2/PK-5_RGB_TRITC.jpg]

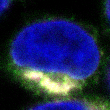

Supplement: S4 Data — This compressed folder contains the underlying numerical data and/or uncropped images used to generate the panels in Fig 5. (ZIP) [file pbio.3003736.s018.zip › S4 Data/Figure 5/F/Co-localization analysis/DMSO/3/PK-5_RGB.png]

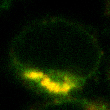

Supplement: S4 Data — This compressed folder contains the underlying numerical data and/or uncropped images used to generate the panels in Fig 5. (ZIP) [file pbio.3003736.s018.zip › S4 Data/Figure 5/F/Co-localization analysis/DMSO/3/PK-5_RGBMERGE.jpg]

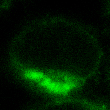

Supplement: S4 Data — This compressed folder contains the underlying numerical data and/or uncropped images used to generate the panels in Fig 5. (ZIP) [file pbio.3003736.s018.zip › S4 Data/Figure 5/F/Co-localization analysis/DMSO/3/PK-5_RGB_Cy5.jpg]

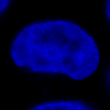

Supplement: S4 Data — This compressed folder contains the underlying numerical data and/or uncropped images used to generate the panels in Fig 5. (ZIP) [file pbio.3003736.s018.zip › S4 Data/Figure 5/F/Co-localization analysis/DMSO/3/PK-5_RGB_DAPI.png]

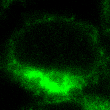

Supplement: S4 Data — This compressed folder contains the underlying numerical data and/or uncropped images used to generate the panels in Fig 5. (ZIP) [file pbio.3003736.s018.zip › S4 Data/Figure 5/F/Co-localization analysis/DMSO/3/PK-5_RGB_FITC.png]

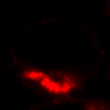

Supplement: S4 Data — This compressed folder contains the underlying numerical data and/or uncropped images used to generate the panels in Fig 5. (ZIP) [file pbio.3003736.s018.zip › S4 Data/Figure 5/F/Co-localization analysis/DMSO/3/PK-5_RGB_TRITC.jpg]

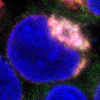

Supplement: S4 Data — This compressed folder contains the underlying numerical data and/or uncropped images used to generate the panels in Fig 5. (ZIP) [file pbio.3003736.s018.zip › S4 Data/Figure 5/F/Co-localization analysis/DMSO/4/PK-6_RGB.png]

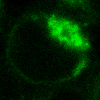

Supplement: S4 Data — This compressed folder contains the underlying numerical data and/or uncropped images used to generate the panels in Fig 5. (ZIP) [file pbio.3003736.s018.zip › S4 Data/Figure 5/F/Co-localization analysis/DMSO/4/PK-6_RGB_Cy5.jpg]

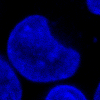

Supplement: S4 Data — This compressed folder contains the underlying numerical data and/or uncropped images used to generate the panels in Fig 5. (ZIP) [file pbio.3003736.s018.zip › S4 Data/Figure 5/F/Co-localization analysis/DMSO/4/PK-6_RGB_DAPI.png]

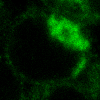

Supplement: S4 Data — This compressed folder contains the underlying numerical data and/or uncropped images used to generate the panels in Fig 5. (ZIP) [file pbio.3003736.s018.zip › S4 Data/Figure 5/F/Co-localization analysis/DMSO/4/PK-6_RGB_FITC.png]

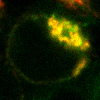

Supplement: S4 Data — This compressed folder contains the underlying numerical data and/or uncropped images used to generate the panels in Fig 5. (ZIP) [file pbio.3003736.s018.zip › S4 Data/Figure 5/F/Co-localization analysis/DMSO/4/PK-6_RGB_MERGE.jpg]

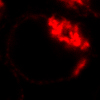

Supplement: S4 Data — This compressed folder contains the underlying numerical data and/or uncropped images used to generate the panels in Fig 5. (ZIP) [file pbio.3003736.s018.zip › S4 Data/Figure 5/F/Co-localization analysis/DMSO/4/PK-6_RGB_TRITC.jpg]

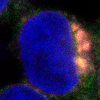

Supplement: S4 Data — This compressed folder contains the underlying numerical data and/or uncropped images used to generate the panels in Fig 5. (ZIP) [file pbio.3003736.s018.zip › S4 Data/Figure 5/F/Co-localization analysis/DMSO/5/PK-6_RGB.png]

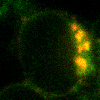

Supplement: S4 Data — This compressed folder contains the underlying numerical data and/or uncropped images used to generate the panels in Fig 5. (ZIP) [file pbio.3003736.s018.zip › S4 Data/Figure 5/F/Co-localization analysis/DMSO/5/PK-6_RGBMERGE.jpg]

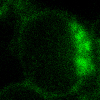

Supplement: S4 Data — This compressed folder contains the underlying numerical data and/or uncropped images used to generate the panels in Fig 5. (ZIP) [file pbio.3003736.s018.zip › S4 Data/Figure 5/F/Co-localization analysis/DMSO/5/PK-6_RGB_Cy5.jpg]

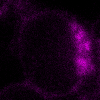

Supplement: S4 Data — This compressed folder contains the underlying numerical data and/or uncropped images used to generate the panels in Fig 5. (ZIP) [file pbio.3003736.s018.zip › S4 Data/Figure 5/F/Co-localization analysis/DMSO/5/PK-6_RGB_Cy5.png]

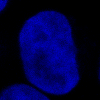

Supplement: S4 Data — This compressed folder contains the underlying numerical data and/or uncropped images used to generate the panels in Fig 5. (ZIP) [file pbio.3003736.s018.zip › S4 Data/Figure 5/F/Co-localization analysis/DMSO/5/PK-6_RGB_DAPI.png]

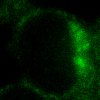

Supplement: S4 Data — This compressed folder contains the underlying numerical data and/or uncropped images used to generate the panels in Fig 5. (ZIP) [file pbio.3003736.s018.zip › S4 Data/Figure 5/F/Co-localization analysis/DMSO/5/PK-6_RGB_FITC.png]

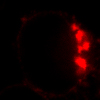

Supplement: S4 Data — This compressed folder contains the underlying numerical data and/or uncropped images used to generate the panels in Fig 5. (ZIP) [file pbio.3003736.s018.zip › S4 Data/Figure 5/F/Co-localization analysis/DMSO/5/PK-6_RGB_TRITC.jpg]

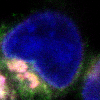

Supplement: S4 Data — This compressed folder contains the underlying numerical data and/or uncropped images used to generate the panels in Fig 5. (ZIP) [file pbio.3003736.s018.zip › S4 Data/Figure 5/F/Co-localization analysis/DMSO/6/PK-6_RGB.png]

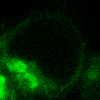

Supplement: S4 Data — This compressed folder contains the underlying numerical data and/or uncropped images used to generate the panels in Fig 5. (ZIP) [file pbio.3003736.s018.zip › S4 Data/Figure 5/F/Co-localization analysis/DMSO/6/PK-6_RGB_Cy5.jpg]

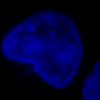

Supplement: S4 Data — This compressed folder contains the underlying numerical data and/or uncropped images used to generate the panels in Fig 5. (ZIP) [file pbio.3003736.s018.zip › S4 Data/Figure 5/F/Co-localization analysis/DMSO/6/PK-6_RGB_DAPI.png]

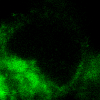

Supplement: S4 Data — This compressed folder contains the underlying numerical data and/or uncropped images used to generate the panels in Fig 5. (ZIP) [file pbio.3003736.s018.zip › S4 Data/Figure 5/F/Co-localization analysis/DMSO/6/PK-6_RGB_FITC.png]

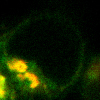

Supplement: S4 Data — This compressed folder contains the underlying numerical data and/or uncropped images used to generate the panels in Fig 5. (ZIP) [file pbio.3003736.s018.zip › S4 Data/Figure 5/F/Co-localization analysis/DMSO/6/PK-6_RGB_MERGE.jpg]

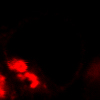

Supplement: S4 Data — This compressed folder contains the underlying numerical data and/or uncropped images used to generate the panels in Fig 5. (ZIP) [file pbio.3003736.s018.zip › S4 Data/Figure 5/F/Co-localization analysis/DMSO/6/PK-6_RGB_TRITC.jpg]

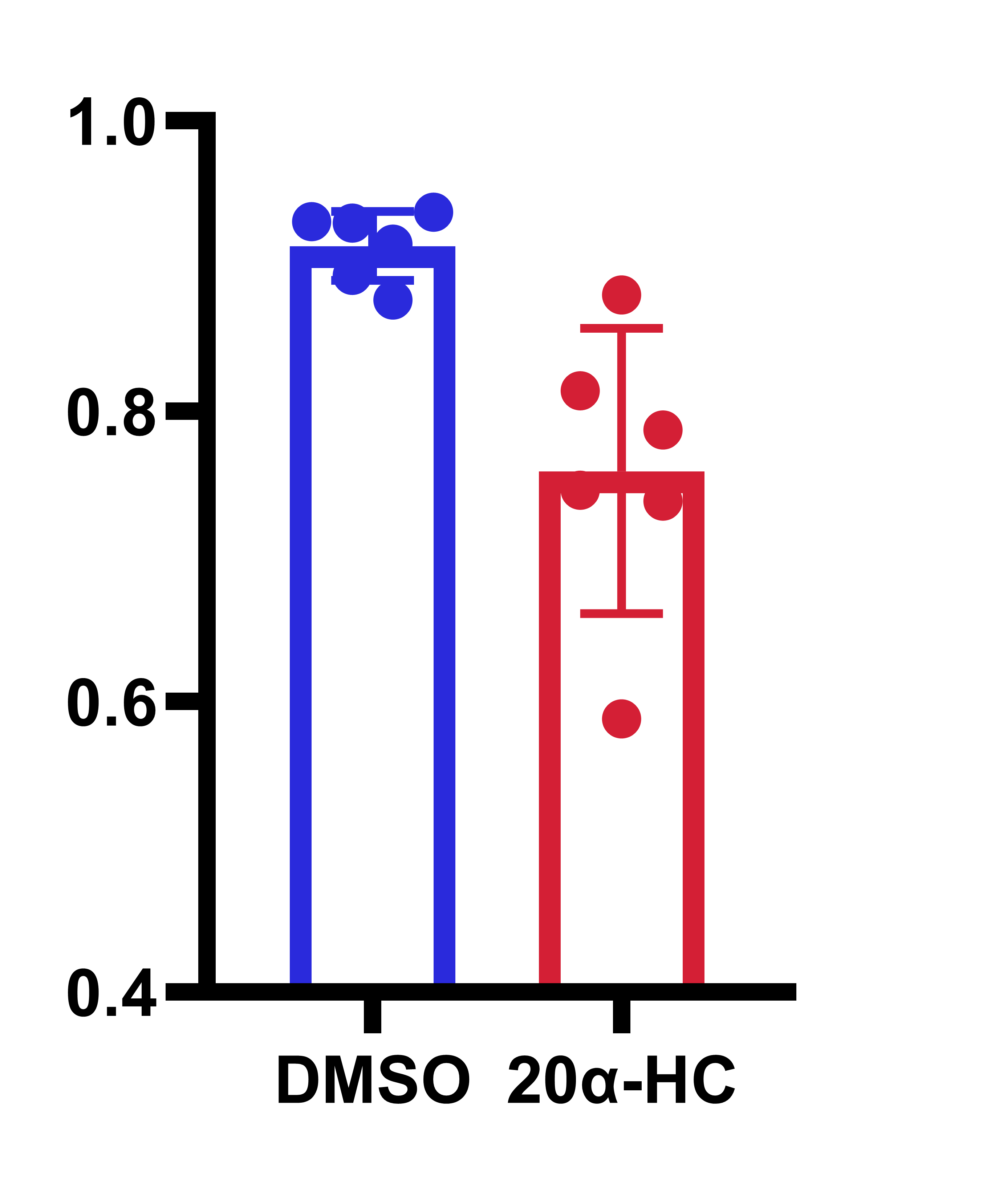

Supplement: S4 Data — This compressed folder contains the underlying numerical data and/or uncropped images used to generate the panels in Fig 5. (ZIP) [file pbio.3003736.s018.zip › S4 Data/Figure 5/F/Data 1.tif]

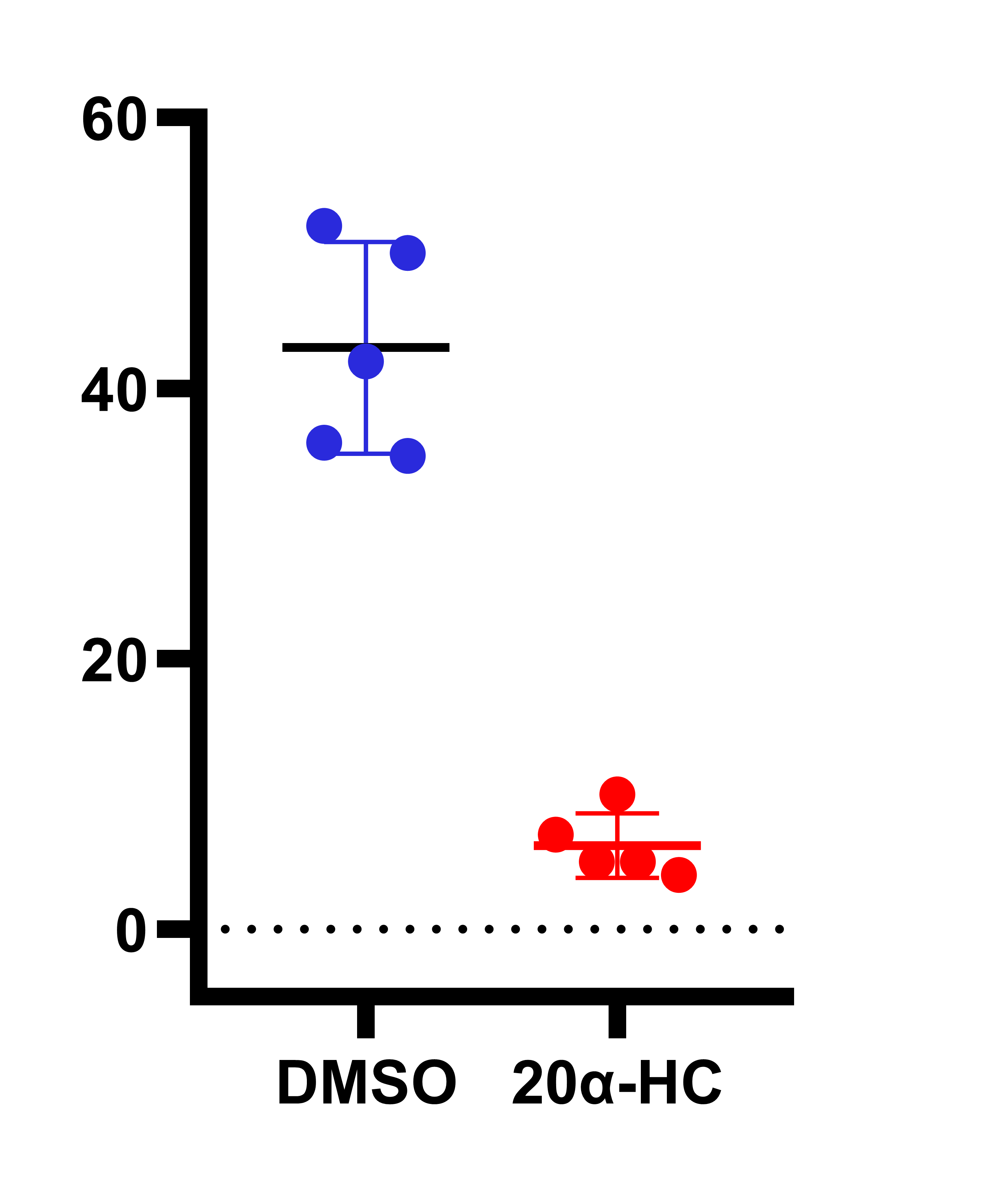

Supplement: S4 Data — This compressed folder contains the underlying numerical data and/or uncropped images used to generate the panels in Fig 5. (ZIP) [file pbio.3003736.s018.zip › S4 Data/Figure 5/G/DMV.tif]

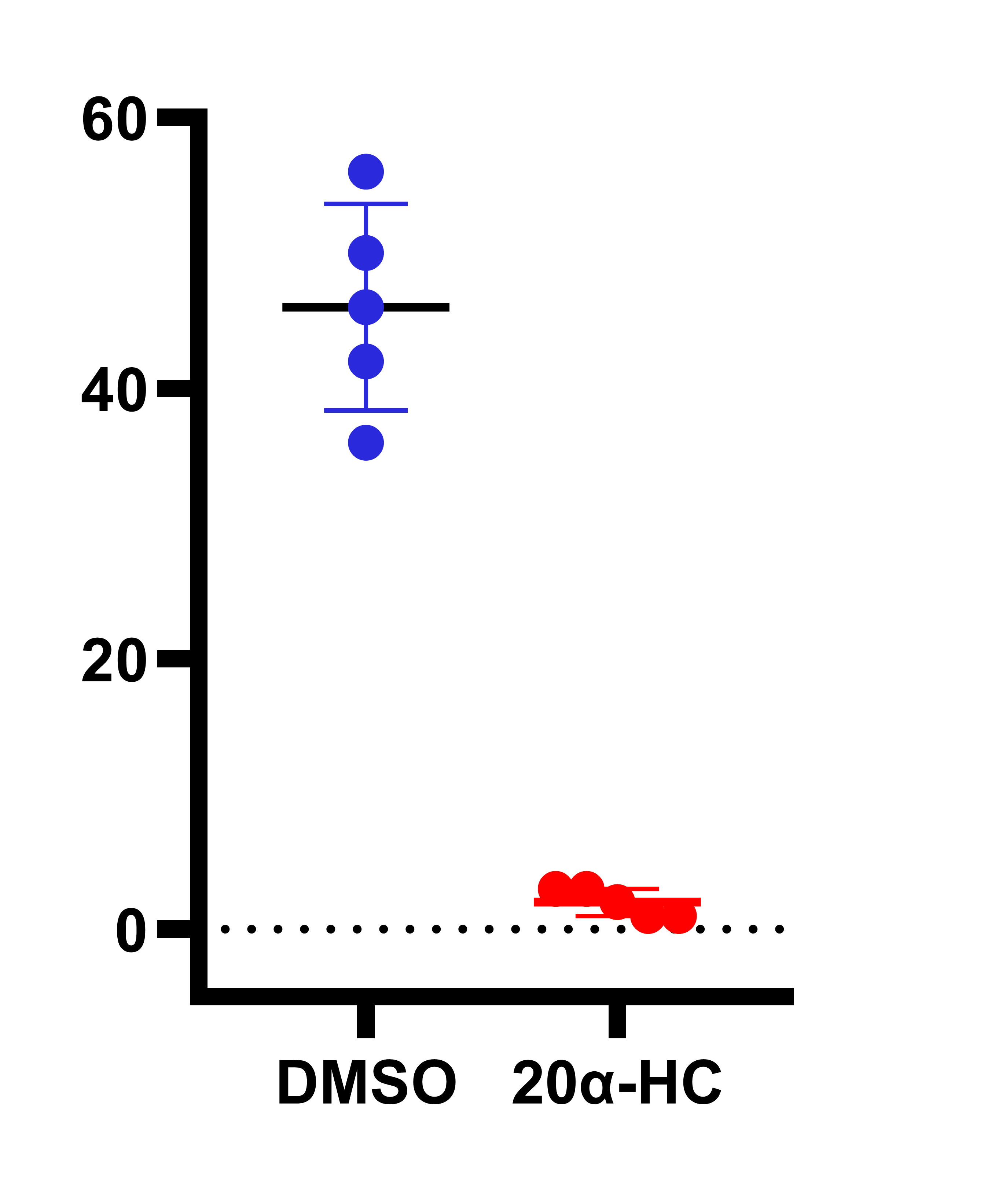

Supplement: S4 Data — This compressed folder contains the underlying numerical data and/or uncropped images used to generate the panels in Fig 5. (ZIP) [file pbio.3003736.s018.zip › S4 Data/Figure 5/H/pk-20HC-DMV.tif]

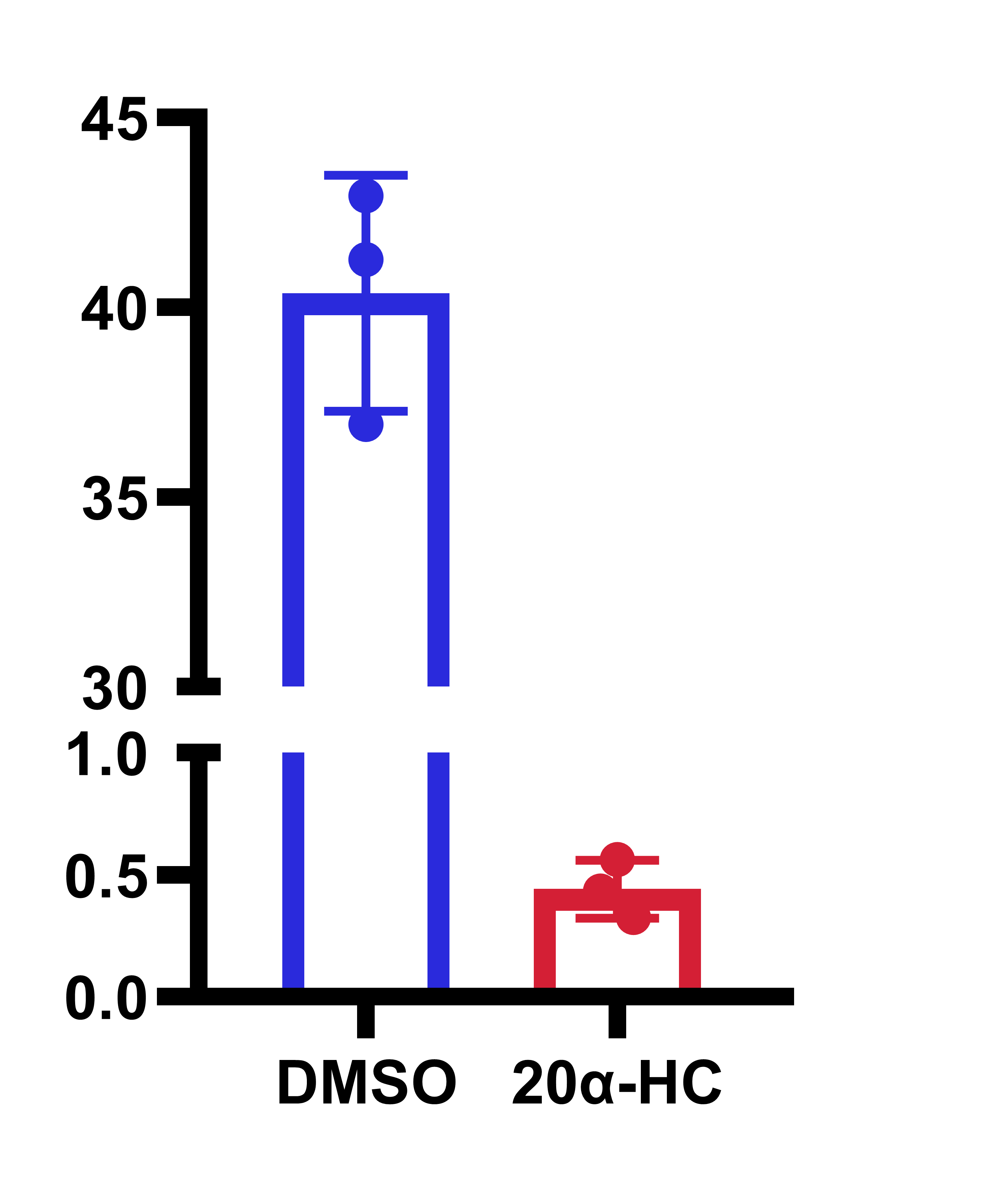

Supplement: S4 Data — This compressed folder contains the underlying numerical data and/or uncropped images used to generate the panels in Fig 5. (ZIP) [file pbio.3003736.s018.zip › S4 Data/Figure 5/I/CACO2-20HC-229E/IFA/229e-20HC-POSITIVE-CELLS.tif]

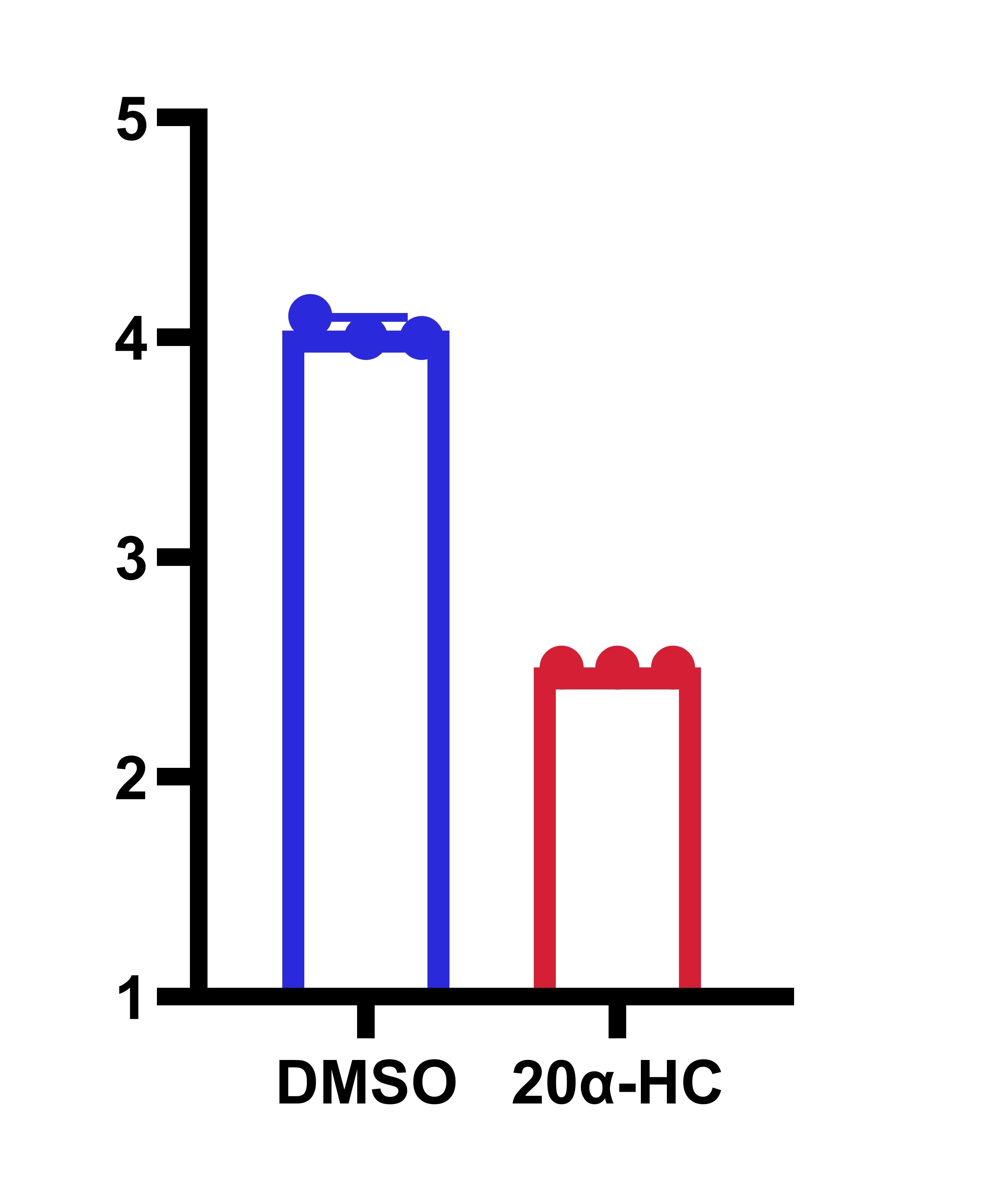

Supplement: S4 Data — This compressed folder contains the underlying numerical data and/or uncropped images used to generate the panels in Fig 5. (ZIP) [file pbio.3003736.s018.zip › S4 Data/Figure 5/I/CACO2-20HC-229E/TITER/229e-20HC-TITER.tif]

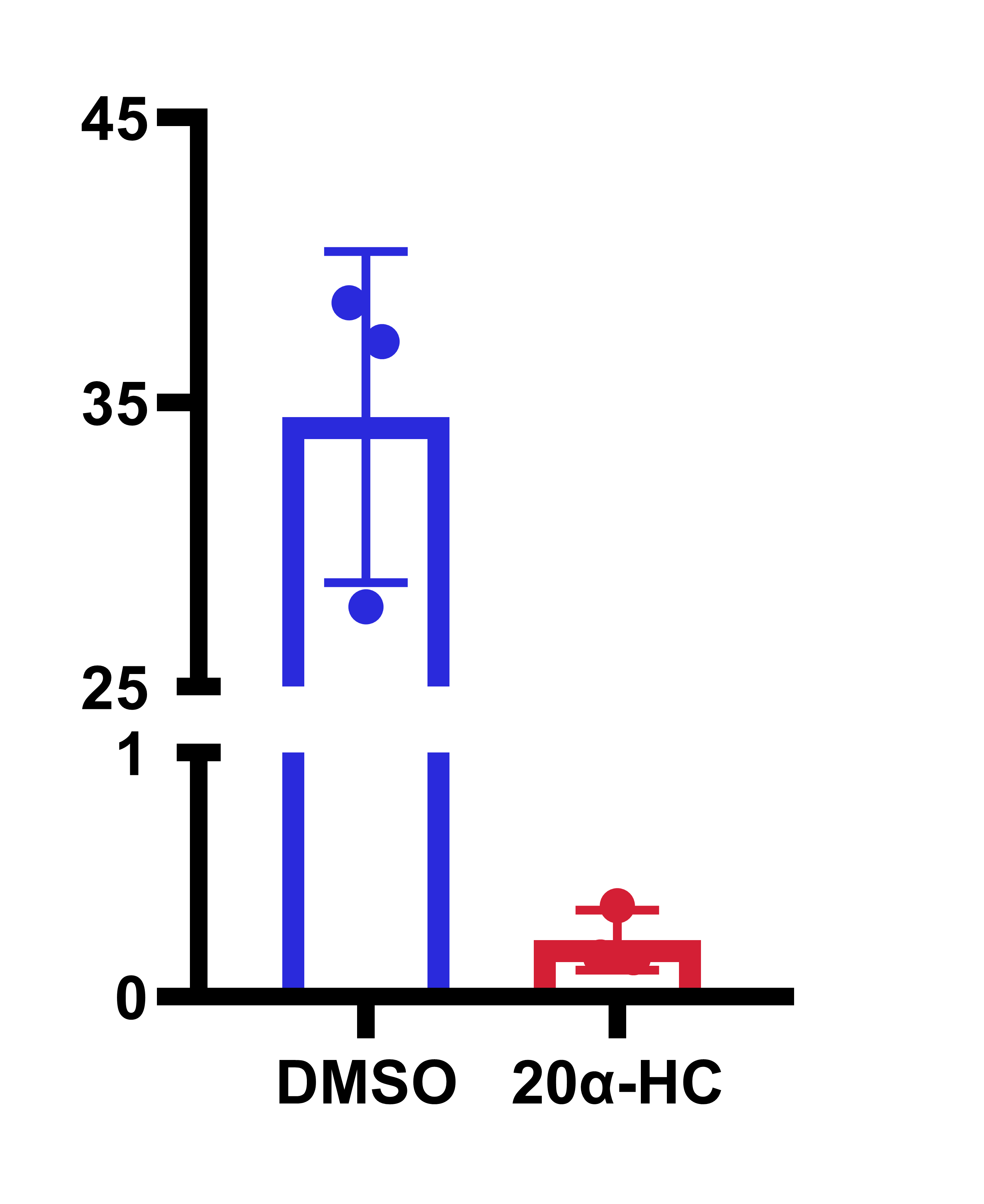

Supplement: S4 Data — This compressed folder contains the underlying numerical data and/or uncropped images used to generate the panels in Fig 5. (ZIP) [file pbio.3003736.s018.zip › S4 Data/Figure 5/J/IFA-0.01MOI-24H/SARS-2-20HC-POSITIVE-CELLS.tif]

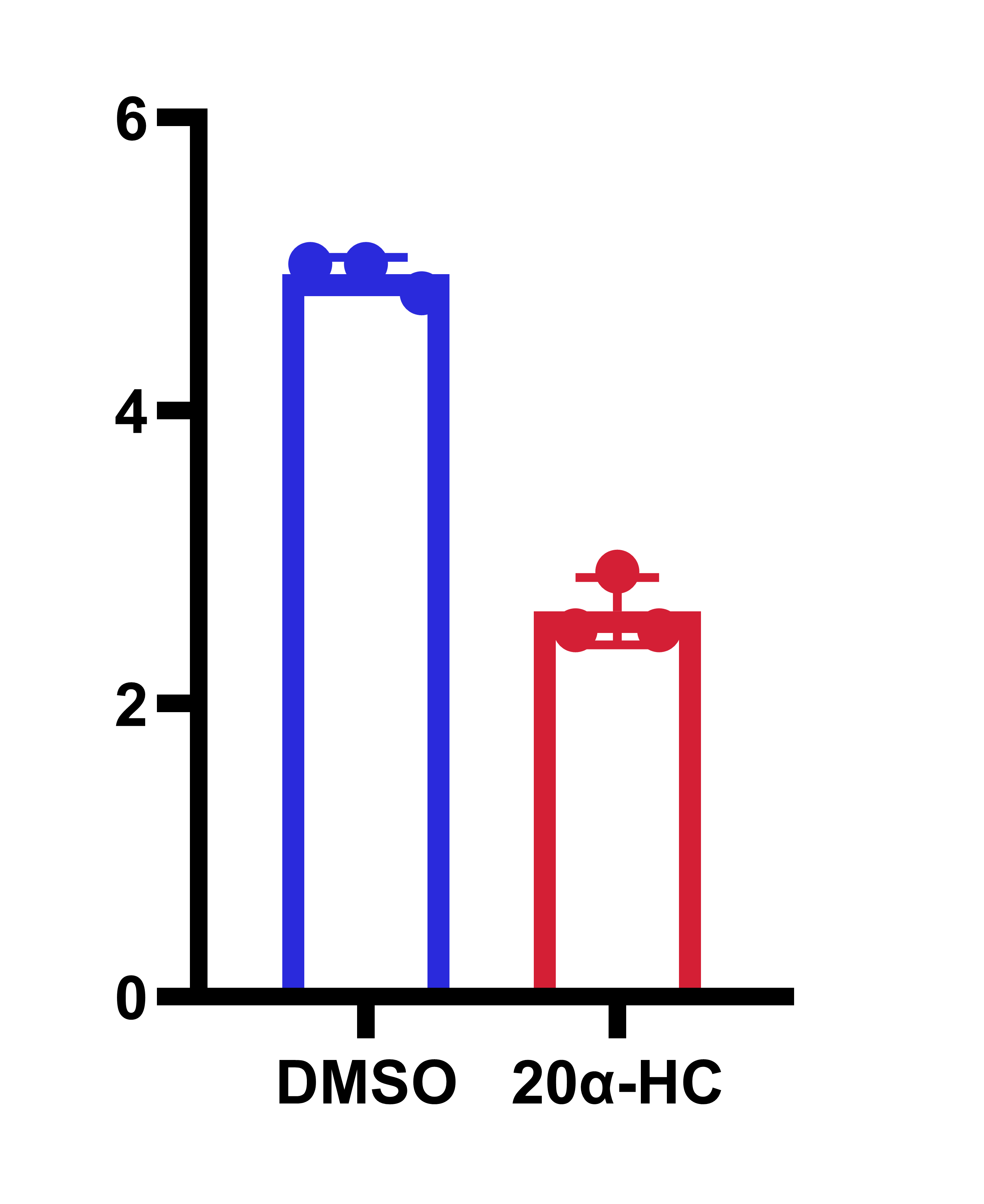

Supplement: S4 Data — This compressed folder contains the underlying numerical data and/or uncropped images used to generate the panels in Fig 5. (ZIP) [file pbio.3003736.s018.zip › S4 Data/Figure 5/J/sars-2-TITER/sars-2-TITER.tif]

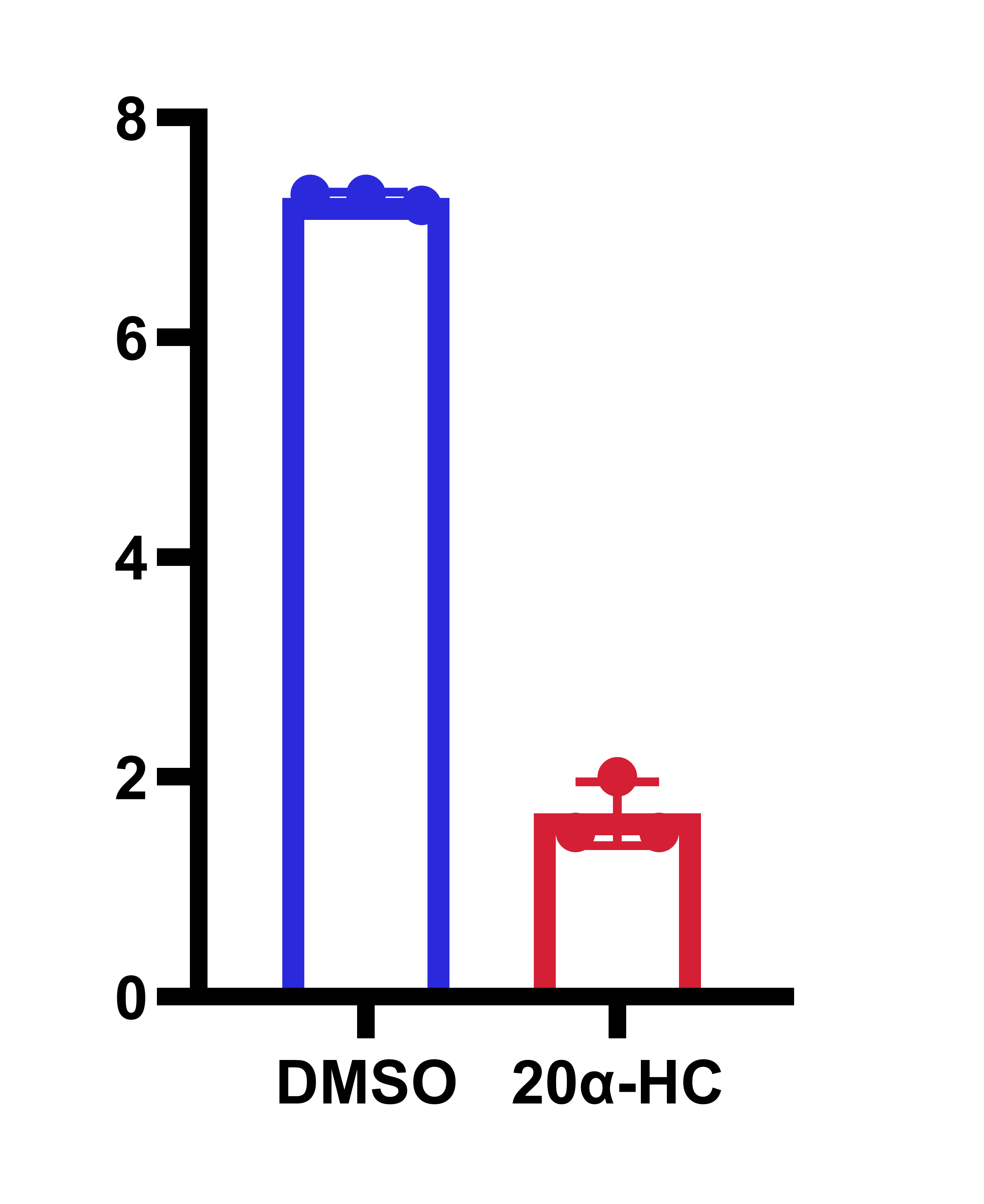

Supplement: S4 Data — This compressed folder contains the underlying numerical data and/or uncropped images used to generate the panels in Fig 5. (ZIP) [file pbio.3003736.s018.zip › S4 Data/Figure 5/K/L929-TITER/mhv-30um-0.1moi-18h.tif]

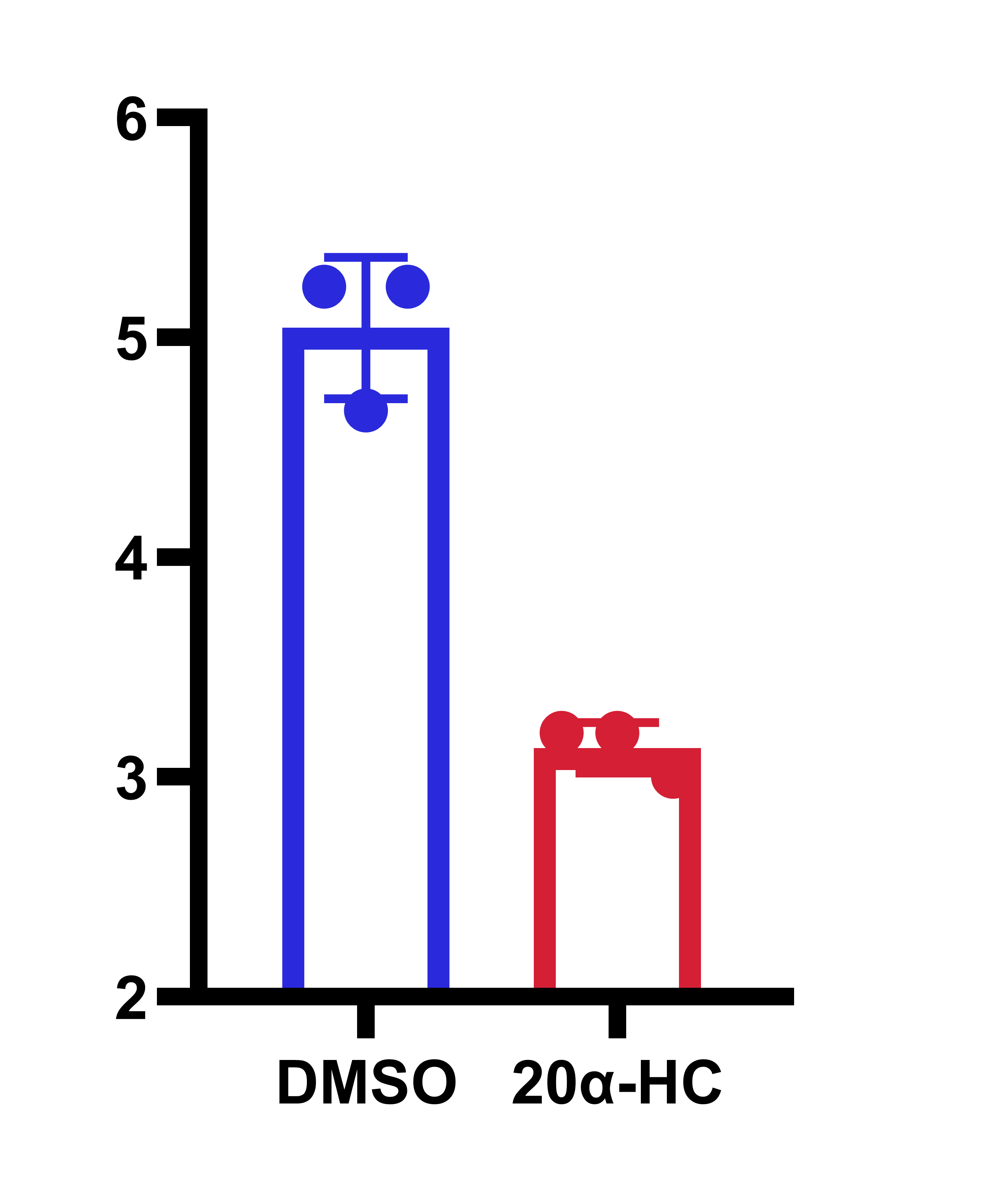

Supplement: S4 Data — This compressed folder contains the underlying numerical data and/or uncropped images used to generate the panels in Fig 5. (ZIP) [file pbio.3003736.s018.zip › S4 Data/Figure 5/K/PDCOV-TITER/pdcov-5moi-30um.tif]

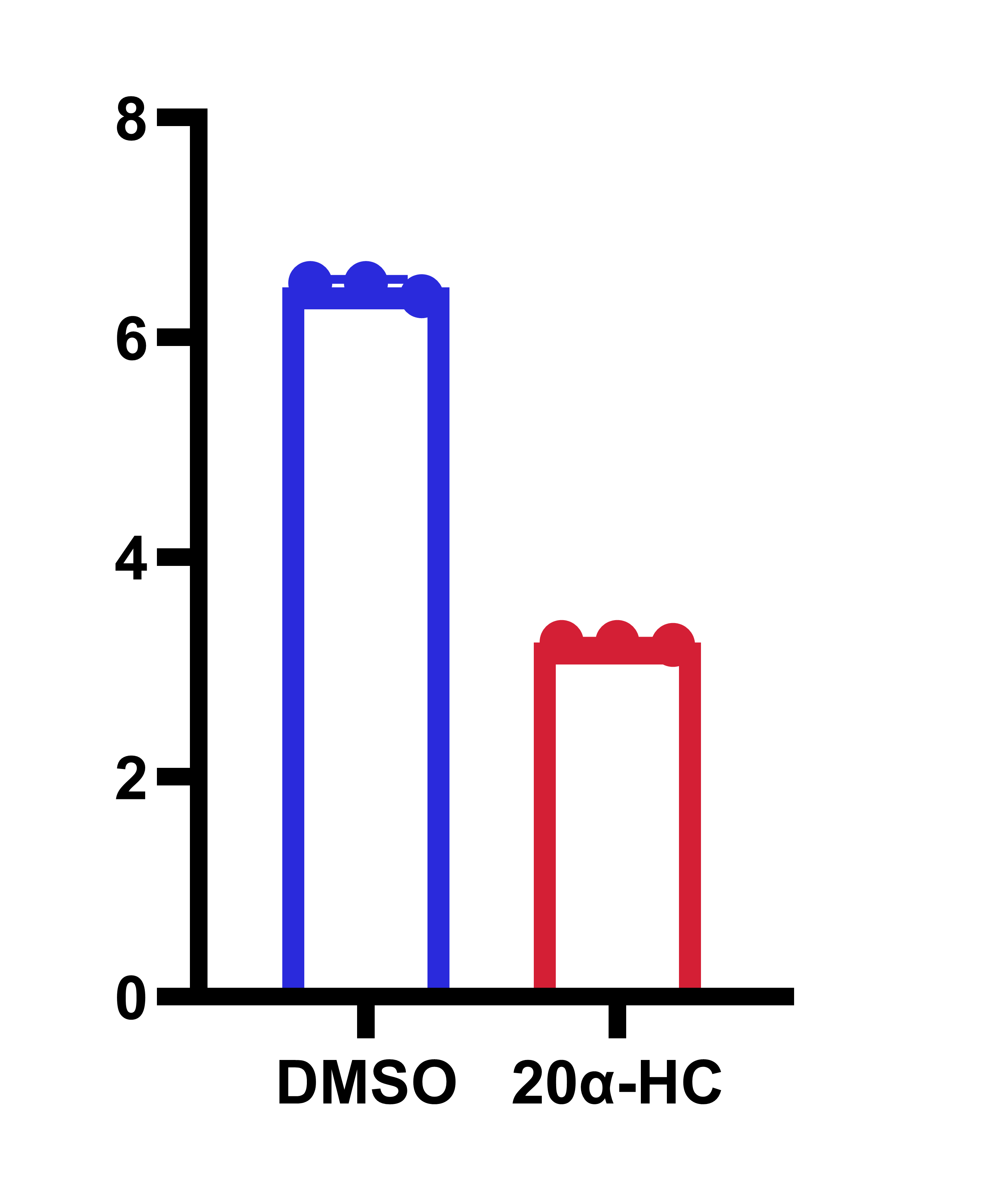

Supplement: S4 Data — This compressed folder contains the underlying numerical data and/or uncropped images used to generate the panels in Fig 5. (ZIP) [file pbio.3003736.s018.zip › S4 Data/Figure 5/K/VERO-PEDV/vero-dr13-30um.tif]

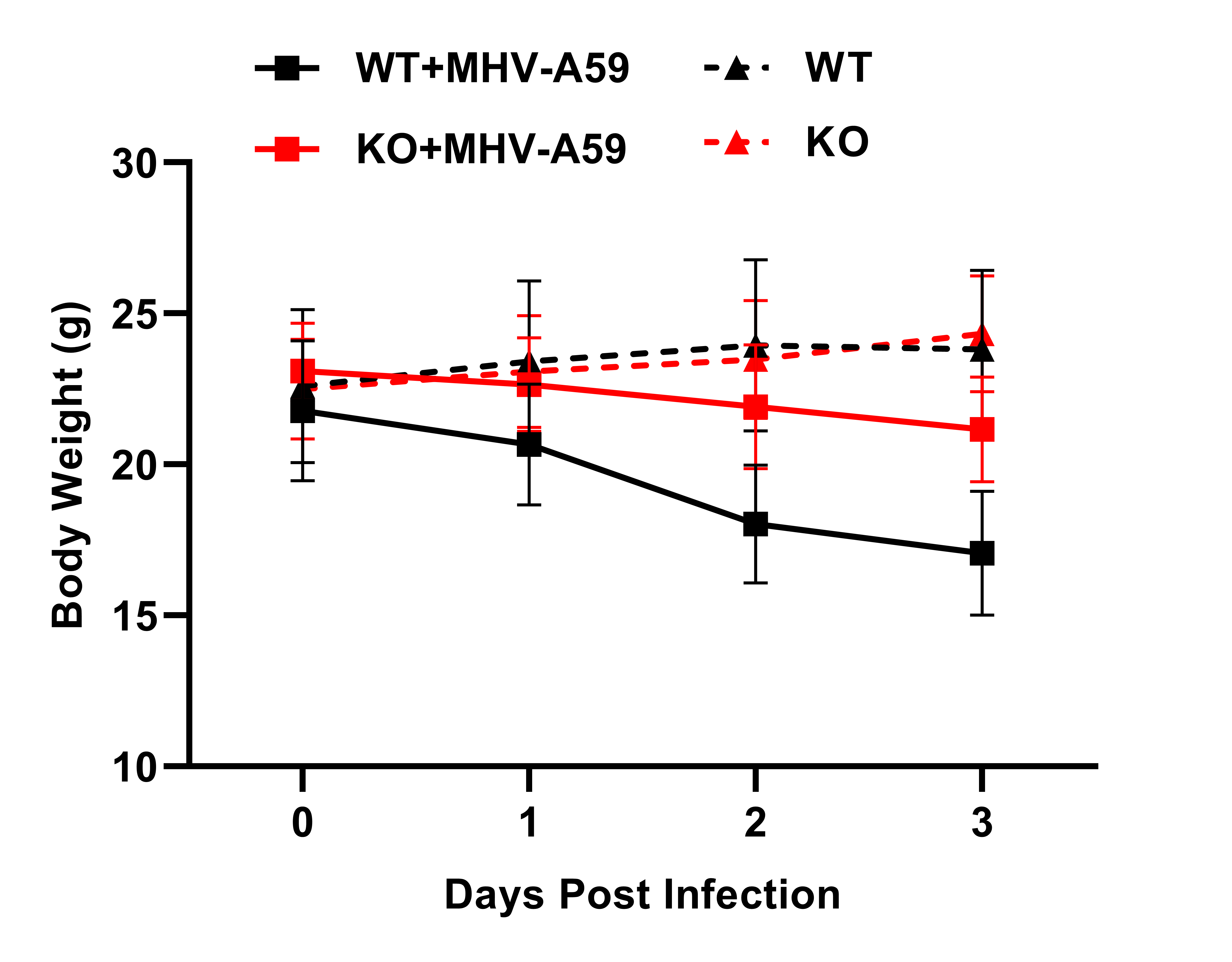

Supplement: S5 Data — This compressed folder contains the underlying numerical data and/or uncropped images used to generate the panels in Figs 6 and S1–S6, and S11. (ZIP) [file pbio.3003736.s019.zip › S5 Data/Figure 6/A/body weight change.tif]

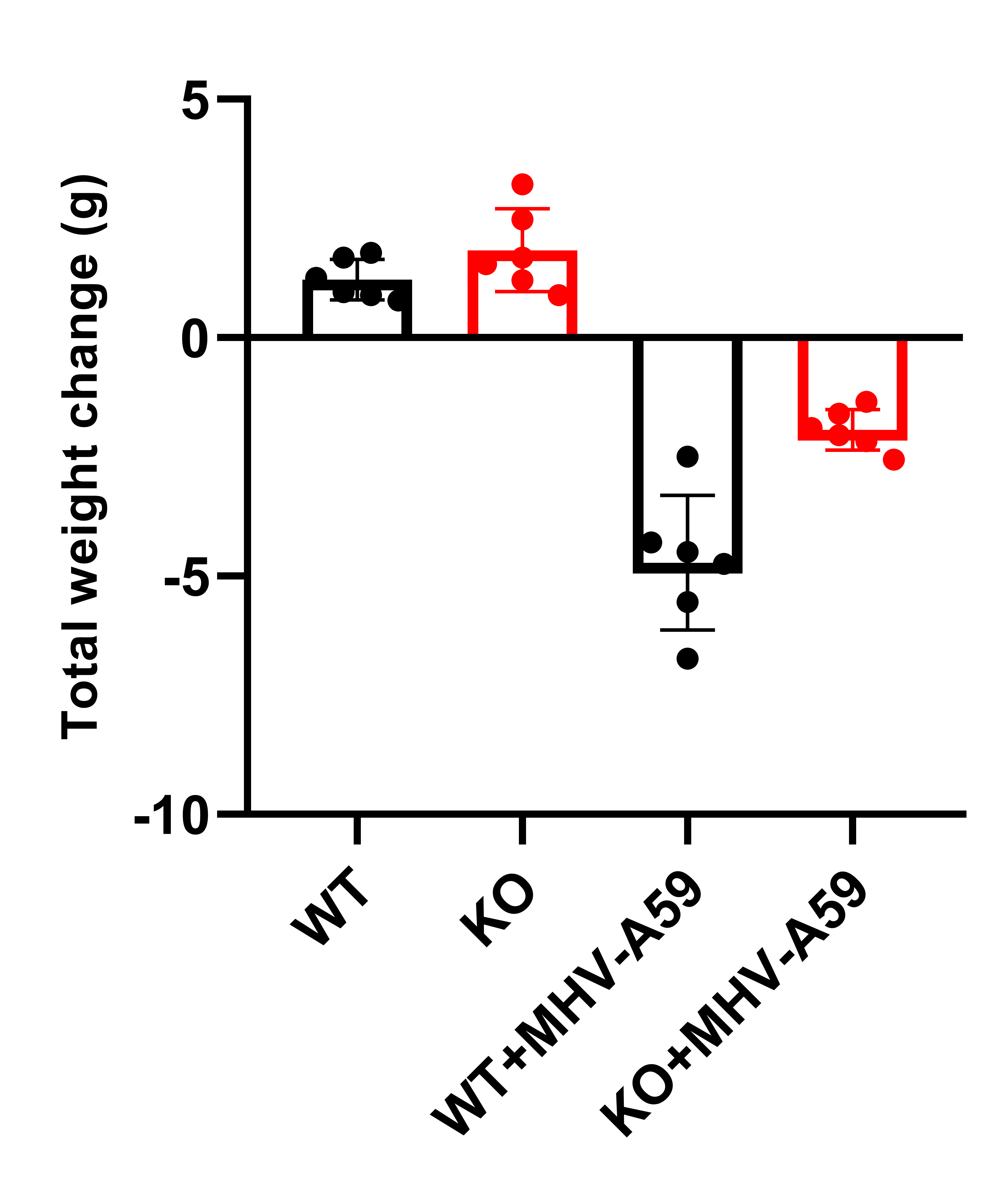

Supplement: S5 Data — This compressed folder contains the underlying numerical data and/or uncropped images used to generate the panels in Figs 6 and S1–S6, and S11. (ZIP) [file pbio.3003736.s019.zip › S5 Data/Figure 6/B/Overall weight change.tif]

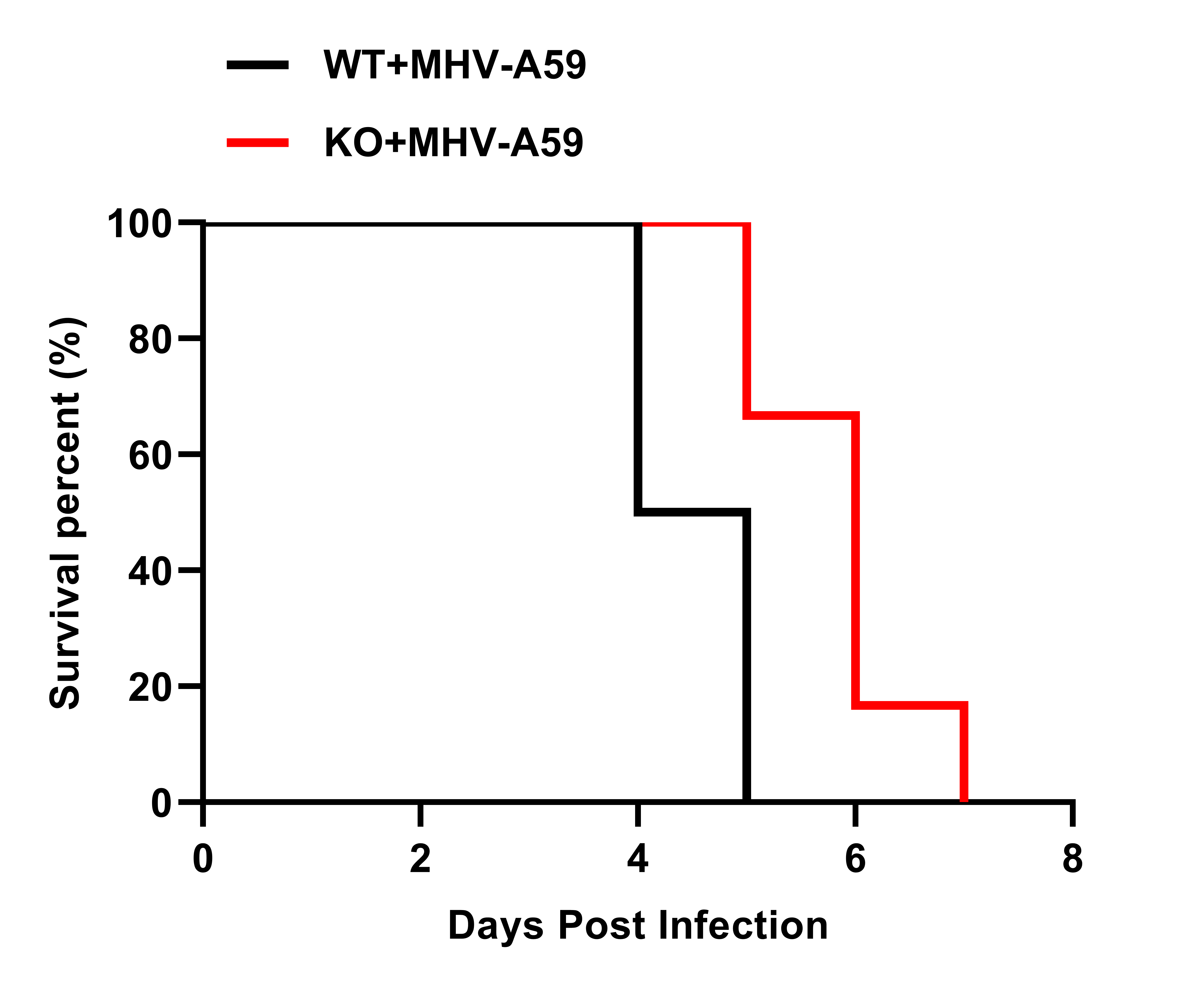

Supplement: S5 Data — This compressed folder contains the underlying numerical data and/or uncropped images used to generate the panels in Figs 6 and S1–S6, and S11. (ZIP) [file pbio.3003736.s019.zip › S5 Data/Figure 6/C/survival rate.tif]

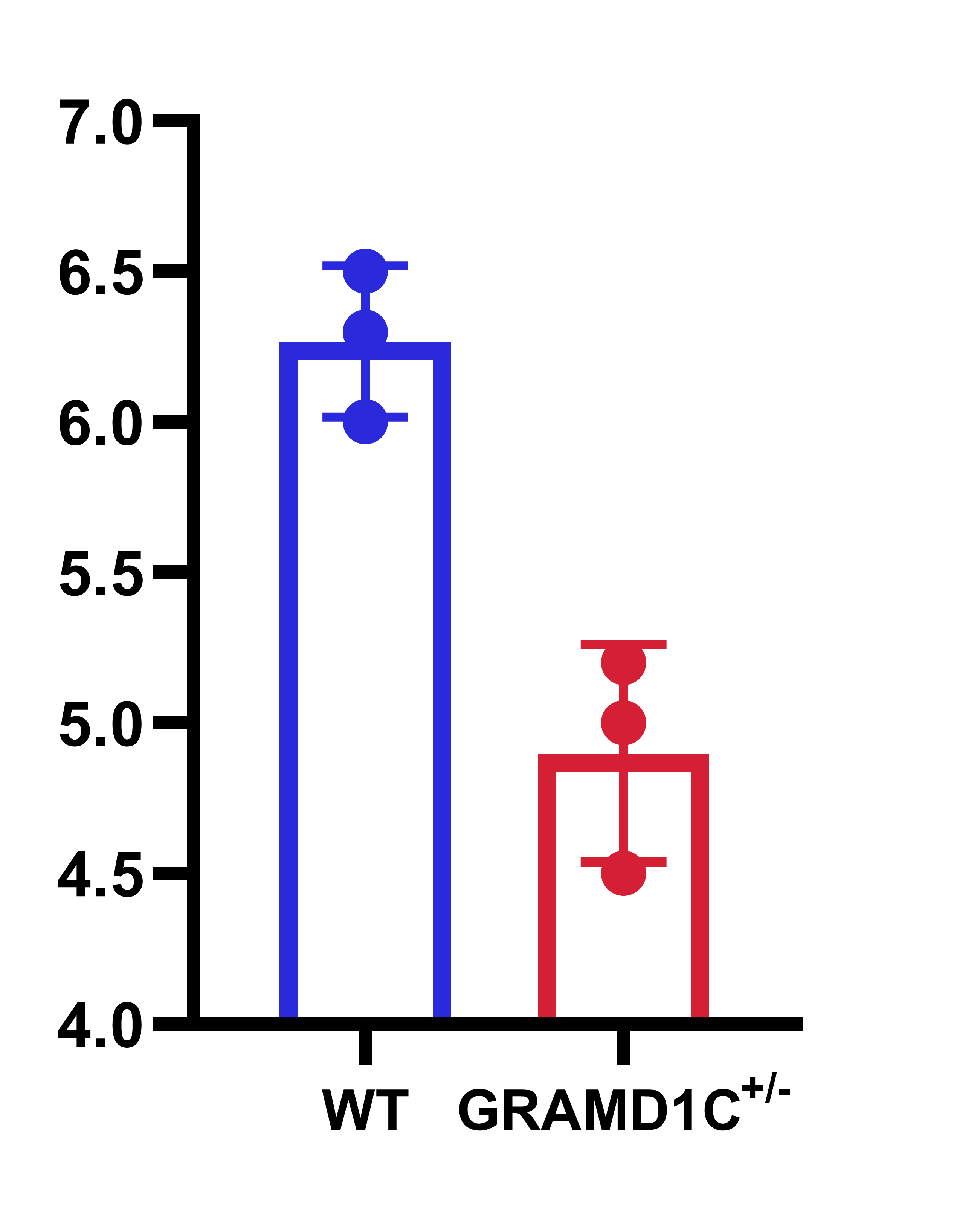

Supplement: S5 Data — This compressed folder contains the underlying numerical data and/or uncropped images used to generate the panels in Figs 6 and S1–S6, and S11. (ZIP) [file pbio.3003736.s019.zip › S5 Data/Figure 6/G/liver-titer.tif]

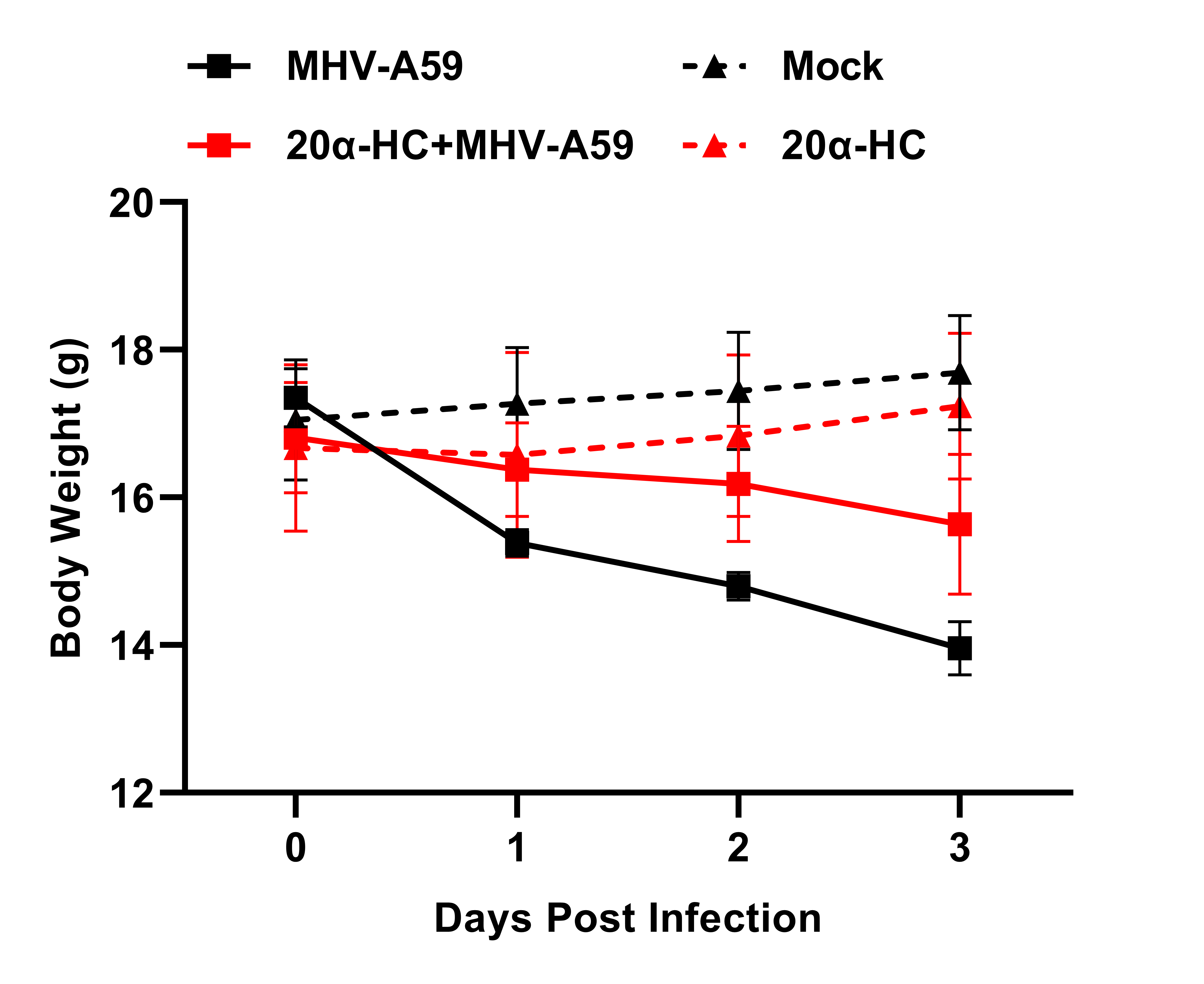

Supplement: S5 Data — This compressed folder contains the underlying numerical data and/or uncropped images used to generate the panels in Figs 6 and S1–S6, and S11. (ZIP) [file pbio.3003736.s019.zip › S5 Data/Figure 6/I/WEIGHT CHANGE.tif]

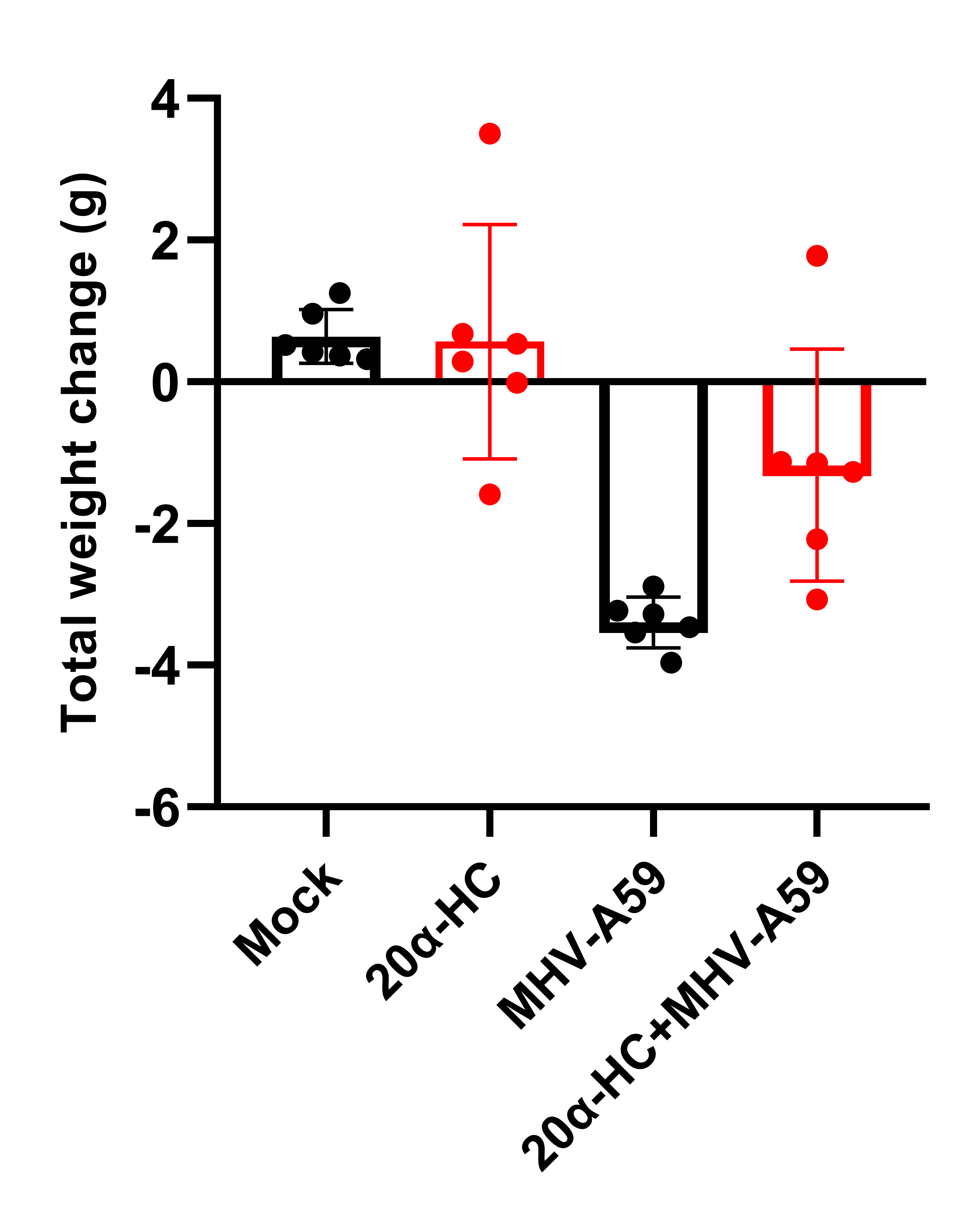

Supplement: S5 Data — This compressed folder contains the underlying numerical data and/or uncropped images used to generate the panels in Figs 6 and S1–S6, and S11. (ZIP) [file pbio.3003736.s019.zip › S5 Data/Figure 6/J/TOTAL-WEIGHT CHANGE.tif]

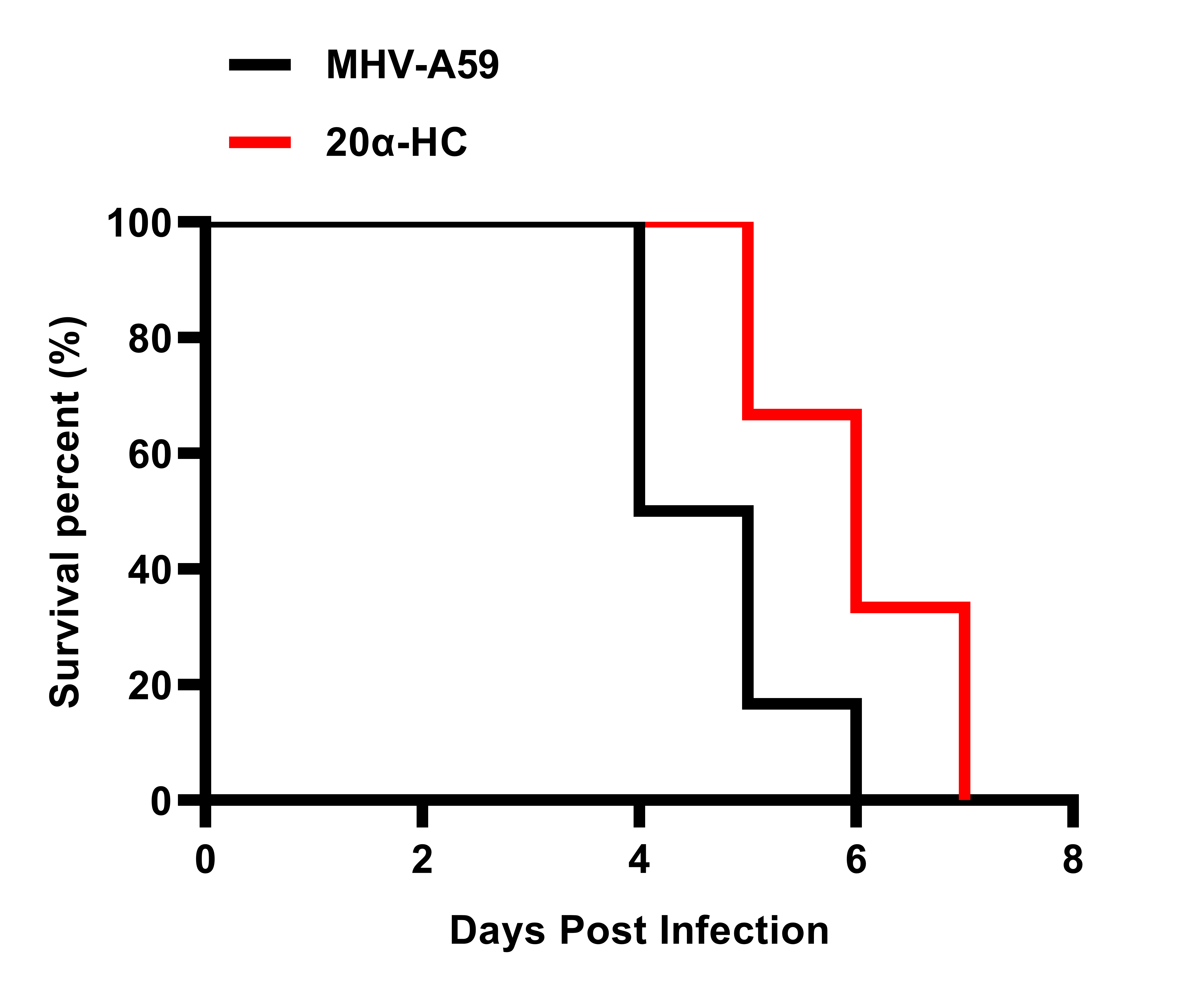

Supplement: S5 Data — This compressed folder contains the underlying numerical data and/or uncropped images used to generate the panels in Figs 6 and S1–S6, and S11. (ZIP) [file pbio.3003736.s019.zip › S5 Data/Figure 6/K/survival rate.tif]

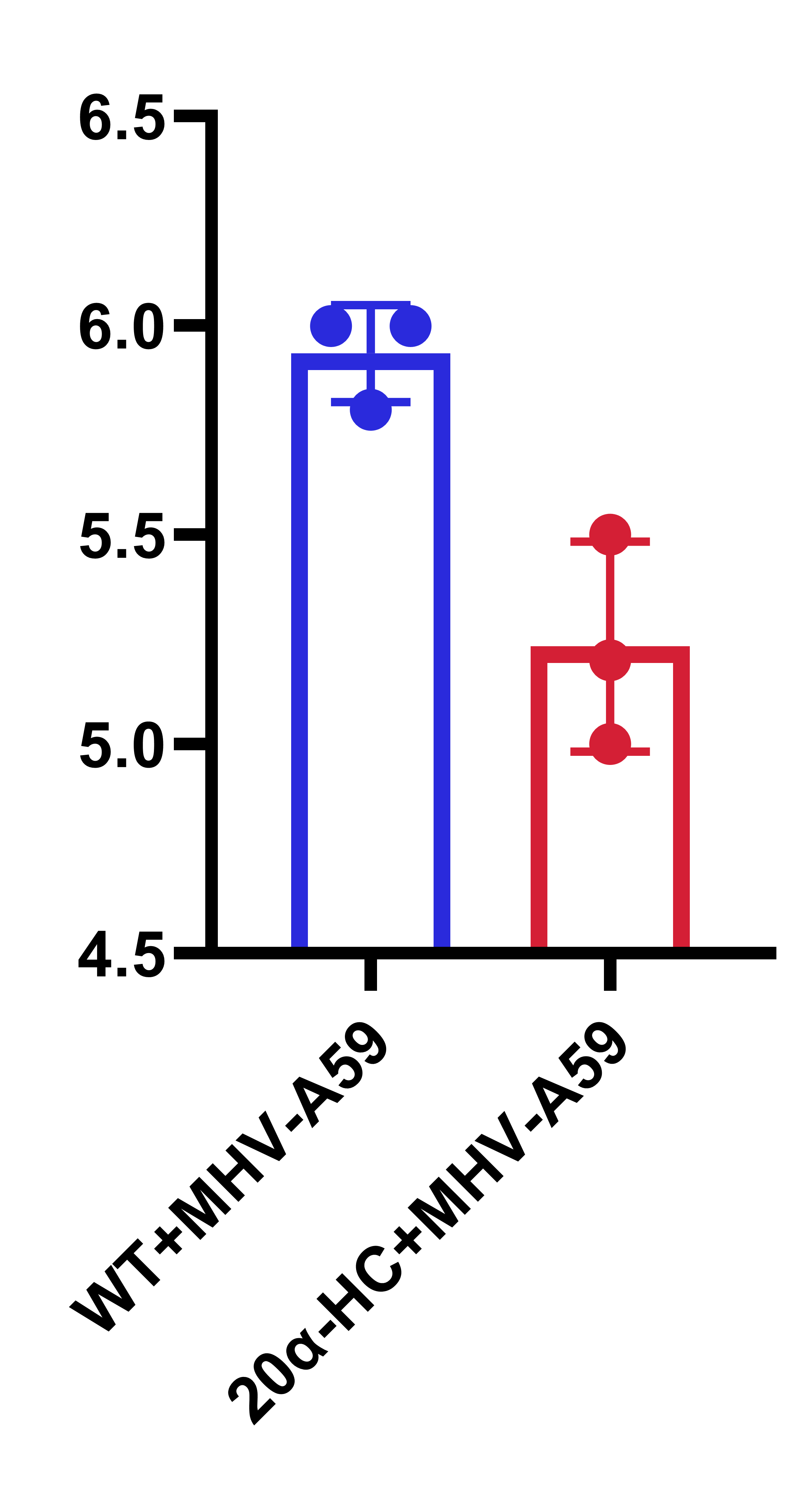

Supplement: S5 Data — This compressed folder contains the underlying numerical data and/or uncropped images used to generate the panels in Figs 6 and S1–S6, and S11. (ZIP) [file pbio.3003736.s019.zip › S5 Data/Figure 6/O/LIVER-TITER.tif]

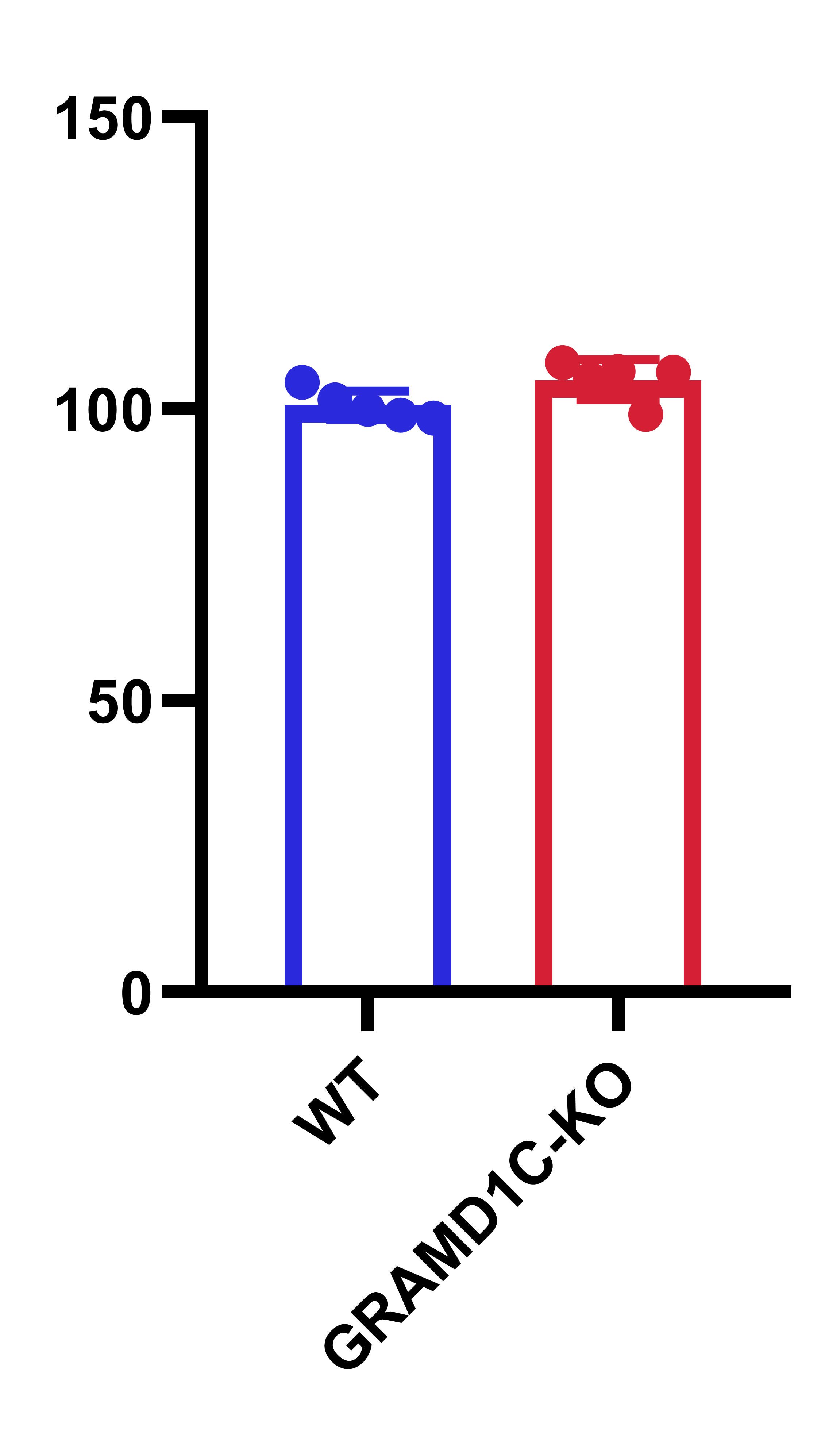

Supplement: S5 Data — This compressed folder contains the underlying numerical data and/or uncropped images used to generate the panels in Figs 6 and S1–S6, and S11. (ZIP) [file pbio.3003736.s019.zip › S5 Data/Supporting Information/Supporting Information fig1/A/PK-KO-Cell Viability.tif]

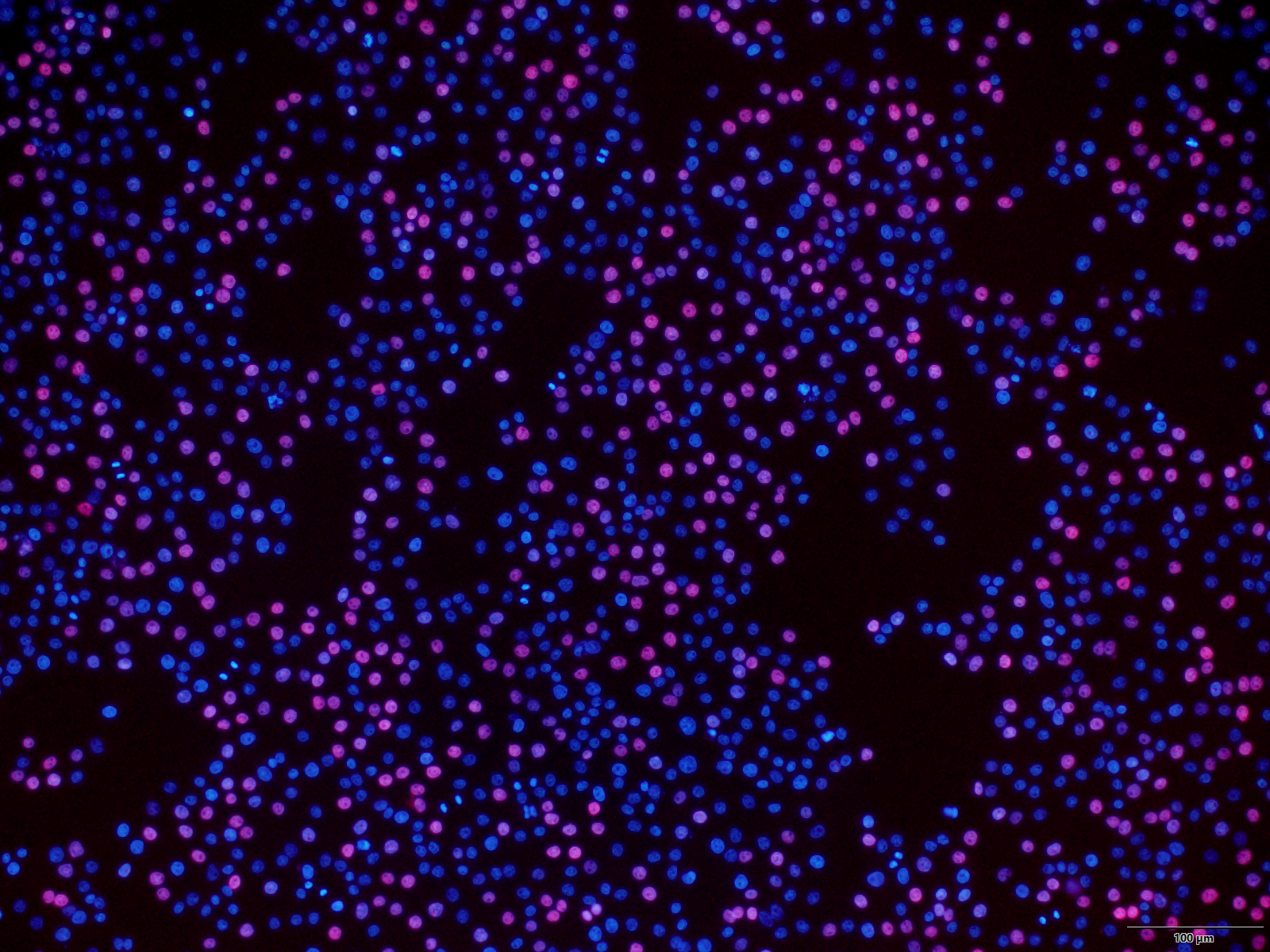

Supplement: S5 Data — This compressed folder contains the underlying numerical data and/or uncropped images used to generate the panels in Figs 6 and S1–S6, and S11. (ZIP) [file pbio.3003736.s019.zip › S5 Data/Supporting Information/Supporting Information fig1/B/EDU/18-edu-5.png]

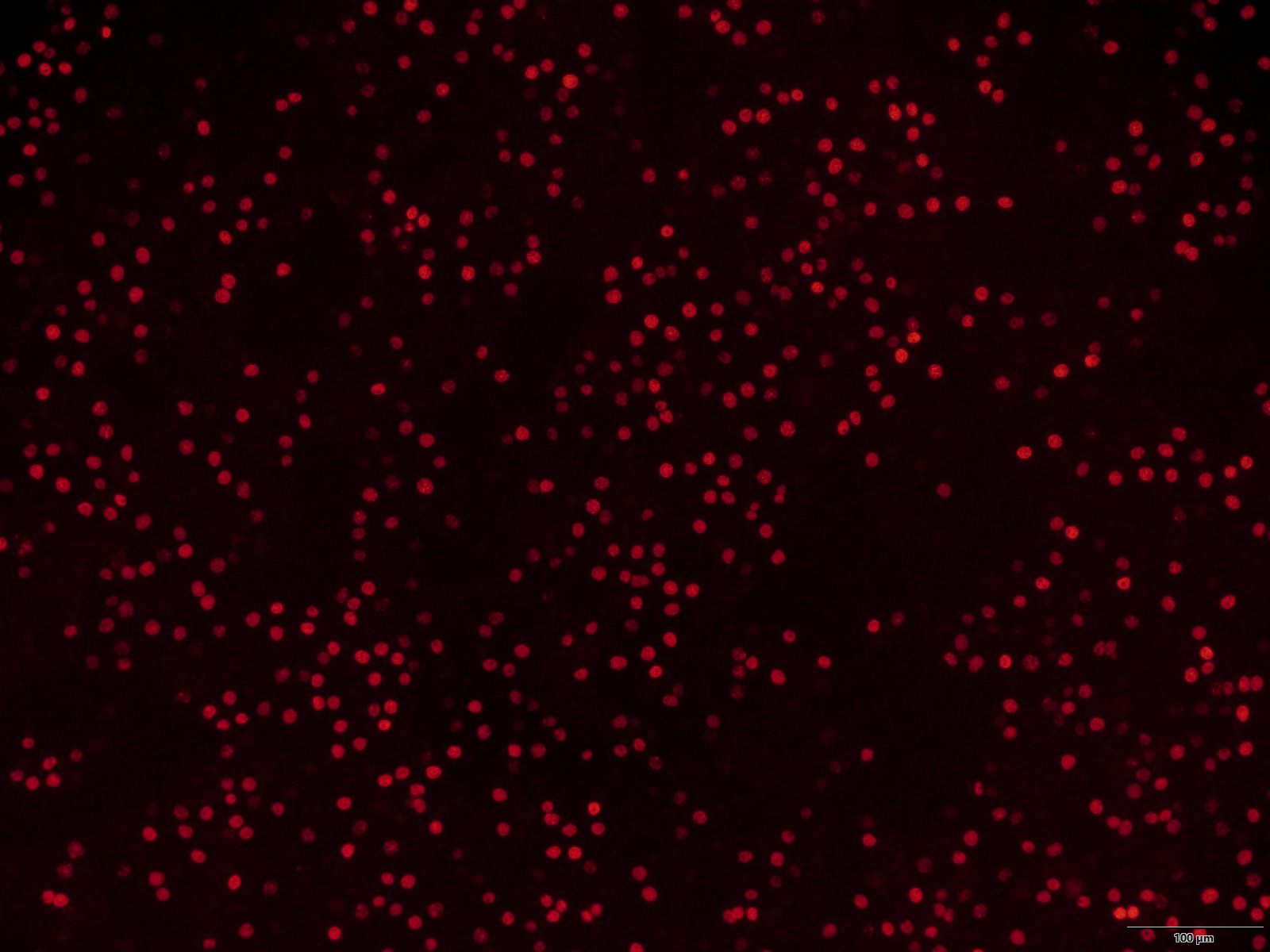

Supplement: S5 Data — This compressed folder contains the underlying numerical data and/or uncropped images used to generate the panels in Figs 6 and S1–S6, and S11. (ZIP) [file pbio.3003736.s019.zip › S5 Data/Supporting Information/Supporting Information fig1/B/EDU/18-edu-594-5.png]

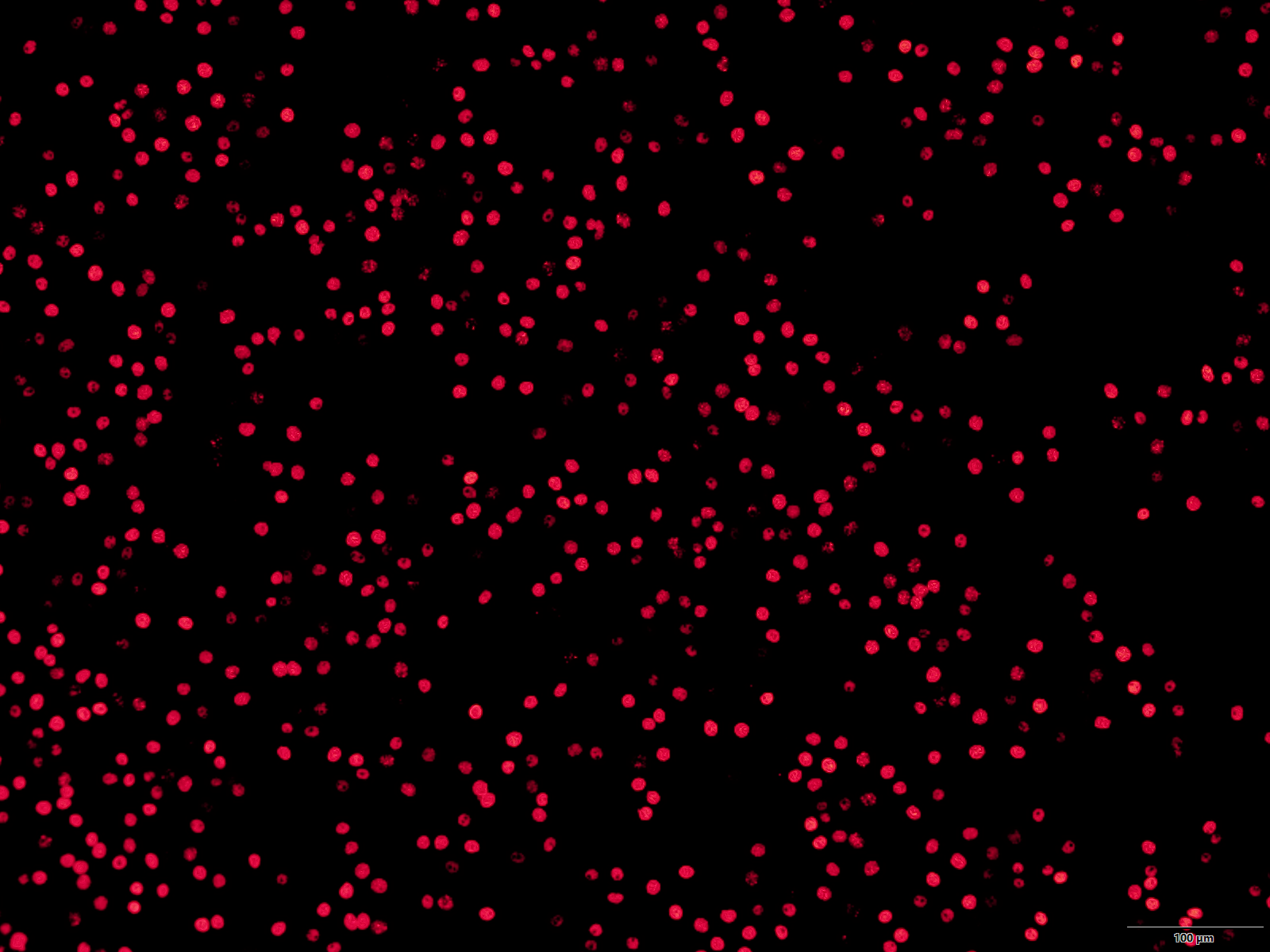

Supplement: S5 Data — This compressed folder contains the underlying numerical data and/or uncropped images used to generate the panels in Figs 6 and S1–S6, and S11. (ZIP) [file pbio.3003736.s019.zip › S5 Data/Supporting Information/Supporting Information fig1/B/EDU/18-edu-594-6.png]

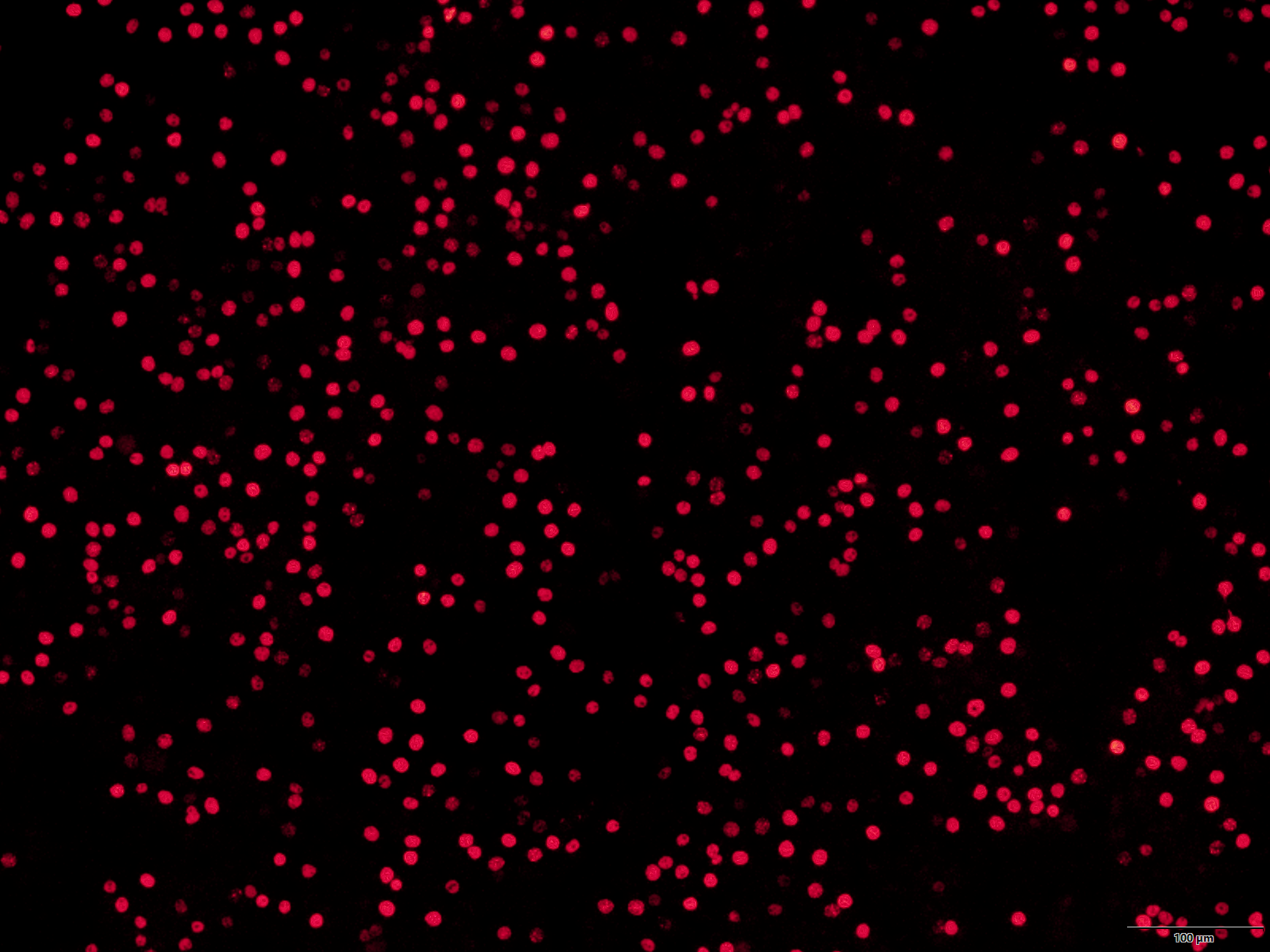

Supplement: S5 Data — This compressed folder contains the underlying numerical data and/or uncropped images used to generate the panels in Figs 6 and S1–S6, and S11. (ZIP) [file pbio.3003736.s019.zip › S5 Data/Supporting Information/Supporting Information fig1/B/EDU/18-edu-594-7.png]

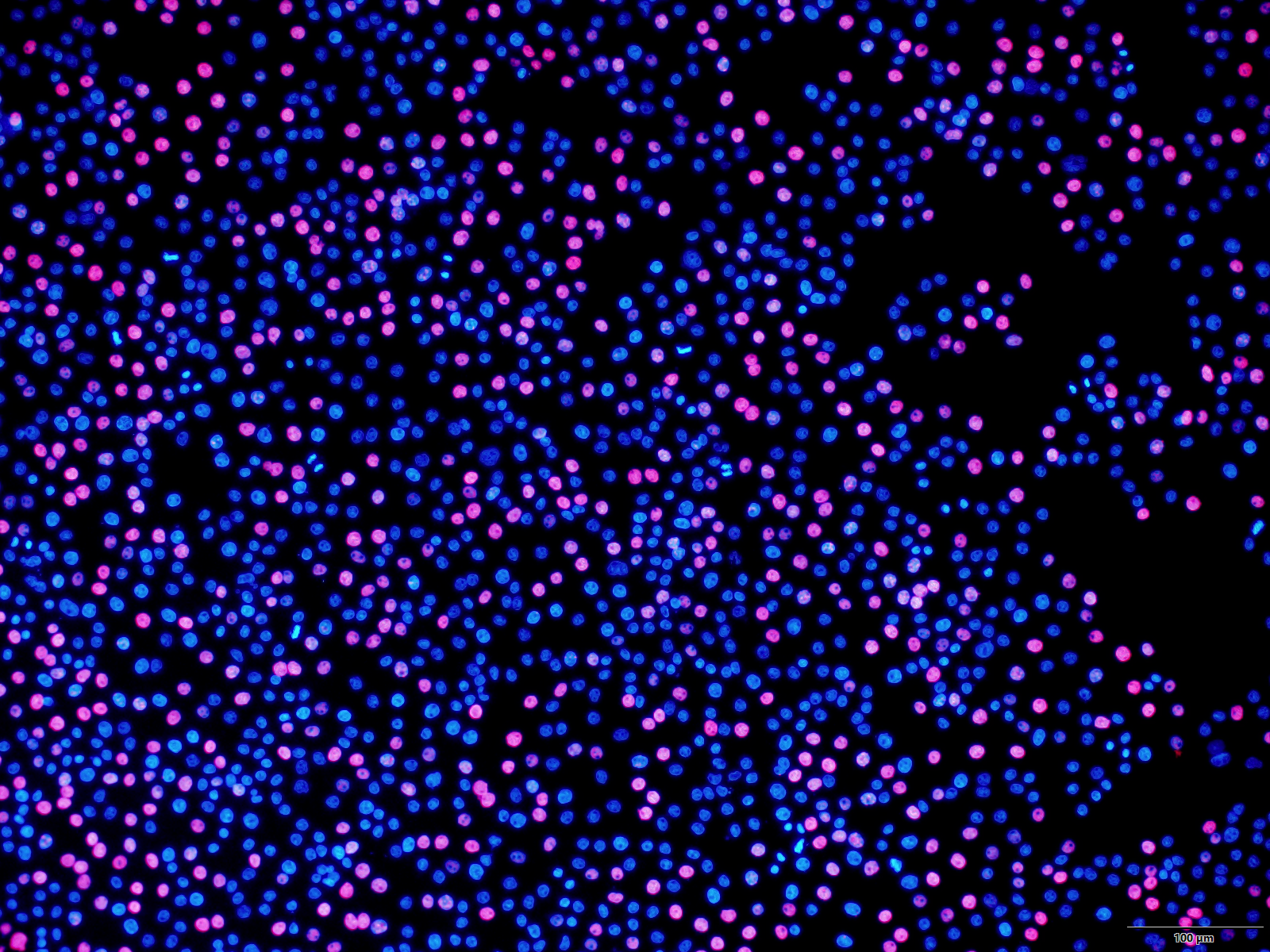

Supplement: S5 Data — This compressed folder contains the underlying numerical data and/or uncropped images used to generate the panels in Figs 6 and S1–S6, and S11. (ZIP) [file pbio.3003736.s019.zip › S5 Data/Supporting Information/Supporting Information fig1/B/EDU/18-edu-6.png]

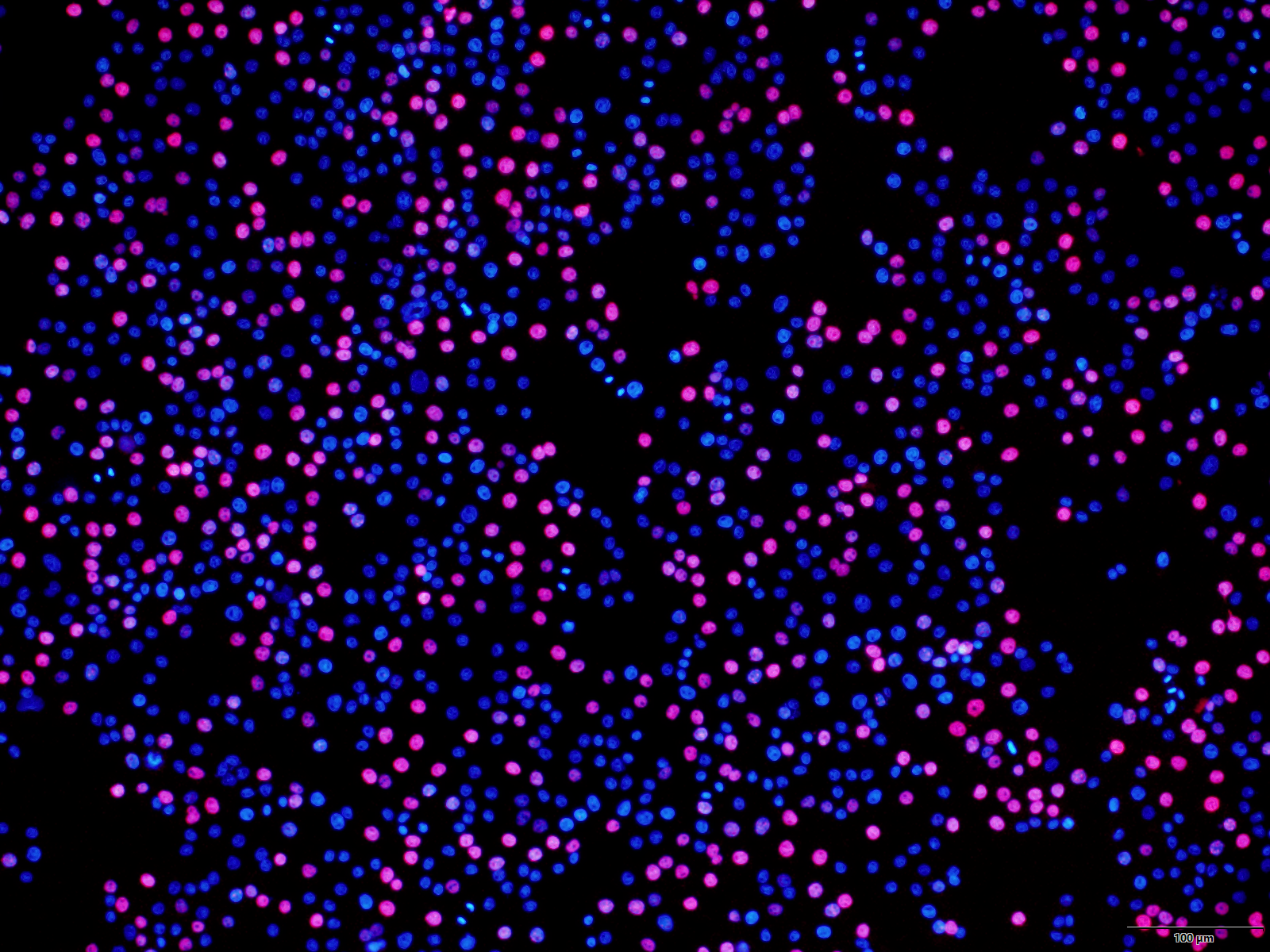

Supplement: S5 Data — This compressed folder contains the underlying numerical data and/or uncropped images used to generate the panels in Figs 6 and S1–S6, and S11. (ZIP) [file pbio.3003736.s019.zip › S5 Data/Supporting Information/Supporting Information fig1/B/EDU/18-edu-7.png]

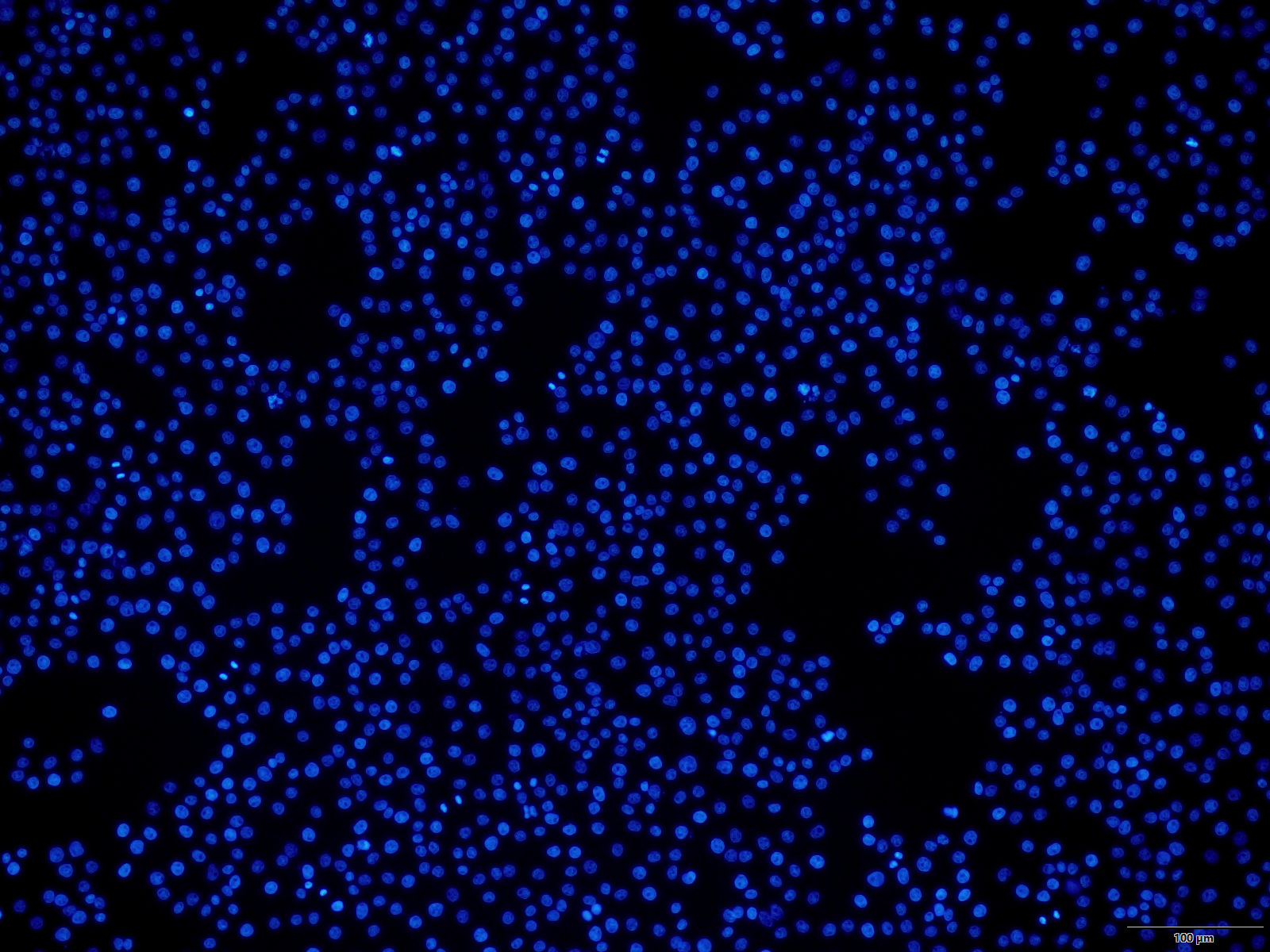

Supplement: S5 Data — This compressed folder contains the underlying numerical data and/or uncropped images used to generate the panels in Figs 6 and S1–S6, and S11. (ZIP) [file pbio.3003736.s019.zip › S5 Data/Supporting Information/Supporting Information fig1/B/EDU/18-edu-dapi-5.png]

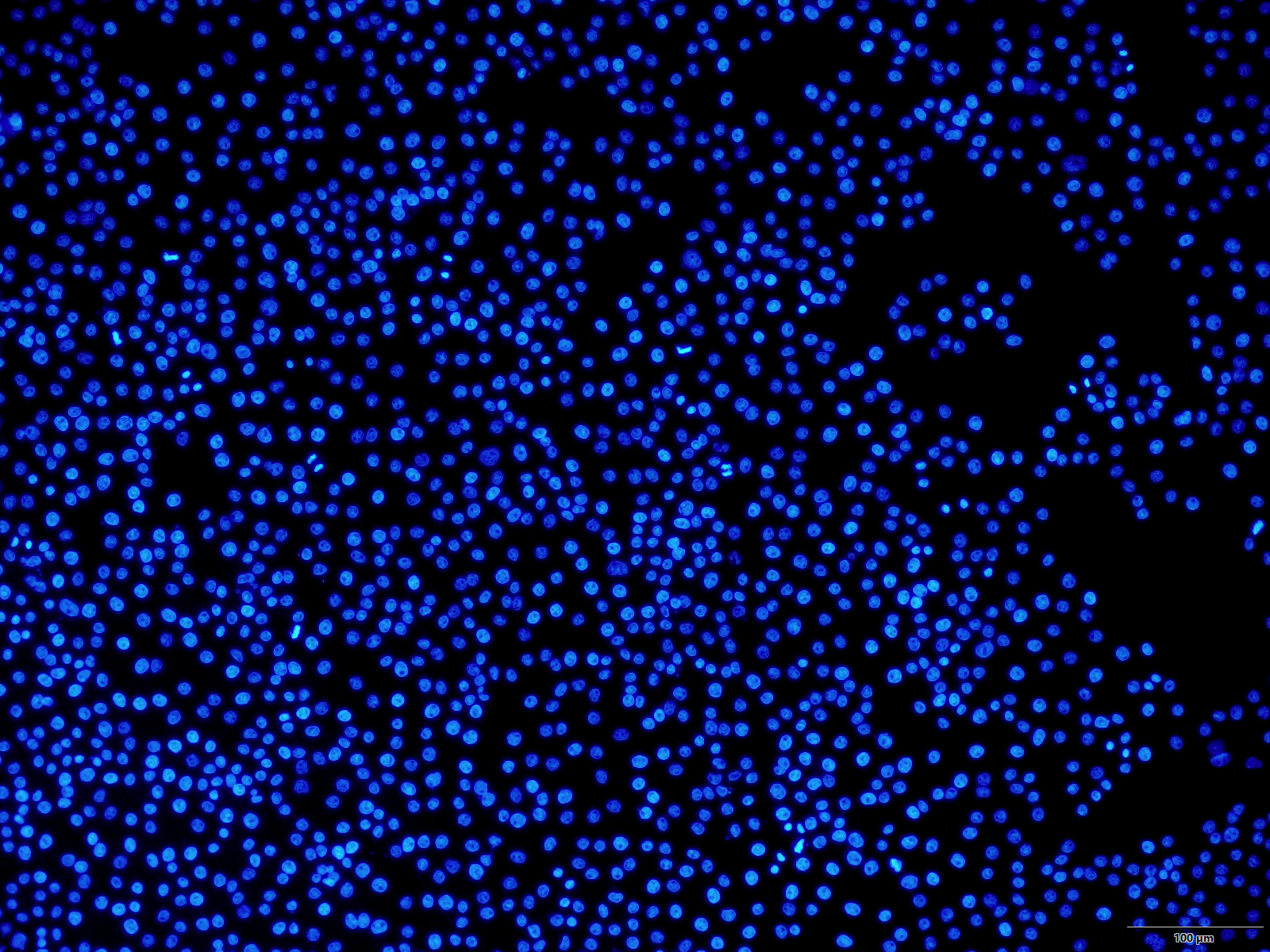

Supplement: S5 Data — This compressed folder contains the underlying numerical data and/or uncropped images used to generate the panels in Figs 6 and S1–S6, and S11. (ZIP) [file pbio.3003736.s019.zip › S5 Data/Supporting Information/Supporting Information fig1/B/EDU/18-edu-dapi-6.png]

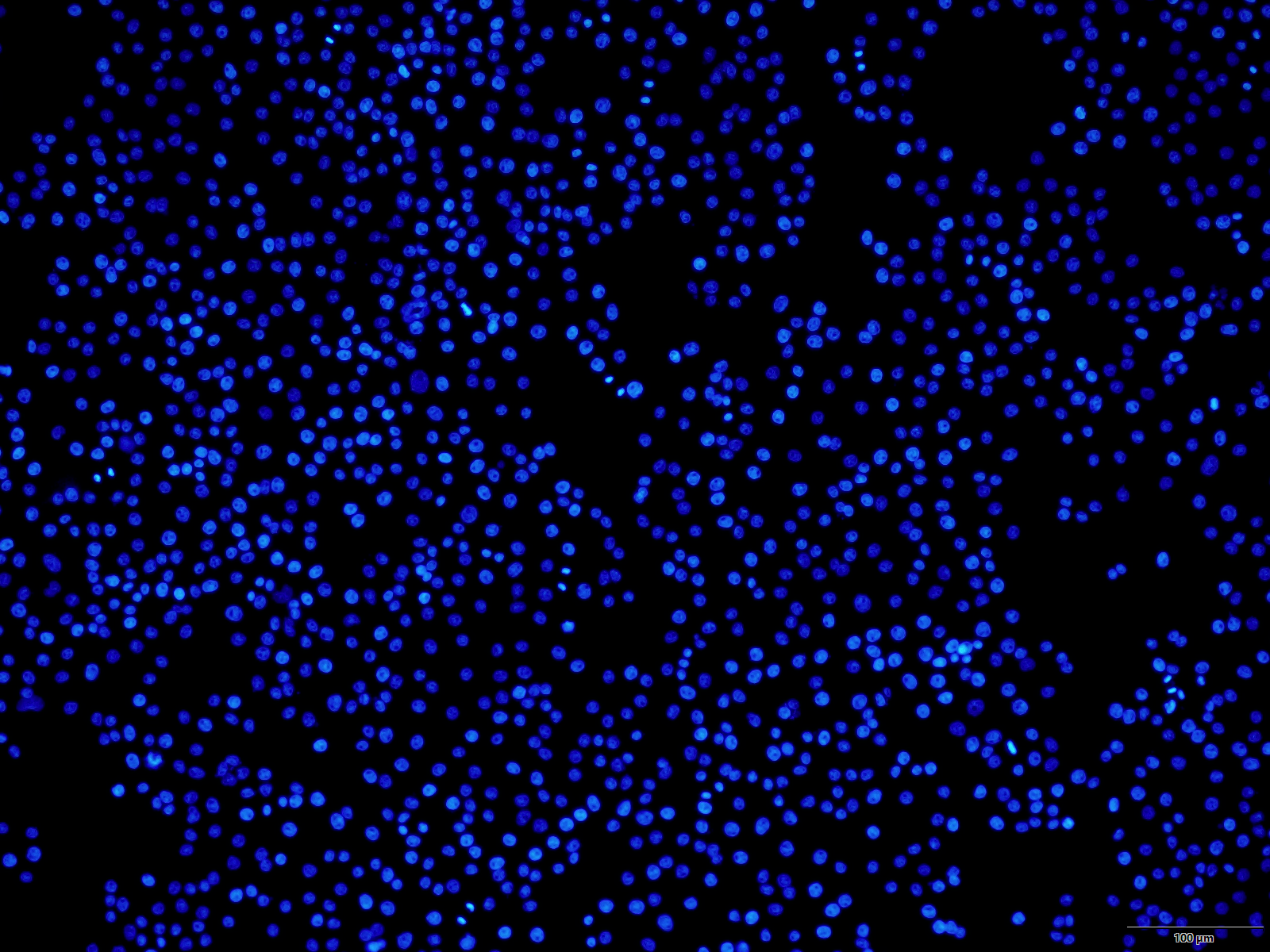

Supplement: S5 Data — This compressed folder contains the underlying numerical data and/or uncropped images used to generate the panels in Figs 6 and S1–S6, and S11. (ZIP) [file pbio.3003736.s019.zip › S5 Data/Supporting Information/Supporting Information fig1/B/EDU/18-edu-dapi-7.png]

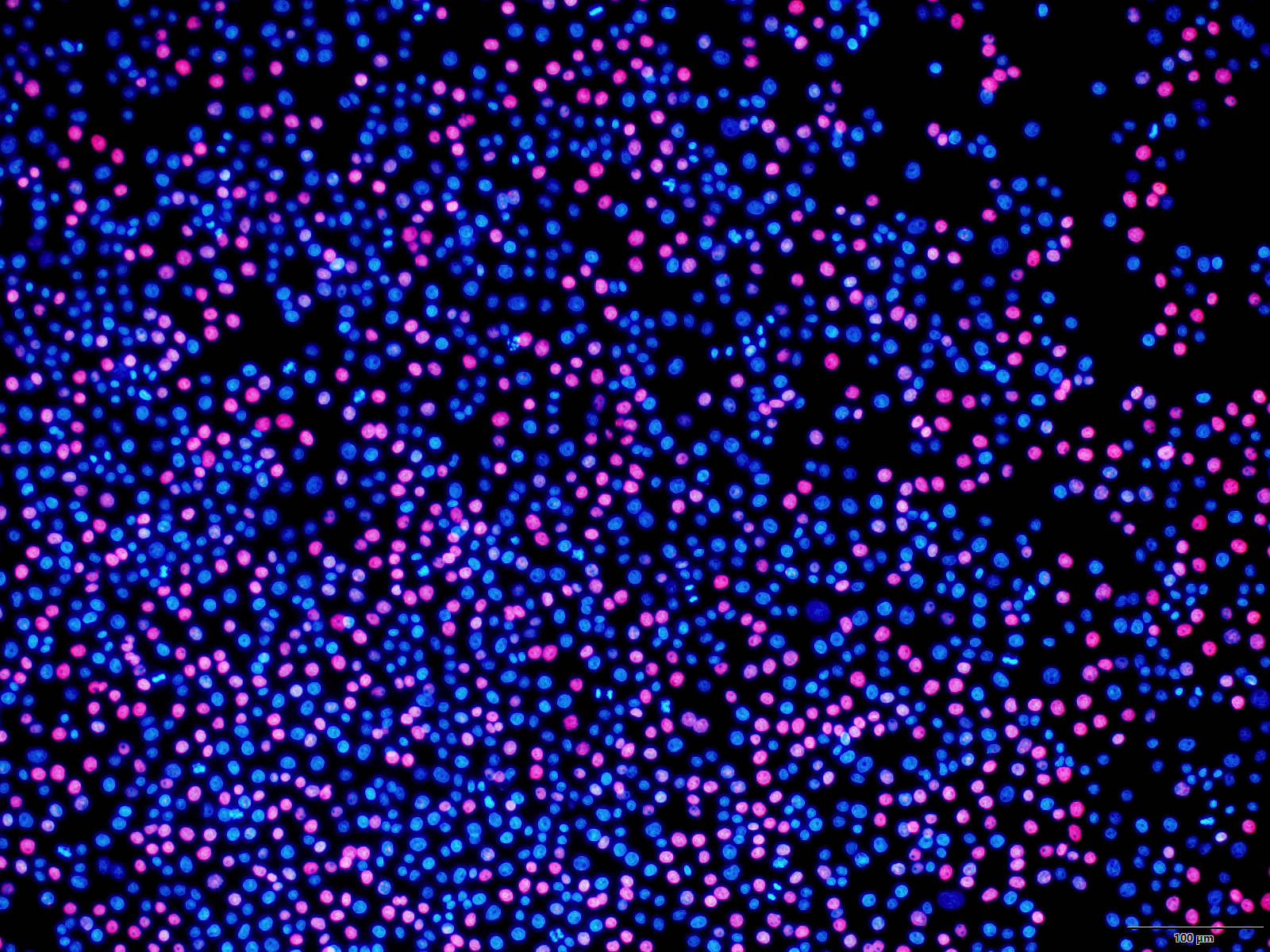

Supplement: S5 Data — This compressed folder contains the underlying numerical data and/or uncropped images used to generate the panels in Figs 6 and S1–S6, and S11. (ZIP) [file pbio.3003736.s019.zip › S5 Data/Supporting Information/Supporting Information fig1/B/EDU/pk-edu-10.png]

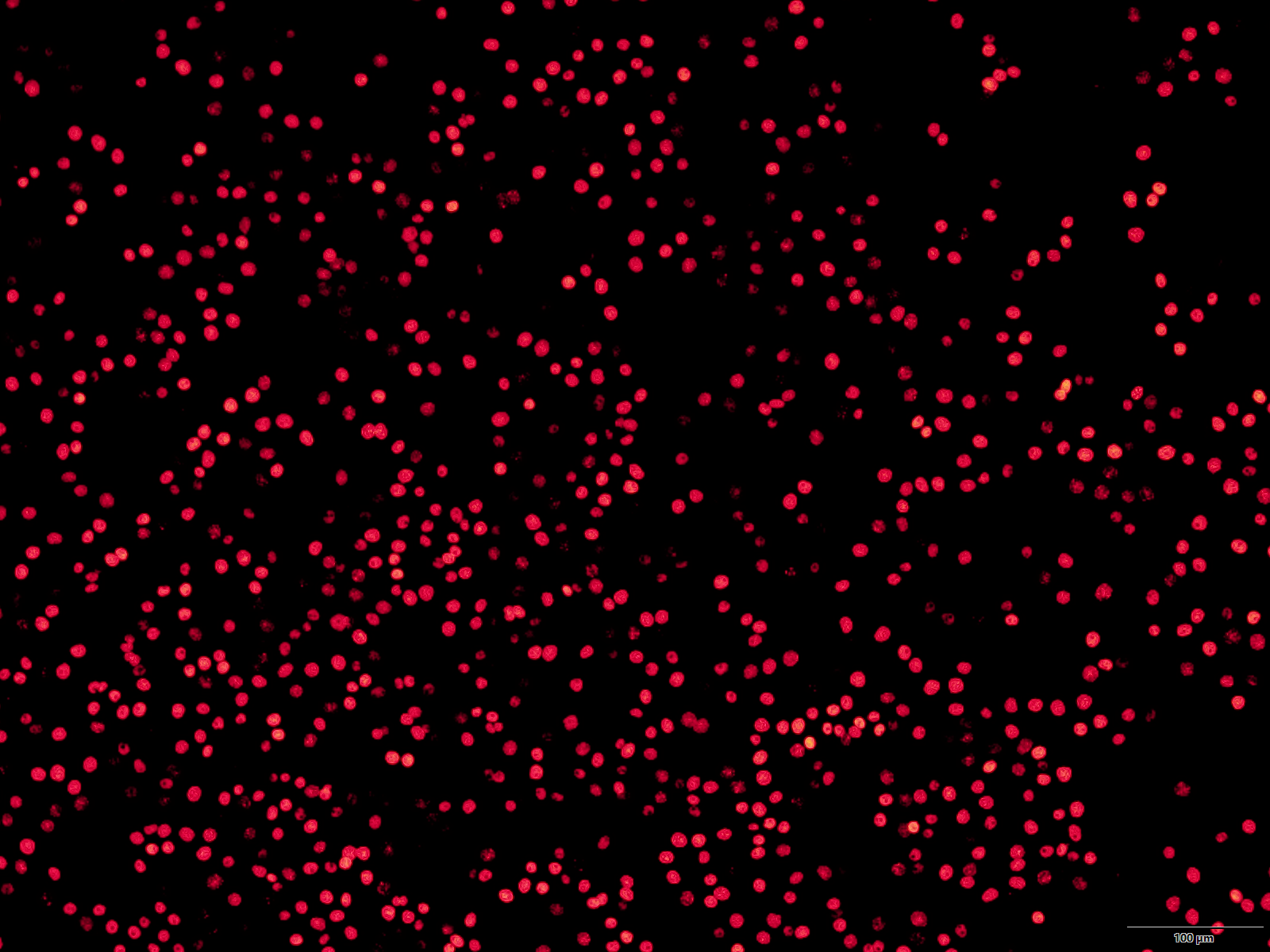

Supplement: S5 Data — This compressed folder contains the underlying numerical data and/or uncropped images used to generate the panels in Figs 6 and S1–S6, and S11. (ZIP) [file pbio.3003736.s019.zip › S5 Data/Supporting Information/Supporting Information fig1/B/EDU/pk-edu-594-10.png]

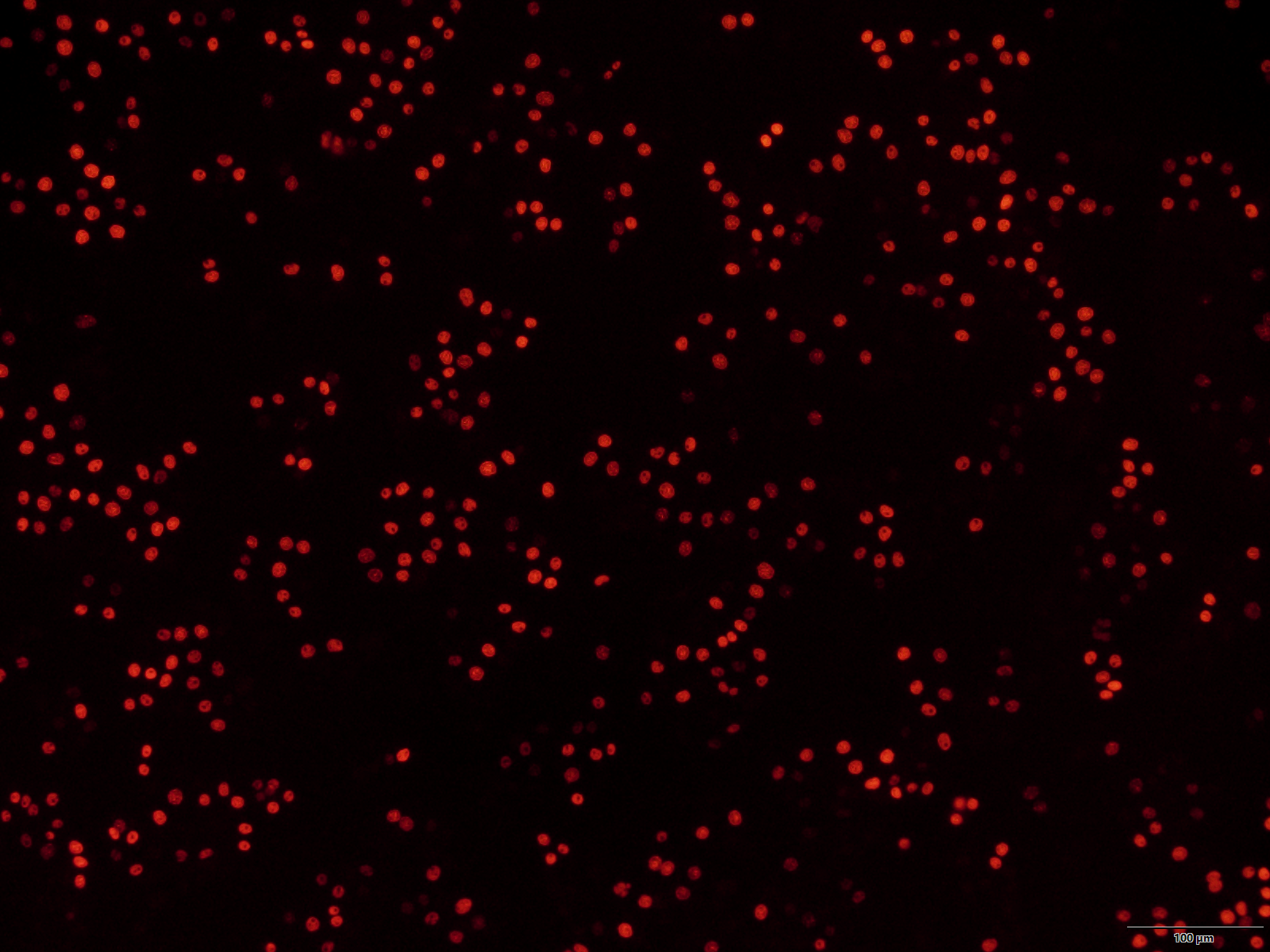

Supplement: S5 Data — This compressed folder contains the underlying numerical data and/or uncropped images used to generate the panels in Figs 6 and S1–S6, and S11. (ZIP) [file pbio.3003736.s019.zip › S5 Data/Supporting Information/Supporting Information fig1/B/EDU/pk-edu-594-8.png]

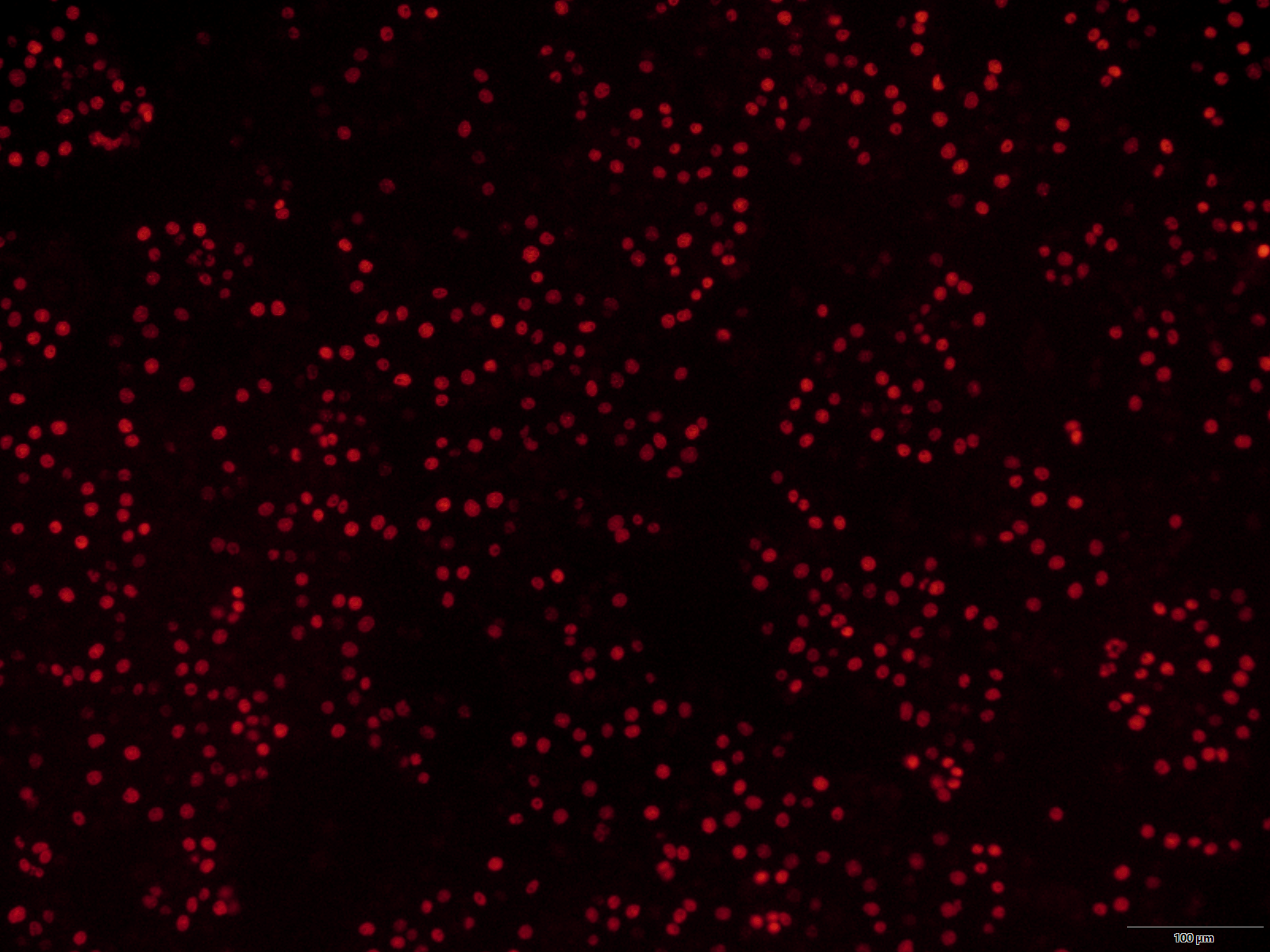

Supplement: S5 Data — This compressed folder contains the underlying numerical data and/or uncropped images used to generate the panels in Figs 6 and S1–S6, and S11. (ZIP) [file pbio.3003736.s019.zip › S5 Data/Supporting Information/Supporting Information fig1/B/EDU/pk-edu-594-9.png]

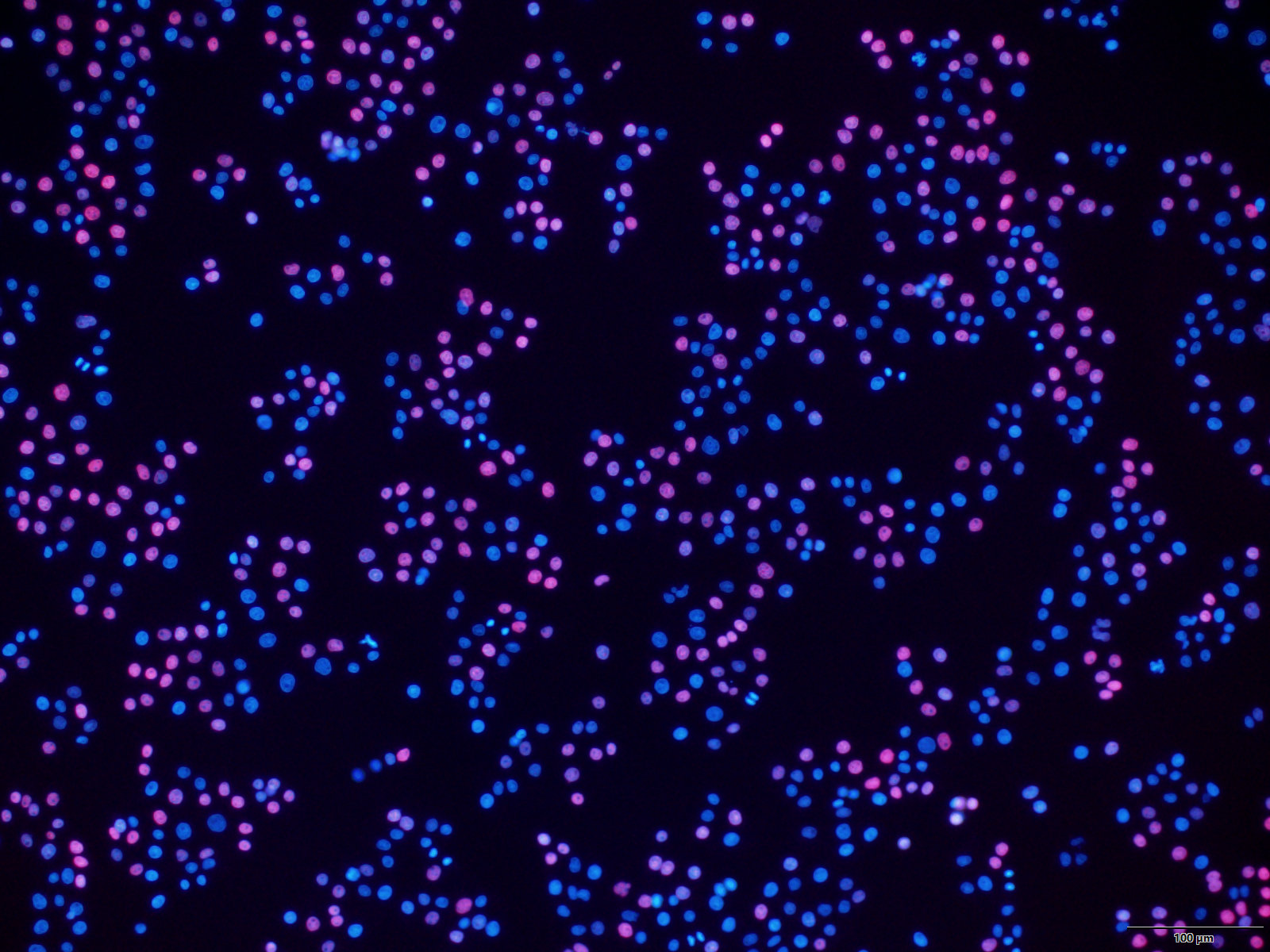

Supplement: S5 Data — This compressed folder contains the underlying numerical data and/or uncropped images used to generate the panels in Figs 6 and S1–S6, and S11. (ZIP) [file pbio.3003736.s019.zip › S5 Data/Supporting Information/Supporting Information fig1/B/EDU/pk-edu-8.png]

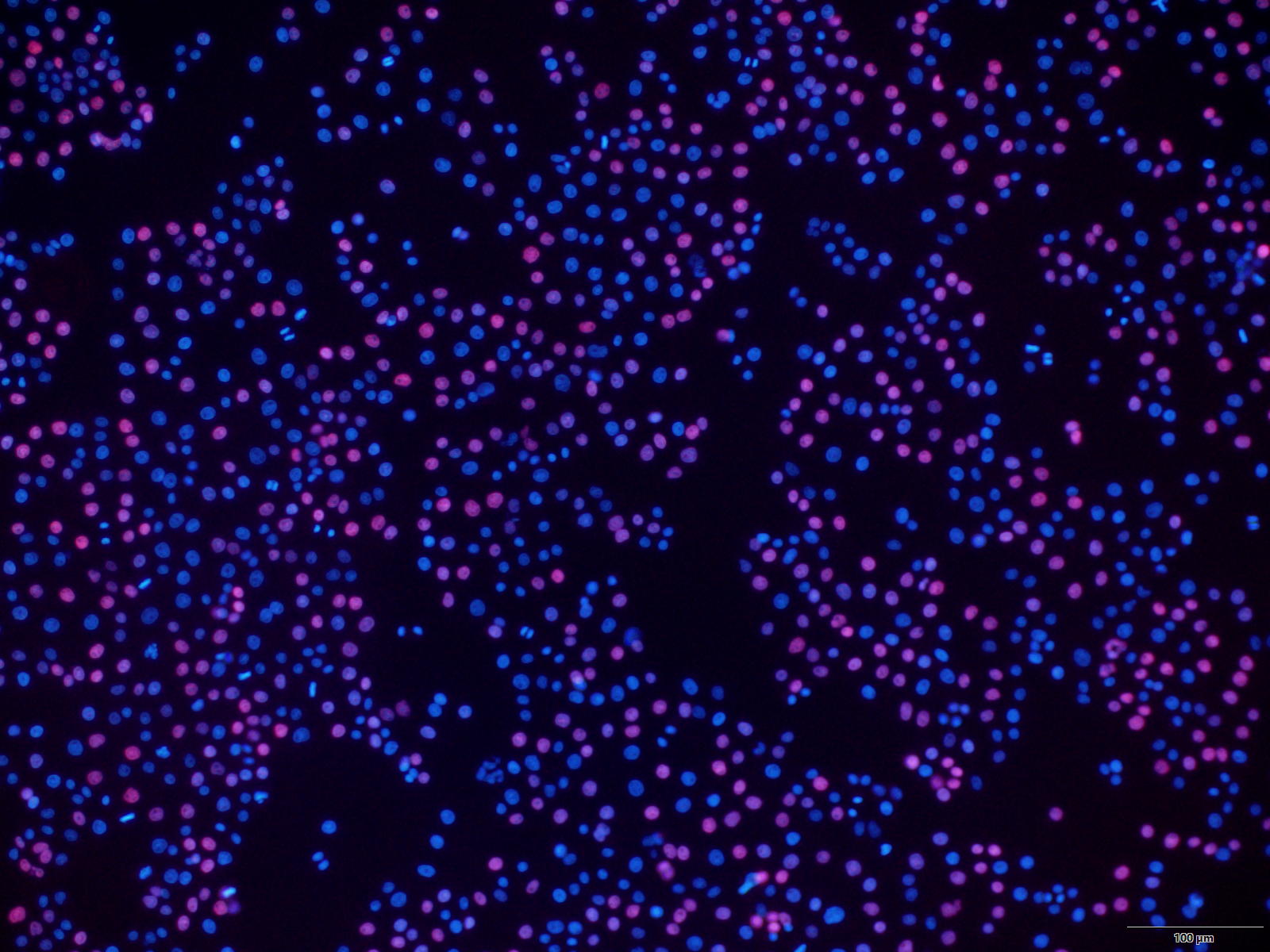

Supplement: S5 Data — This compressed folder contains the underlying numerical data and/or uncropped images used to generate the panels in Figs 6 and S1–S6, and S11. (ZIP) [file pbio.3003736.s019.zip › S5 Data/Supporting Information/Supporting Information fig1/B/EDU/pk-edu-9.png]

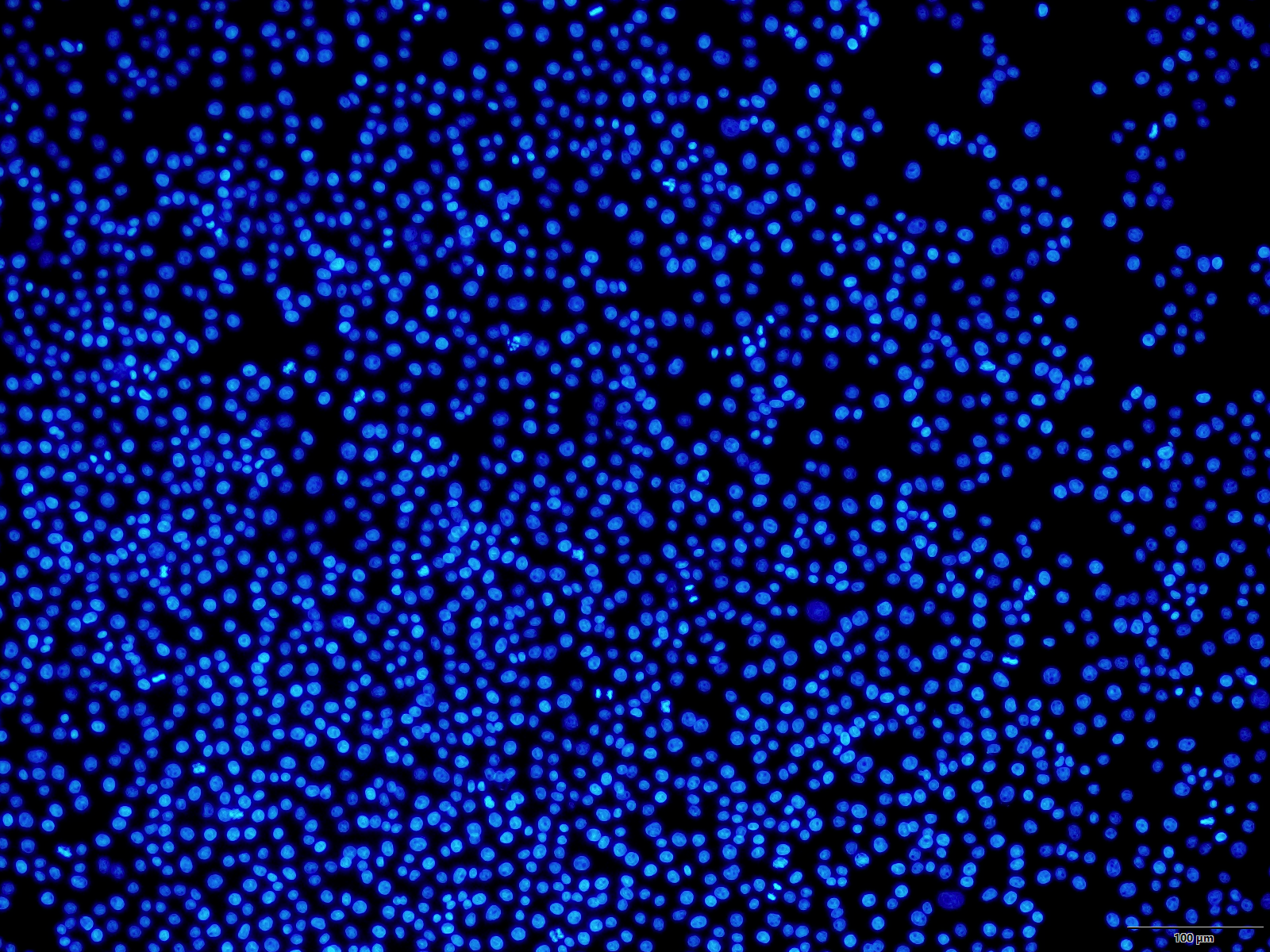

Supplement: S5 Data — This compressed folder contains the underlying numerical data and/or uncropped images used to generate the panels in Figs 6 and S1–S6, and S11. (ZIP) [file pbio.3003736.s019.zip › S5 Data/Supporting Information/Supporting Information fig1/B/EDU/pk-edu-dapi-10.png]

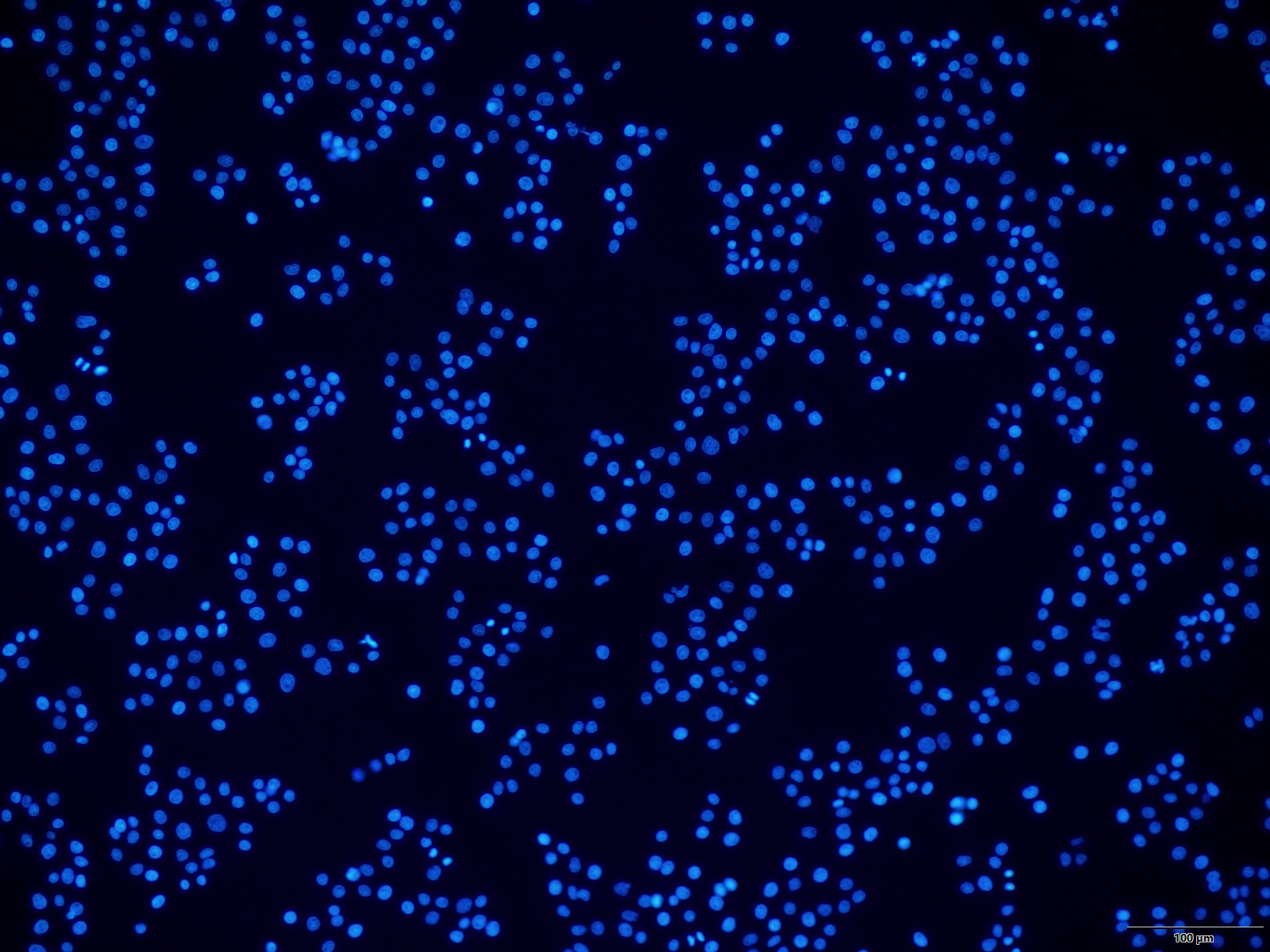

Supplement: S5 Data — This compressed folder contains the underlying numerical data and/or uncropped images used to generate the panels in Figs 6 and S1–S6, and S11. (ZIP) [file pbio.3003736.s019.zip › S5 Data/Supporting Information/Supporting Information fig1/B/EDU/pk-edu-dapi-8.png]

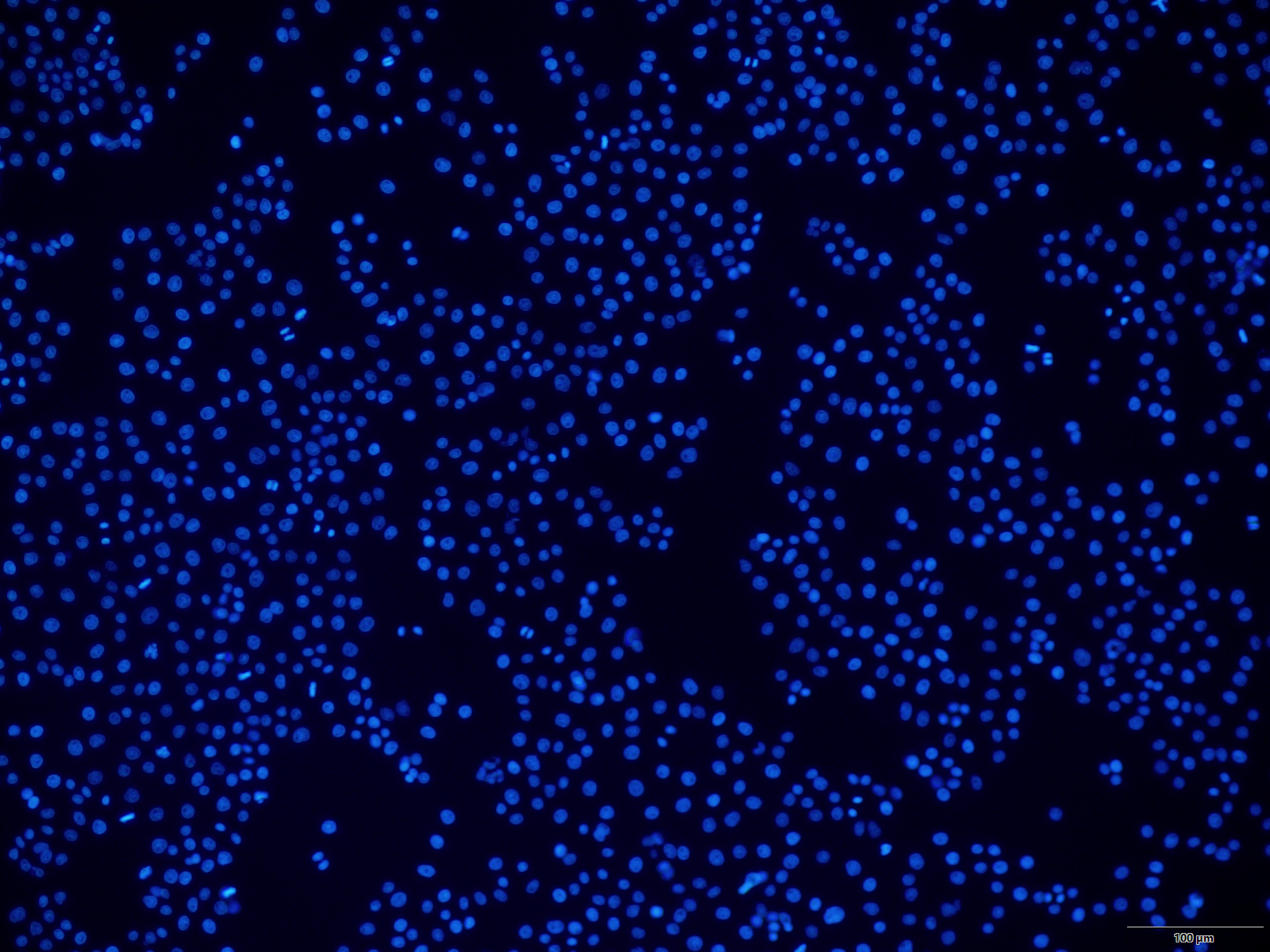

Supplement: S5 Data — This compressed folder contains the underlying numerical data and/or uncropped images used to generate the panels in Figs 6 and S1–S6, and S11. (ZIP) [file pbio.3003736.s019.zip › S5 Data/Supporting Information/Supporting Information fig1/B/EDU/pk-edu-dapi-9.png]

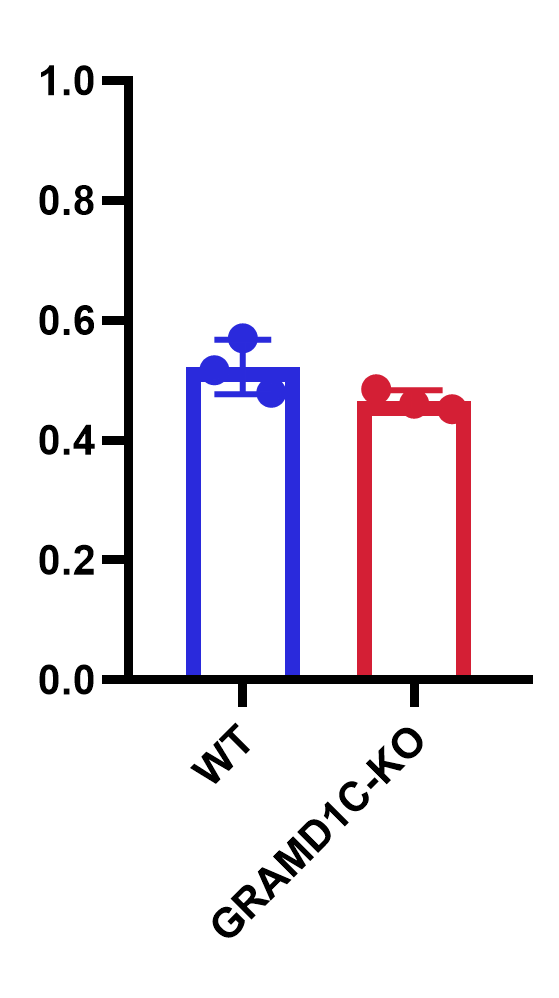

Supplement: S5 Data — This compressed folder contains the underlying numerical data and/or uncropped images used to generate the panels in Figs 6 and S1–S6, and S11. (ZIP) [file pbio.3003736.s019.zip › S5 Data/Supporting Information/Supporting Information fig1/B/EDU.tif]

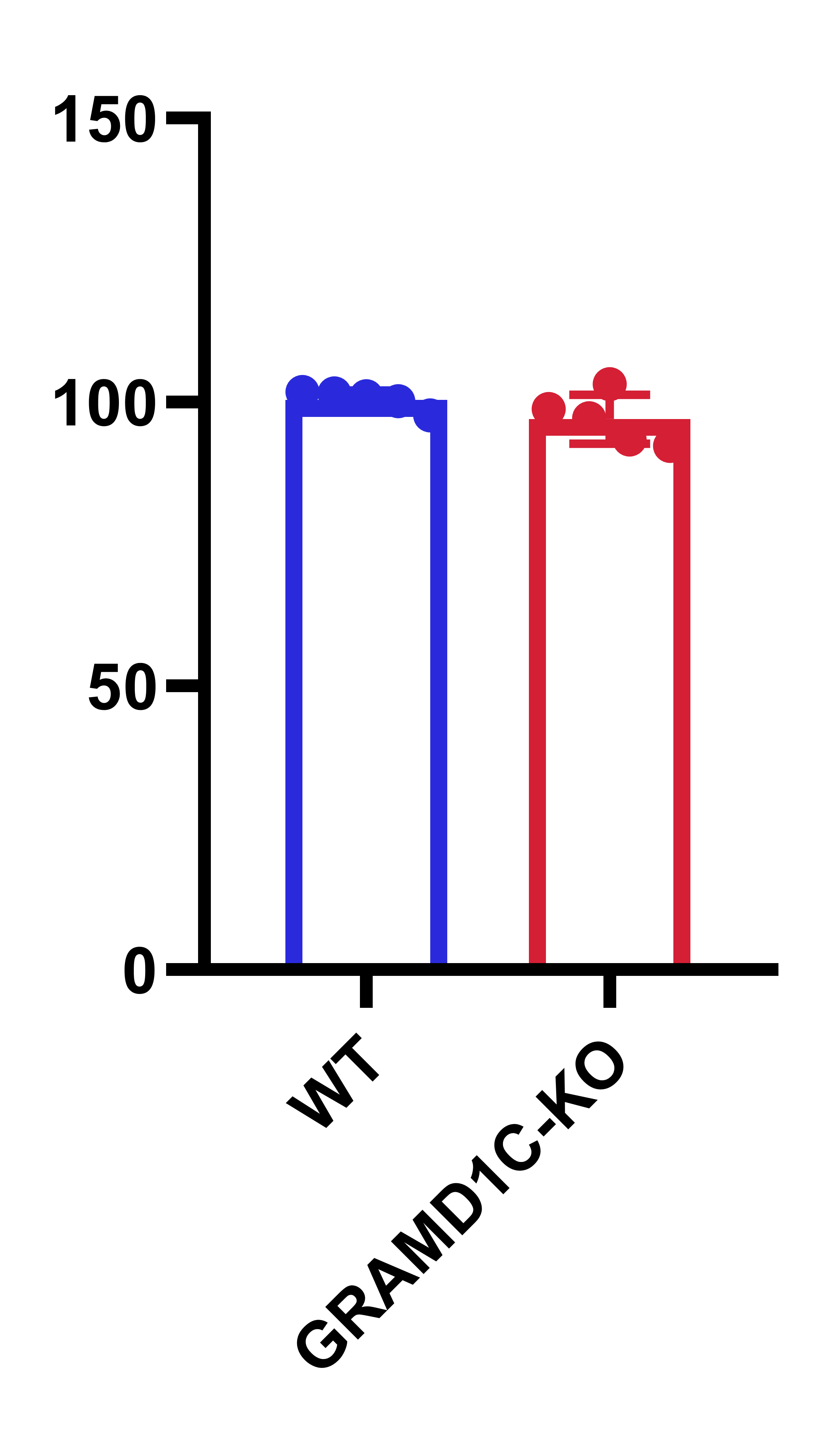

Supplement: S5 Data — This compressed folder contains the underlying numerical data and/or uncropped images used to generate the panels in Figs 6 and S1–S6, and S11. (ZIP) [file pbio.3003736.s019.zip › S5 Data/Supporting Information/Supporting Information fig1/D.VERO-MTS/Copy of VERO-KO.tif]

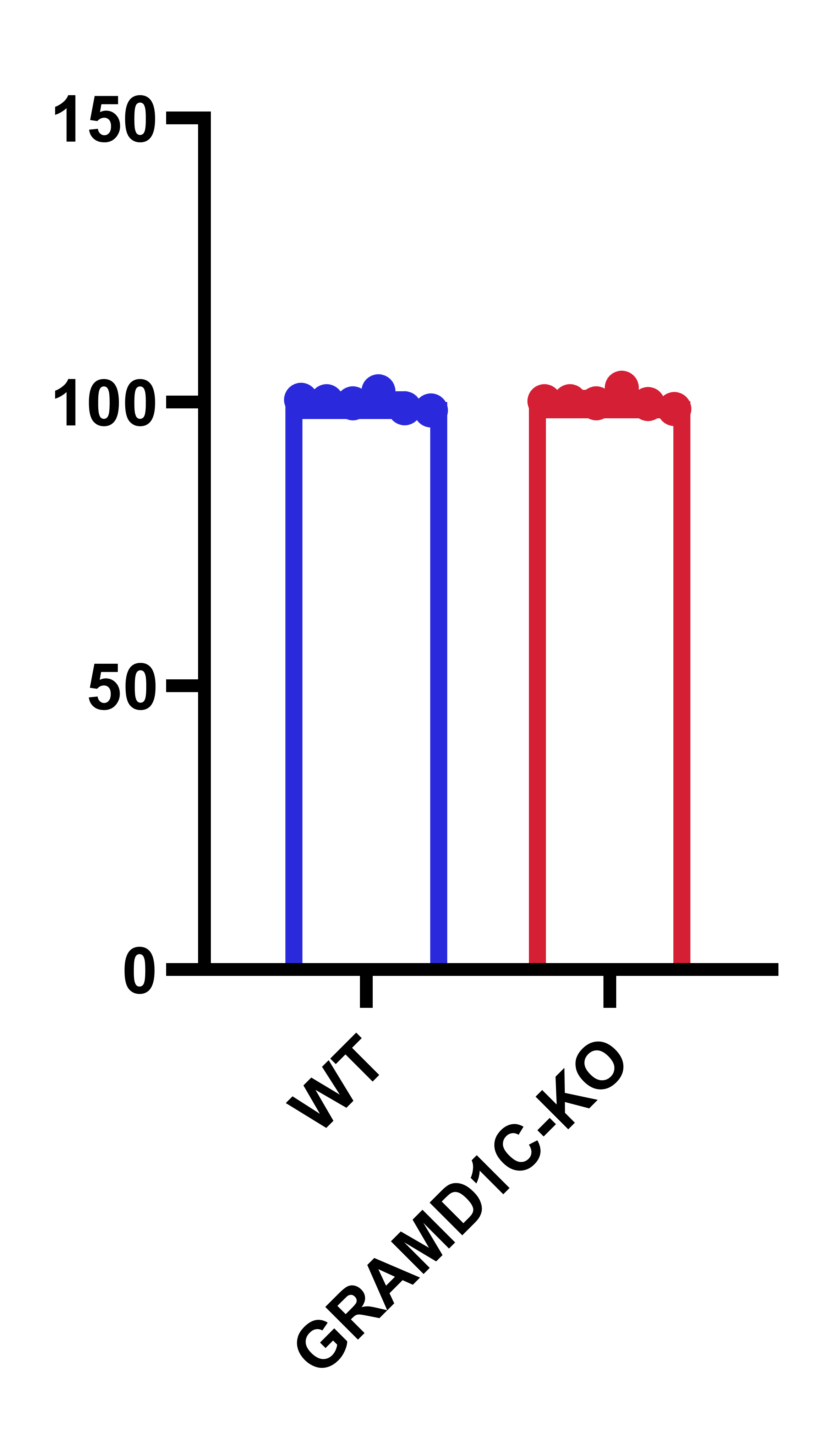

Supplement: S5 Data — This compressed folder contains the underlying numerical data and/or uncropped images used to generate the panels in Figs 6 and S1–S6, and S11. (ZIP) [file pbio.3003736.s019.zip › S5 Data/Supporting Information/Supporting Information fig1/F.Caco2-MTS/Copy of caco2-KO.tif]

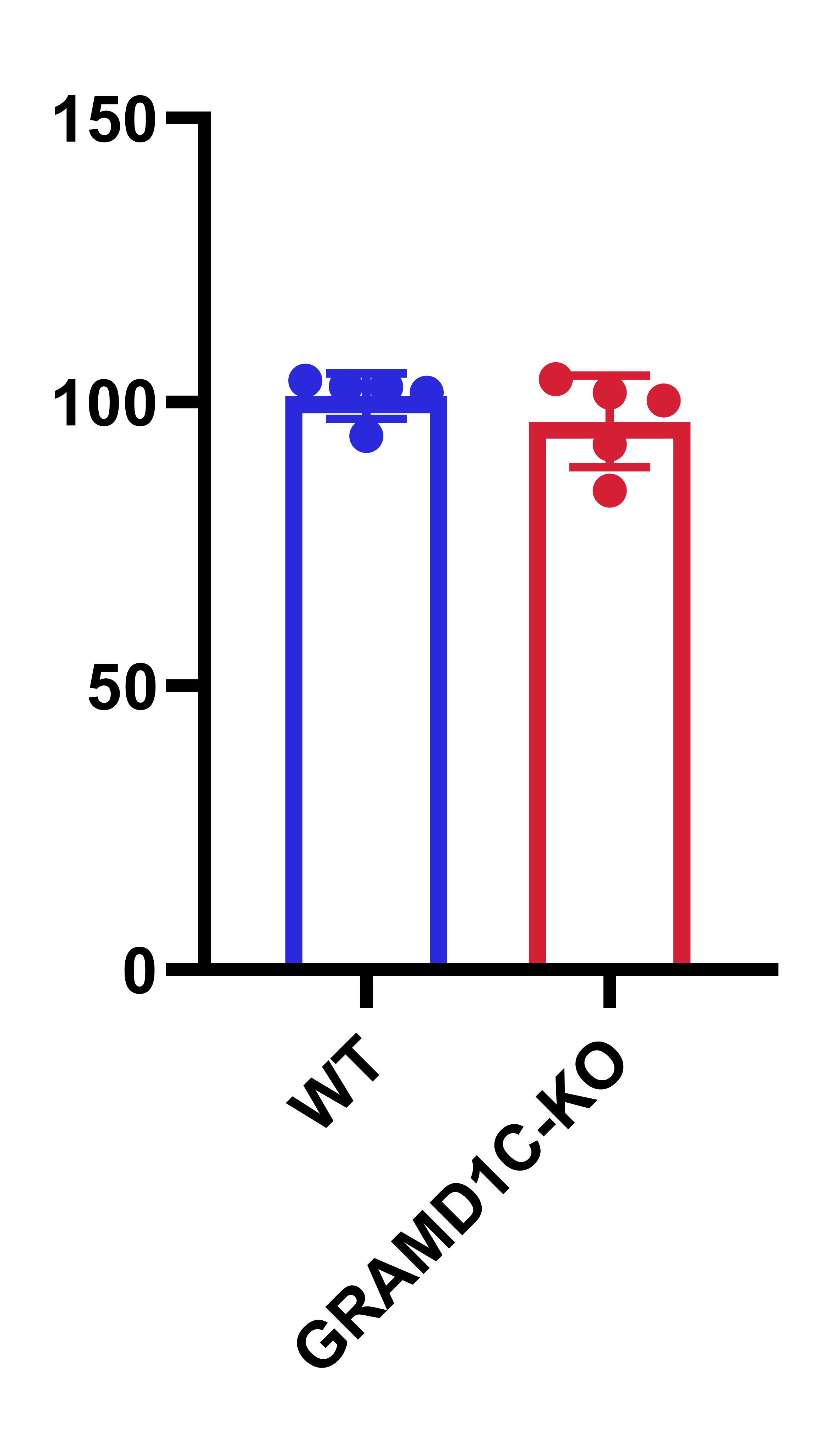

Supplement: S5 Data — This compressed folder contains the underlying numerical data and/or uncropped images used to generate the panels in Figs 6 and S1–S6, and S11. (ZIP) [file pbio.3003736.s019.zip › S5 Data/Supporting Information/Supporting Information fig1/H.L929-MTS/Copy of L929-KO.tif]

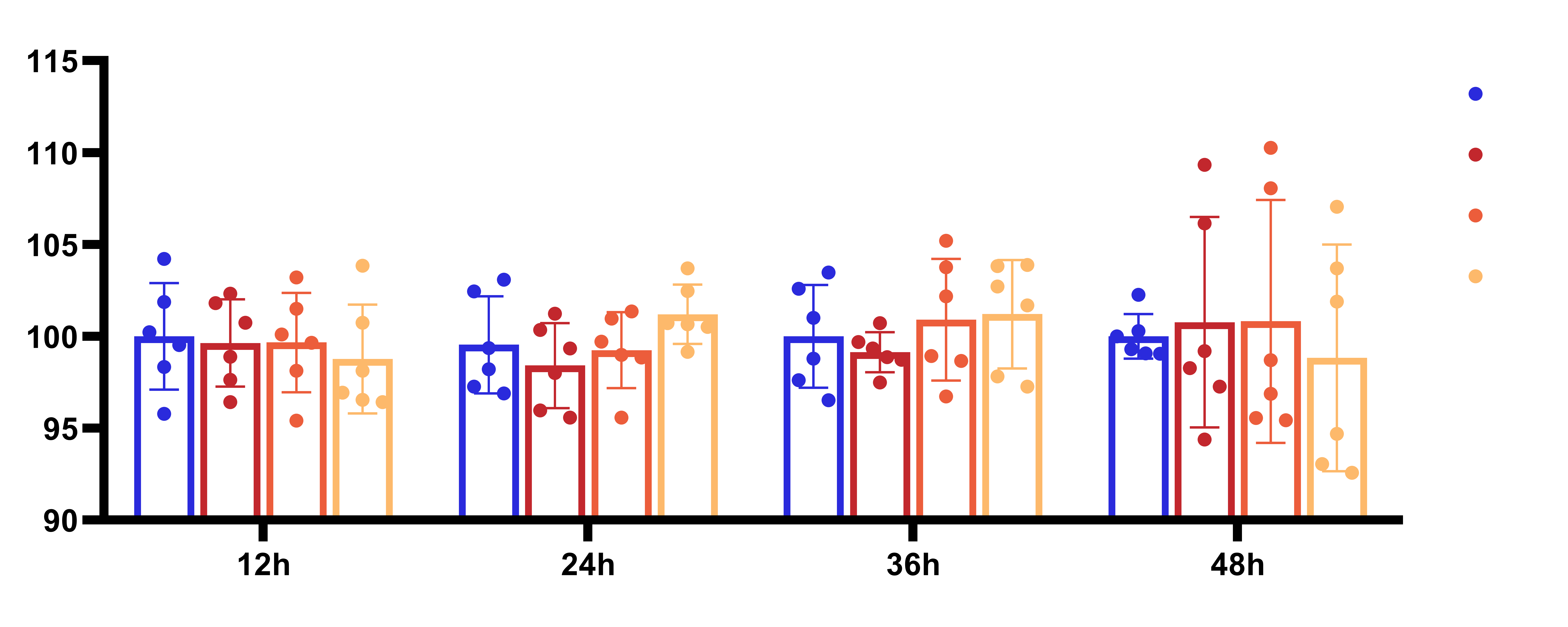

Supplement: S5 Data — This compressed folder contains the underlying numerical data and/or uncropped images used to generate the panels in Figs 6 and S1–S6, and S11. (ZIP) [file pbio.3003736.s019.zip › S5 Data/Supporting Information/Supporting Information fig11/A/12-48H-10-30um.tif]

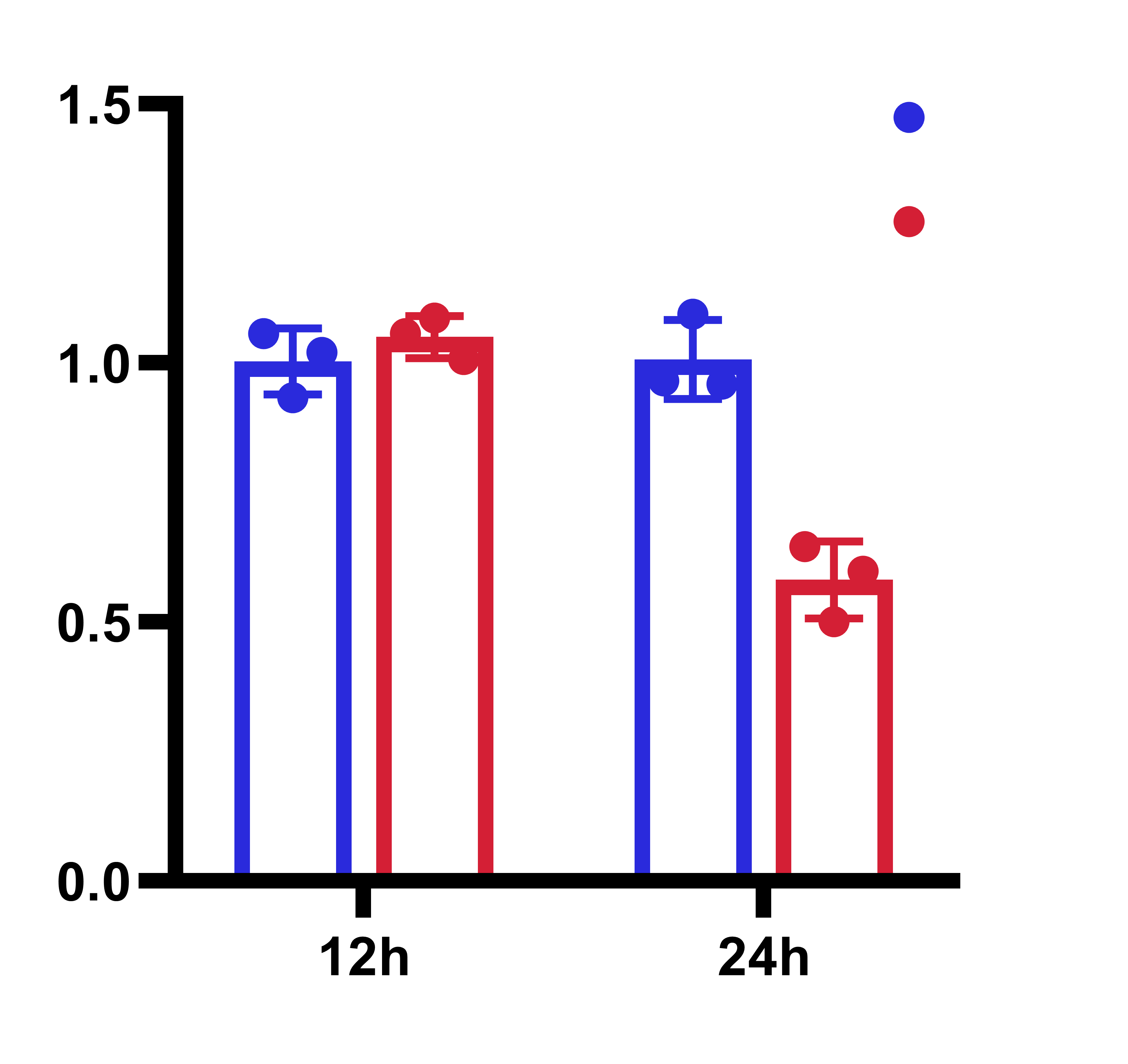

Supplement: S5 Data — This compressed folder contains the underlying numerical data and/or uncropped images used to generate the panels in Figs 6 and S1–S6, and S11. (ZIP) [file pbio.3003736.s019.zip › S5 Data/Supporting Information/Supporting Information fig11/B/APN.tif]

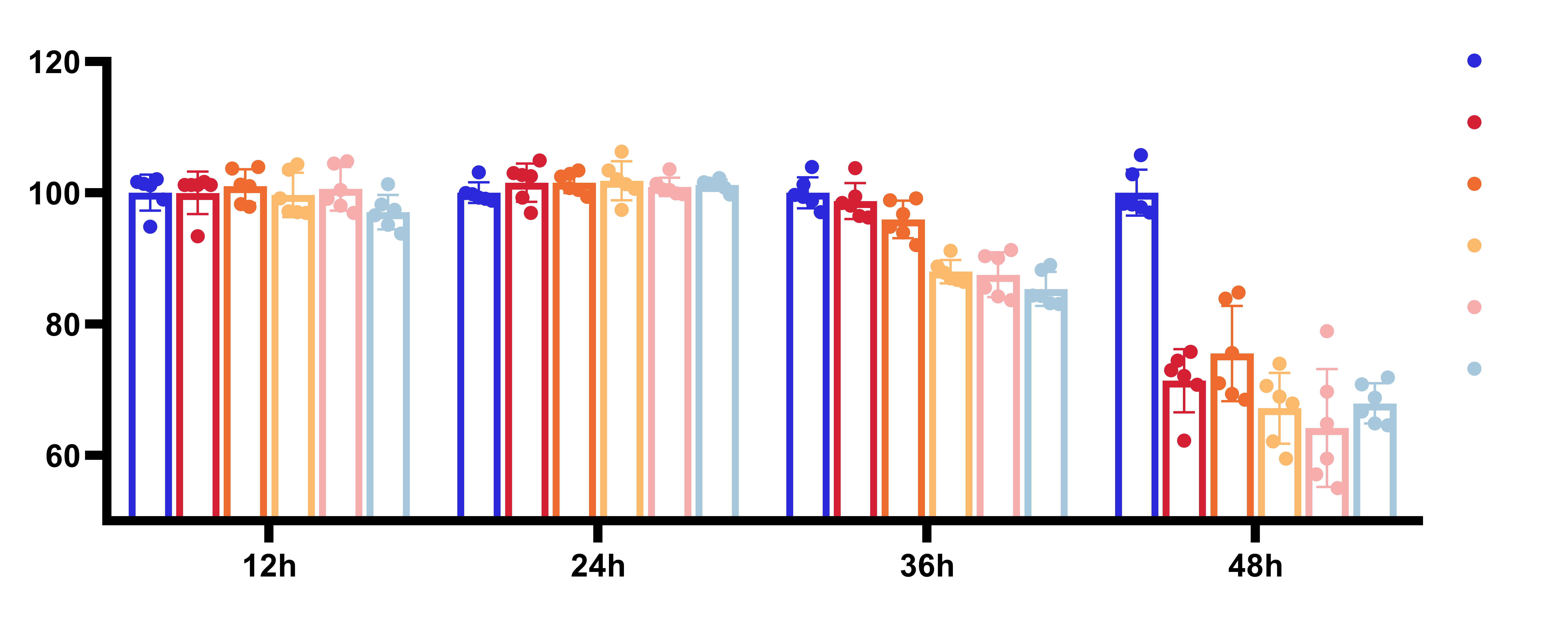

Supplement: S5 Data — This compressed folder contains the underlying numerical data and/or uncropped images used to generate the panels in Figs 6 and S1–S6, and S11. (ZIP) [file pbio.3003736.s019.zip › S5 Data/Supporting Information/Supporting Information fig11/C/293T-20hc.tif]

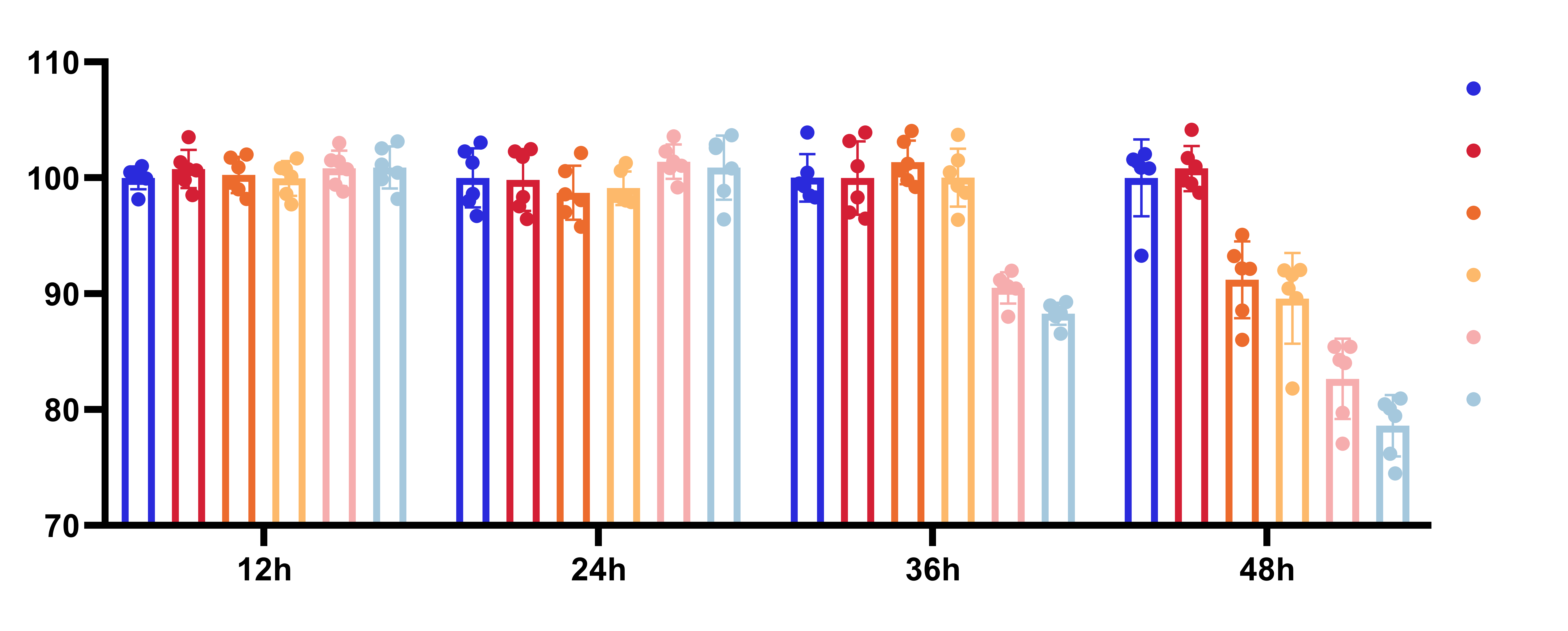

Supplement: S5 Data — This compressed folder contains the underlying numerical data and/or uncropped images used to generate the panels in Figs 6 and S1–S6, and S11. (ZIP) [file pbio.3003736.s019.zip › S5 Data/Supporting Information/Supporting Information fig11/D/CACO2-10UM-48H.tif]

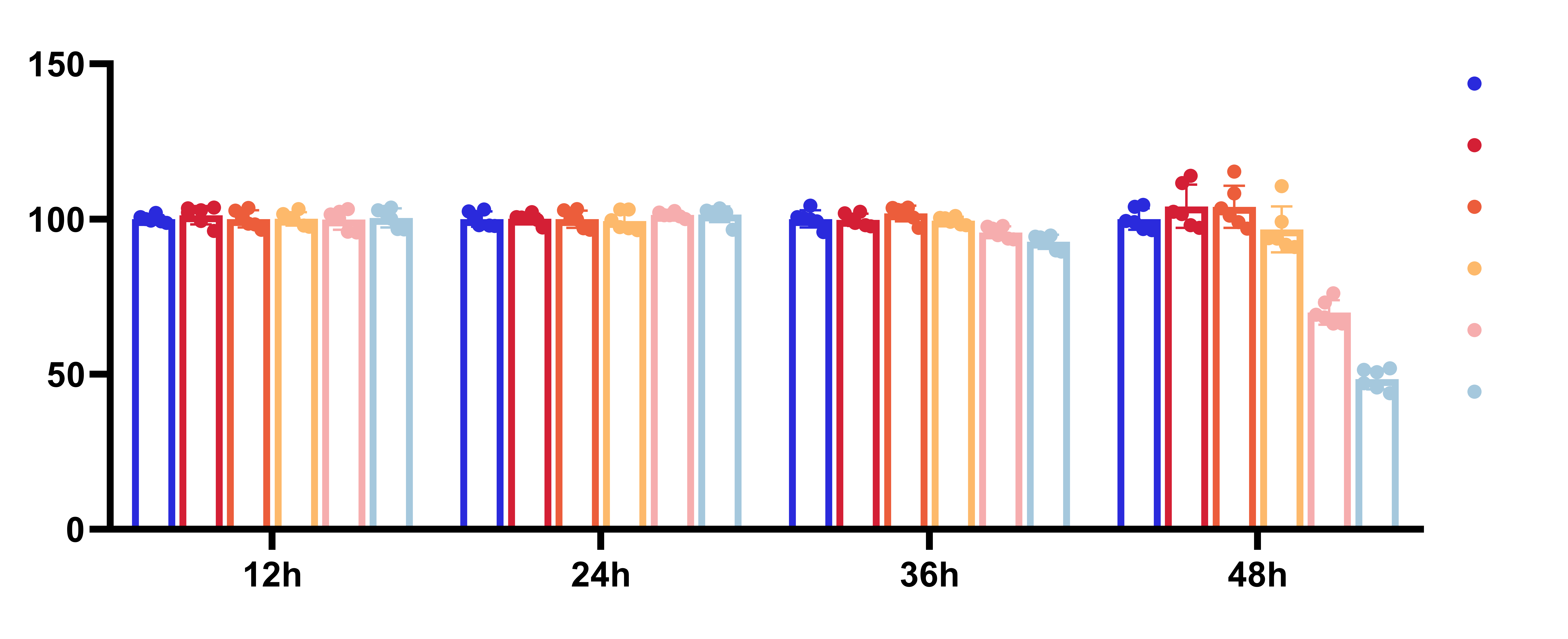

Supplement: S5 Data — This compressed folder contains the underlying numerical data and/or uncropped images used to generate the panels in Figs 6 and S1–S6, and S11. (ZIP) [file pbio.3003736.s019.zip › S5 Data/Supporting Information/Supporting Information fig11/E/vero-12-48-10-50um.tif]

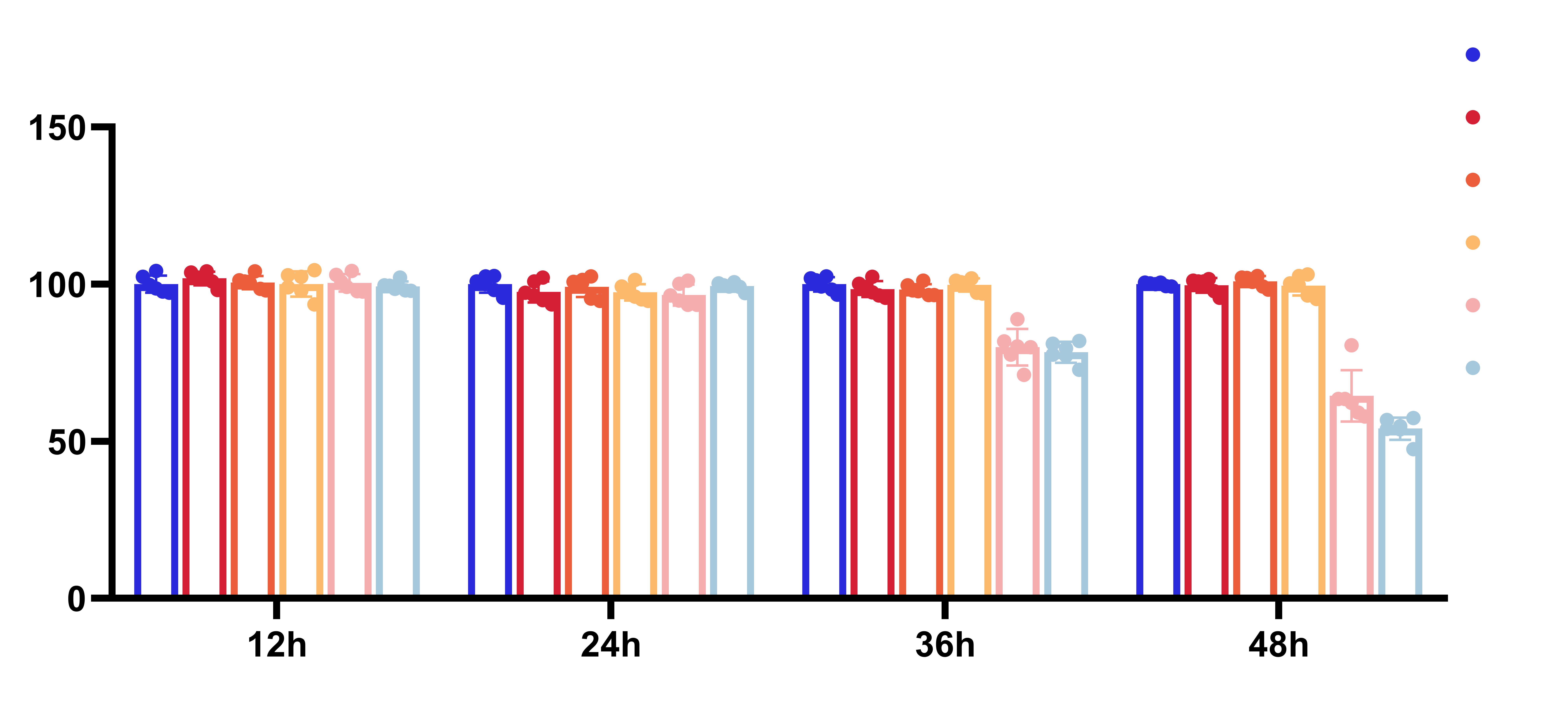

Supplement: S5 Data — This compressed folder contains the underlying numerical data and/or uncropped images used to generate the panels in Figs 6 and S1–S6, and S11. (ZIP) [file pbio.3003736.s019.zip › S5 Data/Supporting Information/Supporting Information fig11/F/L929-12-48H.tif]

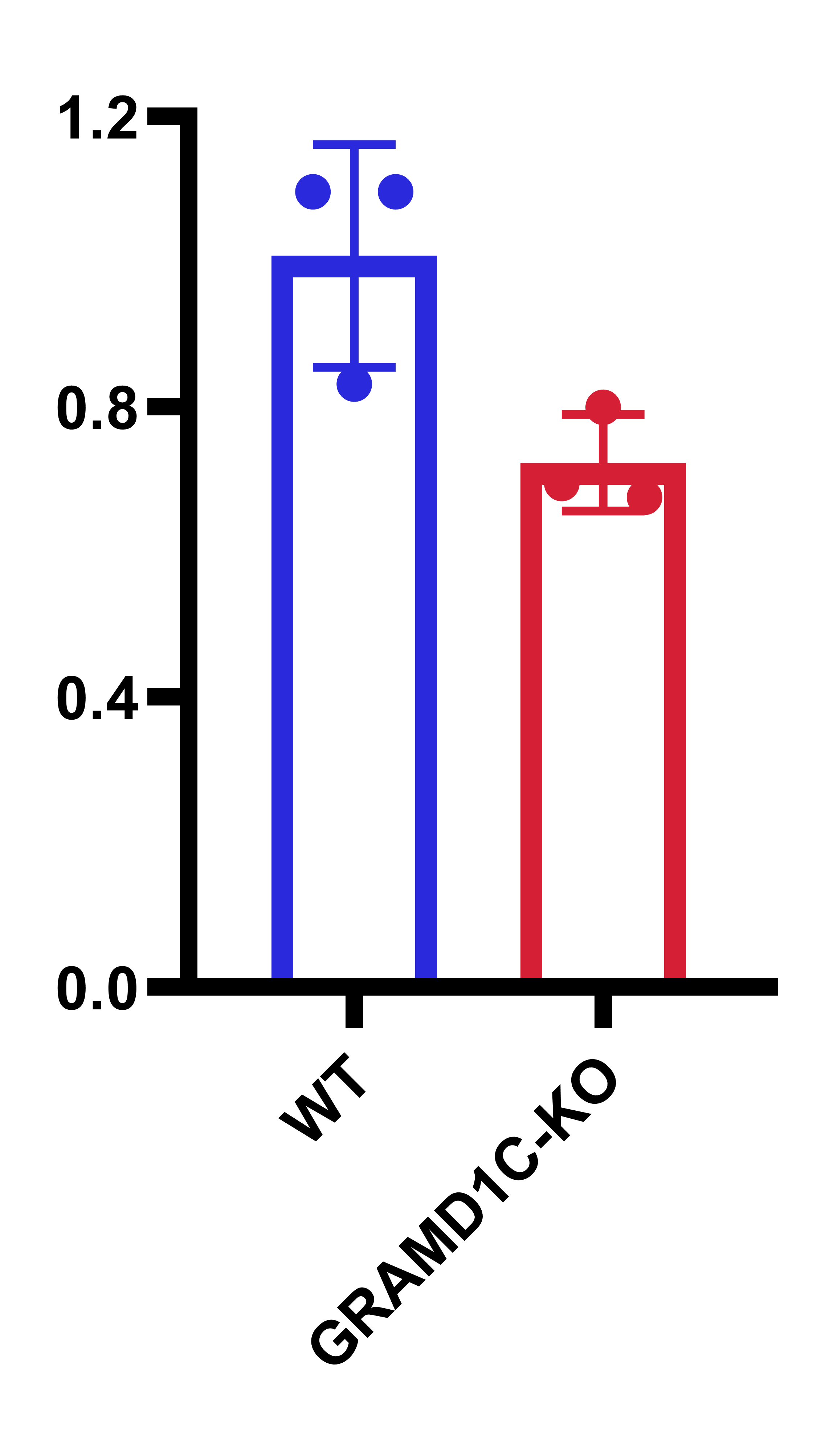

Supplement: S5 Data — This compressed folder contains the underlying numerical data and/or uncropped images used to generate the panels in Figs 6 and S1–S6, and S11. (ZIP) [file pbio.3003736.s019.zip › S5 Data/Supporting Information/Supporting Information fig2/B.C.D.ANPEP-ace2-CEACAM1-QPCR/ACE2.tif]

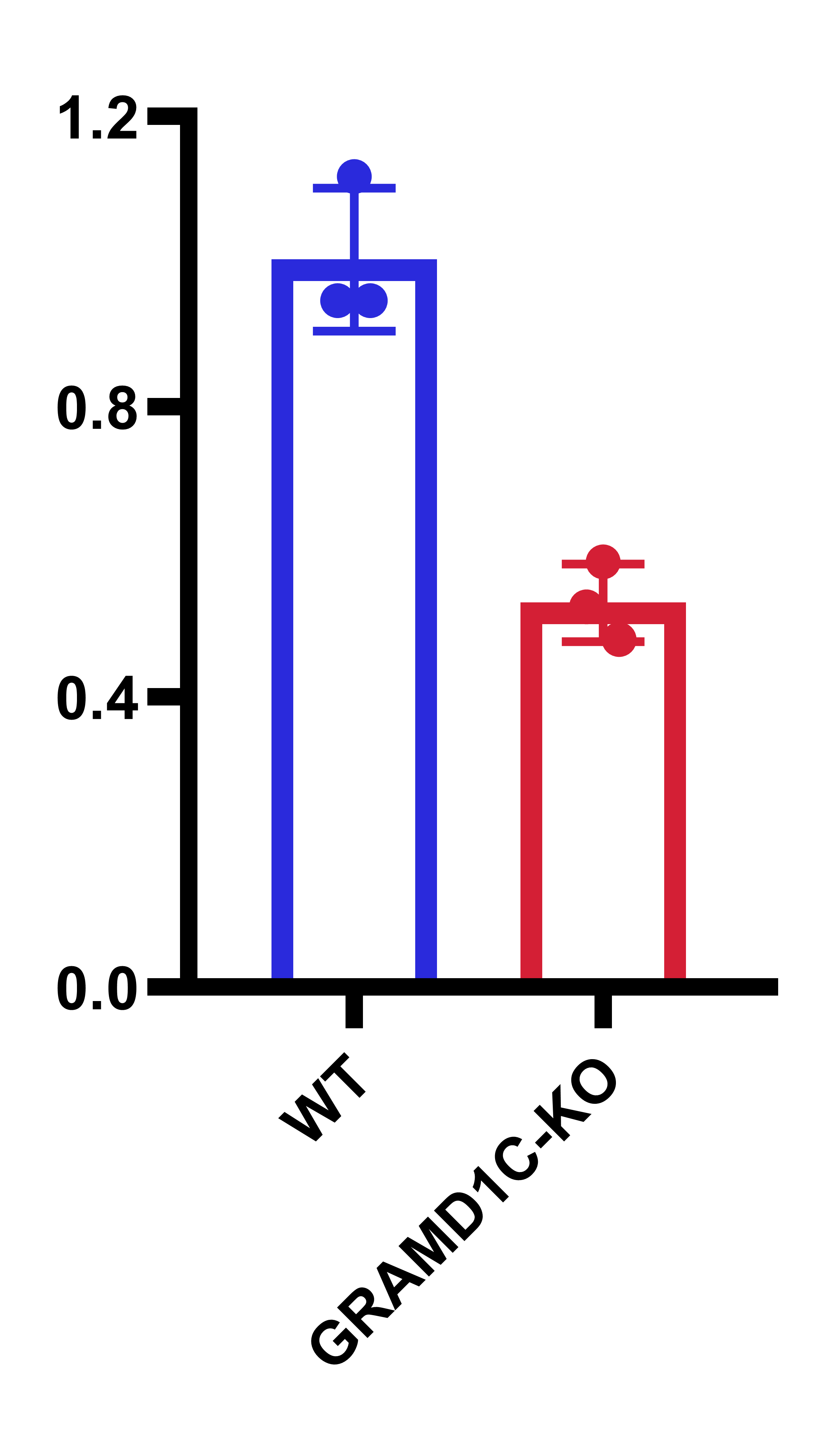

Supplement: S5 Data — This compressed folder contains the underlying numerical data and/or uncropped images used to generate the panels in Figs 6 and S1–S6, and S11. (ZIP) [file pbio.3003736.s019.zip › S5 Data/Supporting Information/Supporting Information fig2/B.C.D.ANPEP-ace2-CEACAM1-QPCR/ANPEP.tif]

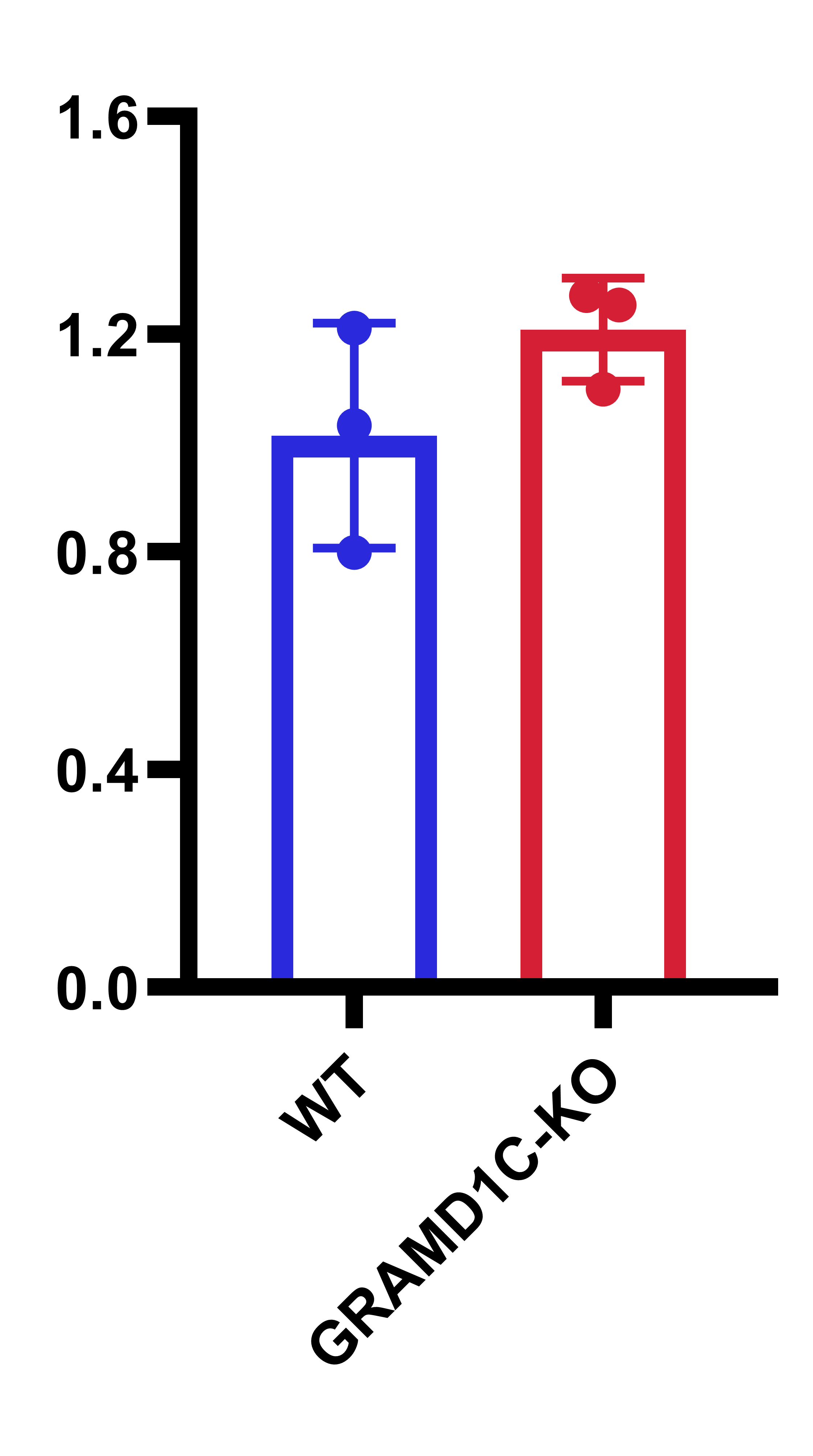

Supplement: S5 Data — This compressed folder contains the underlying numerical data and/or uncropped images used to generate the panels in Figs 6 and S1–S6, and S11. (ZIP) [file pbio.3003736.s019.zip › S5 Data/Supporting Information/Supporting Information fig2/B.C.D.ANPEP-ace2-CEACAM1-QPCR/CEACAM1.tif]

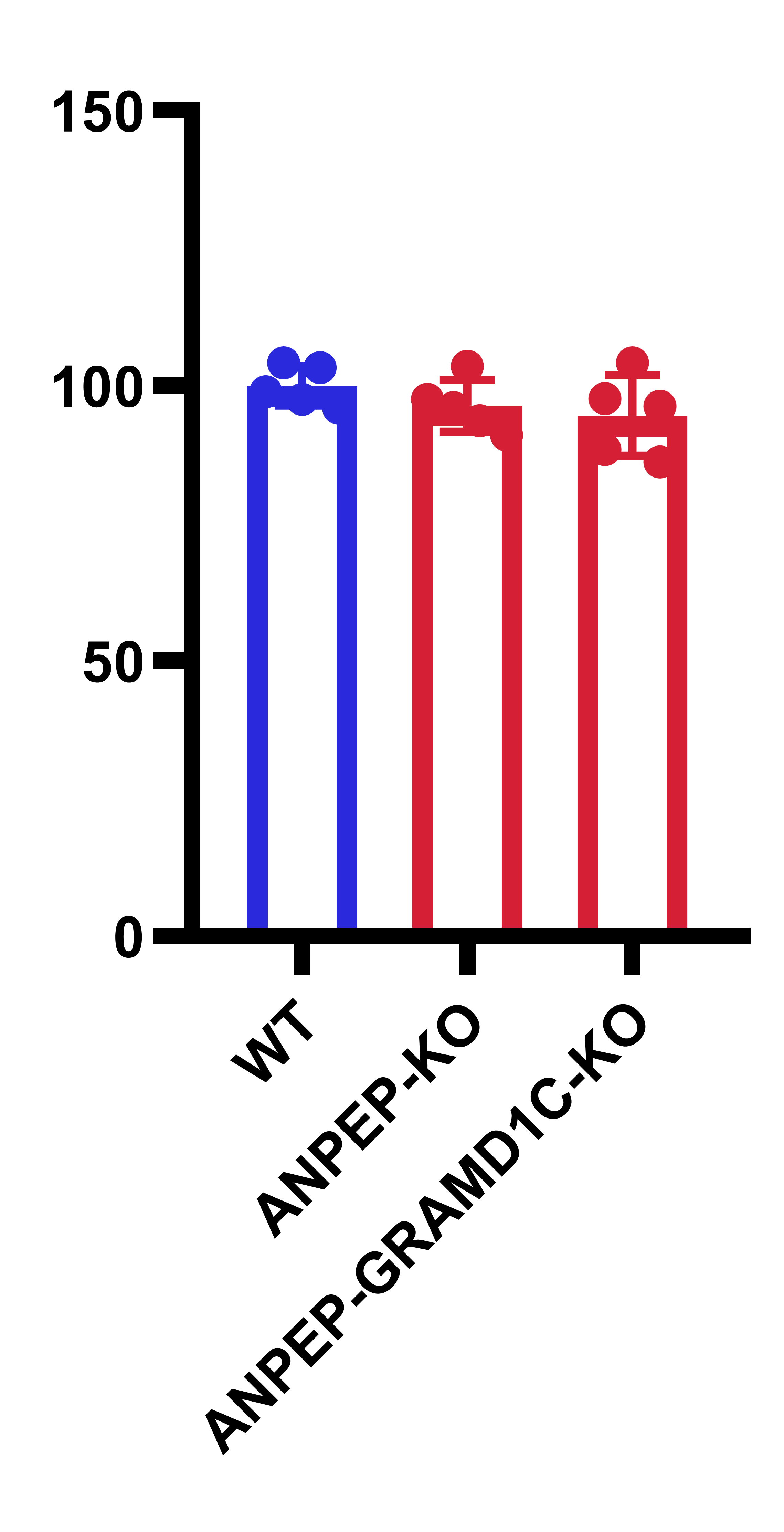

Supplement: S5 Data — This compressed folder contains the underlying numerical data and/or uncropped images used to generate the panels in Figs 6 and S1–S6, and S11. (ZIP) [file pbio.3003736.s019.zip › S5 Data/Supporting Information/Supporting Information fig3/B.MTS/Copy of Data 1.tif]

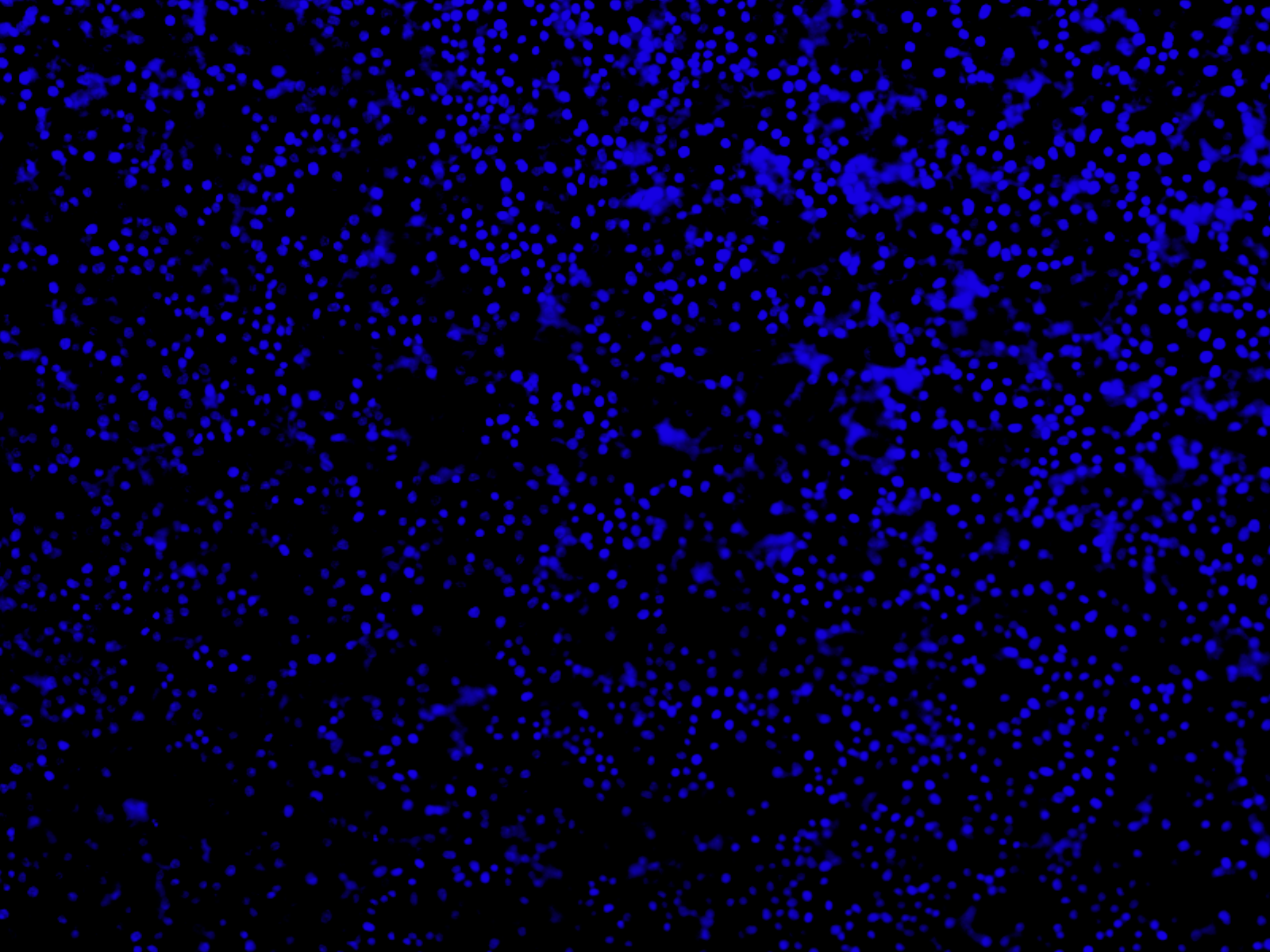

Supplement: S5 Data — This compressed folder contains the underlying numerical data and/or uncropped images used to generate the panels in Figs 6 and S1–S6, and S11. (ZIP) [file pbio.3003736.s019.zip › S5 Data/Supporting Information/Supporting Information fig4/A.PLVX-MCHERRY/apn-1C-ko/6-dapi.png]

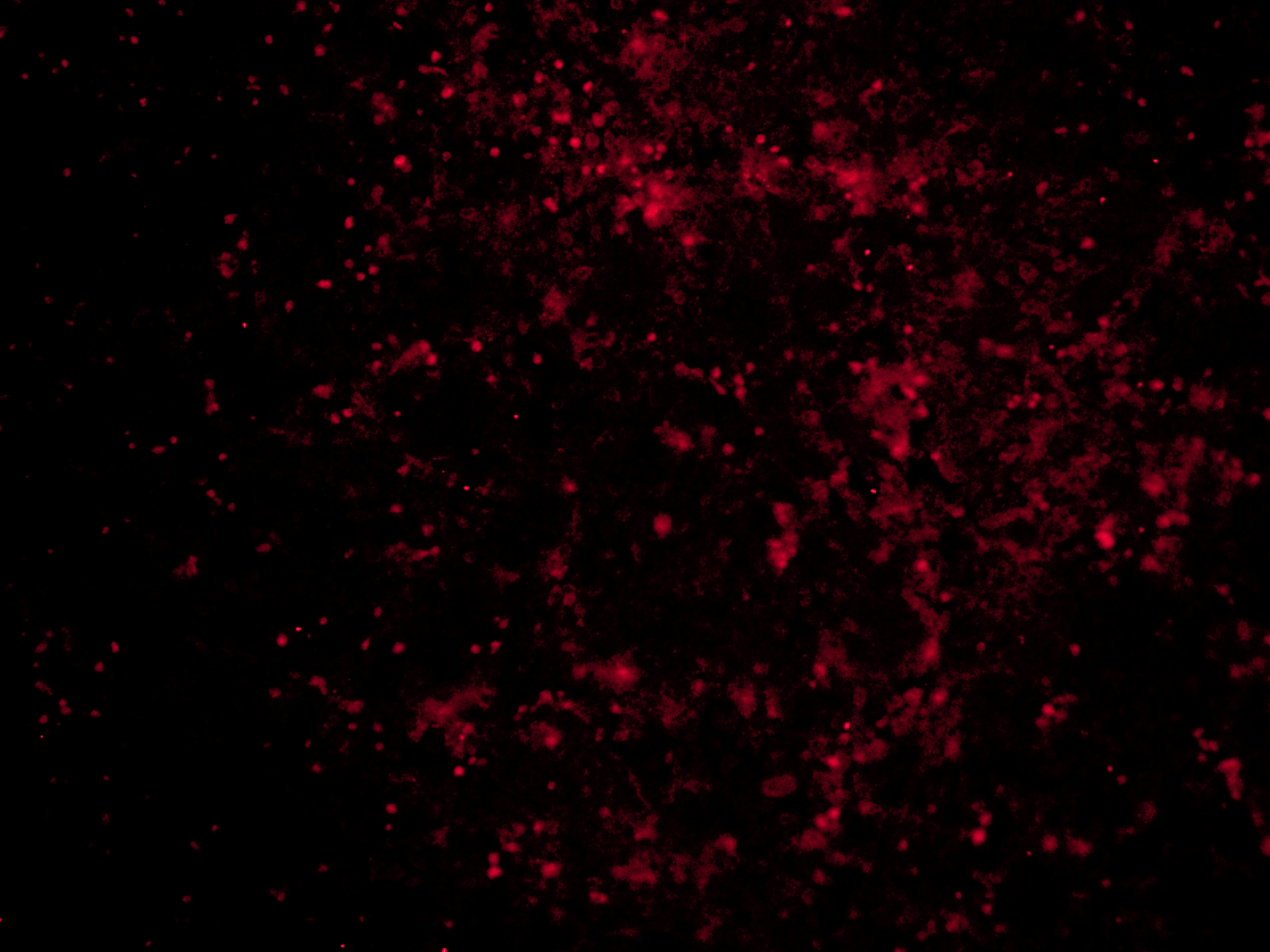

Supplement: S5 Data — This compressed folder contains the underlying numerical data and/or uncropped images used to generate the panels in Figs 6 and S1–S6, and S11. (ZIP) [file pbio.3003736.s019.zip › S5 Data/Supporting Information/Supporting Information fig4/A.PLVX-MCHERRY/apn-1C-ko/6-mcherry.png]
