# Supplementary figures and images for: The non-vesicular cholesterol transporter GRAMD1C is a pan-coronavirus antiviral target (part 4 of 4)
Source: PLoS Biol. 2026 Apr 6;24(4):e3003736. doi: 10.1371/journal.pbio.3003736 (PMC13068348; doi:10.1371/journal.pbio.3003736)

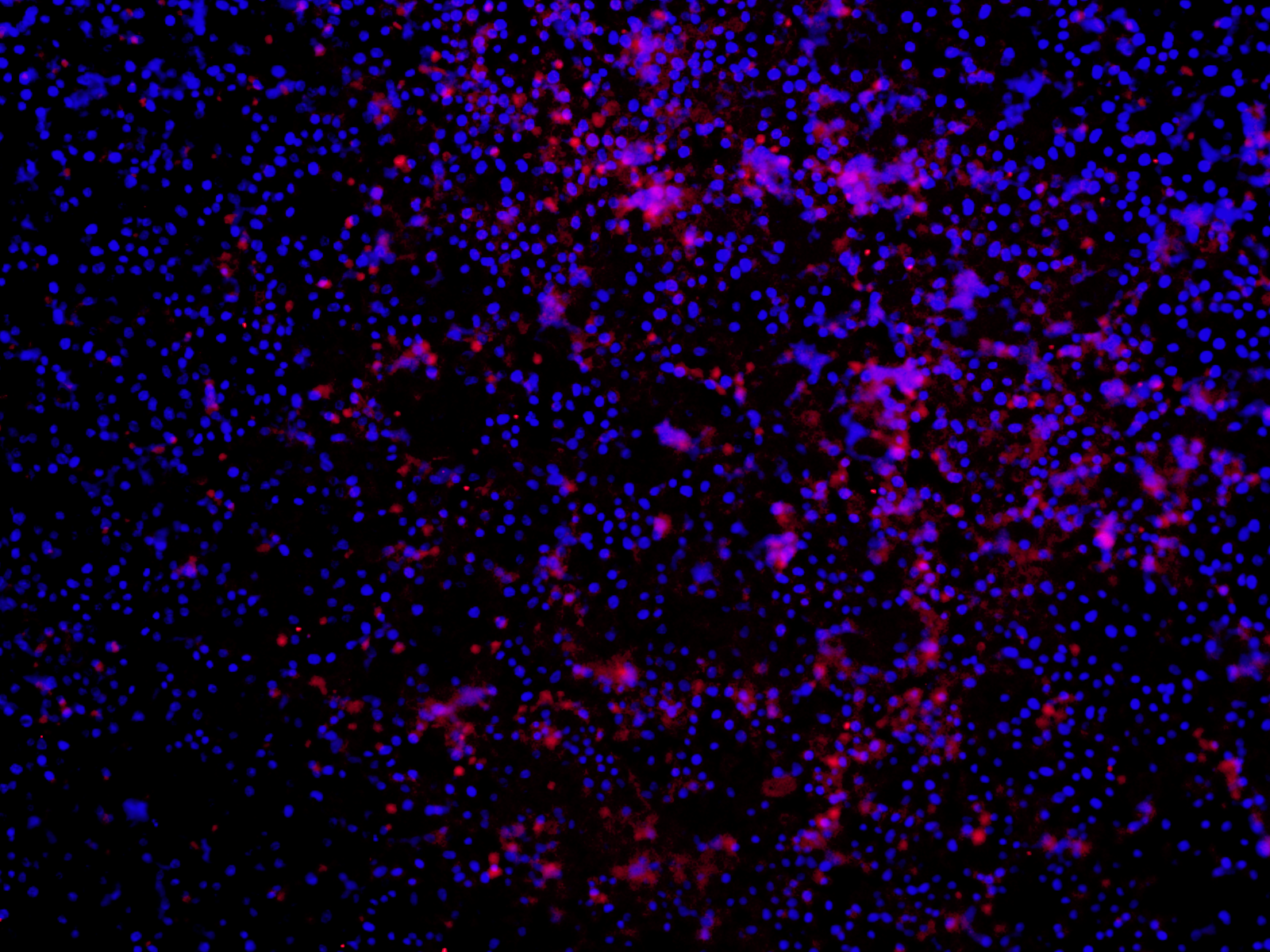

Supplement: S5 Data — This compressed folder contains the underlying numerical data and/or uncropped images used to generate the panels in Figs 6 and S1–S6, and S11. (ZIP) [file pbio.3003736.s019.zip › S5 Data/Supporting Information/Supporting Information fig4/A.PLVX-MCHERRY/apn-1C-ko/6.png]

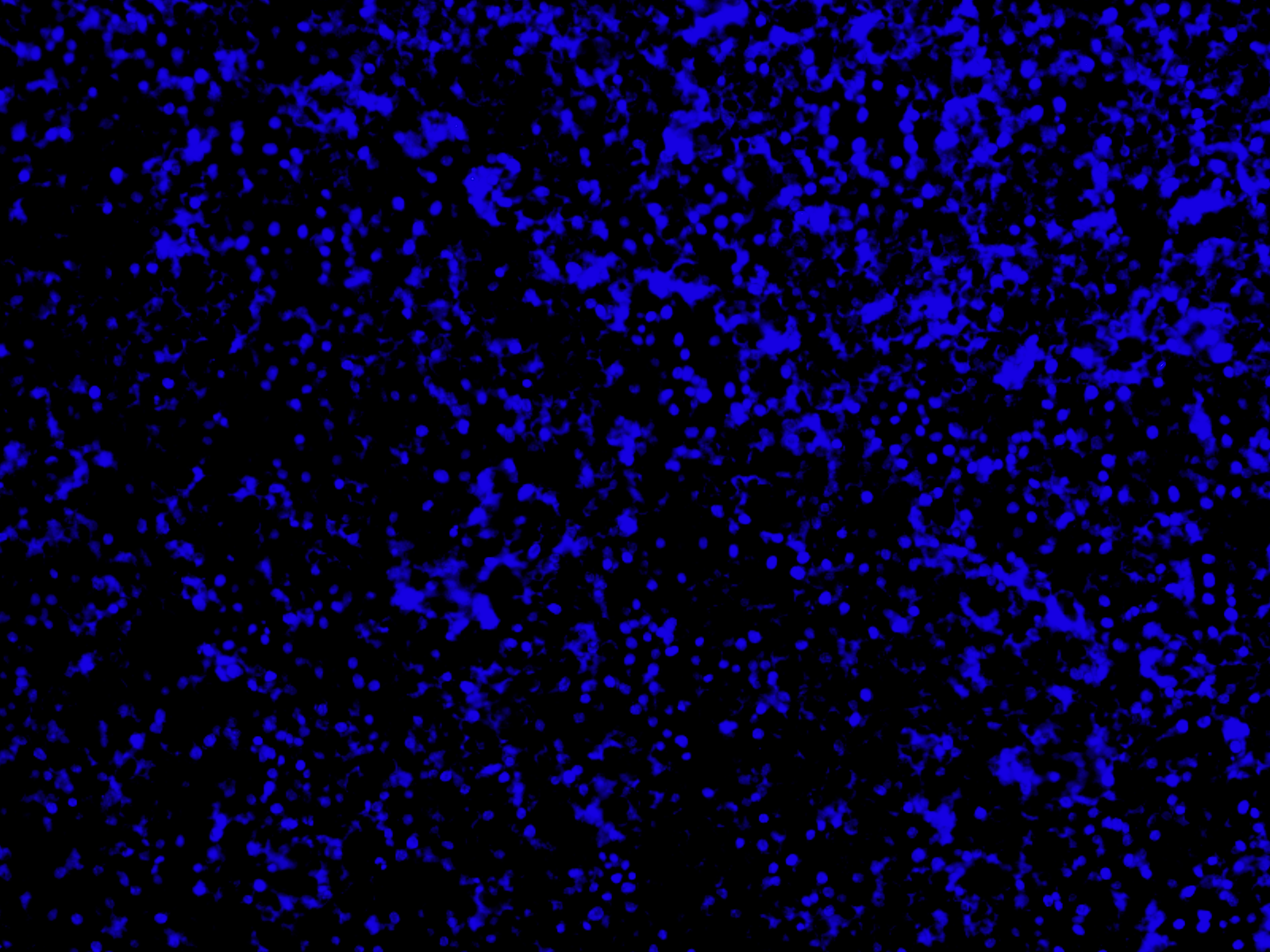

Supplement: S5 Data — This compressed folder contains the underlying numerical data and/or uncropped images used to generate the panels in Figs 6 and S1–S6, and S11. (ZIP) [file pbio.3003736.s019.zip › S5 Data/Supporting Information/Supporting Information fig4/A.PLVX-MCHERRY/apn-1C-ko/7-dapi.png]

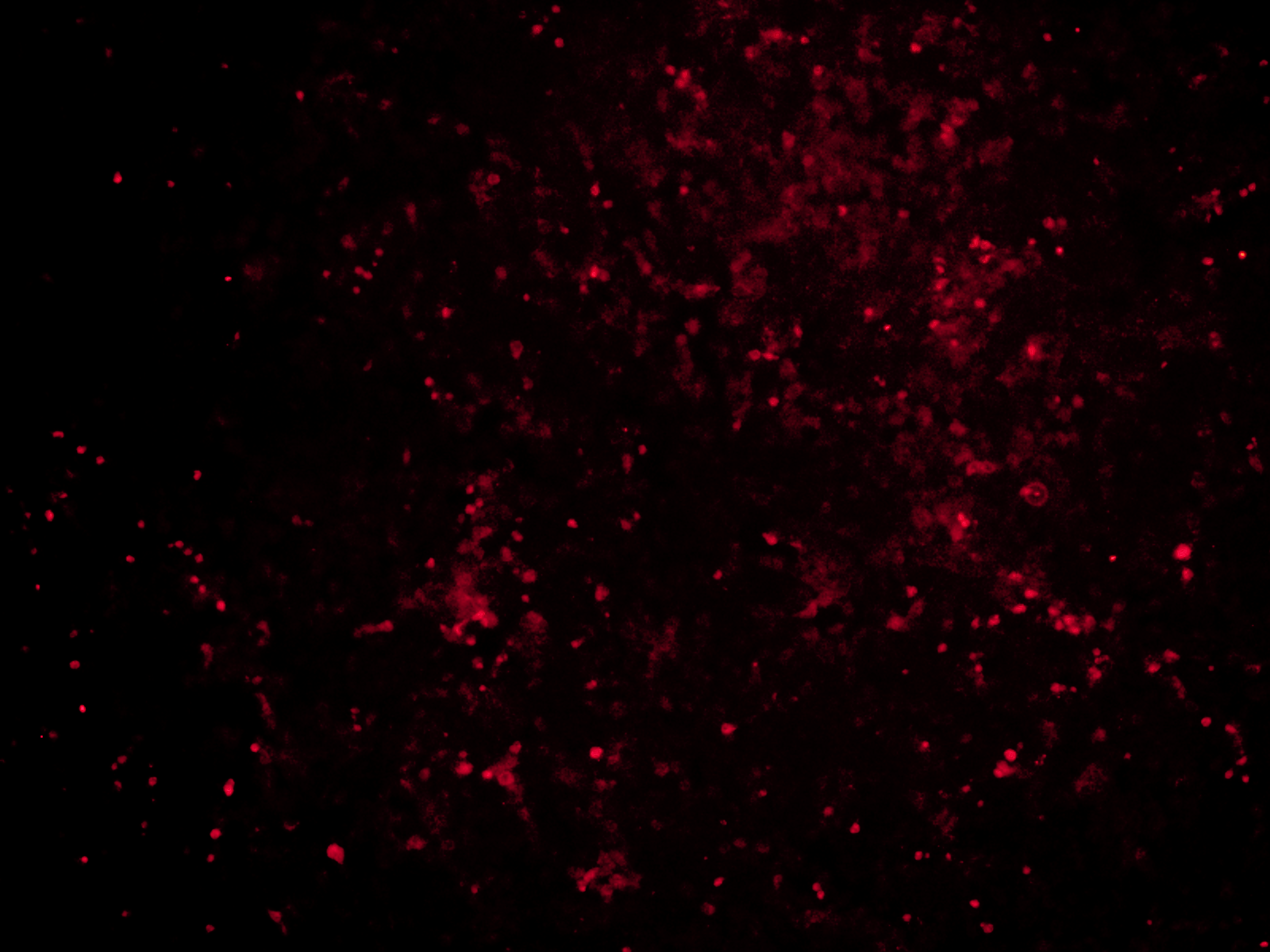

Supplement: S5 Data — This compressed folder contains the underlying numerical data and/or uncropped images used to generate the panels in Figs 6 and S1–S6, and S11. (ZIP) [file pbio.3003736.s019.zip › S5 Data/Supporting Information/Supporting Information fig4/A.PLVX-MCHERRY/apn-1C-ko/7-mcherry.png]

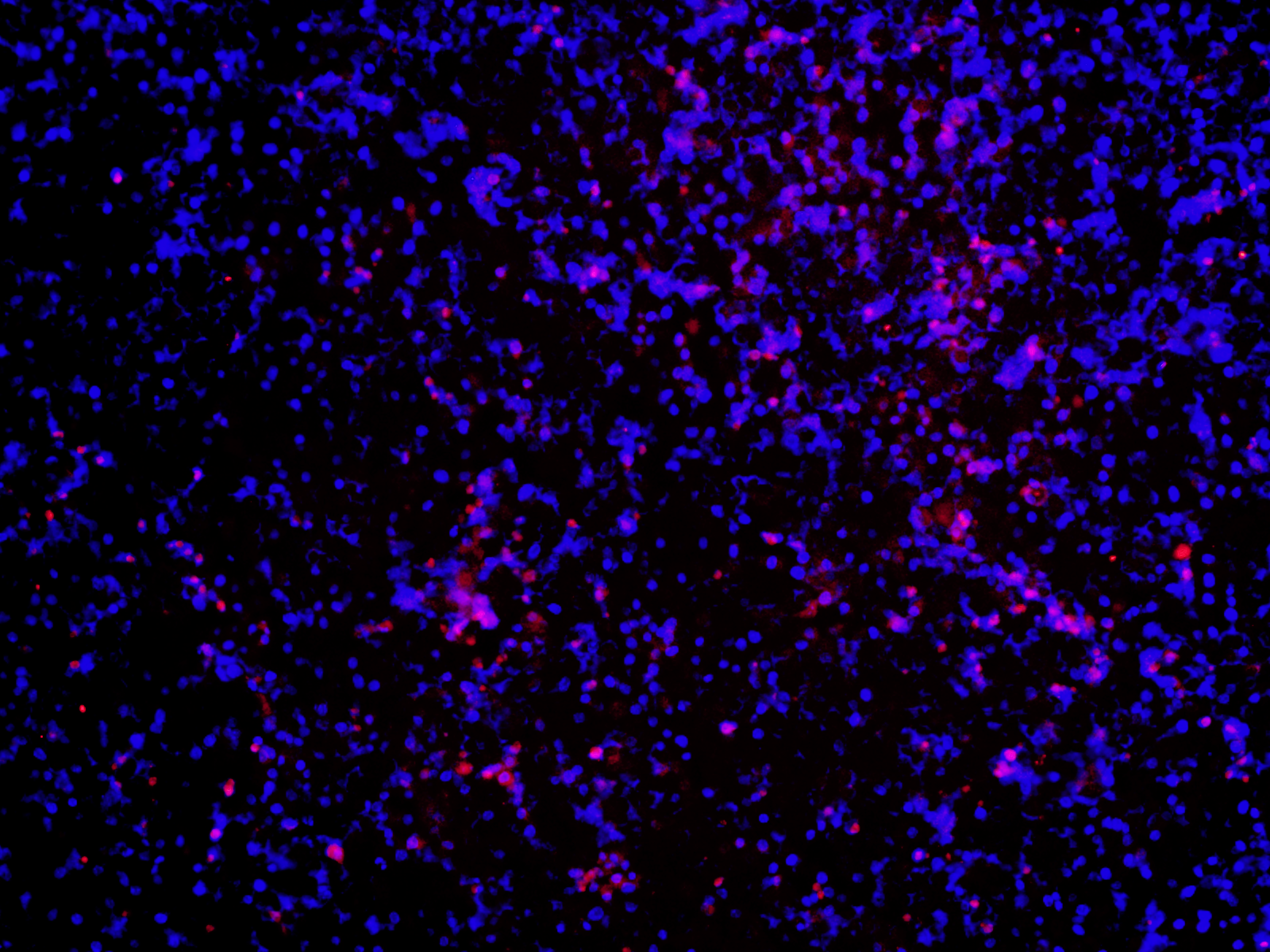

Supplement: S5 Data — This compressed folder contains the underlying numerical data and/or uncropped images used to generate the panels in Figs 6 and S1–S6, and S11. (ZIP) [file pbio.3003736.s019.zip › S5 Data/Supporting Information/Supporting Information fig4/A.PLVX-MCHERRY/apn-1C-ko/7.png]

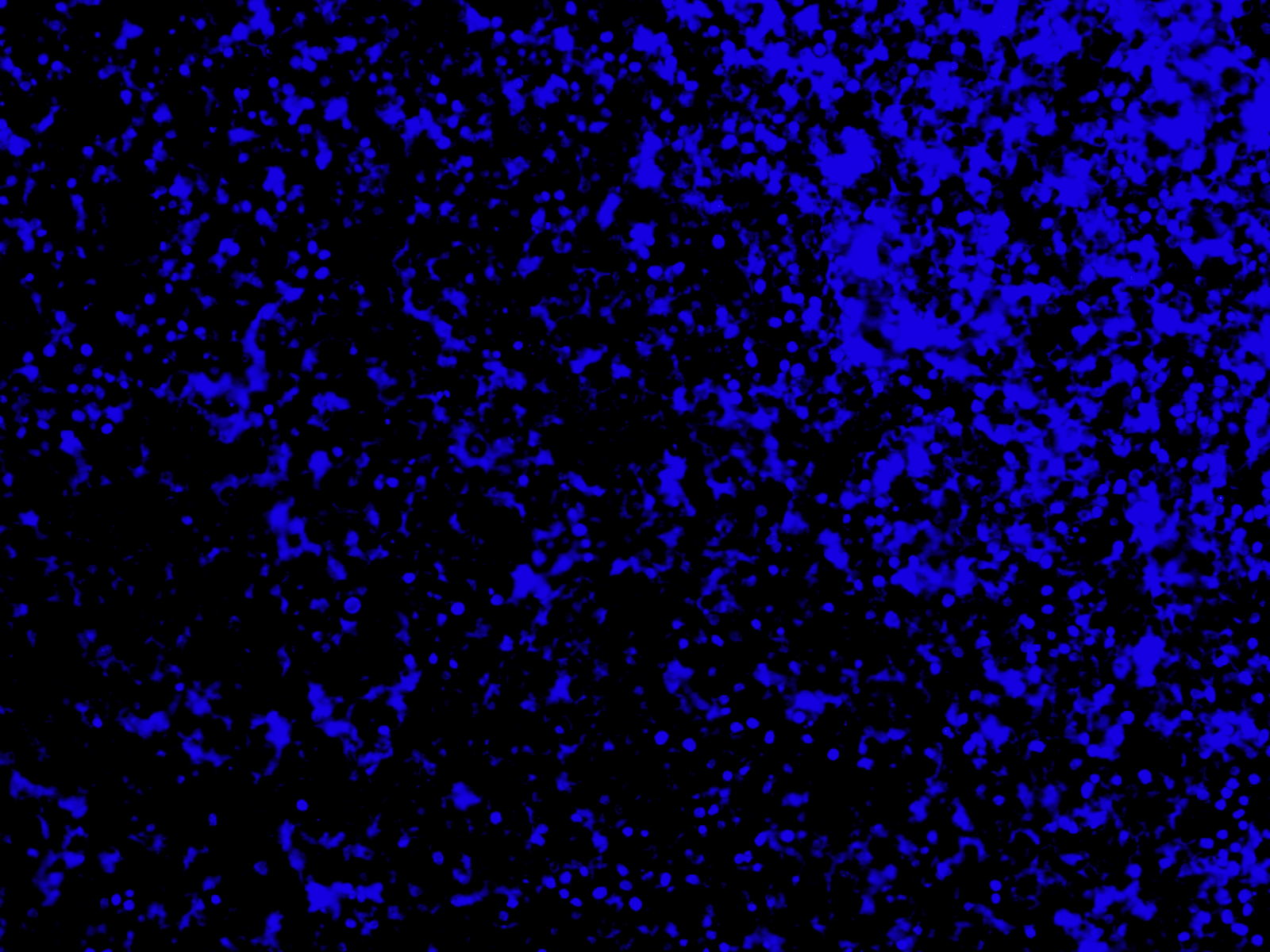

Supplement: S5 Data — This compressed folder contains the underlying numerical data and/or uncropped images used to generate the panels in Figs 6 and S1–S6, and S11. (ZIP) [file pbio.3003736.s019.zip › S5 Data/Supporting Information/Supporting Information fig4/A.PLVX-MCHERRY/apn-1C-ko/8-dapi.png]

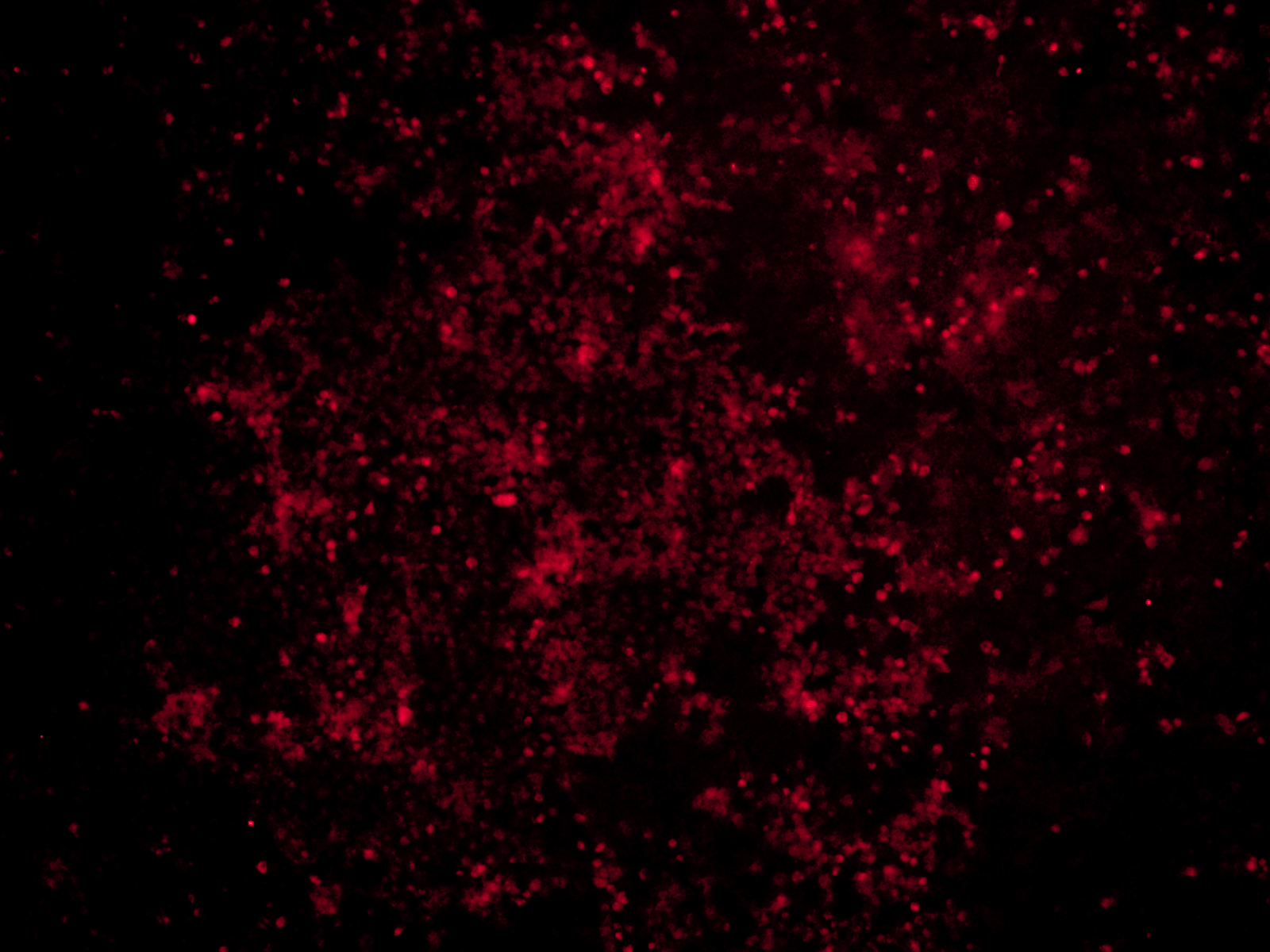

Supplement: S5 Data — This compressed folder contains the underlying numerical data and/or uncropped images used to generate the panels in Figs 6 and S1–S6, and S11. (ZIP) [file pbio.3003736.s019.zip › S5 Data/Supporting Information/Supporting Information fig4/A.PLVX-MCHERRY/apn-1C-ko/8-mcherry.png]

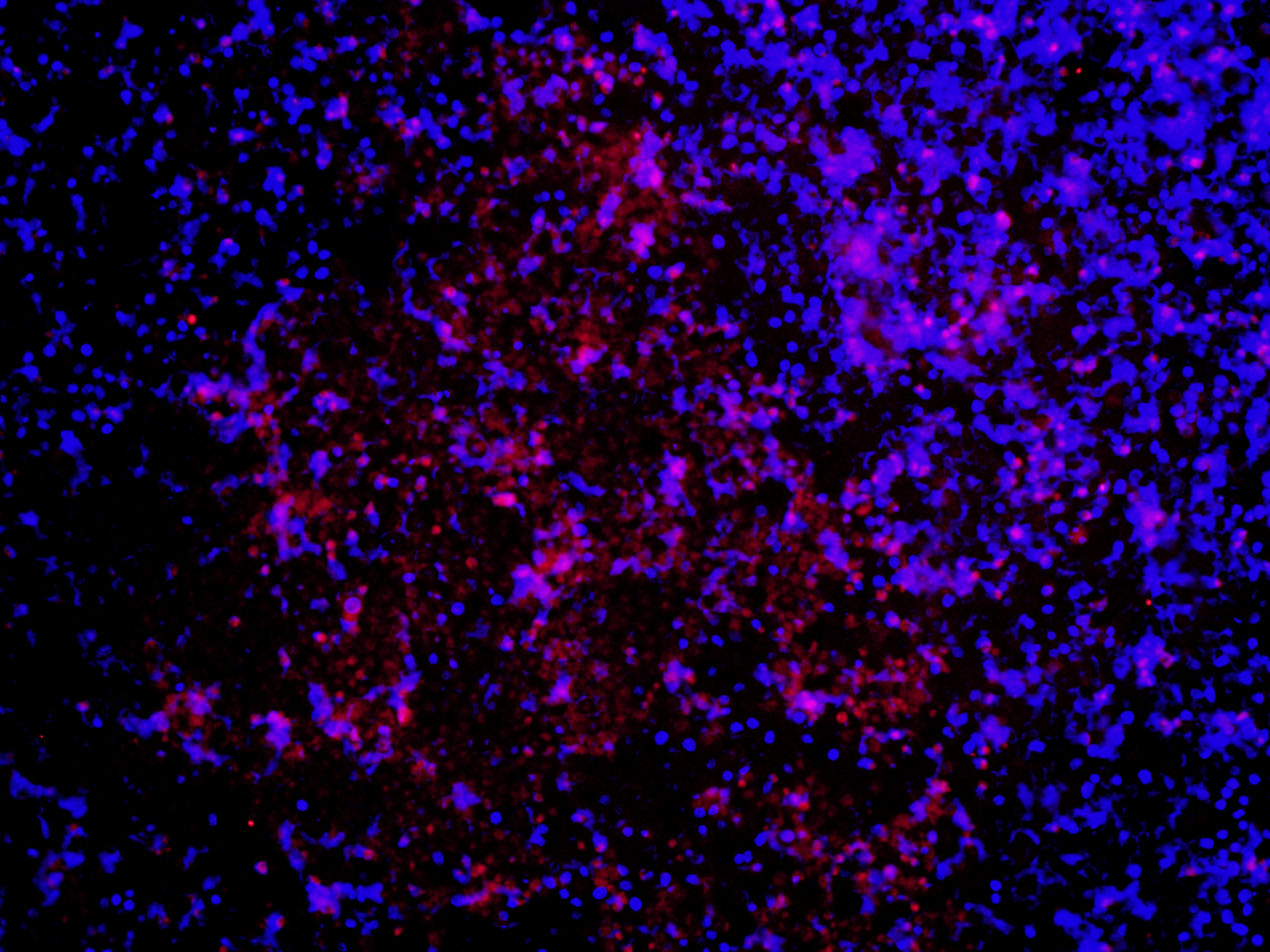

Supplement: S5 Data — This compressed folder contains the underlying numerical data and/or uncropped images used to generate the panels in Figs 6 and S1–S6, and S11. (ZIP) [file pbio.3003736.s019.zip › S5 Data/Supporting Information/Supporting Information fig4/A.PLVX-MCHERRY/apn-1C-ko/8.png]

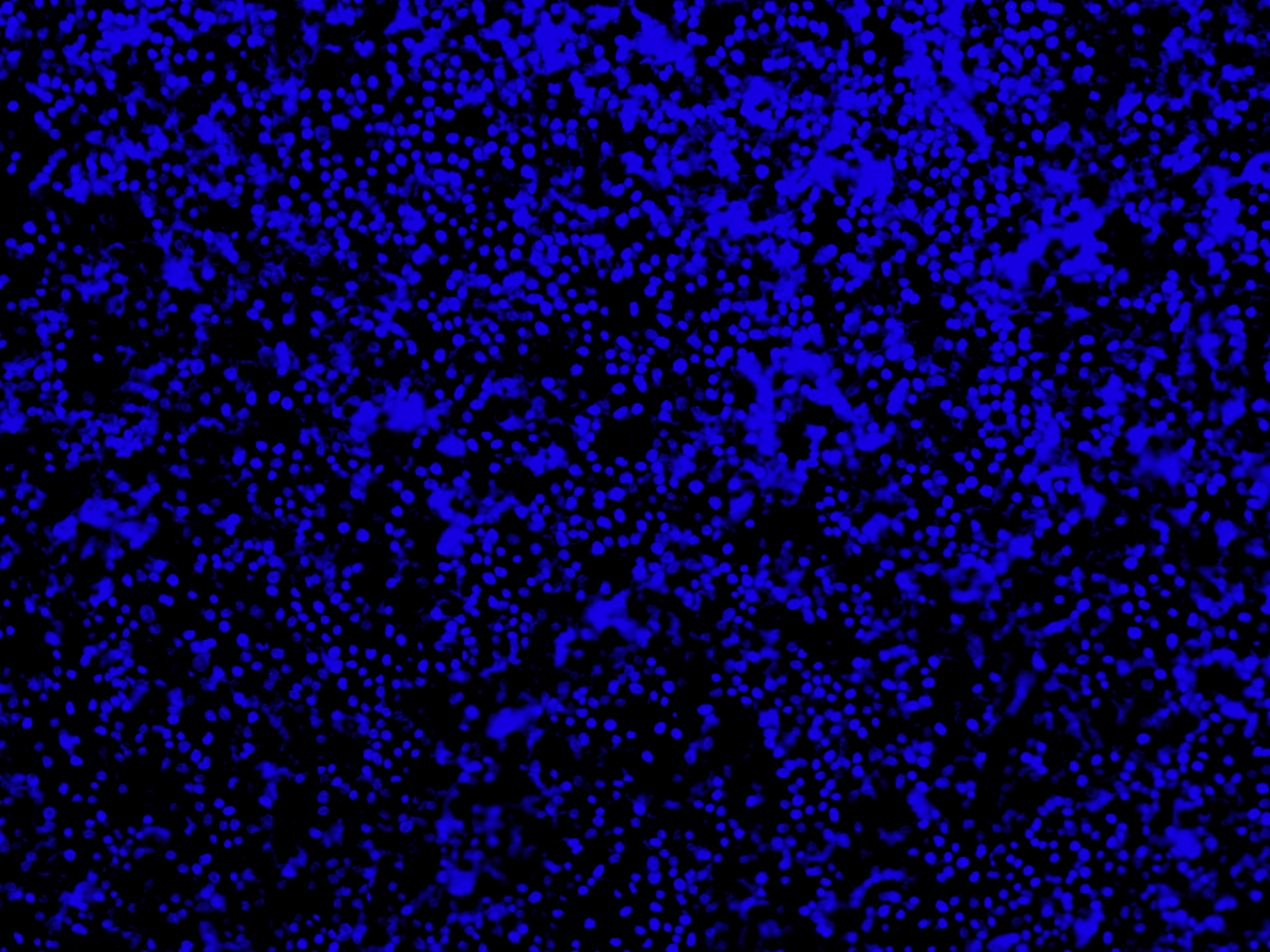

Supplement: S5 Data — This compressed folder contains the underlying numerical data and/or uncropped images used to generate the panels in Figs 6 and S1–S6, and S11. (ZIP) [file pbio.3003736.s019.zip › S5 Data/Supporting Information/Supporting Information fig4/A.PLVX-MCHERRY/apn-ko/3-dapi.png]

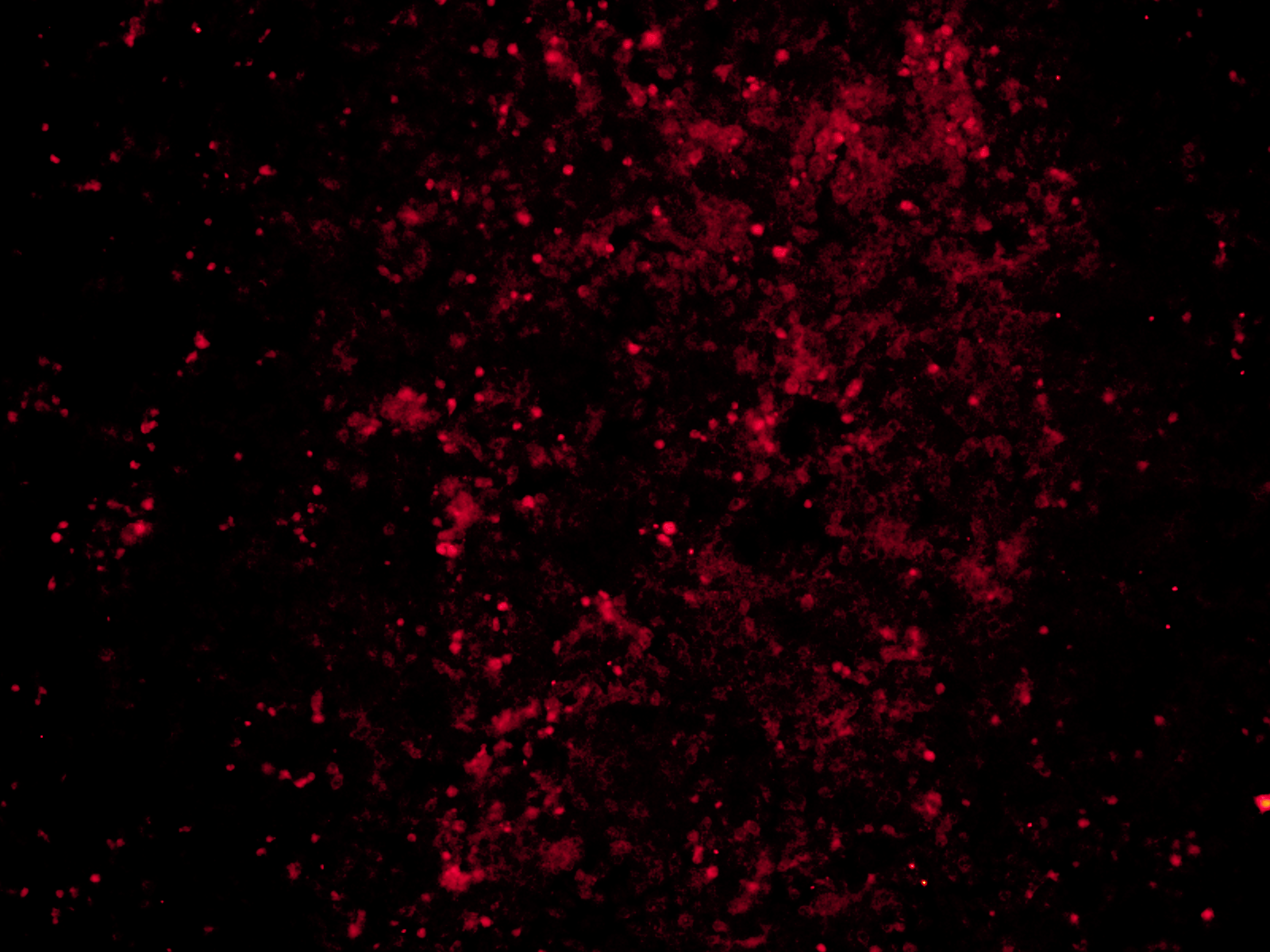

Supplement: S5 Data — This compressed folder contains the underlying numerical data and/or uncropped images used to generate the panels in Figs 6 and S1–S6, and S11. (ZIP) [file pbio.3003736.s019.zip › S5 Data/Supporting Information/Supporting Information fig4/A.PLVX-MCHERRY/apn-ko/3-mcherry.png]

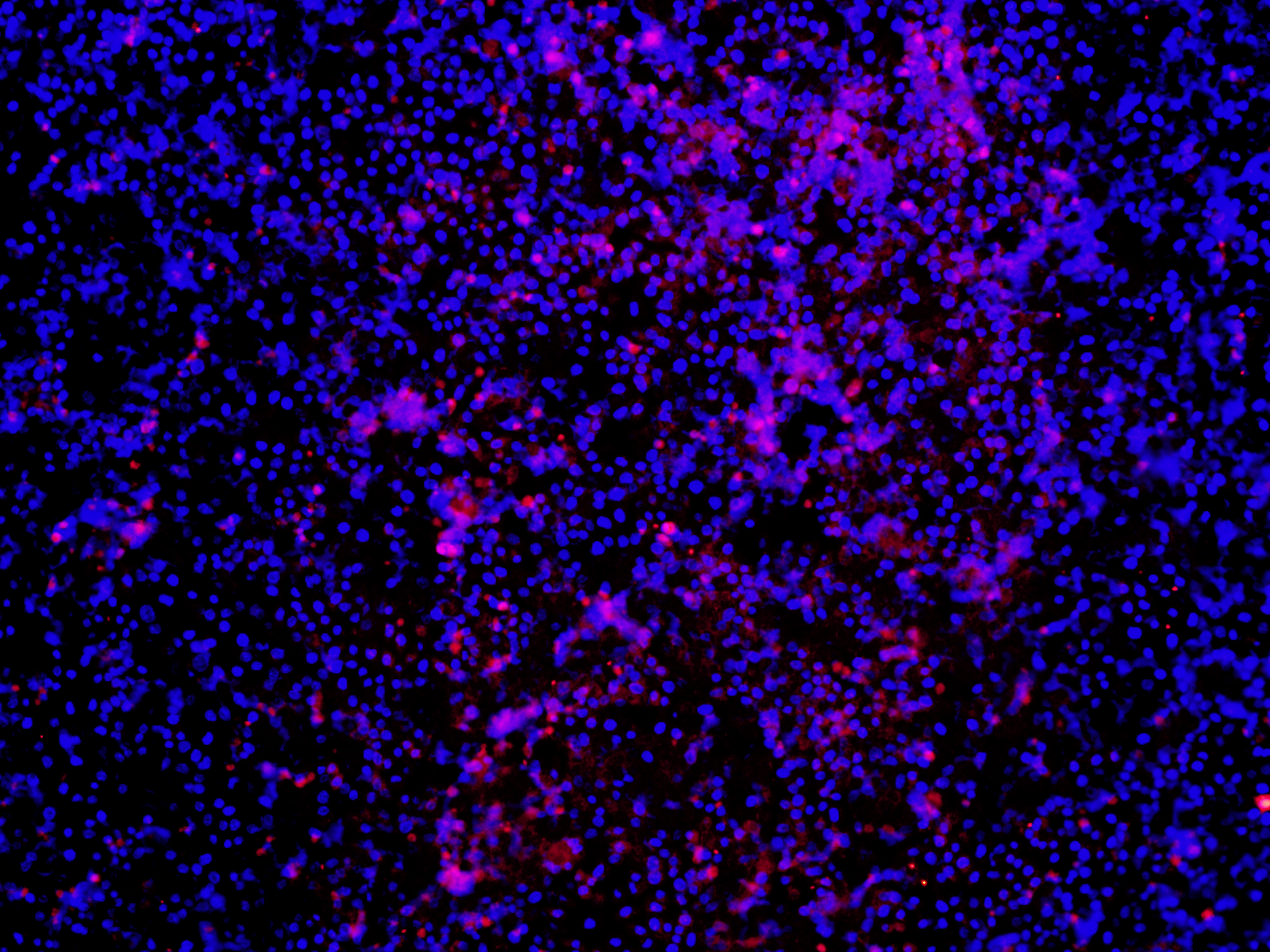

Supplement: S5 Data — This compressed folder contains the underlying numerical data and/or uncropped images used to generate the panels in Figs 6 and S1–S6, and S11. (ZIP) [file pbio.3003736.s019.zip › S5 Data/Supporting Information/Supporting Information fig4/A.PLVX-MCHERRY/apn-ko/3.png]

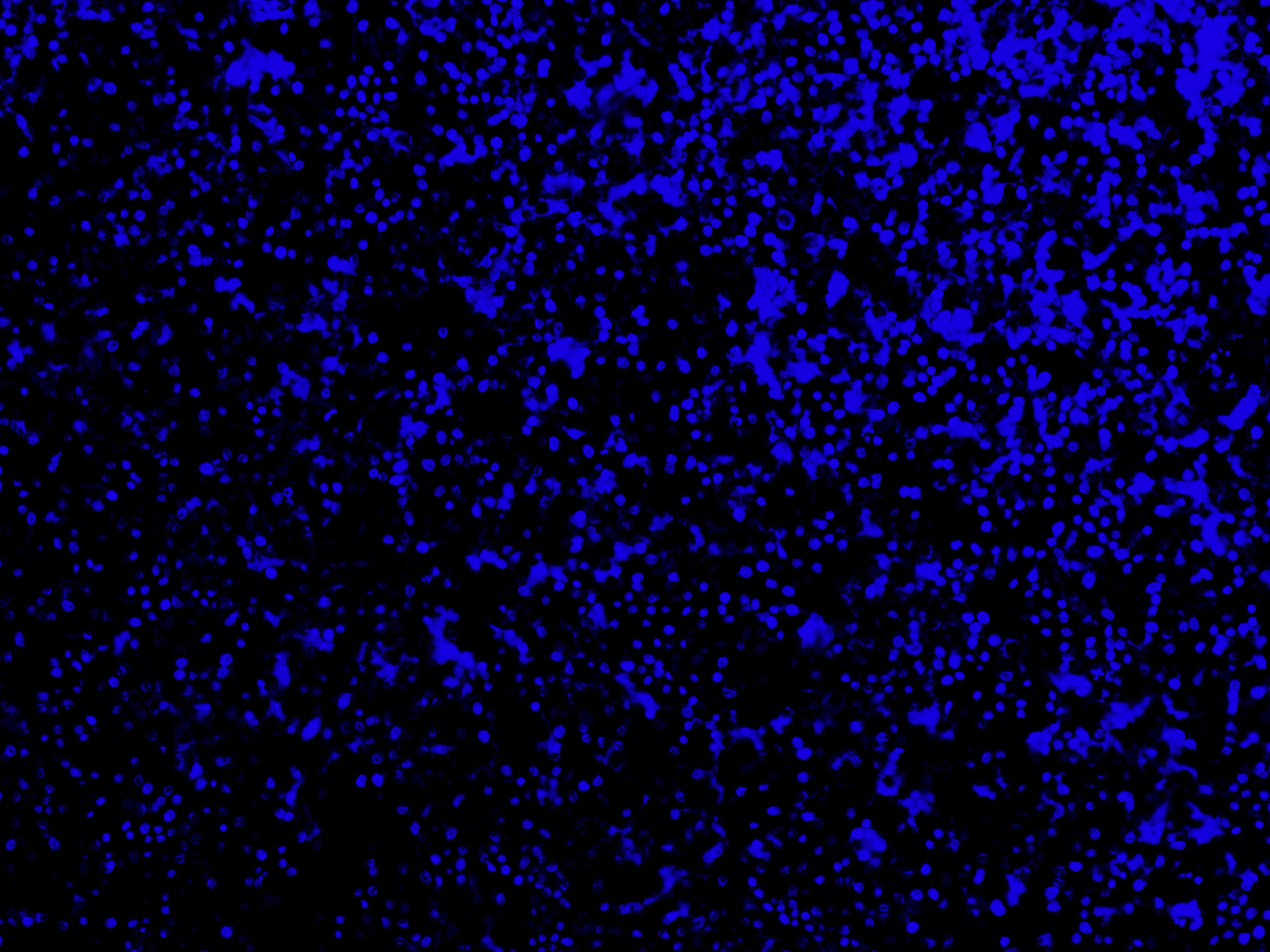

Supplement: S5 Data — This compressed folder contains the underlying numerical data and/or uncropped images used to generate the panels in Figs 6 and S1–S6, and S11. (ZIP) [file pbio.3003736.s019.zip › S5 Data/Supporting Information/Supporting Information fig4/A.PLVX-MCHERRY/apn-ko/4-dapi.png]

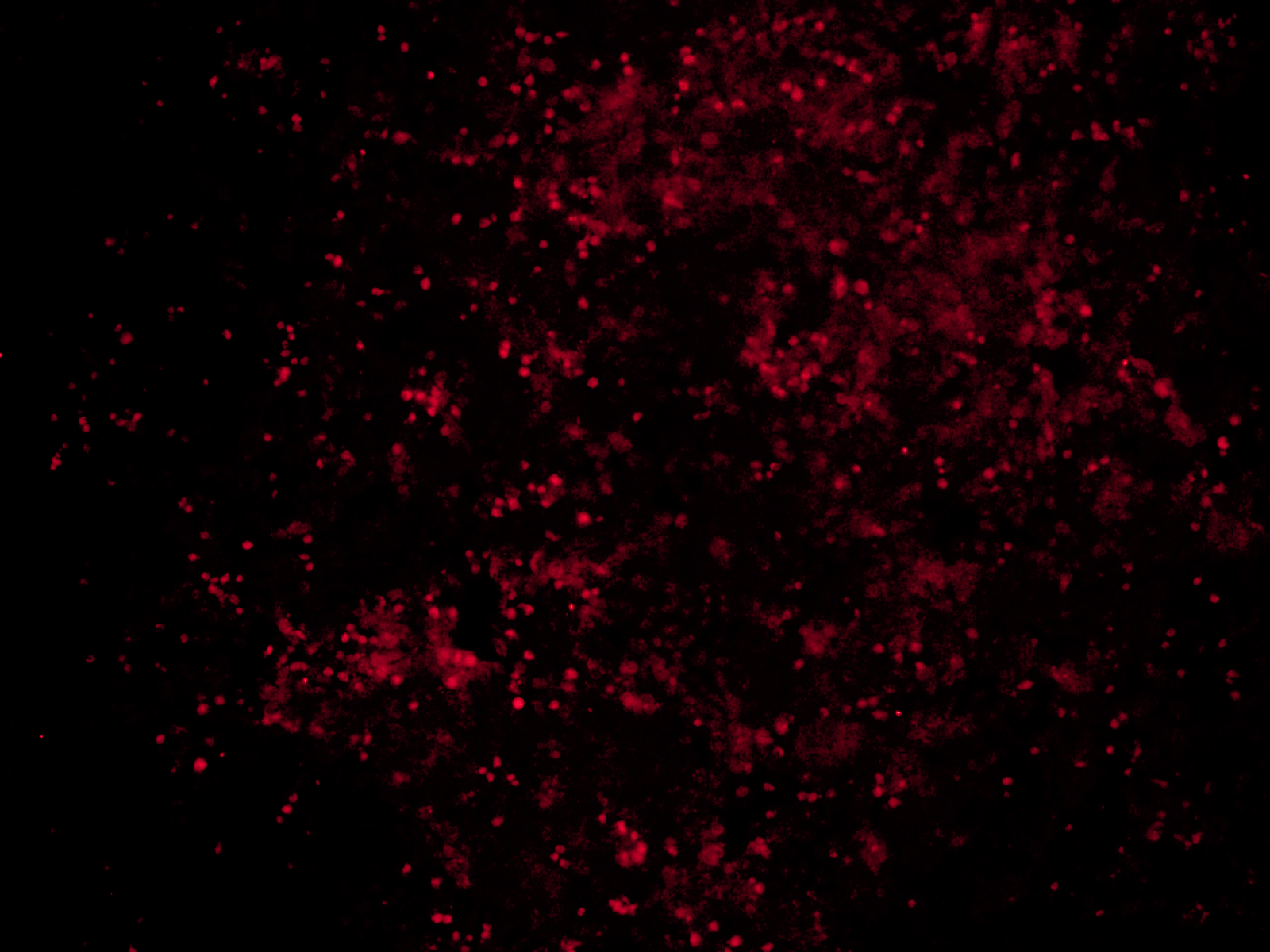

Supplement: S5 Data — This compressed folder contains the underlying numerical data and/or uncropped images used to generate the panels in Figs 6 and S1–S6, and S11. (ZIP) [file pbio.3003736.s019.zip › S5 Data/Supporting Information/Supporting Information fig4/A.PLVX-MCHERRY/apn-ko/4-mcheryy.png]

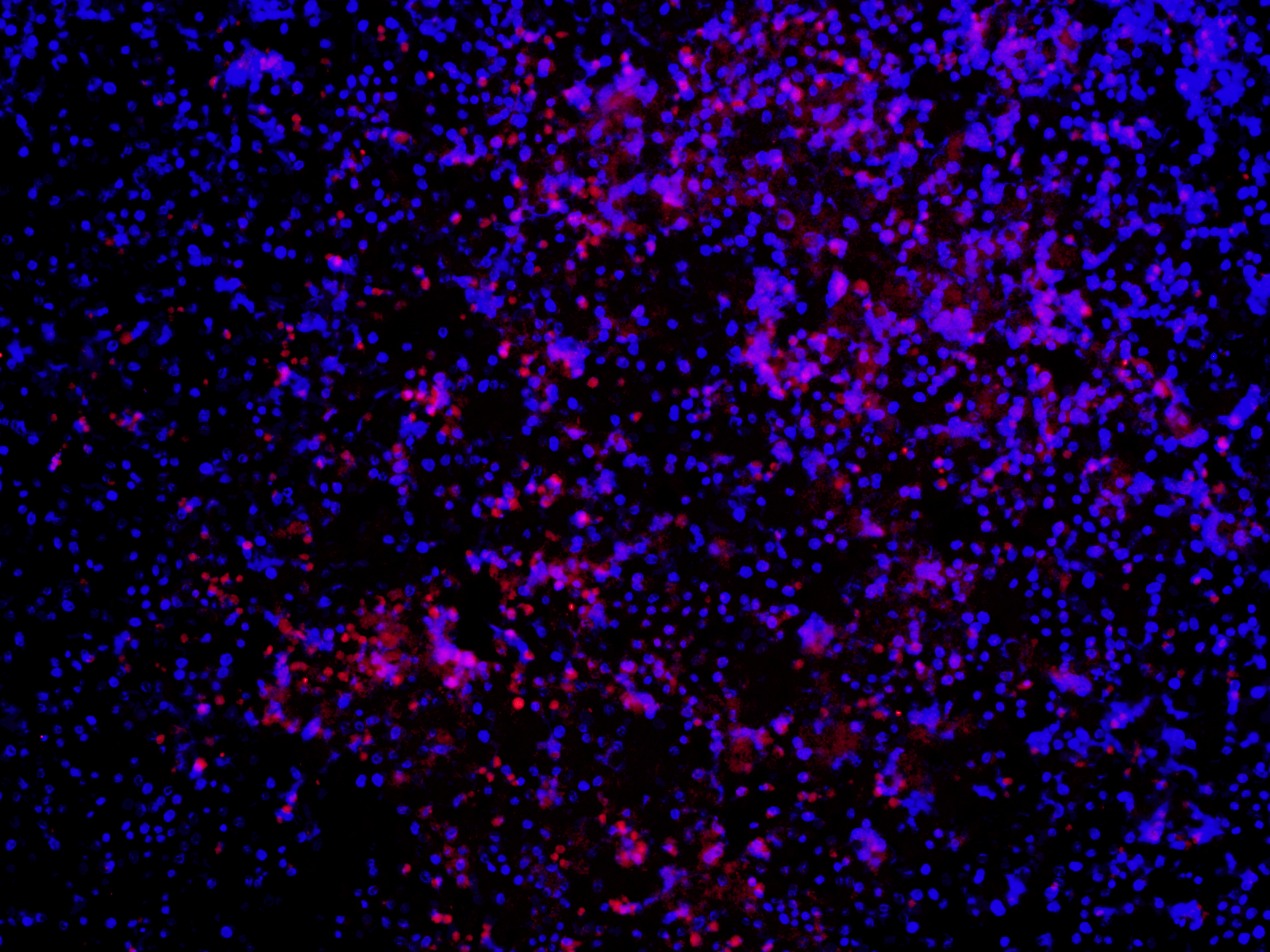

Supplement: S5 Data — This compressed folder contains the underlying numerical data and/or uncropped images used to generate the panels in Figs 6 and S1–S6, and S11. (ZIP) [file pbio.3003736.s019.zip › S5 Data/Supporting Information/Supporting Information fig4/A.PLVX-MCHERRY/apn-ko/4.png]

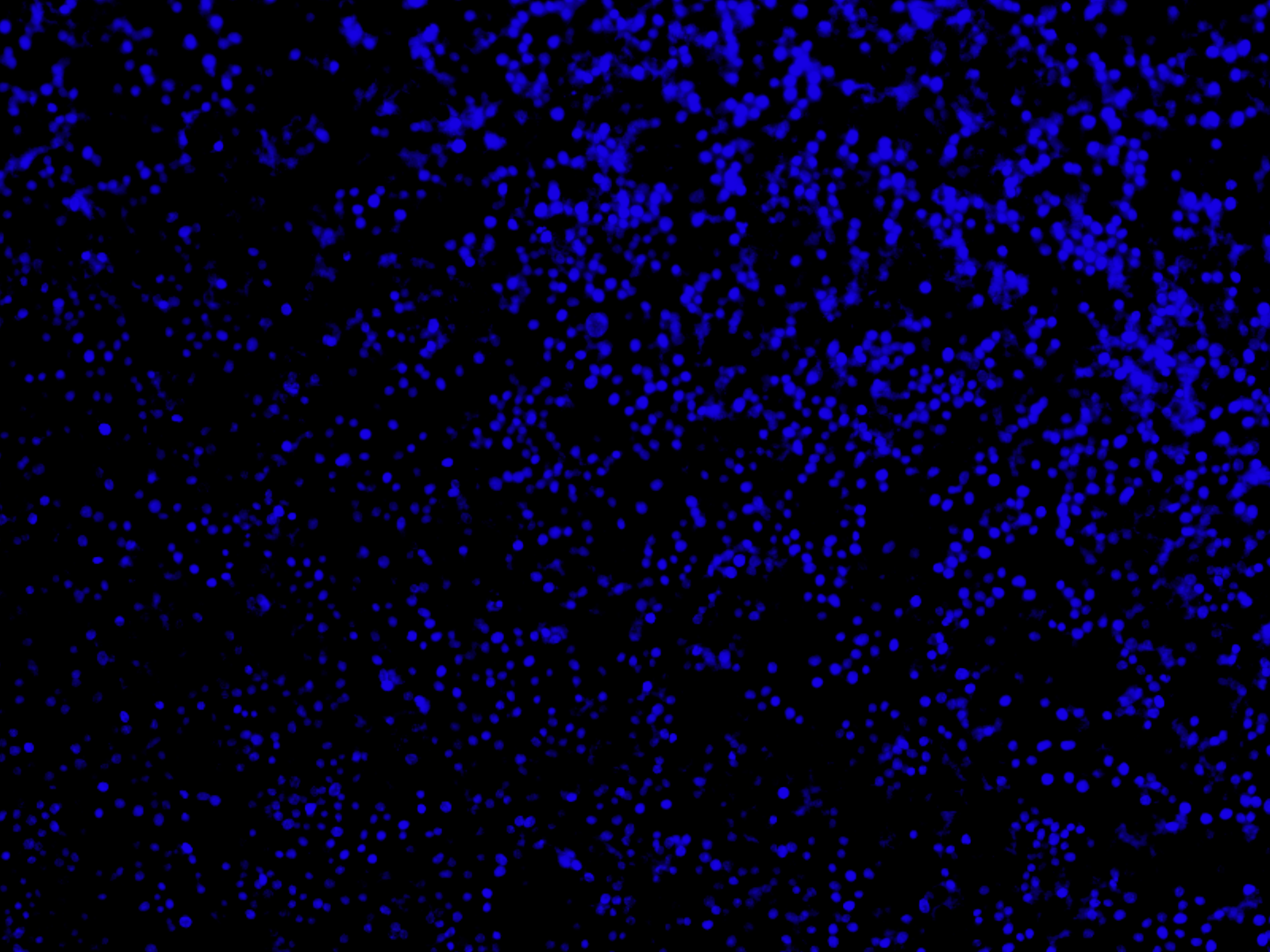

Supplement: S5 Data — This compressed folder contains the underlying numerical data and/or uncropped images used to generate the panels in Figs 6 and S1–S6, and S11. (ZIP) [file pbio.3003736.s019.zip › S5 Data/Supporting Information/Supporting Information fig4/A.PLVX-MCHERRY/apn-ko/5-dapi.png]

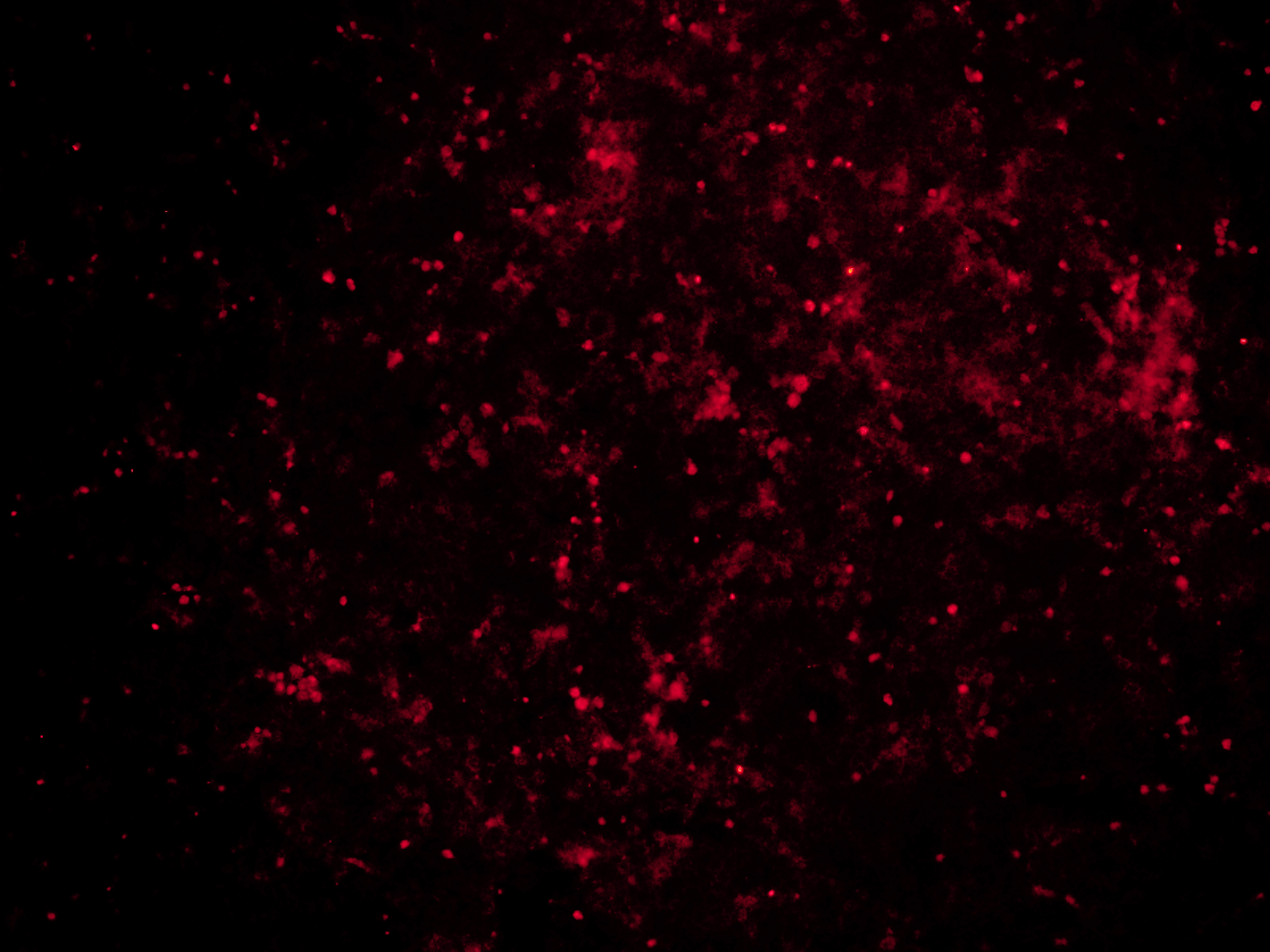

Supplement: S5 Data — This compressed folder contains the underlying numerical data and/or uncropped images used to generate the panels in Figs 6 and S1–S6, and S11. (ZIP) [file pbio.3003736.s019.zip › S5 Data/Supporting Information/Supporting Information fig4/A.PLVX-MCHERRY/apn-ko/5-mcherry.png]

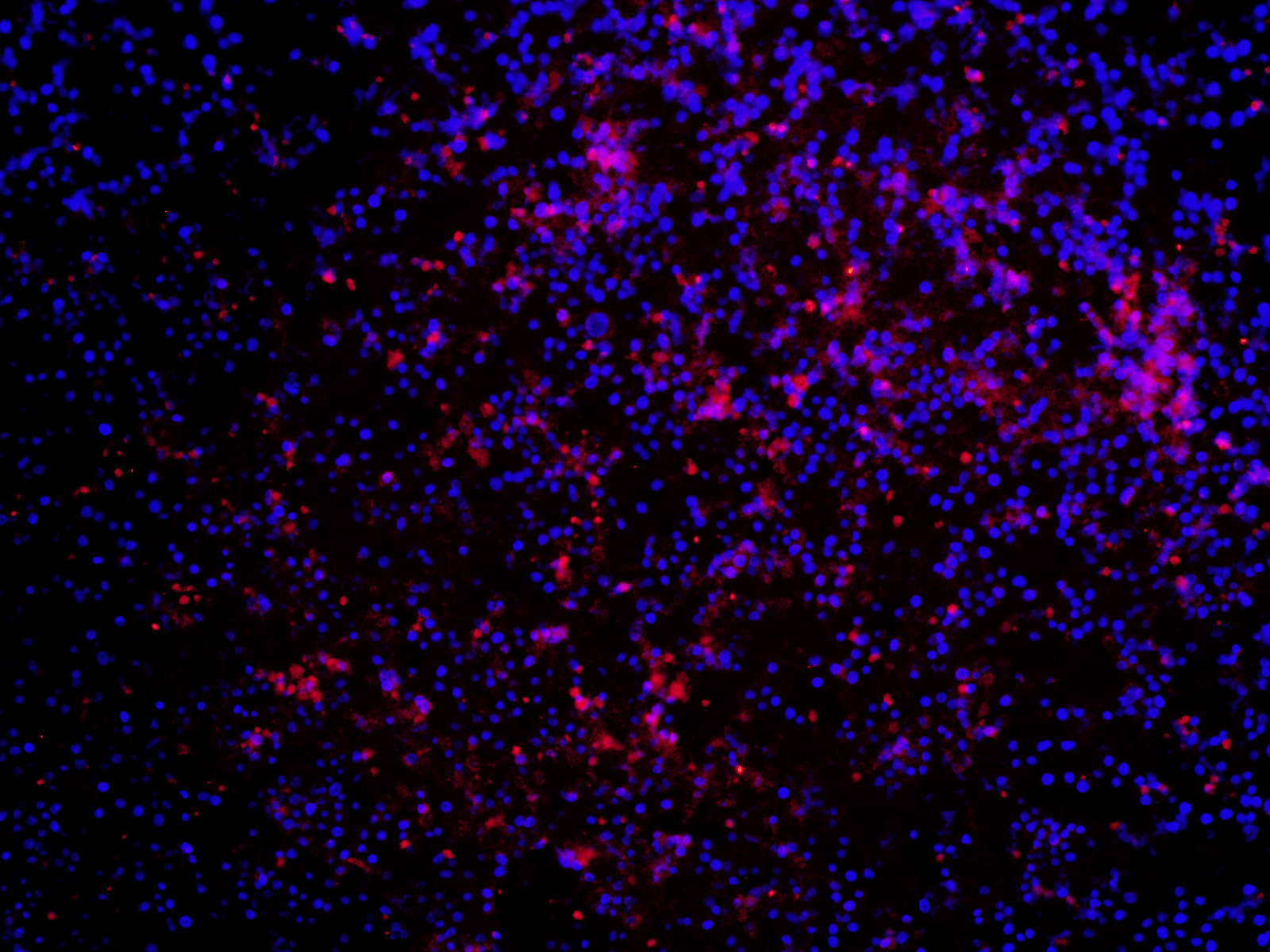

Supplement: S5 Data — This compressed folder contains the underlying numerical data and/or uncropped images used to generate the panels in Figs 6 and S1–S6, and S11. (ZIP) [file pbio.3003736.s019.zip › S5 Data/Supporting Information/Supporting Information fig4/A.PLVX-MCHERRY/apn-ko/5.png]

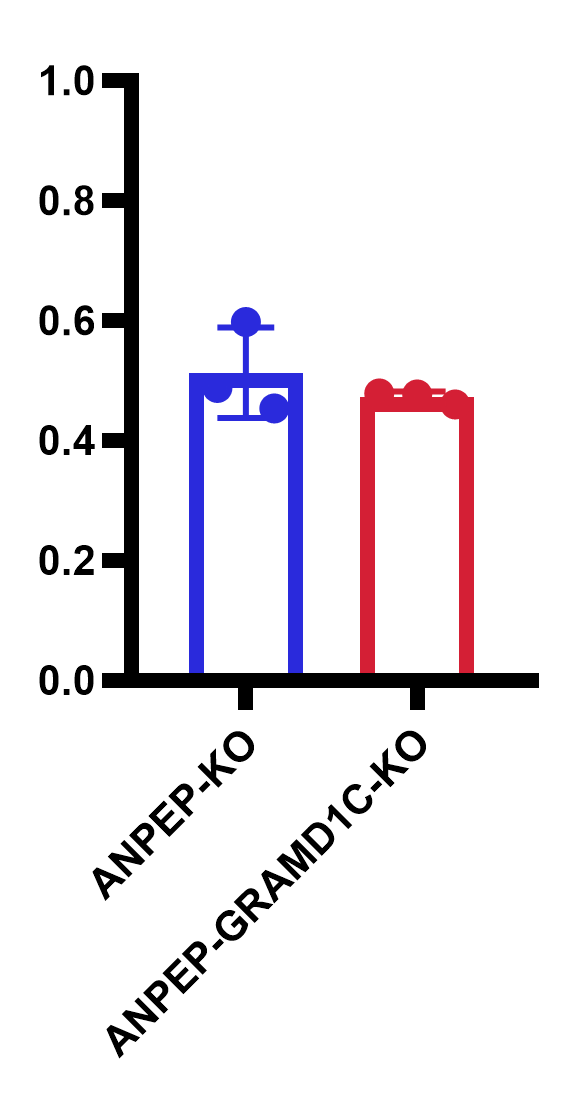

Supplement: S5 Data — This compressed folder contains the underlying numerical data and/or uncropped images used to generate the panels in Figs 6 and S1–S6, and S11. (ZIP) [file pbio.3003736.s019.zip › S5 Data/Supporting Information/Supporting Information fig4/A.PLVX-MCHERRY/transfection efficiency.tif]

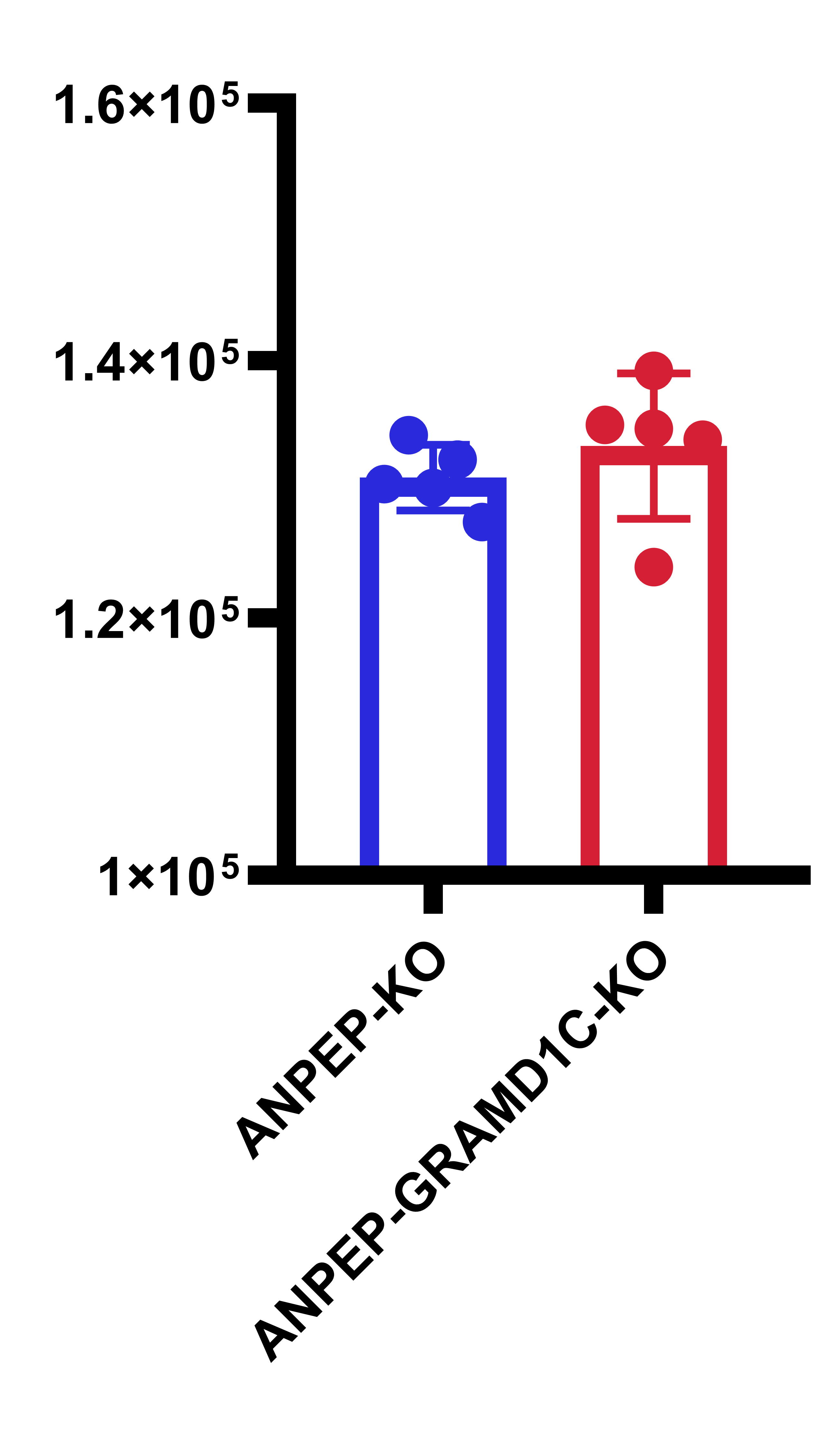

Supplement: S5 Data — This compressed folder contains the underlying numerical data and/or uncropped images used to generate the panels in Figs 6 and S1–S6, and S11. (ZIP) [file pbio.3003736.s019.zip › S5 Data/Supporting Information/Supporting Information fig4/B.RL-TK/RLU-transfection efficiency.tif]

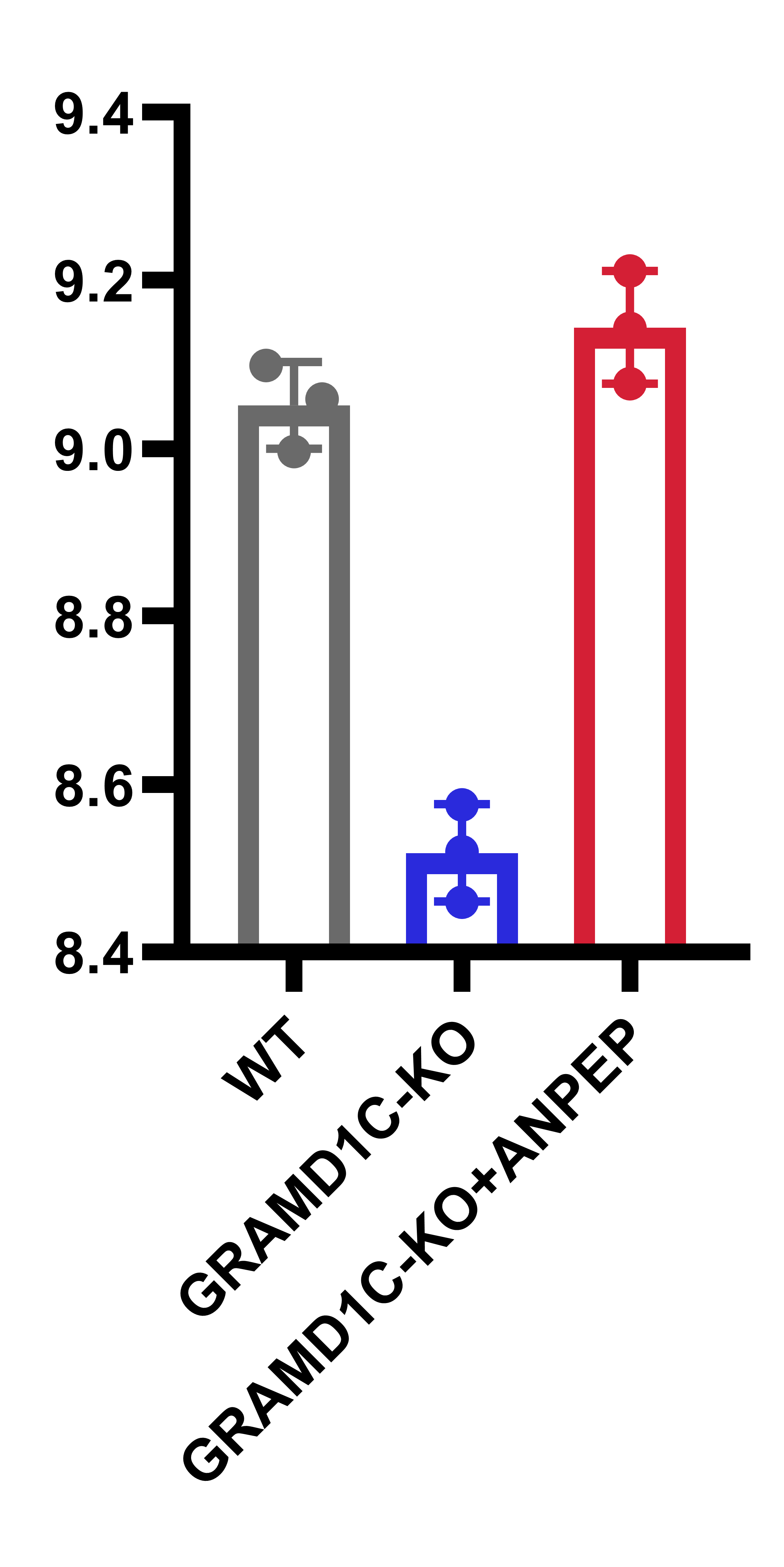

Supplement: S5 Data — This compressed folder contains the underlying numerical data and/or uncropped images used to generate the panels in Figs 6 and S1–S6, and S11. (ZIP) [file pbio.3003736.s019.zip › S5 Data/Supporting Information/Supporting Information fig5/A.attachment/attachment.tif]

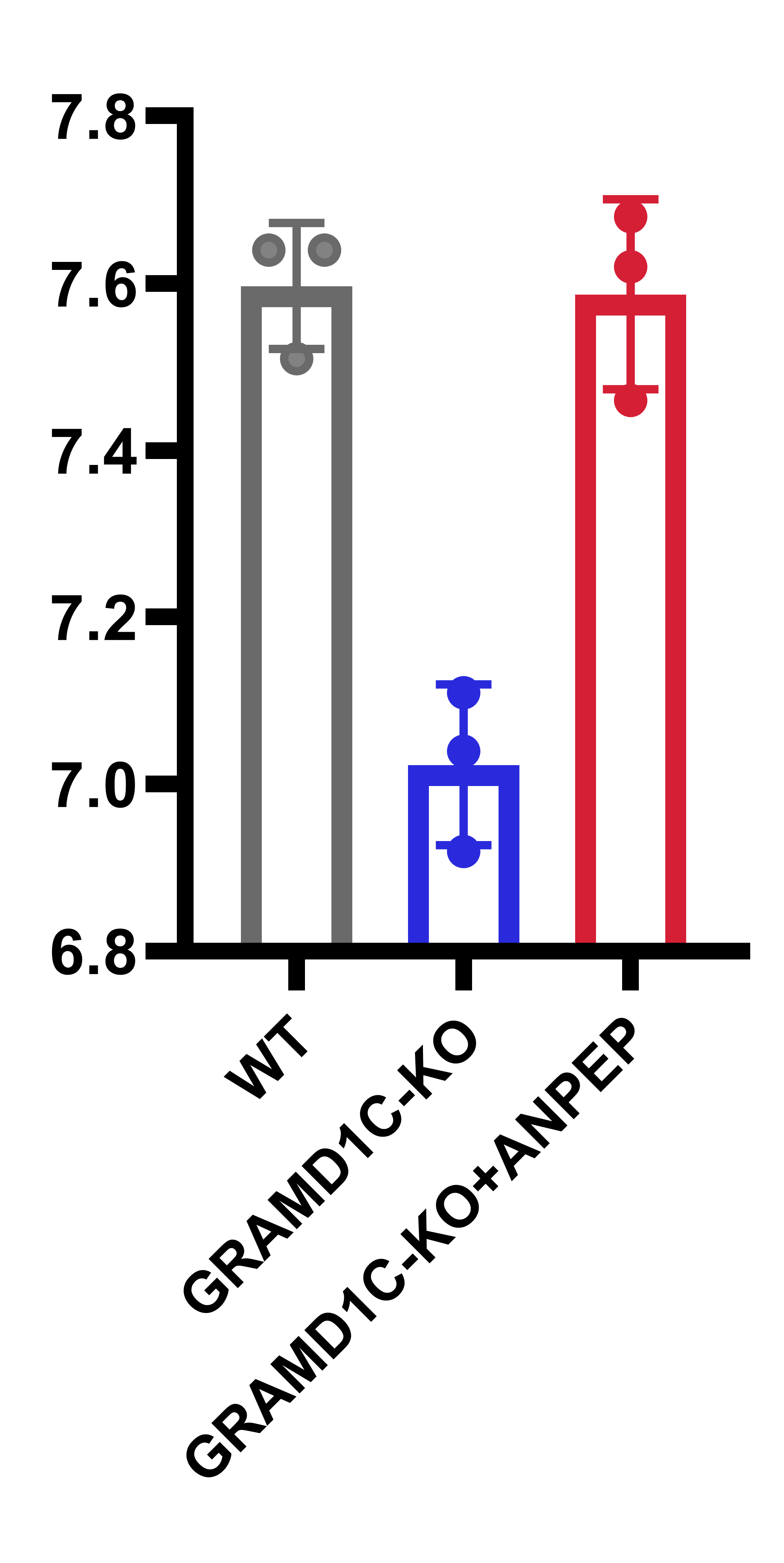

Supplement: S5 Data — This compressed folder contains the underlying numerical data and/or uncropped images used to generate the panels in Figs 6 and S1–S6, and S11. (ZIP) [file pbio.3003736.s019.zip › S5 Data/Supporting Information/Supporting Information fig5/B.endocytosis/endocytosis.tif]

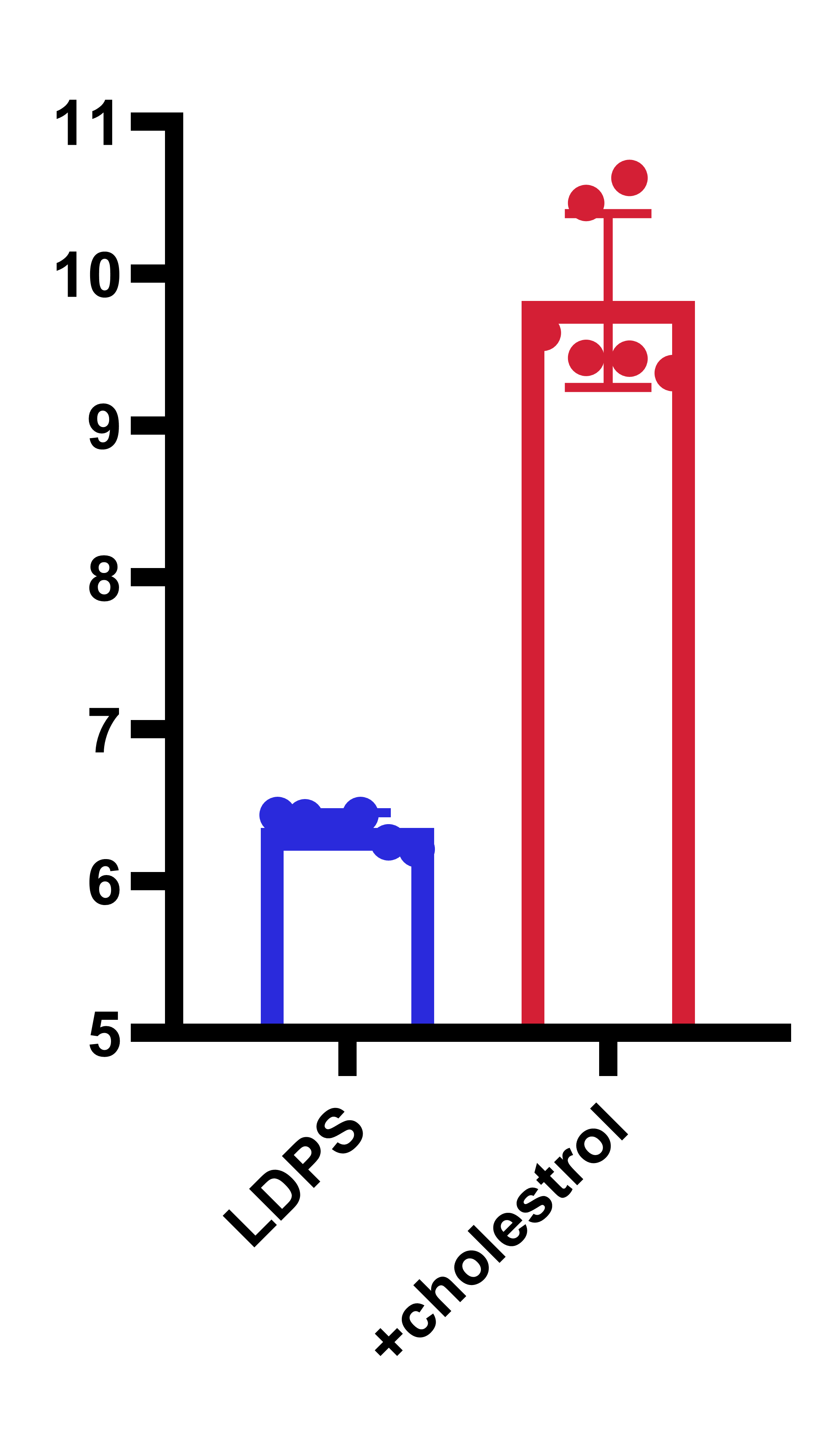

Supplement: S5 Data — This compressed folder contains the underlying numerical data and/or uncropped images used to generate the panels in Figs 6 and S1–S6, and S11. (ZIP) [file pbio.3003736.s019.zip › S5 Data/Supporting Information/Supporting Information fig6/A/293T+CHO.tif]
