# Supplementary material for: The non-vesicular cholesterol transporter GRAMD1C is a pan-coronavirus antiviral target
Source: PLoS Biol. 2026 Apr 6;24(4):e3003736. doi: 10.1371/journal.pbio.3003736 (PMC13068348; doi:10.1371/journal.pbio.3003736)
Supplement: S1 Raw Images — This PDF file contains the original scans of all blots and gels presented in the main figures and supplementary information. Relevant bands or areas are clearly marked where applicable. (PDF) [file pbio.3003736.s020.pdf]

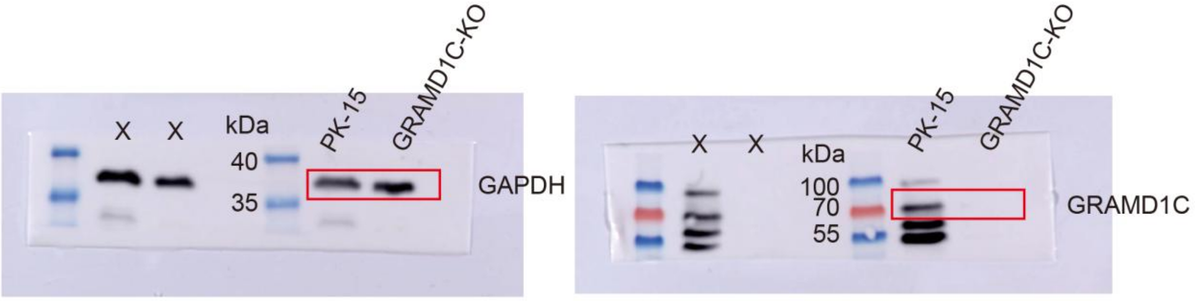

Amersham Imager 600(Cytiva), 1s exp.
Fig 1B in the final figure was generated from this image.

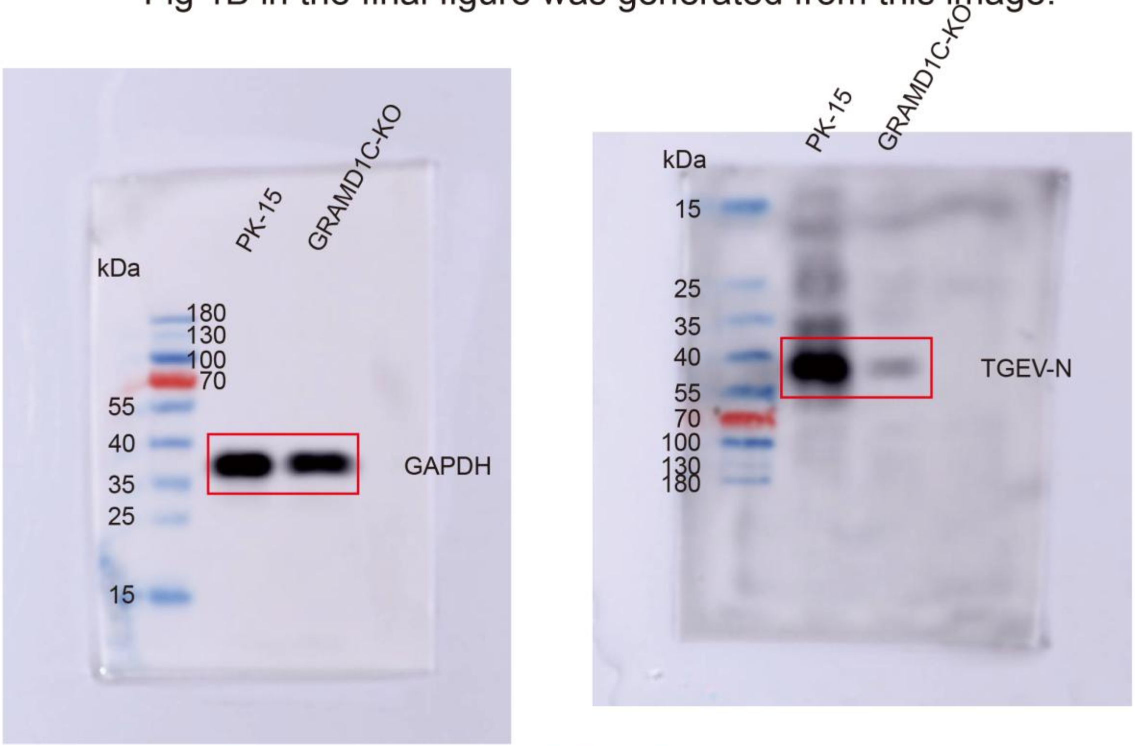

Amersham Imager 600(Cytiva), 1s exp.
Fig 1C in the final figure was generated from this image.

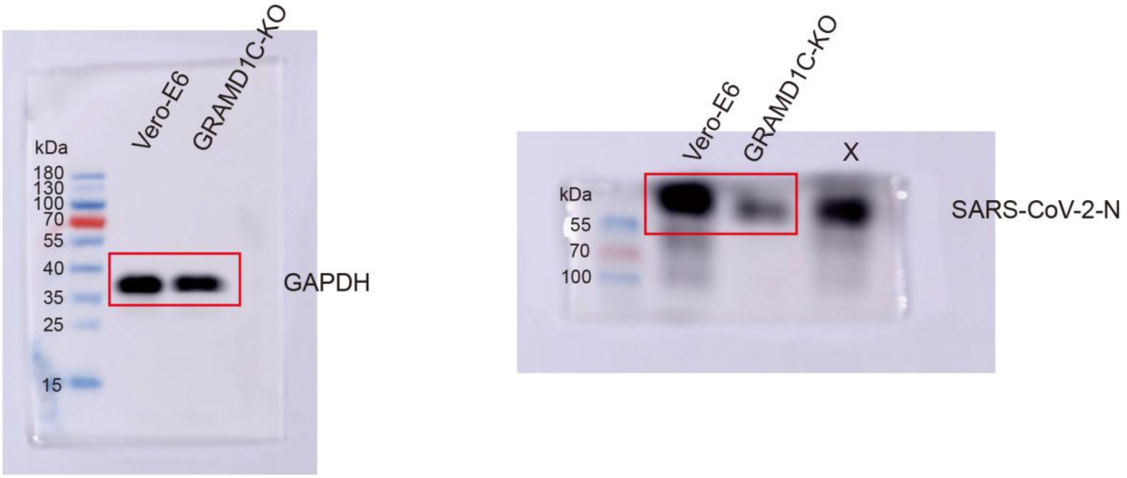

Amersham Imager 600(Cytiva), 1s exp.
Fig 1J in the final figure was generated from this image.

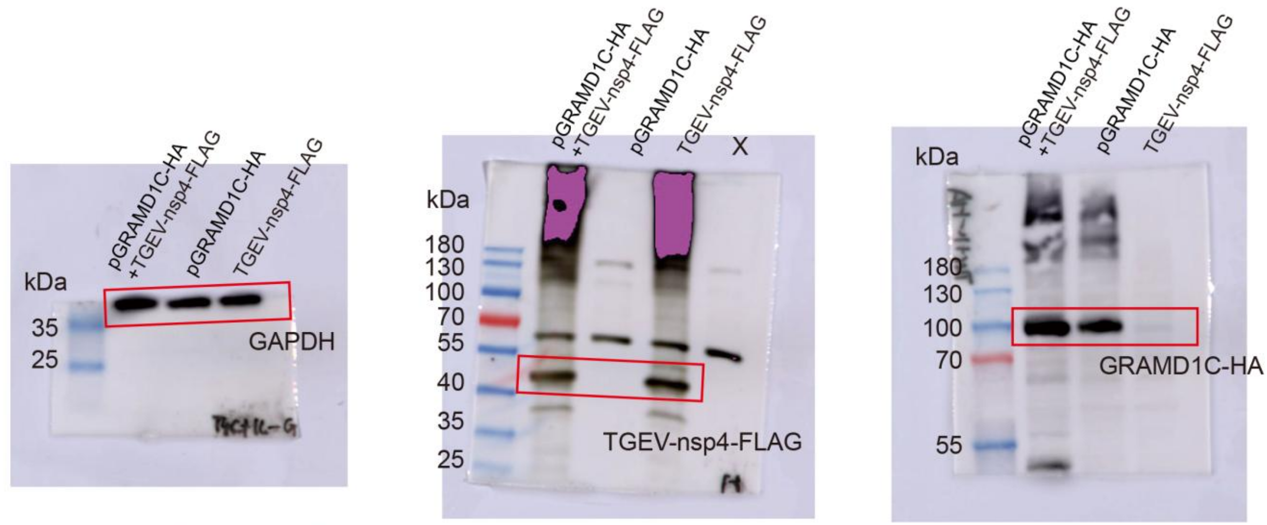

Amersham Imager 600(Cytiva), 1s exp.

Amersham Imager 600(Cytiva), 1s exp.

Amersham Imager 600(Cytiva), 3s exp.

Fig 3B (WCL group) in the final figure was generated from this image.

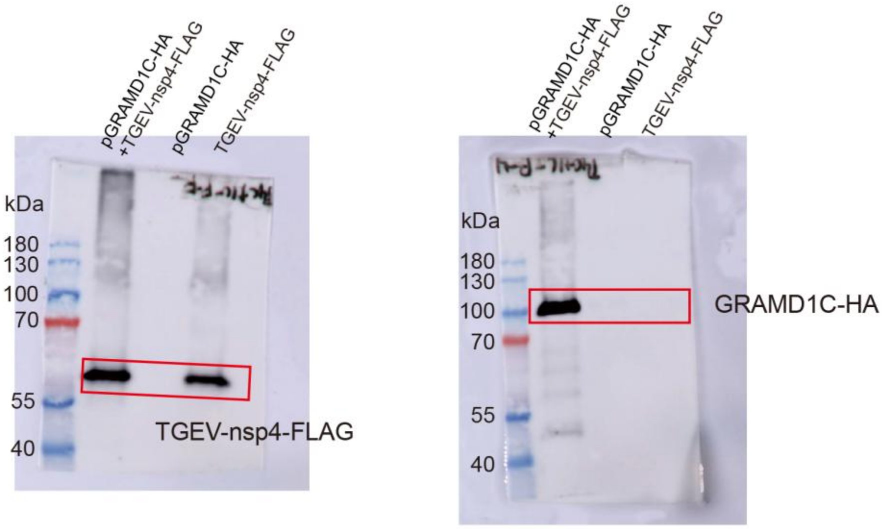

Amersham Imager 600(Cytiva), 1s exp.

Fig 3B (IP group) in the final figure was generated from this image.

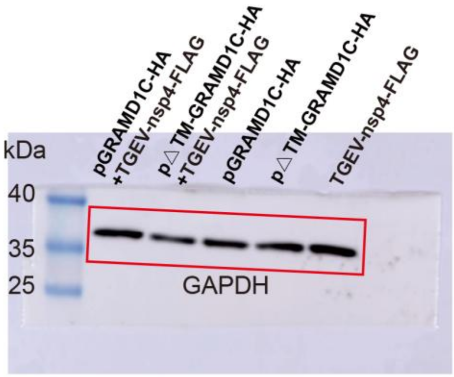

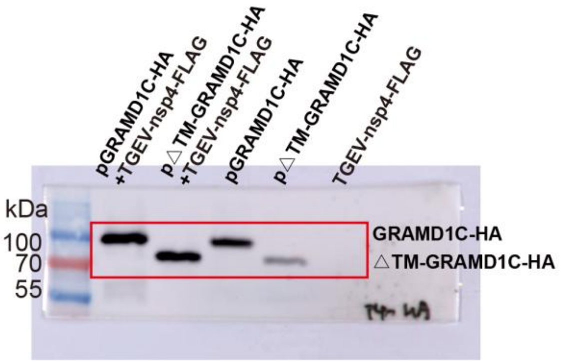

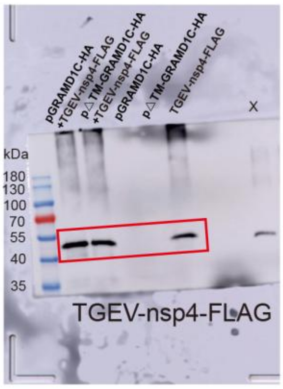

Amersham Imager 600(Cytiva), 1s exp.

Fig 3E (WCL group) in the final figure was generated from this image.

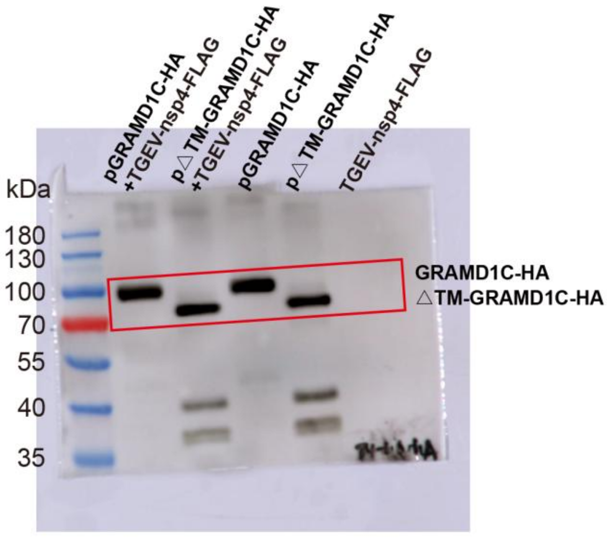

Amersham Imager 600(Cytiva), 1s exp.

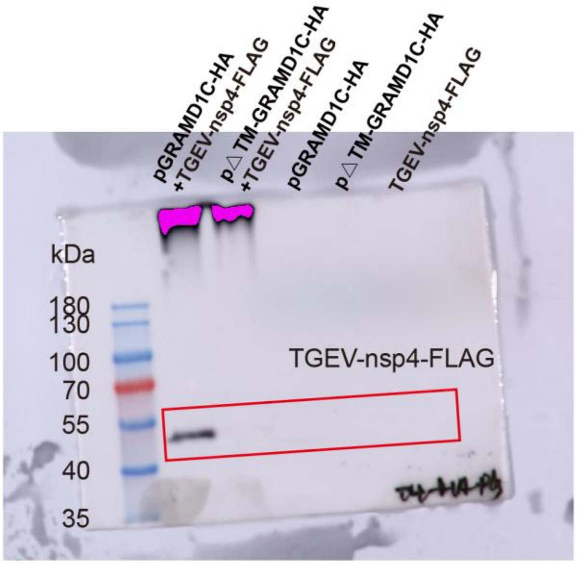

Amersham Imager 600(Cytiva), 3s exp.

Fig 3E (IP group) in the final figure was generated from this image.

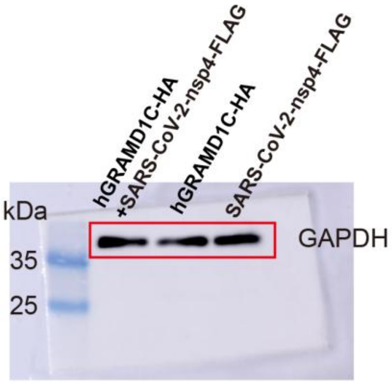

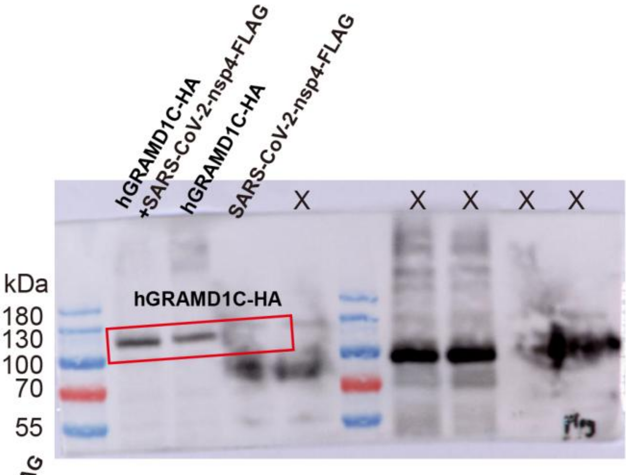

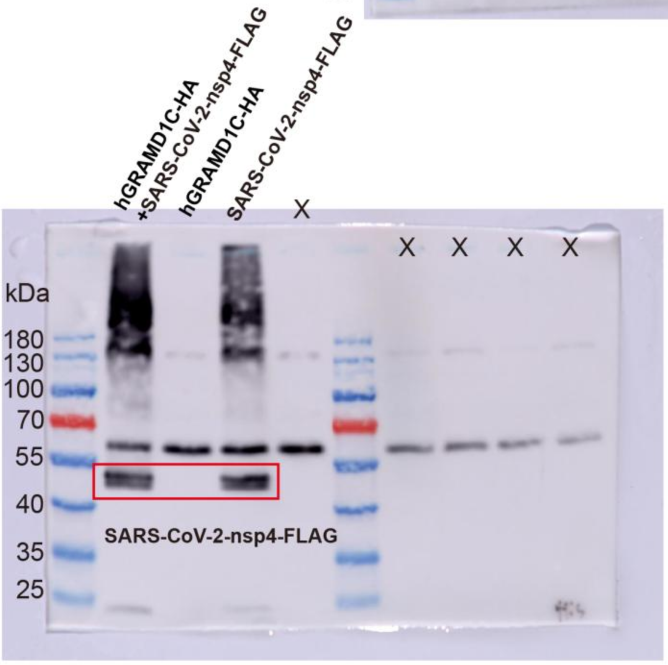

Amersham Imager 600(Cytiva), 1s exp.

Fig 3F (WCL group) in the final figure was generated from this image.

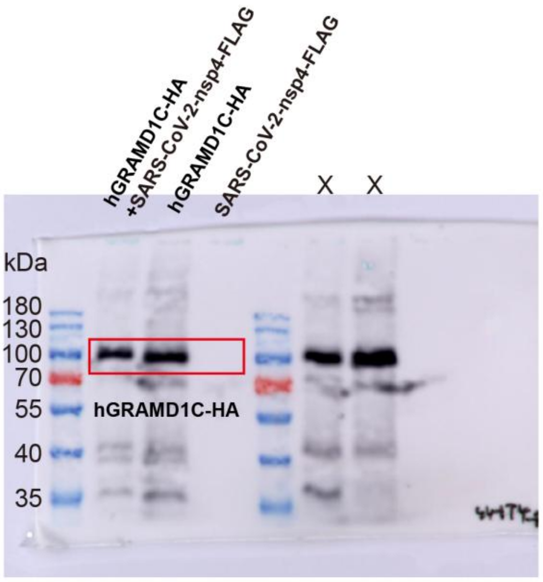

Amersham Imager 600(Cytiva), 1s exp.

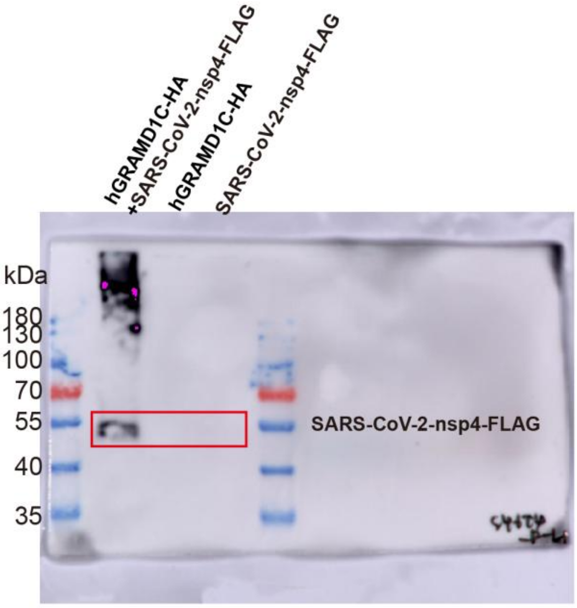

Amersham Imager 600(Cytiva), 4s exp.

Fig 3F (IP group) in the final figure was generated from this image.

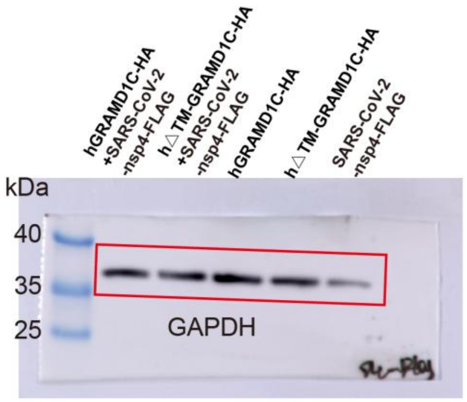

Amersham Imager 600(Cytiva), 1s exp.

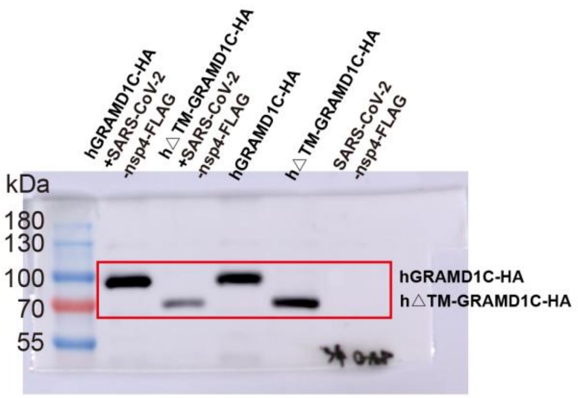

Amersham Imager 600(Cytiva), 1s exp.

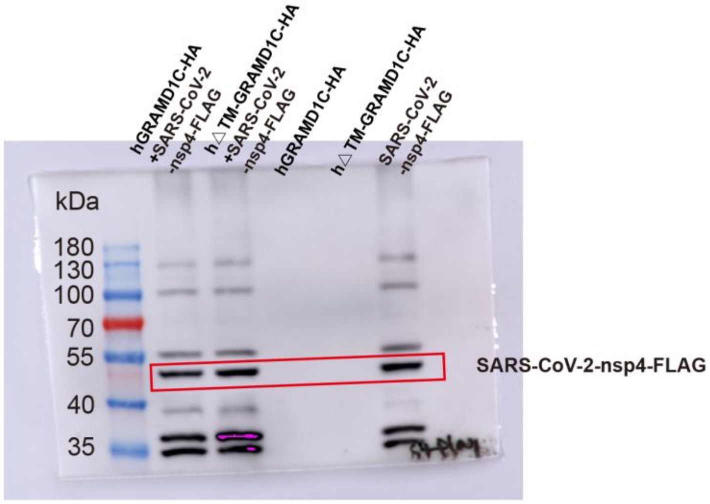

Amersham Imager 600(Cytiva), 2s exp.

Fig 3H (WCL group) in the final figure was generated from this image.

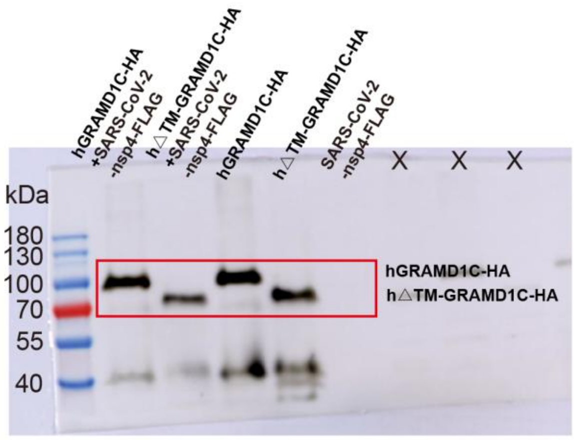

Amersham Imager 600(Cytiva), 2s exp.

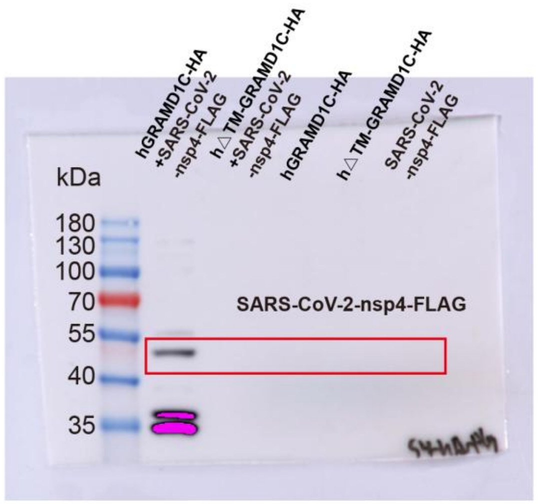

Amersham Imager 600(Cytiva), 4s exp.

Fig 3H (IP group) in the final figure was generated from this image.

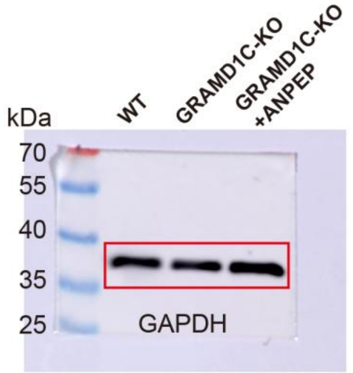

Amersham Imager 600(Cytiva), 1s exp.

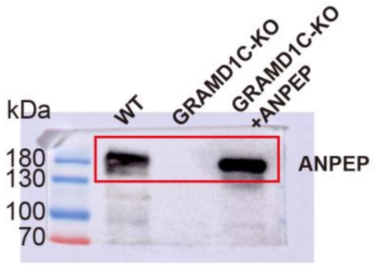

Amersham Imager 600(Cytiva), 4s exp.

S2A Fig in the final figure was generated from this image.

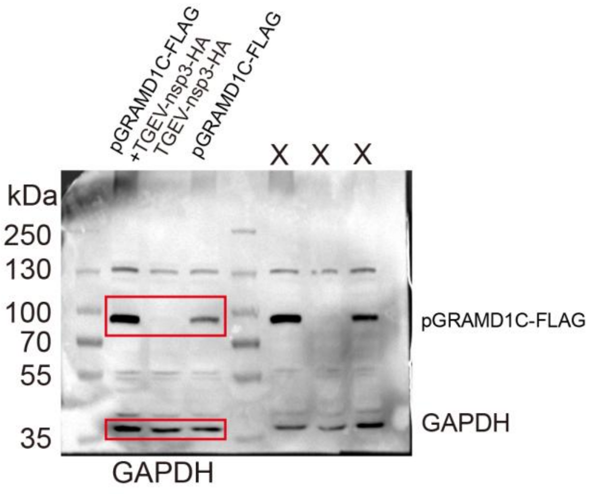

Tanon-5200,3s exp

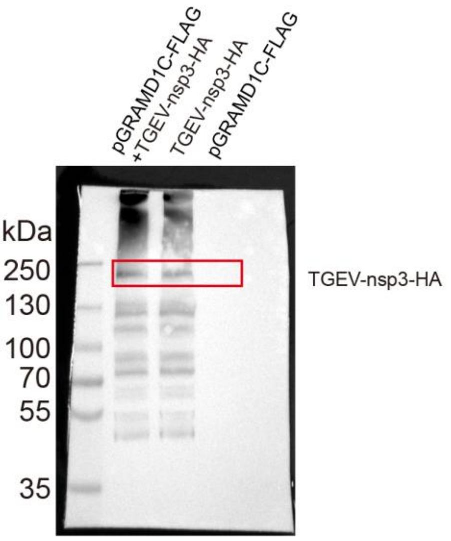

Tanon-5200,10s exp

S7A Fig (WCL group) in the final figure was generated from this image.

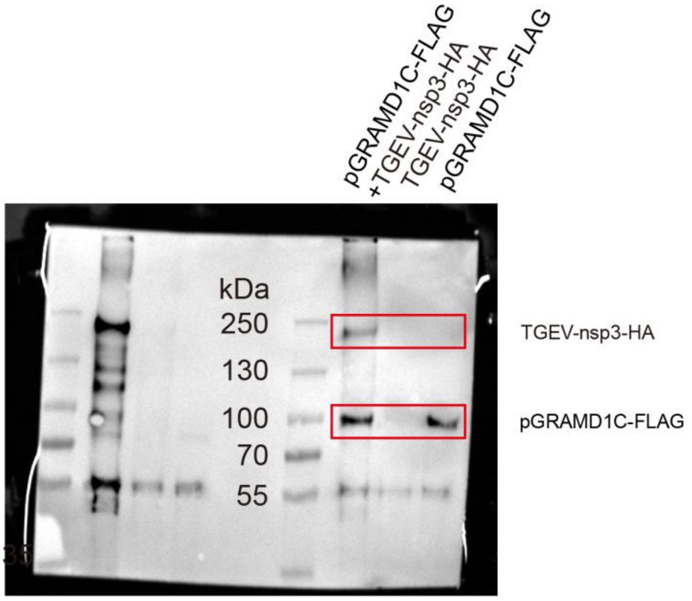

Tanon-5200,10s exp

S7A Fig (IP group) in the final figure was generated from this image.

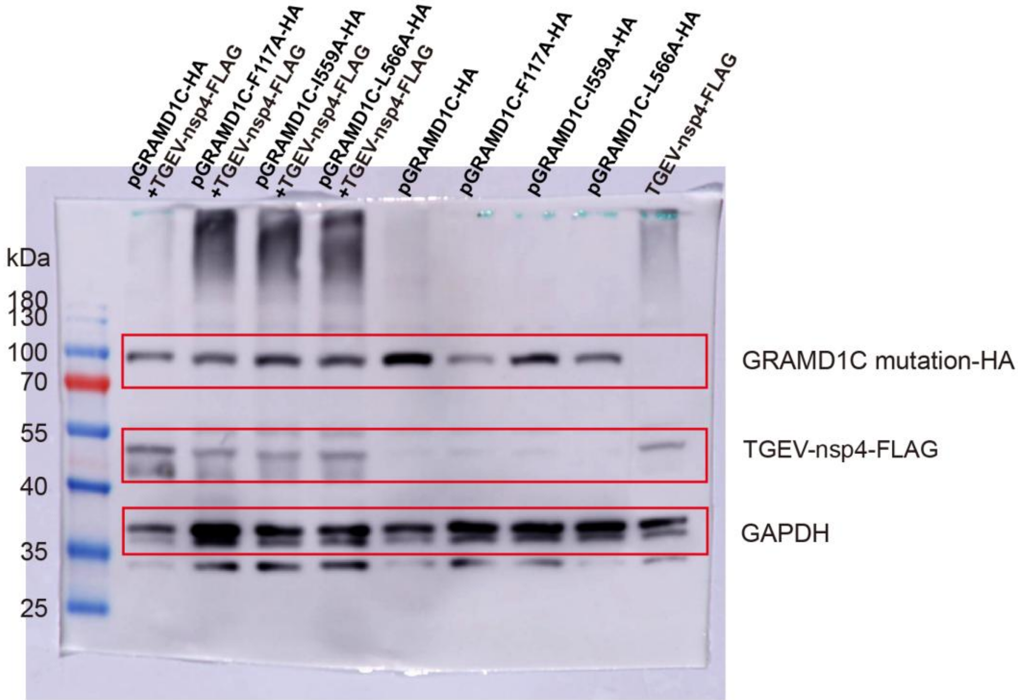

Amersham Imager 600(Cytiva), 4s exp.

S7B Fig (WCL group) in the final figure was generated from this image.

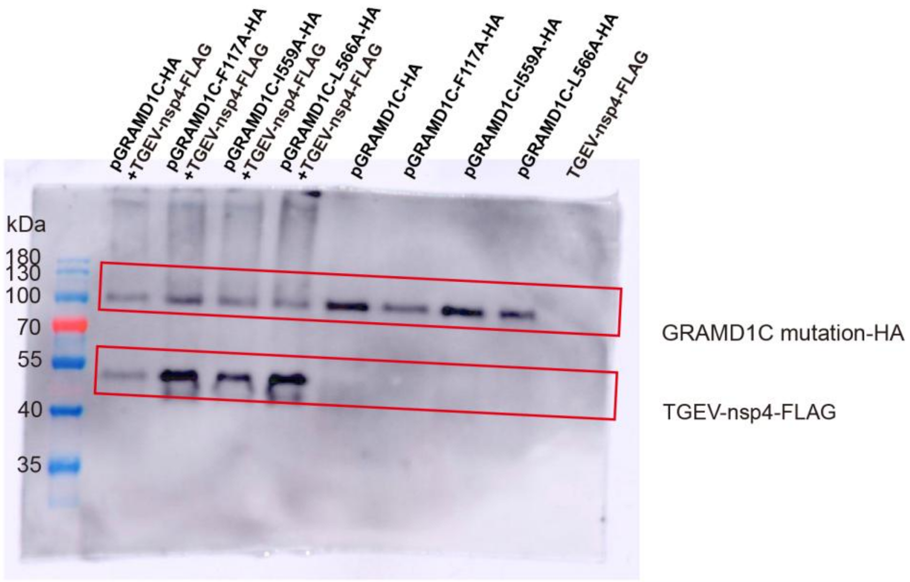

Amersham Imager 600(Cytiva), 4s exp.

S7B Fig (IP group) in the final figure was generated from this image.

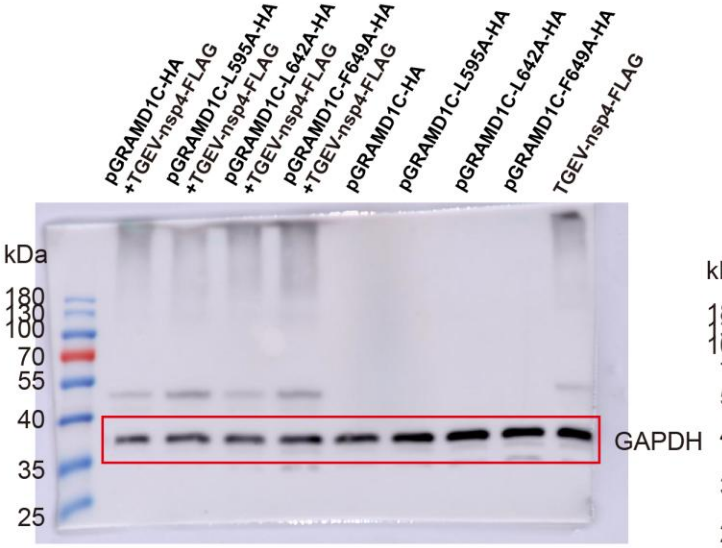

Amersham Imager 600(Cytiva), 1s exp.

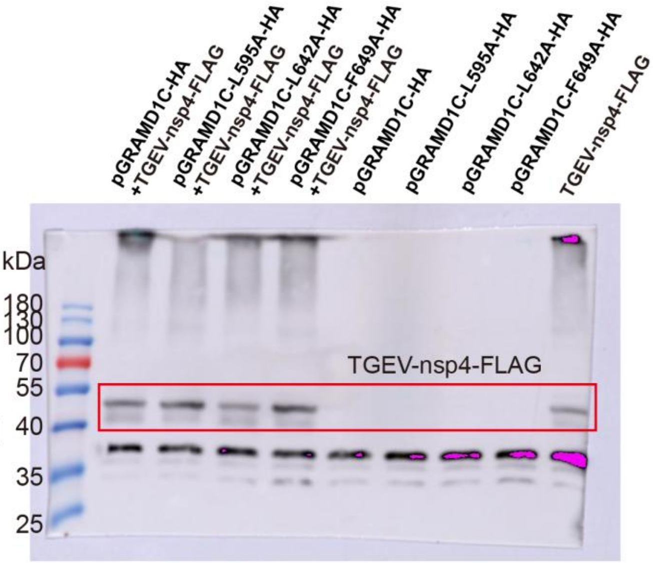

Amersham Imager 600(Cytiva), 4s exp.

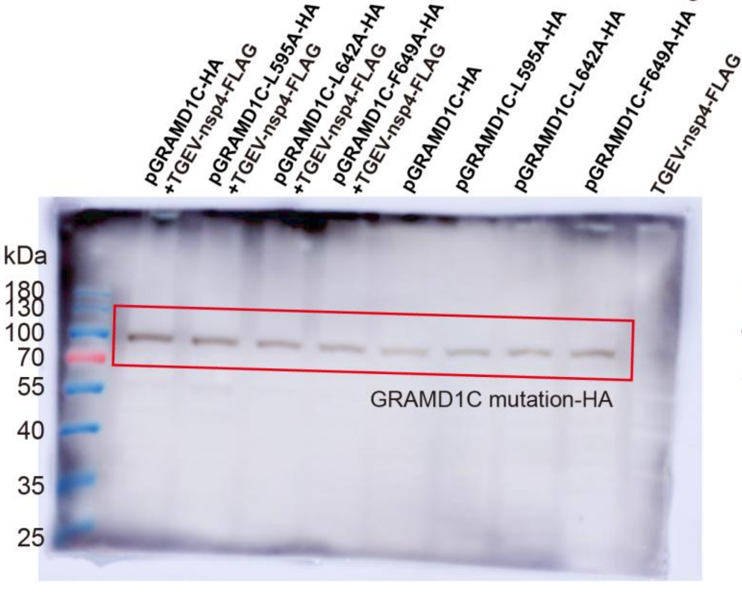

S7C Fig (WCL group) in the final figure was generated from this image.

Amersham Imager 600(Cytiva), 1min 30s exp.

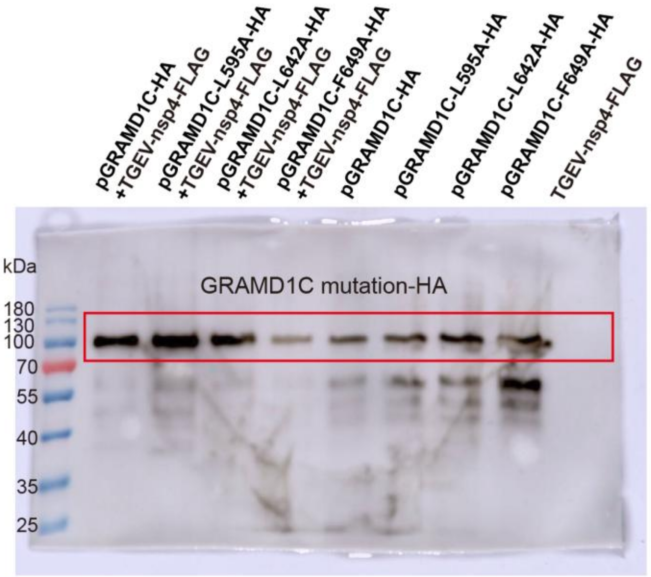

Amersham Imager 600(Cytiva), 1s exp.

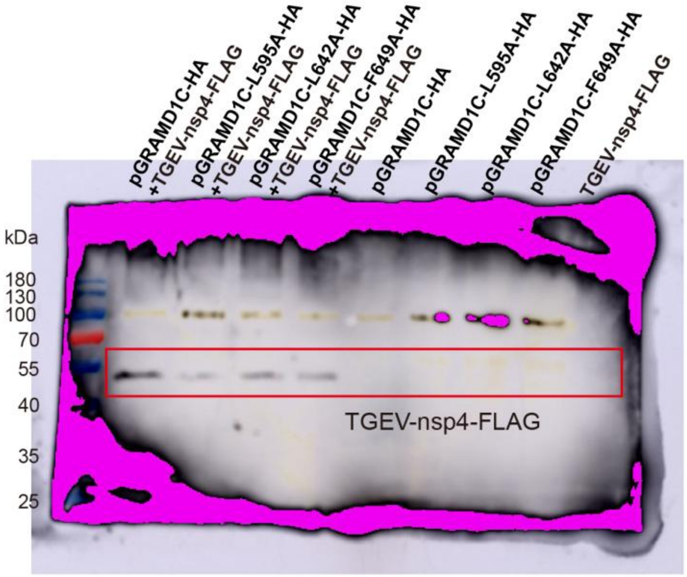

Amersham Imager 600(Cytiva), 1min exp.

S7C Fig (IP group) in the final figure was generated from this image.

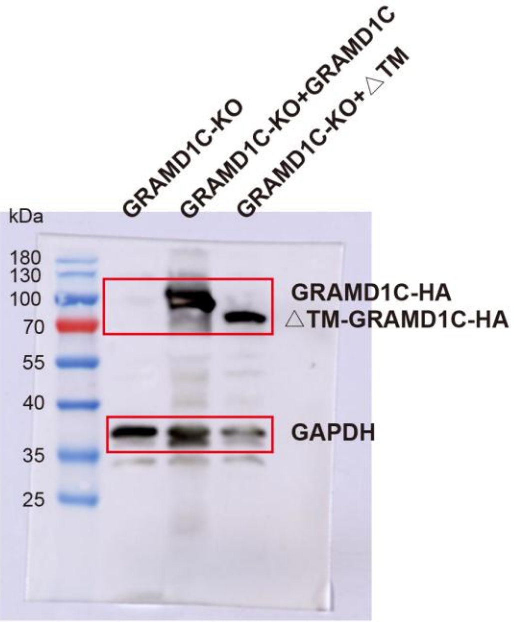

Amersham Imager 600(Cytiva), 1s exp.

S8 Fig in the final figure was generated from this image.

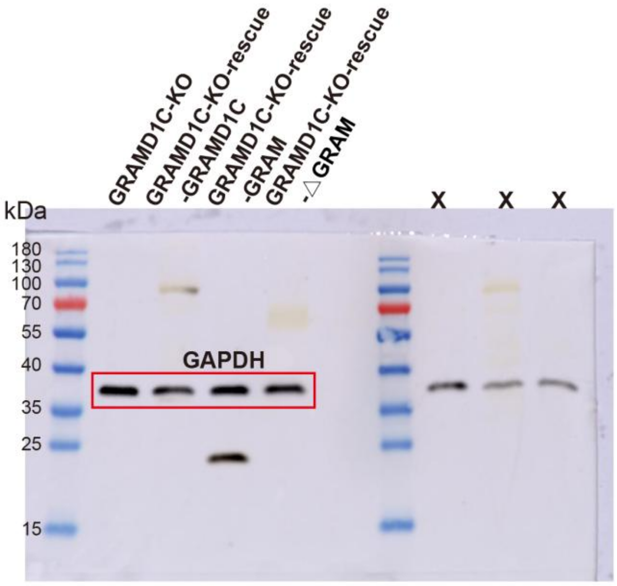

Amersham Imager 600(Cytiva), 1s exp.

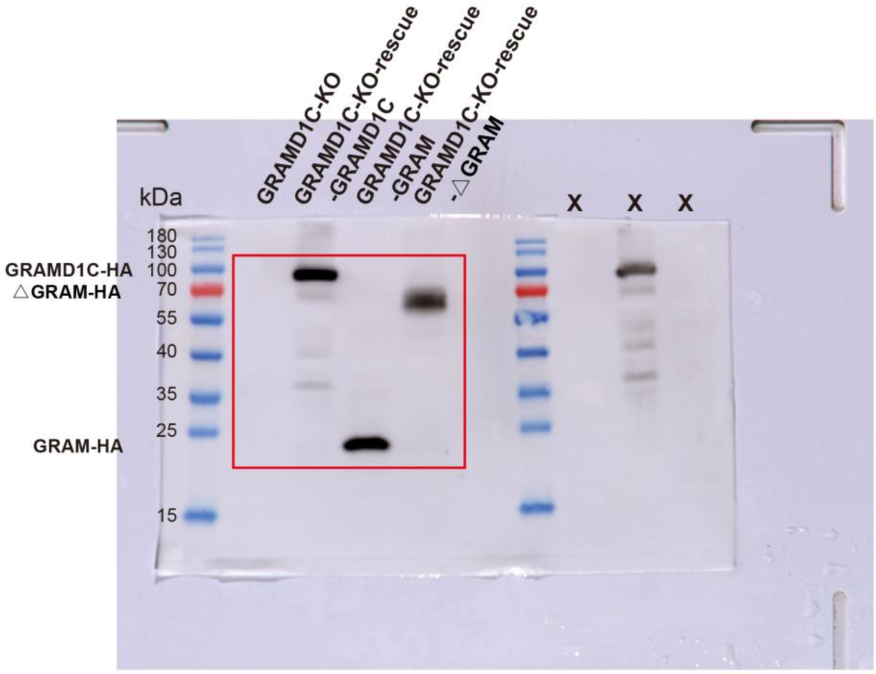

Amersham Imager 600(Cytiva), 1s exp.

S10 Fig in the final figure was generated from this image.

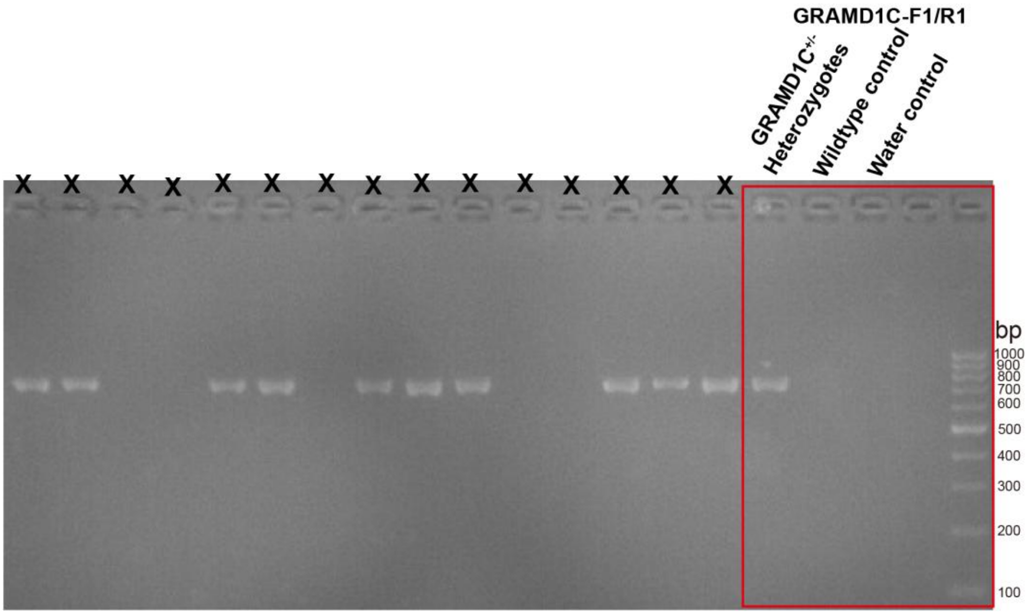

S12 Fig in the final figure was generated from this image.

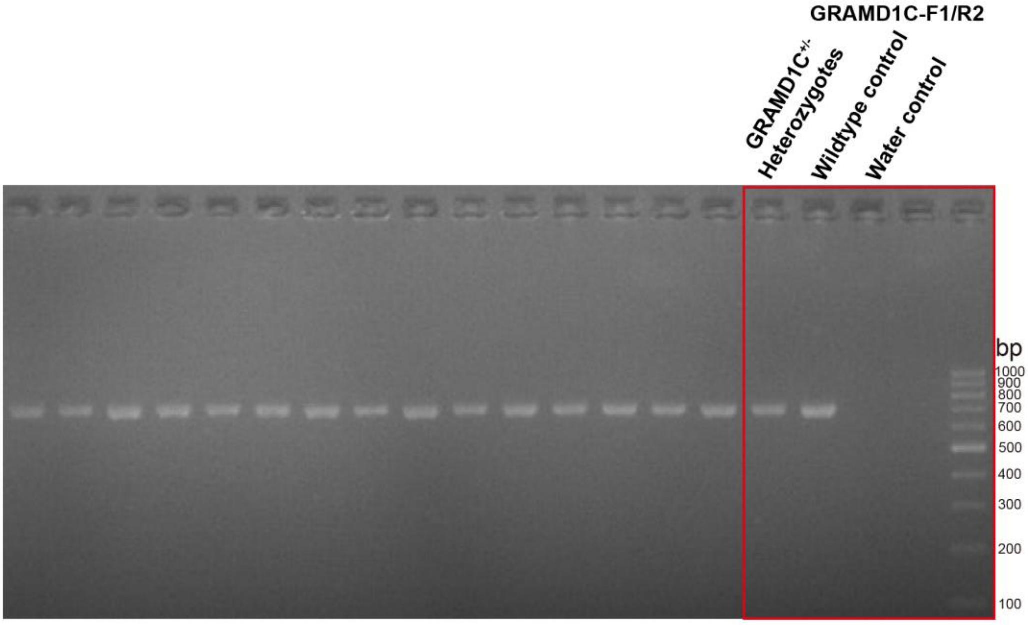

S12 Fig in the final figure was generated from this image.
