# Supplementary material for: The non-vesicular cholesterol transporter GRAMD1C is a pan-coronavirus antiviral target
Source: PLoS Biol. 2026 Apr 6;24(4):e3003736. doi: 10.1371/journal.pbio.3003736 (PMC13068348; doi:10.1371/journal.pbio.3003736)
Supplement: Attachment — Submitted filename: response to reviewers.docx [file pbio.3003736.s021.docx]

**Response to reviewers**

# Reviewer #1 (Remarks to the Author):

COMMENT:

Su and colleagues provide evidence that the cholesterol transport protein GRAMD1C is essential for coronavirus replication, including SARS-CoV-2. They used animal coronaviruses as models to show that GRAMD1C facilitates double-membrane vesicle formation through interaction with nsp4 and propose that GRAMD1C is a suitable targetable host protein that's required for the development of antivirals. This paper is informative and will be of interest to the wider coronavirus research community.

RESPONSE:

Thank you for your valuable feedback. In the following sections, you will find our responses to each of your points and suggestions.

## Major points (1):

Although GRAMD1C depletion markedly reduces TGEV, PEDV, and PDCoV replication, its impact on SARS-CoV-2 is minimal (from ~6 × 10¹² to ~2 × 10¹² copies/mL). How does this translate into changes in viral titers? What is the effect on endemic human coronaviruses such as HCoV-229E, particularly in light of the pronounced reduction of ANPEP? Considering the distinct tropisms of human versus animal coronaviruses, it would also be valuable to assess these effects in human lung cell lines to better reflect physiological relevance.

RESPONSE:

Thank you for raising this point. Based on the cells available in our laboratory, we performed viral titer assays and IFA for SARS-CoV-2 and HCoV-229E in GRAMD1C-KO Vero-E6 cells and GRAMD1C-KO Caco-2 cells, respectively (Fig. 1I and J, revised version). The results revealed that the absence of GRAMD1C inhibits viral proliferation.

A related description of these results has been added to the revised manuscript at lines 120-126, as follows: “*For other alphacoronaviruses such as HCoV-229E, we conducted IFA and viral titer assays in Caco-2 cells (Figs 1I, S1E and S1F). The results indicated that GRAMD1C knockout significantly suppressed the replication of HCoV-229E. As for betacoronaviruses, we investigated the replication of the original SARS-CoV-2 strain in GRAMD1C-KO Vero-E6 cells. IFA, viral titer assays, and western blot analysis all revealed a marked decrease in viral replication compared to WT cells (Fig 1J).*”

Figure 1

## Major points (2):

GRAMD1C KO markedly reduced ANPEP expression, the receptor for TGEV and PDCoV, suggesting that disruption of viral entry contributes to the observed decrease in viral titers. Consistently, restoring ANPEP expression in GRAMD1C KO cells diminished the antiviral effect (i.e., inhibition was significantly weaker upon ANPEP reconstitution). This strongly supports a central role of entry inhibition in the antiviral function of GRAMD1C. By contrast, the authors claim that GRAMD1C impacts intracellular viral replication independently of viral entry. However, in the double KO (ANPEP and GRAMD1C), only a minor reduction in viral replication was observed compared with the single ANPEP KO, further underscoring the importance of entry inhibition as a key pathway. While there is an effect on DMVs formation, as demonstrated by interaction and Co-IP studies this reviewer is doubtful that this is the major antiviral mechanism. The authors might want to carefully rephrase their conclusions, considering viral entry as additional major mechanism.

RESPONSE:

Thank you for raising this important point. To address the concern regarding the modest phenotype observed in the initial TGEV-BAC transfection (where only one round of amplification occurred), we repeated the experiment with increased plasmid amounts and extended the transfection time to 60 hours. Under these optimized conditions, a more pronounced reduction in viral replication in the double KO (ANPEP and GRAMD1C) compared to the single ANPEP KO was observed (Fig. 2B, revised version).

We agree with the reviewer on the important role of ANPEP in viral entry. However, through a series of ANPEP reconstitution experiments, we found that the antiviral phenotype in GRAMD1C-KO cells remains significant. We performed TGEV-BAC transfection in WT cells and in GRAMD1C-KO cells with ANPEP reconstituted to WT levels. The presence of ANPEP allowed released viral particles to re-enter cells, which further amplified the difference between the groups (Fig. 2C, revised version). Furthermore, we conducted qPCR, multi-timepoint viral titer assays, and IFA on the ANPEP-reconstituted cells (Fig. 2D-F, revised version). The results indicate that even after ANPEP reconstitution, GRAMD1C-KO still exhibits a very significant antiviral phenotype comparable to that in GRAMD1C-KO cells. Finally, we transfected TGEV-nsp3/4 plasmids into WT and GRAMD1C-KO cells and observed a significant reduction in the number of DMVs in the KO cells. This provides direct evidence for the impact of GRAMD1C on DMVs formation during the viral replication stage (Fig. 2I, revised version). Based on the above results, we believe that GRAMD1C primarily functions by inhibiting the replication stage.

A related description of these results has been added to the revised manuscript at lines 149-161，as follows:

*RT-qPCR assays showed that viral replication was further reduced in ANPEP-GRAMD1C-KO cells compared to ANPEP-KO cells (Fig 2B), confirming that GRAMD1C affects intracellular viral replication independently of viral entry.*

*We also reconstituted ANPEP expression in the GRAMD1C-KO PK-15 cells (GRAMD1C-KO+ANPEP) (S2A Fig). Attachment and internalization assays confirmed that viral entry was restored to WT levels upon ANPEP complementation (S5A and S5B Fig). Under these conditions, TGEV-BAC transfection in WT and GRAMD1C-KO+ANPEP cells revealed that GRAMD1C deficiency still significantly impaired viral replication (Fig 2C). Consistent with this, infection of GRAMD1C-KO+ANPEP cells with live virus, followed by RT-qPCR, multi-timepoint viral titer assays, and IFA, showed that the antiviral phenotype persisted and was comparable to that in GRAMD1C-KO cells (Fig 2D-2F).*

We also have added a discussion on the impact of GRAMD1C on viral entry in the Discussion section at lines 386-392, as follows: “*When investigating which stage of TGEV proliferation is affected by GRAMD1C, we found that GRAMD1C not only influences viral replication but also its entry. Specifically, the loss of GRAMD1C downregulated the expression of ANPEP, the receptor for TGEV. In contrast, GRAMD1C deficiency had a minimal effect on the expression of other viral receptors, such as ACE2 and CEACAM1. We speculate that the specific downregulation of ANPEP may result from alterations in signaling pathways (e.g., MAPK) induced by the loss of GRAMD1C.*”

Figure 2

## Major points (3):

Inhibitor treatment (20α-HC) strongly reduced MHV titers (much more compared to GRAMD1C KO) while the effect on TGEV stayed in a similar range to the previous observations. How do the authors explain these discrepancies? Could there be additional targets/mechanisms? What is the effect of the inhibitor on SARS-CoV-2 using lung cell culture models?

RESPONSE:

Thank you for your insightful observation. We first examined whether the inhibitor reduced expression of the MHV receptor CEACAM1. Treatment with 30 μM 20α-HC for 24 hours did not alter CEACAM1 levels (Response Figure 1). This result suggests that the antiviral effect of 20α-HC is not mediated through receptor downregulation. Therefore, we speculate that the different cell lines used may have different sensitivity to the inhibitor. The cellular environment where MHV replicates could be inherently more vulnerable to 20α-HC.

Response Figure 1

Based on the cells available in our laboratory, we treated Vero-E6 cells with the inhibitor 20α-HC after SARS-CoV-2 infection. Results from IFA and viral titer assays showed that this inhibitor markedly suppressed viral replication (Fig. 5J, revised version).

A related description of these results has been added to the revised manuscript at lines 292-294，as follows: “*Similarly, in Vero-E6 cells treated with 30 µM 20α-HC (S10E Fig) and infected with SARS-CoV-2, a marked inhibition of viral replication was observed (Fig. 5J).*”

Figure 5

## Minor points (1):

The sentence in the discussion: "Our study provides compelling evidence that GRAMD1C, a non-vesicular cholesterol transport protein, is a critical host factor for the replication of multiple coronaviruses, including SARS-CoV-2, TGEV, PEDV, PDCoV, and MHV-A59.", is an overstatement given the discussion points above and needs rephrasing, specifically given the limitations (strong entry effects, minor effect on SARS-CoV-2 replication).

RESPONSE:

We thank the reviewer for this suggestion. Supported by additional experimental data, we have revised the discussion at lines 357-359, as follows: “*Our study identifies GRAMD1C, a non-vesicular cholesterol transport protein, as a crucial host factor for multiple coronaviruses, mediating DMV formation.*”

## Minor points (2):

Intro: nsp3 and nsp4 are indeed central, but evidence suggests nsp6 also contributes to membrane remodeling, so calling nsp3+nsp4 the minimal viral factors may oversimplify.

RESPONSE:

We thank the reviewer for this valuable clarification. To present a more precise and comprehensive description, we have revised the relevant section in the introduction (lines 64-68), as follows: “*During authentic infection, viral nonstructural proteins nsp3, nsp4, and nsp6 collectively contribute to DMV biogenesis [16-19]. Among these, co-expression of nsp3 and nsp4 has been established as the minimal requirement sufficient to induce DMV formation in vitro [20, 21], highlighting their central role in initiating membrane remodeling.*”

## Minor points (3):

How do the authors explain the faint GRAMD1C band in the KO condition in Fig. 1B? In other words: how stable/complete is the KO? Could these be residual wild-type cells?

RESPONSE:

Thank you for pointing out these issues. We apologize for the ambiguity. We have repeated the experiment using stable single-clone of GRAMD1C-KO cells after multiple passages. The new results confirm that GRAMD1C knockout is complete, as shown in Fig. 1B (revised version).

Figure 1

## Minor points (4):

Cell viability should be tested at multiple timepoints post-treatment (ideally up to 48 h).

RESPONSE:

We thank the reviewer for this suggestion. In response, we have performed additional MTS assays to measure cell viability of all relevant cell lines at 12, 24, 36 and 48 hours post-inhibitor treatment. These new data have been added to the revised manuscript (Fig. S10A and S10C-S10F, revised version).

Supplementary Figure 10

## Minor points (5):

How were numbers of DMVs per cell determined? Manually or computationally? How many cells were assessed? The experimental details should be provided. Same for the determination of the normalized mean intensity of IF images (see Figs. 2, 4, 5) and the quantification of EdU-pos. cells.

RESPONSE:

Thank you for these valuable suggestions regarding the method details.

For the quantification of DMVs per cell (Figs. 2H, 2I, 5G, 5I): The number of cells analyzed each group was 40, 20, 5, and 5 for Figs. 2H, 2I, 5G, and 5I, respectively. Within the boundary of each entire single-cell profile, all DMVs were manually counted.

The description regarding the counted cell numbers has been added into the corresponding figure legends, and the explanation of the DMVs quantification method has been added to the Methods section (lines 748-750), as follows: “*TEM images were quantified for DMV abundance. For each condition, multiple random cell profiles were analyzed. Within the boundary of each entire single-cell profile, all DMVs were manually counted.*”

For the normalized mean fluorescence intensity analysis (dsRNA signal in Figs. 2, 5), the related description of these details has been revised to the manuscript (lines 708-711), as follows: “*The mean fluorescence intensity was measured using ImageJ (National Institutes of Health, Bethesda, MD, USA) for both the DAPI and dsRNA channels within defined regions of interest. The ratio of the dsRNA channel intensity to the DAPI channel intensity was then calculated and used for comparative analysis.*”

Upon careful reevaluation, we think that Fig. 4D was not suitable for precise quantification of free cholesterol. Therefore, the intracellular free cholesterol content was measured using a Free Cholesterol Content Assay Kit (Solarbio, BC1895) according to the manufacturer’s instructions (Fig. 4E, revised version).

For the quantification of EdU-positive cells: The details have been added to the Methods section (lines 712-722), as follows: “*Quantitative analysis of the percentage of positive cells was performed using ImageJ software. For each region of interest (ROI), images were converted to 8-bit grayscale. A consistent threshold was manually set to distinguish positive signal from background, and this threshold was applied uniformly to all images within the same experimental set. The “Analyze Particles” function was then used to count positively stained cells. The total number of cells in the same ROI was determined by DAPI. The percentage of positive cells was calculated as (Number of positive cells / Total number of cells) × 100%. For each condition, at least three independent biological replicates were analyzed, with multiple ROIs quantified per replicate. Data are presented as mean ± standard deviation (SD). Statistical significance was determined using two-tailed unpaired Student’s t-tests, with a p-value < 0.05 considered significant.*”

Figure 4

## Minor points (6):

-What program was used for the protein-protein interaction modeling (Fig. 3C and G)? Include in M&Ms or Fig. legend.

RESPONSE:

Thank you for helping us improve the clarity of our manuscript. We have added the clarifications to the Methods section of the revised manuscript at lines 780-791, as follows: “*The proteins used for docking were porcine GRAMD1C(A0A8D1XGN7), human GRAMD1C(Q8IYS0), TGEV-nsp4(P0C6V2), SARS-CoV-2-nsp4 (P0C6X7). The HDOCK SERVER (http://hdock.phys.hust.edu.cn/) was employed for protein-protein molecular docking (54). The binding sites of the proteins are presented in Table S2-1. The MM/GBSA mode of the HawkDOCK SERVER (http://cadd.zju.edu.cn/hawkdock/) was utilized to calculate the free binding energy of protein-protein interactions and rank the free binding energy of amino acid residue sites (Table S2-2) (55). The docking score was adopted as the evaluation criterion for docking. Among the 10 conformations output by docking, the best docking model was selected and the binding energy was calculated. Finally, we used Pymol 2.4 software to visualize the binding sites with high binding energy and mutual binding. In this way, we could visually observe the binding situation between the proteins.*”

## Minor points (7):

Mock (WT) controls is missing in the data presented in Fig. 3I, Fig. 4F.

RESPONSE:

Thank you for bringing this issue to our attention. We have included WT controls in the corresponding experiments to better evaluate the reconstitution. These data are now presented in Fig. 3I and 4G (revised version).

Figure 3	Figure 4

## Minor points (8):

Fig. 6: histopathology and immunohistochemistry to assess viral antigen distribution would be an informative add-on!

RESPONSE:

We thank the reviewer for this insightful suggestion. Regarding histopathology, H&E staining results are already presented in Fig. 6E and 6M of the current manuscript. For immunohistochemistry (IHC), due to the lack of a validated antibody against MHV-A59, we performed IHC using an anti-dsRNA antibody. The results are presented in Fig. 6F and 6N (revised version).

The related description of these results has been revised to the manuscript (lines 323-326, 335-337), as follows:

*To assess viral antigen distribution, immunohistochemistry (IHC) with an anti-dsRNA antibody was performed. It revealed stronger positive signals in the liver tissues of WT mice compared to GRAMD1C^+/-^ mice (Fig 6F).*

*IHC results revealed that, compared with WT mice, the inhibitor-treated mice exhibited a more restricted distribution and weaker intensity of dsRNA signals (Fig. 6N).*

Figure 6

## Minor points (9):

-It would be great to confirm the mouse genotypes by PCR if the authors aimed for clearly separable band sizes (See Fig. S6) - or run a high-resolution agarose gel?

RESPONSE:

Thank you for your valuable suggestion. In our study, we determined the genotype of the mice by designing specific primer sets and assessing whether corresponding PCR bands were amplified. As shown in Fig. S11A (revised version), we designed two pairs of primers: F1/R1 and F1/R2. In WT mice, only the F1/R2 primer pair produces a visible band (692 bp) under the given PCR conditions. In heterozygous KO mice, both the F1/R1 and F1/R2 products are amplified, resulting in two distinct bands (Fig. S11C, revised version).

This approach allows clear and reliable discrimination among WT, and heterozygous knockout genotypes.

Supplementary Figure 11

## Minor points (10):

The whole manuscript should be proofread for grammar and typos.

RESPONSE:

We thank the reviewer for the suggestion. The manuscript has been thoroughly proofread and edited to correct grammatical errors and typos.

## Minor points (11):

Check all figure labels again for consistency in capitalization.

RESPONSE:

We thank the reviewer for pointing this out. We have checked and corrected the capitalization of labels in all figures as needed.

# Reviewer #2 (Remarks to the Author):

COMMENT:

In this study, Su et al. report the non-vesicular cholesterol transporter GRAM domain containing 1C (GRAMD1C) from the Aster protein family as an important host factor supporting the replication of different coronaviruses, i.e. porcine transmissible gastroenteritis virus (TGEV), porcine delta coronavirus (PDCoV), porcine epidemic diarrhea virus (PEDV), murine hepatitis virus (MHV) and, to a much lesser extent, severe acute respiratory syndrome virus 2 (SARS-CoV-2). These viruses share a similar strategy of replicating in ER-derived double membrane vesicles (DMVs). The authors provide compelling evidence that GRAMD1C depletion or pharmacological inhibition impairs viral RNA replication and appears to affect DMV production. Additionally, the authors validated the potent GRAMD1C inhibitor 20α-hydroxycholesterol (20α-HC) as a potential inhibitor impairing the replication of coronaviruses, at least porcine ones and MHV, in cell culture and the latter also in a mouse model. Moreover, a heterozygous deletion of the GRAMD1C gene in mice attenuated MHV replication and reduced pathogenicity in these animals.
Overall, the data are strong and they reveal an important role of the cholesterol transporter GRAMD1C in coronavirus replication, possibly by contributing to the formation of DMVs. A strength of this study is the use of different viruses and cell models and the demonstration of antiviral activity in vivo either by depletion of GRAMD1C expression in animals or inhibition of the enzyme. Nevertheless, I found a couple of instances where far-reaching statements have been made that are not supported by the data as well as a few technical flaws that should be fixed.

RESPONSE:

Thank you for your valuable feedback. In the following sections, you will find our responses to each of your points and suggestions.

## Major points (1):

The authors frequently switch between different viruses, most of them being porcine viruses. While the phenotype exerted by GRAMD1C interference is very strong on these viruses, and for MHV (around 100-fold reduction of viral replication), this is not the case with SARS-CoV-2 where the effect is just 3-fold (Fig. 1H). This is a major difference raising questions about physiological relevance for SARS-CoV-2. Such further evidence could be obtained by including a test of 20α-HC against SARS-CoV-2 that unfortunately is missing in this study, but needs to be done.

RESPONSE:

Thank you for raising this point. We performed viral titer assays and IFA for SARS-CoV-2 in GRAMD1C-KO Vero-E6 cells (Fig. 1J, revised version). The results revealed that the absence of GRAMD1C inhibits viral proliferation.

A related description of these results has been added to the revised manuscript at lines 123-126, as follows: “*As for betacoronaviruses, we investigated the replication of the original SARS-CoV-2 strain in GRAMD1C-KO Vero-E6 cells. IFA, viral titer assays, and western blot analysis all revealed a marked decrease in viral replication compared to WT cells (Fig 1J).*”

We also treated Vero-E6 cells with the inhibitor 20α-HC after SARS-CoV-2 infection. Results from IFA and viral titer assays showed that this inhibitor markedly suppressed viral replication (Fig. 5J, revised version).

A related description of these results has been added to the revised manuscript at lines 292-294，as follows: “*Similarly, in Vero-E6 cells treated with 30 µM 20α-HC (Fig S5E) and infected with SARS-CoV-2, a marked inhibition of viral replication was observed (Fig 5J).*”

Figure 1

Figure 5

## Major points (2):

In Figure 1, the authors make claims that GRAMD1C depletion (KO) affects viral replication. However, in Figure S2A they show that this KO virtually depleted the viral receptor ANPEP from the cells. If receptor expression is so much reduced by GRAMD1C KO, how can the authors exclude that the phenotypes see in Fig. 1 are not caused by reduced receptor expression? Notably, the effect of KO on SARS-CoV-2 is very small whereas the effect on the porcine viruses is very strong. Therefore, the expression of ANPEP needs to be confirmed in all used porcine cell lines given the role of ANPEP in the entry of at least TGEV and PDCoV (https://doi.org/10.1016/j.virol.2019.12.007) and the same needs to be done for the MHV and SARS-CoV-2 receptors. Although the subsequent data show that RNA replication appears to be the main target of GRAMD1C, I find it disturbing to first show the data in Fig. 1 and then revealing that the KO has such a strong effect on receptor expression and thus, (also) virus entry.

RESPONSE:

Thank you for bringing this issue to our attention. To exclude the possibility that the phenotypes observed in Figure 1 resulted solely from reduced receptor (ANPEP) expression, we rescued ANPEP expression in GRAMD1C-KO cells (GRAMD1C-KO + ANPEP), restoring ANPEP expression to WT levels. When we repeated the infection assays using these reconstituted cells, a strong antiviral phenotype persisted, as evidenced by qPCR, viral titer, and IFA (Fig. 2D-F, revised version). This confirms that the antiviral phenotype is mainly caused by the loss of GRAMD1C itself, rather than by indirect downregulation of the viral receptor.

We examined the expression levels of *ANPEP*, *ACE2* and *CEACAM1* in GRAMD1C-KO Caco-2, GRAMD1C-KO Vero-E6 and GRAMD1C-KO L929 cells, respectively. RT-qPCR analysis revealed only a minimal decrease in ACE2 expression, whereas CEACAM1 levels remained unchanged (Fig. S2B-S2D, revised version). These findings also indicate that GRAMD1C primarily affects the viral replication stage.

A related description of these results has been added to the revised manuscript at lines 138-142, 158-161, as follows:

*To determine whether GRAMD1C's effect on viral receptors is specific to ANPEP, we measured the expression levels of ACE2 and CEACAM1 in GRAMD1C-KO Vero-E6 and GRAMD1C-KO L929 cells, respectively. RT-qPCR analysis revealed only a minimal decrease in ACE2 expression, whereas CEACAM1 levels remained unchanged (S2C and S2D Fig).*

*Consistent with this, infection of GRAMD1C-KO+ANPEP cells with live virus, followed by RT-qPCR, multi-timepoint viral titer assays, and IFA, showed that the antiviral phenotype persisted and was comparable to that in GRAMD1C-KO cells (Fig. 2D-2F).*

Figure 2

Supplementary Figure 2

## Major points (3):

Figure 2B: Why is there a further reduction in the double KO cells? The difference between ANPEP-KO and the double-KO is very moderate (2-3 fold) when comparing with the phenotypes reported in Figure 1 (100-fold) although with the BAC transfection, entry should have been bypassed. So why does depletion of the receptor impair so much viral replication and why is the difference between the single- and double-KO so minor? That needs to be explained.

RESPONSE:

Thank you for bringing this issue to our attention. In ANPEP-KO and double-KO (GRAMD1C and ANPEP), only one round of amplification occurred after transfection with TGEV-BAC. Even though GRAMD1C deletion significantly inhibited viral replication, the difference from the control was less distinct, resulting in a relatively modest phenotype.To address this, we repeated the TGEV-BAC transfection experiment with increased plasmid amounts and extended transfection times. After optimizing these conditions, the difference between double KO (ANPEP and GRAMD1C) and single ANPEP KO became more evident (Fig. 2B, revised version).

Additionally, we performed TGEV-BAC transfection in WT cells and in GRAMD1C-KO cells with ANPEP reconstituted to wild-type levels. In these settings, the presence of ANPEP allowed released viral particles to re-enter cells, which further amplified the difference between the groups (Fig. 2C, revised version).

Furthermore, we conducted qPCR, multi-timepoint viral titer assays, and IFA on the ANPEP-reconstituted cells (Fig. 2D-F, revised version). The results indicate that both in ANPEP deletion and reconstitution, GRAMD1C knockout significantly inhibited viral replication independently of the entry stage.

A related description of these results has been added to the revised manuscript at lines 149-152, 156-161, as follows:

*RT-qPCR assays showed that viral replication was further reduced in ANPEP-GRAMD1C-KO cells compared to ANPEP-KO cells (Fig 2B), confirming that GRAMD1C affects intracellular viral replication independently of viral entry.*

*Under these conditions, TGEV-BAC transfection in WT and GRAMD1C-KO+ANPEP cells revealed that GRAMD1C deficiency still significantly impaired viral replication (Fig. 2C).* *Consistent with this, infection of GRAMD1C-KO+ANPEP cells with live virus, followed by RT-qPCR, multi-timepoint viral titer assays, and IFA, showed that the antiviral phenotype persisted and was comparable to that in GRAMD1C-KO cells (Fig. 2D-2F).*

Figure 2

## Major points (4):

Moreover, a transfection control is missing in Figure 2 to exclude that the double-KO cells were indeed transfected. Otherwise, one cannot exclude that the reduction is due to reduced transfection efficiency, rather than the KO itself.

RESPONSE:

Thank you for bringing this issue to our attention. We confirmed the transfection efficiency between the two cell lines using two distinct methods: first by transfecting the pLVX-mCherry empty vector, and second by using the pRL-TK plasmid. (Fig. S4A and S4B, revised version). The results indicated that there was no difference in transfection efficiency between the two cell lines.

Supplementary Figure 4

## Major points (5):

Figure 2D: I find this data set not very meaningful, because replication is also very much reduced; and reduced replication also means less viral proteins and therefore, also less DMV. Only the data in Fig. 5 allow to draw the authors' conclusion. Either the authors add a statement to that or I suggest to remove this data from Figure 2.

RESPONSE:

Thank you for your valuable suggestion. We fully agree with your point that. To directly test whether GRAMD1C knockout affects DMVs biogenesis independently of viral replication and protein expression, we co-transfected TGEV nsp3 and nsp4 in WT and GRAMD1C-KO cells. The results showed that DMVs readily formed adjacent to the ER in WT cells, but were almost absent in GRAMD1C-KO cells (Fig. 2I, revised version), indicating that the loss of GRAMD1C directly impairs DMVs formation. We believe this new data directly addresses your concern and strengthens our conclusion. Therefore, we have retained the original Fig. 2D (Fig. 2H, revised version) as part of the broader phenotypic analysis while adding the new data in Fig. 2I.

A related description of these results has been added to the revised manuscript at lines 165-172, as follows: “*Transmission electron microscopy (TEM) at 12 hpi to visualize replication organelles showed that DMVs were scarcely detected in GRAMD1C-KO+ANPEP cells (Fig 2H). To test if GRAMD1C is directly required for DMV biogenesis, we co-transfected WT and GRAMD1C-KO cells with TGEV nsp3 and nsp4, which are sufficient to form viral DMVs [20, 21]. The results showed that DMVs readily formed adjacent to the ER in WT cells, but were almost absent in GRAMD1C-KO cells (Fig 2I), indicating that the loss of GRAMD1C directly impairs DMV formation.*”

Figure 2

## Major points (6):

From the data in Figure 3, the authors conclude that "Collectively, these results demonstrate that GRAMD1C engages coronavirus nsp4 via its TM domain to promote DMV biogenesis……". This is not formally shown, the only point that can be made is that membrane association of GRAMD1C is required to support viral replication. The authors cannot exclude that the TM is necessary to recruit the protein to the membrane, but the interaction occurs via some other regions in the protein. Therefore, one can only state that the TM domain is required for the interaction.

RESPONSE:

We thank the reviewer for this important point. We have revised the conclusion to be more precise. The relevant sentence has been added to the revised manuscript at lines 215-217, as follows: “*Collectively, these results demonstrate that the TM domain of GRAMD1C is required for its association with coronavirus nsp3/4 complexes and for promoting DMV biogenesis and viral replication.*”

## Major points (7):

How is the cholesterol recruitment timed with respect to DMV formation? The authors show an interaction of GRAMD1C with nsp4, which indicates a recruitment of GRAMD1C (and thereby cholesterol) to the DMVs. In the assembled DMVs, nsp4 is located mainly on the inner bilayer of the DMV, so how would GRAMD1C be able to exert its cholesterol-recruitment function? An interesting experiment would be analogous to Sandhu et al. (Figure 3/S4, DOI: 10.1016/j.cell.2018.08.033) to see whether cholesterol-dependent re-localization of GRAMD1C / Aster to the plasma membrane can still happen under conditions of DMV formation, or whether the nsp-associated GRAMD1C portion is "trapped" on the inner DMV bilayer.

RESPONSE:

We thank the reviewer for this insightful suggestion. We agree that this is a very interesting point to explore. We co-expressed GRAMD1C and TGEV-nsp3/4 in HEK-293T cells. After 24 h, cells were switched to DMEM containing 5% lipoprotein-deficient serum (LPDS), with or without 200 μM cholesterol/cyclodextrin (Fig. S6A, revised version). Our results show that in HEK-293T cells, GRAMD1C consistently co-localizes with TGEV-nsp3/4 in punctate structures, both under standard lipid-poor conditions (5% LPDS) and upon cholesterol loading (Fig. S6B, revised version). This indicates that once DMVs are formed, GRAMD1C is retained on the DMV membranes.

We have added these related results to the revised manuscript at lines 184-188, as follows: “*Notably, we observed that GRAMD1C consistently co-localized with TGEV-nsp3/4 in punctate structures under both lipid-poor (1% lipoprotein-deficient serum, LPDS) and cholesterol-loaded conditions, indicating its retention on DMV membranes following their formation (S6A and S6B Fig).*”

Supplementary Figure 6

## Major points (8):

Figure 4C: The authors show intense cholesterol signal in dsRNA punctae and claim specific cholesterol recruitment to replication sites. Since DMV formation goes hand in hand with significant membrane remodelling, this experiment does not indisputably support the authors' statement. Specificity of cholesterol recruitment rather than cholesterol accumulation as a result of membrane remodeling should be demonstrated either by staining an ER-resident lipid species that does not show this enrichment or by using nsp3/4 expressing cells, where upon GRAMD1C-KO the cholesterol enrichment in nsp4-stained punctae should be abrogated.

RESPONSE:

We thank the reviewer for raising this critical point. To test the specificity of cholesterol enrichment at viral replication compartments, we transfected PK-15 cells with an ER marker plasmid (pDsRed2-ER), then infected the cells with TGEV (Fig. 4C, revised version).

The results demonstrate that while cholesterol is enriched around dsRNA puncta, the ER marker signal does not show a corresponding aggregation at these same locations. This finding indicates that the observed cholesterol enrichment represents a specific recruitment to the replication compartment, rather than a consequence of non-specific ER membrane coiling.

We have therefore replaced the data in the original Figure 4C with these updated results. The corresponding text in the revised manuscript (lines 233-241) has been revised, as follows: “*PK-15 cells transfected with an ER marker (pDsRed2-ER) to label the ER, were infected with TGEV, and then stained for cholesterol (CY5-cholesterol) and viral dsRNA. Confocal imaging revealed specific, intense accumulation of cholesterol at dsRNA-positive puncta, whereas the ER marker did not cluster at these sites (Fig 4C). Quantitative colocalization analysis confirmed a strong spatial correlation between cholesterol-enriched domains and viral dsRNA, but not between these domains and ER. This indicates that cholesterol enrichment at replication organelles results from a targeted recruitment event, distinct from passive accumulation due to ER membrane remodeling.*”

Figure 4

## Minor points (1):

In the introduction, the authors frequently switch between different viruses, but make claims that read like being universally applicable to coronaviruses. For instance, the authors state: "As a key lipid component of ER membranes, cholesterol enriches at DMVs assembly sites to maintain membrane fluidity and curvature (18-20)." However, these references refer to hepatitis C virus and enteroviruses. This is just one example and there are several more of that (e.g. on APOE). The authors should be more specific when making such claims to which virus these data refer to.

RESPONSE:

We thank the reviewer for pointing out the inaccurate generalization in our introduction.

In the revised manuscript, we have carefully amended the relevant sections to clarify the relevant viruses for these findings. The related text has been revised at lines 68-74, 75-78, as follows:

*The role of cholesterol in the formation of replication organelles has been well-demonstrated for other viruses, such as hepatitis C virus (HCV) [22, 23] and Enteroviruses [24]. As a key lipid component of ER membranes, cholesterol enriches at DMV assembly sites to maintain membrane fluidity and curvature. Pharmacological targeting of cholesterol disrupts both disease progression and viral replication cycles, positioning cholesterol metabolism as a pivotal host-directed target against viral pathogenesis [25-27].*

*Recent studies utilizing CRISPR screening have identified several cholesterol-associated host factors in viral replication, including Niemann-Pick type C protein 1 (NPC1) and Sterol Regulatory Element-Binding Transcription Factor 2 (SREBF2) in coronaviruses [28, 29] and Apolipoprotein E (APOE) in influenza virus [30].*

## Minor points (2):

The same applies to used cell lines. They need to be properly described throughout the manuscript. For instance, Figure S2, which cell line is this?

RESPONSE:

Thank you for pointing out this issue. We apologize for this oversight. In response, we have revised the manuscript to ensure that all cell lines are now explicitly identified.

## Minor points (3):

The authors frequently report a "direct" interaction between nsp4 and GRAMD1C. However, the authors cannot exclude the interaction to be indirect, mediated via an adaptor protein. AlphaFold allows to make predictions, but they need to be confirmed experimentally and the pull-down data do not exclude an indirect interaction. Therefore, these statements need to be toned-down, mentioning just "interaction" and omitting "direct".

RESPONSE:

We thank the reviewer for this important comment. We have revised the manuscript accordingly. All claims of a “direct” interaction have been removed.

-The sentence in lines 87-89 was revised as “*Mechanistically, GRAMD1C is recruited to RTCs through its interaction with nsp4, enabling cholesterol flux necessary for DMV morphogenesis.*”

-The sentence in lines 188-189 was revised as “*Co-immunoprecipitation (**Co-IP) assays confirmed an interaction between GRAMD1C and TGEV-nsp4 (Fig. 3B).*”

## Minor points (4):

Figure 1E, G, I, Figure 5C: Please add quantification of Imaging Data.

The images are very small and thereby hard to evaluate (although the phenotypes displayed are admittedly very clear, but smaller differences as in 5C are not as clear).

RESPONSE:

We thank the reviewer for this constructive suggestion. In response, we have now performed quantitative analysis of the imaging data presented in Figure 1 and Figure 5 (revised version)

The detailed methodology for this quantification has been added to the manuscript (lines 712-722), as follows: “*Quantitative analysis of the percentage of positive cells was performed using ImageJ software. For each region of interest (ROI), images were converted to 8-bit grayscale. A consistent threshold was manually set to distinguish positive signal from background, and this threshold was applied uniformly to all images within the same experimental set. The “Analyze Particles” function was then used to count positively stained cells. The total number of cells in the same ROI was determined by DAPI. The percentage of positive cells was calculated as (Number of positive cells / Total number of cells) × 100%. For each condition, at least three independent biological replicates were analyzed, with multiple ROIs quantified per replicate. Data are presented as mean ± standard deviation (SD). Statistical significance was determined using two-tailed unpaired Student’s t-tests, with a p-value < 0.05 considered significant.*”

Figure 1

Figure 5

## Minor points (5):

Figure 2D and TEM analysis in other figures: Is it really number of DMV per cell? This would require a complete cross-section of the whole-cell volume; or is it per cell profile of view field? I did not find any statement how the TEM imagens have been quantified. That needs to stated.

RESPONSE:

We thank the reviewer for raising this important point. The detailed methodology for this quantification has been added to the manuscript (lines 748-750), as follows: “*TEM images were quantified for DMV abundance. For each condition, multiple random cell profiles were analyzed. Within the boundary of each entire single-cell profile, all DMVs were manually counted.*”

## Minor points (6):

Figure 3: What is the subcellular distribution of GRAMD1C expressed alone (i.e. without TGEV)?

RESPONSE:

We thank the reviewer for this relevant question. The result showing the subcellular distribution of GRAMD1C expressed alone has been included as a control in the revised manuscript (Fig. 3A, revised version).

Figure 3

## Minor points (7):

Figure 3A: What are the co-localization parameters for GRAMD1C and nsp3? By eye it is hard to identify a difference to the GRAMD1C co-localization with nsp4.

RESPONSE:

Thank you for this insightful observation. The parameters for GRAMD1C and nsp3 are as follows:

Pearson's correlation	Rr=	0.987622

Overlap coefficient	R =	0.996630

## Minor points (8):

Figure 3B: Was the pulldown performed under expression of nsp4 only or nsp3/4 co-expression? It would be very valuable to validate GRAMD1C preference for nsp4 over nsp3.

RESPONSE:

We thank the reviewer for this insightful suggestion. The Co-IP experiment presented in Figure 3B was conducted under conditions where only nsp4 was expressed. Following the reviewer's suggestion, we performed additional Co-IP experiments and found that GRAMD1C also interacts with nsp3 (Fig. S7A, revised version). However, due to the large size of the nsp3 protein, we were unable to reliably compare the binding affinities of GRAMD1C for nsp3 versus nsp4 in this assay. Determining whether GRAMD1C exhibits a binding preference for one protein over the other is an important question that needs further investigation in future studies.

The related text has been revised at lines 189-191, as follows: “*An interaction with nsp3 was also detected (S7A Fig). However, due to the large size of nsp3 which posed technical challenges, subsequent investigations were primarily focused on nsp4.*”

Supplementary Figure 7

## Minor points (9):

Figure 4: It would be helpful to include replication of naïve/control cells in order to judge the degree of rescue relative to cells without GRAMD1C KO.

RESPONSE:

Thank you for bringing this issue to our attention. We have included WT controls in the corresponding experiments to better evaluate the reconstitution. These data are now presented in Fig. 4G (revised version).

Figure 4

## Minor points (10):

Figure 4B: Why did the authors use an overexpression setting rather than the endogenous protein? That should be explained.

RESPONSE:

We thank the reviewer for raising this point. In the immunofluorescence experiments, we used epitope-tagged GRAMD1C overexpression because the available antibodies against endogenous GRAMD1C showed poor specificity and weak signal under our experimental conditions, making them unsuitable for clear and reliable detection.

We have now clarified this rationale in the revised manuscript at lines 180-182, as follows: “*To better visualize the subcellular localization of GRAMD1C, we exogenously overexpressed a FLAG-tagged GRAMD1C, instead of detecting the endogenous protein.*”

## Minor points (11):

Figure 4D: The authors use an imaging-based setting of cholesterol and conclude that "… indicating that cholesterol consumption is necessary for productive viral replication." However, this is not obvious from the imaging data. Therefore, a more biochemical approach such as TLC would be more appropriate.

RESPONSE:

We thank the reviewer for this insightful comment. We performed a biochemical measurement of free cholesterol levels using Free Cholesterol (FC) Content Assay Kit (Solarbio, BC1895) according to the manufacturer’s instructions. As shown in the Fig. 4E (revised version), viral infection led to a significant reduction in intracellular free cholesterol at 24 h post-infection.

The related description has been added to the manuscript in lines 245-250, as follows: “*To more directly assess alterations in intracellular cholesterol levels upon infection, we performed a biochemical measurement of free cholesterol using a Free Cholesterol Content Assay Kit. The results showed that viral infection led to a significant reduction in intracellular free cholesterol at 24 hpi (Fig 4E), indicating that cholesterol consumption is necessary for productive viral replication.*”

Figure 4

## Minor points (12):

Figure 4F: Can the authors rescue viral replication in GRAMD1C KO cells by the addition of cholesterol? This would be a most convincing way to demonstrate that cholesterol is responsible for the observed phenotype.

RESPONSE:

We thank the reviewer for this excellent suggestion. To directly test this hypothesis, we exogenously supplemented cholesterol to GRAMD1C‑KO PK‑15 cells (Response Figure 2A). The results clearly showed that cholesterol add‑back did not rescue TGEV replication (Response Figure 2B). We propose that this is because GRAMD1C is required for the proper trafficking of cholesterol to sites of DMVs biogenesis. In its absence, simply increasing cholesterol availability is insufficient. This hypothesis is further supported by our observation that total cellular free cholesterol levels are unchanged in KO cells(Response Figure 3A), but its subcellular distribution is altered (Response Figure 3B). Together, these data indicate that GRAMD1C facilitates viral replication not by modulating cholesterol abundance, but by mediating its spatial localization.

Response Figure 2

Response Figure 3

## Minor points (13):

The model in Figure 5J is nice, but I wonder whether it is correct that the TM of GRAMD1C really spans both ER lipid bilayer and the ER lumen as indicated.

RESPONSE:

We thank the reviewer for pointing out this issue. Indeed, the TM domain of GRAMD1C is unlikely to span the ER lumen. We have revised the positioning of the TM domain in the schematic diagram accordingly (Figure 5L, revised version).

Figure 5

## Minor points (14):

The discussion could be improved by considering earlier publications on cholesterol transport by OSBP and the entry inhibition by 24HC, and how this relates to the findings by the authors with 20α-HC.

RESPONSE:

We thank the reviewer for this valuable suggestion. We have now included a discussion of OSBP and 24HC in the revised manuscript at lines 370-377, 393-403, as follows:

*The involvement of non-vesicular lipid transport proteins in viral replication has been previously reported. The oxysterol-binding protein (OSBP), which exchanges cholesterol/phosphatidylinositol 4-phosphate (PI4P) at ER-Golgi interfaces, is recruited by SARS-CoV-2 nsp3, nsp4, and nsp6 to supply cholesterol to DMVs [46, 47]. While OSBP operates at the ER-Golgi interface, our work reveals GRAMD1C as a distinct host factor operating at PM - ER contact sites, highlighting a spatially complementary cholesterol hijacking pathway, which suggests coronaviruses can flexibly exploit multiple cellular cholesterol sources.*

*20α-HC, an inhibitor of GRAMD1C, exhibits structural similarity to the previously reported oxysterol 24HC. Both 24-HC and 20α-HC are cholesterol-derived oxysterols, but their distinct hydroxyl group positions confer different mechanisms and specificities. 24HC primarily disrupts OSBP function, elevating cholesterol levels in multivesicular bodies (MVBs) or late endosomes (LEs) and inhibiting viral entry [52]. In contrast, 20α-HC directly binds to the hydrophobic pocket of the ASTER domain of GRAMD1C, blocking its cholesterol transfer activity and impairing viral DMV formation [35]. This comparison illustrates how different oxysterols can selectively target distinct nodes within the host cholesterol transport network for antiviral effect. Although 20α-HC showed potent cellular activity, its reduced efficacy in vivo highlights the need for further pharmacokinetic optimization to advance its therapeutic potential.*
