# Supplementary material for: The non-vesicular cholesterol transporter GRAMD1C is a pan-coronavirus antiviral target
Source: PLoS Biol. 2026 Apr 6;24(4):e3003736. doi: 10.1371/journal.pbio.3003736 (PMC13068348; doi:10.1371/journal.pbio.3003736)
Supplement: Attachment — Submitted filename: response to reviewers-2.0.docx [file pbio.3003736.s022.docx]

**Response to reviewers**

**Reviewer #2 (Remarks to the Author):**

COMMENT:

In this revision, Su et al have well addressed most of my comments. The authors provide additional data addressing gaps and unclear aspects existing in the first version of the manuscript. Overall, I consider this a greatly improved version of the study that addresses a relevant and interesting aspect of coronavirus biology. Nevertheless, a few minor points need further consideration.

RESPONSE:

Thank you for your valuable feedback. In the following sections, you will find our responses to each of your points and suggestions.

## 1. Answer to major point 2:

"RT-qPCR analysis revealed only a minimal decrease in ACE2 expression, whereas CEACAM1 levels remained unchanged (Fig. S2B-S2D, revised version)": To call a 25% decrease "minimal" is an understatement considering the comparably weak reduction in SARS-CoV-2 replication. The authors still cannot exclude that reduced ACE2 expression contributes to the phenotype. Therefore, the authors should tone down their statement and indicate that while replication appears to be the primary effect of GRAMD1C, reduced viral entry as additional factor responsible for the phenotype cannot be excluded.

RESPONSE:

We thank the reviewer for the thoughtful suggestion. In response, we have revised the description of ACE2 expression changes and included a discussion to address this point more cautiously. The modified text can now be found at lines 141–142 and 399-403 as follows:

*RT-qPCR analysis revealed a decrease in ACE2 expression, whereas CEACAM1 levels remained unchanged (S2C and S2D Fig).*

*To determine which stage of TGEV proliferation is affected by GRAMD1C, we found that GRAMD1C influences both viral replication and entry. Specifically, loss of GRAMD1C downregulated the expression of ANPEP and ACE2. In the case of SARS-CoV-2, although GRAMD1C appears to primarily act on viral replication, we cannot exclude its contribution to viral entry.*

## 2. Regarding the same comment：

The authors state "… we rescued ANPEP expression in GRAMD1C-KO cells (GRAMD1C-KO+ ANPEP), restoring ANPEP expression to WT levels." However, this data (ANPEP expression level in the reconstituted cells in comparison to naïve cells) is not shown, but needs to be included.

RESPONSE:

We thank the reviewer for pointing this out. The relevant results have been included in Fig. S2A (revised version). A corresponding description has been added at lines 152–153, as follows:

*We also reconstituted ANPEP expression in the GRAMD1C-KO PK-15 cells (GRAMD1C-KO+ANPEP), restoring ANPEP expression to WT levels. (S2A Fig). Attachment and internalization assays confirmed that viral entry was restored to WT levels upon ANPEP complementation (S5A and S5B Fig).*

Supplementary Figure 2

## 3. Regarding major point 8:

I thank the authors for including this experiment in the manuscript. However, since the Pearson correlation coefficient for both conditions is very high (PCC of >0.7 is considered a strong correlation), bulk analysis of such samples is rarely robust. Therefore, the authors should consider to show exemplary line intensity profiles along a line drawn through the dsRNA puncta and demonstrating that ER signal decreases and cholesterol signal increases in this spot and showing 2 more exemplary cells in the supplement to underline the consistency of this phenotype.

RESPONSE:

We thank the reviewer for this valuable suggestion. In response, we have performed a line intensity profile analysis along lines drawn through individual dsRNA puncta (Fig. 4C, revised version). In addition to the representative example, we have included two additional exemplary cells in Fig. S9 (revised version) to demonstrate the consistency of this phenotype.

Figure 4

Supplementary Figure 9

## 4. Regarding minor points 6 and 7:

The correlation coefficient for GRAMD1C with single nsp3 and nsp4, respectively is similarly strong. This needs to be discussed since the authors claim nsp4-specificity, but taken together with their revised figure 3, it appears that nsp3 and 4 are equally responsible for GRAMD1C recruitment, which needs to be clearly stated and discussed.

RESPONSE:

We thank the reviewer for this insightful comment. Accordingly, we have revised the manuscript to clarify this point and avoid overstating nsp4 specificity. The relevant sentence has been added to the revised manuscript at lines 190-192, 373-378, as follows:

*An interaction with nsp3 was also detected (S7A Fig). Together with the colocalization analysis, these results suggest that GRAMD1C may bind to nsp3 and nsp4 with comparable affinity.*

*These findings position GRAMD1C, through its interaction with nsp3 and nsp4, as a facilitator that establishes ER-DMV contact sites to exploit host cholesterol for viral replication. Notably, the co-localization and interaction patterns of GRAMD1C with nsp3 were similar to those with nsp4, indicating a potential cooperative role for nsp3 and nsp4 in GRAMD1C recruitment.*

## 5. Regarding minor point 12:

This is an important result and the authors should consider to include it into the manuscript. However, this would require two positive controls: TGEV in WTcells without GRAMD1C KO +/- cholesterol and proper image quantification.

RESPONSE:

We thank the reviewer for this helpful suggestion. We have now included the requested experiment in the manuscript. The relevant sentence has been added to the revised manuscript at lines 263-270, as follows:

*To further demonstrate that cholesterol is responsible for the observed phenotype, we exogenously supplemented cholesterol in GRAMD1C-KO PK-15 cells to assess whether viral replication could be restored. IFA results showed that exogenous cholesterol supplementation indeed enhanced viral replication in WT PK-15 cells; however, it failed to restore viral replication in GRAMD1C-KO cells (Fig 4H). We propose that this is because GRAMD1C is required for the proper trafficking of cholesterol to sites of DMV biogenesis. In its absence, simply increasing cholesterol availability is insufficient.*

Figure 4
